# Synthesis of 1,3-disubstituted bicyclo[1.1.0]butanes *via* directed bridgehead functionalization†

**DOI:** 10.1039/d1sc01836a

**Published:** 2021-04-27

**Authors:** Ryan E. McNamee, Marius M. Haugland, Jeremy Nugent, Rachel Chan, Kirsten E. Christensen, Edward A. Anderson

**Affiliations:** Chemistry Research Laboratory 12 Mansfield Road Oxford OX1 3TA UK edward.anderson@chem.ox.ac.uk

## Abstract

Bicyclo[1.1.0]butanes (BCBs) are increasingly valued as intermediates in ‘strain release’ chemistry for the synthesis of substituted four membered rings and bicyclo[1.1.1]pentanes, with applications including bioconjugation processes. Variation of the BCB bridgehead substituents can be challenging due to the inherent strain of the bicyclic scaffold, often necessitating linear syntheses of specific BCB targets. Here we report the first palladium catalyzed cross-coupling on pre-formed BCBs which enables a ‘late stage’ diversification of the bridgehead position, and the conversion of the resultant products into a range of useful small ring building blocks.

## Abstract 2

Bicyclo[1.1.0]butanes (BCBs) are valuable precursors to four-membered rings and bicyclo[1.1.1]pentanes, and useful bioconjugation agents. We describe a versatile approach to access 1,3-disubstituted BCBs, which are otherwise challenging to prepare.

## Graphical abstract

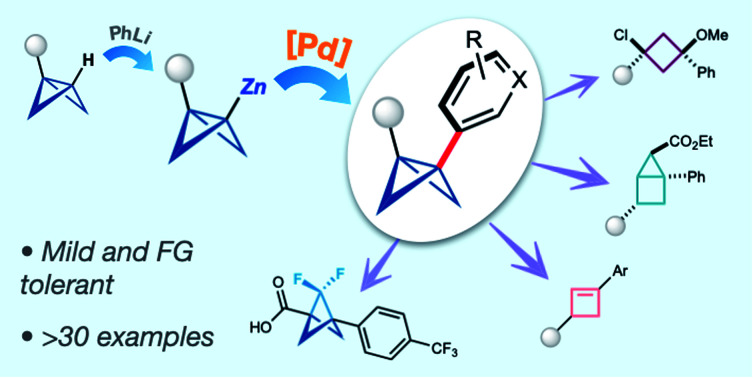

## Introduction

**Fig. 1:**
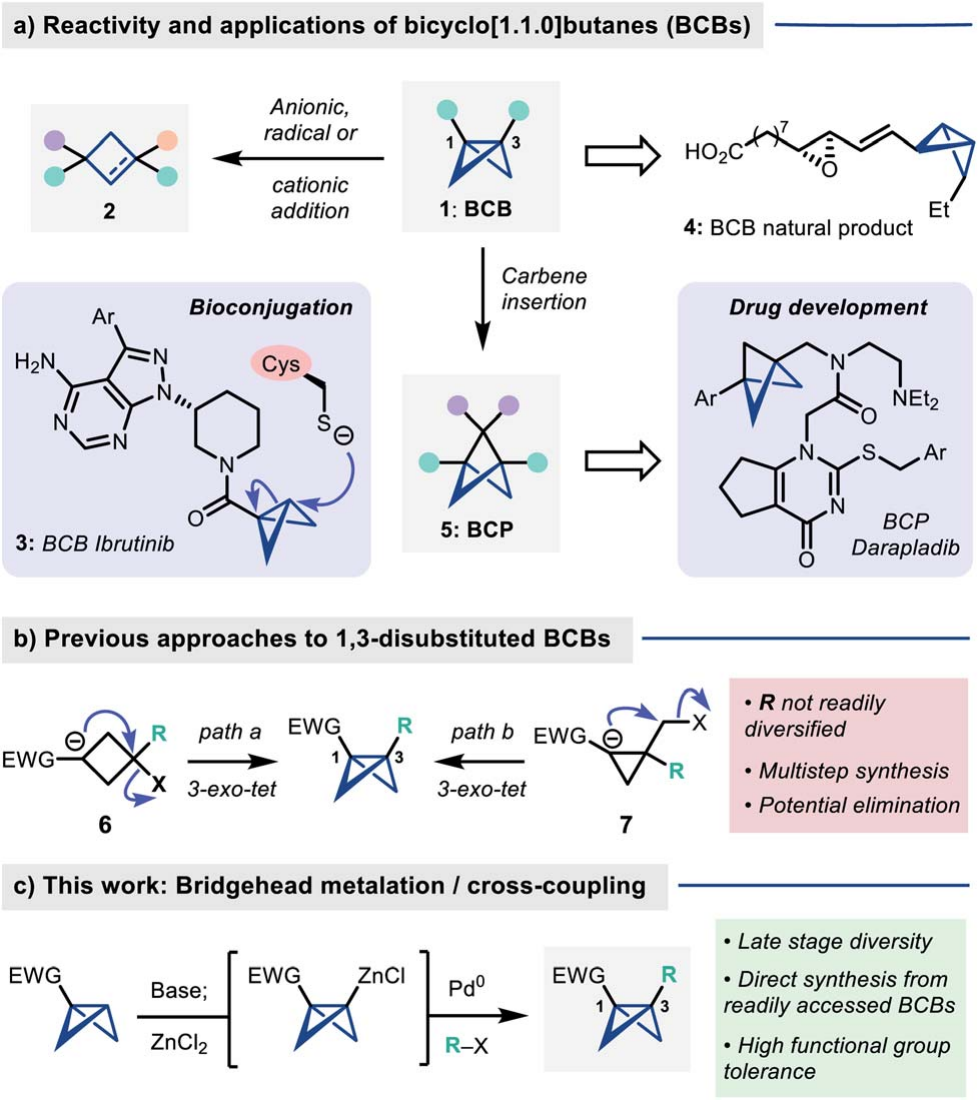
(a) Reactivity/applications of BCBs. (b) Existing routes to 1,3-disubstituted BCBs. (c) This work: Bridgehead cross-coupling.

Bicyclo[1.1.0]butanes (BCBs **1**, Fig. 1a) are a class of hydrocarbons consisting of two cyclopropane rings fused through a common C–C bond. The fused bicyclic structure is highly strained (ring strain energy ∼66 kcal mol^−1^, more than twice that of cyclopropane),^1^ which renders BCBs valuable intermediates in ‘strain release’ chemistry for the synthesis of four membered ring systems.^2,3^ In this context, BCBs that are monosubstituted at the bridgehead positions have been widely explored due to their ready accessibility^4–10^ and facility of ring opening reactions with nucleophiles,^4,11–14^ radicals^15,16^ and electrophiles^17–19^ to give cyclobutanes and cyclobutenes (**2**). BCBs have also recently found applications as tools for protein bioconjugation, where they exhibit high chemoselectivity for the alkylation of cysteine residues under mild conditions (*e.g.* BCB Ibrutinib, **3**).^10,20,21^ A BCB-containing natural product (**4**) has even been isolated and synthesised.^22,23^

1,3-disubstituted BCBs are attractive precursors to bicyclo[1.1.1]pentanes (BCPs, **5**), which are valuable motifs in drug design,^24–26^*via* a formal carbene insertion into the C1–C3 bond.^26–30^ However, access to 1,3-disubstituted BCBs is more challenging than their monosubstituted counterparts: the predominant routes consist of transannular 1,3-cyclization of a cyclobutyl carbanion onto a tertiary halide (**6**, Fig. 1b),^25,28,31–33^ which can suffer from competing elimination to a cyclobutene, or 3-*exo-tet* cyclization of a cyclopropyl carbanion onto a halomethyl substituent (**7**).^34^ Both require multistep synthetic sequences which limits facile bridgehead diversification.

We questioned whether direct introduction of a bridgehead substituent might be possible from a pre-formed mono-substituted BCB (Fig. 1c). Previous work has shown that selective deprotonation of this position is possible,^12,35^ and in specific tricyclic systems has been used for cross-coupling reactions,^36–38^ however the use of this strategy for the general synthesis of 1,3-difunctionalized BCBs is, to our knowledge, unknown. Here we report the development of a directed metalation/Negishi cross-coupling^39,40^ route to access these useful building blocks. The reaction proceeds under mild conditions and displays broad scope and functional group tolerance with respect to the electrophile, delivering an array of bio-relevant BCBs. These products can be readily transformed into a range of other polysubstituted small ring building blocks.

## Results and discussion

### BCB metalation

We first investigated deprotonation/deuterium quenching of the C3-bridgehead position of BCBs **8a–d**, featuring various directing groups (Table 1). Pleasingly, sulfone **8a** could be fully deuterated using PhLi or *n*-BuLi as base (Table 1, entries 1 and 2). While sulfoxide **8b** could direct deprotonation using lithium amide bases (entries 3 and 4), lithium-sulfoxide exchange (to form **9**) was found to be a competing pathway. Reaction of amide **8c** proved more fruitful: complete deprotonation was achieved using PhLi or *n*-BuLi at −78 °C (entries 5 and 6). However, metalation of ester **8d** was unsuccessful or resulted in nucleophilic addition to the carbonyl to give **10** (entries 7 and 8).

**Table 1:** Directed metalation studies on BCBs **8a–d**^a^

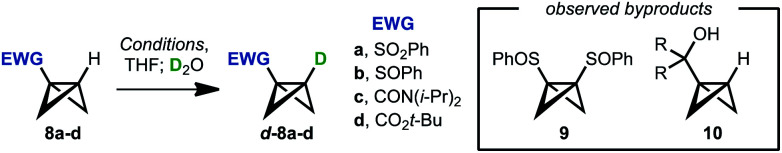				
Entry	BCB	Conditions	Equiv. base	**d-8** (conversion, %)^b^
**1**	**8a**	**PhLi, −78 °C**	**1.1**	**100**
**2**	**8a**	***n*-BuLi, −78 °C**	**1.1**	**100**
3	**8b**	LiTMP, 0 °C or −78 °C	3.0	65–72^c^
4	**8b**	LDA, −78 °C	3.0	75^c^
**5**	**8c**	**PhLi, −78 °C**	**1.1**	**100**
**6**	**8c**	***n*-BuLi, −78 °C**	**1.1**	**100**
7	**8d**	Mg(TMP)_2_, LiCl, 0 °C	1.1	n.r.^d^
8	**8d**	*n*-BuLi, −78 °C	1.0	50^e^

a Reactions conducted on 0.1 mmol scale, quenched with D_2_O at the indicated temperature.

b Conversion based on ratio of **d-8** : **8** as determined by ^1^H NMR spectroscopic analysis of the crude reaction mixture.

c Conversion as judged by integration of C3 in **8b***vs.* C2/C4 integration of **8b**, **d-8b** and **9** in the ^1^H NMR spectrum of the crude reaction mixture; analysis complicated by diastereomers of **9**.

d n.r. = no reaction.

e 1 : 1 mixture of **8d** and **10**. See the ESI for full details of optimization.

### BCB cross-coupling

With sulfone and amide directing groups successfully identified, cross-coupling of the resultant carbanions was addressed. The use of palladium catalysis for the synthesis of functionalized BCBs could potentially be problematic due to competing rearrangements in which Pd(ii) species promote electrophilic cleavage of the C1–C3 bond to give ring-opened diene products,^41–43^ a process that is of value in the synthesis of functionalized cyclobutanes.^17^ We anticipated that the pi-acidity of palladium complexes could be lowered by the use of electron-rich ligands, enabling the selective cross-coupling of metalated BCBs while avoiding rearrangement. We also conjectured that rapid transmetalation might further limit the potential of Pd(ii) intermediates to effect ring-opening processes, and thus targeted the use of a BCB-zinc species, formed by treatment of lithiated **8a** (generated using *n*-BuLi) with zinc chloride. The organozinc reagent, which could be isolated and is stable at room temperature for several hours,^44^ was then heated to 60 °C with Pd(dba)_2_ (5 mol%), trifurylphosphine (tfp, 10 mol%) and iodobenzene in THF. Pleasingly, the desired 1,3-disubstituted BCB **11** was obtained in excellent yield (Table 2, entry 1, 72%), with no isomerization to the cyclobutene **12** observed in the ^1^H NMR spectrum of the crude reaction mixture. However, substantial isomerization to **12** (>50%, <1 h) occurred on standing in deuterated chloroform which had not been pre-treated with potassium carbonate, indicating that acid-promoted isomerization of **11** is facile, in particular when a benzylic cation is generated. The use of Pd(PPh_3_)_4_, or the less electron-rich SPhos ligand proved less effective unless employed at higher catalyst loading (entry 3), while Pd(ii) catalysts indeed resulted in low conversion and/or competing isomerization of **11** to **12** (entries 5 and 6). Variation of solvent and temperature (entries 8–11) showed optimal conversion was achieved in THF at 40 °C. The use of PhLi in the metalation/coupling sequence further improved the yield (98%, entry 12), and was taken forward as the optimized conditions due to its less hazardous and more functional group tolerant nature.

**Table 2:** BCB cross-coupling optimization^a^

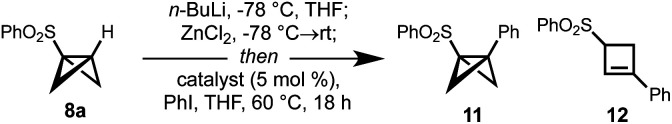				
Entry	Catalyst	Solvent	Temp (°C)	**11** b (%)
1	Pd(dba)_2_/2 tfp	THF	60	72 (75)
2	Pd(PPh_3_)_4_	THF	60	47 (50)
3^c^	Pd(PPh_3_)_4_	THF	60	81 (83)
4	Pd(dba)_2_/2 SPhos	THF	60	n.d.^d^ (13)
5	Pd(dppf)Cl_2_	THF	60	n.d. (5)
6	Pd(PhCN)_2_Cl_2_	THF	60	n.d. (6)^e^
7	Pd(*t*-Bu_3_P)_2_	THF	60	n.d. (18)^f^
8	Pd(dba)_2_/2 tfp	1,4-Dioxane	60	n.d. (23)
9	Pd(dba)_2_/2 tfp	DMF	60	n.d. (47)
10	Pd(dba)_2_/2 tfp	THF	40	89 (n.d.)
11	Pd(dba)_2_/2 tfp	THF	rt	n.d. (67)
**12** g	**Pd(dba)**_**2**_ **/2 tfp**	**THF**	**40**	**98 (100)**

a Cross-couplings run on 0.1 mmol scale with 1.0 equiv. of PhI.

b Isolated yields. Values in parentheses indicate conversion based on the ratio of **11** to **8a** and **12** as determined by ^1^H NMR spectroscopic analysis of the crude reaction mixture.

c 10 mol% catalyst.

d n.d. = not determined.

e 29% conversion to **12**.

f 9% conversion to **12**.

g 0.25 mmol scale using **8a** (1.2 equiv.), PhLi (1.2 equiv.) and PhI (1.0 equiv.). tfp = 2-trifurylphosphine. rt = room temperature.

**Scheme 1:**
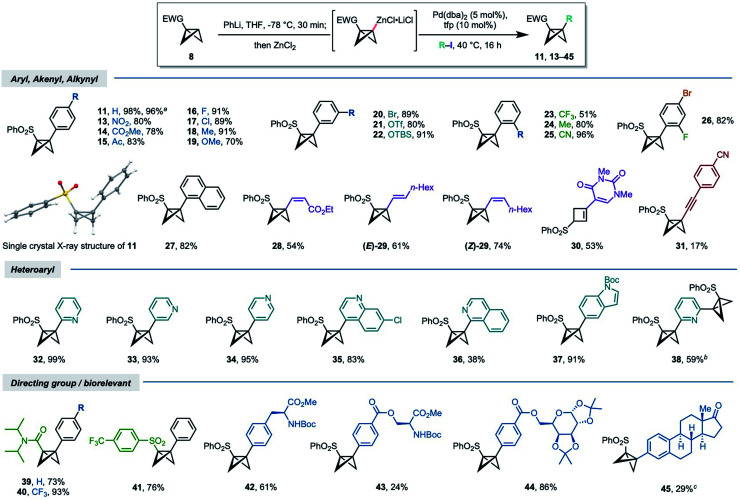
Synthesis of 1,3-disubstituted BCBs: reaction scope. Reaction conditions unless stated otherwise: **8a** or **8c** (0.30 mmol, 1.2 equiv.), PhLi (0.30 mmol, 1.2 equiv. 1.6–1.8 M in Bu_2_O), THF, −78 °C, 15 min; ZnCl_2_ (0.30 mmol, 1.2 equiv.), THF, −78 °C to rt; Pd(dba)_2_ (5 mol%), tfp (10 mol%), R–I (0.25 mmol, 1.0 equiv.) THF, 40 °C, 16 h. ^*a*^Reaction conducted with 8.76 mmol (1.70 g) of **8a**. ^*b*^0.48 equiv. of heteroaryl iodide. ^*c*^Reaction run using the aryl triflate/10 mol% Pd(PPh_3_)_4_ as catalyst. **11**, **14**, **16** and **18** were determined by single crystal X-ray diffraction studies.^45^ See the ESI† for details of unsuccessful substrates.

Having identified optimal conditions, we next assessed the scope of the reaction (Scheme 1). A series of aryl iodides bearing electron withdrawing and donating groups were first investigated; to our delight, these couplings proceeded in good to excellent yields (70–96%, **13–25**), irrespective of the nature of the arene substituent or its position (*para*, *meta*, *ortho*), with the exception of *o*-CF_3_ product **23** (51%).^45^ Many of these aryl-BCBs are hard to access *via* traditional routes, in particular for *ortho*-substituted aryl BCB products.^26,29^ The scalability of the chemistry was demonstrated through the successful synthesis of BCB **11** on >gram scale, with no decrease in yield (1.89 g, 96%). These disubstituted BCB products were found to be relatively stable, with resistance to rearrangement increasing with electron withdrawing character of the coupled aryl group; as noted above, little to no isomerization took place under the reaction conditions.

Trisubstituted aryl (**26**) and naphthyl (**27**) iodides also served as effective coupling partners, the former of which demonstrated useful selectivity for coupling of the aryl iodide (both 82% yield). Alkenyl iodides were similarly competent substrates, using either electron-poor (**28**, 54%) or electron-rich alkenes (**E**/**Z-29**, 61–74%). Interestingly, the product of coupling 5-iodo-1,3-dimethyluracil underwent rearrangement to cyclobutene **30** (53%). sp^3^–sp coupling using a 1-iodoalkyne proved more challenging, proceeding in low yield (**31**, 17%).

Heterocycle-substituted BCBs are highly appealing from a medicinal chemistry perspective. We were delighted to find that a range of azacycles could be successfully installed at the BCB bridgehead, including 2-, 3-, and 4-substituted pyridines (**32–34**), quinoline (**35**), isoquinoline (**36**), and indole (**37**) motifs. The yields for these couplings were uniformly high (83–93%) with the exception of isoquinoline **36** (38%). A double cross-coupling could even be achieved in relatively high yield using 2,6-diiodopyridine (**38**, 59%).

The use of other directing groups was briefly tested. The metalation/coupling chemistry was successfully applied to *N*,*N*-diisopropylamide BCB **8c**, which gave products **39** and **40** in good yields (73% and 93% respectively). Coupling of commercially available 4-trifluoromethylphenyl BCB sulfone (**41**, 76%) further underlined the potential for variation at this position. Finally, the use of more complex, bio-relevant coupling partners was explored: this includes the coupling of phenylalanine and serine derivatives (**42** and **43** respectively), and a galactose aryl ester (**44**). The aryl triflate of estrone also underwent coupling using Pd(PPh_3_)_4_ as the catalyst, albeit in low yield.

### BCB diversification

**Scheme 2:**
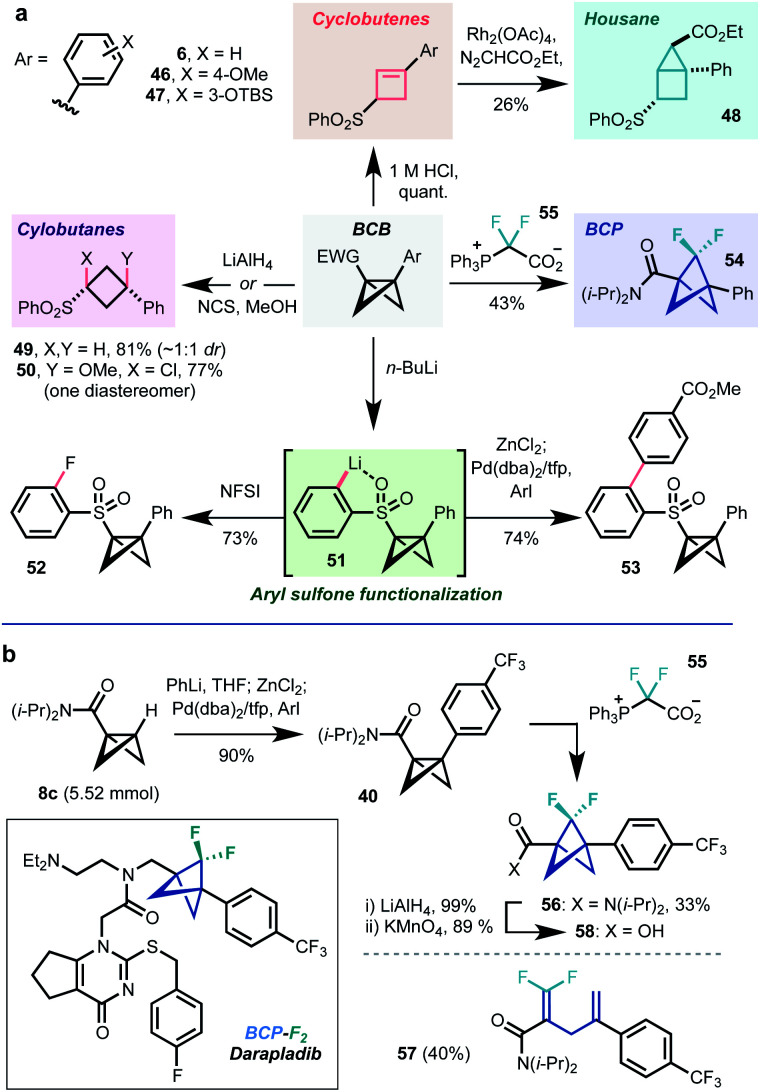
(a) Further transformations of 1,3-disubstituted BCBs. (b) Application to synthesis of the BCP-F_2_-darapladib sidechain.

1,3-disubstituted BCBs should offer useful opportunities in synthesis due to the potential for strain release. For example, the rapid BCB-to-cyclobutene rearrangement noted above for compound **30** could be effected in quantitative yield on subjection of chloroform solutions of BCBs to a catalytic amount of 1 M HCl (**6**, **46**, **47**, Scheme 2). Cyclobutene **12** could be further converted to [2.1.0]housane **48***via* Rh-catalyzed cyclopropanation. The central C1–C3 bond could also be reduced using LiAlH_4_ (**49**), or oxidised (NCS, MeOH, **50**) to cyclobutane derivatives. While the former transformation afforded a 1 : 1 mixture of diastereomers of **49**, presumably due to the formation of a configurationally labile α-sulfone carbanion intermediate, the latter gave exclusively *syn* addition of the two heteroatoms. The reasons for this are unclear, but may reflect a concerted addition process or single electron transfer mechanism.^46,47^

Having enabled the initial bridgehead functionalization, the sulfone functionality was found to be capable of directing further lithiation chemistry: treatment with *n*-BuLi led to metalation of the sulfone phenyl substituent (**51**), which could be trapped on reaction with electrophiles (NFSI, **52**) or subjected to transmetalation/Negishi cross-coupling (**53**). These transformations enable fine-tuning of the electronic properties of the sulfone substituent, which have been demonstrated to be important in strain release reactivity.^14^ We were also able to access the difluorobicyclo[1.1.1]pentane (BCP-F_2_) **54** on treatment with difluorocarbene precursor **55**, developed in our group. Collectively, these reactions show how BCBs represent a source of numerous small ring scaffolds.

As a final demonstration of potential utility in pharmaceutical research, we applied this chemistry to the synthesis of the BCP-F_2_ analogue of the atherosclerosis candidate darapladib, a compound with an existing BCP analogue.^25^ Gram scale lithiation of **8c**/cross-coupling delivered BCP **40** in 90% yield (1.35 g), treatment of which with difluorocarbene source **55** afforded the desired BCP-F_2_**56** in 33% yield (60% brsm) along with 40% of the undesired rearrangement product **57**. Notably, other difluorocarbene sources^26,29^ failed to deliver **56**. The *N*,*N*-diisopropylamide group in **56** could be transformed to the corresponding BCP-F_2_ acid **58***via* a two step reduction/oxidation sequence;^48^ the BCP analogue of this compound has been converted to BCP-darapladib in previous work.^25^

## Conclusions

In summary, we have developed a general cross-coupling strategy to access 1,3-disubstituted bicyclo[1.1.0]butanes featuring a wide variety of substituents at the 3-position. This approach minimizes the degree of BCB isomerization typically seen with transition metals through use of an electron-rich Pd(0) catalyst, and generally proceeds in high yields. The methodology enables the introduction of arenes and heteroarenes, and can be extended to scaffolds of importance in biology and medicine. In addition to the diversity of coupling partners, the functional group tolerance is notable: nitro, ester, ketone, halide, triflate, silyl, nitrile, ether, carbamate and acetal groups are all successfully accommodated, which augurs well for applications in medicinal chemistry. Further diversifications of the BCB products into other small ring systems, and synthesis of the BCP-F_2_ analogue of a darapladib fragment, highlight the utility of these ‘strain release’ precursors in synthesis.

## Author contributions

R. E. M. and E. A. A. conceived the work. R. E. M., M. M. H., J. N. and R. C. carried out the experimental work. K. E. C. acquired and solved the X-ray crystal structure. E. A. A. directed the work. All authors co-wrote the manuscript.

## Conflicts of interest

There are no conflicts to declare.

## Supplementary Material

SC-012-D1SC01836A-s001

SC-012-D1SC01836A-s002
