# Supplementary material for: Synthesis of 1,3-disubstituted bicyclo[1.1.0]butanes via directed bridgehead functionalization
Source: Chem Sci. 2021 Apr 27;12(21):7480–5. doi: 10.1039/d1sc01836a (PMC8171340; doi:10.1039/d1sc01836a)

Electronic Supplementary Material (ESI) for Chemical Science.
This journal is © The Royal Society of Chemistry 2021

Supporting Information

Synthesis of 1,3-Disubstituted Bicyclo[1.1.0]butanes via Directed Bridgehead Functionalization

Ryan E. McNamee, Marius M. Haugland, Jeremy Nugent, Rachel Chan, Kirsten E. Christensen and Edward A. Anderson*

Chemistry Research Laboratory, University of Oxford, 12 Mansfield Road,

Oxford, OX1 3TA, UK.

Contents

1. Experimental procedures 3
  1.1 General comments 3
  1.3 Synthesis of monosubstituted BCBs 4
  1.4 Deprotonation screen 11
  1.5 Coupling screen 12
  1.6 Synthesis of disubstituted BCBs 13
    1.6.1 Failed coupling reactions 34
  1.7 Diversification of BCBs 34
  1.8 Synthesis of coupling partners and reagents 42
2. X-Ray crystallography 48
  2.1 Crystal data and structure refinement for 11 collected at 300K 49
  2.2 Crystal data and structure refinement for 11 collected at 150K 50
  2.3 Crystal data and structure refinement for 14 51
  2.4 Crystal data and structure refinement for 16 52
  2.5 Crystal data and structure refinement for 18 53
3. References 54
4. Copies of NMR spectra 54

1. Experimental procedures

1.1 General comments

NMR Spectroscopy: Proton (^1H), carbon (^{13}C) and fluorine (^{19}F) NMR spectra were recorded on a Bruker AVIII HD 400, NEO 400, AVIII HD 500 or AVII 500 spectrometer. Proton, carbon and fluorine chemical shifts (δ) are quoted in parts per million (ppm). ^1H NMR spectra were recorded using an internal deuterium lock for the residual protons in chloroform-*d* (δ = 7.26) or benzene-d_6 (δ = 7.16). ^{13}C NMR spectra were recorded using an internal deuterium lock in chloroform-*d* (δ = 77.16) or benzene-d_6 (δ = 128.06). Assignments were determined either on the basis of unambiguous chemical shift or coupling patterns, COSY, HSQC, HMBC and/or NOESY experiments. Peak multiplicities are defined as s (singlet), d (doublet), t (triplet), q (quartet), m (multiplet) and br (broad). Coupling constants (*J*) are reported to the nearest 0.1 Hz.

Mass Spectroscopy: High-resolution mass spectra (HRMS) were recorded by the Departmental Mass Spectrometry Service, University of Oxford on a Thermo Scientific Exactive Mass Spectrometer (using a Waters Equity autosampler and pump) for electrospray ionization (ESI) and an Agilent 7200 Accurate Mass QTOF GCMS (using a SIM Direct Insertion Probe) for electron ionization (EI) and chemical ionization (CI). High resolution values are calculated to 4 decimal places from the molecular formula, and all values are within a tolerance of 5 ppm.

Infrared Spectroscopy: Infrared spectra were obtained as a thin film, by evaporation of a dichloromethane solution of the sample, on a diamond ATR module on a Bruker Tensor 27 FT-IR spectrometer. Wavelengths of maximum absorbance (ν_{max}) are quoted in cm^{-1}. Only selected, characteristic IR absorption data are provided for each compound.

Melting Points: Melting points were obtained using a Griffin melting point apparatus and are uncorrected.

Polarimetry: Optical rotations were recorded on a Perkin Elmer 241 or 341 polarimeter with a path length of 1 dm (using the sodium D line, 589 nm). $[\alpha]_D$ are reported in units of 10^{-1} deg cm^2 g^{-1}. Concentrations are reported in g/100 mL. Temperatures are reported in °C.

X-ray crystallography: Details of instrumentation and techniques are reported in Section 3.

Chromatography: Column chromatography refers to normal phase column chromatography and was performed on silica gel obtained from Merck (Silica gel Si 60, 0.040-0.063 mm) under a positive pressure of nitrogen, using the stated solvent system. Analytical thin-layer chromatography was performed on pre-coated aluminium-backed plates (Merck Kieselgel 60 F_{254} plates) with visualization by ultraviolet light (254 nm) and/or by staining with phosphomolybdic acid and potassium permanganate. Retention factors (R_f) are reported with the solvent system in parentheses.

Materials/procedures: All air- or moisture-sensitive reactions were carried out with anhydrous solvents in flame-dried glassware under an inert atmosphere of argon or nitrogen. Light sensitive reactions were carried out under aluminium foil protection. Heating was performed using an oil. Dry tetrahydrofuran (THF), DCM, pyridine (py) and diethyl ether (Et_2O) were collected fresh from an mBraun SPS-800 solvent purification system, having been passed through anhydrous alumina columns. All other commercially available reagents and solvents, where appropriate, were dried and purified before use using standard procedures.

1.3 Synthesis of monosubstituted BCBs

(But-3-en-1-ylsulfonyl)benzene, S1

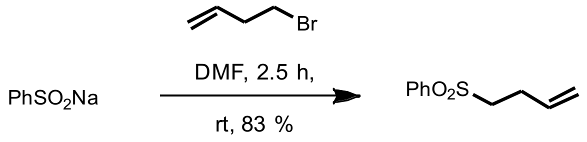

To a suspension of sodium benzenesulfinate (5.00 g, 30.5 mmol, 1.0 eq.) in DMF (50 mL) was added 4-bromobutene (3.70 mL, 36.5 mmol, 1.2 eq.) dropwise at rt. The reaction mixture was heated to 60 °C and stirred for 2.5 h before diluting with water (150 mL) and extracting with EtOAc (150 mL x 3). The organics were combined, washed with 5% aq. LiCl solution (50 mL x 3), dried with anhydrous Na_2SO_4, filtered and evaporated in vacuo to afford a yellow oil. The oil was purified via flash chromatography (10→20% EtOAc in pentane) to afford **S1** as a clear oil (5.00 g, 25.4 mol, 83%). Data identical to literature values.[1]

R R_f = 0.24 (20% EtOAc in pentane)

^{1}H NMR (400 MHz, Chloroform-*d*): δ 7.94-7.90 (m, 2H), 7.70-7.64 (m, 1H), 7.61-7.55 (m, 2H), 5.73 (ddt, *J* = 16.8, 10.2, 6.5 Hz, 1H), 5.09-5.02 (m, 2H), 3.20-3.14 (m, 2H), 2.51-2.42 (m, 2H)

^{13}C NMR (101 MHz, Chloroform-*d*): δ 139.2, 133.9, 133.9, 129.5, 128.7, 117.3, 56.1, 25.3

2-(2-(Phenylsulfonyl)ethyl)oxirane, S2

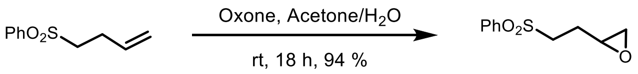

To a solution of **S1** (5.00 g, 25.4 mmol, 1.0 eq.) in acetone/water (1:1, 100 mL) was added $NaHCO_3$ (21.3 g, 0.25 mol, 10.0 eq.), followed by Oxone (20.3 g, 66.0 mmol, 2.6 eq.) at rt. The suspension was stirred for 18 h at rt before filtering, reducing *in vacuo* (removing acetone) and extracting with EtOAc (100 mL x 2). The organics were combined, dried with anhydrous Na_2SO_4, filtered and evaporated to afford a yellow oil. The oil was purified via flash chromatography (20→30% EtOAc in pentane) to afford **S2** as a clear oil (5.10 g, 23.8 mol, 94%). Data identical to literature values.[1]

R_f = 0.24 (40% EtOAc in pentane)

^{1}H NMR (400 MHz, Chloroform-*d*): δ 7.96-7.88 (m, 2H), 7.72-7.63 (m, 1H), 7.59 (ddt, *J* = 8.2, 6.7, 1.2 Hz, 2H), 3.29-3.16 (m, 2H), 3.01 (dtd, *J* = 6.7, 4.0, 2.6 Hz, 1H), 2.78 (dd, *J* = 4.8, 3.9 Hz, 1H), 2.50 (dd, *J* = 4.8, 2.6 Hz, 1H), 2.17 (dddd, *J* = 14.3, 8.7, 7.0, 4.2 Hz, 1H), 1.89-1.77 (m, 1H)

^{13}C NMR (101 MHz, Chloroform-*d*): δ 138.2, 134.1, 129.6, 128.2, 53.4, 50.8, 44.8, 26.0

(2-(Phenylsulfonyl)cyclopropyl)methanol, S3

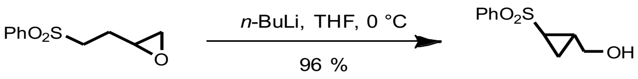

To a solution of **S2** (5.10 g, 23.8 mmol, 1.0 eq.) in THF (150 mL) was added *n*-BuLi (12.6 mL, 1.9 M in hexane, 23.8 mmol, 1.0 eq.) dropwise over 10 min at 0 °C. The reaction mixture was stirred for 15 min at 0 °C before quenching with sat. aq. NH_4Cl (5 mL). The mixture was partitioned between EtOAc (150 mL) and water (100 mL). The phases were separated and the aqueous phase was extracted with EtOAc (100 mL x 2). The combined organic phases were dried with anhydrous Na_2SO_4, filtered and concentrated to afford a yellow oil. The oil was purified via flash chromatography (50→70% EtOAc in pentane) to afford **S3** as a clear oil (4.90 g, 22.8 mmol, 96%). Data identical to literature values.[1]

$\boldsymbol{R_f}$ = 0.26 (80% EtOAc in pentane)

^{1}H NMR (400 MHz, Chloroform-*d*): δ 7.94-7.87 (m, 2H), 7.68-7.61 (m, 1H), 7.61-7.52 (m, 2H), 3.71 (dt, *J* = 11.2, 5.5 Hz, 1H), 3.54 (dt, *J* = 11.4, 5.7 Hz, 1H), 2.47 (dt, *J* = 8.3, 4.7 Hz, 1H), 2.11-2.00 (m, 1H), 1.54-1.45 (m, 2H), 1.10 (ddd, *J* = 8.3, 6.5, 5.4 Hz, 1H)

^{13}C NMR (101 MHz, Chloroform-*d*): δ 140.7, 133.6, 129.4, 127.7, 62.2, 37.0, 21.7, 10.2

(2-(Phenylsulfonyl)cyclopropyl)methyl methanesulfonate, S4

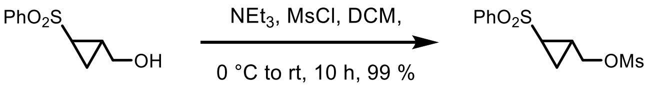

To a solution of **S3** (4.90 g, 22.8 mmol, 1.0 eq.) and NEt_3 (3.80 mL, 27.4 mmol, 1.2 eq.) in dichloromethane (150 mL) was added MsCl (2.10 mL, 27.4 mmol, 1.2 eq.) dropwise at 0 °C. The reaction mixture was stirred for 1 h at 0 °C, then warmed to rt and stirred for an additional 9 h. The reaction mixture was concentrated, and the residue dissolved in Et_2O (100 mL) and washed with water (50 mL x 2). The organic phase was dried with anhydrous Na_2SO_4, filtered and evaporated to yield **S4** as a yellow oil (6.60 g, 22.8 mmol, 99%). Data identical to literature values.[1]

^{1}H NMR (400 MHz, Chloroform-*d*): δ 7.95-7.88 (m, 2H), 7.71-7.64 (m, 1H), 7.63-7.54 (m, 2H), 4.30 (dd, *J* = 11.3, 5.9 Hz, 1H), 3.98 (dd, *J* = 11.3, 7.7 Hz, 1H), 2.95 (s, 3H), 2.59 (ddd, *J* = 8.5, 5.3, 4.3 Hz, 1H), 2.18 (ddtd, *J* = 9.5, 7.7, 6.1, 4.3 Hz, 1H), 1.66 (ddd, *J* = 9.5, 5.9, 5.3 Hz, 1H), 1.17 (dt, *J* = 8.5, 6.0 Hz, 1H)

^{13}C NMR (101 MHz, Chloroform-*d*): δ 140.2, 134.8, 128.8, 127.9, 69.0, 38.3, 38.0, 18.7, 11.4

1-(Phenylsulfonyl)bicyclo[1.1.0]butane, 8a

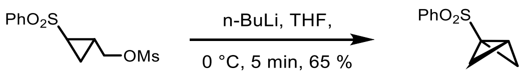

To a solution of **S4** (5.90 g, 20.4 mmol, 1.0 eq.) in THF (150 mL) was added *n*-BuLi (10.7 mL, 1.9 M in hexane, 20.4 mmol, 1.0 eq.) over 1 min at 0 °C.[A] The reaction mixture was stirred for 5 min at 0 °C before quenching with sat. aq. NH_4Cl (5 mL). The mixture was partitioned between Et_2O (100 mL) and water (100 mL), the phases were separated, and the aqueous phase was extracted with Et_2O (100 mL x 2). The organic phases were combined, dried with anhydrous Na_2SO_4, filtered and evaporated to afford a yellow oil. The oil was purified via flash chromatography (5→10% EtOAc in pentane) to afford **8a** as a colourless solid (2.60 g, 13.4 mmol, 65%). Data identical to literature values.[1]

Note A: *n*-BuLi must be added as a gentle stream to **S4**. If added too slowly the SM can polymerise.

R$_f$ = 0.26 (15% EtOAc in pentane), 0.23 (40% Et_2O in pentane)

^{1}H NMR (400 MHz, Chloroform-*d*): δ 7.97-7.93 (m, 2H), 7.66-7.60 (m, 1H), 7.61-7.52 (m, 2H), 2.57 (dq, *J* = 4.0, 2.7 Hz, 1H), 2.52 (dt, *J* = 3.7, 1.0 Hz, 2H), 1.39 (dt, *J* = 2.7, 0.9 Hz, 2H)

^{13}C NMR (101 MHz, Chloroform-*d*): δ 142.1, 134.8, 130.6, 127.3, 36.6, 23.2, 13.9

1,1-Dibromo-2-(chloromethyl)cyclopropane, S5

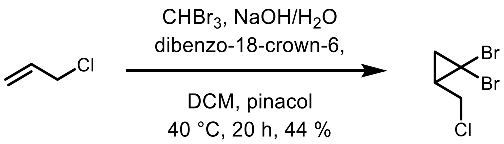

To a solution of dibenzo-18-crown-6 (1.80 g, 5.00 mmol, 0.05 eq.), allyl chloride (12.2 mL, 0.15 mol, 1.5 eq.), pinacol (0.47 g, 4.00 mmol, 0.04 eq.), and bromoform (8.80 mL, 0.10 mol, 1.0 eq.) in DCM (65 mL) was added a solution of NaOH in water (50% wt, 50 mL, 1.00 mol, 10.0 eq.). The reaction vessel was equipped with a reflux condenser, heated to 40 °C and stirred for 20 h. The reaction mixture was cooled, then poured into a beaker containing 200 mL of pentane, stirred and sonicated. The suspension was left to settle, and the solution was decanted. This was repeated twice followed by combining the organic extracts, filtering through a silica pad, and evaporating in vacuo to yield a yellow oil. The oil was purified via flash chromatography (pentane) to afford **S5** as a yellow oil (11.0 g, 0.44 mol, 44%). Data identical to literature values.[2]

R$_f$ = 0.28 (pentane)

^{1}H NMR (400 MHz, Chloroform-*d*): δ 3.65 (d, *J* = 7.4 Hz, 2H), 2.04 (dq, *J* = 10.3, 7.4 Hz, 1H), 1.93 (dd, *J* = 10.3, 7.5 Hz, 1H), 1.48 (t, *J* = 7.5 Hz, 1H)

^{13}C NMR (101 MHz, Chloroform-*d*): δ 46.3, 32.3, 29.1, 25.8

1-(Phenylsulfinyl)bicyclo[1.1.0]butane, 8b

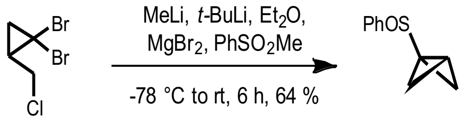

To a solution of **S5** (60 mg, 0.24 mmol, 1.2 eq.) in Et_2O (1.3 mL) was added methyllithium (1.4 M in Et_2O, 170 µL, 0.24 mmol, 1.2 eq.) dropwise at –78 °C. The reaction was stirred for 30 min at –78 °C followed by 1 h at –50 °C. The solution was then cooled back to –78 °C and a high vacuum was applied for 5 min (removing MeBr). *t*-BuLi (1.5 M in Et_2O, 160 µL, 0.24 mmol, 1.2 eq.) was then added dropwise and stirred for 20 min. A solution of freshly prepared $MgBr_2$•Et_2O[A] added via syringe. After stirring for 2 h at –78 °C, methyl 4-methylbenzenesulfinate (26 µL, 0.20 mmol, 1.0 eq.) was added dropwise and the reaction was stirred for 5 min at –78 °C, and finally for 30 min at rt. The solution was quenched with sat. aq. NH_4Cl (2 mL), the phases were separated, and the aqueous phases was extracted with Et_2O (5 mL). The combined organic phases were dried with anhydrous $MgSO_4$, filtered, and concentrated to afford a yellow oil. The oil was purified via flash chromatography (20→40% EtOAc in pentane) to afford **8b** as a light yellow oil (23 mg, 0.13 mmol, 64%). Data identical to literature values.[2]

Note A: $MgBr_2$•Et_2O was prepared during the 1 h stir at –50 °C as follows: To Et_2O (1 mL) containing magnesium turnings (47 mg, 1.9 mmol, 9.6 eq.) and was added dropwise 1,2-dibromoethane (42 µL, 0.50 mmol, 2.4 eq.) until reflux was initiated. The suspension was then cooled to 0 °C and the remaining 1,2-dibromoethane was added dropwise. After gas evolution had ceased, the mixture was stirred for an additional 30 min at rt.

^{1}H NMR (400 MHz, Chloroform-*d*): δ 7.69-7.65 (m, 2H), 7.48-7.46 (m, 3H), 2.37 (ddd, *J* = 6.1, 3.5, 1.5 Hz, 1H), 2.13 (ddd, *J* = 6.0, 3.5, 1.7 Hz, 1H), 2.08 (tt, *J* = 3.5, 2.3 Hz, 1H), 1.40 (dt, *J* = 2.6, 1.3 Hz, 1H), 1.19 (dt, *J* = 2.5, 1.3 Hz, 1H)

^{13}C NMR (101 MHz, Chloroform-*d*): δ 144.9, 131.0, 129.3, 124.6, 37.7, 34.0, 25.1, 8.7

3-Chlorocyclobutanecarboxylic acid, S6

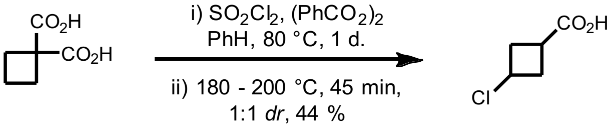

Sulfuryl chloride (34.1 mL, 0.42 mol, 1.05 eq.) was added dropwise via a dropping funnel over 30 min to a stirred solution of cyclobutane-1,1-dicarboxylic acid (57.7 g, 0.40 mol, 1.0 eq.) in benzene (500 mL) at 80 °C. During this addition was added, in two portion at 15 min internals, benzoyl peroxide (1.00 g, 4.10 mmol, 1 mol%) from the top of the condenser. The reflux condenser was fitted with a drying tube and stirred for 1 d at 80 °C. The reaction mixture was cooled and concentrated to yield a colourless solid, which was heated to 180-200 °C for 45 min. The resulting dark oil was distilled (90 °C, 2 mbar) to afford **S6** as a colourless oil (23.9 g, 0.18 mol, 44%, 1:1 *dr*).

^{1}H NMR (400 MHz, Chloroform-*d*): δ 4.61 (ttd, *J* = 7.6, 6.5, 1.0 Hz, 0.5H), 4.32 (tt, *J* = 8.6, 7.2 Hz, 0.5H), 3.45-3.30 (m, 0.5H), 3.30-3.15 (m, 0.5H), 2.98-2.77 (m, 2H), 2.68-2.53 (m, 2H)

^{13}C NMR (101 MHz, Chloroform-*d*): δ 181.4, 179.6, 51.0, 49.4, 47.7, 37.7, 37.1, 34.4

Data identical to literature values.[3]

***N*,*N*-Diisopropylbicyclo[1.1.0]butane-1-carboxamide, 8c**

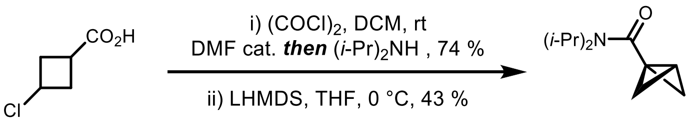

To a solution of **S6** (1.35 g, 10.0 mmol, 1.0 eq.) in DCM (30 mL) was added oxalyl chloride (1.00 mL, 12.0 mmol, 1.2 eq.) and DMF (2 drops) at rt. The reaction was stirred for 1 h at rt before concentrating in vacuo and placing under high vacuum for 5 min. The crude acyl chloride was dissolved in DCM (20 mL) and a solution of (*i*-Pr)$_2$NH (1.54 mL, 11.0 mmol, 1.1 eq.) in DCM (20 mL) was added slowly at rt. The solution was stirred for 1 d at rt, followed by washing with aq. HCl (1 M, 50 mL), sat. aq. $NaHCO_3$ (50 mL), and brine (50 mL). The organic phase was dried with anhydrous $MgSO_4$, filtered, concentrated and purified by a silica pad filtration (Et_2O/pentane, 1:1) to yield 3-chloro-*N*,*N*-diisopropylcyclobutanecarboxamide as a clear yellow oil (1.43 g, 6.60 mmol, 74%, 1:1 *dr*).

To a solution of 3-chloro-*N*,*N*-diisopropylcyclobutanecarboxamide (0.70 g, 3.24 mmol, 1.0 eq.) in THF (8 mL) was added a solution of LHMDS in THF (0.46 M, 8.0 mL, 3.65 mmol, 1.1 eq.) at 0 °C. The reaction was stirred for 4 h at 0 °C before diluting with DCM and quenching with a few drops of water. The mixture was evaporated in vacuo, and the residue was purified by flash column chromatography (1% NEt_3, 10→20% EtOAc in pentane) to afford **8c** as a colourless solid (0.28 g, 1.60 mmol, 43%).

R$_f$ = 0.29 (20% EtOAc in pentane)

MP: 55 °C

IR (thin film, ν_{max} / cm^{-1}): 2999, 1627, 1482, 1383, 1283

HRMS (ESI^+) calc. for $C_{11}H_{20}ON$ $[M+H]^+$ 182.1539, found 182.1541

^{1}H NMR (500 MHz, Chloroform-*d*): δ 4.86 (s, 1H), 3.41 (s, 1H), 2.16 (d, *J* = 3.3 Hz, 2H), 1.87 (tt, *J* = 3.4, 2.2 Hz, 1H), 1.50-1.31 (m, 6H), 1.30-1.12 (m, 6H), 1.05 (d, *J* = 2.2 Hz, 2H)

^{13}C NMR (126 MHz, Chloroform-*d*): δ 170.0, 49.6, 46.0, 36.5, 21.3, 20.9, 11.6, 9.5

Methyl bicyclo[1.1.0]butane-1-carboxylate, S7

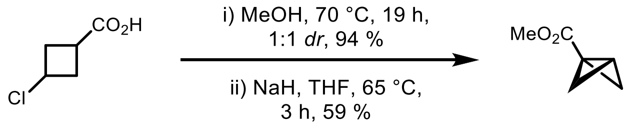

To a solution of **S6** (13.5 g, 0.10 mol, 1.0 eq.) in 2,2-dimethoxypropane (18 mL) was added a solution of methanesulfonic acid (50.0 µL, 0.77 mmol, 0.77 mol%) in MeOH (3 mL). The solution was heated at 70 °C for 19 h before being cooled to room temperature and diluted with Et_2O (150 mL). The organic phase was washed with sat. aq. Na_2CO_3 solution (100 mL), brine (100 mL), dried with anhydrous $MgSO_4$ and filtered. The organic phase was passed through a silica pad with a further 100 mL of Et_2O, and the filtrate was evaporated in vacuo to yield the intermediate methyl 3-chlorocyclobutanecarboxylate as a yellow oil (13.9 g, 93.5 mmol, 94%, 1:1 *dr*).

^{1}H NMR (400 MHz, Chloroform-*d*): δ 4.60 (ttd, *J* = 7.5, 6.4, 1.0 Hz, 0.5H), 4.30 (tt, *J* = 8.6, 7.2 Hz, 0.5H), 3.71 (s, 1.5H), 3.70 (s, 1.5H), 3.31 (ttd, *J* = 9.7, 5.3, 4.8, 1.0 Hz, 0.5H), 3.26-3.10 (m, 0.5H), 2.92-2.73 (m, 2H), 2.63-2.50 (m, 2H).

To a suspension of NaH (60% in mineral oil, 3.94 g, 98.4 mmol, 1.05 eq.) in THF (80 mL) was added a solution of methyl 3-chlorocyclobutanecarboxylate (13.9 g, 93.6 mmol, 1.0 eq.) in THF (20 mL) dropwise. The reaction was heated to reflux and stirred for 3 h. The mixture was then cooled to rt, diluted with Et_2O (200 mL), washed with water (100 mL), brine (100 mL) and dried with anhydrous $MgSO_4$. The organic phase was filtered and evaporated to yield a yellow oil. The oil was purified by distillation (39-41 °C, 13.0 mbar) to afford **S7** as a colourless oil (6.15 g, 50.4 mmol, 59%). Data identical to literature values.[3]

^{1}H NMR (400 MHz, Chloroform-*d*): δ 3.69 (s, 3H), 2.35 (dt, *J* = 3.5, 1.0 Hz, 2H), 2.07 (tt, *J* = 3.5, 2.8 Hz, 1H), 1.14 (dt, *J* = 2.8, 1.0 Hz, 2H)

^{13}C NMR (101 MHz, Chloroform-*d*): δ 173.6, 52.0, 35.6, 16.5, 9.0

Tert-butyl bicyclo[1.1.0]butane-1-carboxylate, 8d

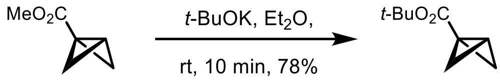

To a solution of **S7** (1.00 g, 8.92 mmol, 1.0 eq.) in Et_2O (100 mL) was added *t*-BuOK (2.00 g, 17.8 mmol, 2.0 eq.). The mixture was stirred for 10 min before filtering through a thin alumina bed and concentrating to yield **8d** as a clear oil (1.05 g, 6.81 mmol, 78%). Data identical to literature values.[4]

^{1}H NMR (400 MHz, Chloroform-*d*): δ 2.29 (dt, *J* = 3.4, 1.0 Hz, 2H), 1.97 (tt, *J* = 3.4, 2.8 Hz, 1H), 1.45 (s, 9H), 1.07 (dt, *J* = 2.8, 0.9 Hz, 2H)

^{13}C NMR (101 MHz, Chloroform-*d*): δ 172.2, 80.5, 35.5, 28.3, 15.9, 10.2

1.4 Deprotonation screen

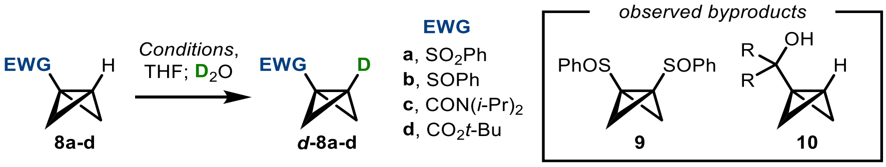

Entry	BCB	conditions	Base equiv.	***d*-8** (Conversion, %)[b]
1	**8a**	*n*-BuLi, -78 °C, 5 min	1.10	100
2	**8a**	PhLi, -78 °C, 0.5 h	1.10	100
3	**8a**	LiTMP, -78 °C, 2.0 h	2.00	100
4	**8a**	LDA, -78 °C, 2.0 h	2.00	42
5	**8b**	LiTMP, 0 °C, 2 h	3.00	72[b]
6	**8b**	LiTMP, -78 °C, 5.5 h	3.00	65[b]
7	**8b**	LDA, -78 °C, 3 h	3.00	75[b]
8	**8c**	PhLi, -78 °C, 0.5 h	1.10	100
9	**8c**	*n*-BuLi, -78 °C, 0.5 h	1.10	100
10	**8c**	*s*-BuLi, -78 °C, 0.5 h	1.10	23
11	**8c**	*t*-BuLi, -78 °C, 0.5 h	1.10	100
12	**8d**	$ZnCl_2$, then LiTMP, -78 °C[5]	1.10	0
13	**8d**	TMP_2Mg, LiCl, 0 °C to rt[6]	1.10	0
14	**8d**	*n*-BuLi, -78 °C, 5 min	1.00	0
15	**8d**	*t*-BuLi, -78 °C, 5 min	1.00	0

Table 1. [a] Reactions conducted on 0.1 mmol scale, quenched with D_2O at the indicated temperature; [b] Conversion based on ratio of ***d*-8**:**8** as determined by ^1H NMR spectroscopic analysis of the crude reaction mixture; [c] Conversion as judged by integration of C3 in **8b** vs. C2/C4 integration of **8b**, **8b** and **9** in the ^1H NMR spectrum of the crude reaction mixture; analysis complicated by diastereomers of **9**; [d] n.r. = no reaction; [e] 1:1 mixture of **8d** and **10**.

1.5 Coupling screen

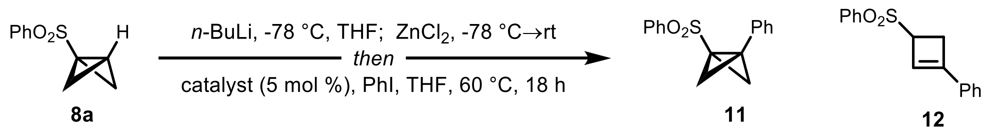

Entry	Catalyst	Solvent	Temp (°C)	**11** (%)[b]	**12** (%)[b]
1	$Pd(dba)_2$ / 2 tfp	THF	60	72	n.d. (0)
2	$Pd(PPh_3)_4$	THF	60	47 (50)	n.d. (0)
3[c]	$Pd(PPh_3)_4$	THF	60	81 (83)	n.d. (0)
4	$Pd(dba)_2$ / 2 SPhos	THF	60	n.d.[d] (13)	n.d. (0)
5	$Pd(dppf)Cl_2$	THF	60	n.d. (5)	n.d. (0)
6	$Pd(PhCN)_2Cl_2$	THF	60	n.d. (6)	n.d. (29)
7	$Pd(dba)_2$ / 2 $RuPhos)_2$	THF	60	n.d. (4)	n.d. (0)
8	$Pd_2(dba)_3$ / 4 tfp	THF	60	n.d. (68)	n.d. (0)
9	$Pd(dba)_2$ / 2 dpep	THF	60	n.d. (63)	n.d. (0)
10	$Pd(dba)_2$ / 2 Ptol	THF	60	n.d. (17)	n.d. (0)
11	$Pd(dba)_2$ / 2 dppf	THF	60	n.d. (0)	n.d. (0)
12	$Pd(dba)_2$ / 2 dppe	THF	60	n.d. (0)	n.d. (0)
13	$Pd(dba)_2$ / 2 dppp	THF	60	n.d. (0)	n.d. (0)
14	$Pd(dba)_2$ / 2 dppb	THF	60	n.d. (4)	n.d. (0)
15	$Pd(dba)_2$ / 2 dcpe	THF	60	n.d. (0)	n.d. (0)
16	$Pd(t\text{-}Bu_3P)_2$	THF	60	n.d. (18)	n.d. (9)
17	$Pd(dppf)_2Cl_2$	THF	60	n.d. (5)	n.d. (0)
18	$Pd(MeCN)_2Cl_2$	THF	60	n.d. (6)	n.d. (15)
19	$Pd(bpy)_2Cl_2$	THF	60	n.d. (2)	n.d. (9)
20	Pd2$(Allyl\text{-})_2Cl_2$	THF	60	n.d. (4)	n.d. (21)
21	$Pd(dba)_2$ / 2 tfp	1,4-dioxane	60	n.d. (23)	n.d. (0)
22	$Pd(dba)_2$ / 2 tfp	DMF	60	n.d. (47)	n.d. (0)
23	$Pd(dba)_2$ / 2 tfp	2-MeTHF	60	n.d. (0)	n.d. (0)
24	$Pd(dba)_2$ / 2 tfp	NMP/THF (1:1)	60	n.d. (0)	n.d. (0)
25	$Pd(dba)_2$ / 2 tfp	THF	60	n.d. (75)[g]	n.d. (0)
26	$Pd(dba)_2$ / 2 tfp	THF	40	89	n.d. (0)
27	$Pd(dba)_2$ / 2 tfp	THF	rt	n.d. (67)	n.d. (0)
**28**[f]	**$Pd(dba)_2$ / 2 tfp**	**THF**	**40**	**98**	n.d. (0)

Table 2. [a] Cross-couplings run on 0.1 mmol scale with 1.0 equiv. of PhI; [b] Isolated yields. Values in parentheses indicate conversion based on the ratio of **11** to **8a** and **12** as determined by ^{1}H NMR spectroscopic analysis of the crude reaction mixture; [c] 10 mol% catalyst; [d] n.d. = not determined; [e] 2.0 eq. of PhI; [f] 0.25 mmol scale using **8a** (1.2 eq.), PhLi (1.2 eq.) and PhI (1.0 eq.). tfp = 2-trifurylphosphine. rt = room temperature.

1.6 Synthesis of disubstituted BCBs

General BCB Cross-coupling Procedure

To a solution of BCB (0.30 mmol, 1.2 eq.) in THF (0.40 mL) was added PhLi (1.6-1.8 M in *n*-Bu_2O, 0.19-0.17 mL, 1.2 eq.) dropwise at –78 °C. The mixture was stirred for 30 min, then a solution of $ZnCl_2$ (41 mg, 0.30 mmol, 1.2 eq.) in THF (0.5 mL) was added, and the reaction was stirred for 5 min at –78 °C before bringing to rt, and stirred for a further 5-10 min. The solution of organozinc was transferred via syringe to a vial containing $Pd(dba)_2$ (7.5 mg, 13.0 µmol, 5 mol%), trifurylphosphine (tfp, 5.8 mg, 25.0 µmol, 10 mol%) and the coupling partner[A] (0.25 mmol, 1.0 eq.). The reaction mixture was stirred for 1 d at 40 °C, then it was diluted with DCM or Et_2O (5 mL), washed with water (5 mL), dried with anhydrous $MgSO_4$ and filtered. The filtrate was evaporated in vacuo and the residue purified by flash chromatography[B] with an appropriate gradient.[C]

Note A: If coupling partner is a solid, or liquid with unknown density, it was added to the vial before addition of the organozinc. If the liquid had a known density it was added after addition of the organozinc.

Note B: Compounds found to readily isomerise to their respective cyclobutenes should be chromatographed on neutralised silica (1% NEt_3 with eluting solvent) and analysed (NMR) in benzene-*d*. Such compounds include: 18, 19, 20, 22, 24, 26, (*E/Z*)-29, 37, 39, 40, 42 and 45.

Note C: If column chromatography does not afford sufficient purity, trituration with Et_2O was found to be effective at further purifying products. Crystallisations were also performed by layering Et_2O solutions of BCBs with pentane, or heating and cooling BCB solutions in Et_2O.

1-Phenyl-3-(phenylsulfonyl)bicyclo[1.1.0]butane, 11

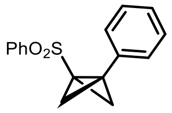

Prepared according to the general procedure using iodobenzene (28 µL, 0.25 mmol, 1.0 eq.) as coupling partner. The title compound was purified by flash chromatography (20→40% Et_2O in pentane), and isolated as a colourless solid (66 mg, 0.24 mmol, 98%).

Large scale: **8a** (1.70 g, 8.76 mmol, 1.20 eq.), PhLi (1.8 M in *n*-Bu_2O, 4.87 mL, 8.76 mmol, 1.20 eq.), $ZnCl_2$ (1.19 g, 8.76 mmol, 1.20 eq.), $Pd(dba)_2$ (0.21 g, 0.38 mmol, 5 mol%), tfp (0.17 g, 0.73 mmol, 10 mol%) and iodobenzene (0.82 mL, 7.30 mmol, 1.0 eq.) afforded **11** as a light yellow solid (1.89 g, 7.00 mmol, 96%).

$\boldsymbol{R_f}$ = 0.28 (40% Et_2O in pentane)

MP: 119 °C (recrystallized from Et_2O)

IR (thin film, ν_{max} / cm^{-1}): 3061, 2924, 2852, 1483, 1306, 1147, 879, 760, 727, 691, 624

HRMS (ESI^+) calc. for $C_{16}H_{14}O_2{}^{23}Na^{32}S$ $[M+Na]^+$ 293.0607, found 293.0608

^{1}H NMR (400 MHz, Chloroform-*d*): δ 8.19 (d, *J* = 8.9 Hz, 2H), 7.75 (d, *J* = 8.5, 1.2 Hz, 1H), 7.61 (tt, *J* = 7.5, 1.2 Hz, 2H), 7.35-7.23 (m, 5H), 2.90 (s, 2H), 1.66 (s, 2H)

^{13}C NMR (101 MHz, Chloroform-*d*): δ 140.3, 133.2, 130.5, 129.0, 128.6, 127.6, 127.5, 127.1, 35.5, 34.8, 31.0

1-(4-Nitrophenyl)-3-(phenylsulfonyl)bicyclo[1.1.0]butane, 13

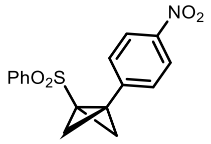

Prepared according to the general procedure using 1-iodo-4-nitrobenzene (62 mg, 0.25 mmol, 1.0 eq.) as coupling partner. The title compound was purified by trituration with Et_2O, and isolated as an off-white solid (63 mg, 0.20 mmol, 80%).

MP: 180 °C (recrystallized from DCM/Et_2O)

IR (thin film, ν_{max} / cm^{-1}): 1598, 1510, 1342, 1296, 1143, 878, 855, 758, 691, 634

HRMS (ESI^+) calc. for $C_{16}H_{14}O_4NS$ $[M+H]^+$ 32, 316.0638, found 316.0639

^{1}H NMR (400 MHz, Chloroform-*d*): δ 8.19 (d, *J* = 8.9 Hz, 2H), 7.75 (dd, *J* = 8.4, 1.3 Hz, 2H), 7.61 (tt, *J* = 7.5, 1.2 Hz, 1H), 7.53-7.46 (m, 4H), 3.06 (s, 2H), 1.80 (s, 2H)

^{13}C NMR (101 MHz, Chloroform-*d*): δ 147.2, 140.7, 139.3, 133.7, 129.3, 127.9, 127.5, 123.9, 37.1, 36.8, 30.1

Methyl 4-(3-(phenylsulfonyl)bicyclo[1.1.0]butan-1-yl)benzoate, 14

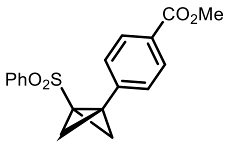

Prepared according to the general procedure using methyl 4-iodobenzoate (58 mg, 0.25 mmol, 1.0 eq.) as coupling partner. The title compound was purified by flash chromatography (30→50% Et_2O in pentane), and isolated as a colourless solid (64 mg, 0.20 mmol, 78%).

R_f = 0.19 (50% Et_2O in pentane)

MP: 141 °C (recrystallized from Et_2O)

IR (thin film, ν_{max} / cm^{-1}): 3062, 2951, 1718, 1609, 1279, 1253, 1118, 858, 773, 729, 629

HRMS (ESI^+) calc. for $C_{18}H_{17}O_4{}^{32}S$ $[M+Na]^+$ 329.0842, found 329.0842

^{1}H NMR (400 MHz, Chloroform-*d*): δ 7.96 (d, *J* = 8.5 Hz, 2H), 7.64 (dd, *J* = 8.4, 1.3 Hz, 1H), 7.54 (tt, *J* = 7.5, 1.2 Hz, 1H), 7.40 (t, *J* = 8.0 Hz, 2H), 7.34 (d, *J* = 8.5 Hz, 2H), 3.91 (s, 3H), 2.96 (s, 2H), 1.71 (s, 2H)

^{13}C NMR (101 MHz, Chloroform-*d*): δ 166.8, 140.2, 136.3, 133.4, 129.8, 129.1, 129.1, 127.6, 127.0, 52.2, 36.2, 36.0, 30.4

1-(4-(3-(Phenylsulfonyl)bicyclo[1.1.0]butan-1-yl)phenyl)ethan-1-one, 15

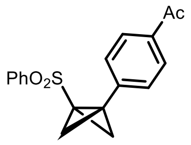

Prepared according to the general procedure using 1-(4-iodophenyl)ethan-1-one (62 mg, 0.25 mmol, 1.0 eq.) as coupling partner. The title compound was purified by flash chromatography (30→40% Et_2O in pentane), and isolated as a brown solid (65 mg, 0.20 mmol, 83%).

R$_f$ = 0.28 (40% EtOAc in pentane)

MP: 118 °C (recrystallized from Et_2O)

IR (thin film, ν_{max} / cm^{-1}): 1679, 1565, 1318, 1306, 1115, 785

HRMS (ESI^+) calc. for $C_{18}H_{17}O_3S$ $[M+H]^+$ 313.0893, found 313.0892

^{1}H NMR (500 MHz, Chloroform-*d*): δ 7.91 (d, *J* = 8.5 Hz, 2H), 7.70 (dd, *J* = 8.5, 1.2 Hz, 2H), 7.58 (tt, *J* = 7.5, 1.2 Hz, 1H), 7.45 (d, *J* = 7.9 Hz, 2H) 7.41 (d, *J* = 8.5 Hz, 2H), 3.01 (s, 2H), 2.61 (s, 3H), 1.74 (s, 2H)

^{13}C NMR (126 MHz, Chloroform-*d*): δ 197.6, 140.7, 136.6, 136.1, 133.4, 129.2, 128.6, 127.5, 127.2, 36.3, 36.3, 30.6, 26.8

1-(4-Fluorophenyl)-3-(phenylsulfonyl)bicyclo[1.1.0]butane, 16

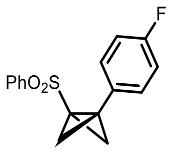

Prepared according to the general procedure using 1-fluoro-4-iodobenzene (29 µL, 0.25 mmol, 1.0 eq.) as coupling partner. The title compound was purified by flash chromatography (20→30% Et_2O in pentane), and isolated as a colourless solid (66 mg, 0.23 mmol, 91%).

R$_f$ = 0.37 (50% Et_2O in pentane)

MP: 114 °C (recrystallized from Et_2O)

IR (thin film, ν_{max} / cm^{-1}): 1523, 1305, 1229, 1147, 883, 836, 812, 729

HRMS (ESI^+) calc. for $C_{16}H_{13}O_2F^{23}Na^{32}S$ $[M+Na]^+$ 311.0512, found 311.0511

^{1}H NMR (400 MHz, Chloroform-*d*): δ 7.69 (d, *J* = 8.4, 1.3 Hz, 2H), 7.49 (tt, *J* = 7.5, Hz, 1H), 7.44 (t, *J* = 7.3 Hz, 2H), 7.24-7.18 (m, 2H), 7.01 (t, *J* = 8.7 Hz, 2H), 2.81 (t, *J* = 0.9 Hz, 2H), 1.59 (t, *J* = 0.9 Hz, 2H)

^{13}C NMR (101 MHz, Chloroform-*d*): δ 162.5 (d, *J* = 246.8 Hz), 140.7, 133.3, 129.1, 128.8 (d, *J* = 8.3 Hz), 127.5, 126.4 (d, *J* = 3.2 Hz), 115.7 (d, *J* = 21.9 Hz), 35.9, 34.2, 30.7

^{19}F NMR (376 MHz, Chloroform-*d*) δ -114.5

1-(4-Chlorophenyl)-3-(phenylsulfonyl)bicyclo[1.1.0]butane, 17

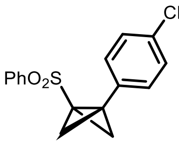

Prepared according to the general procedure using 1-chloro-4-iodobenzene (60 mg, 0.25 mmol, 1.0 eq.) as coupling partner. The title compound was purified by flash chromatography (20→40% Et_2O in pentane), and isolated as a colourless solid (63 mg, 0.21 mmol, 89%).

R_f = 0.32 (50% Et_2O in pentane)

MP: 91 °C (recrystallized from Et_2O)

IR (thin film, ν_{max} / cm^{-1}): 1317, 1147, 879, 883, 631

HRMS (ESI^+) calc. for $C_{16}H_{14}O_2{}^{35}ClS$ $[M+H]^+$ 305.0398, found 305.0399

^{1}H NMR (400 MHz, Chloroform-*d*): δ 7.71 (dd, *J* = 8.5, 1.2 Hz, 2H), 7.6 (tt, *J* = 7.5, 1.2 Hz, 1H), 7.47 (t, *J* = 7.8 Hz, 2H), 7.30 (t, *J* = 8.7 Hz, 2H) 7.27 (t, *J* = 8.7 Hz, 2H), 2.93 (s, 2H), 1.71 (s, 2H)

^{13}C NMR (101 MHz, Chloroform-*d*): δ 140.6, 133.7, 133.3, 129.3, 129.1, 128.8, 128.4, 127.5, 35.9, 34.9, 30.5

1-(Phenylsulfonyl)-3-(p-tolyl)bicyclo[1.1.0]butane, 18

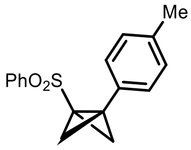

Prepared according to the general procedure using 1-iodo-4-methylbenzene (55 mg, 0.25 mmol, 1.0 eq.) as coupling partner. The title compound was purified by flash chromatography (20→30% Et_2O in pentane), and isolated as a colourless solid (65 mg, 0.23 mmol, 91%).

R R_f = 0.28 (40% Et_2O in pentane)

MP: 86 °C (recrystallized from Et_2O)

IR (thin film, ν_{max} / cm^{-1}): 3064, 2921, 1446, 1305, 1111, 882, 818, 728, 689, 627

HRMS (ESI^+) calc. for $C_{17}H_{17}O_2{}^{32}S$ $[M+H]^+$ 285.0944, found 285.0946

^{1}H NMR (400 MHz, Chloroform-*d*): δ 7.66 (dd, *J* = 8.5, 1.2 Hz, 2H), 7.54 (tt, *J* = 7.5, 1.2 Hz, 1H), 7.40 (t, *J* = 7.8 Hz, 2H), 7.19 (d, *J* = 8.3 Hz, 2H), 7.11 (d, *J* = 8.0 Hz, 2H), 2.90 (s, 2H), 2.35 (s, 3H), 1.64 (s, 2H)

^{13}C NMR (101 MHz, Chloroform-*d*): δ 142.0, 137.4, 133.1, 129.4, 129.0, 127.5, 127.3, 127.0, 35.7, 34.3, 31.3, 21.3

1-(4-Methoxyphenyl)-3-(phenylsulfonyl)bicyclo[1.1.0]butane, 19

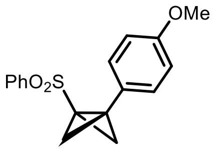

Prepared according to the general procedure using 1-iodo-4-methoxybenzene (59 mg, 0.25 mmol, 1.0 eq.) as coupling partner. The title compound was purified by flash chromatography (20→30% Et_2O in pentane, 1% NEt_3), and isolated as a colourless solid (53 mg, 0.17 mmol, 70%).

R_f = 0.21 (50% Et_2O in pentane)

MP: 100 °C (recrystallized from Et_2O)

IR (thin film, ν_{max} / cm^{-1}): 2962, 1304, 1248, 1146, 883, 758

HRMS (ESI^+) calc. for $C_{17}H_{16}O_3{}^{23}Na^{32}S$ $[M+Na]^+$ 323.0712, found 323.0713

^{1}H NMR (500 MHz, Benzene-*d*): δ 7.84 (d, *J* = 6.9 Hz, 2H), 7.30 (d, *J* = 8.8 Hz, 2H), 7.00 (m, 1H), 6.97 (m, 2H), 6.81 (d, *J* = 8.8 Hz, 2H) 3.38 (s, 3H), 2.80 (s, 2H), 1.18 (s, 2H)

^{13}C NMR (126 MHz, Benzene-*d*) δ 159.4, 142.2, 132.1, 128.5, 128.5, 127.3, 122.4, 114.0, 54.4, 35.3, 33.5, 31.0

1-(3-Bromophenyl)-3-(phenylsulfonyl)bicyclo[1.1.0]butane, 20

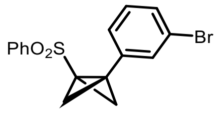

Prepared according to the general procedure using 1-bromo-3-iodobenzene (32 µL, 0.25 mmol, 1.0 eq.) as coupling partner. The title compound was purified by flash chromatography (20→40% Et_2O in pentane), and isolated as a colourless solid (78 mg, 0.22 mmol, 89%).

R_f = 0.30 (50% Et_2O in pentane)

MP: 148 °C (recrystallized from Et_2O)

IR (thin film, ν_{max} / cm^{-1}): 1318, 1148, 887, 782, 687, 625

HRMS (ESI^+) calc. for $C_{16}H_{14}O_2{}^{79}BrS$ $[M+H]^+$ 348.9892, found 348.9894

^{1}H NMR (500 MHz, Benzene-*d*): δ 7.59 (dd, *J* = 8.3, 1.3 Hz, 2H), 7.25 (t, *J* = 1.8 Hz, 1H), 7.11 (ddd, *J* = 8.0, 2.0, 1.0 Hz, 1H), 7.00 (ddd, *J* = 7.8, 1.7, 0.9 Hz, 1H), 6.93 (t, *J* = 7.5, 1.3 Hz, 1H), 6.83 (t, *J* = 8.0 Hz, 2H), 6.66 (t, *J* = 7.9 Hz, 1H), 2.48 (s, 2H), 0.93 (s, 2H)

^{13}C NMR (126 MHz, Benzene-*d*): δ 141.3, 133.8, 132.9, 130.5, 130.4, 130.1, 128.9, 127.6, 125.8, 122.8, 35.6, 35.4, 29.6

3-(3-(Phenylsulfonyl)bicyclo[1.1.0]butan-1-yl)phenyl trifluoromethanesulfonate, 21

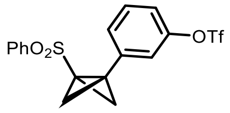

Prepared according to the general procedure using 3-iodophenyl trifluoromethanesulfonate **S8** (88 mg, 0.25 mmol, 1.0 eq.) as coupling partner. The title compound was purified by flash chromatography (30→50% Et_2O in pentane), and isolated as a yellow solid (84 mg, 0.20 mmol, 80%).

R_f = 0.24 (50% Et_2O in pentane)

MP: 78 °C (recrystallized from Et_2O)

IR (thin film, ν_{max} / cm^{-1}): 1422, 1209, 1139, 931, 871, 802,782, 652, 625

HRMS (ESI^+) calc. for $C_{17}H_{14}O_5F_3S_2$ $[M+H]^+$ 419.0229, found 419.0230

^{1}H NMR (500 MHz, Chloroform-*d*): δ 7.70 (dd, *J* = 8.5, 1.2 Hz, 2H), 7.58 (tt, *J* = 7.5, 1.2 Hz, 1H), 7.45 (t, *J* = 7.9 Hz, 2H), 7.40 (t, *J* = 7.8 Hz, 1H), 7.36 (dt, *J* = 7.9, 1.3 Hz, 1H) 7.22-7.16 (m, 2H), 2.93 (s, 2H), 1.73 (s, 2H)

^{13}C NMR (126 MHz, Chloroform-*d*): δ 149.6, 140.2, 134.2, 133.4, 130.3, 129.1, 127.4, 126.9, 120.2, 120.1, 118.7 (q, *J* = 320.9 Hz), 36.0, 35.7, 29.8

***Tert*-butyldimethyl(3-(3-(phenylsulfonyl)bicyclo[1.1.0]butan-1-yl)phenoxy)silane, 22**

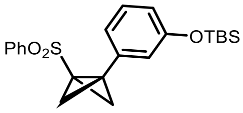

Prepared according to the general procedure using *tert*-butyl(3-iodophenoxy)dimethylsilane **S9** (84 mg, 0.25 mmol, 1.0 eq.) as coupling partner. The title compound was purified by flash chromatography (10→30% Et_2O in pentane), and isolated as a colourless solid (91 mg, 0.23 mmol, 91%).

$\boldsymbol{R_f}$ = 0.47 (50% Et_2O in pentane)

MP: 52 °C (recrystallized from Et_2O)

IR (thin film, ν_{max} / cm^{-1}): 2956, 2929, 2857, 1601, 1581, 1307, 1253, 1113, 959, 877, 783

HRMS (ESI^+) calc. for $C_{22}H_{29}O_3SSi$ $[M+H]^+$ 401.1601, found 401.1597

^{1}H NMR (500 MHz, Benzene-*d*): δ 7.72-7.68 (d, *J* = 7.1 2H), 7.09 (t, *J* = 2.1 Hz, 1H), 7.00 (t, *J* = 7.9 Hz, 1H), 6.96-6.84 (m, 4H), 6.78 (ddd, *J* = 8.1, 2.4, 1.0 Hz, 1H), 2.67 (s, 2H), 1.02 (s, 9H), 0.21 (s, 6H)

^{13}C NMR (126 MHz, Benzene-*d*): δ 156.2, 142.2, 132.9, 132.6, 129.7, 128.9, 127.7, 121.1, 119.7, 119.4, 35.7, 34.8, 30.8, 25.9, 18.4, -4.2

1-(Phenylsulfonyl)-3-(2-(trifluoromethyl)phenyl)bicyclo[1.1.0]butane, 23

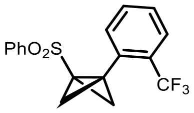

Prepared according to the general procedure using 1-iodo-2-(trifluoromethyl)benzene (35 µL, 0.25 mmol, 1.0 eq.) as coupling partner. The title compound was purified by flash chromatography (10→30% Et_2O in pentane), and isolated as a colourless solid (43 mg, 0.13 mmol, 51%).

$\boldsymbol{R_f}$ = 0.47 (50% Et_2O in pentane)

MP: 78 °C (recrystallized from Et_2O)

IR (thin film, ν_{max} / cm^{-1}): 1315, 1149, 1127, 1102, 1048, 887, 770, 755, 647

HRMS (ESI^+) calc. for $C_{17}H_{13}O_2F_3NaS$ $[M+Na]^+$ 361.0481, found 361.0480

^{1}H NMR (500 MHz, Chloroform-*d*): δ 8.01 (d, *J* = 7.9 Hz, 1H), 7.94 (dd, *J* = 7.1, 1.5 Hz, 2H), 7.71 (d, *J* = 8.1 Hz, 1H), 7.64 (tt, *J* = 7.5, 1.3 Hz, 1H), 7.62-7.53 (m, 3H), 7.44 (t, *J* = 1.1 Hz, 1H), 2.64 (s, 2H), 1.64 (s, 2H)

^{13}C NMR (101 MHz, Chloroform-*d*): δ 141.9, 133.5, 132.3, 131.7 (q, *J* = 30.5 Hz), 130.5, 130.3, 129.4, 128.2, 127.4, 126.5 (q, *J* = 5.5 Hz), 124.4 (q, *J* = 273.7 Hz), 34.0, 33.8, 30.9.

^{19}F NMR (471 MHz, Chloroform-*d*): δ -58.78

1-(Phenylsulfonyl)-3-(o-tolyl)bicyclo[1.1.0]butane, 24

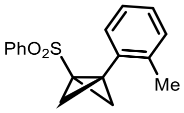

Prepared according to the general procedure using 1-iodo-2-methylbenzene (32 µL, 0.25 mmol, 1.0 eq.) as coupling partner. The title compound was purified by flash chromatography (20→30% Et_2O in pentane), and isolated as a colourless solid (57 mg, 0.20 mmol, 80%).

***R**$_f$* = 0.35 (50% Et_2O in pentane)

MP: 102 °C (recrystallized from Et_2O)

IR (thin film, $ν_{max}$ / cm^{-1}): 1317, 1147, 888, 798, 758, 689, 629

HRMS (ESI^+) calc. for $C_{17}H_{17}O_2S$ $[M+H]^+$ 285.0944, found 285.0944

^{1}H NMR (400 MHz, Chloroform-*d*): δ 7.89 (dd, *J* = 7.8, 1.4 Hz, 2H), 7.67-7.63 (m, 1H), 7.61 (tt, *J* = 7.4, 1.2 Hz, 1H), 7.52 (t, *J* = 7.5 Hz, 2H), 7.24-7.15 (m, 3H), 2.64 (s, 2H), 2.40 (s, 3H), 1.63 (s, 2H)

^{13}C NMR (101 MHz, Chloroform-*d*): δ 141.9, 139.1, 133.2, 130.7, 129.5, 129.2, 128.6, 128.0, 127.4, 126.4, 39.1, 33.6, 31.4, 20.7

2-(3-(Phenylsulfonyl)bicyclo[1.1.0]butan-1-yl)benzonitrile, 25

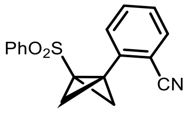

Prepared according to the general procedure using 2-iodobenzonitrile (57 mg, 0.25 mmol, 1.0 eq.) as coupling partner. The title compound was purified by flash chromatography (20→30% Et_2O in pentane), and isolated as a colourless solid (71 mg, 0.24 mmol, 96%).

***R**$_f$* = 0.32 (50% Et_2O in pentane)

MP: 138 °C (recrystallized from Et_2O)

IR (thin film, ν_{max} / cm^{-1}): 2224, 1319, 1148, 883, 759, 725, 689, 626

HRMS (ESI^+) calc. for $C_{17}H_{14}O_2NaS$ $[M+H]^+$ 296.0740, found 296.0738

^{1}H NMR (500 MHz, Chloroform-*d*): δ 7.83 (d, *J* = 7.2 Hz, 2H), 7.77 (d, *J* = 8.1 Hz, 1H), 7.67-7.62 (m, 2H), 7.60 (td, *J* = 7.8, 1.4 Hz, 1H), 7.53 (t, *J* = 7.8 Hz, 2H), 7.39 (t, *J* = 7.7 Hz, 1H), 3.06 (s, 2H), 1.86 (s, 2H)

^{13}C NMR (126 MHz, Chloroform-*d*): δ 141.1, 135.5, 133.9, 133.7, 132.8, 129.4, 128.5, 128.1, 127.5, 118.3, 113.7, 39.1, 36.3, 29.1

1-(4-Bromo-2-fluorophenyl)-3-(phenylsulfonyl)bicyclo[1.1.0]butane, 26

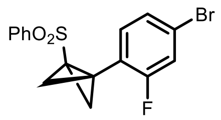

Prepared according to the general procedure using 4-bromo-2-fluoro-1-iodobenzene (75 mg, 0.25 mmol, 1.0 eq.) as a coupling partner. The title compound was purified by flash chromatography (20→30% Et_2O in pentane), and isolated as colourless solid (75 mg, 0.20 mmol, 82%).

R_f = 0.37 (50% Et_2O in pentane)

MP: 128 °C (recrystallized from Et_2O)

IR (thin film, ν_{max} / cm^{-1}): 1319, 1149, 855, 732, 631

HRMS (ESI^+) calc. for $C_{16}H_{13}O_2BrFS$ $[M+H]^+$ 366.9798, 368.9777, found 366.9801, 368.9779

^{1}H NMR (500 MHz, Benzene-*d*): δ 7.74-7.70 (d, *J* = 7.1 Hz 2H), 6.96-6.89 (m, 2H), 6.88-6.84 (m, 4H), 2.72 (s, 2H), 1.04 (s, 2H)

^{13}C NMR (126 MHz, Benzene-*d*): δ 161.5 (d, *J* = 253.2 Hz), 141.8, 132.5, 130.7, 128.7, 127.3 (d, *J* = 3.6 Hz), 127.2, 121.2 (d, *J* = 9.5 Hz), 119.5 (d, *J* = 25.4 Hz), 118.1 (d, *J* = 12.0 Hz), 37.3, 34.0, 25.5

^{19}F NMR (470 MHz, Benzene-*d*): δ -111.9

1-(3-(Phenylsulfonyl)bicyclo[1.1.0]butan-1-yl)naphthalene, 27

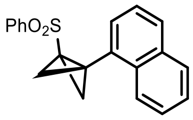

Prepared according to the general procedure using 1-iodonaphthalene (37 µl, 0.25 mmol, 1.0 eq.) as coupling partner. The title compound was purified by flash chromatography (20→40% Et_2O in pentane), and isolated as a colourless solid (63 mg, 0.20 mmol, 82%).

R_f = 0.31 (50% Et_2O in pentane)

MP: 121 °C (recrystallized from Et_2O)

IR (thin film, ν_{max} / cm^{-1}): 3062, 1317, 1149, 801, 729, 625

HRMS (ESI^+) calc. for $C_{20}H_{17}O_2S$ $[M+Na]^+$ 321.0944, found 321.0942

^{1}H NMR (500 MHz, Chloroform-*d*): δ 8.20-8.18 (m, 1H), 8.01 (dd, *J* = 7.2, 1.2 Hz, 1H), 7.96 (d, *J* = 7.2 Hz, 2H), 7.91-7.87 (m, 1H), 7.85 (d, *J* = 8.3 Hz, 1H), 7.62 (tt, *J* = 7.5, 1.2 Hz, 1H), 7.57-7.48 (m, 5H), 2.75 (s, 2H), 1.84 (s, 2H)

^{13}C NMR (126 MHz, Chloroform-*d*): δ 142.2, 134.5, 133.9, 133.3, 129.3, 128.9, 128.6, 127.7, 127.4, 126.6, 126.3, 125.9, 125.8, 124.7, 40.0, 33.3, 31.2

Ethyl (*Z*)-3-(3-(phenylsulfonyl)bicyclo[1.1.0]butan-1-yl)acrylate, 28

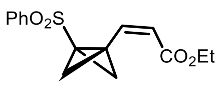

Prepared according to the general procedure using ethyl (*Z*)-3-iodoacrylate (32 µL, 0.25 mmol, 1.0 eq.) as coupling partner. The title compound was purified by flash chromatography (20→40% Et_2O in pentane), and isolated as a green-yellow oil (40 mg, 0.13 mmol, 54%).

R_f = 0.29 (50% Et_2O in pentane)

IR (thin film, ν_{max} / cm^{-1}): 1719, 1638, 1318, 1217, 1149, 1147, 909, 735, 690, 615

HRMS (ESI^+) calc. for $C_{15}H_{17}O_4S$ $[M+H]^+$ 293.0842, found 293.0841

^{1}H NMR (500 MHz, Chloroform-*d*): δ 7.94 (d, *J* = 7.1 Hz, 2H), 7.66 (tt, *J* = 7.5, 1.2 Hz 1H), 7.59 (t, *J* = 7.6 Hz, 2H), 6.57 (d, *J* = 11.4 Hz, 1H), 6.15 (d, *J* = 11.4 Hz, 1H), 4.21 (q, *J* = 7.1 Hz, 2H), 2.84 (s, 2H), 1.89 (s, 2H), 1.32 (t, *J* = 7.1 Hz, 3H)

^{13}C NMR (126 MHz, Chloroform-*d*): δ 165.4, 141.6, 137.8, 133.6, 129.2, 127.3, 125.1, 60.3, 41.8, 35.2, 28.4, 14.2

(*E*)-1-(Oct-1-en-1-yl)-3-(phenylsulfonyl)bicyclo[1.1.0]butane, (*E*)-29

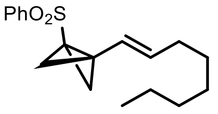

Prepared according to the general procedure using (*E*)-1-iodooct-1-ene (58 mg, 0.25 mmol, 1.0 eq.) as coupling partner. The title compound was purified by flash chromatography (10→30% Et_2O in pentane), and isolated as a yellow oil (46 mg, 0.15 mmol, 61%).

$\boldsymbol{R_f}$ = 0.5 (40 % Et_2O in pentane)

IR (thin film, ν_{max} / cm^{-1}): 2926, 1317, 1146, 623

HRMS (ESI^+) calc. for $C_{18}H_{24}O_2{}^{23}NaS$ $[M+Na]^+$ 327.1388, found F327.1390

^{1}H NMR (400 MHz, Benzene-*d*): δ 7.96-7.86 (m, 2H), 7.00-6.88 (m, 3H), 5.92 (d, *J* = 15.1 Hz, 1H), 5.78 (dt, *J* = 15.0, 6.9 Hz, 1H), 2.54 (s, 2H), 1.99 (q, *J* = 7.2 Hz, 2H), 1.40-1.16 (m, 8H), 1.00 (s, 2H), 0.91 (t, *J* = 7.1 Hz, 3H)

^{13}C NMR (126 MHz, Benzene-*d*): δ 143.3, 136.2, 132.1, 128.8, 127.2, 120.6, 37.2, 32.6, 31.7, 31.1, 29.5, 29.1, 28.8, 22.7, 14.0

(*Z*)-1-(Oct-1-en-1-yl)-3-(phenylsulfonyl)bicyclo[1.1.0]butane, (*Z*)-29

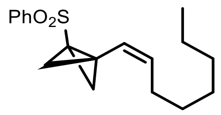

Prepared according to the general procedure using (*Z*)-1-iodooct-1-ene **S10** (58 mg, 0.25 mmol, 1.0 eq.) as coupling partner. The title compound was purified by flash chromatography (10→30% Et_2O in pentane), and isolated as a yellow oil (56 mg, 0.18 mmol, 74%).

$\boldsymbol{R_f}$ = 0.5 (40 % Et_2O in pentane)

IR (thin film, ν_{max} / cm^{-1}): 2926, 1316, 1146, 622

HRMS (ESI^+) calc. for $C_{18}H_{24}O_2{}^{23}NaS$ $[M+Na]^+$ 327.1389, found 327.1390

^{1}H NMR (500 MHz, Benzene-*d*): δ 7.94-7.88 (m, 2H), 6.97-6.93 (m, 3H), 5.98 (d, *J* = 10.9 Hz, 1H), 5.67 (dt, *J* = 10.8, 7.5 Hz, 1H), 2.54 (s, 2H), 2.17 (qd, *J* = 7.5, 1.6 Hz, 2H), 1.35-1.16 (m, 8H), 1.13 (s, 2H), 0.89 (t, *J* = 7.1 Hz, 3H)

^{13}C NMR (126 MHz, Benzene-*d*): δ 143.6, 137.5, 132.6, 128.7, 127.4, 119.9, 39.8, 32.1, 30.1, 29.4, 29.2, 28.4, 27.9, 23.0, 14.3

1,3-Dimethyl-5-(3-(phenylsulfonyl)cyclobut-1-en-1-yl)pyrimidine-2,4(1*H*,3*H*)-dione, 30

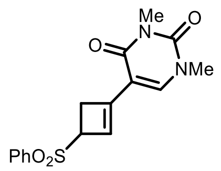

Prepared according to the general procedure using 5-iodo-1,3-dimethylpyrimidine-2,4(1*H*,3*H*)-dione (67 mg, 0.25 mmol, 1.0 eq.) as coupling partner. The title compound was purified by flash chromatography (80→100% EtOAc in pentane), and isolated as a colourless oil (43 mg, 0.13 mmol, 53%).

***R*$_f$** = 0.28 (EtOAc)

IR (thin film, ν_{max} / cm^{-1}): 1706, 1664, 1598, 1446, 1303, 1146, 726

HRMS (ESI^+) calc. for $C_{16}H_{17}O_4SN_2$ $[M+H]^+$ 333.0904, found 333.0903

^{1}H NMR (500 MHz, Chloroform-*d*): δ 7.92 (d, *J* = 7.9 Hz, 2H), 7.66 (tt, *J* = 7.4, 1.2 Hz, 1H), 7.56 (t, *J* = 7.8 Hz, 2H), 7.12 (s, 1H), 6.24 (d, *J* = 1.1 Hz, 1H), 4.33 (dt, *J* = 4.6, 1.4 Hz, 1H), 3.45 (s, 3H), 3.35 (s, 3H), 3.07 (dd, *J* = 13.2, 1.7 Hz, 1H), 2.93 (dd, *J* = 13.2, 4.5 Hz, 1H)

^{13}C NMR (126 MHz, Chloroform-*d*): δ 161.2, 151.1, 142.7, 140.1, 138.1, 133.9, 129.4, 128.7, 124.4, 108.0, 61.7, 37.6, 30.0, 28.0

4-((-3-(Phenylsulfonyl)bicyclo[1.1.0]butan-1-yl)ethynyl)benzonitrile, 31

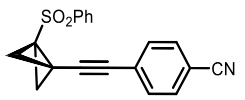

Prepared according to the general procedure using 4-(iodoethynyl)benzonitrile **S11** (80 mg, 0.25 mmol, 1.0 eq.) as coupling partner. The title compound was purified by flash chromatography (30→50% Et_2O in pentane), and isolated as a yellow solid (14 mg, 0.04 mmol, 17%).

***R*$_f$** = 0.25 (50% Et_2O in pentane)

MP: 148 °C (recrystallized from Et_2O)

IR (thin film, ν_{max} / cm^{-1}): 2852, 2226, 1603, 1500, 1318, 1146, 1106, 837, 754, 629

HRMS (ESI^+) calc. for $C_{19}H_{13}O_2NNaS$ $[M+Na]^+$ 342.0559, found 342.0557

^{1}H NMR (500 MHz, Chloroform-*d*): δ 7.96 (d, *J* = 7.1 Hz, 2H), 7.67-7.62 (m, 1H), 7.61 (d, *J* = 8.4 Hz, 2H), 7.56 (d, *J* = 8.1 Hz, 2H), 7.53 (d, *J* = 8.4 Hz, 2H), 2.75 (s, 2H), 1.75 (s, 2H)

^{13}C NMR (126 MHz, Chloroform-*d*): δ 140.3, 133.8, 132.6, 132.1, 129.3, 127.9, 127.6, 118.6, 111.9, 86.2, 83.1, 41.3, 33.7, 16.0

2-(3-(Phenylsulfonyl)bicyclo[1.1.0]butan-1-yl)pyridine, 32

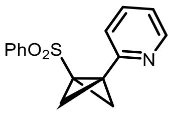

Prepared according to the general procedure using 2-iodopyridine (27 µL, 0.25 mmol, 1.0 eq.) as coupling partner. The title compound was purified by flash chromatography (40→70% EtOAc in pentane), and isolated as a yellow solid (67 mg, 0.25 mmol, 99%).

R_f = 0.13 (50% EtOAc in pentane)

MP: 118 °C (recrystallized from Et_2O/EtOAc)

IR (thin film, ν_{max} / cm^{-1}): 1588, 1318, 1149, 878, 784, 729, 628

HRMS (ESI^+) calc. for $C_{15}H_{14}O_2NS$ $[M+H]^+$ 272.0740, found 272.0740

^{1}H NMR (400 MHz, Chloroform-*d*): δ 8.50 (ddd, *J* = 4.9, 1.8, 0.9 Hz, 1H), 7.69-7.63 (m, 3H), 7.58 (tt, *J* = 7.4, 1.2 Hz, 1H), 7.46-7.41 (t, *J* = 7.9 Hz, 2H), 7.40 (d, *J* = 7.9 Hz, 1H), 7.20 (ddd, *J* = 7.5, 4.8, 1.1 Hz, 1H), 3.21 (s, 2H), 1.80 (s, 2H)

^{13}C NMR (101 MHz, Chloroform-*d*): δ 151.4, 149.7, 140.4, 136.4, 133.3, 129.1, 127.6, 122.1, 121.5, 36.5, 36.3, 31.8

3-(3-(Phenylsulfonyl)bicyclo[1.1.0]butan-1-yl)pyridine, 33

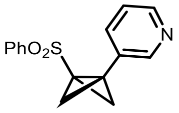

Prepared according to the general procedure using 3-iodopyridine (51 mg, 0.25 mmol, 1.0 eq.) as coupling partner. The title compound was purified by flash chromatography (20→40% Et_2OAc in pentane), and isolated as a colourless solid (63 mg, 0.23 mmol, 93%).

R_f = 0.08 (50% Et_2O in pentane)

MP: 121 °C (recrystallized from Et_2O/EtOAc)

IR (thin film, ν_{max} / cm^{-1}): 1317, 1147, 879, 730, 630

HRMS (ESI^+) calc. for $C_{15}H_{14}O_2NS$ $[M+H]^+$ 272.0740, Found 272.0742

^{1}H NMR (400 MHz, Chloroform-*d*): δ 8.54 (dd, *J* = 4.8, 1.6 Hz, 1H), 8.51 (dd, *J* = 2.4, 0.8 Hz, 1H), 7.72-7.67 (m, 3H), 7.59 (tt, *J* = 7.5, 1.2, 1H), 7.46 (t, *J* = 7.4, 2H), 7.26 (dd, J = 8.1, 4.8 Hz, 1H), 2.97 (s, 2H), 1.73 (s, 2H)

^{13}**C NMR** (101 MHz, Chloroform-*d*): δ 148.8, 148.6, 140.5, 134.2, 133.6, 129.2, 127.5, 127.1, 123.3, 36.0, 34.6, 28.4

4-(3-(Phenylsulfonyl)bicyclo[1.1.0]butan-1-yl)pyridine, 34

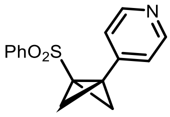

Prepared according to the general procedure using 4-iodopyridine (51 mg, 0.25 mmol, 1.0 eq.) as coupling partner. The title compound was purified by trituration with Et_2O, and isolated as a light-yellow solid (64 mg, 0.24 mmol, 95%).

MP: 160 °C (recrystallized from DCM/Et_2O)

IR (thin film, ν_{max} / cm^{-1}): 1599, 1307, 1148, 879, 879, 728, 624

HRMS (ESI^+) calc. for $C_{15}H_{14}O_2NS$ $[M+H]^+$ 272.0740, found 272.0739

^{1}**H NMR** (400 MHz, Chloroform-*d*): δ 8.52 (d, *J* = 4.6, 2H), 7.68 (d, *J* = 8.4 Hz, 2H), 7.59 (tt, *J* = 7.5, 1.2, 1H), 7.46 (t, *J* = 7.9 Hz, 2H), 7.21-7.16 (d, *J* = 4.6, 2H), 2.98 (s, 2H), 1.74 (s, 2H)

^{13}**C NMR** (101 MHz, Chloroform-*d*): δ 149.8, 140.7, 140.1, 133.6, 129.3, 127.5, 121.9, 37.0, 36.0, 28.9

7-Chloro-4-(3-(phenylsulfonyl)bicyclo[1.1.0]butan-1-yl)quinolone, 35

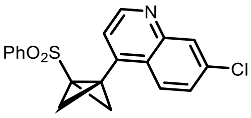

Prepared according to the general procedure using 7-chloro-4-iodoquinoline (72 mg, 0.25 mmol, 1.0 eq.) as coupling partner. The title compound was purified by flash chromatography (20→40% EtOAc in pentane), and isolated as an off-white solid (70 mg, 0.21 mmol, 83%).

R_f = 0.14 (50% Et_2O in pentane)

MP: 168 °C (recrystallized from Et_2O/EtOAc)

IR (thin film, ν_{max} / cm^{-1}): 1582, 1319, 1143, 1080, 865, 730, 615

HRMS (ESI^+) calc. for $C_{19}H_{15}O_2NClS$ $[M+H]^+$ 356.0507 and 358.0476, found 356.0510 and 358.0480

^{1}H NMR (400 MHz, Chloroform-*d*): δ 8.91 (d, *J* = 4.6 Hz, 1H), 8.15 (d, *J* = 2.2 Hz, 1H), 8.13 (d, *J* = 9.0 Hz, 1H), 7.92-7.88 (m, 2H), 7.81 (d, *J* = 4.6 Hz, 1H), 7.63 (tt, *J* = 7.5, 1.3 Hz, 1H), 7.54 (t, *J* = 7.9 Hz, 2H), 7.52 (dd, *J* = 9.10, 2.2 Hz, 1H) 2.90 (s, 2H), 1.93 (s, 2H)

^{13}C NMR (101 MHz, Chloroform-*d*): δ 151.2, 149.2, 141.7, 138.3, 135.5, 133.7, 129.5, 129.4, 127.9, 127.7, 127.5, 125.9, 120.6, 40.1, 34.9, 28.0

1-(3-(Phenylsulfonyl)bicyclo[1.1.0]butan-1-yl)isoquinoline, 36

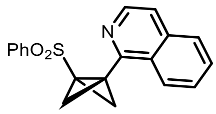

Prepared according to the general procedure using 1-iodoisoquinoline (64 mg, 0.25 mmol, 1.0 eq.) as coupling partner. The title compound was purified by flash chromatography (30→50% EtOAc in pentane), and isolated as a off-white solid (31 mg, 0.09 mmol, 38%).

R_f = 0.15 (50% Et_2O in pentane)

MP: 169 °C (recrystallized from Et_2O)

IR (thin film, $ν_{max}$ / cm^{-1}): 1320, 1153, 893, 818, 727. 686, 625

HRMS (ESI^+) calc. for $C_{19}H_{16}O_2SN$ $[M+H]^+$ 322.0896, found 322.0894

^{1}H NMR (500 MHz, Chloroform-*d*): δ 8.48 (d, *J* = 5.6 Hz, 1H), 8.29 (d, *J* = 8.6 Hz, 1H), 7.82 (d, *J* = 8.3 Hz, 1H), 7.74-7.70 (dd, *J* = 8.5, 1.3, 2H), 7.66 (ddd, *J* = 8.1, 6.8, 1.2 Hz, 1H), 7.58-7.53 (m, 2H), 7.48 (tt, *J* = 7.4, 1.2, Hz, 1H), 7.37 (t, *J* = 8.1, 2H), 3.33 (s, 2H), 1.98 (s, 2H)

^{13}C NMR (126 MHz, Chloroform-*d*): δ 151.4, 142.2, 140.8, 136.8, 133.2, 130.1, 128.9, 128.7, 127.8, 127.6, 127.3, 125.5, 120.2, 39.3, 36.3, 30.3

***Tert*-butyl 5-(3-(phenylsulfonyl)bicyclo[1.1.0]butan-1-yl)-1*H*-indole-1-carboxylate, 37**

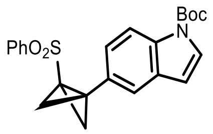

Prepared according to the general procedure using *tert*-butyl 5-iodo-1*H*-indole-1-carboxylate **S12** (86 mg, 0.25 mmol, 1.0 eq.) as coupling partner. The title compound was purified by flash chromatography (30→50% Et_2O in pentane, 1% NEt_3), and isolated as a colourless solid (92 mg, 0.23 mmol, 91%).

R_f = 0.34 (50% Et_2O in pentane)

MP: 110 °C (recrystallized from Et_2O)

IR (thin film, ν_{max} / cm^{-1}): 3062, 2979, 1731, 1396, 1138, 864, 766, 727

HRMS (ESI^+) calc. for $C_{23}H_{24}O_4NS$ $[M+H]^+$ 410.1421, found 410.1423

^{1}H NMR (500 MHz, Benzene-*d*): δ 8.34 (br, 1H), 7.70 (d, *J* = 6.9 Hz, 2H), 7.53 (br, 1H), 7.47 (d, *J* = 1.9 Hz, 1H), 7.31 (d, *J* = 7.9 Hz, 1H), 6.88 (t, *J* = 7.4 Hz, 1H), 6.81 (t, *J* = 7.5 Hz, 2H), 6.24 (d, *J* = 3.6 Hz, 1H), 2.80 (s, 2H), 1.37 (s, 9H), 1.14 (s, 2H)

^{13}C NMR (126 MHz, Benzene-*d*): δ 149.7, 142.2, 135.3, 132.5, 131.3, 128.3, 127.9, 126.6, 125.7, 123.9, 120.2, 115.7, 107.5, 83.2, 35.6, 34.7, 31.7, 27.9

2,6-Bis(3-(phenylsulfonyl)bicyclo[1.1.0]butan-1-yl)pyridine, 38

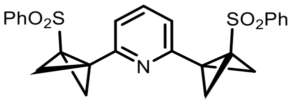

Prepared according to the general procedure using 2,6-diiodopyridine (40 mg, 0.12 mmol, 0.48 eq.) as coupling partner. The title compound was purified by flash chromatography (30→50% Et_2O in pentane), and isolated as a light-brown solid (33 mg, 0.07 mmol, 59%).

R$_f$ = 0.60 (80% EtOAc in pentane)

MP: 167 °C (recrystallized from Et_2O)

IR (thin film, ν_{max} / cm^{-1}): 2959, 1589, 1446, 1262, 1108, 1067, 876, 750, 659, 624

HRMS (ESI^+) calc. for $C_{25}H_{22}O_4N_1S_2$ $[M+H]^+$ 464.0985, found 464.0980

^{1}H NMR (500 MHz, Chloroform-*d*): δ 7.64 (dd, *J* = 7.4, 1.2 Hz, 4H), 7.51 (tt, *J* = 7.5, 1.2 Hz, 2H), 7.42 (t, *J* = 7.9 Hz, 1H), 7.39 (t, *J* = 7.9 Hz, 4H), 7.09 (d, *J* = 7.8 Hz, 2H), 3.11 (s, 4H), 1.73 (s, 4H)

^{13}C NMR (126 MHz, Chloroform-*d*): δ 151.2, 140.2, 136.5, 133.3, 129.1, 127.7, 119.7, 37.0, 36.3, 32.0

***N*-Diisopropyl-3-phenylbicyclo[1.1.0]butane-1-carboxamide, 39**

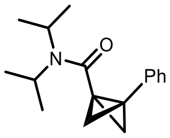

Prepared according to the general procedure using iodobenzene (28 µL, 0.25 mmol, 1.0 eq.) as coupling partner. The title compound was purified by flash chromatography (20→40% Et_2O in pentane, 1% NEt_3), and isolated as a colourless solid (47 mg, 0.18 mmol, 73%).

R_f = 0.54 (20% EtOAc in pentane)

MP: 56 °C (recrystallized from Et_2O)

IR (thin film, ν_{max} / cm^{-1}): 2965, 2930, 1622, 1484, 1346

HRMS (ESI^+) calc. for $C_{17}H_{24}ON$ $[M+H]^+$ 258.1852, found 258.1851

^{1}H NMR (500 MHz, Chloroform-*d*): δ 7.32-7.27 (m, 4H), 7.17 (tt, *J* = 8.6, 1.8 Hz, 1H), 4.70 (br, 1H), 3.23 (br, 1H), 2.81 (s, 2H), 1.52 (s, 2H), 1.19 (br, H6), 1.15 (br, H6)

^{13}C NMR (126 MHz, Chloroform-*d*): δ 167.6, 134.4, 128.1, 126.2, 126.2, 49.3, 45.9, 36.3, 30.3, 22.3, 21.5, 20.8

***N*,*N*-Diisopropyl-3-(4-(trifluoromethyl)phenyl)bicyclo[1.1.0]butane-1-carboxamide, 40**

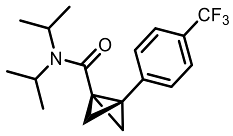

Prepared according to the general procedure using 1-iodo-4-(trifluoromethyl)benzene (54 mg, 0.25 mmol, 1.0 eq.) as coupling partner. The title compound was purified by flash chromatography (20→40% Et_2O in pentane, 1% NEt_3), and isolated as a colourless solid (76 mg, 0.23 mmol, 93%).

Large scale: **8c** (1.00 g, 5.52 mmol, 1.20 eq.), PhLi (1.8 M in *n*-Bu_2O, 3.07 mL, 5.52 mmol, 1.20 eq.), $ZnCl_2$ (0.75 g, 5.52 mmol, 1.20 eq.), $Pd(dba)_2$ (132 mg, 0.26 mmol, 5 mol%), tfp (107 mg, 0.46 mmol, 10 mol%) and 1-iodo-4-(trifluoromethyl)benzene (0.68 mL, 4.60 mmol, 1.0 eq.) afforded **40** as a colourless solid (1.35 g, 4.15 mmol, 96%).

R_f = 0.31 (40% Et_2O in pentane)

MP: 57 °C (recrystallized from Et_2O)

IR (thin film, ν_{max} / cm^{-1}): 2963, 2934, 1630, 1483, 1341

HRMS (ESI^+) calc. for $C_{18}H_{23}ONF_3$ $[M+H]^+$ 326.1726, found 326.1724

^{1}H NMR (500 MHz, Chloroform-*d*): δ 7.52 (d, *J* = 8.2 Hz, 2H), 7.38 (d, *J* = 8.1 Hz, 2H), 4.69 (sep, *J* = 6.6 Hz, 1H), 3.26 (sep, *J* = 6.7 Hz, 1H), 2.82 (s, 2H), 1.59 (s, 2H), 1.21 (d, *J* = 6.7 Hz, 6H), 1.14 (d, *J* = 6.8 Hz, 6H)

^{13}C NMR (126 MHz, Chloroform-*d*): δ 166.9, 139.3, 128.2 (q, *J* = 32.3 Hz), 126.5, 125.1 (q, *J* = 3.8 Hz), 124.5 (q, *J* = 270.2 Hz), 49.6, 46.2, 36.7, 29.2, 23.9, 21.6, 20.8

^{19}**F NMR** (471 MHz, Chloroform-*d*): δ -62.4

1-Phenyl-3-((4-(trifluoromethyl)phenyl)sulfonyl)bicyclo[1.1.0]butane, 41

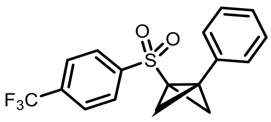

Prepared according to the general procedure using iodobenzene (28 µL, 0.25 mmol, 1.0 eq.) as coupling partner. The title compound was purified by flash chromatography (20→30% Et_2O in pentane, 1% NEt_3), and isolated as a colourless solid (64 mg, 0.19 mmol, 76%).

R_f = 0.44 (30% Et_2O in pentane)

MP: 104 °C (recrystallized from Et_2O)

IR (thin film, ν_{max} / cm^{-1}): 1449, 1304, 1133, 765

HRMS (ESI^+) calc. for $C_{17}H_{13}O_2F_3NaS$ $[M+Na]^+$ 361.0481, found 361.0479

^{1}H NMR (500 MHz, Benzene-*d*): δ 7.41 (d, *J* = 8.1 Hz, 2H), 7.05-7.01 (m, 2H), 7.01-6.95 (m, 5H), 2.58 (s, 2H), 1.05 (s, 2H)

^{13}C NMR (126 MHz, Benzene-*d*): δ 144.4, 134.0 (q, *J* = 32.8 Hz), 128.7, 128.4, 128.0, 127.7, 127.2, 125.9 (q, *J* = 3.6 Hz), 123.9 (d, *J* = 272.9 Hz), 35.4, 34.5, 31.6

^{19}F NMR (470 MHz, Benzene-*d*) δ -63.0

Methyl (2*S*)-2-((tert-butoxycarbonyl)amino)-3-(4-(3-(phenylsulfonyl)bicyclo[1.1.0]butan-1-yl)phenyl) propanoate, 42

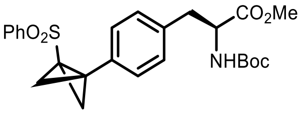

Prepared according to the general procedure using methyl (S)-2-((*tert*-butoxycarbonyl)amino)-3-(4-iodophenyl)propanoate **S13** (101 mg, 0.25 mmol, 1.0 eq.) as coupling partner. The title compound was purified by flash chromatography (30→40% EtOAc in pentane), and isolated as a light-orange solid (72 mg, 0.15 mmol, 61%).

R_f = 0.10 (50% Et_2O in pentane)

$[\alpha]_D^{25}$ −30 (c = 1.0, $CHCl_3$)

MP: 64 °C (recrystallized from Et_2O/EtOAc)

IR (thin film, ν_{max} / cm^{-1}): 3400, 2978, 1746, 1712, 1494, 1306, 1147, 882, 690

HRMS (ESI^+) calc. for $C_{25}H_{30}O_6NS$ $[M+H]^+$ 472.1788, found 472.1781

^{1}H NMR (500 MHz, Chloroform-*d*): δ 7.61 (d, *J* = 7.8 Hz, 2H), 7.56 (t, *J* = 7.4 Hz, 1H), 7.42 (t, *J* = 7.8 Hz, 2H), 7.20 (d, *J* = 7.8 Hz, 2H), 7.06 (d, *J* = 7.9 Hz, 2H), 4.99 (d, *J* = 8.2 Hz, 1H), 4.58 (q, *J* = 6.7 Hz, 1H), 3.72 (s, 3H), 3.08 (qd, *J* = 13.7, 6.1 Hz, 2H), 2.87 (s, 2H), 1.65 (s, 2H), 1.44 (s, 9H)

^{13}C NMR (126 MHz, Chloroform-*d*): δ 172.4, 155.2, 140.2, 135.5, 133.3, 129.3, 129.3, 129.1, 127.6, 127.3, 80.2, 54.6, 52.4, 38.2, 35.6, 34.9, 30.7, 28.5

(*S*)-2-((Tert-butoxycarbonyl)amino)-3-methoxy-3-oxopropyl 4-(3-(phenylsulfonyl)bicyclo[1.1.0]butan-1-yl)benzoate, 43

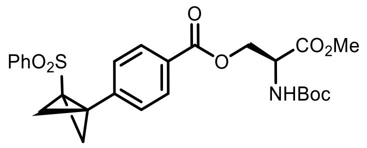

Prepared according to the general procedure using (*S*)-2-((*tert*-butoxycarbonyl)amino)-3-methoxy-3-oxopropyl 4-iodobenzoate **S14** (112 mg, 0.25 mmol, 1.0 eq.) as coupling partner. The title compound was purified by flash chromatography (30→40% EtOAc in pentane), and isolated as a colourless solid (31 mg, 0.06 mmol, 24%).

R$_f$ = 0.55 (80% EtOAc in pentane)

$[\alpha]_D^{25}$ +37 (c = 1.0, $CHCl_3$)

MP: 109 °C (recrystallized from Et_2O/EtOAc)

IR (thin film, ν_{max} / cm^{-1}): 3450, 2978, 1719, 1307, 1273, 1148, 879, 771, 729, 629

HRMS (ESI^+) calc. for $C_{23}H_{24}O_4NS$ $[M+2H-Boc]^+$ 416.1168, found 416.1157

^{1}H NMR (500 MHz, Chloroform-*d*): δ 7.94 (d, *J* = 8.4 Hz, 2H), 7.70 (d, *J* = 6.9 Hz, 2H), 7.58 (tt, *J* = 7.5, 1.2 Hz, 1H), 7.45 (t, *J* = 7.8, 2H), 7.39 (d, *J* = 8.4 Hz, 2H), 5.39 (d, *J* = 8.5 Hz, 1H), 4.72 (dt, *J* = 8.1, 3.7 Hz, 1H), 4.68–4.57 (m, 2H), 3.80 (s, 3H), 3.01 (s, 2H), 1.74 (s, 2H), 1.46 (s, 9H)

^{13}C NMR (126 MHz, Chloroform-*d*): δ 170.4, 165.7, 155.2, 140.5, 136.8, 133.3, 129.9, 129.1, 128.3, 127.3, 127.0, 80.5, 65.0, 53.0, 52.9, 36.2, 30.4, 30.3, 28.3

((3a*R*,5*R*,5a*S*,8a*S*,8b*R*)-2,2,7,7-Tetramethyltetrahydro-5*H*-bis([1,3]dioxolo)[4,5-b:4',5'-*d*]pyran-5-yl)methyl 4-(3-(phenylsulfonyl)bicyclo[1.1.0]butan-1-yl)benzoate, 44

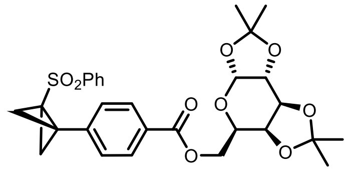

Prepared according to the general procedure using ((3a*R*,5*R*,5a*S*,8a*S*,8b*R*)-2,2,7,7-tetramethyltetrahydro-5*H*-bis([1,3]dioxolo)[4,5-b:4',5'-*d*]pyran-5-yl)methyl 4-iodobenzoate **S15** (123 mg, 0.25 mmol, 1.0 eq.) as coupling partner. The title compound was purified by flash chromatography (40→70% Et_2O in pentane), and isolated as a light-orange solid (120 mg, 0.22 mmol, 86%).

R$_f$ = 0.10 (50% Et_2O in pentane)

MP: 89 °C (recrystallized from Et_2O/EtOAc)

$[\alpha]_D^{25}$ –41 (c = 1.0, $CHCl_3$)

IR (thin film, vmax / cm^{-1}): 1715, 1253, 1211, 1148, 1067, 1004, 879, 772, 730, 629

HRMS (ESI^+) calc. for $C_{29}H_{33}O_9S$ $[M+H]^+$ 557.1840, found 557.1835

^{1}H NMR (400 MHz, Chloroform-*d*): δ 7.98 (d, *J* = 8.4 Hz, 2H), 7.63 (dd, *J* = 7.1, 1.2 Hz, 2H), 7.56 (tt, J = 7.5, 1.2 Hz, 1H), 7.41 (t, *J* = 7.9 Hz, 2H), 7.33 (d, *J* = 8.5 Hz, 2H), 5.58 (d, *J* = 5.0 Hz, 1H), 4.66 (dd, *J* = 7.9, 2.5 Hz, 1H), 4.54 (dd, *J* = 11.5, 4.9 Hz, 1H), 4.44 (dd, *J* = 11.4, 7.6 Hz, 1H), 4.37-4.32 (m, 2H), 4.19 (ddd, *J* = 7.1, 4.9, 1.9 Hz, 1H), 2.99-2.95 (m, 2H), 1.73 (s, 2H), 1.53 (s, 3H), 1.49 (s, 3H), 1.37 (s, 3H), 1.34 (s, 3H)

^{13}C NMR (126 MHz, Chloroform-*d*): δ 166.2, 140.2, 136.4, 133.4, 123.0, 129.1, 129.1, 127.5, 127.0, 109.9, 109.0, 96.5, 71.3, 70.9, 70.7, 66.3, 64.1, 36.3, 36.0, 30.5, 26.2, 26.1, 25.2, 24.7

(8*R*,9*S*,13*S*,14*S*)-13-Methyl-3-(3-(phenylsulfonyl)bicyclo[1.1.0]butan-1-yl)-6,7,8,9,11,12,13,14,15,16-decahydro-17*H*-cyclopenta[a]phenanthren-17-one, 45

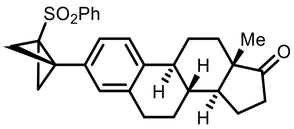

Prepared according to the general procedure using estrone triflate **S16** (101 mg, 0.25 mmol, 1.0 eq.) as coupling partner and $Pd(PPh_3)_4$ (29 mg, 0.03 mmol, 10 mol%) as catalyst. The title compound was purified by flash chromatography (30→60% Et_2O in pentane), and isolated as a colourless solid (32 mg, 0.07 mmol, 29%).

R$_f$ = 0.28 (50% Et_2O in pentane)

$[\alpha]_D^{25}$ + 105 (c = 1.0, $CHCl_3$)

MP: 147 °C (recrystallized from Et_2O/EtOAc)

IR (thin film, ν_{max} / cm^{-1}): 2934, 1736, 1317, 1147, 758

HRMS (ESI^+) calc. for $C_{28}H_{31}O_3S$ $[M+H]^+$ 447.1988, found 447.1991

^{1}H NMR (500 MHz, Chloroform-*d*): δ 7.69 (dd, *J* = 7.1, 1.3 Hz, 2H), 7.56 (tt, *J* = 7.5, 1.2 Hz, 1H), 7.42 (t, *J* = 7.5 Hz, 2H), 7.23 (d, *J* = 8.2 Hz, 1H), 7.12 (dd, *J* = 8.1, 2.1 Hz, 1H), 6.99 (d, *J* = 2.0 Hz, 1H), 2.95-2.89 (m, 2H), 2.89-2.83 (m, 2H), 2.56-2.47 (m, 1H), 2.46-2.39 (m, 1H), 2.35-2.26 (m, 1H), 2.21-1.94 (m, 4H), 1.70-1.39 (m, 8H), 0.93 (s, 3H)

^{13}C NMR (126 MHz, Chloroform-*d*): δ 221.0, 141.0, 139.3, 136.7, 133.0, 129.0, 127.7, 127.7, 127.5, 125.7, 124.5, 50.7, 48.1, 44.5, 38.3, 36.0, 35.8, 35.7, 34.3, 31.7, 31.4, 29.4, 26.6, 25.9, 21.8, 14.0

1.6.1 Failed coupling reactions

Coupling partners that failed to couple or resulted in decomposition.

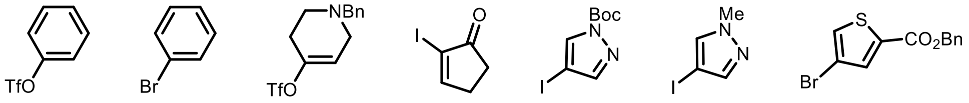

1.7 Diversification of BCBs

General BCB to cyclobutene procedure

To a solution of BCB (0.05-0.40 mmol, 1.0 eq.) in $CHCl_3$ (0.50-1.50 mL) was added 1 drop of 1M HCl in Et_2O at rt. The reaction was stirred for 30 min at rt before evaporating *in vacuo* to afford pure cyclobutene.

((3-Phenylcyclobut-2-en-1-yl)sulfonyl)benzene, 12

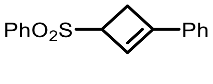

Prepared according to the general procedure using **11** (150 mg, 0.55 mmol, 1.0 eq.) to afford the title compound as a clear oil (150 mg, 0.55 mmol, quant.).

IR (thin film, ν_{max} / cm^{-1}): 1446, 1304, 1146, 1084, 765, 728, 692

HRMS (ESI$^+$) calc. for $C_{16}H_{14}O_2{}^{23}Na^{32}S$ [M+Na]$^+$ 293.0607, found 293.0608

^{1}H NMR (500 MHz, Chloroform-*d*): δ 7.98 (d, *J* = 7.2 Hz, 2H), 7.68 (tt, *J* = 7.5, 1.2, 1H), 7.60 (t, *J* = 8.4 Hz, 2H), 7.39-7.3 (m, 5H), 6.16 (d, *J* = 1.2 Hz, 1H), 4.36 (dt, *J* = 4.3, 1.6 Hz, 1H), 3.14 (dd, *J* = 13.6, 1.9 Hz, 1H), 3.08 (dd, *J* = 13.6, 4.3 Hz, 1H)

^{13}C NMR (126 MHz, Chloroform-*d*): δ 151.6, 138.2, 133.8, 132.6, 129.5, 129.3, 128.7, 128.6, 125.3, 120.2, 60.4, 31.0

1-Methoxy-4-(3-(phenylsulfonyl)cyclobut-1-en-1-yl)benzene, 46

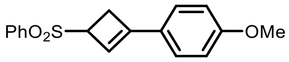

Prepared according to the general procedure using **19** (40 mg, 0.55 mmol, 1.0 eq.) to afford the title compound as a clear oil (40 mg, 0.13 mmol, quant.).

IR (thin film, ν_{max} / cm^{-1}): 2956, 1507, 1304, 1217, 1146, 727, 673

HRMS (ESI^+) calc. for $C_{17}H_{16}O_3NaS$ $[M+Na]^+$ 323.0712, found 323.0713

^{1}H NMR (400 MHz, Chloroform-*d*): δ 7.95 (dd, *J* = 7.1, 1.4 Hz, 2H), 7.65 (tt, *J* = 7.5, 1.3, 1H), 7.56 (t, *J* = 7.8, 2H), 7.27 (d, *J* = 8.8 Hz, 2H), 6.86 (d, *J* = 8.8 Hz, 2H), 5.90 (d, *J* = 1.1 Hz, 1H), 4.24 (dt, *J* = 4.1, 1.6 Hz, 1H), 3.74 (s, 3H), 3.00 (dd, *J* = 13.6, 2.0 Hz, 1H), 2.94 (dd, *J* = 13.6, 4.3 Hz, 1H)

^{13}C NMR (101 MHz, Chloroform-*d*): δ 160.7, 150.2, 138.3, 133.8, 129.2, 128.7, 126.9, 125.7, 117.45, 114.8, 60.7, 55.5, 31.0

***Tert*-butyldimethyl(3-(3-(phenylsulfonyl)cyclobut-1-en-1-yl)phenoxy)silane, 47**

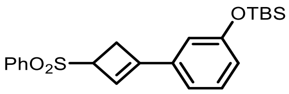

Prepared according to the general procedure using **22** (10 mg, 0.03 mmol, 1.0 eq.) to afford the title compound as a clear oil (10 mg, 0.03 mmol, quant.).

IR (thin film, ν_{max} / cm^{-1}): 2956, 2929, 2857, 1601, 1581, 1476, 1320, 1113, 959, 840, 783

HRMS (ESI^+) calc. for $C_{22}H_{29}O_3SSi$ $[M+H]^+$ 401.1601, found 401.1597

^{1}H NMR (500 MHz, Chloroform-*d*): δ 7.85 (d, *J* = 8.5 Hz, 2H), 7.55 (tt, *J* = 7.5, 1.2 Hz, 1H), 7.47 (t, *J* = 7.5 Hz, 1H), 7.09 (t, *J* = 7.8 Hz, 1H), 6.83 (dt, *J* = 7.7, 1.3 Hz, 1H), 6.70 (ddd, *J* = 8.0, 2.4, 1.0 Hz, 1H), 6.67 (t, *J* = 2.0 Hz, 2H), 6.00 (d, *J* = 1.2 Hz, 1H), 4.22 (dt, *J* = 4.3, 1.5 Hz, 1H), 2.98 (dd, *J* = 13.6, 1.9 Hz, 1H), 2.91 (dd, *J* = 13.6, 4.4 Hz, 1H), 0.88 (s, 9H), 0.09 (s, 6H)

^{13}C NMR (126 MHz, Chloroform-*d*): δ 156.0 151.5, 138.2, 134.1, 133.8, 129.7, 129.3, 128.7, 121.3, 120.3, 118.5, 116.8, 60.3, 31.0, 25.8, 18.4, -4.2

Ethyl (1*S,3*S**,5*R**)-1-phenyl-3-(phenylsulfonyl)bicyclo[2.1.0]pentane-5-carboxylate, 48**

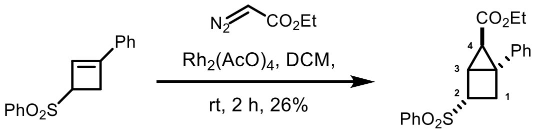

To a solution of **6** (54 mg, 0.20 mmol, 1.0 eq.) in DCM (1 mL) was added a solution of ethyl diazoacetate (≥13 wt% in dichloromethane, 42 µL, 0.40 mmol, 2.00 eq.) in DCM (1 mL) dropwise *via* syringe pump over 1 h at rt. The solvent was removed *in vacuo* to afford a yellow oil which was purified *via* flash chromatography (1st column: 20% Et_2O in pentane, 2nd column: 100% DCM) to afford **48** as a colourless waxy solid (19 mg, 0.05 mmol, 26%).

R R_f = 0.37 (50% Et_2O in pentane), 0.24 (100% DCM)

IR (thin film, ν_{max} / cm^{-1}): 1726, 1446, 1307, 1186, 1148, 730, 695

HRMS (ESI^+) calc. for $C_{20}H_{20}O_4NaS$ $[M+Na]^+$ 379.0975, found 379.0968

^{1}H NMR (400 MHz, Chloroform-*d*): δ 7.96 (dd, *J* = 8.5, 1.4 Hz, 2H), 7.66 (tt, *J* = 7.4, 1.3 Hz, 1H), 7.56 (t, *J* = 7.9 Hz, 2H), 7.38-7.29 (m, 4H), 7.28-7.22 (m, 1H), 4.20 (qd, *J* = 7.1, 2.3 Hz, 2H), 4.07 (dd, *J* = 5.2, 3.5 Hz, 1H, H2), 2.94 (ddd, *J* = 12.3, 3.5, 1.1 Hz, 1H, H1), 2.70 (dd, *J* = 6.0, 1.0 Hz, 1H, H3), 2.60 (ddd, *J* = 12.4, 5.3, 1.1 Hz, 1H, H1), 2.38 (dd, *J* = 6.0, 1.0 Hz, 1H, H4), 1.30 (t, *J* = 7.1 Hz, 3H)

^{13}C NMR (101 MHz, Chloroform-*d*): δ 170.3, 138.4, 138.0, 134.0, 129.5, 128.8, 128.6, 127.8, 127.6, 61.0, 55.4, 37.2, 31.2, 28.6, 28.1, 14.4

((3-Phenylcyclobutyl)sulfonyl)benzene, 49

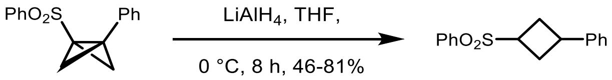

~1:3, trans:cis, MeOH quench; ~1:1, trans:cis, H_2O quench

To a solution of **11** (27 mg, 0.10 mmol, 1.0 eq.) in THF (0.5 mL) was added a solution of $LiAlH_4$ in Et_2O (4 M, 50.0 µL, 0.20 mmol, 2.00 eq.) at 0 °C. The reaction was stirred for 8 h at 0 °C and quenched with methanol. The solution was diluted with Et_2O (2 mL) mixture and passed through a silica plug. The organics were evaporated in vacuo and purified *via* flash chromatography (DCM) to yield an off-white solid (13-22 mg, 0.05-0.08 mmol, 46-81%, 2:3 *dr*, major anti/trans). Data identical to literature values.[7]

R_f = 0.22 (100% DCM)

^{1}H NMR (400 MHz, Chloroform-*d*): δ 7.97-7.93 (m, 0.25H, trans **2H**), 7.93-7.88 (m, 0.75H, cis **2H**), 7.70-7.63 (m, 2H, trans + cis **2H**), 7.61-7.53 (m, 2H, trans + cis **4H**), 7.36-7.28 (m, 2H, trans + cis **4H**), 7.28-7.17 (m, 3H, trans + cis **6H**), 3.90-3.72 (m, 1.25H, trans + cis **3H**), 3.41 (tt, *J* = 10.0, 8.2 Hz, 0.75H, cis **1H**), 3.03-2.94 (m, 0.5H, trans **2H**), 2.75 (qd, *J* = 9.8, 2.6 Hz, 1.5H, cis **2H**), 2.62-2.41 (m, 2H, trans + cis **4H**)

^{13}**C NMR** (101 MHz, Chloroform-*d*): δ 144.1 (trans), 143.2 (cis), 138.4 (cis), 138.2 (trans), 133.8 (cis & trans), 129.4 (cis & trans), 128.7 (cis & trans), 128.5 (trans), 128.4 (cis), 126.9 (cis), 126.8 (cis), 126.7 (trans), 126.3 (trans), 54.9 (trans), 53.2 (cis), 35.6 (trans), 34.3 (cis), 30.8 (cis), 29.8 (trans).

3-Chloro-1-methoxy-3-(phenylsulfonyl)cyclobutyl)benzene, 50

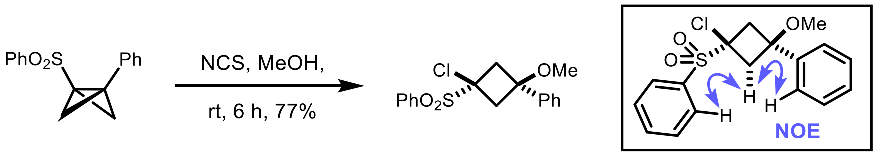

To a solution of **11** (27 mg, 0.10 mmol, 1.0 eq.) in MeOH (0.5 mL) at rt was added *N*-chlorosuccinimide (13 mg, 0.10 mmol, 1.0 eq.). The mixture was stirred at rt for 6 h before evaporating in vacuo. The resulting residue was purified by flash chromatography (0→20% Et_2O in pentane) to afford **50** as a colourless oil (26 mg, 0.08 mmol, 77%).

R$_f$ = 0.37 (10% Et2O in pentane)

IR (thin film, ν_{max} / cm^{-1}): 2948, 1496, 1398, 727

HRMS (ESI$^+$) calc. for $C_{17}H_{17}ClO_3{}^{23}Na^{32}S$ $[M+Na]^+$ 359.0484, found 359.0479.

^{1}**H NMR** (500 MHz, Chloroform-*d*): δ 8.05-7.86 (m, 2H), 7.75-7.64 (m, 1H), 7.64-7.51 (m, 2H), 7.48-7.33 (m, 5H), 3.76-3.65 (m, 2H), 3.02 (s, 3H), 2.91-2.83 (m, 2H)

^{13}**C NMR** (126 MHz, Chloroform-*d*): δ 140.3, 134.6, 134.2, 130.7, 129.1, 128.7, 128.3, 126.1, 76.8, 75.4, 51.2, 44.0

1-((2-Fluorophenyl)sulfonyl)-3-phenylbicyclo[1.1.0]butane, 52

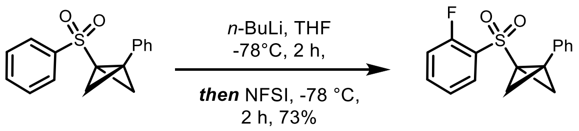

To a solution of **5** (32 mg, 0.12 mmol. 1.20 eq.) in THF (0.4 mL) was added *n*-BuLi in pentane (1.9 M, 64.0 µL, 0.12 mmol, 1.2 eq.) at –78 °C. The reaction was stirred for 2 h before addition of a solution of NFSI (16 mg, 0.12 mmol, 1.2 eq.) in THF (0.5 mL) at –78 °C, and stirred for 2 h before quenching by addition of 2 drops of water. The organic phase was diluted with DCM, dried with anhydrous $MgSO_4$, filtered and evaporated *in vacuo.* The residue was purified *via* flash chromatography (1st column: 20% Et_2O in pentane, 2nd column: 100% DCM) to yield **52** as an off-white solid (21 mg, 0.07 mmol, 73%).

R$_f$ = 0.28 (40% Et_2O in pentane)

MP: 110 °C (recrystallized from Et_2O)

IR (thin film, ν_{max} / cm^{-1}): 1474, 1322, 1264, 1146, 879, 826, 760, 690, 622

HRMS (ESI^+) calc. for $C_{16}H_{14}O_2FNaS$ $[M+H]^+$ 311.0512, found 311.0512

^{1}H NMR (500 MHz, Chloroform-*d*): δ 7.74-7.69 (m, 1H), 7.60-7.54 (m, 1H), 7.45-7.41 (m, 2H), 7.36-7.27 (m, 3H), 7.23-7.17 (m, 2H), 3.10 (s, 2H), 1.74 (s, 2H)

^{13}C NMR (126 MHz, Chloroform-*d*): δ 159.4 (d, *J* = 256.5 Hz), 135.3 (d, *J* = 8.4 Hz), 130.3, 129.6 (d, *J* = 14.3 Hz), 129.5, 129.1, 128.5, 127.6, 124.4 (d, *J* = 3.8 Hz), 117.1 (d, *J* = 21.0 Hz), 36.6, 34.2, 32.4

^{19}F NMR (377 MHz, Chloroform-*d*): δ -108.6

Methyl 2'-((3-phenylbicyclo[1.1.0]butan-1-yl)sulfonyl)-[1,1'-biphenyl]-4-carboxylate, 53

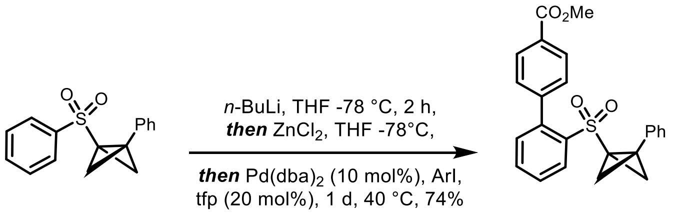

To a solution of **5** (32 mg, 0.12 mmol. 1.2 eq.) in THF (0.4 mL) at –78 °C was added *n*-BuLi (1.9 M in pentane, 64 µL, 0.12 mmol, 1.2 eq.). The reaction was stirred for 2 h before addition of a solution of $ZnCl_2$ (16 mg, 0.12 mmol, 1.20 eq.) in THF (0.5 mL) at –78 °C, and stirred for 5 min before bringing to rt. The solution of organozinc was transferred *via* syringe to a vial containing $Pd(dba)_2$ (11.5 mg, 0.02 mmol, 10 mol%), tfp (9.2 mg, 0.04 mmol, 20 mol%) and methyl 4-iodobenzoate (26 mg, 0.10 mmol, 1.0 eq.). The reaction mixture was stirred for 1 d at 40 °C, followed by cooling to room temperature, diluting with DCM (5 mL) and filtering. The organic phase was evaporated *in vacuo* and purified *via* flash chromatography (30-50% Et_2O in pentane) to afford **53** as a light yellow solid (36 mg, 0.07 mmol, 74%).

R_f = 0.33 (50% Et_2O in pentane)

MP: 120 °C (recrystallized from Et_2O)

IR (thin film, ν_{max} / cm^{-1}): 1721, 1277, 1252, 1147, 1101, 859, 766, 643

HRMS (ESI^+) calc. for $C_{24}H_{21}O_4S$ $[M+H]^+$ 401.1601, found 401.1597

^{1}H NMR (500 MHz, Chloroform-*d*): δ 8.25 (d, *J* = 8.3 Hz, 2H), 7.75 (td, *J* = 7.5, 1.4 Hz, 1H), 7.71-7.65 (m, 3H), 7.50-7.35 (m, 7H), 4.10 (s, 3H), 2.67 (s, 2H), 1.34 (s, 2H)

^{13}C NMR (101 MHz, Chloroform-*d*): δ 167.0, 144.2, 141.7, 140.4, 132.6, 132.3, 130.7, 130.7, 129.8, 128.9, 128.5, 128.3, 128.01, 127.7, 127.3, 52.4, 37.0, 33.9, 33.7

2,2-Difluoro-*N*,*N*-diisopropyl-3-phenylbicyclo[1.1.1]pentane-1-carboxamide, 54

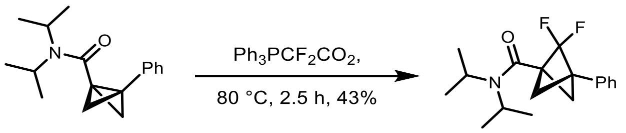

To a solution of **37** (26 mg, 0.10 mmol, 1.0 eq.) in mesitylene (1 mL) was added $Ph_3PCF_2CO_2$ (107 mg, 0.3 mmol, 3.0 eq.). The suspension was heated to 80 °C and stirred for 2.5 h before evaporating *in vacuo* and purifying *via* flash chromatography (5-15% EtOAc in pentane) to yield a colourless solid (13.3 mg, 0.04 mmol, 43%).

R$_f$ = 0.50 (15% EtOAc in pentane)

MP: 99 °C (recrystallized from Et_2O)

IR (thin film, ν_{max} / cm^{-1}): 2968, 1631, 1439, 1373, 1213, 1100, 711

HRMS (ESI^+) calc. for $C_{18}H_{24}ONF_2S$ $[M+H]^+$ 308.1820, found 308.1817

^{1}H NMR (500 MHz, Chloroform-*d*): δ 7.39-7.31 (m, 3H), 7.29 (dd, *J* = 8.0, 1.7 Hz, 2H), 4.26 (sep, *J* = 6.6 Hz, 1H), 3.42 (sep, *J* = 6.8 Hz, 1H), 2.62 (s, 2H), 2.11 (t, *J* = 10.6 Hz, 1H), 1.44 (d, *J* = 6.8 Hz, 6H), 1.23 (d, *J* = 6.7 Hz, 6H)

^{13}C NMR (126 MHz, Chloroform-*d*): δ 163.8, 132.6, 128.7, 128.3, 127.3, 123.9 (t, *J* = 296.6 Hz), 54.2 (t, *J* = 19.0 Hz), 53.3 (t, *J* = 19.5 Hz), 48.6, 46.4, 44.0 (t, *J* = 7.3 Hz), 21.1, 20.6

2,2-Difluoro-*N*,*N*-diisopropyl-3-(4-(trifluoromethyl)phenyl)bicyclo[1.1.1]pentane-1-carboxamide, 56; 2,2-Difluoro-*N*,*N*-diisopropyl-3-(4-(trifluoromethyl)phenyl)bicyclo[1.1.1]pentane-1-carboxamide, 57

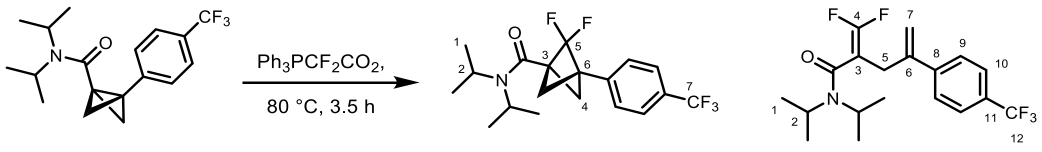

56, 33% **57**, 40%

To a solution of **40** (1.20 g, 3.67 mmol, 1.0 eq.) in mesitylene (37 mL) was added $Ph_3PCF_2CO_2$ (3.92 g, 11.0 mmol, 3.0 eq.). The suspension was heated to 80 °C and stirred for 3.5 h before cooling to rt, diluting with EtOAc (200 mL) and washing with water (100 mL x 2). The organic phase was dried with anhydrous $MgSO_4$, filtered and concentrated *in vacuo* to remove EtOAc (residual mesitylene). The residue was purified using a 1% NEt_3, 5% Et_2O in pentane preconditioned silica column, with 1% NEt_3, 5→30% Et_2O in pentane eluent, to afford **56** as a white solid (0.45 g, 1.21 mmol, 33%) and **57** as a yellow-green oil (0.55 g, 1.47 mmol, 40%).

2,2-Difluoro-*N*,*N*-diisopropyl-3-(4-(trifluoromethyl)phenyl)bicyclo[1.1.1]pentane-1-carboxamide, 56

R$_f$ = 0.39 (40% Et_2O in pentane)

MP: 135 °C (recrystallized from Et_2O)

IR (thin film, ν_{max} / cm^{-1}): 2971, 1632, 1450, 1325, 1128

HRMS (ESI$^+$) calc. for $C_{19}H_{23}ONF_5$ $[M+H]^+$ 376.1694, found 376.1692

^{1}H NMR (500 MHz, Chloroform-*d*): δ 7.62 (d, *J* = 8.0 Hz, 2H), 7.40 (d, *J* = 8.0 Hz, 2H), 4.24 (sep, *J* = 6.7 Hz, 1H, H2), 3.43 (sep, *J* = 6.7 Hz, 1H, H2), 2.65 (s, 2H, H4), 2.15 (t, *J* = 10.9 Hz, 2H, H4), 1.44 (d, *J* = 6.8 Hz, 6H, H1), 1.24 (d, *J* = 6.7 Hz, 6H, H1)

^{13}C NMR (126 MHz, Chloroform-*d*): δ 163.4, 136.5, 130.6 (q, *J* = 32.5 Hz), 127.7, 125.7 (q, *J* = 3.8 Hz), 123.7 (t, *J* = 296.3 Hz, C5), 123.0 (q, *J* = 272.8 Hz, C7), 54.4 (t, *J* = 18.9 Hz, C3), 52.9 (t, *J* = 19.6 Hz, C6), 48.7 (C2), 46.5 (C2), 44.0 (t, *J* = 7.1 Hz, C4), 21.1 (C1), 20.5 (C1).

^{19}F NMR (470 MHz, Chloroform-*d*): δ -62.7, -117.5

2,2-Difluoro-*N*,*N*-diisopropyl-3-(4-(trifluoromethyl)phenyl)bicyclo[1.1.1]pentane-1-carboxamide, 57

R$_f$ = 0.52 (40% Et_2O in pentane)

IR (thin film, ν_{max} / cm^{-1}): 2971, 1632, 1450, 1325, 1128

HRMS (ESI$^+$) calc. for $C_{19}H_{23}ONF_5$ $[M+H]^+$ 376.1694, found 376.1693

^{1}H NMR (500 MHz, Chloroform-*d*): δ 7.59 (d, *J* = 8.7 Hz, 2H), 7.56 (d, *J* = 8.7 Hz, 2H), 5.54 (s, 1H, H7), 5.34 (s, 1H, H7), 3.69 (sep, *J* = 6.9 Hz, 1H, H2), 3.51 (s, 2H, H5), 3.31 (sep, *J* = 6.7 Hz, 1H, H2), 1.37 (d, *J* = 6.8 Hz, 6H, H1), 0.93 (br, 6H, H1)

^{13}C NMR (126 MHz, Chloroform-*d*): δ 163.4 (dd, *J* = 7.4, 2.0 Hz), 153.0 (dd, *J* = 292.8, 288.1 Hz, C4), 142.9 (C6 or 8), 142.5 (C6 or 8), 130.1 (q, *J* = 32.6 Hz, C11), 126.4 (C9), 125.6 (q, *J* = 3.8 Hz, C10), 124.2 (q, *J* = 271.9 Hz, C12), 117.7 (C7), 88.9 (dd, *J* = 19.5, 16.2 Hz, C3), 50.8 (C2), 46.3 (C2), 31.8 (d, *J* = 2.4 Hz, C5), 20.9 (C1), 20.3 (C1)

^{19}F NMR (470 MHz, Chloroform-*d*): δ -62.7, -83.0 (d, *J* = 39.7 Hz), -91.8 (d, *J* = 39.7 Hz)

2,2-Difluoro-3-(4-(trifluoromethyl)phenyl)bicyclo[1.1.1]pentane-1-carboxylic acid, 58

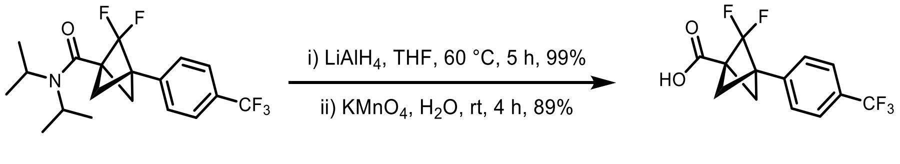

$LiAlH_4$ (7.6 mg, 0.20 mmol, 2.0 eq.) was suspended in THF (0.5 mL) and transferred to vial containing BCP (38 mg, 0.10 mmol, 1.0 eq.) *via* syringe at rt. The resulting suspension was heated to 60 °C for 5 h before cooling to room

temperature and diluting with THF (0.5 mL). Sat. aq. Na_2SO_4 (0.5 mL) was added dropwise with vigorous stirring. The mixture was stirred for 10 min before diluting with Et_2O and drying with anhydrous $MgSO_4$. The solution was filtered and solvent removed *in vacuo* to afford a clear oil, which was dissolved in 30% Et_2O in pentane and passed through a pipette of silica. The solvent was removed in vacuo to afford **56**-*amine* as a clear oil (36 mg, 0.10 mmol, 99%).

R_f = 0.6 (40% Et_2O in pentane); **^{1}H NMR** (400 MHz, Chloroform-*d*): δ 7.63-7.56 (m, 2H), 7.41-7.35 (m, 2H), 2.97 (sept, *J* = 6.6 Hz, 2H), 2.73 (s, 2H), 2.26 (s, 2H), 1.72 (t, *J* = 10.7, 10.2 Hz, 2H), 1.00 (d, *J* = 6.6 Hz, 12H); **^{19}F NMR** (377 MHz, Chloroform-*d*): δ -62.6, -123.3

To a vigorously stirred solution of **56**-*amine* (36 mg, 0.10 mmol, 1.0 eq.) in aq. NaOH (1%, 1 mL) was slowly added a solution of $KMnO_4$ (318 mg, 2.00 mmol, 20 eq.) in water (4 mL). After stirring for 4 h, sat. aq. $Na_2S_2O_3$ (1 mL) was added dropwise followed by dilution with water (25 mL) and filtering through Celite®. The filtrate was acidified to pH 1-2 with aq. HCl (1.2 M) and extracted with ethyl acetate (25 mL x 3). The combined organic phases were dried with anhydrous $MgSO_4$, filtered, and concentrated to afford a cream solid. The solid was triturated with a small amount of cold pentane (1 mL x 2) to yield **58** as a colourless solid (26 mg, 0.09 mmol, 89%).

MP: 179 °C (recrystallized from acetone)

IR (thin film, ν_{max} / cm^{-1}): 3059, 2360, 1713, 1327, 1153, 1126

HRMS (ESI^-) calc. for $C_{13}H_8O_2NF_5$ [$M-H^+$] 291.045 found 291.0449

^{1}H NMR (500 MHz, Chloroform-*d*): δ 7.64 (d, *J* = 8.1 Hz, 2H), 7.41 (d, *J* = 7.7 Hz, 2H), 2.72 (s, 2H), 2.21 (t, *J* = 10.1 Hz, 2H)

^{13}C NMR (126 MHz, Chloroform-*d*): δ 169.3, 135.7, 130.9 (q, *J* = 32.6 Hz), 127.7, 125.8 (q, *J* = 3.8 Hz), 124.0 (q, *J* = 272.2 Hz), 122.7 (t, *J* = 243.5 Hz), 54.7 (t, *J* = 19.4 Hz), 50.2 (t, *J* = 19.6 Hz), 43.5 (t, *J* = 6.9 Hz)

^{19}F NMR (471 MHz, Chloroform-*d*): δ -62.8, -120.7 (t, *J* = 10.2 Hz)

1.8 Synthesis of coupling partners and reagents

2,2-Difluoro-2-(triphenylphosphonio)acetate, 55

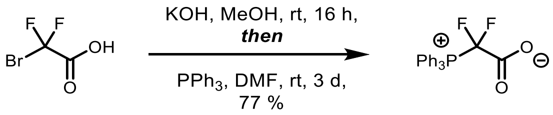

To a cooled solution of potassium hydroxide (2.76 g, 49.3 mmol, 1.0 eq.) in methanol (45 mL) at 0 °C was added ethyl bromodifluoroacetate (6.30 mL, 49.3 mmol, 1.0 eq.). The mixture was stirred at rt for 16 h then concentrated to give the potassium salt as a white powder, which was used without further purification. The salt was dissolved in DMF (56 mL) and PPh_3 (12.9 g, 49.3 mmol, 1.0 eq.) was added and the mixture stirred at rt for 3 d. The mixture was suction filtered, and the residual solid washed with DMF (2 x 5 mL), water (2 x 5 mL), Et_2O (2 x 5 mL) and dried under high vacuum to give **55** as a white solid (13.5 g, 37.8 mmol, 77%). Data identical to literature values.[8]

^{1}H NMR (400 MHz, methanol-*d*) δ 7.97-7.83 (m, 9H), 7.79-7.70 (m, 6H)

^{13}C NMR (101 MHz, methanol-*d*) 161.3 (d, *J* = 15.0 Hz), 135.6 (d, *J* = 3.2 Hz), 134.6 (d, *J* = 10.1 Hz), 130.1 (d, *J* = 13.1 Hz), 117.0 (d, *J* = 80.8 Hz), 115.2 (d, *J* = 85.9 Hz)

^{19}F NMR (377 MHz, methanol-*d*) δ -96.0 (d, *J* = 96.9 Hz)

^{31}P NMR (162 MHz, methanol-*d*) δ 27.1 (t, *J* = 96.6 Hz)

3-Iodophenyl trifluoromethanesulfonate, S8

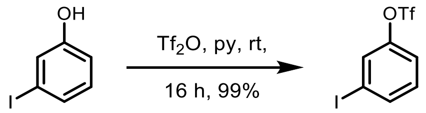

To a solution of 3-iodophenol (442 mg, 2.00 mmol, 1.0 eq.) in pyridine (2 mL) was added Tf_2O (0.38 mL, 2.20 mmol, 1.10 eq.) dropwise at 0 °C. The reaction mixture was stirred at 0 °C for 30 min, warmed to rt and stirred for 16 h. The reaction mixture was diluted with Et_2O (25 mL), washed with aq. 1 M HCl (20 mL x 2) and the aqueous phase was extracted with Et_2O. The organics were combined, dried with anhydrous $MgSO_4$, filtered, and concentrated to afford a dark oil. The oil was purified *via* flash chromatography (Et_2O in pentane) to yield **S8** as a yellow oil (700 mg, 1.98 mmol, 99%). Data identical to literature values.[9]

^{1}H NMR (400 MHz, Chloroform-*d*): δ 7.74 (dt, *J* = 7.8, 1.3 Hz, 1H), 7.63 (dd, *J* = 2.4, 1.6 Hz, 1H), 7.28 (dd, *J* = 8.4, 2.4 Hz, 1H), 7.19 (t, *J* = 8.1 Hz, 1H).

^{13}C NMR (101 MHz, Chloroform-*d*): δ 149.4, 137.8, 131.6, 130.6, 121.0, 118.9 (q, *J* = 320.7 Hz), 94.0.

***Tert*-butyl(3-iodophenoxy)dimethylsilane, S9**

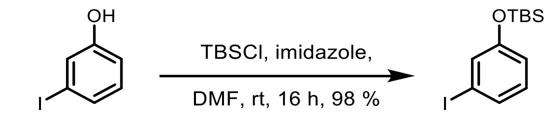

To a solution of 3-iodophenol (442 mg, 2.00 mmol, 1.0 eq.) and imidazole (136 mg, 2.00 mmol, 1.0 eq.) in DMF (2 ml) was added a solution of TBSCl (301 mg, 2.00 mmol, 1.0 eq.) in DMF (2 mL) dropwise at rt. The mixture was stirred for 16 h, then diluted with Et_2O (20 mL). The organic phase was washed with water (10 mL x 2), brine (10 mL), dried with anhydrous $MgSO_4$, filtered and concentrated to afford a yellow oil. The oil was purified *via* flash chromatography (10% Et_2O in pentane) to yield **S9** as a clear oil (532 mg, 1.96 mmol, 98%). Data identical to literature values.[10]

^{1}H NMR (400 MHz, Chloroform-*d*): δ 7.29 (ddd, *J* = 7.8, 1.7, 1.0 Hz, 1H), 7.21 (dd, *J* = 2.3, 1.6 Hz, 1H), 6.94 (t, *J* = 8.0 Hz, 1H), 6.79 (ddd, *J* = 8.2, 2.4, 1.0 Hz, 1H), 0.98 (s, 9H), 0.20 (s, 6H).

^{13}C NMR (101 MHz, Chloroform-*d*): δ 156.6, 130.9, 130.7, 129.7, 119.7, 94.3, 26.0, 18.4, -4.2

***(Z)-1-iodooct-1-ene*, S9**

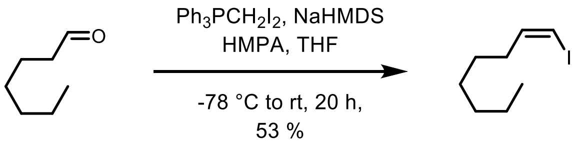

A solution of NaHMDS in THF (2.20 mL, 1.00 M, 2.20 mmol, 1.10 eq.) was added to a suspension of $Ph_3PCH_2I_2$ (1.17 g, 2.20 mmol, 1.10 eq.) in THF (10 mL) at rt. The reaction was stirred for 20 min before addition of HMPA (1.80 mL, 10.0 mmol, 5.00 eq.). The reaction was stirred for 10 min before cooling to –78 °C and addition of *n*-heptanal (285 µL, 2.00 mmol, 1.0 eq.). The mixture was stirred for 1 h at –78 °C before warming to rt and stirring for an additional 20 h at rt. The reaction was quenched by addition of sat. aq. $NaHCO_3$ (50 mL), then the aqueous phase was extracted with Et_2O (50 mL x 2). The combined organic phases were dried with anhydrous $MgSO_4$, filtered and concentrated to afford a yellow oil. The oil was purified *via* flash chromatography (pentane) to yield **S9** as a clear oil (251 mg, 1.05 mmol, 53%). Data identical to literature values.[11]

^{1}H NMR (400 MHz, Chloroform-*d*): δ 6.20-6.14 (m, 2H), 2.19-2.09 (m, 2H), 1.48-1.23 (m, 8H), 0.95-0.83 (m, 3H).

^{13}C NMR (101 MHz, Chloroform-*d*): δ 141.7, 82.2, 34.9, 31.8, 28.9, 28.1, 22.7, 14.2

4-(Iodoethynyl)benzonitrile, S11

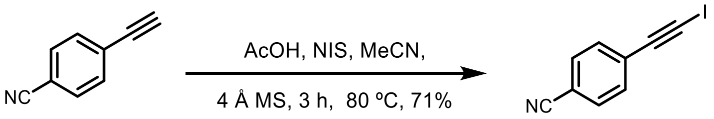

To a solution of 4-ethynylbenzonitrile (200 mg, 1.57 mmol, 1.0 eq.) and AcOH (0.12 mL, 2.00 mmol, 1.30 eq.) in MeCN (7.5 mL) was added 4 Å MS (200 mg) and *N*-iodosuccinimide (389 mg, 1.73 mmol, 1.10 eq.) at rt. The reaction mixture was heated to 80 °C for 3 h. The reaction mixture was then cooled to room temperature, diluted with EtOAc (20 mL), washed with sat. aq. $Na_2S_2O_3$ (10 mL), dried with anhydrous $MgSO_4$, filtered and evaporated *in vacuo* to afford a brown oil. The oil was purified *via* flash chromatography (10→20% Et_2O in pentane) to yield the title compound as a colourless solid (281 mg, 1.11 mmol, 71%). Data identical to literature values.[12]

^{1}H NMR (400 MHz, Chloroform-*d*): δ 7.60 (d, *J* = 8.7 Hz, 2H), 7.51 (d, *J* = 8.6 Hz, 2H)

^{13}C NMR (101 MHz, Chloroform-*d*): δ 133.0, 132.1, 128.2, 118.4, 112.3, 92.7, 13.1

***Tert*-butyl 5-iodo-1*H*-indole-1-carboxylate, S12**

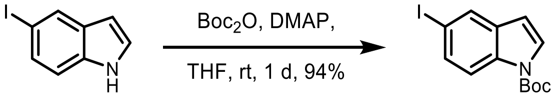

To a solution of 5-iodoindole (242 mg, 1.00 mmol, 1.0 eq.) in THF (8 mL) was added DMAP (12.0 mg, 0.1 mmol, 10 mol%) and Boc_2O (290 µL, 1.1 mmol, 1.10 eq.) at rt. The reaction was stirred for 1 d at rt before quenching with water (20 mL) and extracting with Et_2O (20 mL x 3). The combined organic phases were dried with anhydrous $MgSO_4$, filtered, and evaporated *in vacuo*. The residue was purified *via* flash chromatography (10% Et_2O in pentane) to yield **S12** as a clear oil (323 mg, 0.94 mmol, 94%). Data identical to literature values.[13]

^{1}H NMR (400 MHz, Chloroform-*d*): δ 7.91 (d, *J* = 8.5 Hz, 1H), 7.90-7.89 (m, 1H), 7.57 (dd, *J* = 8.8, 1.8 Hz, 1H), 7.55 (d, *J* = 3.8 Hz, 1H), 6.49 (dd, *J* = 3.7, 0.8 Hz, 1H), 1.67 (s, 9H)

^{13}C NMR (101 MHz, Chloroform-*d*): δ 149.6, 134.6, 133.0, 132.8, 129.9, 126.8, 117.2, 106.4, 86.8, 84.3, 28.3

Methyl (*S*)-2-((tert-butoxycarbonyl)amino)-3-(4-iodophenyl)propanoate, S13

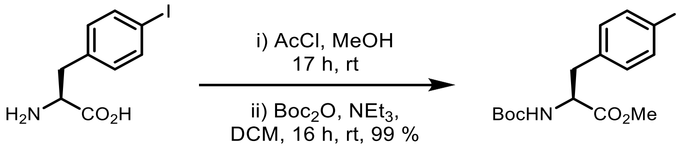

AcCl (2.5 mL, 35.2 mmol, 10.2 eq.) was added dropwise to MeOH (15 mL) at 0 °C and stirred for 10 min before addition of 4-iodo-L-phenylalanine (1.00 g, 3.44 mmol, 1.0 eq.). The reaction mixture was stirred for 17 h at rt,

then concentrated to afford a colourless solid which was redissolved in DCM (15 mL). To this solution was added NEt_3 (1.20 mL, 8.60 mmol, 2.50 eq.) and Boc anhydride (1.10 g, 5.22 mmol, 1.50 eq.) and the reaction was stirred for 16 h at room temperature. The mixture was then diluted with DCM, washed with water and extracted with DCM. The combined organic phases were washed with brine, dried with anhydrous $MgSO_4$, filtered and concentrated *in vacuo*. The residue was purified by flash chromatography (EtOAc/pentane) to yield **S13** as a yellow oil (1.40 g, 3.41 mmol, 99%). Data identical to literature values.[14]

^{1}H NMR (400 MHz, Chloroform-*d*): δ 7.61 (d, *J* = 8.4 Hz, 2H), 6.87 (d, *J* = 8.3 Hz, 2H), 4.97 (d, *J* = 8.3 Hz, 1H), 4.56 (q, *J* = 6.7 Hz, 1H), 3.71 (s, 3H), 3.18-2.87 (m, 2H), 1.42 (s, 9H)

^{13}C NMR (101 MHz, Chloroform-*d*): δ 172.2, 155.2, 137.8, 135.9, 131.5, 92.7, 80.2, 54.3, 52.5, 38.1, 28.4

(*S*)-2-((*Tert*-butoxycarbonyl)amino)-3-methoxy-3-oxopropyl 4-iodobenzoate, S14

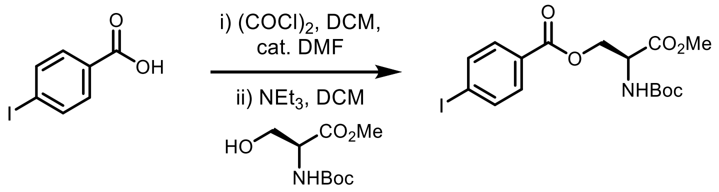

To a solution of 4-iodobenzoic acid (298 mg, 1.20 mmol, 1.0 eq.) in DCM (5 mL) was added oxalyl chloride (375 µL, 4.40 mmol, 3.70 eq.), followed by 2 drops of DMF. The reaction was stirred for 45 min at room temperature, then the volatiles were removed under a stream of nitrogen. The residue was placed under high vacuum for 15 min. The crude acyl chloride was dissolved in DCM (5 mL), taken up *via* syringe and added dropwise to a solution of Boc-Ser-OMe (438 mg, 2.00 mmol, 1.70 eq.) and NEt_3 (0.56 mL, 4.00 mmol, 3.30 eq.) in DCM (10 mL) at 0 °C. The reaction mixture was stirred for 20 h, before being diluted with EtOAc (50 mL), and washed with water (25 mL x 2) and sat. aq. $NaHCO_3$ (25 mL). The organic phase was dried with anhydrous $MgSO_4$, filtered, and evaporated *in vacuo* to yield a yellow oil. The oil was purified *via* flash chromatography (20% EtOAc in pentane) to yield **S14** a yellow oil (0.74 g, 0.98 mmol, 82%). Data identical to literature values.[15]

^{1}H NMR (400 MHz, Chloroform-*d*): δ 7.83-7.76 (dt, *J* = 8.6 Hz, 1.8, 2H), 7.73-7.65 (dt, *J* = 8.6 Hz, 1.9, 2H), 5.37 (d, *J* = 8.2 Hz, 1H), 4.74-4.66 (m, 1H), 4.59 (d, *J* = 4.0 Hz, 1H), 3.77 (s, 3H), 1.44 (s, 9H)

^{13}C NMR (101 MHz, Chloroform-*d*): δ 170.4, 165.7, 155.2, 138.0, 131.2, 129.0, 101.4, 80.6, 65.3, 53.1, 53.0, 28.4

((3a*R*,5*R*,5a*S*,8a*S*,8b*R*)-2,2,7,7-tetramethyltetrahydro-5*H*-bis([1,3]dioxolo)[4,5-b:4',5'-d]pyran-5-yl)methyl 4-iodobenzoate, S15

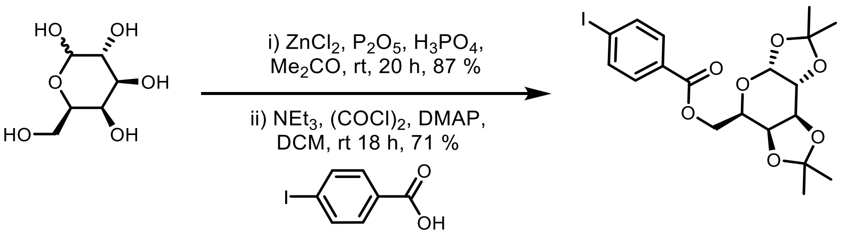

To a suspension of D-(+)-galactose (1.80 g, 10.0 mmol, 1.0 eq.) in acetone (40 mL) was added $ZnCl_2$ (1.85 g, 13.6 mmol, 1.40 eq.) followed by P_2O_5 (370 mg, 2.6 mmol, 0.3 eq.) and H_3PO_4 (730 mg, 7.4 mmol, 0.7 eq.), and the mixture was stirred at room temperature for 20 h. The reaction was quenched by addition of sat. aq. Na_2CO_3 (100 mL) and reduced in vacuo (removing acetone). The residual aqueous phase was extracted with Et_2O (100 mL x 2), the organic phases were combined, dried with anhydrous $MgSO_4$ and evaporated in vacuo to yield a yellow oil (D-(+)-Galactose-*diketal*) (2.26 g, 8.68 mmol, 87%).

To a solution of 4-Iodobenzoic acid (595 mg, 2.40 mmol, 1.20 eq.) in DCM (15 mL) was added oxalyl chloride (375 µL, 4.00 mmol, 2.00 eq.), followed by 2 drops of DMF, and the mixture was stirred for 45 min at room temperature. The volatiles were removed under a stream of nitrogen, and the residue placed under high vacuum for 15 min. The crude acyl chloride was dissolved in DCM (5 mL), taken up *via* syringe and added dropwise to a solution of D-(+)-galactose diketal (520 mg, 2.00 mmol, 1.0 eq.), DMAP (49.0 mg, 0.40 mmol, 20 mol%) and NEt_3 (0.56 mL, 4.00 mmol, 4.00 eq.) in DCM (10 mL) at 0 °C. The reaction mixture was stirred for 18 h before being diluted with EtOAc (50 mL), washed with water (20 mL x 2) and sat. aq. $NaHCO_3$ (20 mL). The organic phase was dried with anhydrous $MgSO_4$, filtered, and concentrated to yield a yellow oil. The oil was purified *via* flash chromatography (15% EtOAc in pentane) to yield **S15** as a colourless solid (700 mg, 1.42 mmol, 71%). Data identical to literature values.[16]

^{1}H NMR (400 MHz, Chloroform-*d*): δ 7.79 (d, *J* = 8.6 Hz, 2H), 7.75 (d, *J* = 8.6 Hz, 2H), 5.56 (d, *J* = 4.9 Hz, 1H), 4.65 (dd, *J* = 7.9, 2.5 Hz, 1H), 4.52 (dd, *J* = 11.6, 4.7 Hz, 1H), 4.41 (dd, *J* = 11.5, 7.6 Hz, 1H), 4.34 (dd, *J* = 5.0, 2.5 Hz, 1H), 4.31 (dd, *J* = 7.9, 1.9 Hz, 1H), 4.16 (ddd, *J* = 7.7, 4.6, 1.9 Hz, 1H), 1.50 (s, 3H), 1.47 (s, 3H), 1.35 (s, 3H), 1.33 (s, 3H)

^{13}C NMR (101 MHz, Chloroform-*d*): δ 166.1, 137.9, 131.3, 129.7, 109.9, 109.0, 101.0, 96.5, 71.3, 70.9, 70.7, 66.3, 64.3, 26.2, 26.1, 25.1, 24.7

(8*R*,9*S*,13*S*,14*S*)-13-methyl-17-oxo-7,8,9,11,12,13,14,15,16,17-decahydro-6*H*-cyclopenta[a]phenanthren-3-yl trifluoromethanesulfonate, S16

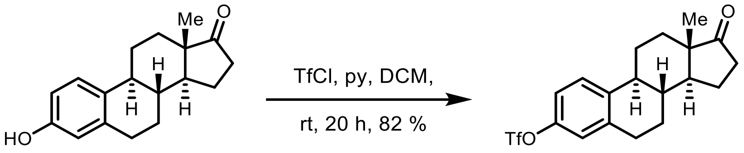

To a suspension of estrone (270 mg, 1.00 mmol, 1.0 eq.) in DCM (5 mL) at 0 °C was added py (81 µL, 1.00 mmol, 1.0 eq.) followed by TfCl (128 µl, 1.20 mmol, 1.20 eq.) dropwise. The reaction was stirred for 15 min at 0 °C before warming to rt and stirring for 20 h. The reaction mixture was diluted with EtOAc (20 mL), washed with aq. 1 M HCl (10 mL) and sat. aq. $NaHCO_3$ (10 mL). The organic phase was dried with anhydrous $MgSO_4$, filtered and evaporated *in vacuo* to afford a yellow oil. The oil was purified *via* flash chromatography (20→40% EtOAc in pentane) to yield **S16** (329 mg, 0.82 mmol, 82%) as a colourless solid. Data identical to literature values.[17]

^{1}H NMR (400 MHz, Chloroform-*d*): δ 7.34 (d, *J* = 1.2 Hz, 1H), 7.04 (dd, *J* = 8.6, 2.8 Hz, 1H), 6.99 (d, *J* = 2.5 Hz, 1H), 2.98-2.90 (m, 2H), 2.52 (dd, 1H), 2.45-2.37 (m, 1H), 2.30 (td, *J* = 10.6, 4.3 Hz, 1H), 2.21-2.01 (m, 3H), 2.01-1.95 (m, 1H), 1.71-1.38 (m, 6H), 0.92 (s, 3H)

^{13}C NMR (101 MHz, Chloroform-*d*): δ 220.5, 147.7, 140.4, 139.4, 127.3, 121.4, 118.8 (q, *J* = 322.3 Hz), 118.5, 50.5, 48.0, 44.2, 37.9, 36.0, 31.6, 29.5, 26.2, 25.8, 21.7, 13.9

2. X-Ray crystallography

For **11**, **14**, **16** and **18**: Single crystal X-ray diffraction data were collected using an Rigaku Oxford Diffraction SuperNova diffractometer fitted with an Oxford Cryosystems Cryostream 700 plus open flow nitrogen cooling device.[18] The CrysAlisPro software was used for data collection and integration. The structure was solved using charge flipping[19] with SuperFlip method[20] within the CRYSTALS suite.[21] The structures were then modified, improved and optimised by full-matrix least squares on F^2 as per the SI (CIF). Full refinement details are given in the Supporting Information (CIF); Crystallographic data have been deposited with the Cambridge Crystallographic Data Centre (CCDC 2074459-63) and can be obtained via www. ccdc.cam.ac.uk/data_request/cif.

Compound **11** has a phase transition between 300 K and 150 K. At 300 K there is a single molecule in the asymmetric cell, *i.e.* Z'=1. At 150 K the cell has tripled along the ***b***-axis and there are three molecules in the asymmetric cell, *i.e.* Z'=3. No further studies were performed to find the exact phase transition temperature.

2.1 Crystal data and structure refinement for 11 collected at 300K

Empirical formula	C16 H14 O2 S	
Formula weight	270.35	
Temperature	300 K	
Wavelength	1.54184 Å	
Crystal system	Orthorhombic	
Space group	$P\ 2_1\ 2_1\ 2_1$	
Unit cell dimensions	a = 6.2560(3) Å	α= 90°.
	b = 7.6732(3) Å	β= 90°.
	c = 28.1296(8) Å	γ = 90°.
Volume	1350.32(9) $Å^3$	
Z	4	
Density (calculated)	1.330 Mg/m^3	
Absorption coefficient	2.081 mm^{-1}	
F(000)	568	
Crystal size	0.14 x 0.10 x 0.08 mm^3	
Theta range for data collection	3.142 to 77.094°.	
Index ranges	-6<=h<=7, -9<=k<=8, -35<=l<=25	
Reflections collected	4335	
Independent reflections	2516 [R(int) = 0.018]	
Completeness to theta = 74.781°	99.5 %	
Absorption correction	Semi-empirical from equivalents	
Max. and min. transmission	0.85 and 0.82	
Refinement method	Full-matrix least-squares on F^2	
Data / restraints / parameters	2515 / 0 / 174	
Goodness-of-fit on F^2	1.0021	
Final R indices [I>2sigma(I)]	R1 = 0.0343, wR2 = 0.0884	
R indices (all data)	R1 = 0.0389, wR2 = 0.0947	
Absolute structure parameter	-0.032(12)	
Extinction coefficient	20(4)	
Largest diff. peak and hole	0.13 and -0.13 e.$Å^{-3}$	

2.2 Crystal data and structure refinement for 11 collected at 150K

Empirical formula	C16 H14 O2 S	
Formula weight	270.35	
Temperature	150 K	
Wavelength	1.54184 Å	
Crystal system	Orthorhombic	
Space group	P 2_1 2_1 2_1	
Unit cell dimensions	a = 6.19930(10) Å	α= 90°.
	b = 22.8971(3) Å	β= 90°.
	c = 27.5542(3) Å	γ = 90°.
Volume	3911.21(9) $Å^3$	
Z	12	
Density (calculated)	1.377 Mg/m^3	
Absorption coefficient	2.156 mm^{-1}	
F(000)	1704	
Crystal size	0.22 x 0.18 x 0.04 mm^3	
Theta range for data collection	3.744 to 76.292°.	
Index ranges	-7<=h<=7, -28<=k<=27, -34<=l<=34	
Reflections collected	22609	
Independent reflections	8095 [R(int) = 0.033]	
Completeness to theta = 74.767°	99.6 %	
Absorption correction	Semi-empirical from equivalents	
Max. and min. transmission	0.92 and 0.83	
Refinement method	Full-matrix least-squares on F^2	
Data / restraints / parameters	8095 / 0 / 515	
Goodness-of-fit on F^2	1.0069	
Final R indices [I>2sigma(I)]	R1 = 0.0314, wR2 = 0.0783	
R indices (all data)	R1 = 0.0342, wR2 = 0.0824	
Absolute structure parameter	0.028(6)	
Largest diff. peak and hole	0.32 and -0.26 e.$Å^{-3}$	

2.3 Crystal data and structure refinement for 14

Empirical formula	C18 H16 O4 S	
Formula weight	328.39	
Temperature	150 K	
Wavelength	1.54184 Å	
Crystal system	Monoclinic	
Space group	P 2_1/c	
Unit cell dimensions	a = 7.22830(10) Å	α= 90°.
	b = 19.74990(10) Å	β= 90.4390(5)°.
	c = 21.88370(10) Å	γ = 90°.
Volume	3123.99(5) $Å^3$	
Z	8	
Density (calculated)	1.396 Mg/m^3	
Absorption coefficient	2.001 mm^{-1}	
F(000)	1376	
Crystal size	0.20 x 0.18 x 0.10 mm^3	
Theta range for data collection	4.040 to 76.500°.	
Index ranges	-9<=h<=9, -24<=k<=23, -27<=l<=27	
Reflections collected	86693	
Independent reflections	6514 [R(int) = 0.028]	
Completeness to theta = 76.500°	99.5 %	
Absorption correction	Semi-empirical from equivalents	
Max. and min. transmission	0.82 and 0.71	
Refinement method	Full-matrix least-squares on F^2	
Data / restraints / parameters	6513 / 0 / 416	
Goodness-of-fit on F^2	0.9991	
Final R indices [I>2sigma(I)]	R1 = 0.0285, wR2 = 0.0820	
R indices (all data)	R1 = 0.0298, wR2 = 0.0832	
Extinction coefficient	21(3)	
Largest diff. peak and hole	0.30 and -0.37 e.$Å^{-3}$	

2.4 Crystal data and structure refinement for 16

Empirical formula	C16 H13 F O2 S	
Formula weight	288.34	
Temperature	150 K	
Wavelength	1.54184 Å	
Crystal system	Monoclinic	
Space group	P c	
Unit cell dimensions	a = 7.5201(2) Å	α= 90°.
	b = 6.1363(2) Å	β= 93.778(2)°.
	c = 29.1109(7) Å	γ = 90°.
Volume	1340.42(7) $Å^3$	
Z	4	
Density (calculated)	1.429 Mg/m^3	
Absorption coefficient	2.245 mm^{-1}	
F(000)	600	
Crystal size	0.20 x 0.10 x 0.05 mm^3	
Theta range for data collection	3.043 to 77.111°.	
Index ranges	-7<=h<=9, -7<=k<=7, -36<=l<=33	
Reflections collected	11889	
Independent reflections	3740 [R(int) = 0.033]	
Completeness to theta = 75.569°	99.6 %	
Absorption correction	Semi-empirical from equivalents	
Max. and min. transmission	0.89 and 0.86	
Refinement method	Full-matrix least-squares on F^2	
Data / restraints / parameters	3738 / 2 / 362	
Goodness-of-fit on F^2	1.0019	
Final R indices [I>2sigma(I)]	R1 = 0.0295, wR2 = 0.0734	
R indices (all data)	R1 = 0.0318, wR2 = 0.0754	
Absolute structure parameter	0.008(14)	
Largest diff. peak and hole	0.18 and -0.21 e.$Å^{-3}$	

2.5 Crystal data and structure refinement for 18

Empirical formula	C17 H16 O2 S	
Formula weight	284.38	
Temperature	150 K	
Wavelength	1.54184 Å	
Crystal system	Monoclinic	
Space group	P 2_1/n	
Unit cell dimensions	a = 5.91440(10) Å	α= 90°.
	b = 16.5154(2) Å	β= 95.2702(14)°.
	c = 14.7712(2) Å	γ = 90°.
Volume	1436.73(4) $Å^3$	
Z	4	
Density (calculated)	1.315 Mg/m^3	
Absorption coefficient	1.981 mm^{-1}	
F(000)	600	
Crystal size	0.25 x 0.10 x 0.08 mm^3	
Theta range for data collection	4.025 to 77.086°.	
Index ranges	-7<=h<=7, -13<=k<=20, -18<=l<=18	
Reflections collected	11872	
Independent reflections	3022 [R(int) = 0.022]	
Completeness to theta = 75.545°	99.6 %	
Absorption correction	Semi-empirical from equivalents	
Max. and min. transmission	0.85 and 0.77	
Refinement method	Full-matrix least-squares on F^2	
Data / restraints / parameters	3022 / 0 / 182	
Goodness-of-fit on F^2	1.0022	
Final R indices [I>2sigma(I)]	R1 = 0.0295, wR2 = 0.0806	
R indices (all data)	R1 = 0.0308, wR2 = 0.0820	
Extinction coefficient	24(4)	
Largest diff. peak and hole	0.35 and -0.33 e.$Å^{-3}$	

3. References

1 J. M. Lopchuk, K. Fjelbye, Y. Kawamata, L. R. Malins, C.-M. Pan, R. Gianatassio, J. Wang, L. Prieto, J. Bradow, T. A. Brandt, M. R. Collins, J. Elleraas, J. Ewanicki, W. Farrell, O. O. Fadeyi, G. M. Gallego, J. J. Mousseau, R. Oliver, N. W. Sach, J. K. Smith, J. E. Spangler, H. Zhu, J. Zhu and P. S. Baran, *J. Am. Chem. Soc.*, 2017, **139**, 3209-3226.

2 G. Ernouf, E. Chirkin, L. Rhyman, P. Ramasami and J.-C. Cintrat, *Angew. Chem. Int. Ed.*, 2020, **59**, 2618-2622.

3 H. K. Hall, C. D. Smith, E. P. Blanchard, S. C. Cherkofsky and J. B. Sieja, *J. Am. Chem. Soc.*, 1971, **93**, 121-130.

4 V. A. Vasin and V. V. Razin, *Synlett*, 2001, 0658-0660.

5 A. Frischmuth, M. Fernández, N. M. Barl, F. Achrainer, H. Zipse, G. Berionni, H. Mayr, K. Karaghiosoff and P. Knochel, *Angew. Chem. Int. Ed.*, 2014, **53**, 7928-7932.

6 G. C. Clososki, C. J. Rohbogner and P. Knochel, *Angew. Chem. Int. Ed.*, 2007, **46**, 7681-7684.

7 M. Ociepa, A. J. Wierzba, J. Turkowska and D. Gryko, *J. Am. Chem. Soc.*, 2020, **142**, 5355-5361.

8 J. Zheng, J. Cai, J.-H. Lin, Y. Guo and J.-C. Xiao, *Chem. Commun.*, 2013, **49**, 7513-7515.

9 M. J. Cabrera-Afonso, Z.-P. Lu, C. B. Kelly, S. B. Lang, R. Dykstra, O. Gutierrez and G. A. Molander, *Chem. Sci.*, 2018, **9**, 3186-3191.

10 O. Bobileva, M. Ikaunieks, G. Duburs, I. Mandrika, R. Petrovska, J. Klovins and E. Loza, *Bioorg. Med. Chem.*, 2017, **25**, 4314-4329.

11 X.-L. Lu, M. Shannon, X.-S. Peng and H. N. C. Wong, *Org. Lett.*, 2019, **21**, 2546-2549.

12 M. Yao, J. Zhang, S. Yang, E. Liu and H. Xiong, *Synlett*, 2020, **31**, 1102-1106.

13 S. A. Green, J. L. M. Matos, A. Yagi and R. A. Shenvi, *J. Am. Chem. Soc.*, 2016, **138**, 12779-12782.

14 M. H. Jeon, B. P. Mathew, M. R. Kuram, K. Myung and S. Y. Hong, *Org. Biomol. Chem.*, 2016, **14**, 11518-11524.

15 J. Dadová, S. R. G. Galan and B. G. Davis, *Curr. Opin. Chem. Biol.*, 2018, **46**, 71-81.

16 Q. Bai, Z. Bai and H. Wang, *Org. Lett.*, 2019, **21**, 8225-8228.

17 Y. H. Lee and B. Morandi, *Angew. Chem. Int. Ed.*, 2019, **58**, 6444-6448.

18 J. Cosier and A. M. Glazer, *J. Appl. Cryst.*, 1986, **19**, 105-107.

19 (a) L. Palatinus and A. J. van der Lee, *Appl. Crystallogr.* 2008, **41**, 975–984. (b) L. Palatinus, *Acta Crystallogr. Sect. B Struct. Sci. Cryst. Eng. Mater.* 2013 **69**, 1–16.

20 L. Palatinus, G. J. Chapuis, *Appl. Crystallogr.* 2007 **40**, 786–790.

21 (a) P. Parois, R.I. Cooper and A.L. Thompson *Chem. Cent. J.*, 2015, **9**:30. (b) R. I. Cooper, A. L. Thompson and D. J. Watkin, *J. Appl. Cryst.* 2010, **43**, 1100-1107.

4. Copies of NMR spectra

1-(phenylsulfonyl)bicyclo[1.1.0]butane, 8a

^{1}H NMR (400 MHz, Chloroform-*d*)

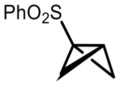

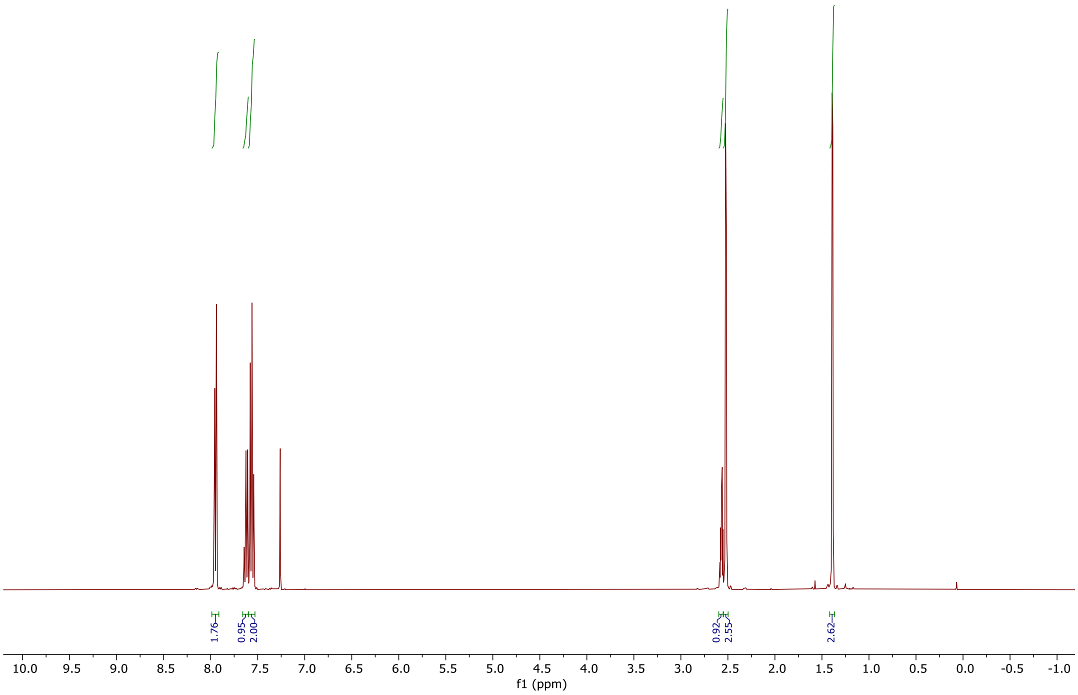

^{13}C NMR (101 MHz, Chloroform-*d*)

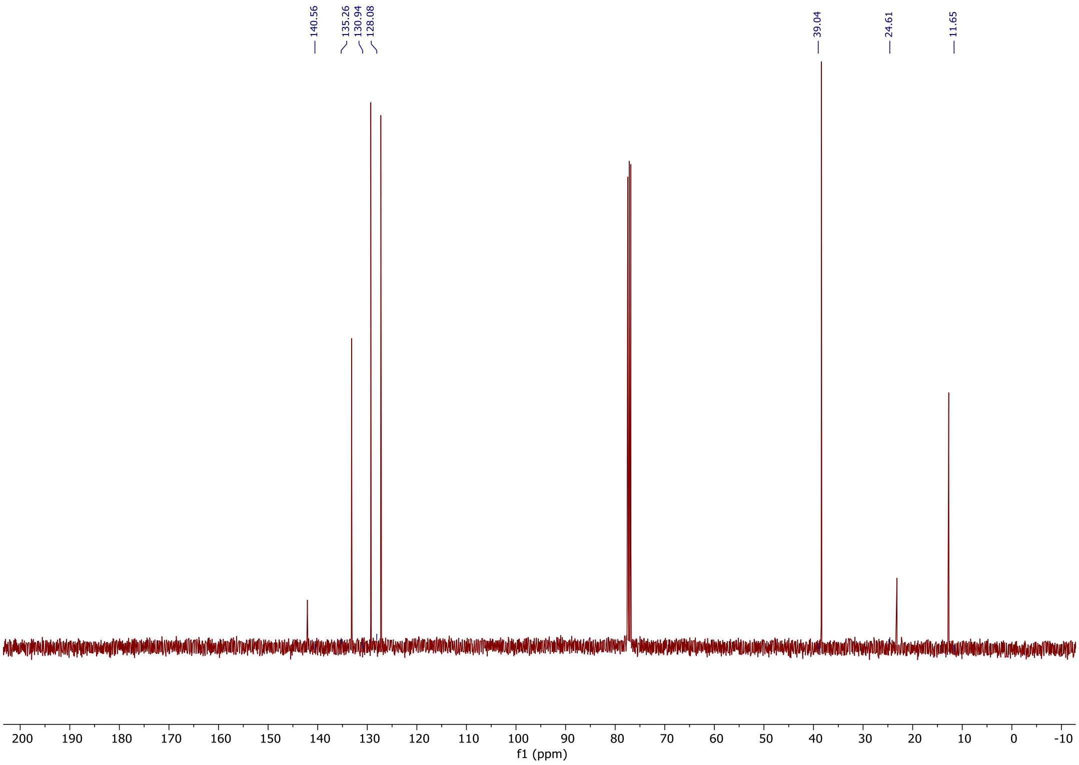

1-(phenylsulfinyl)bicyclo[1.1.0]butane, 8b

^{1}H NMR (400 MHz, Chloroform-*d*)

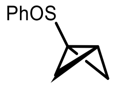

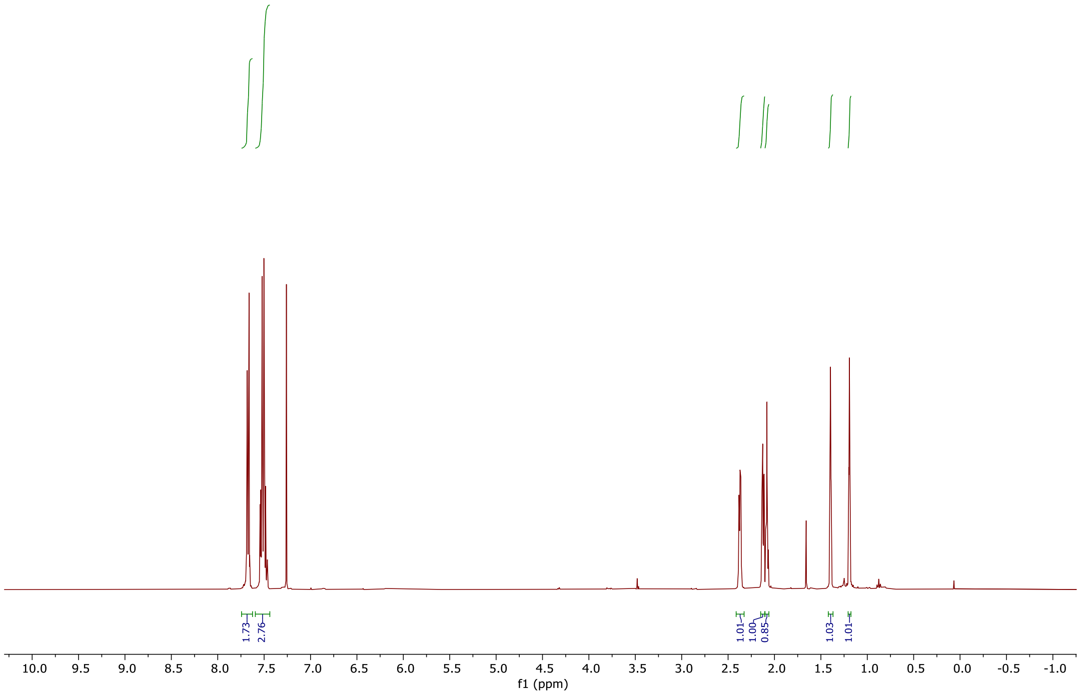

^{13}C NMR (101 MHz, Chloroform-*d*)

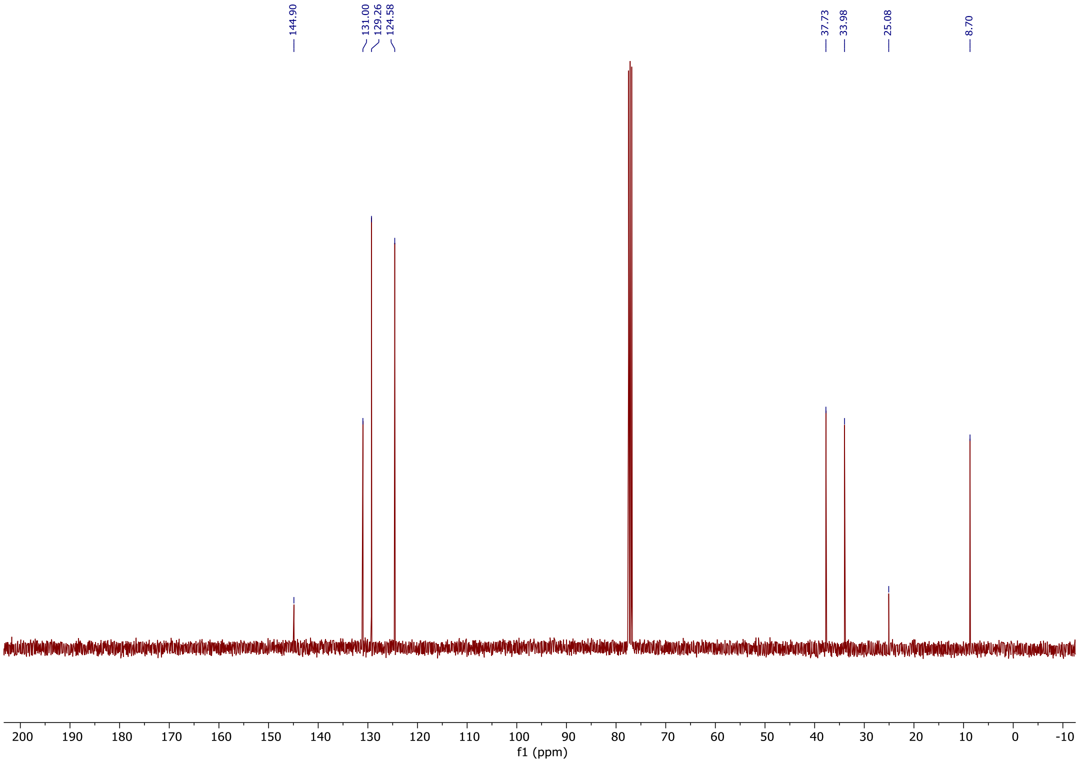

***N*,*N*-diisopropylbicyclo[1.1.0]butane-1-carboxamide, 8c**

^{1}H NMR (500 MHz, Chloroform-*d*)

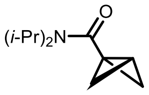

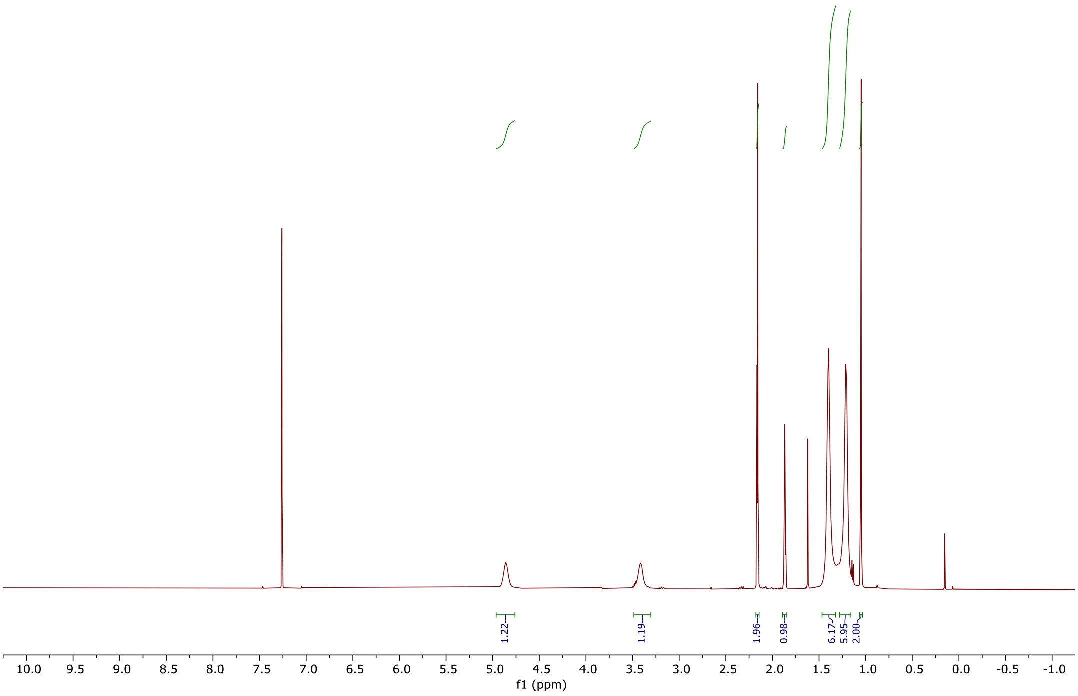

^{13}C NMR (126 MHz, Chloroform-*d*)

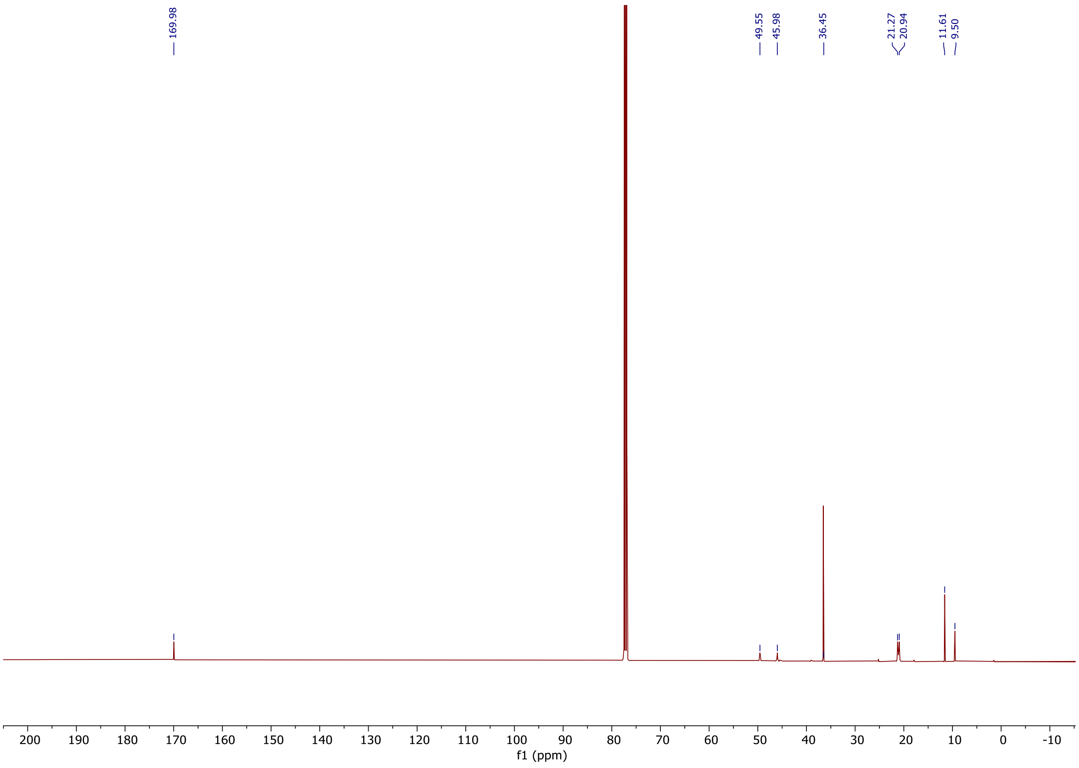

^{1}H COSY (500 MHz, Chloroform-*d*)

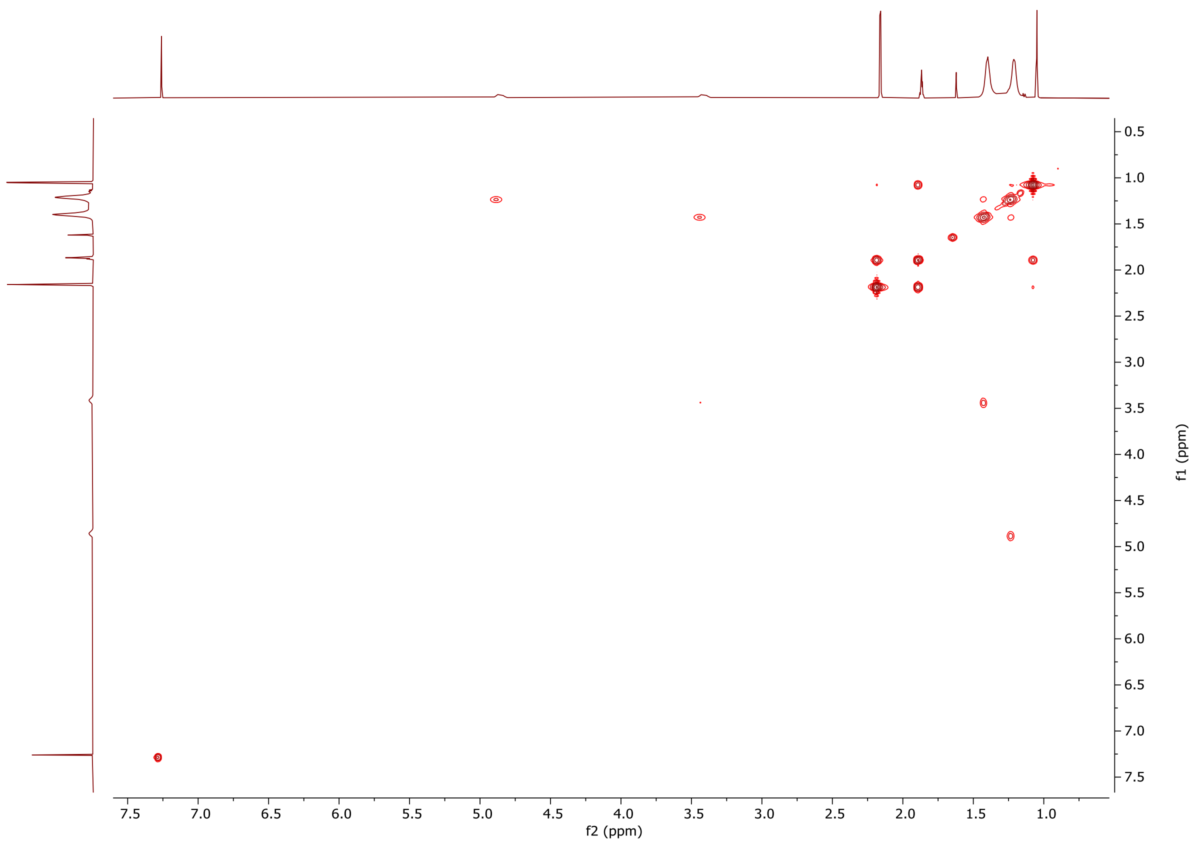

^{1}H/^{13}C HSQC (500/126 MHz, Chloroform-*d*)

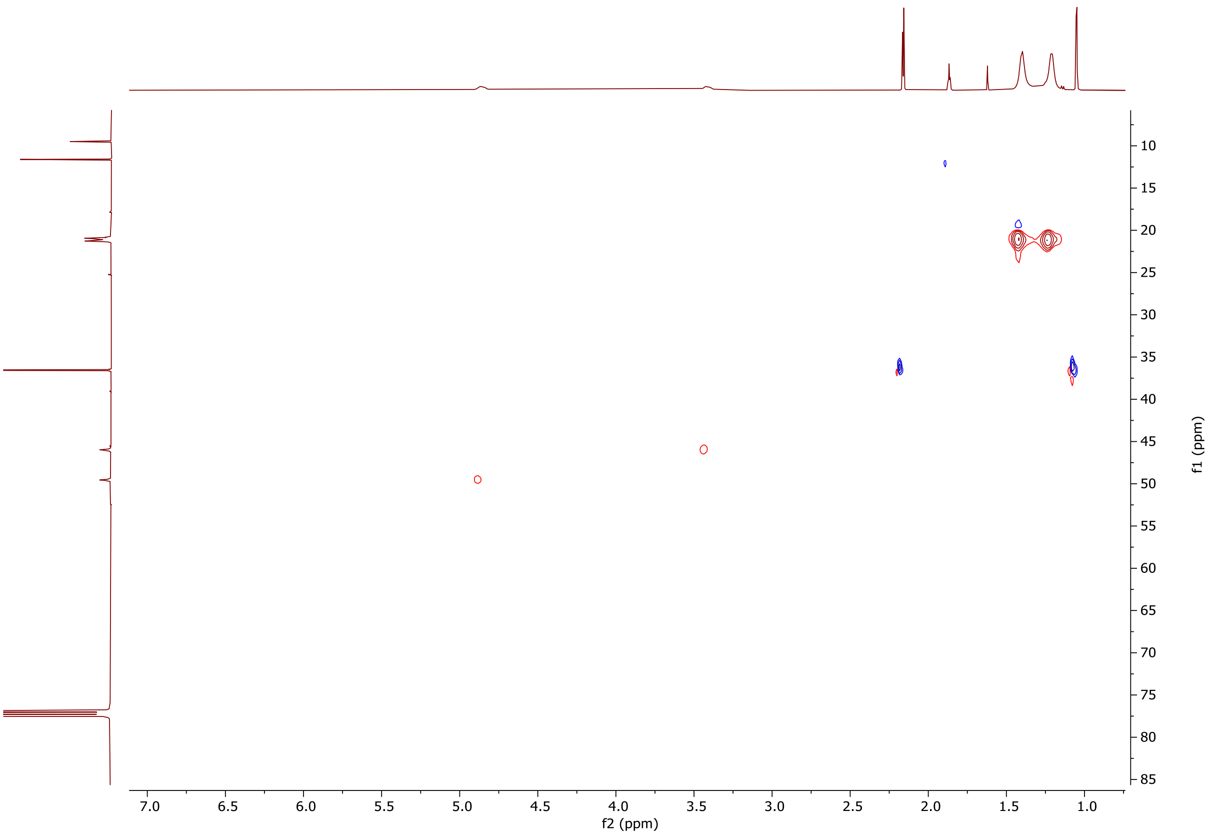

^{1}H/^{13}C HMBC (500/126 MHz, Chloroform-*d*)

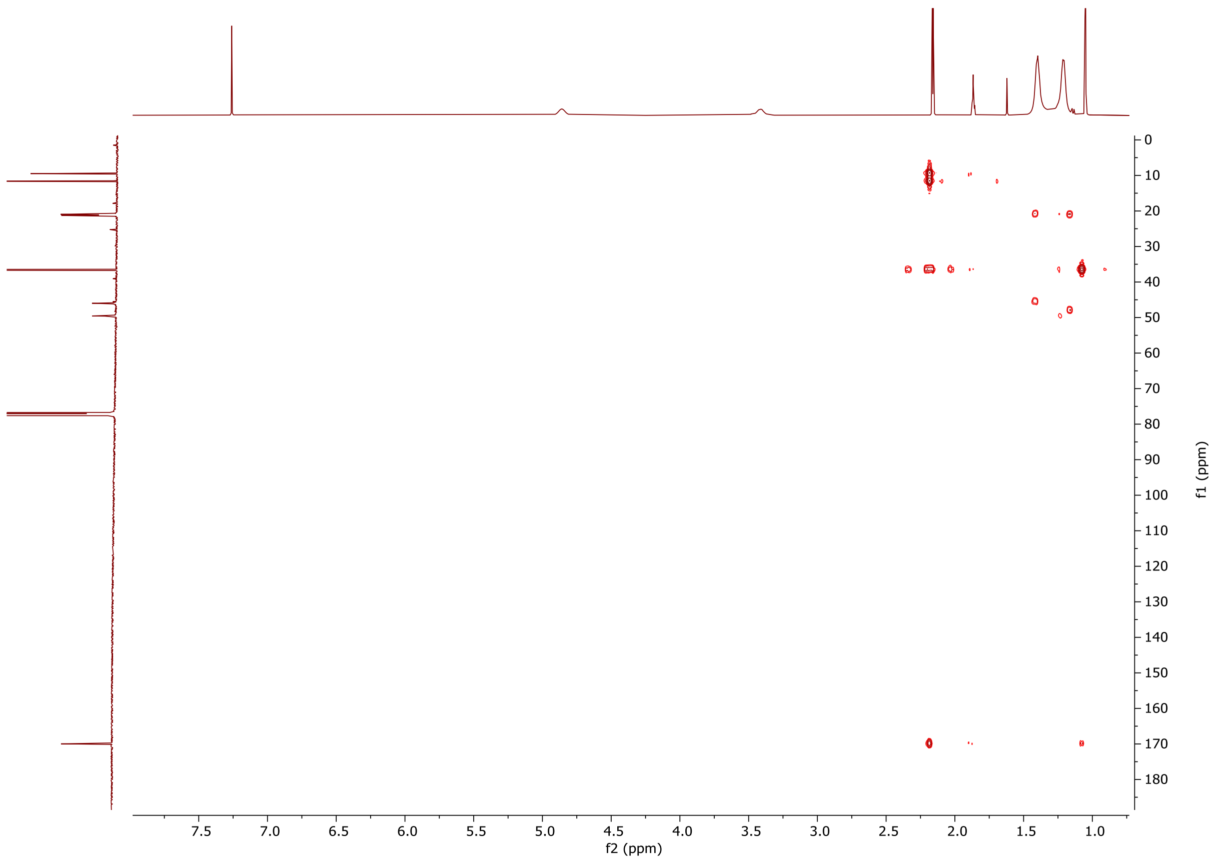

Tert-butylbicyclo[1.1.0]butane-1-carboxylate, 8d

^{1}H NMR (400 MHz, Chloroform-*d*)

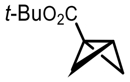

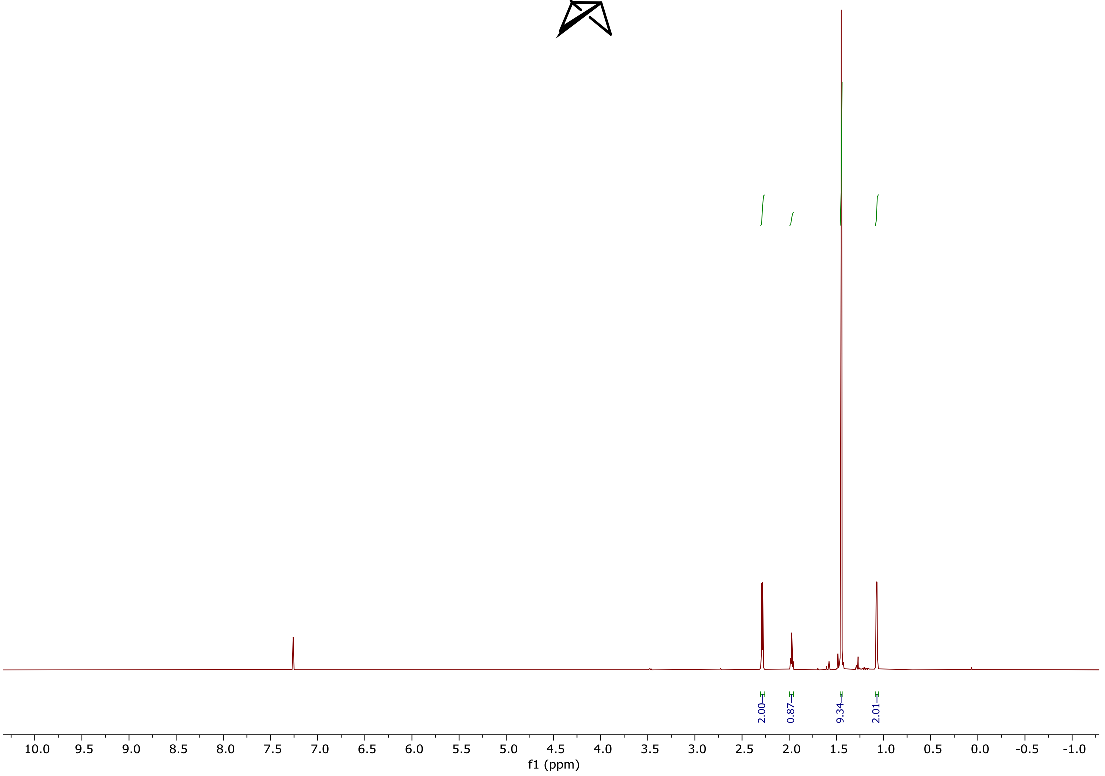

^{13}C NMR (101 MHz, Chloroform-*d*)

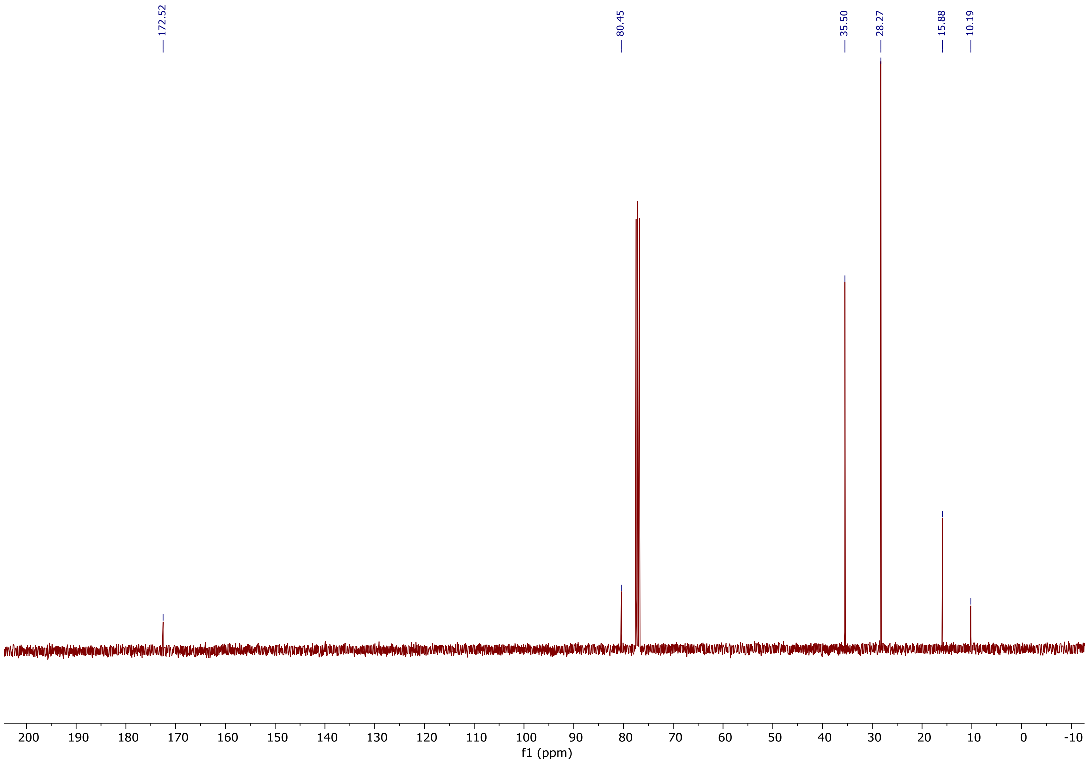

1-phenyl-3-(phenylsulfonyl)bicyclo[1.1.0]butane, 11

^{1}H NMR (400 MHz, Chloroform-*d*)

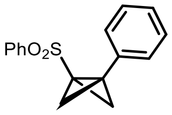

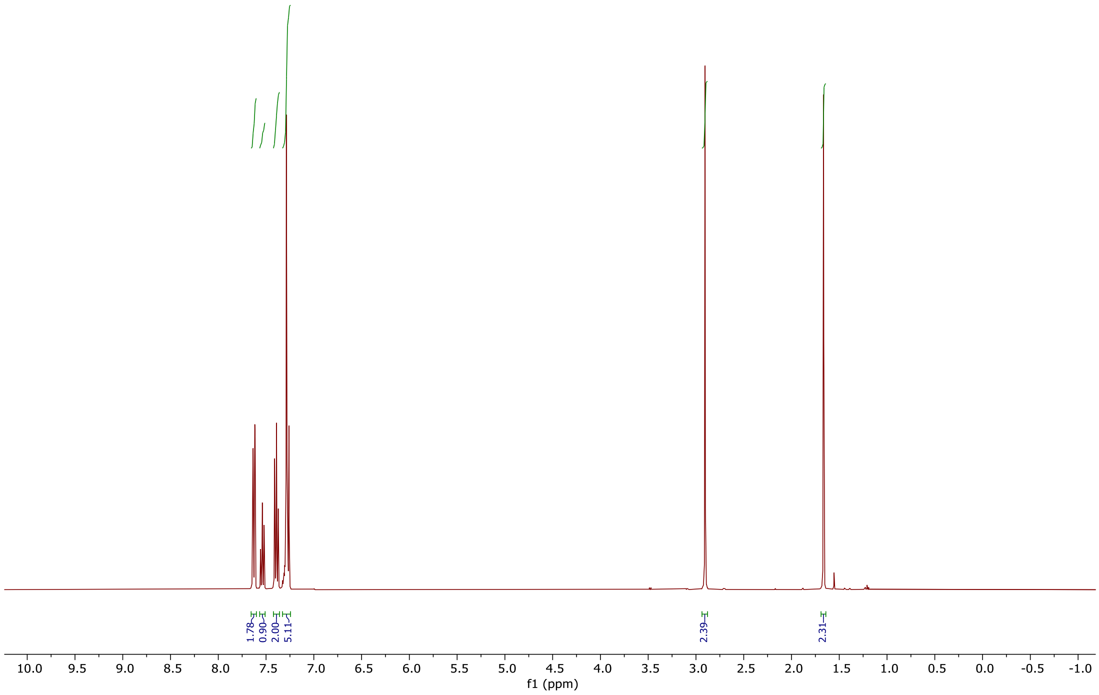

^{13}C NMR (101 MHz, Chloroform-*d*)

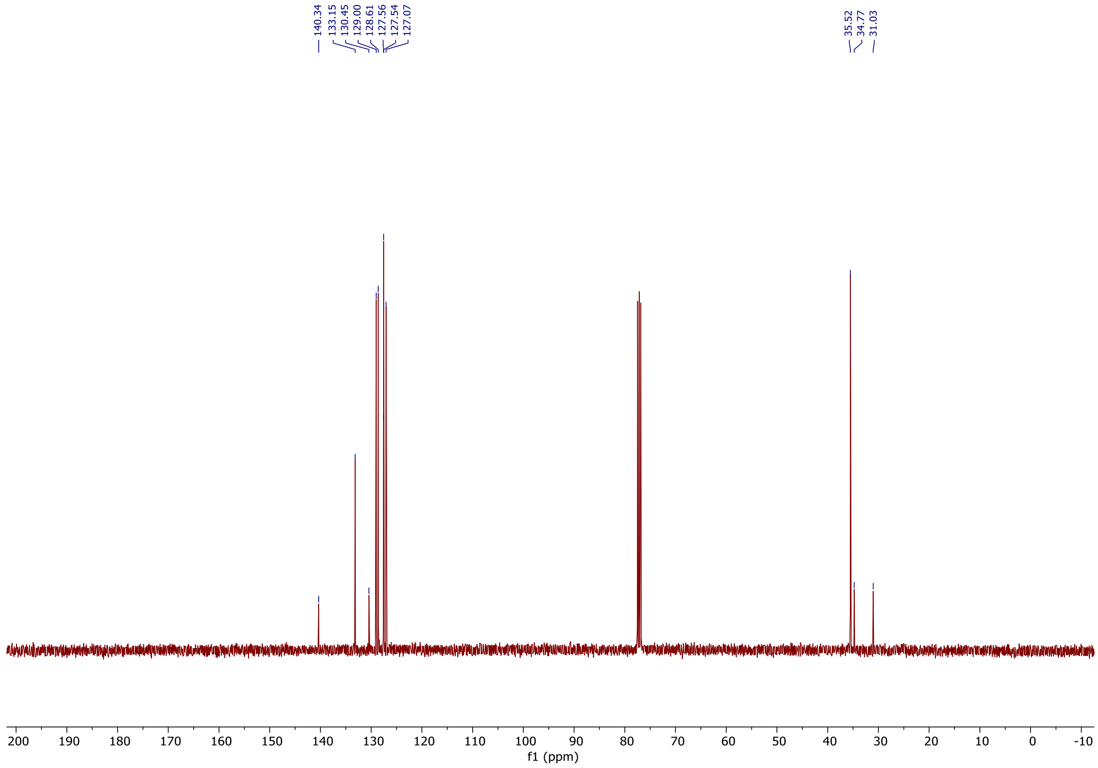

((3-phenylcyclobut-2-en-1-yl)sulfonyl)benzene, 12

^{1}H NMR (500 MHz, Chloroform-*d*)

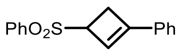

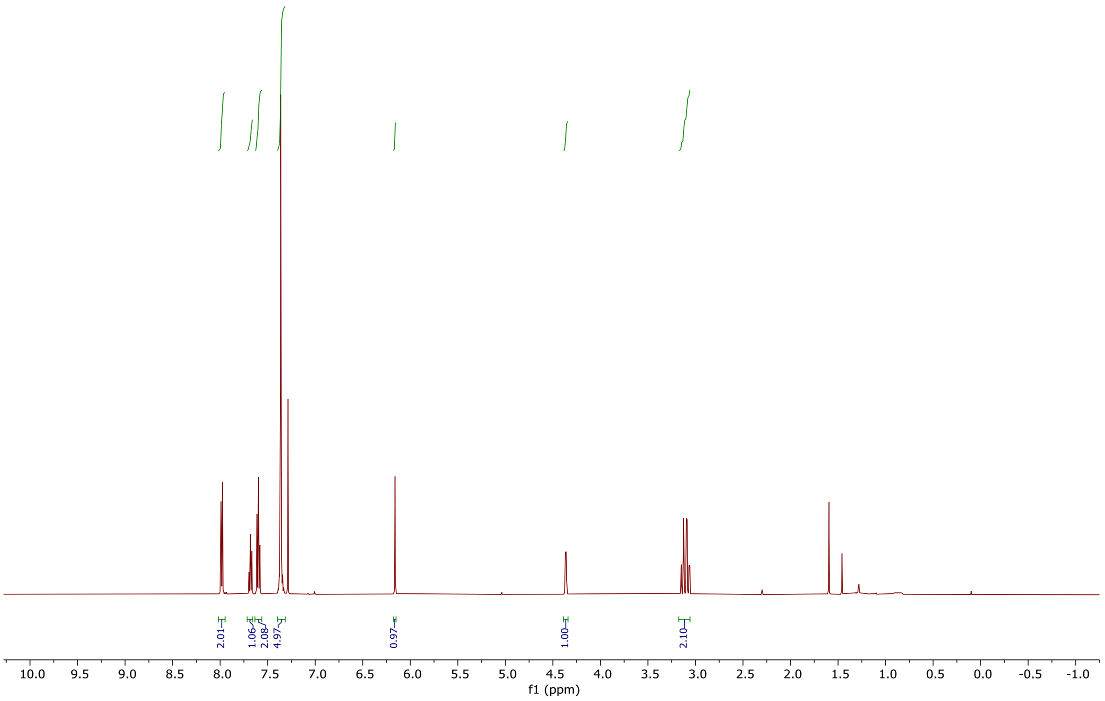

^{13}C NMR (126 MHz, Chloroform-*d*)

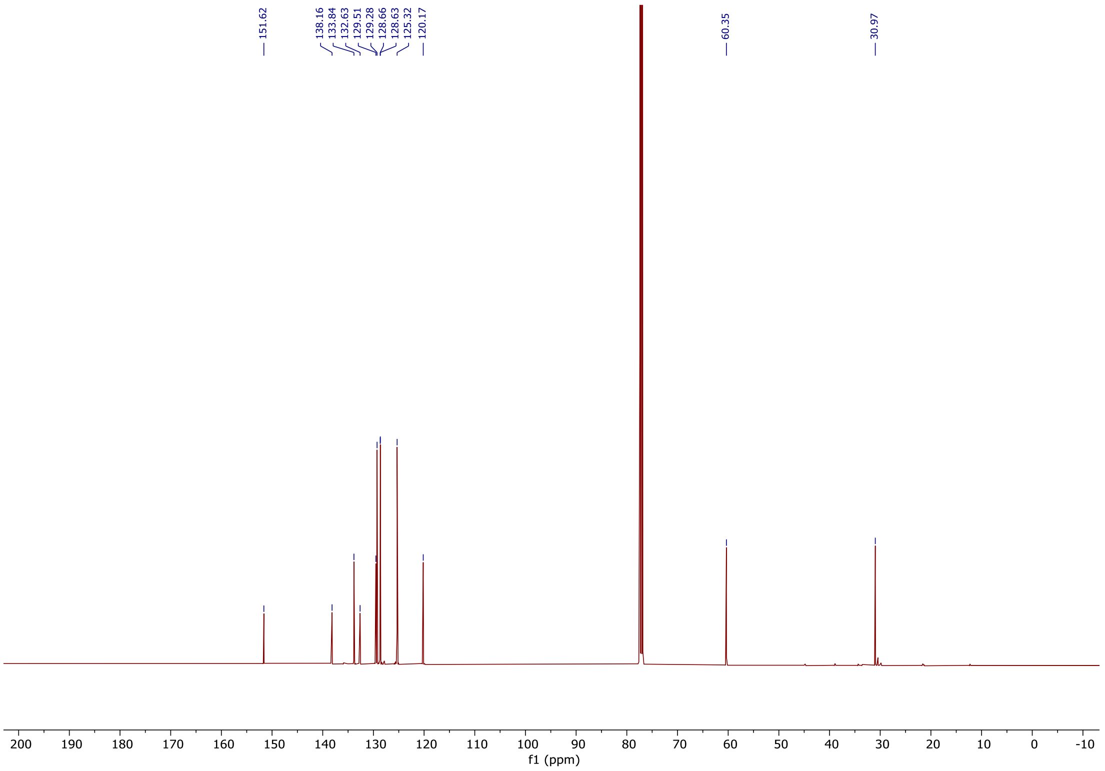

^{1}H COSY (500 MHz, Chloroform-*d*)

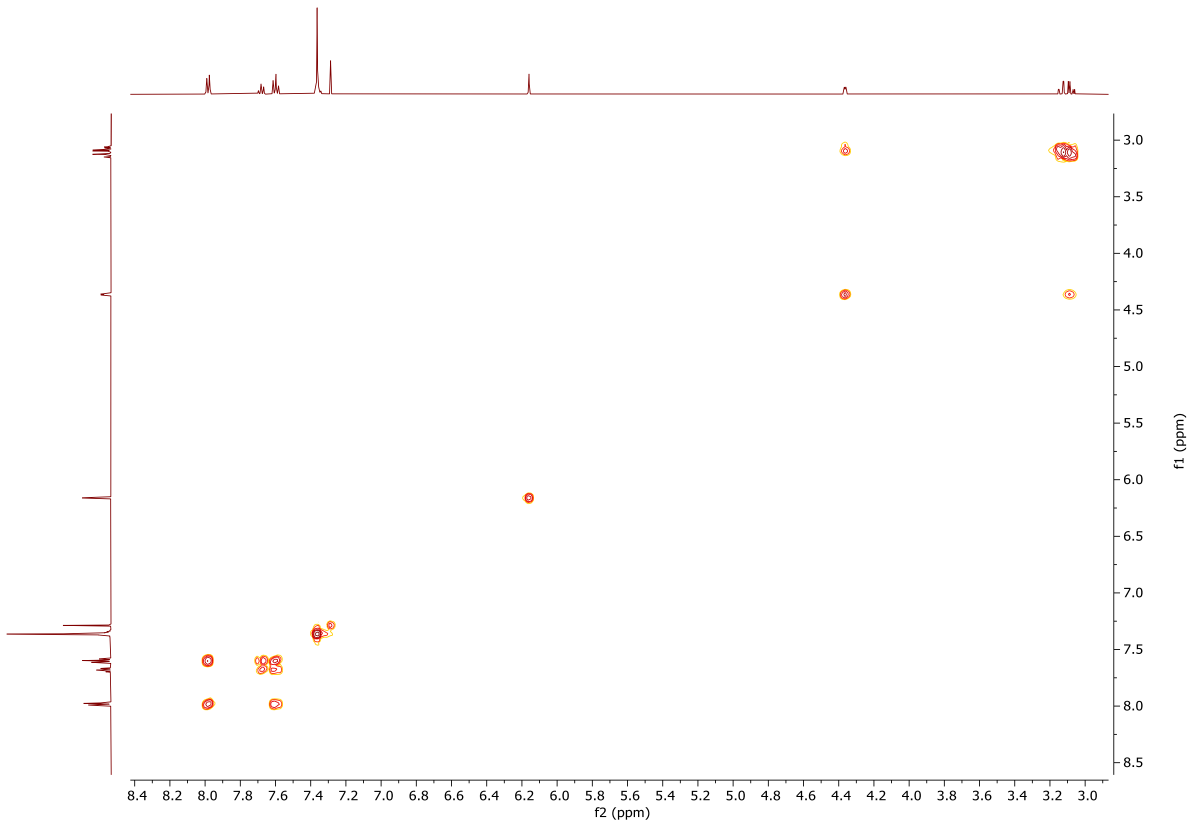

^{1}H/^{13}C HSQC (500/126 MHz, Chloroform-*d*)

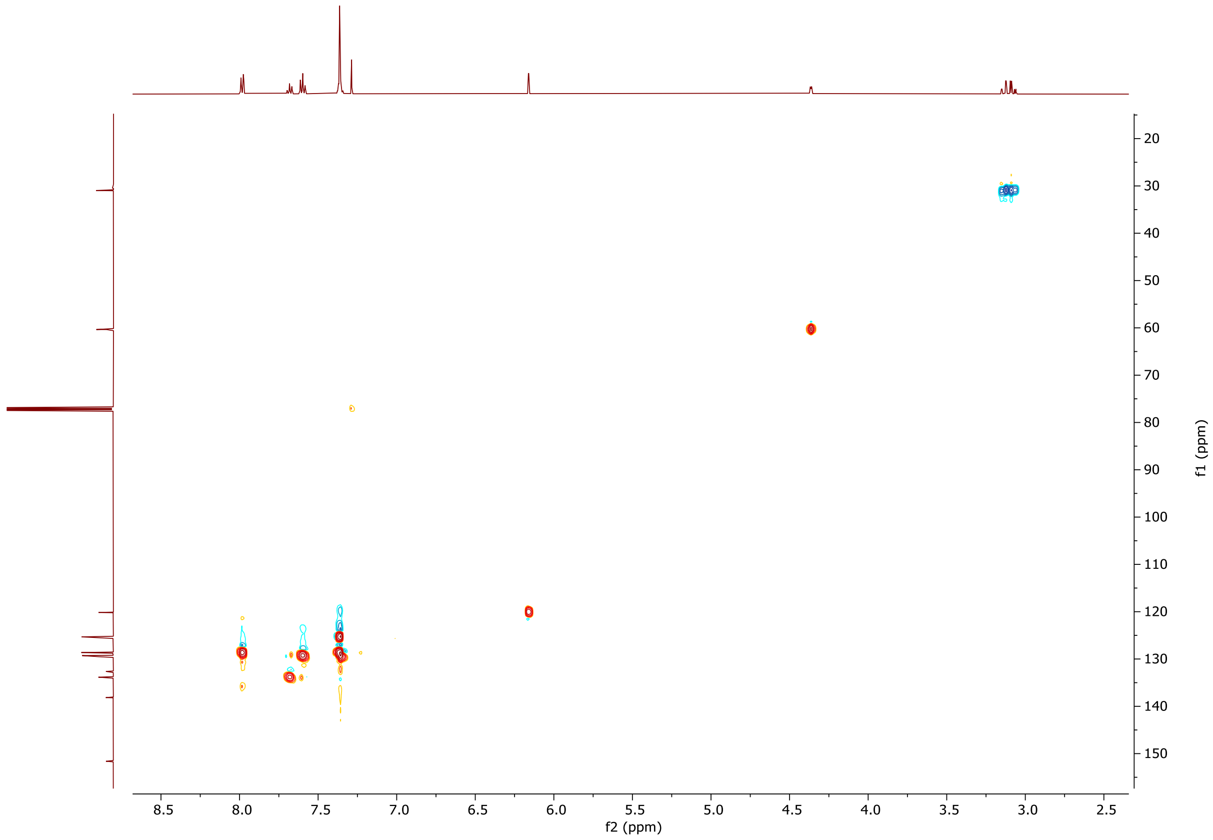

^{1}H/^{13}C HMBC (500/126 MHz, Chloroform-*d*)

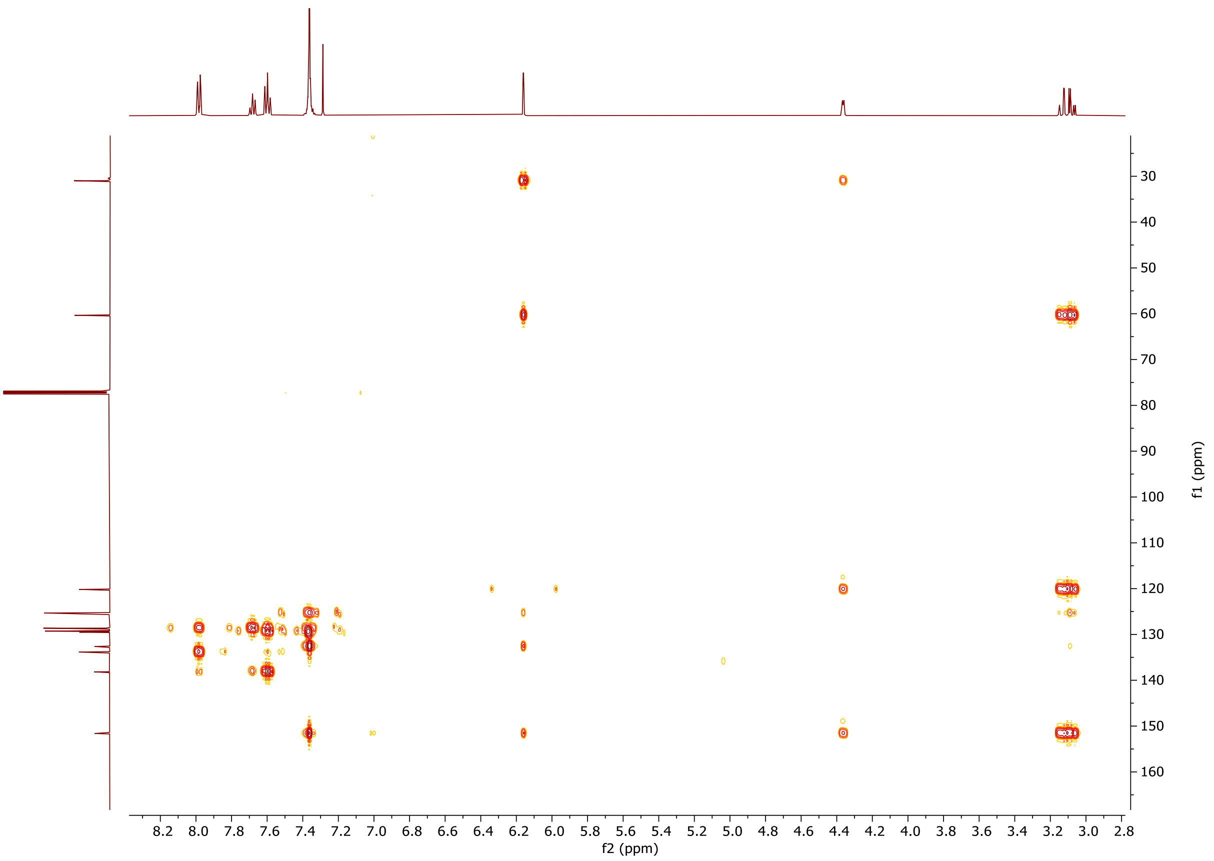

1-(4-nitrophenyl)-3-(phenylsulfonyl)bicyclo[1.1.0]butane, 13

^{1}H NMR (500 MHz, Chloroform-*d*)

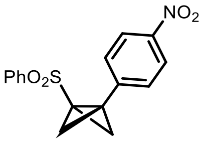

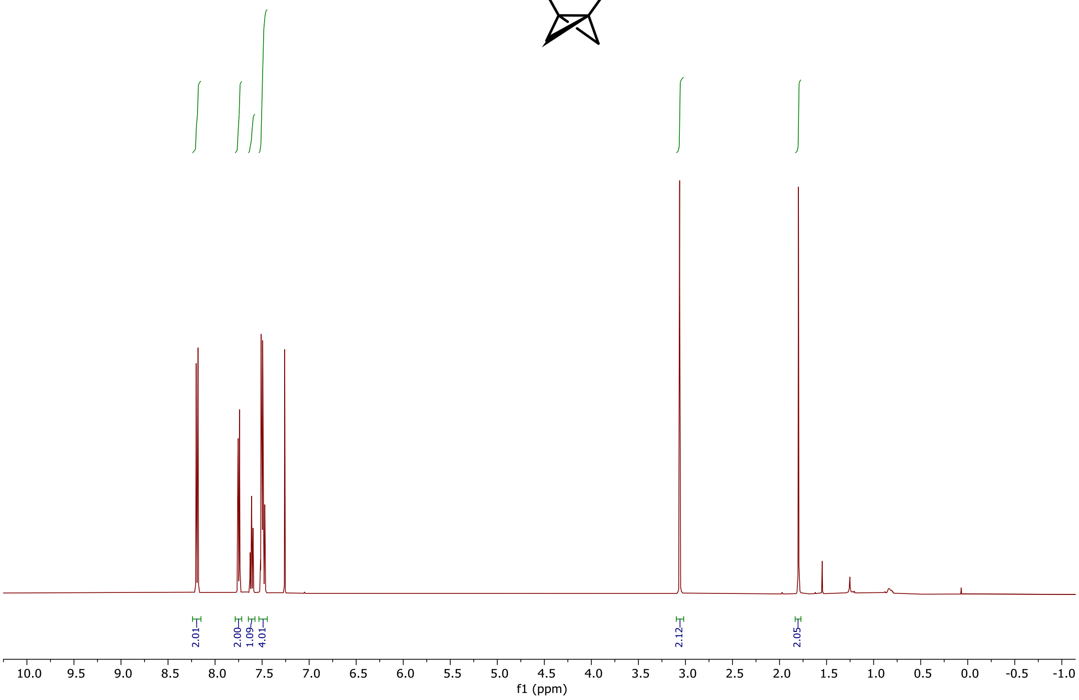

^{13}C NMR (126 MHz, Chloroform-*d*)

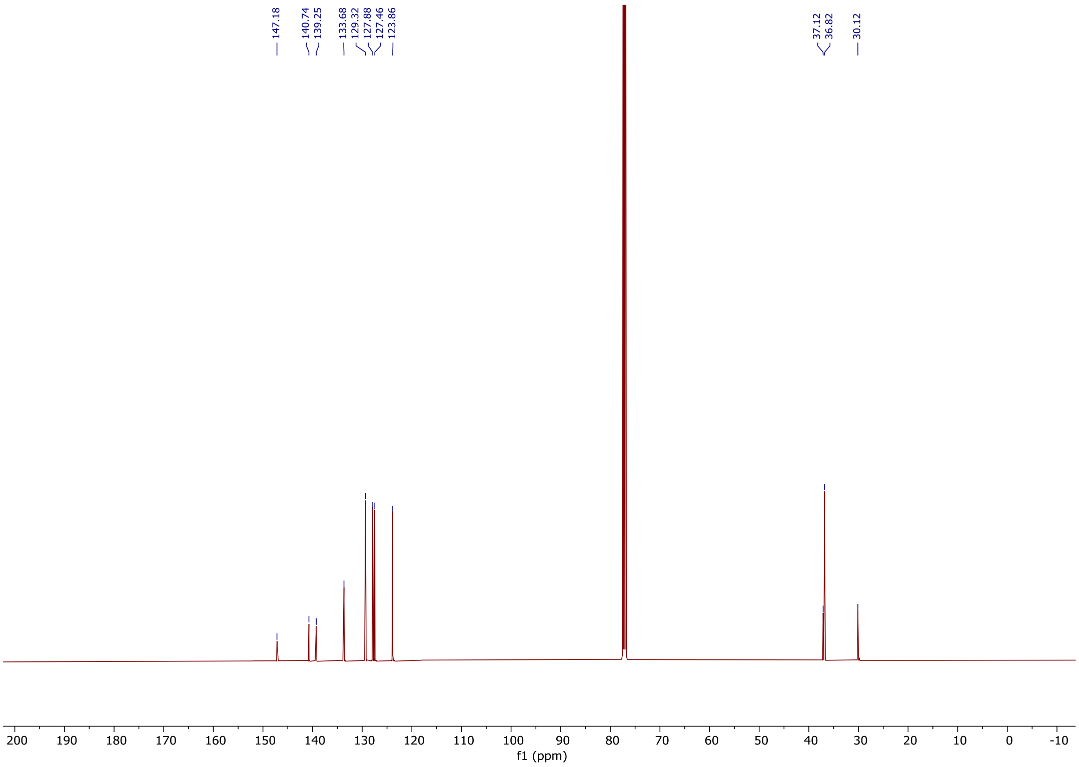

^{1}H COSY (500 MHz, Chloroform-*d*)

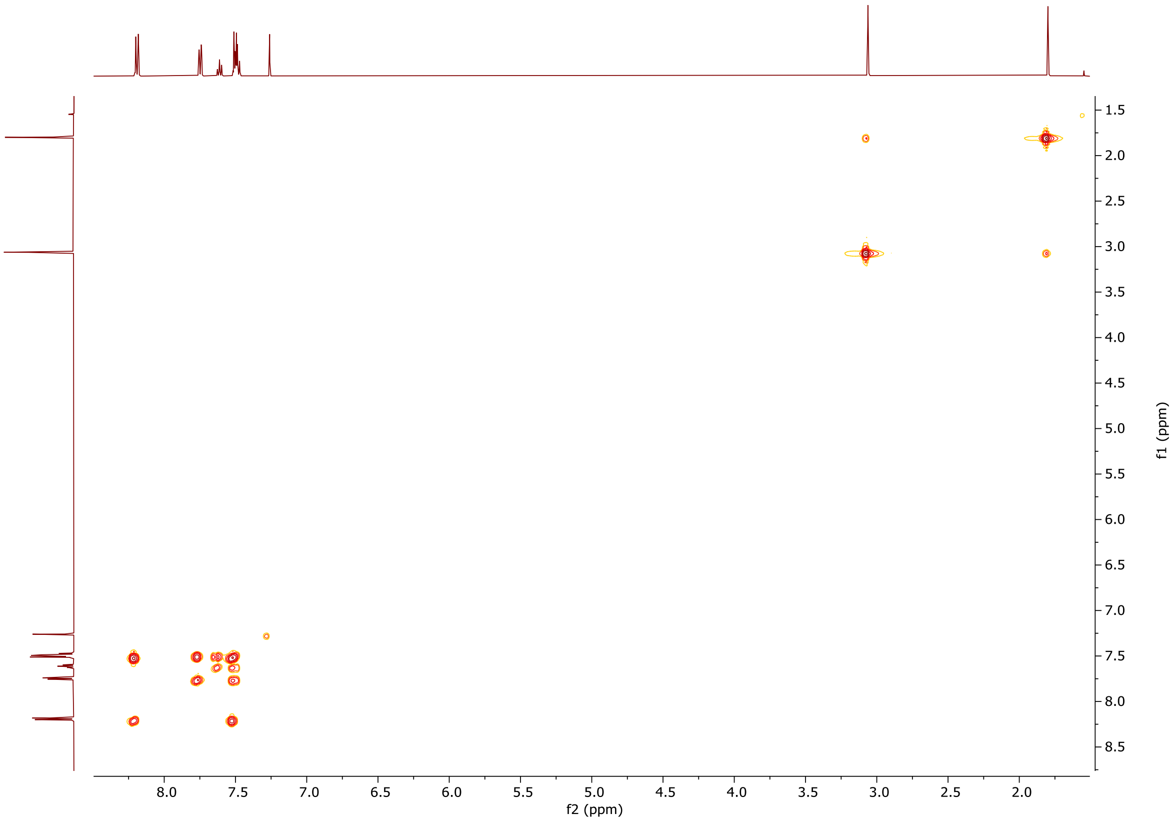

^{1}H/^{13}C HSQC (500/126 MHz, Chloroform-*d*)

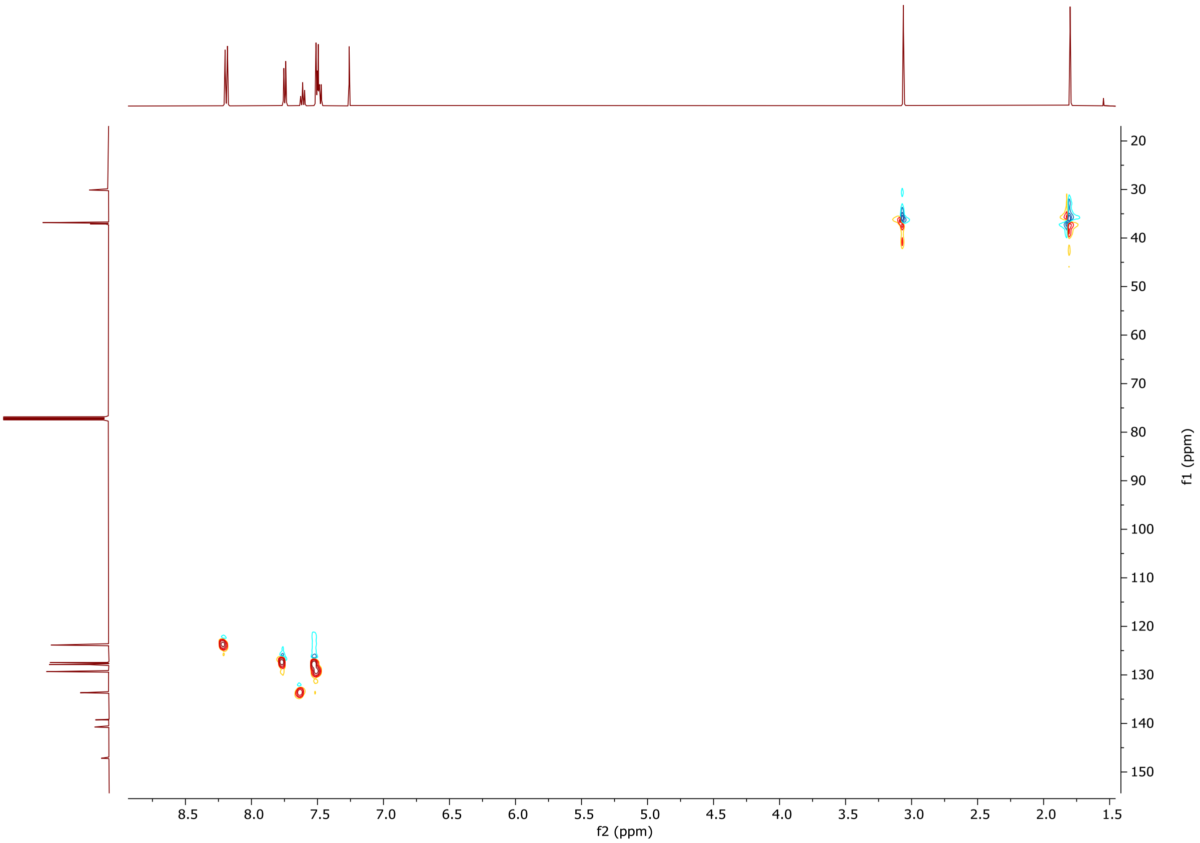

$^1H/^{13}C$ HMBC (500/126 MHz, Chloroform-*d*)

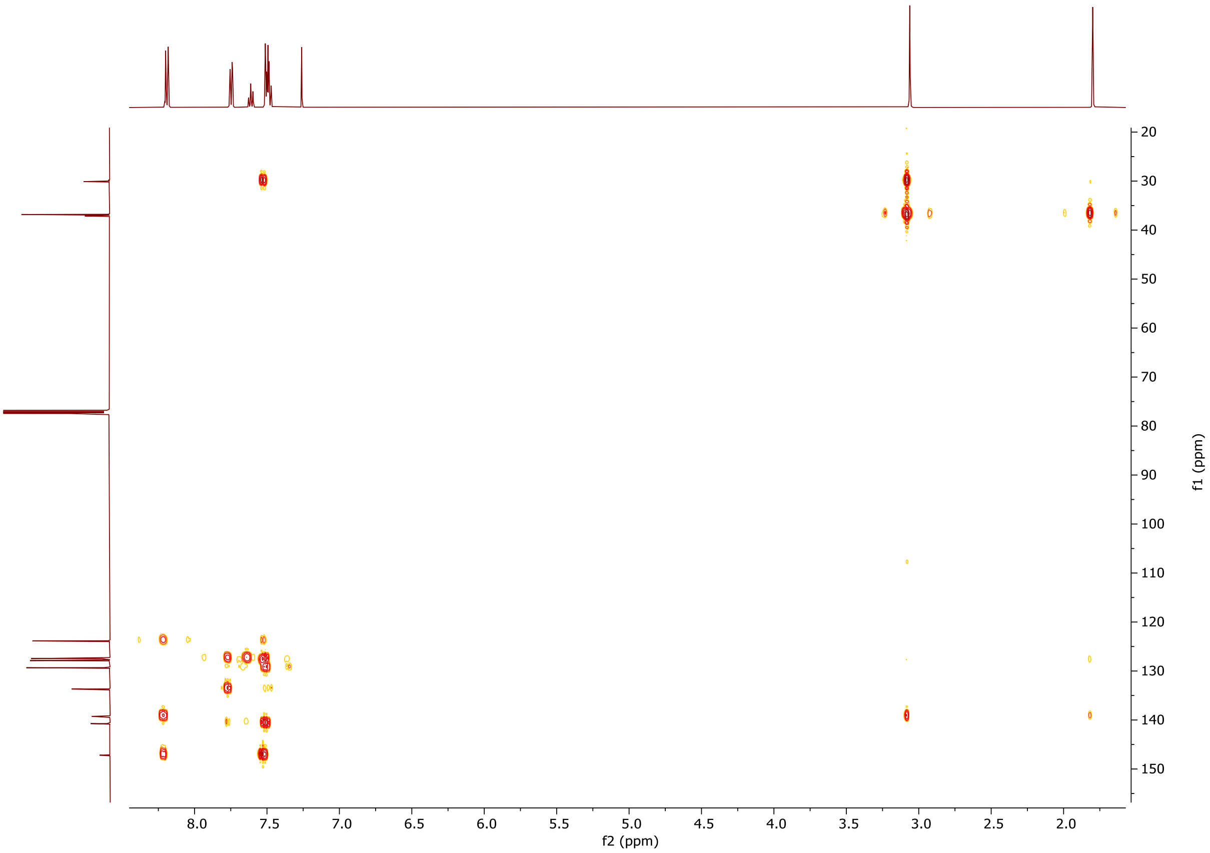

Methyl 4-(3-(phenylsulfonyl)bicyclo[1.1.0]butan-1-yl)benzoate, 14

^{1}H NMR (400 MHz, Chloroform-*d*)

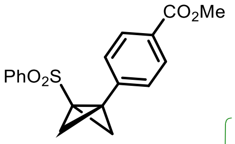

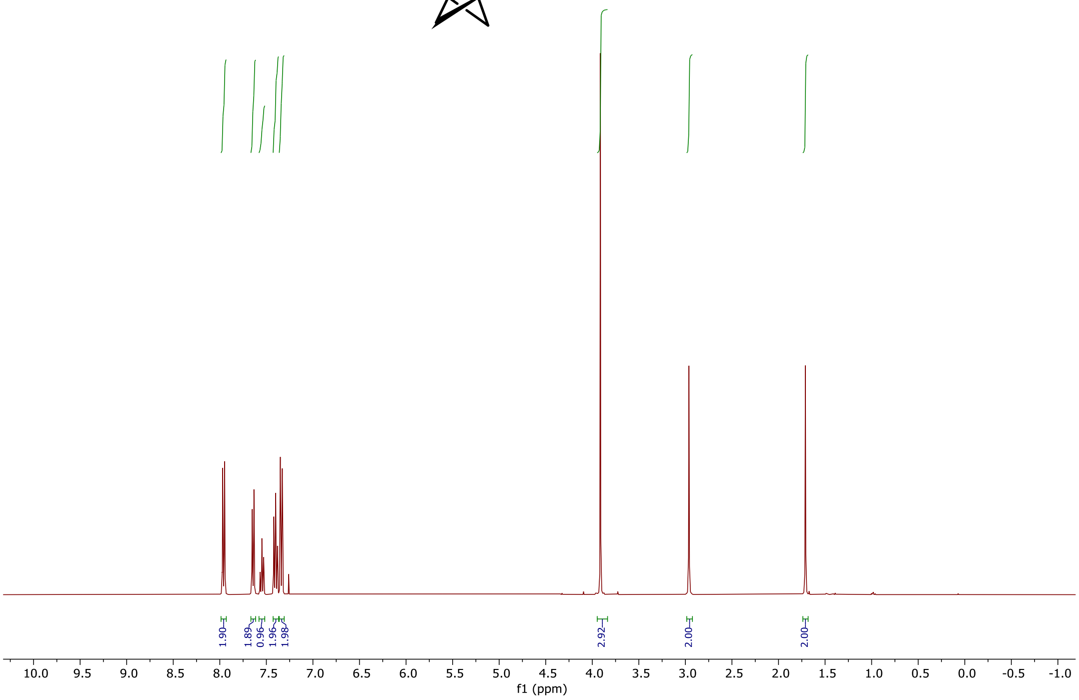

^{13}C NMR (101 MHz, Chloroform-*d*)

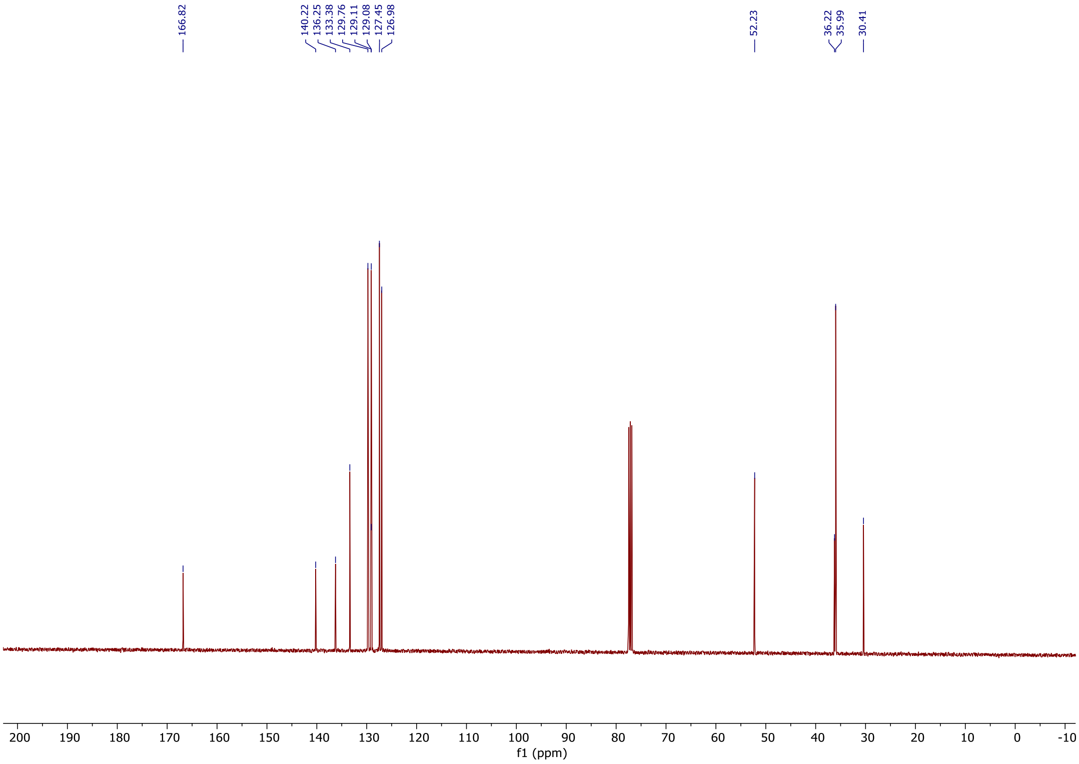

^{1}**H COSY** (400 MHz, Chloroform-*d*)

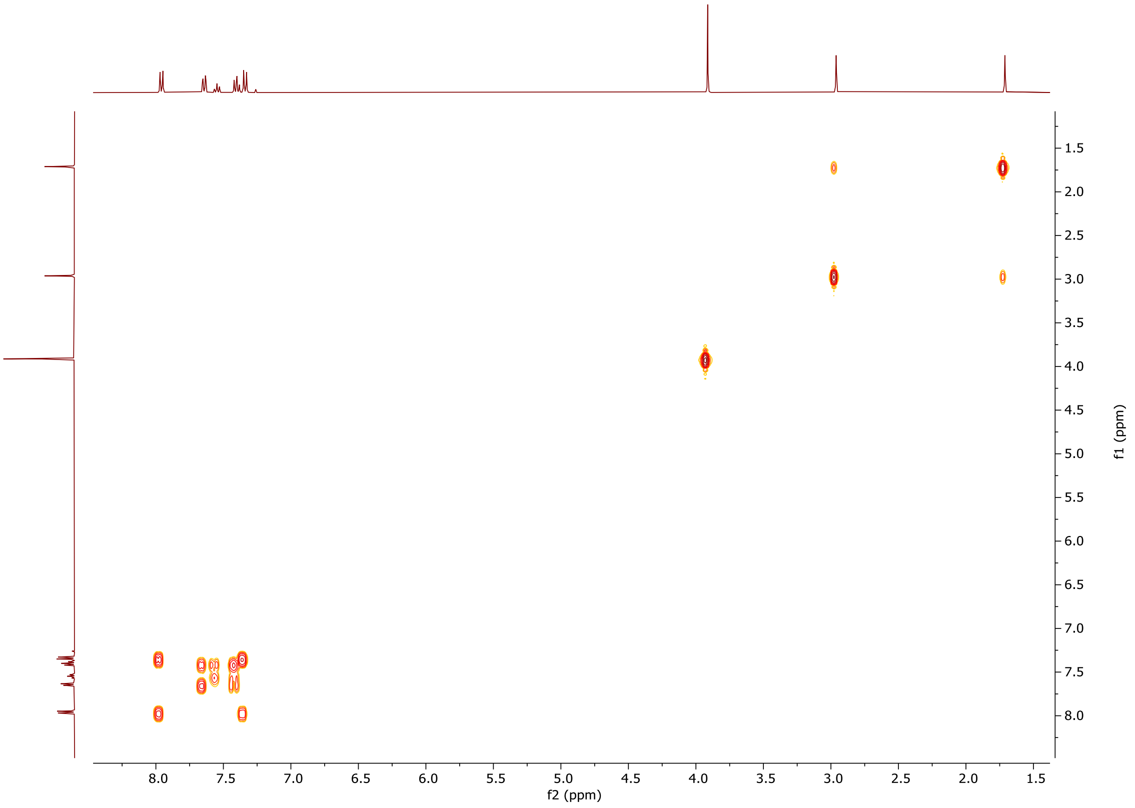

^{1}**H/**^{13}**C HSQC** (400/101 MHz, Chloroform-*d*)

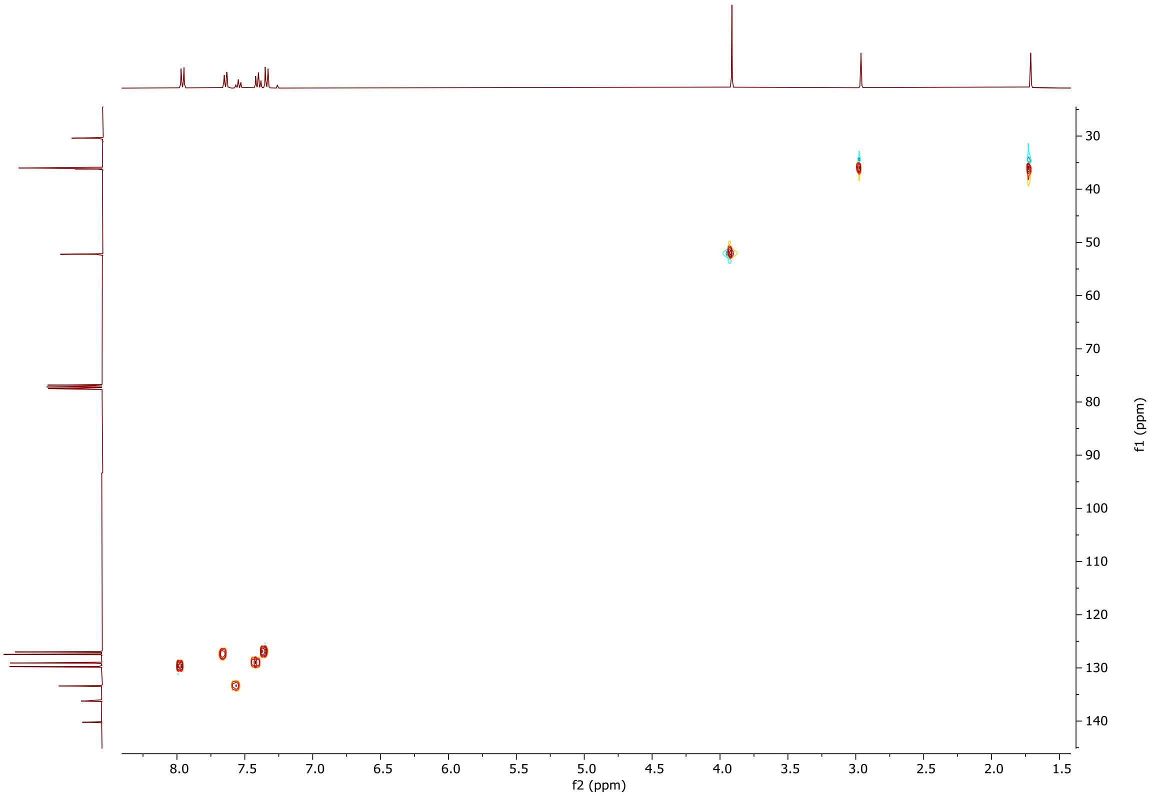

1-(4-(3-(phenylsulfonyl)bicyclo[1.1.0]butan-1-yl)phenyl)ethan-1-one, 15

^{1}H NMR (500 MHz, Chloroform-*d*)

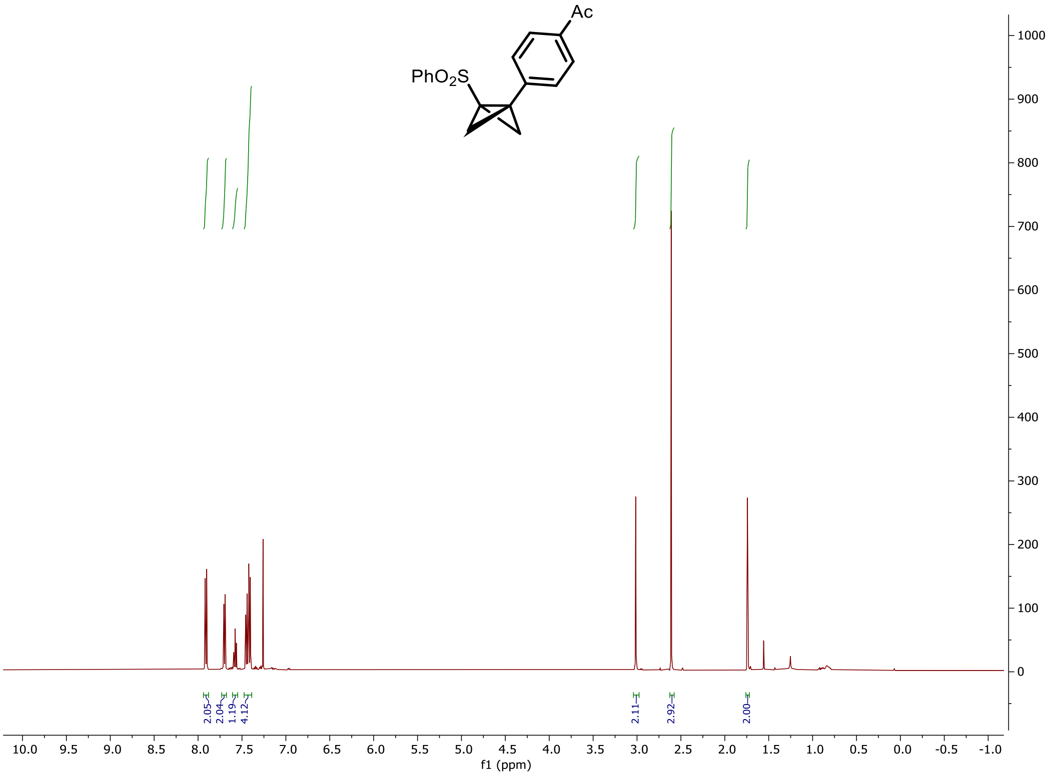

^{13}C NMR (126 MHz, Chloroform-*d*)

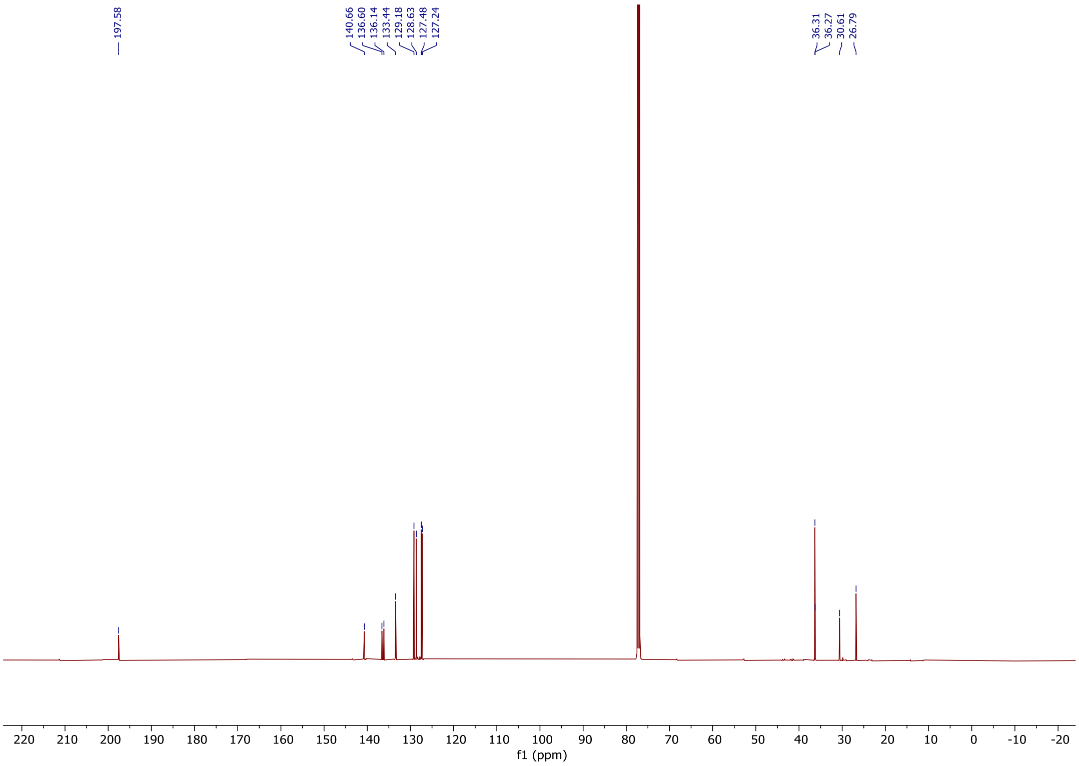

¹H COSY (500 MHz, Chloroform-*d*)

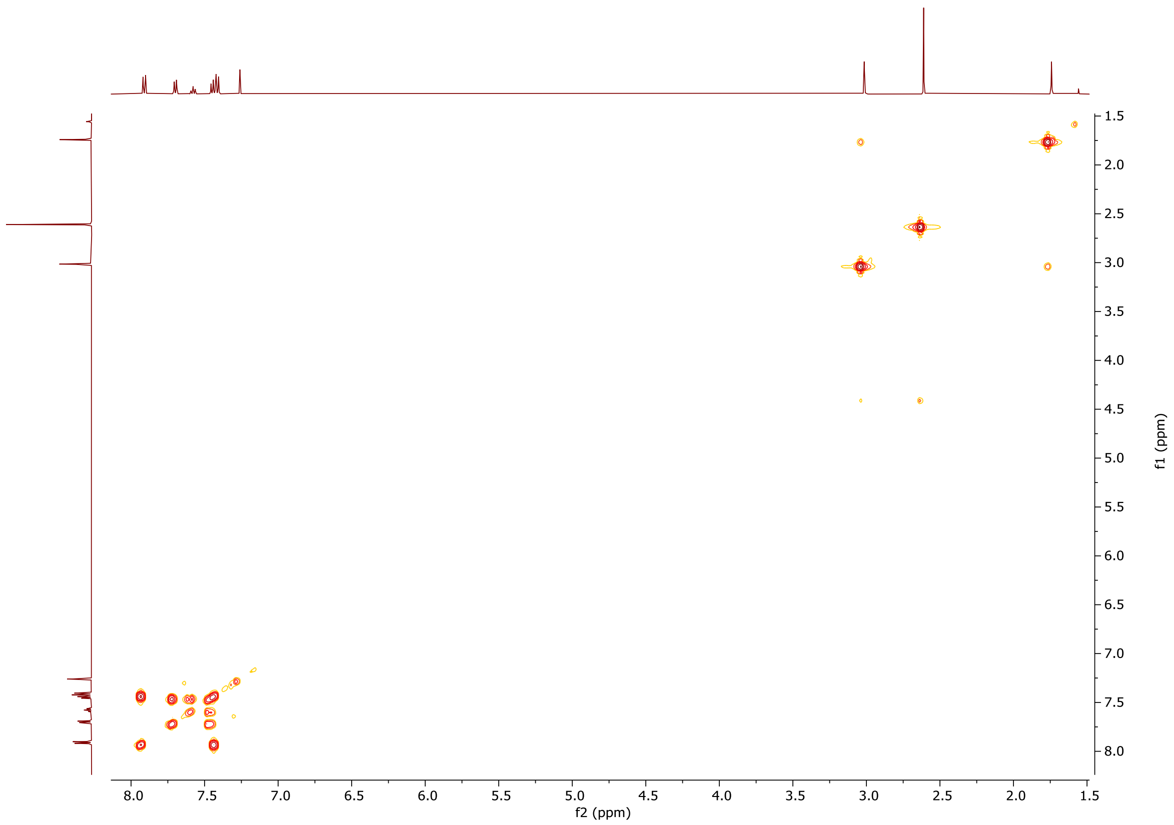

¹H/¹³C HSQC (500/126 MHz, Chloroform-*d*)

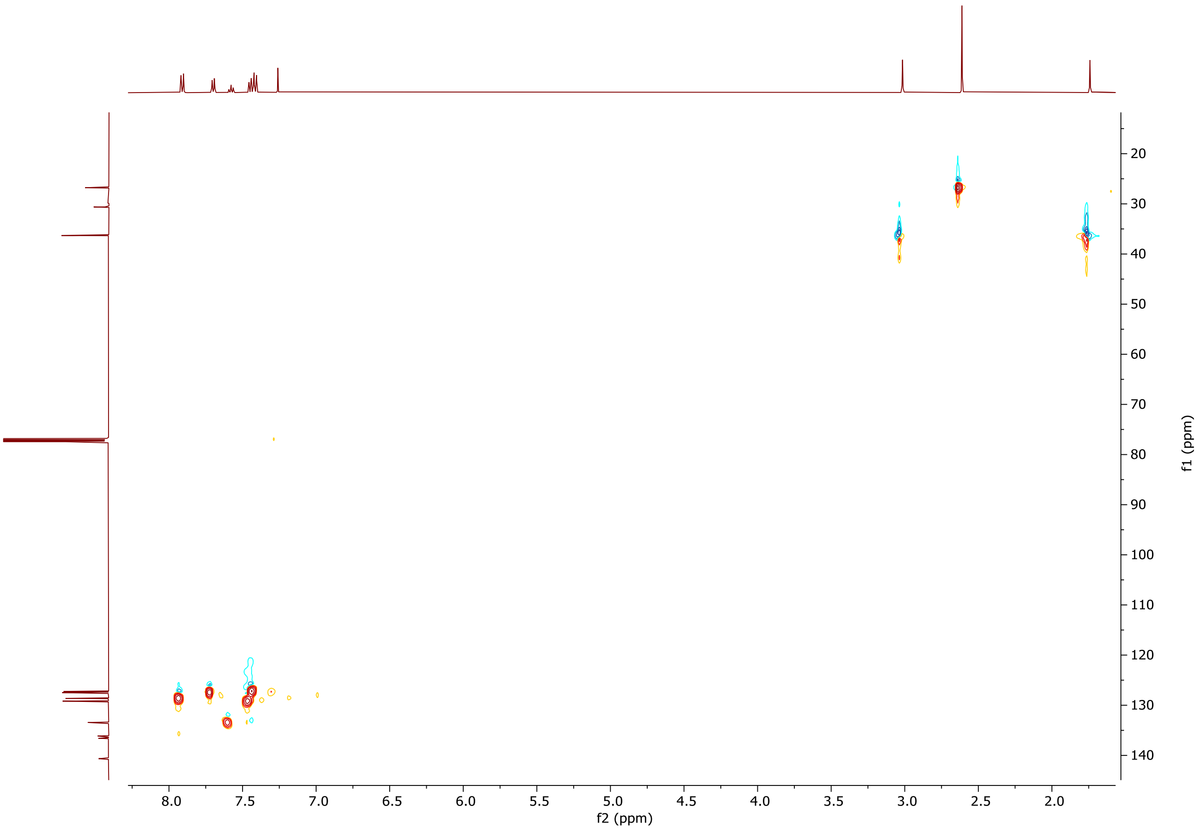

$^{1}H/^{13}C$ HMBC (500/126 MHz, Chloroform-*d*)

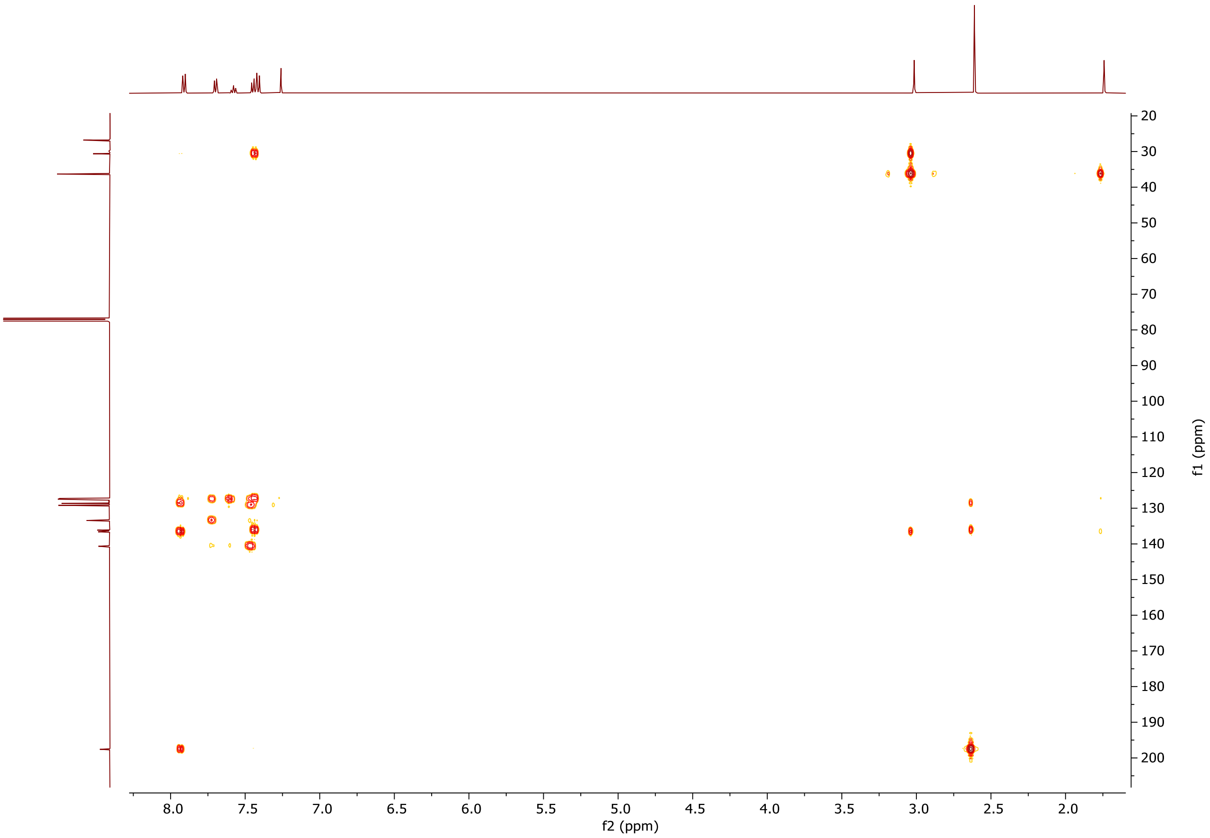

1-(4-fluorophenyl)-3-(phenylsulfonyl)bicyclo[1.1.0]butane, 16

^{1}H NMR (400 MHz, Chloroform-*d*)

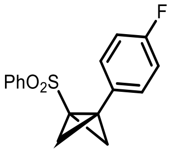

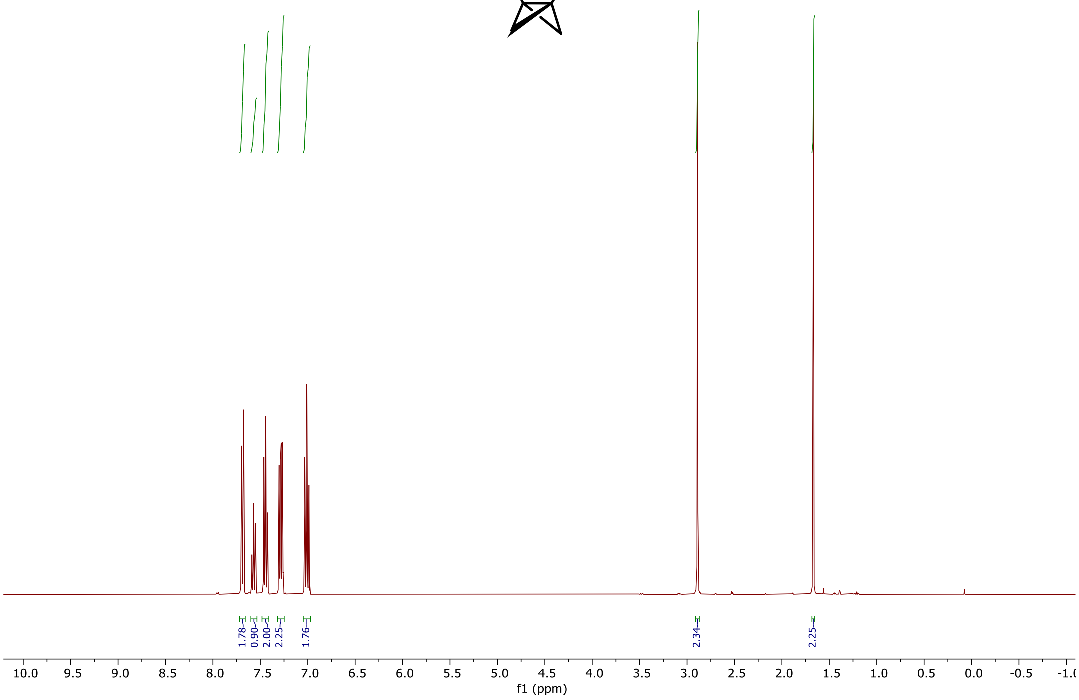

^{13}C NMR (101 MHz, Chloroform-*d*)

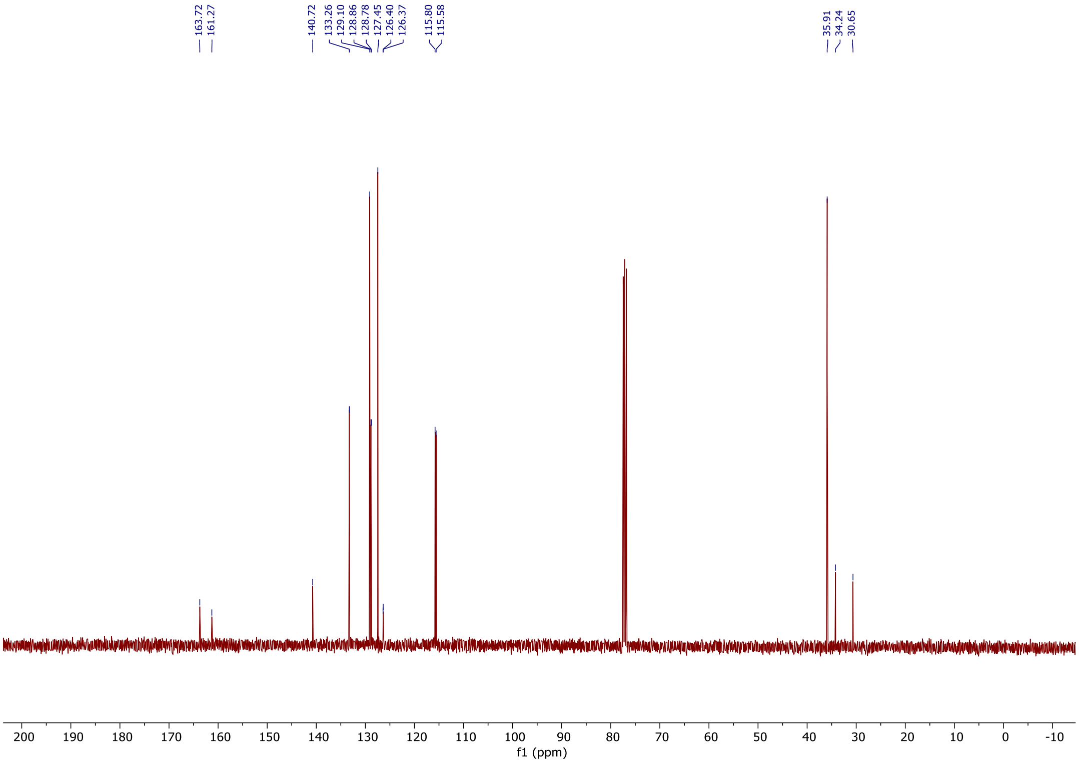

^{1}H COSY (400 MHz, Chloroform-*d*)

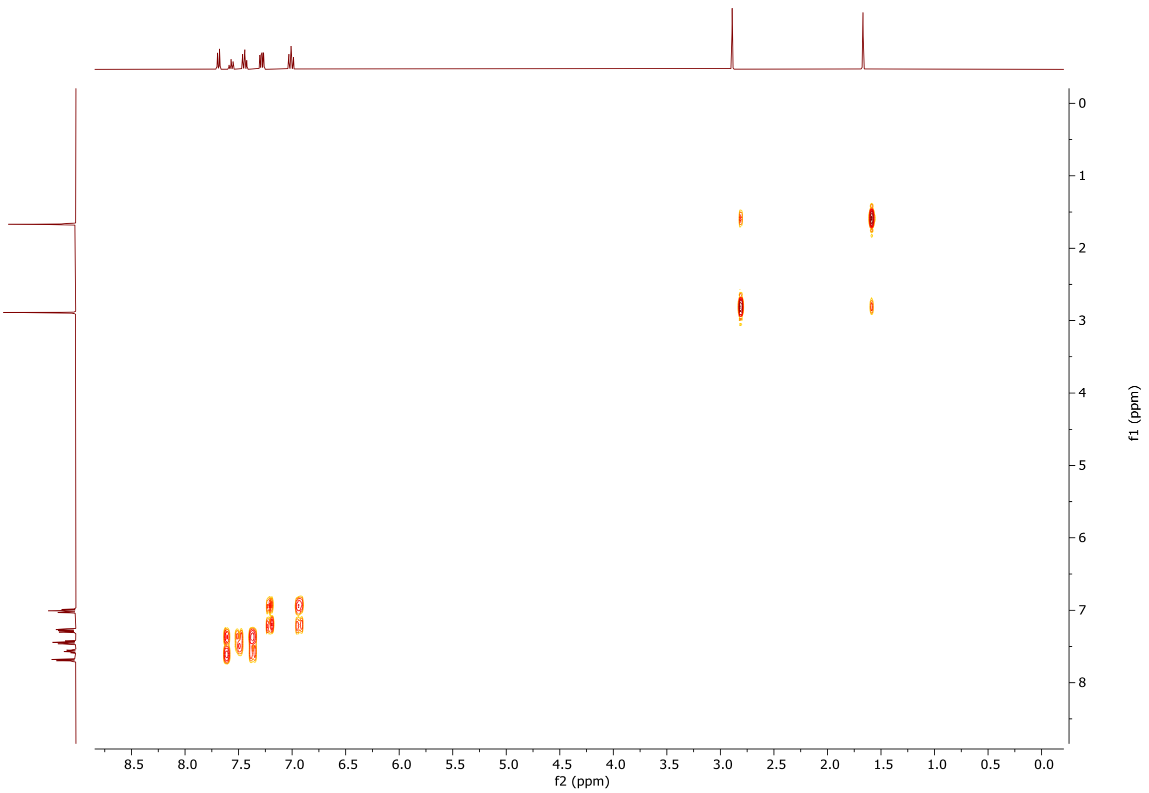

^{1}H/^{13}C HSQC (400/101 MHz, Chloroform-*d*)

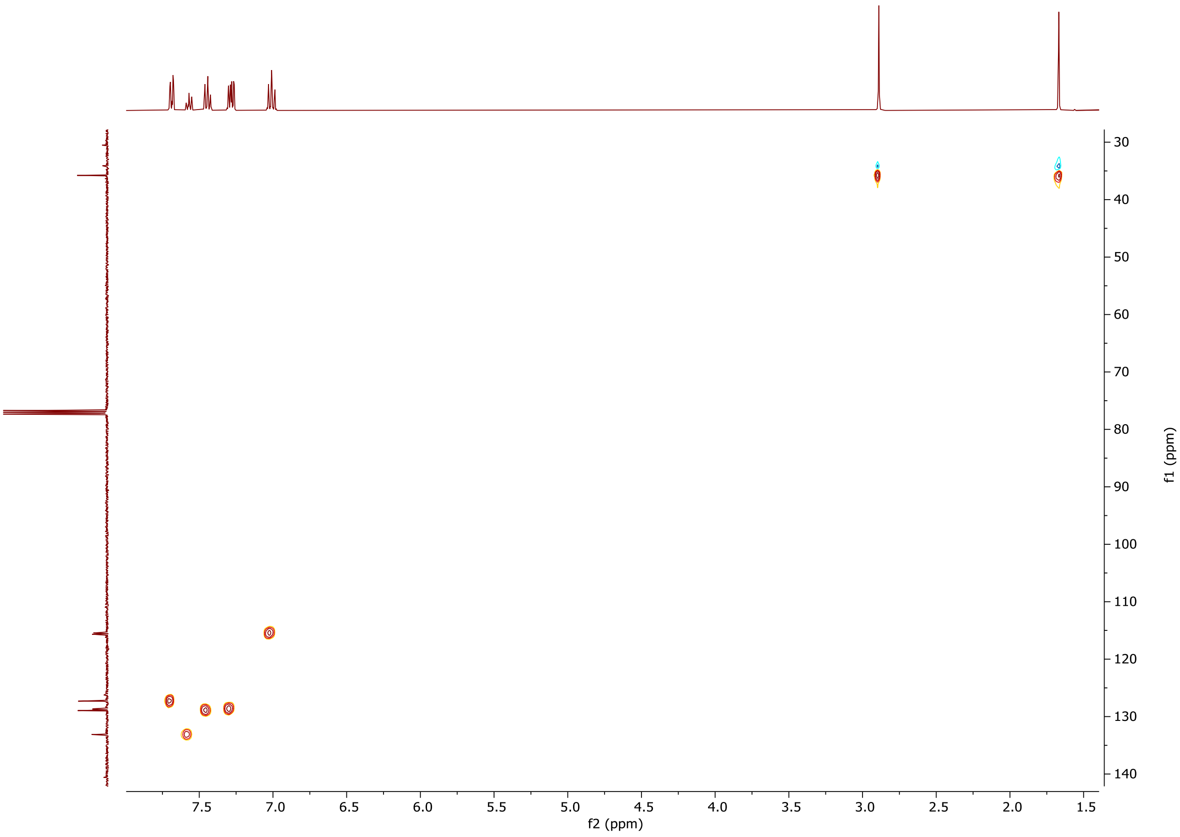

^{19}F NMR (377 MHz, Chloroform-*d*)

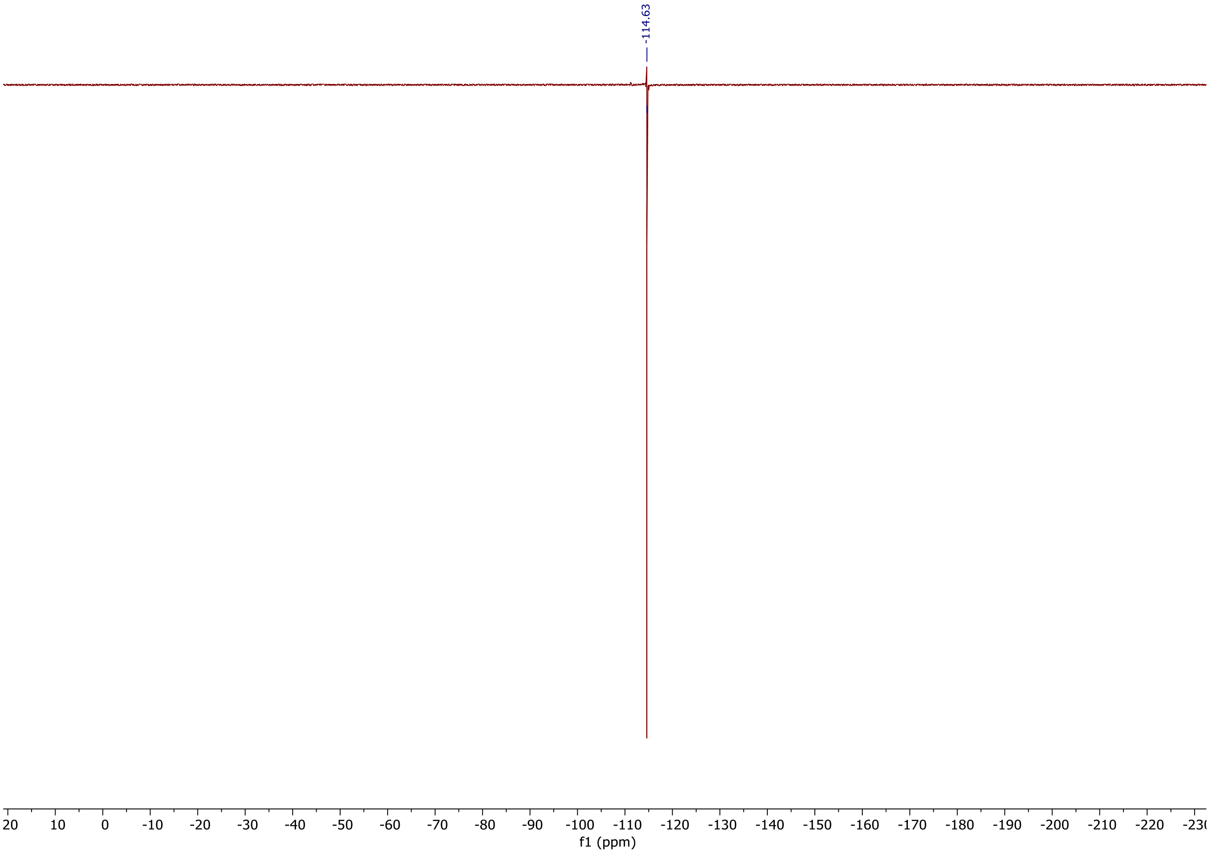

1-(4-chlorophenyl)-3-(phenylsulfonyl)bicyclo[1.1.0]butane, 17

^{1}H NMR (500 MHz, Chloroform-*d*)

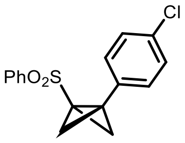

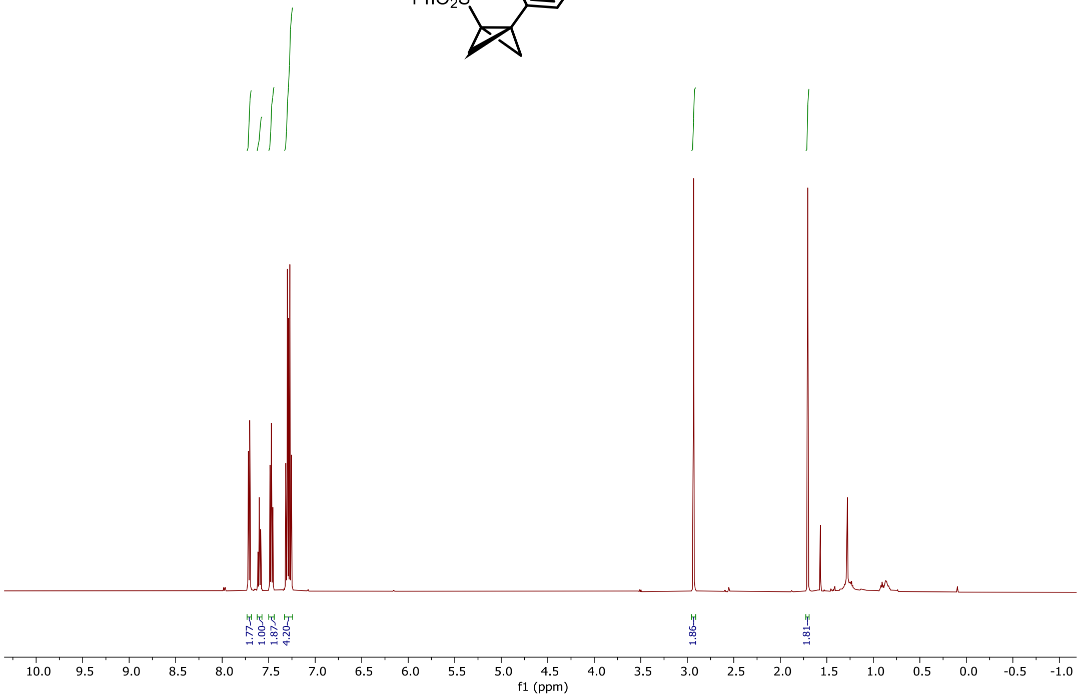

^{13}C NMR (126 MHz, Chloroform-*d*)

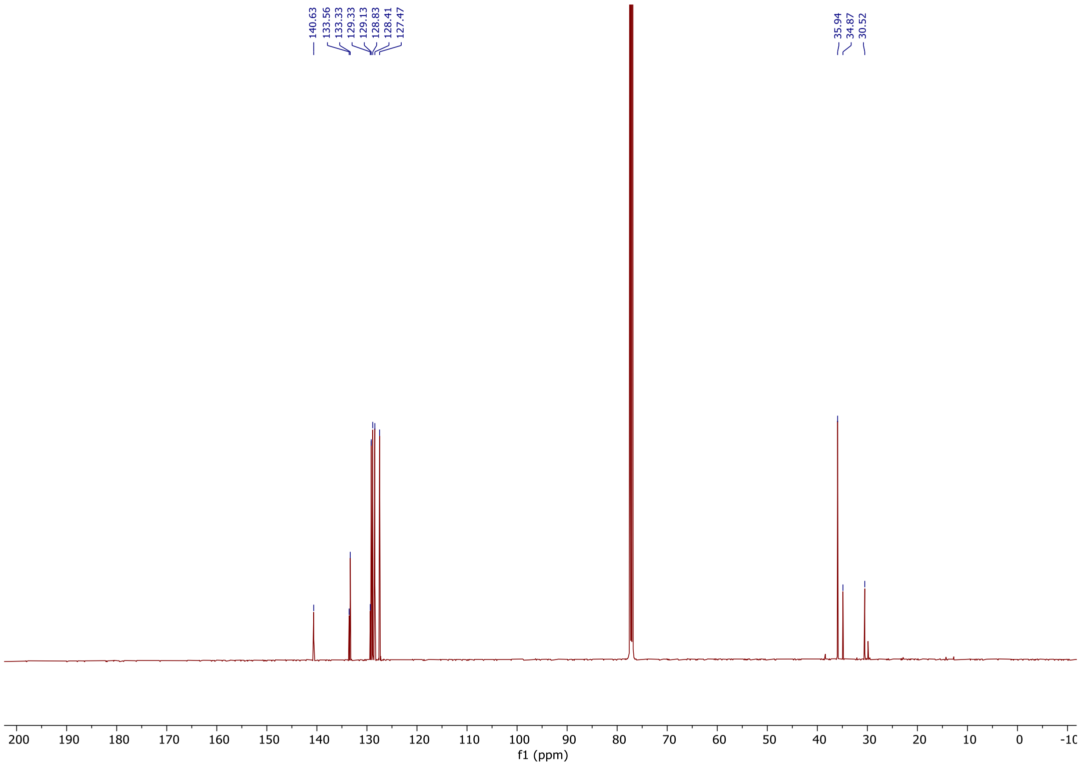

^{1}H COSY (500 MHz, Chloroform-*d*)

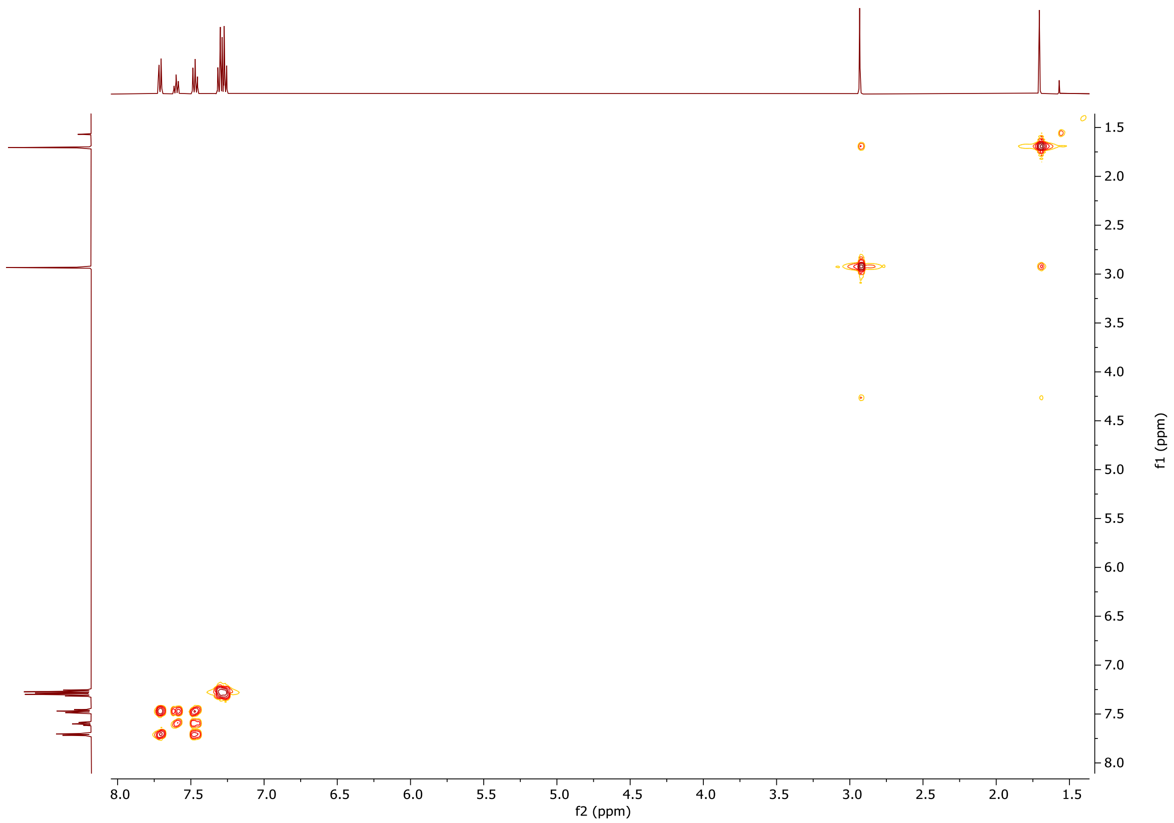

^{1}H/^{13}C HSQC (500/126 MHz, Chloroform-*d*)

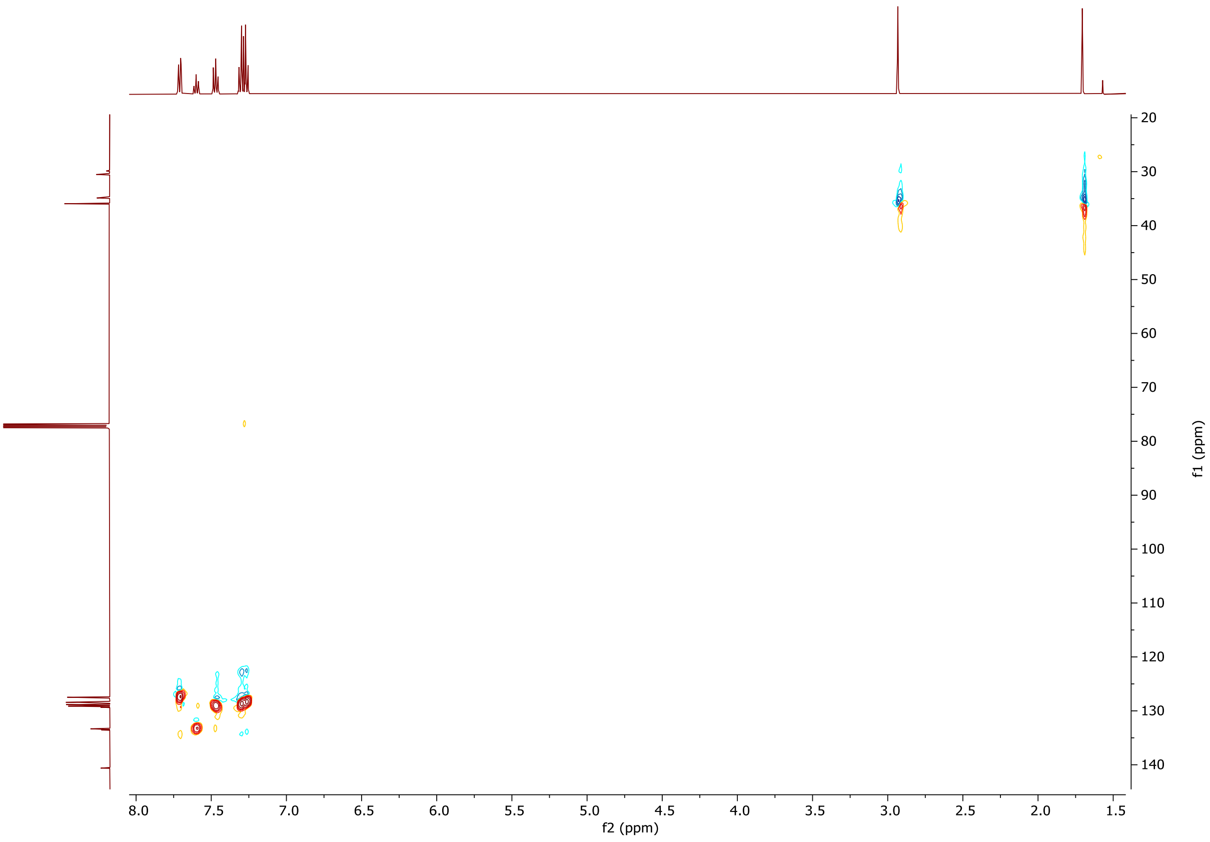

$^1H/^{13}C$ HMBC (500/126 MHz, Chloroform-*d*)

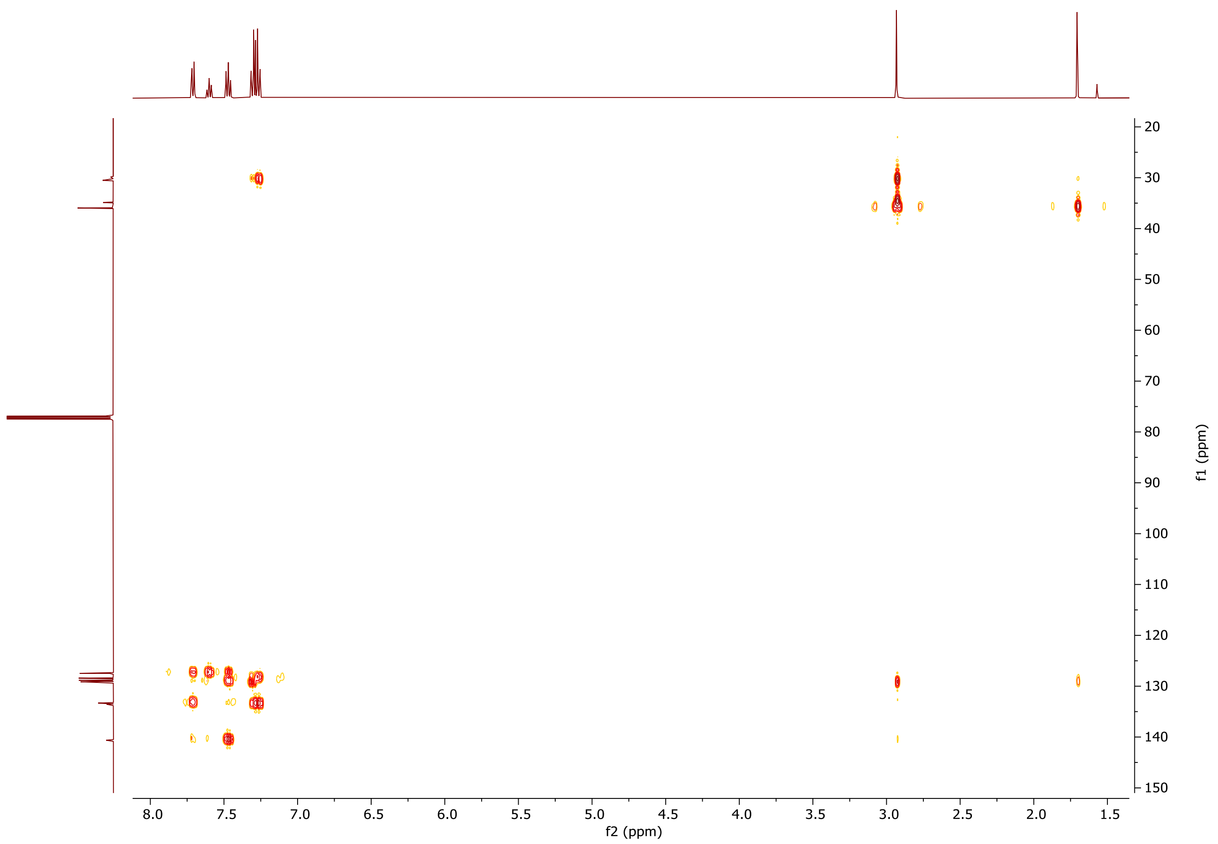

1-(phenylsulfonyl)-3-(*p*-tolyl)bicyclo[1.1.0]butane, 18

^{1}H NMR (400 MHz, Chloroform-*d*)

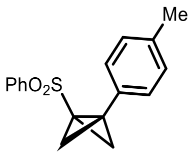

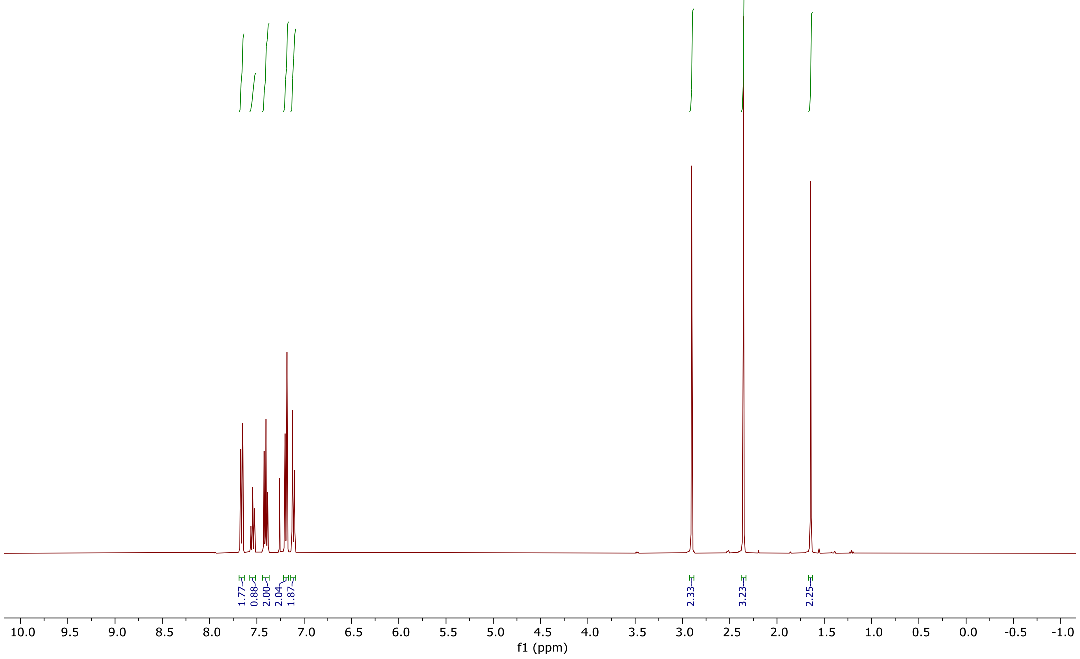

^{13}C NMR (101 MHz, Chloroform-*d*)

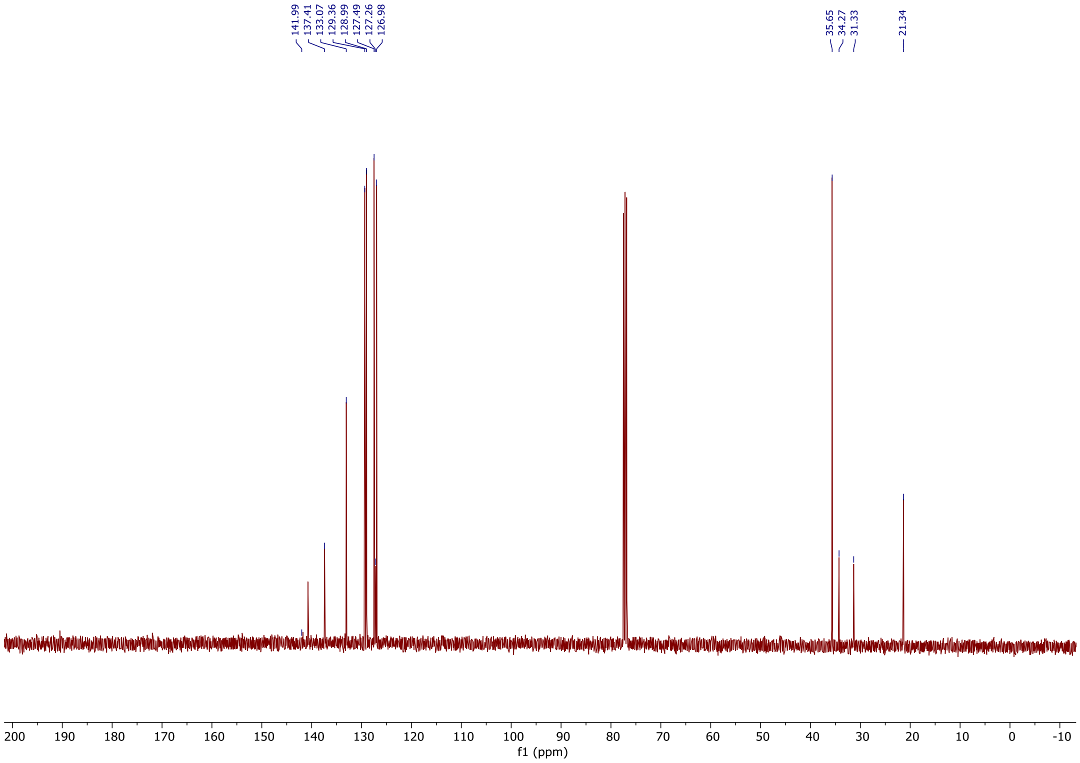

^{1}H COSY (400 MHz, Chloroform-*d*)

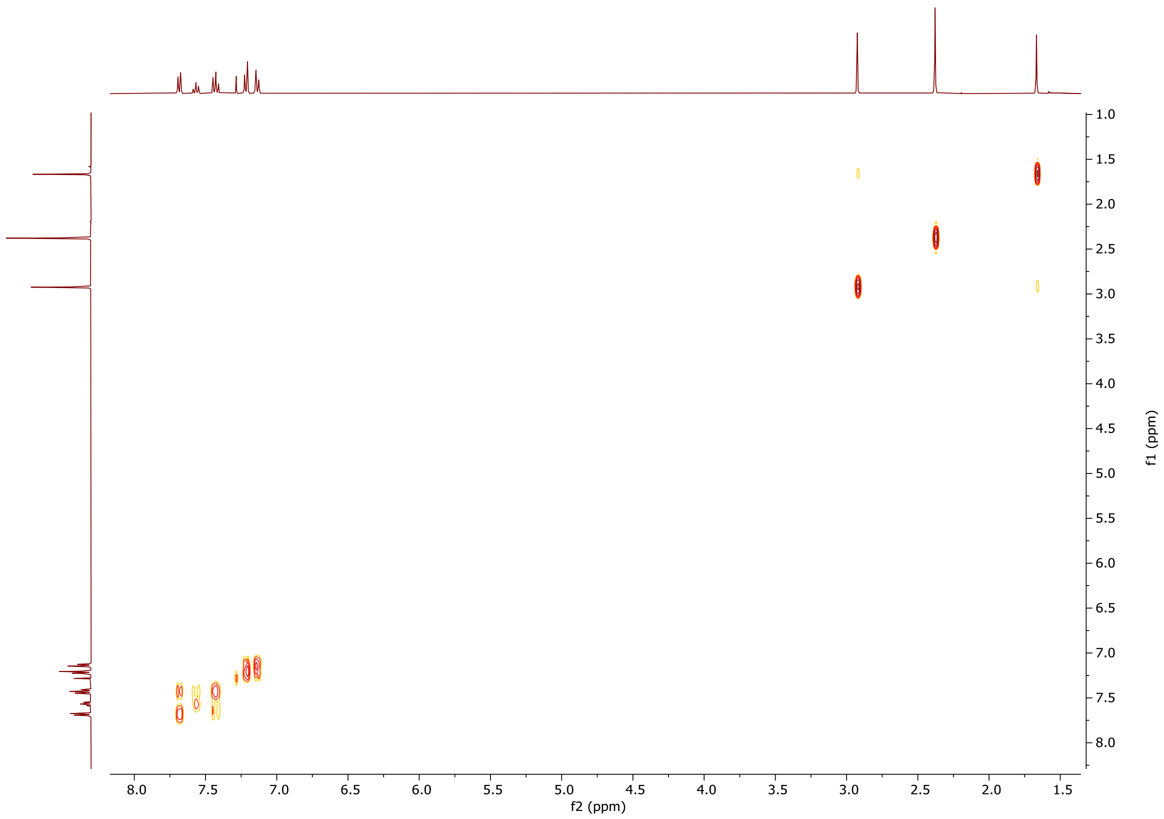

^{1}H/^{13}C HSQC (400/101 MHz, Chloroform-*d*)

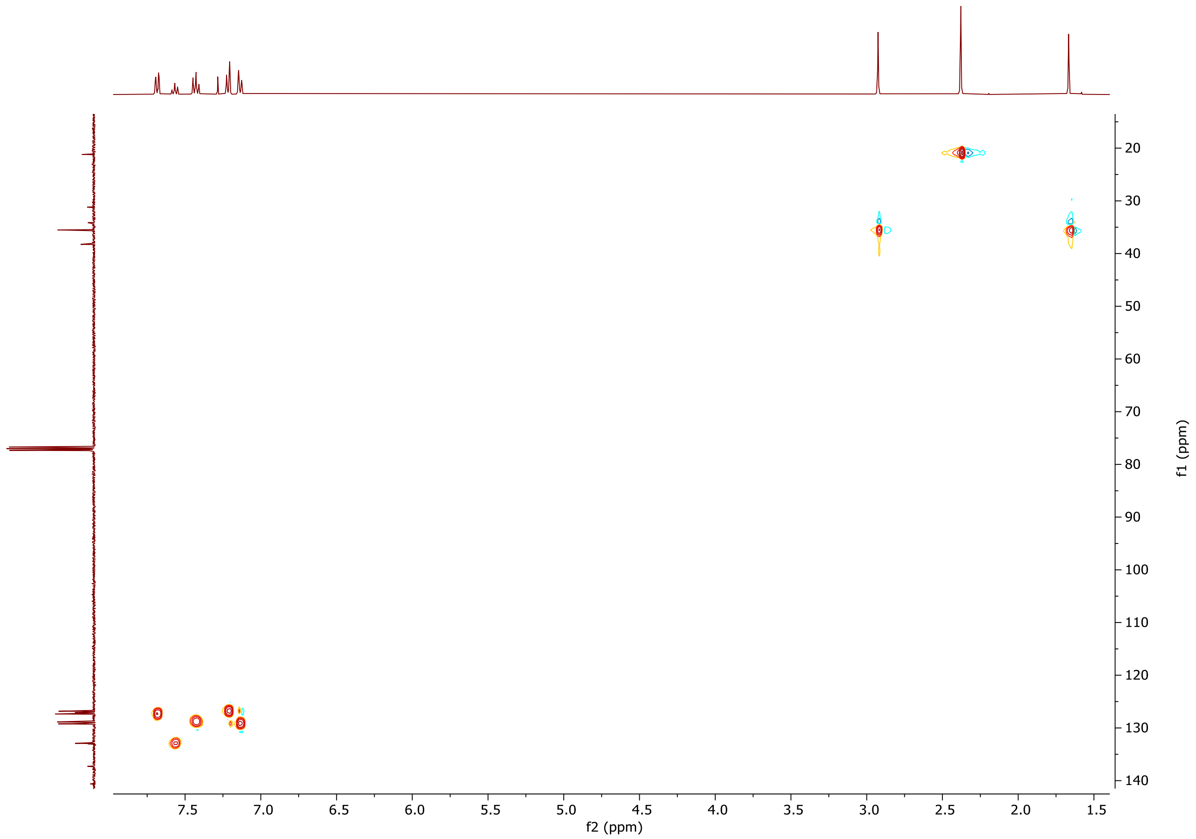

1-(4-methoxyphenyl)-3-(phenylsulfonyl)bicyclo[1.1.0]butane, 19

^{1}H NMR (500 MHz, Benzene-*d*)

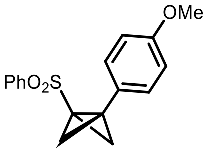

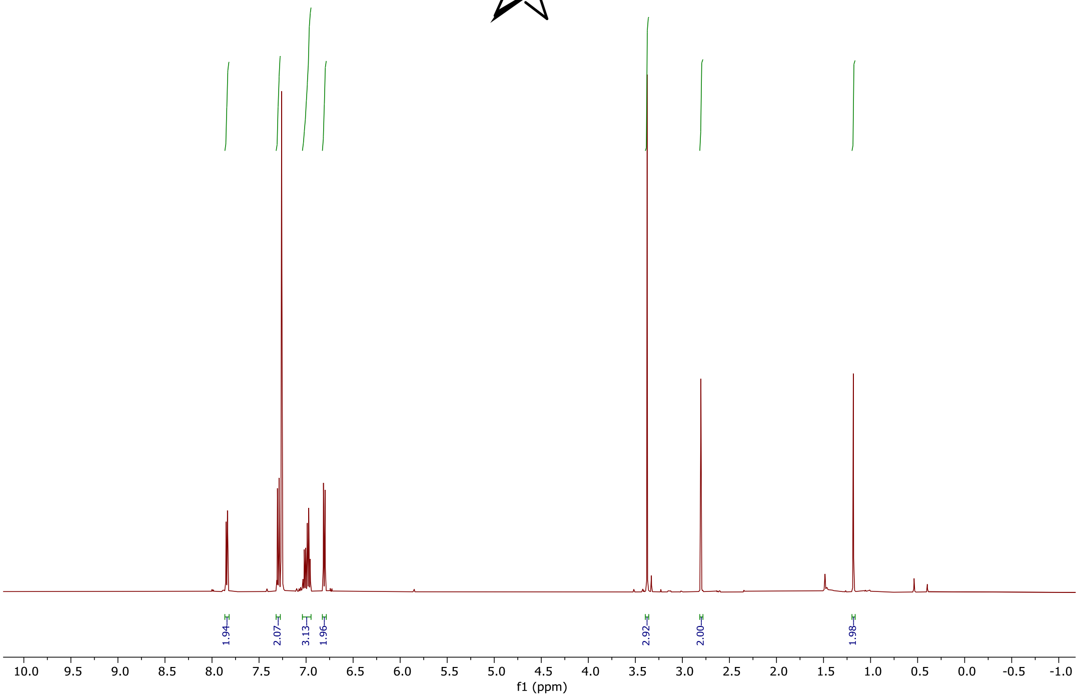

^{13}C NMR (126 MHz, Benzene-*d*)

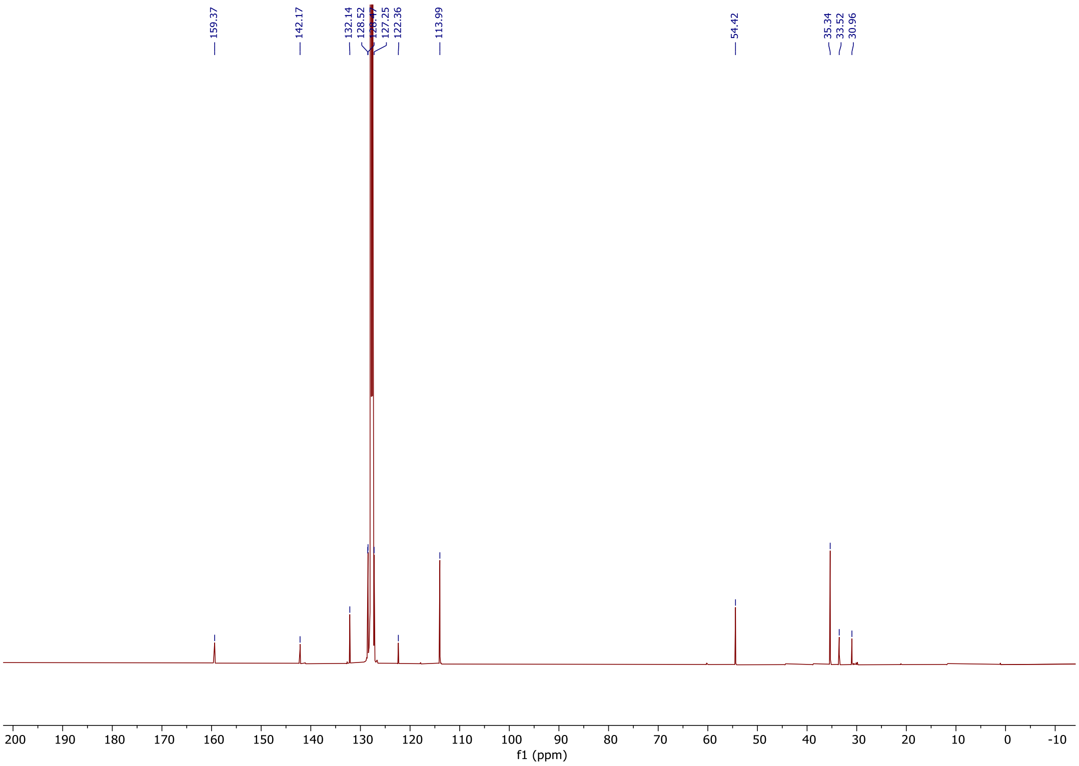

^{1}H COSY (500 MHz, Benzene-*d*)

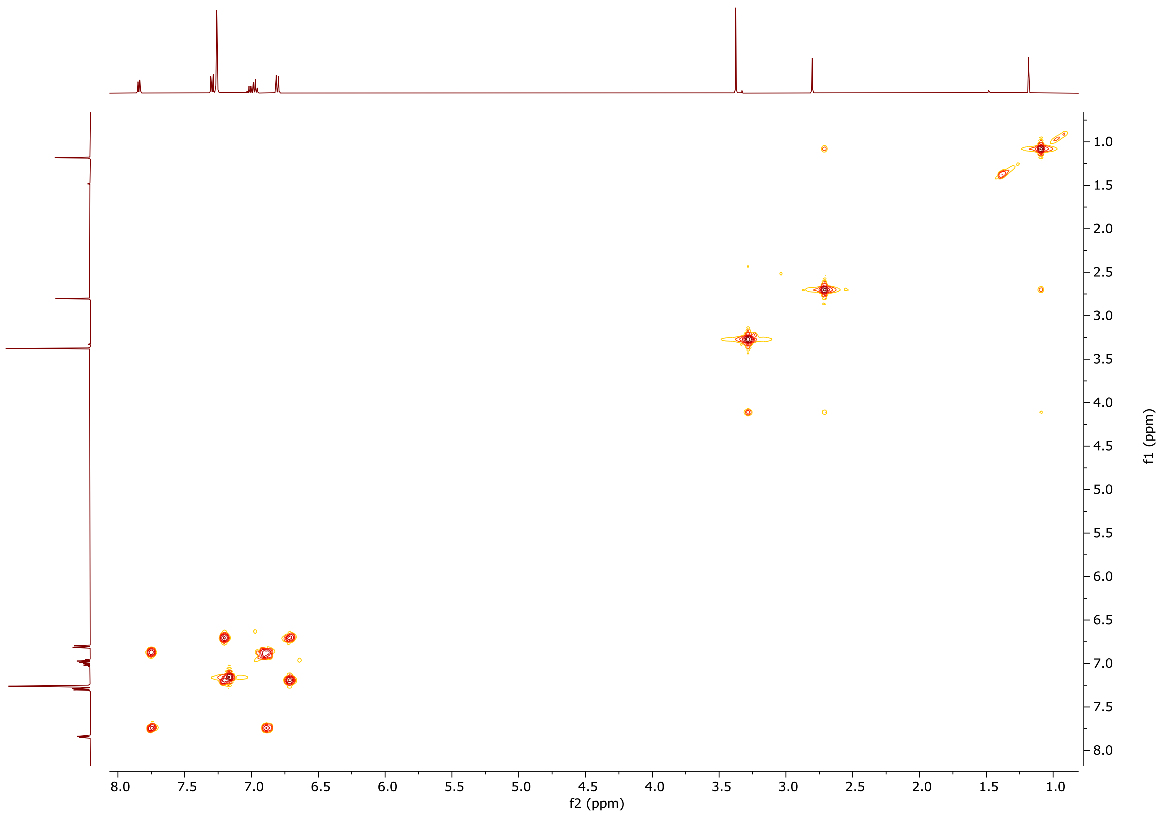

^{1}H/^{13}C HSQC (500/126 MHz, Benzene-*d*)

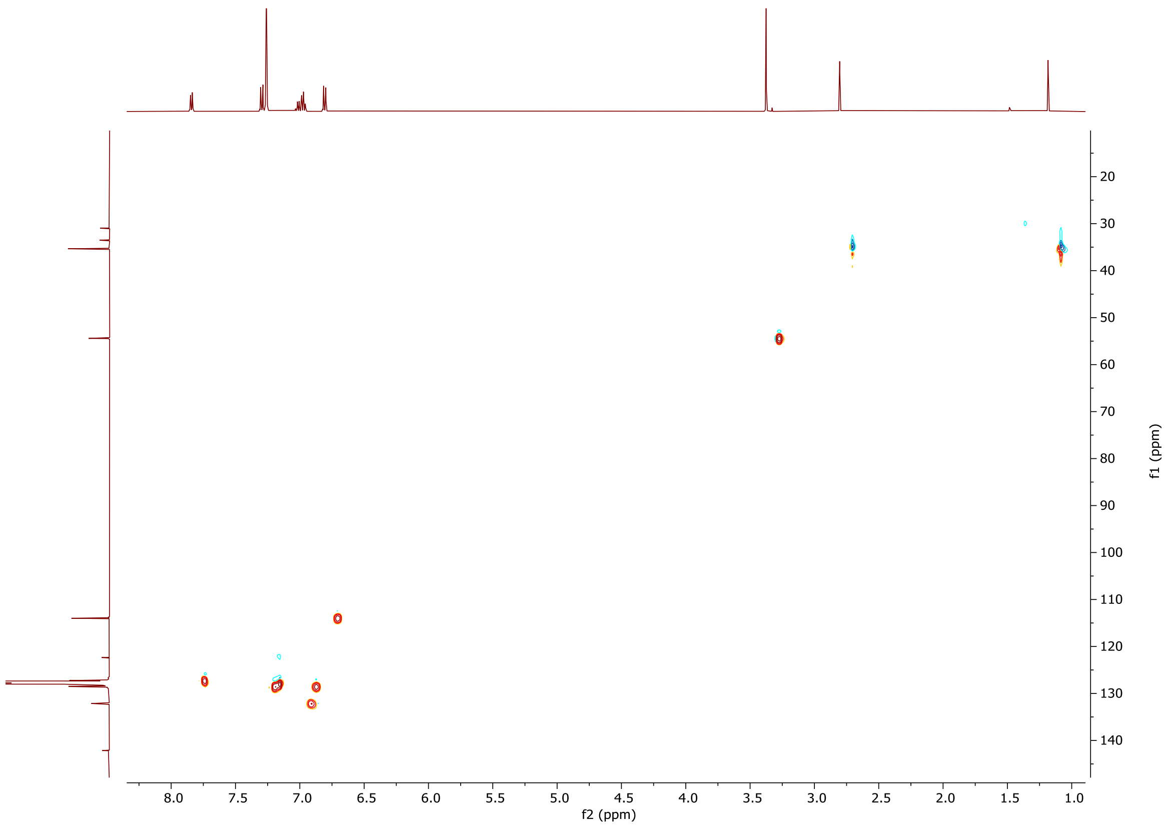

^{1}H/^{13}C HMBC (500/126 MHz, Benzene-*d*)

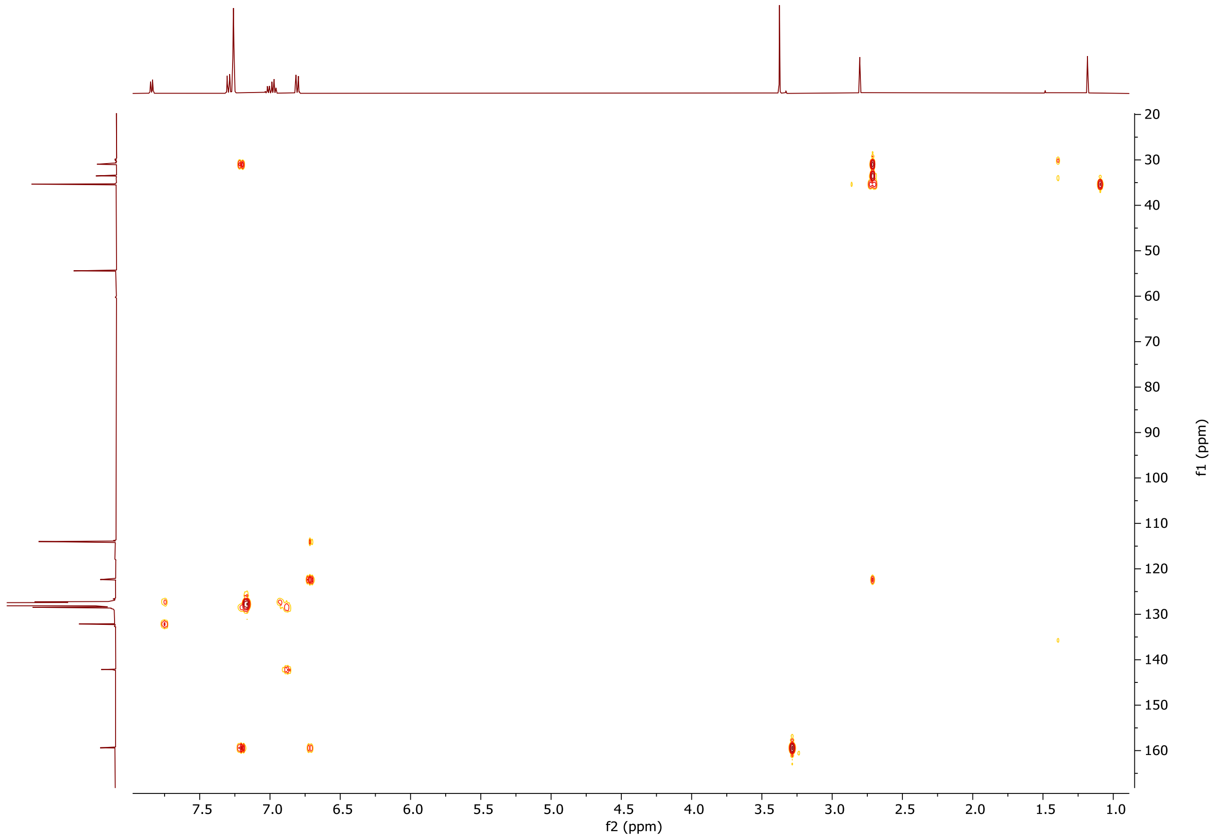

1-(3-bromophenyl)-3-(phenylsulfonyl)bicyclo[1.1.0]butane, 20

^{1}H NMR (500 MHz, Benzene-*d*)

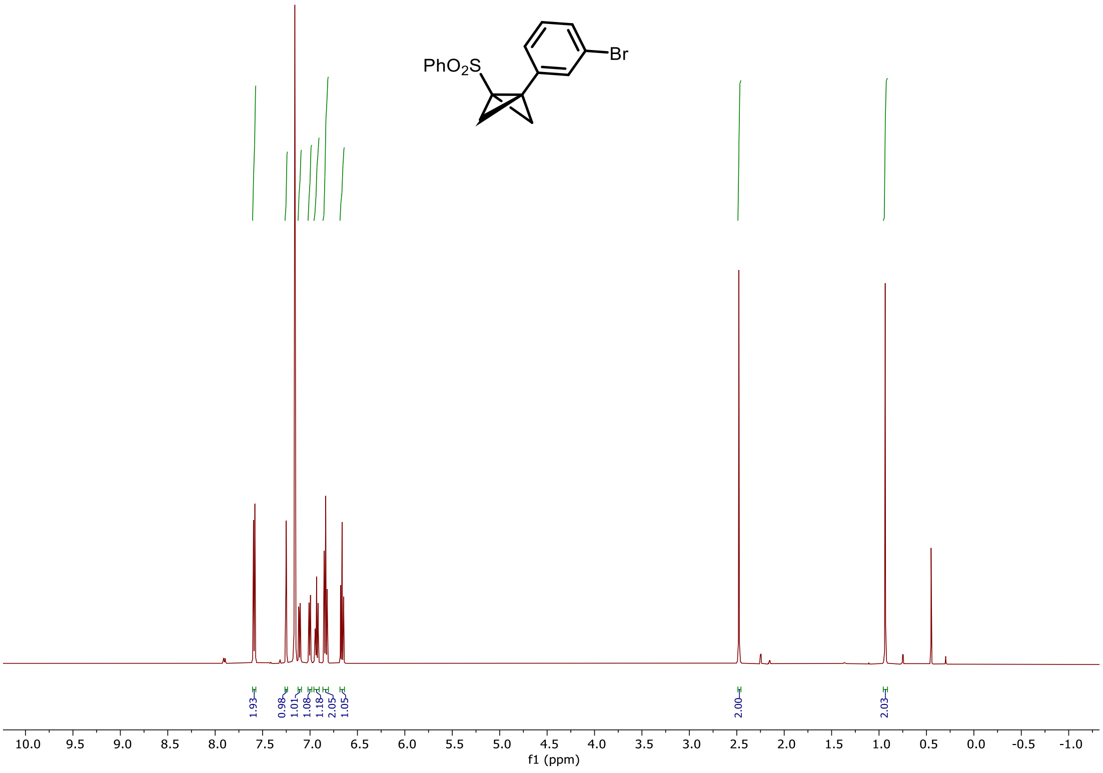

^{13}C NMR (126 MHz, Benzene-*d*)

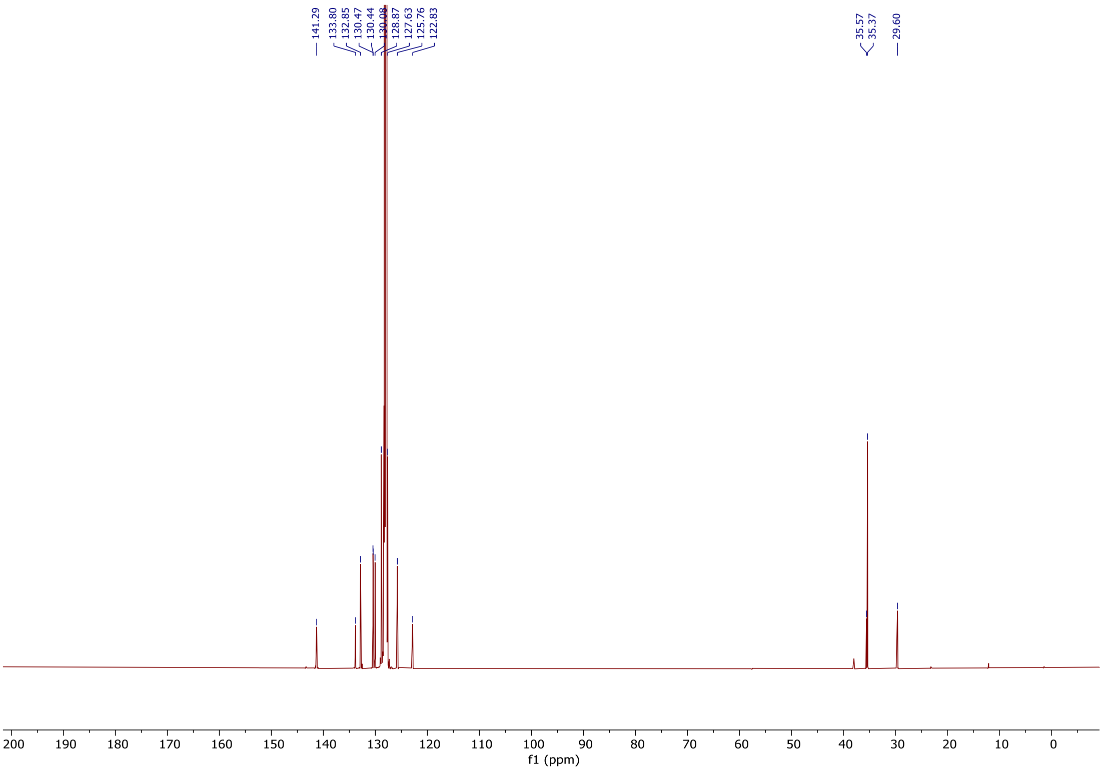

^{1}H COSY (500 MHz, Benzene-*d*)

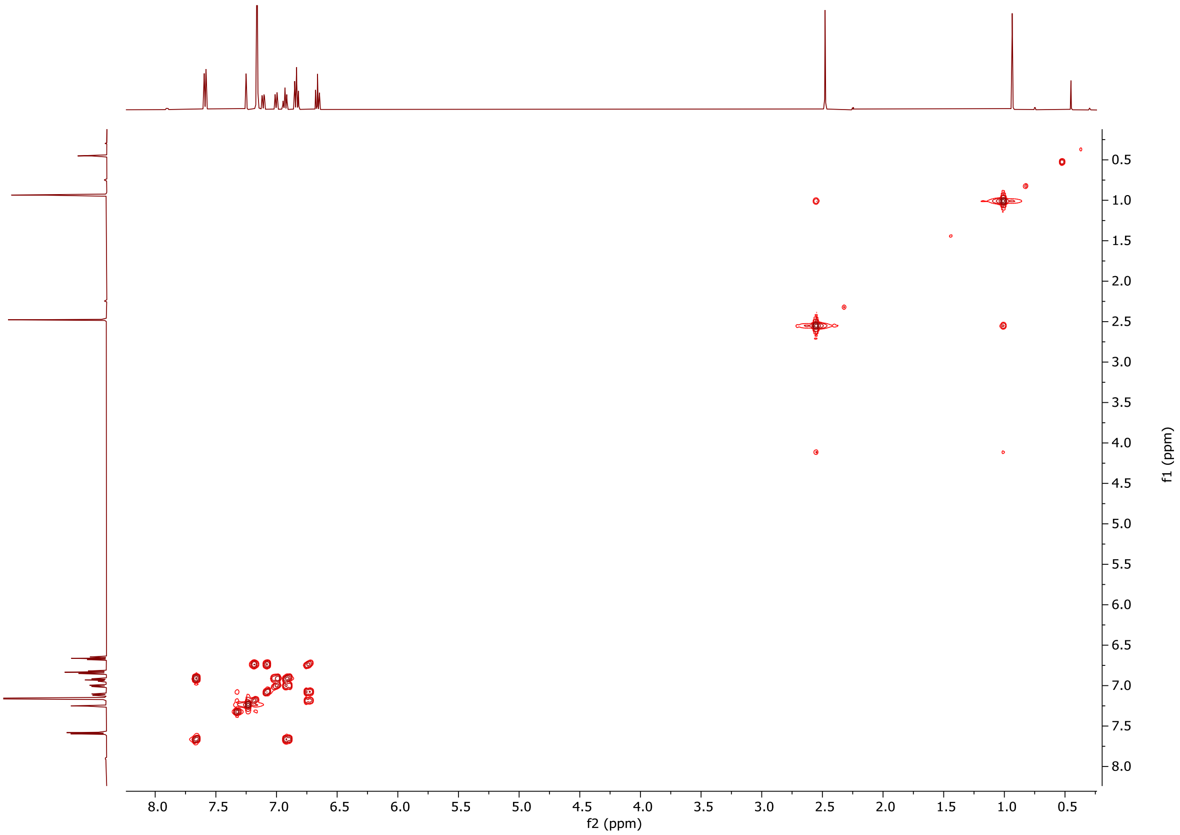

^{1}H/^{13}C HSQC (500/126 MHz, Benzene-*d*)

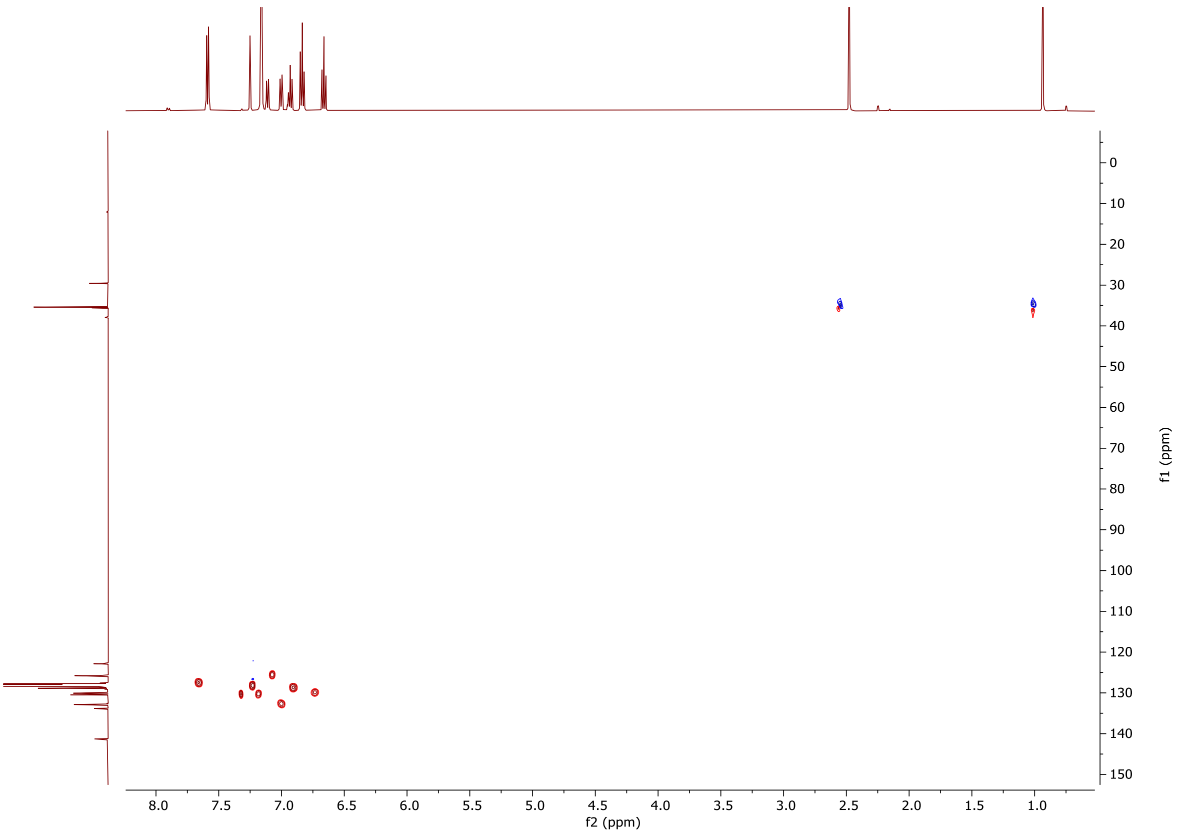

^{1}H/^{13}C HMBC (500/126 MHz, Benzene-*d*)

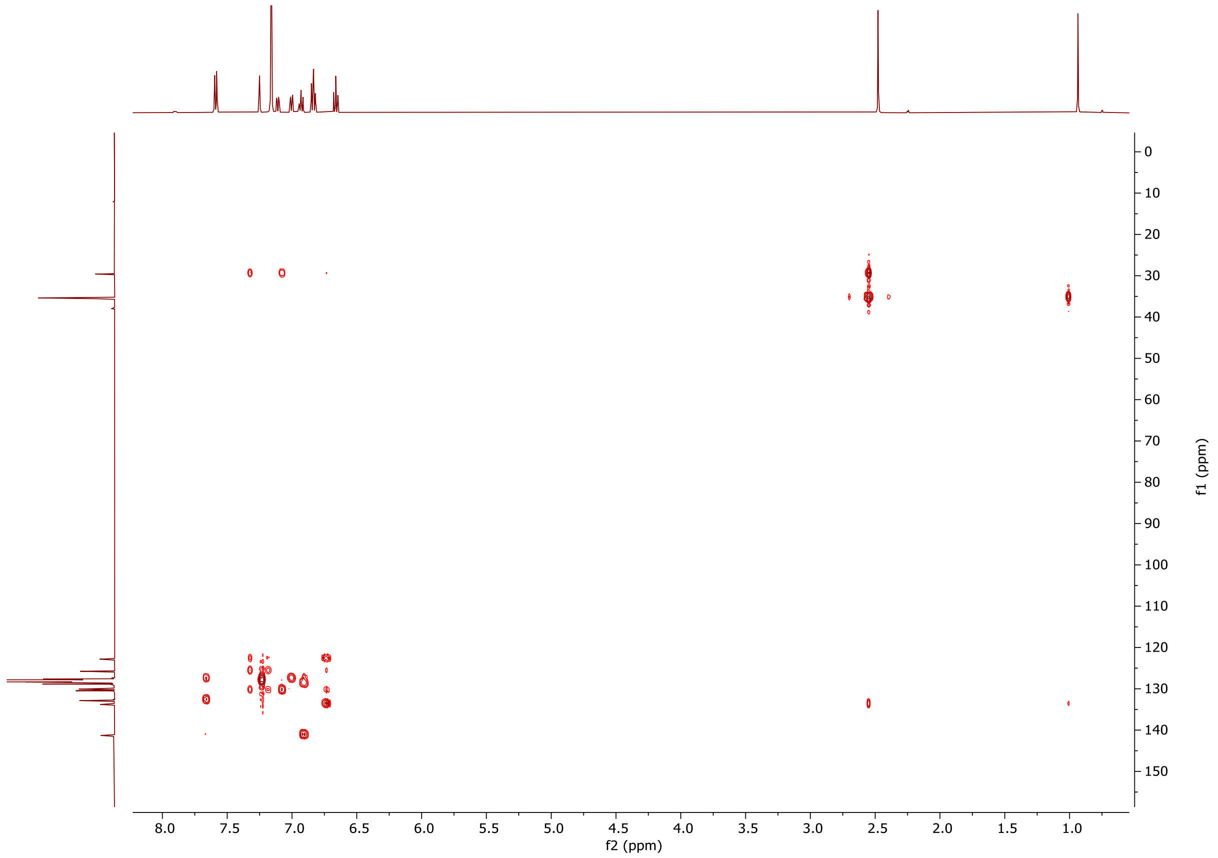

3-(3-(phenylsulfonyl)bicyclo[1.1.0]butan-1-yl)phenyl trifluoromethanesulfonate, 21

^{1}H NMR (500 MHz, Chloroform-*d*)

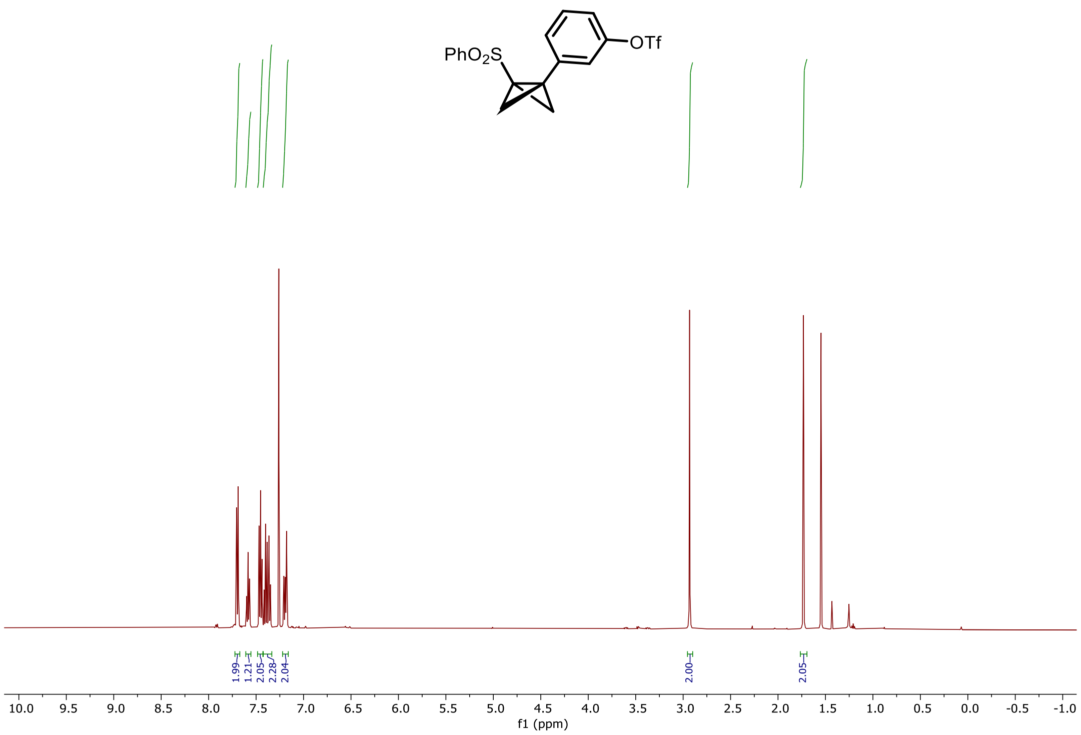

^{13}C NMR (126 MHz, Chloroform-*d*)

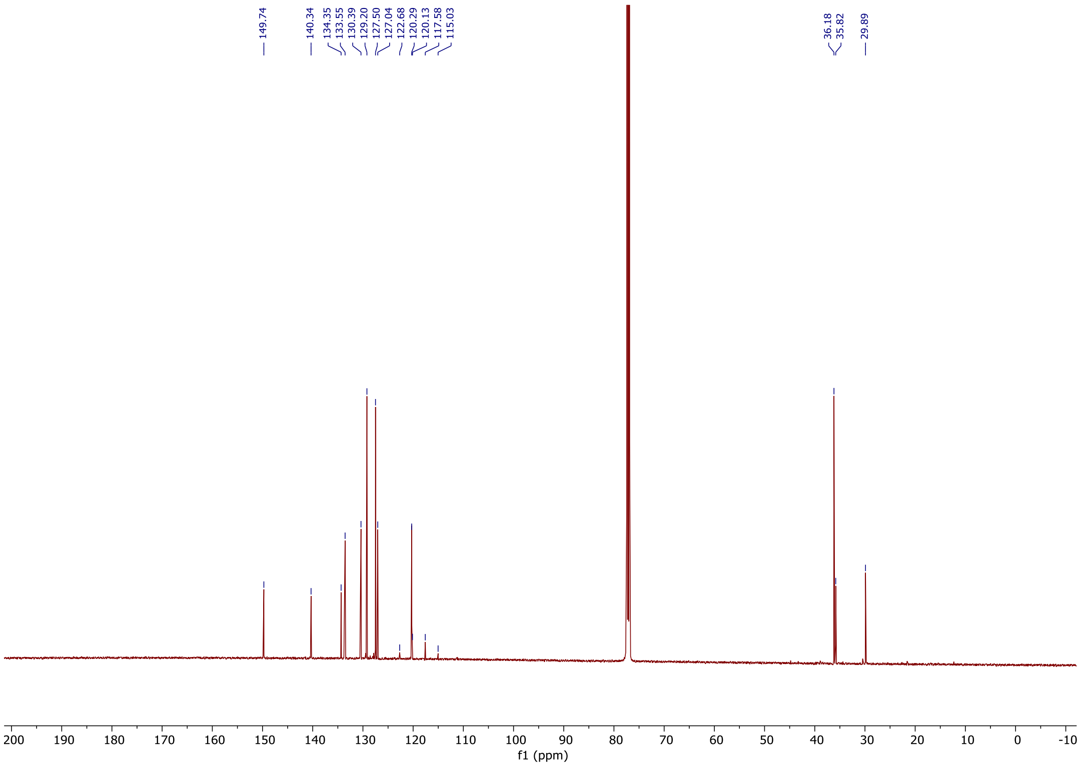

^{1}H COSY (500 MHz, Chloroform-*d*)

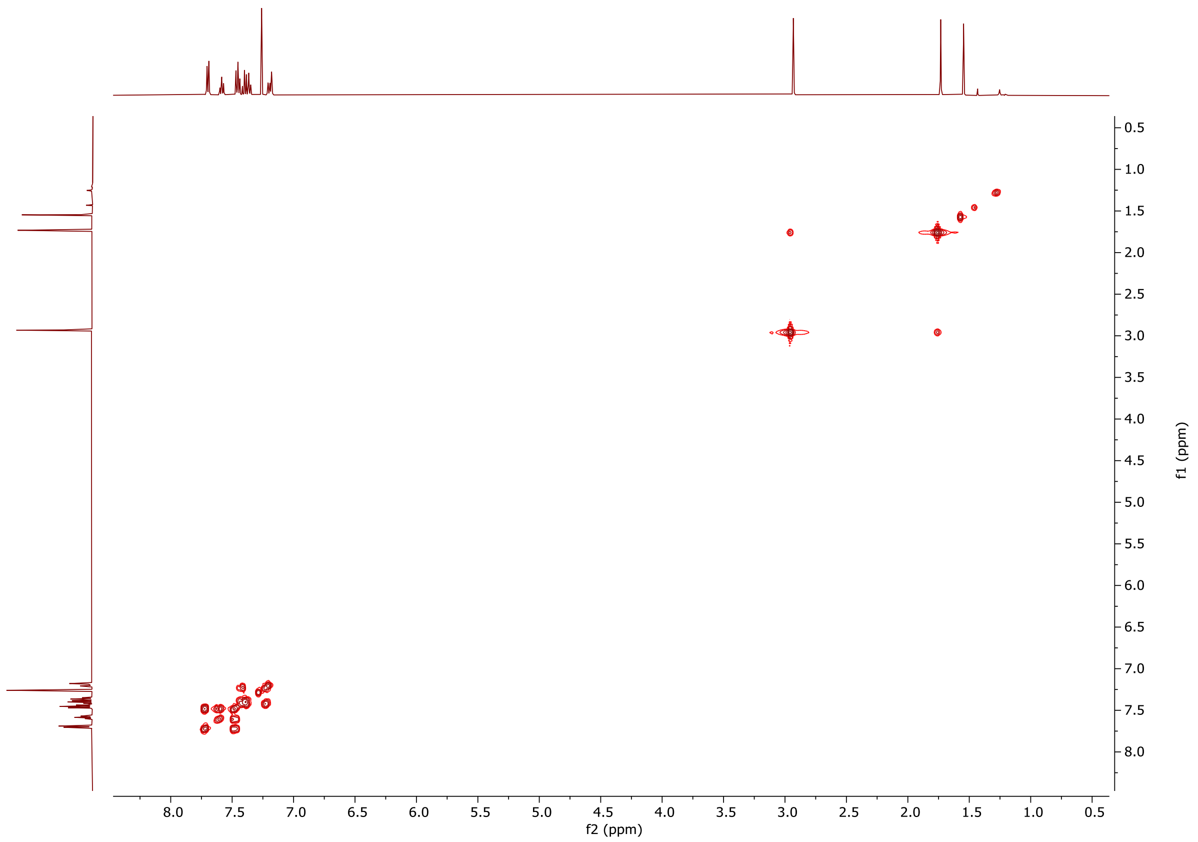

^{1}H/^{13}C HSQC (500/126 MHz, Chloroform-*d*)

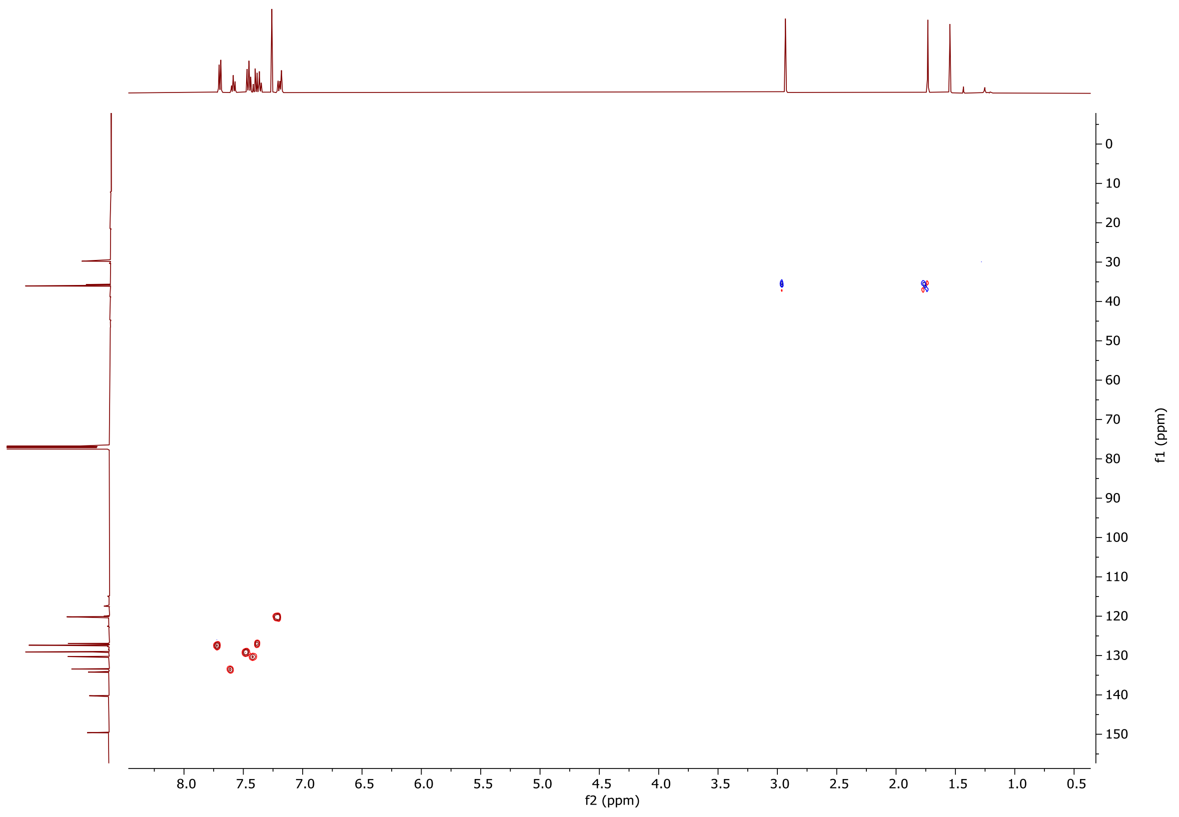

$^{1}H/^{13}C$ HMBC (500/126 MHz, Chloroform-*d*)

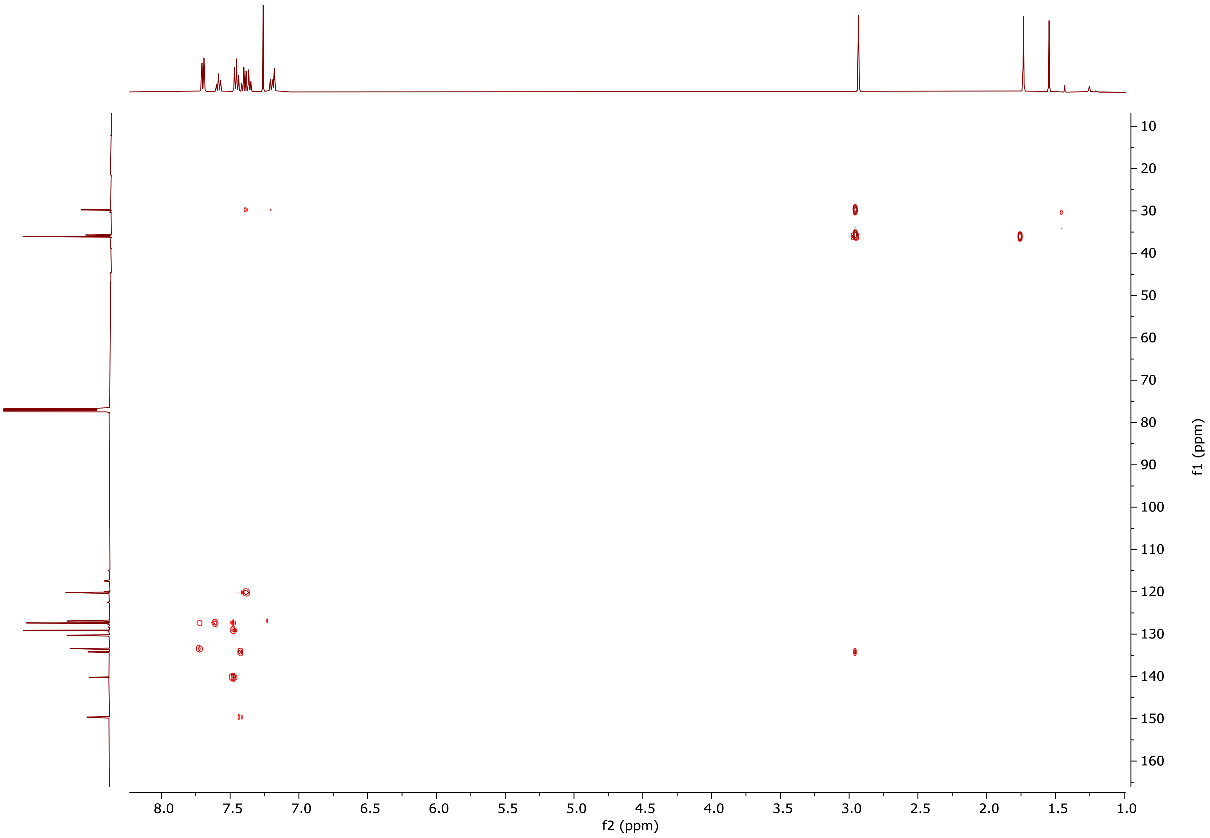

^{19}F NMR (470 MHz, Chloroform-*d*)

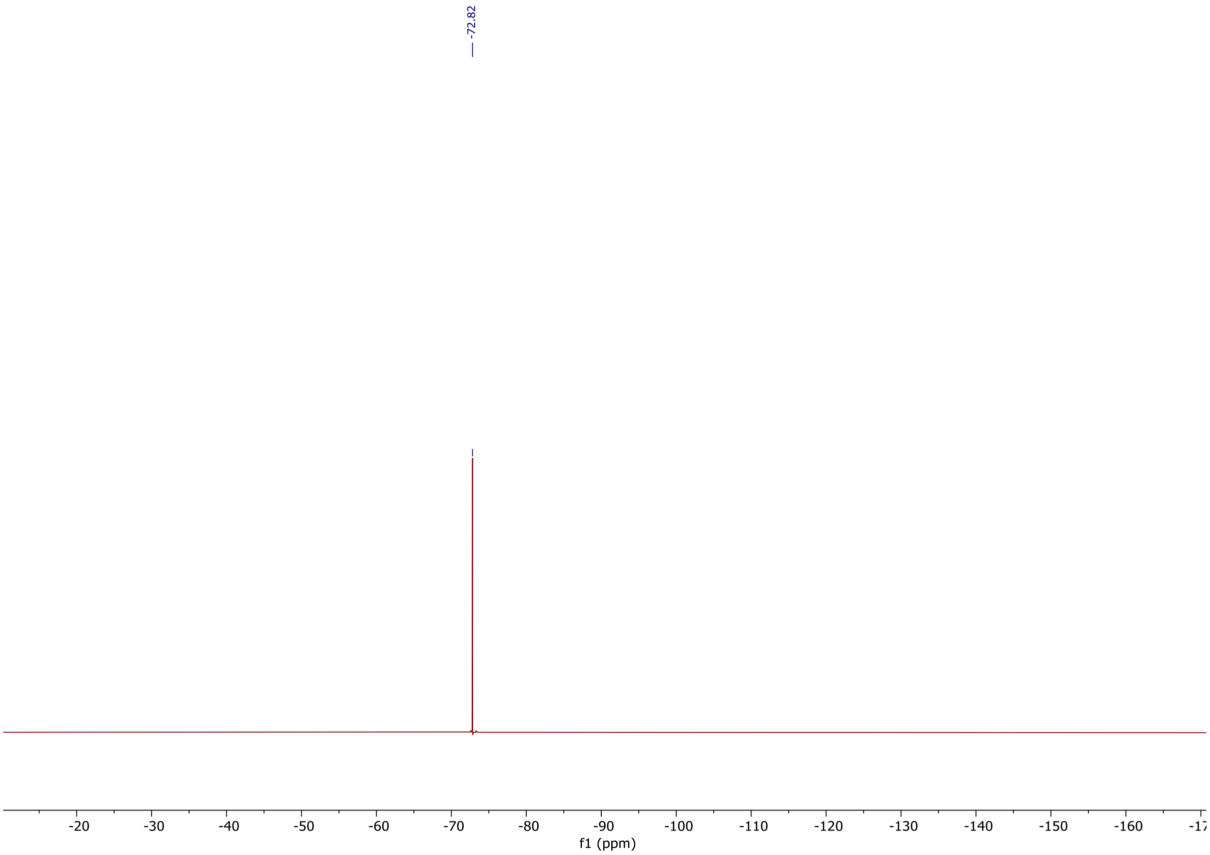

***Tert*-butyldimethyl(3-(3-(phenylsulfonyl)bicyclo[1.1.0]butan-1-yl)phenoxy)silane, 22**

^{1}H NMR (500 MHz, Benzene-*d*)

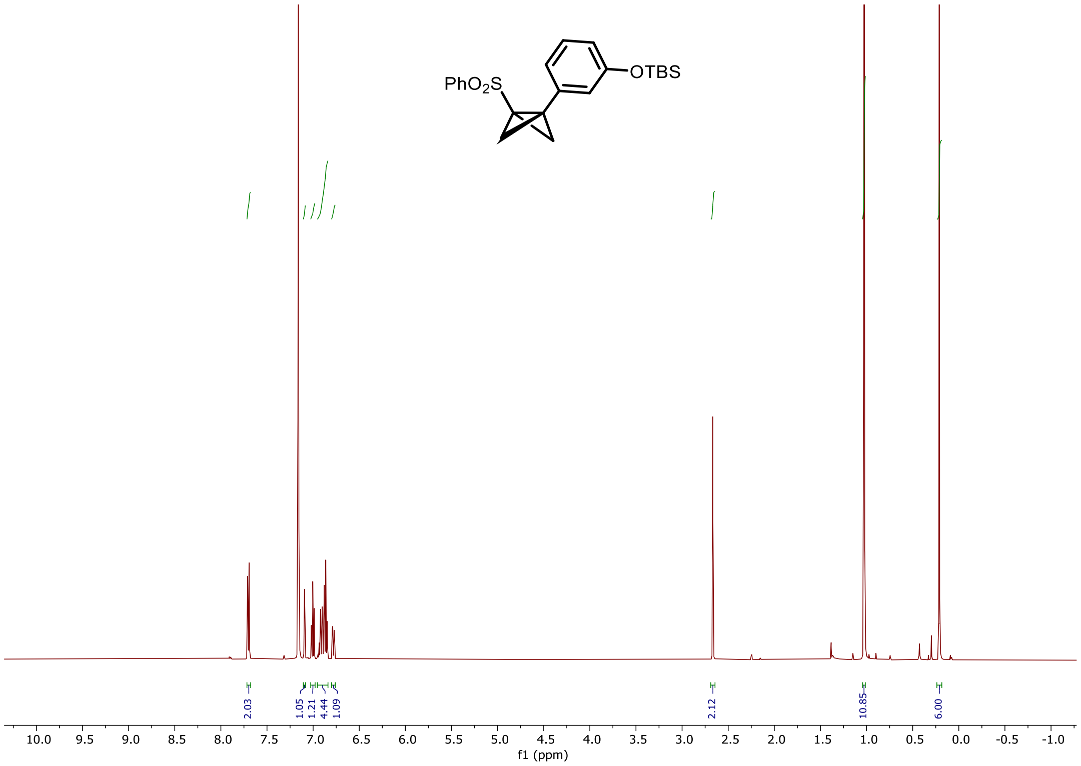

^{13}C NMR (126 MHz, Benzene-*d*)

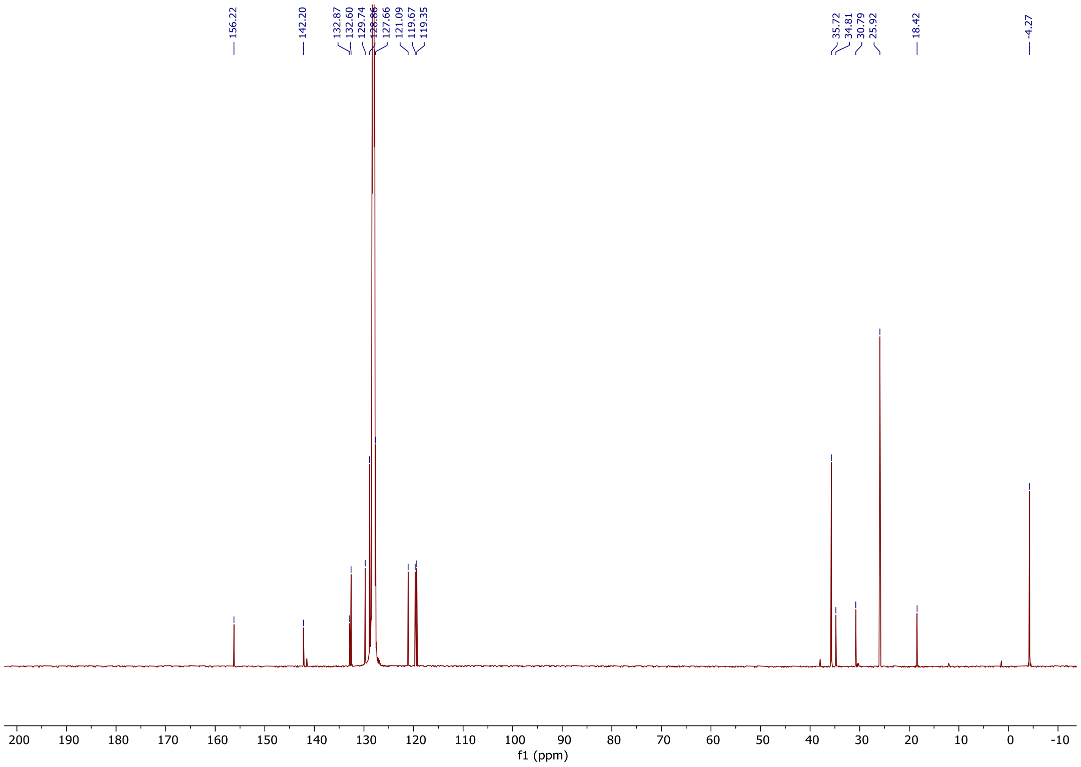

^{1}H COSY (500 MHz, Benzene-*d*)

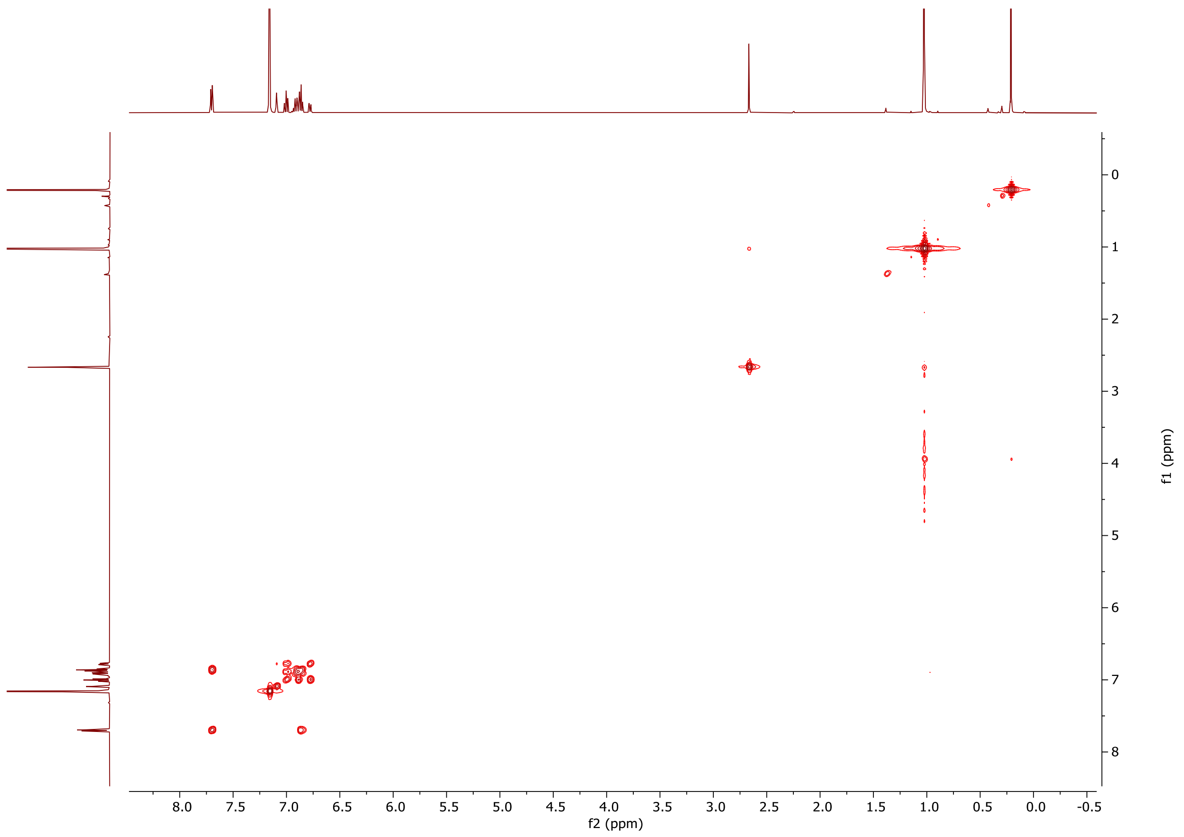

^{1}H/^{13}C HSQC (500/126 MHz, Benzene-*d*)

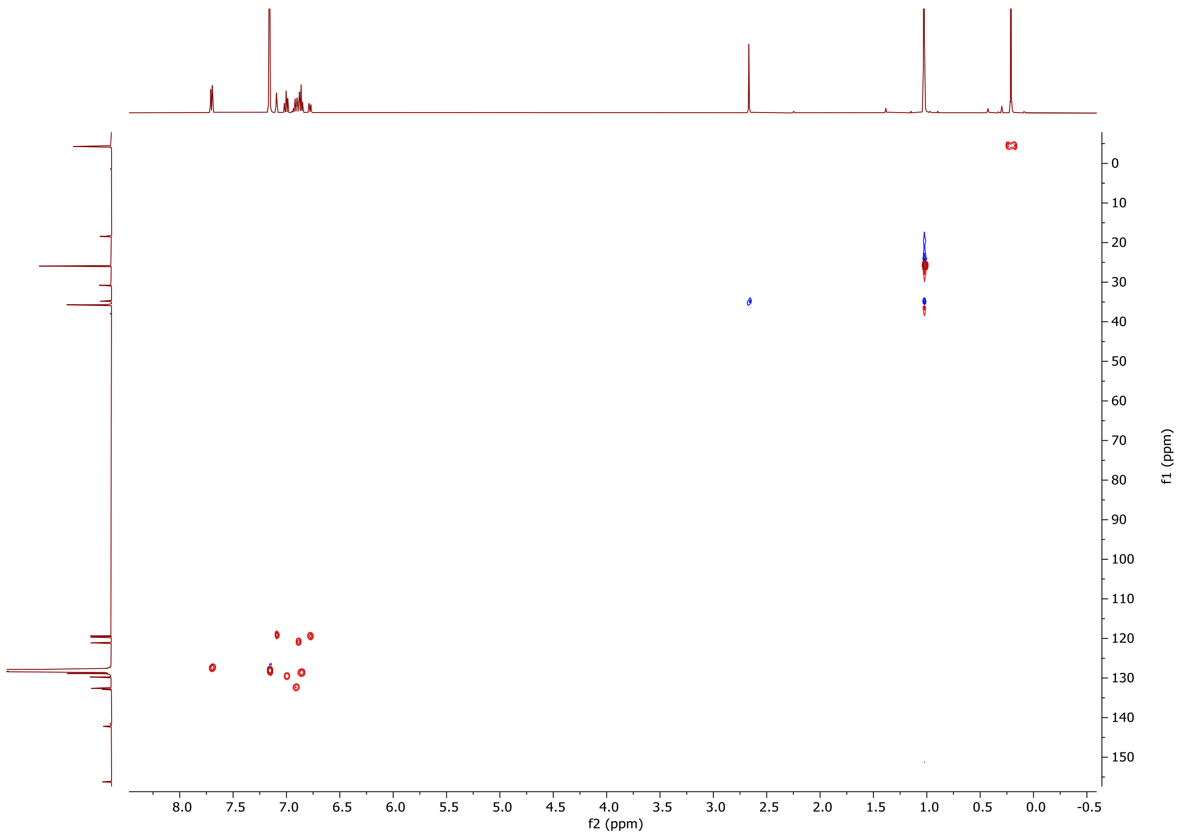

$^{1}H/^{13}C$ HMBC (500/126 MHz, Benzene-*d*)

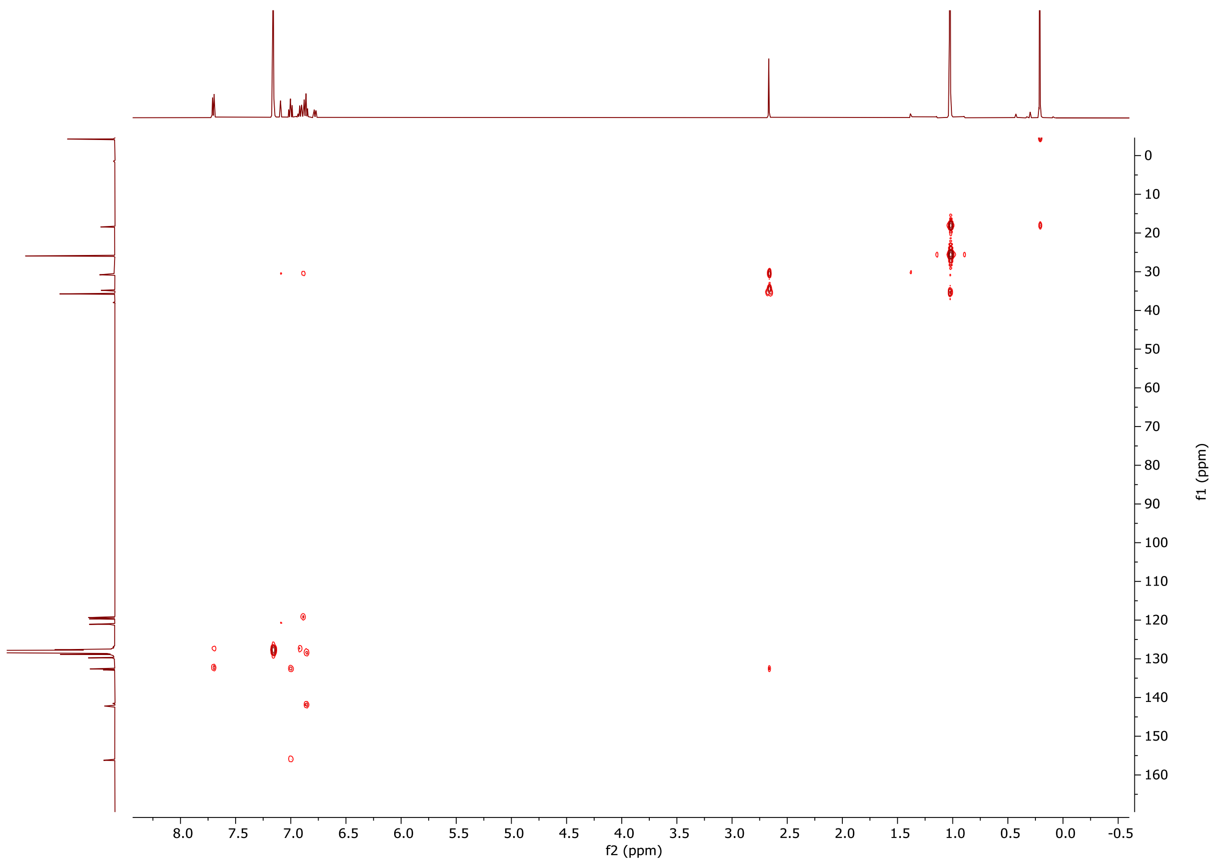

1-(phenylsulfonyl)-3-(2-(trifluoromethyl)phenyl)bicyclo[1.1.0]butane, 23

^{1}H NMR (500 MHz, Chloroform-*d*)

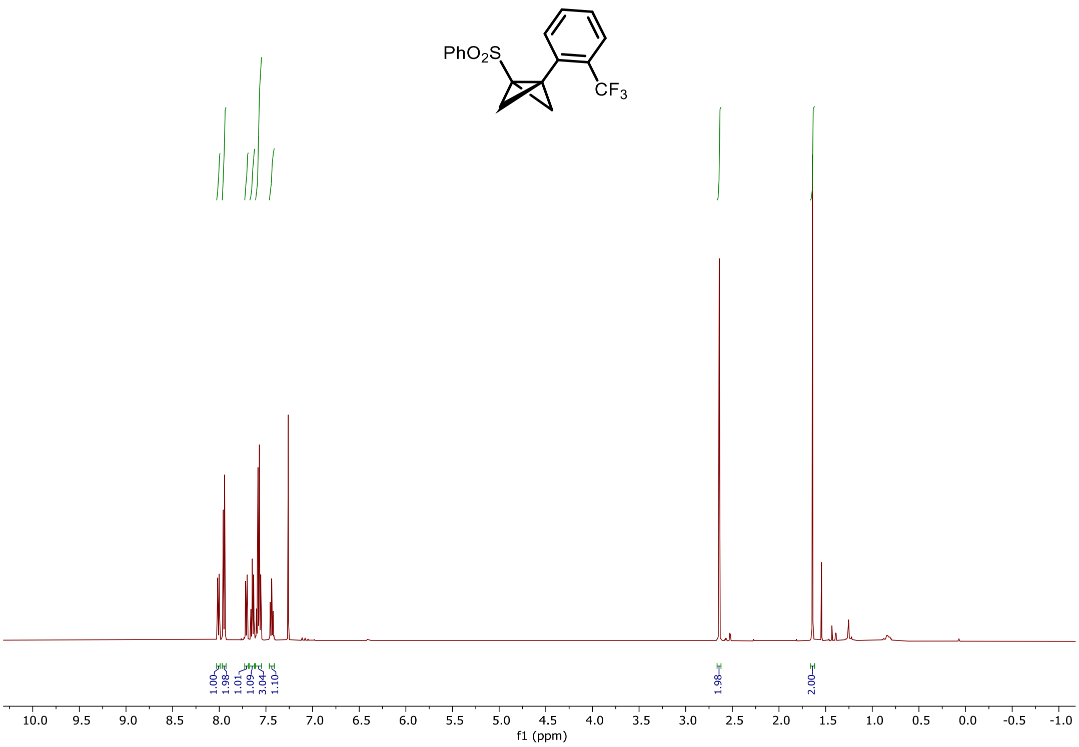

^{13}C NMR (126 MHz, Chloroform-*d*)

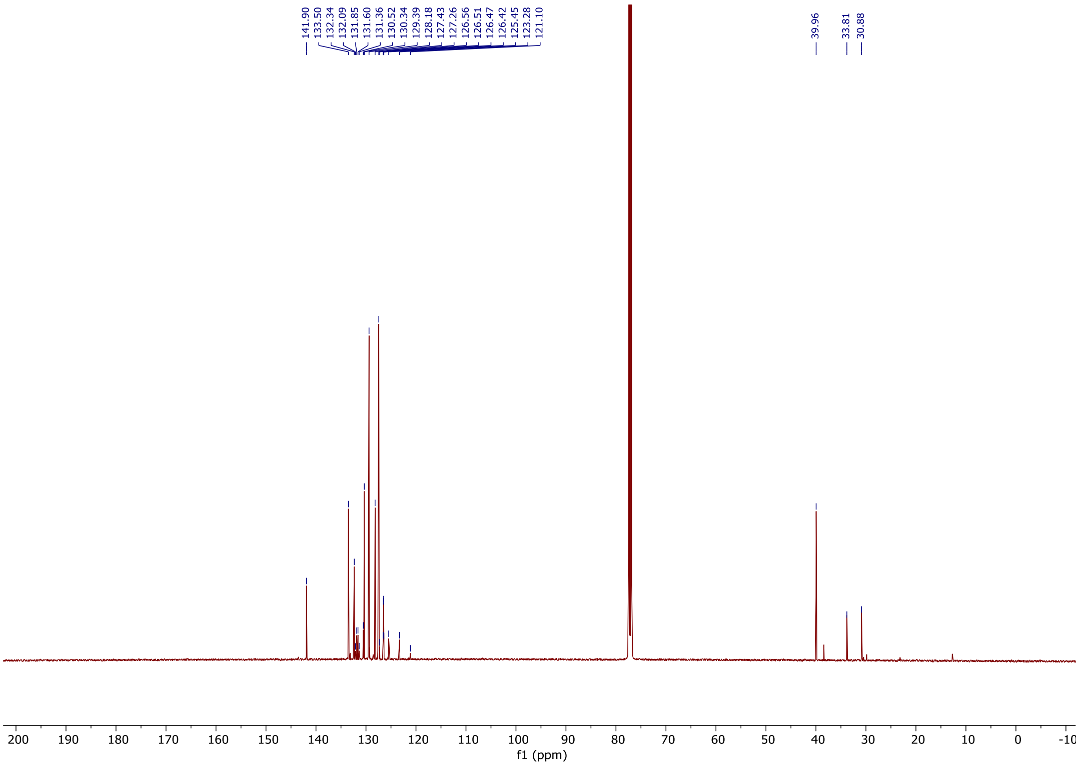

^{1}H COSY (500 MHz, Chloroform-*d*)

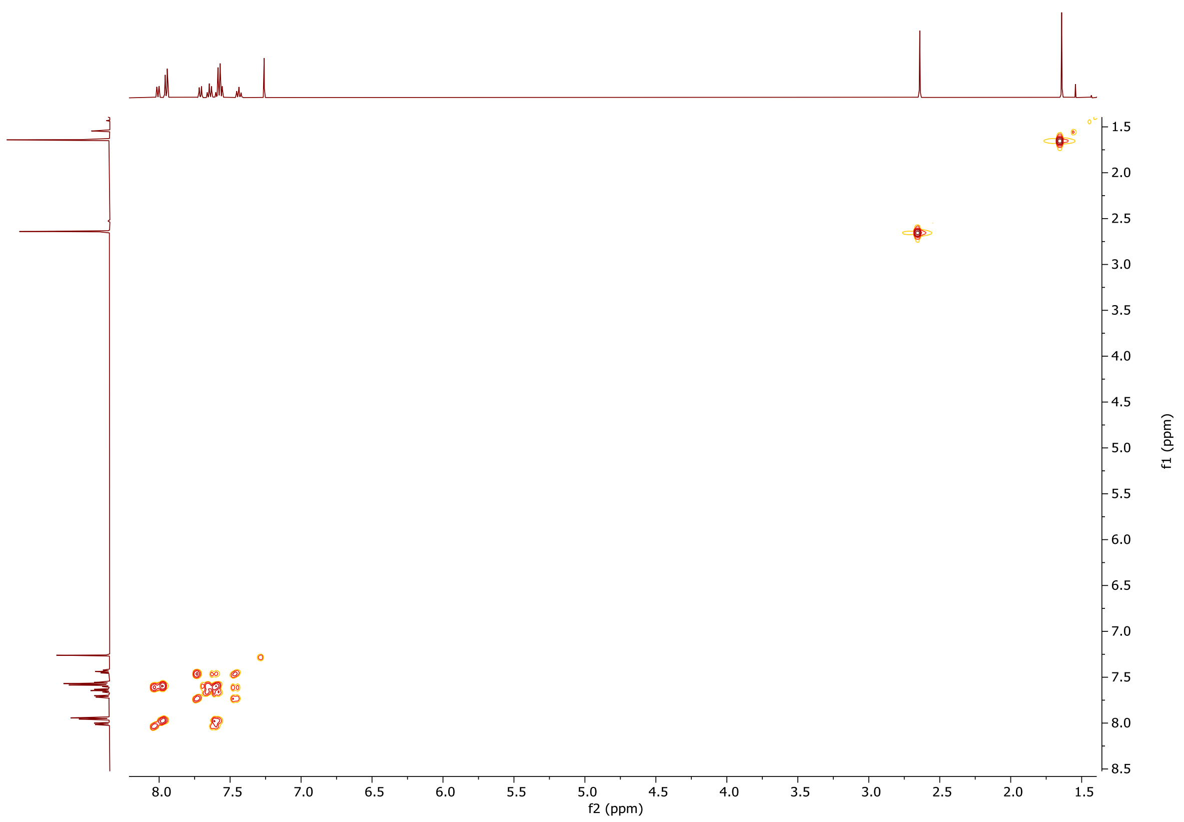

$^{1}H/^{13}C$ HSQC (500/126 MHz, Chloroform-*d*)

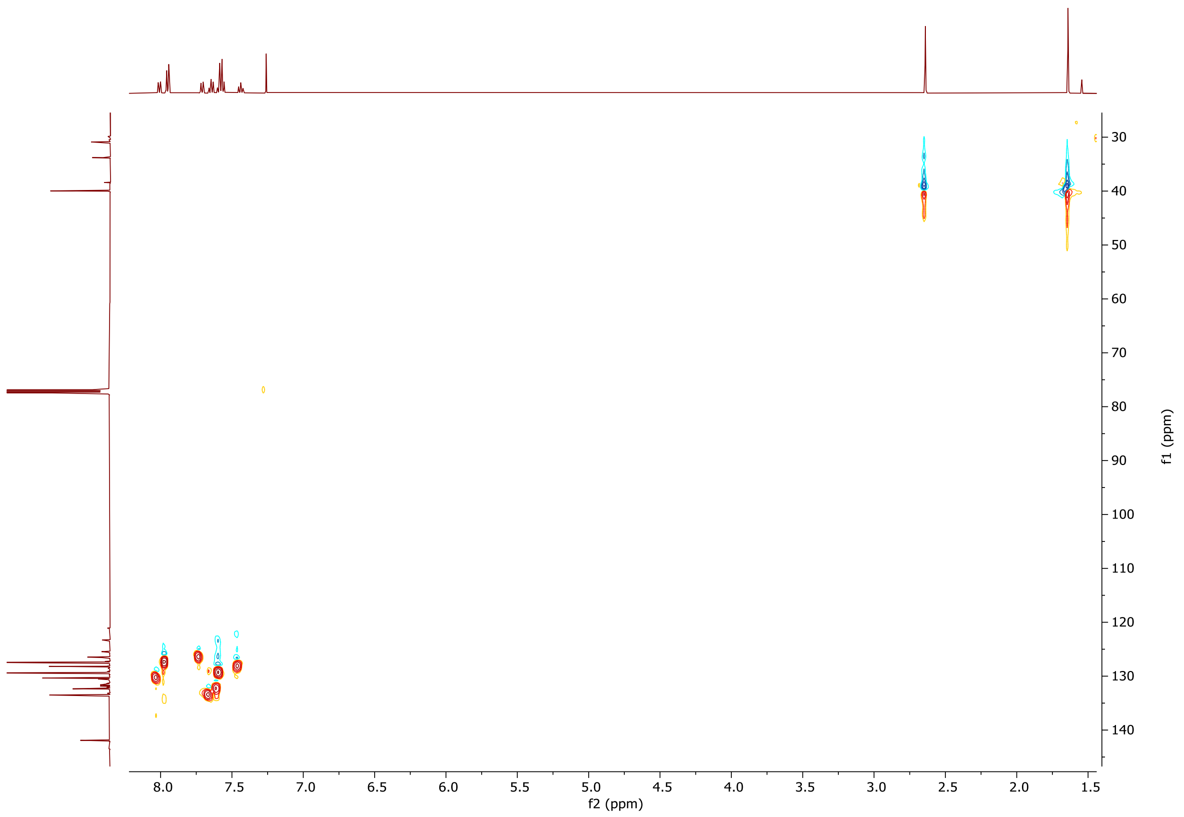

$^{1}H/^{13}C$ HMBC (500/126 MHz, Chloroform-*d*)

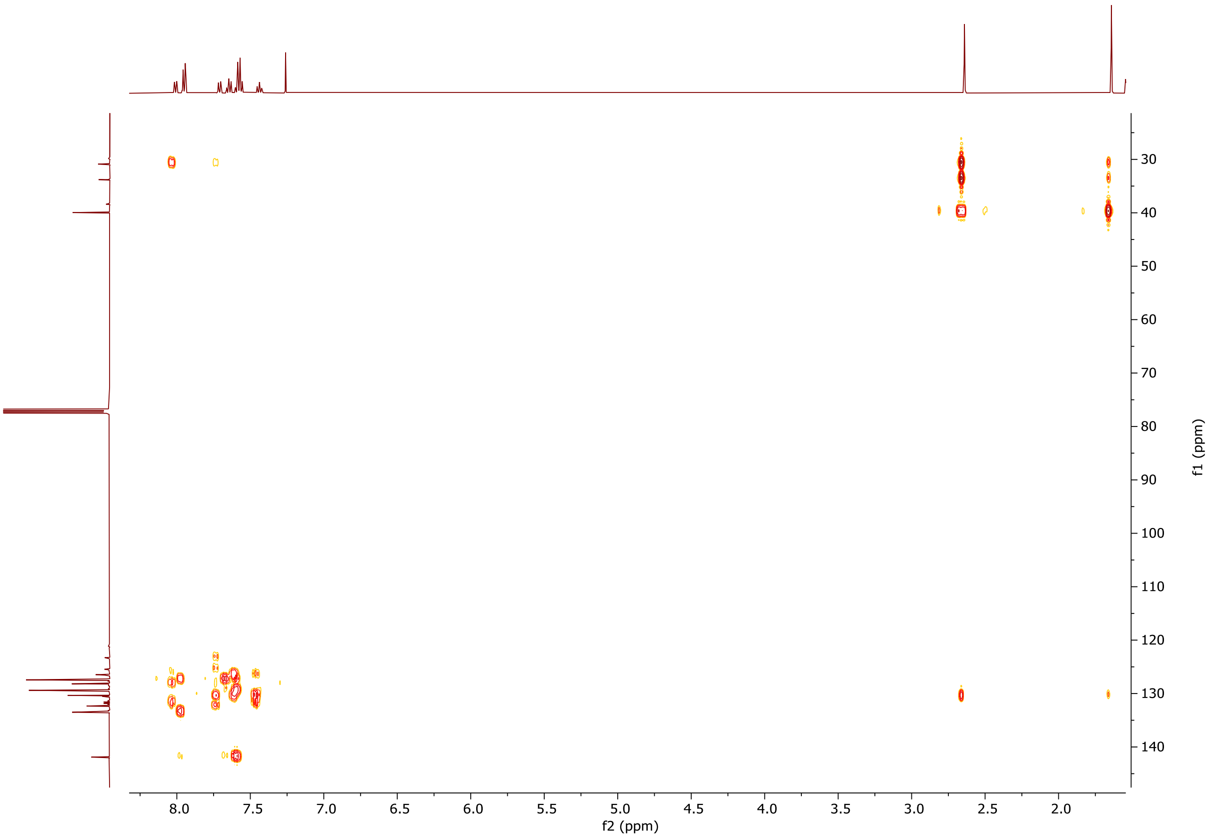

^{19}F NMR (470 MHz, Chloroform-*d*)

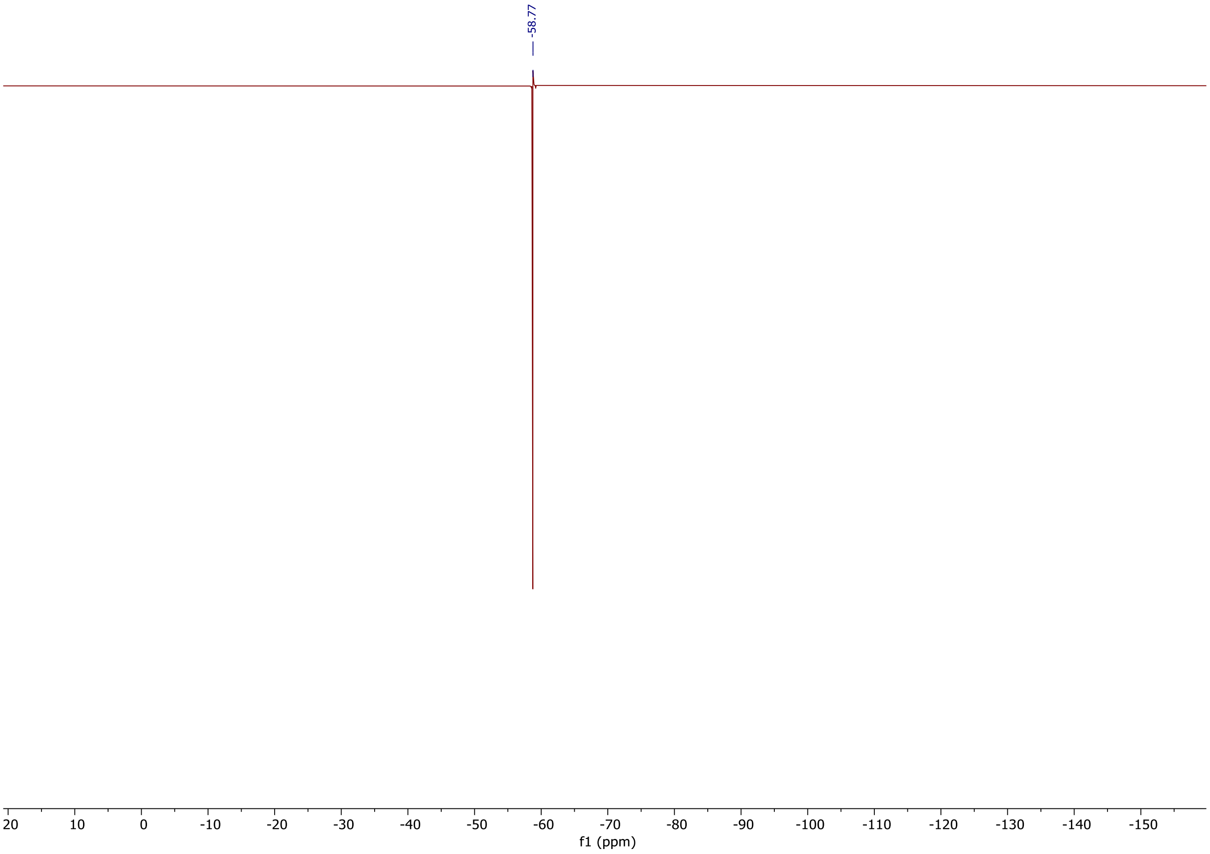

1-(phenylsulfonyl)-3-(o-tolyl)bicyclo[1.1.0]butane, 24

^{1}H NMR (500 MHz, Chloroform-*d*)

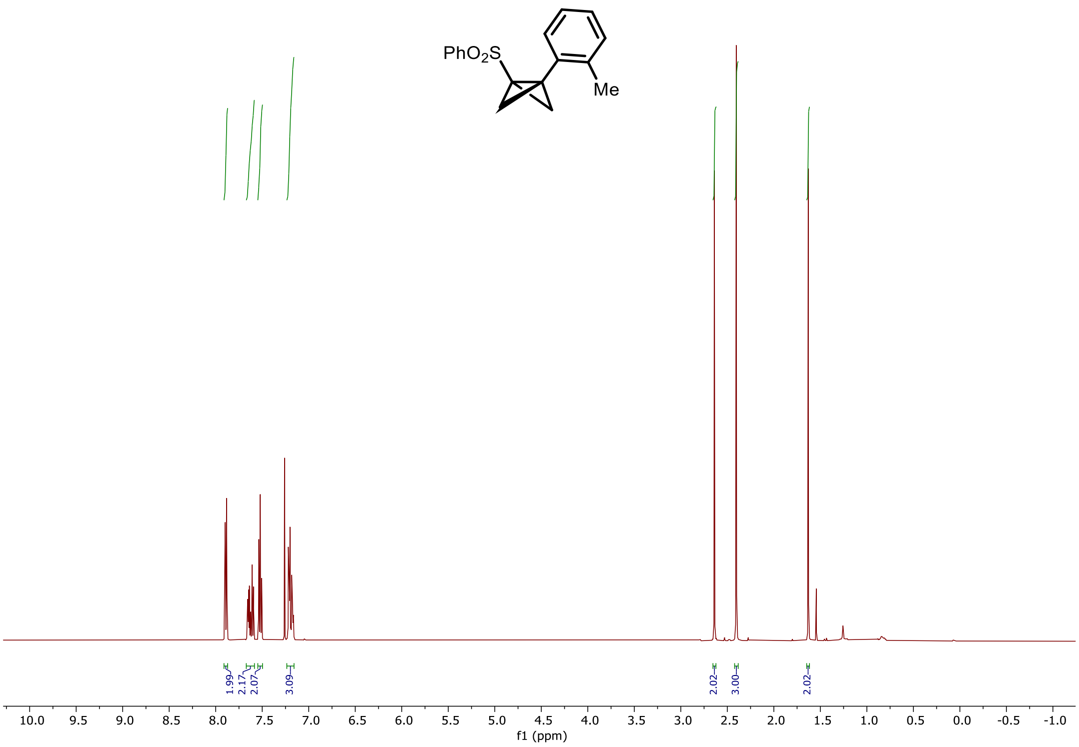

^{13}C NMR (126 MHz, Chloroform-*d*)

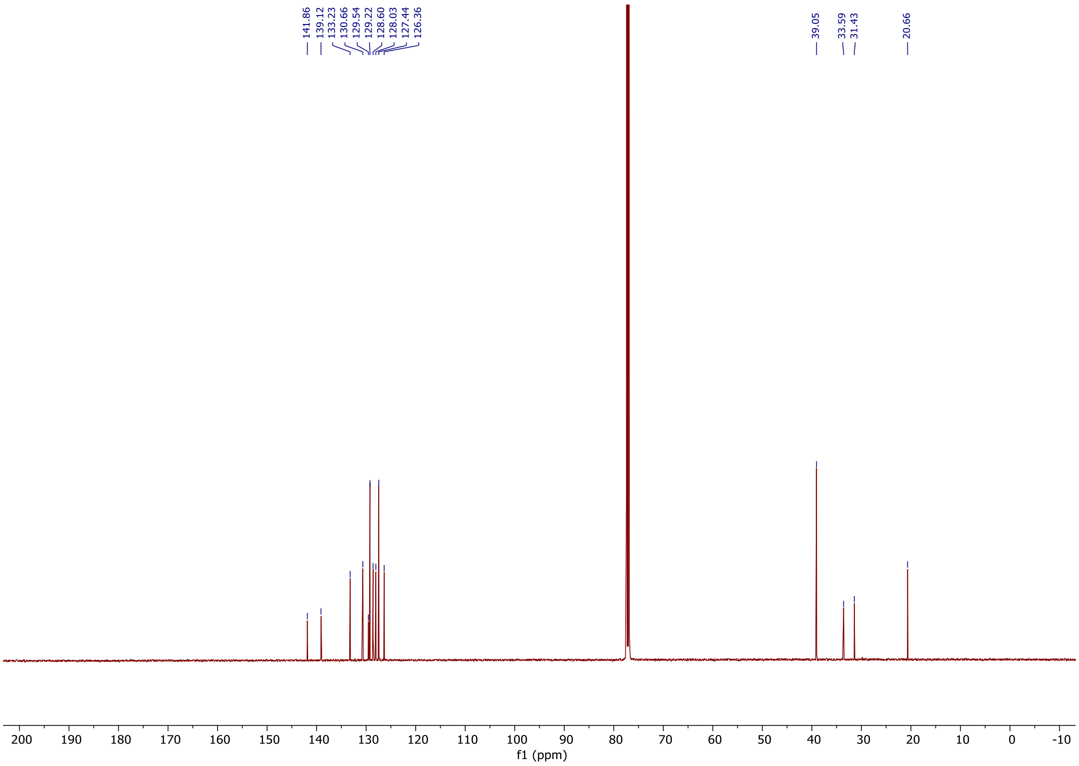

^{1}H COSY (500 MHz, Chloroform-*d*)

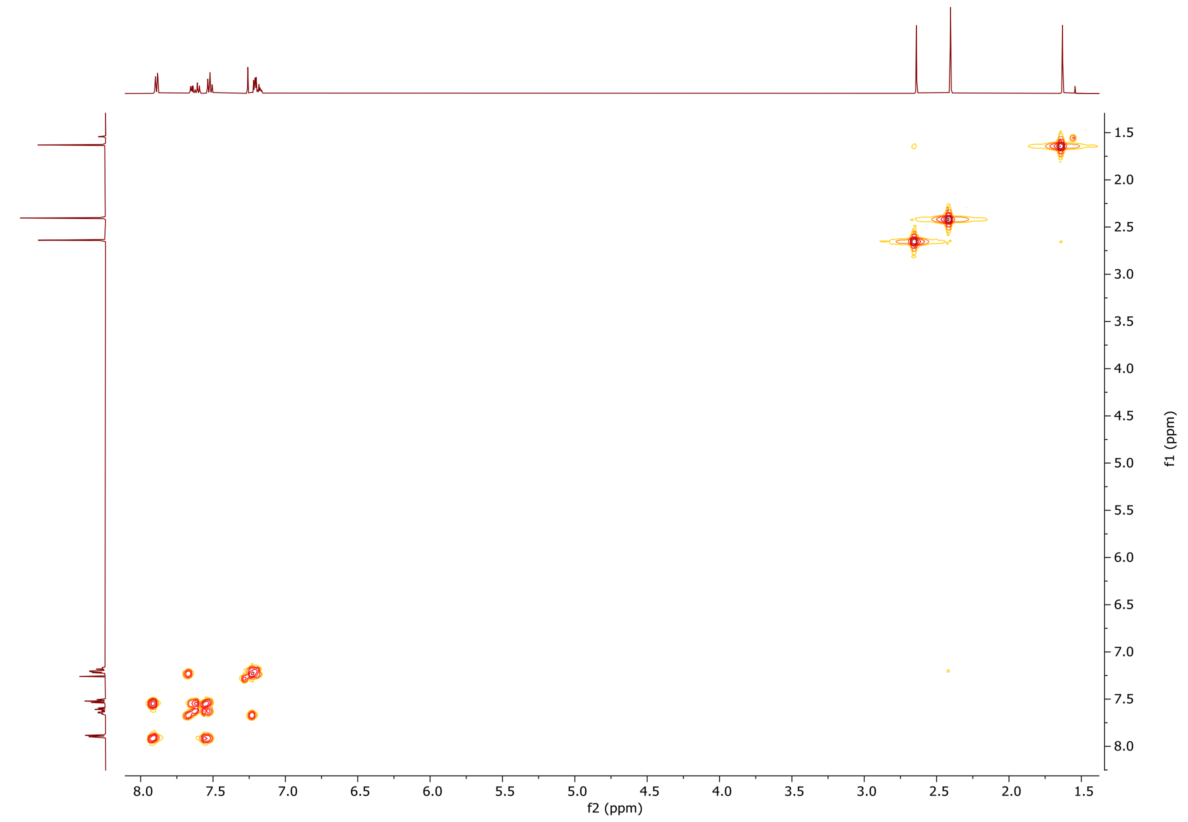

^{1}H/^{13}C HSQC (500/126 MHz, Chloroform-*d*)

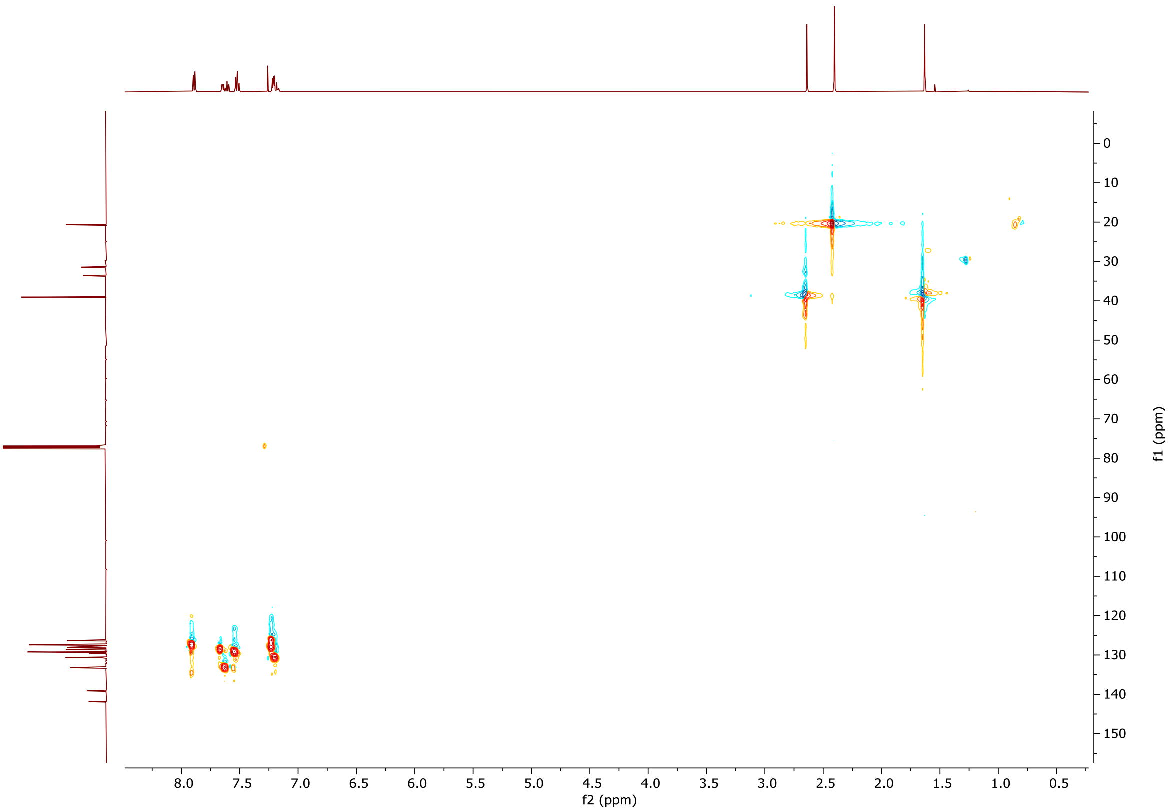

$^{1}H/^{13}C$ HMBC (500/126 MHz, Chloroform-*d*)

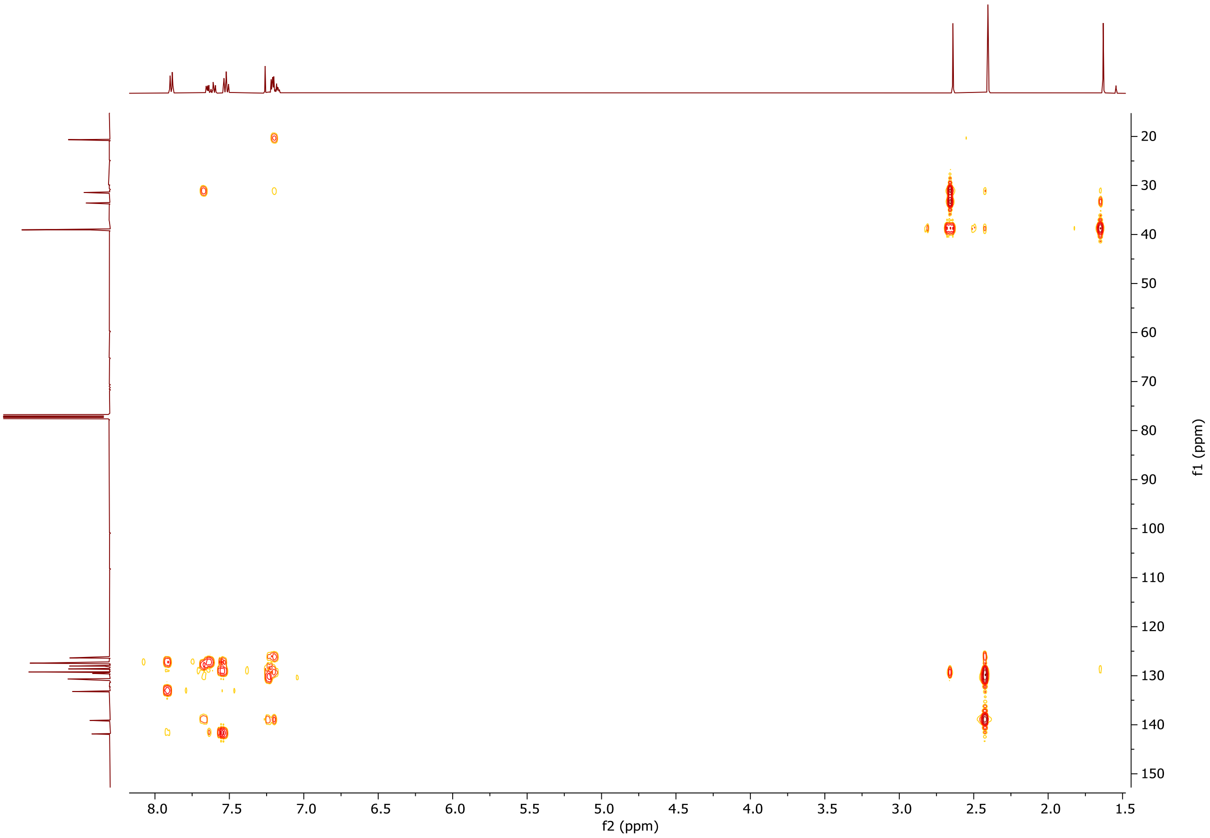

2-(3-(phenylsulfonyl)bicyclo[1.1.0]butan-1-yl)benzonitrile, 25

^{1}H NMR (500 MHz, Chloroform-*d*)

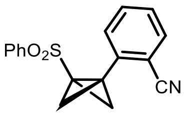

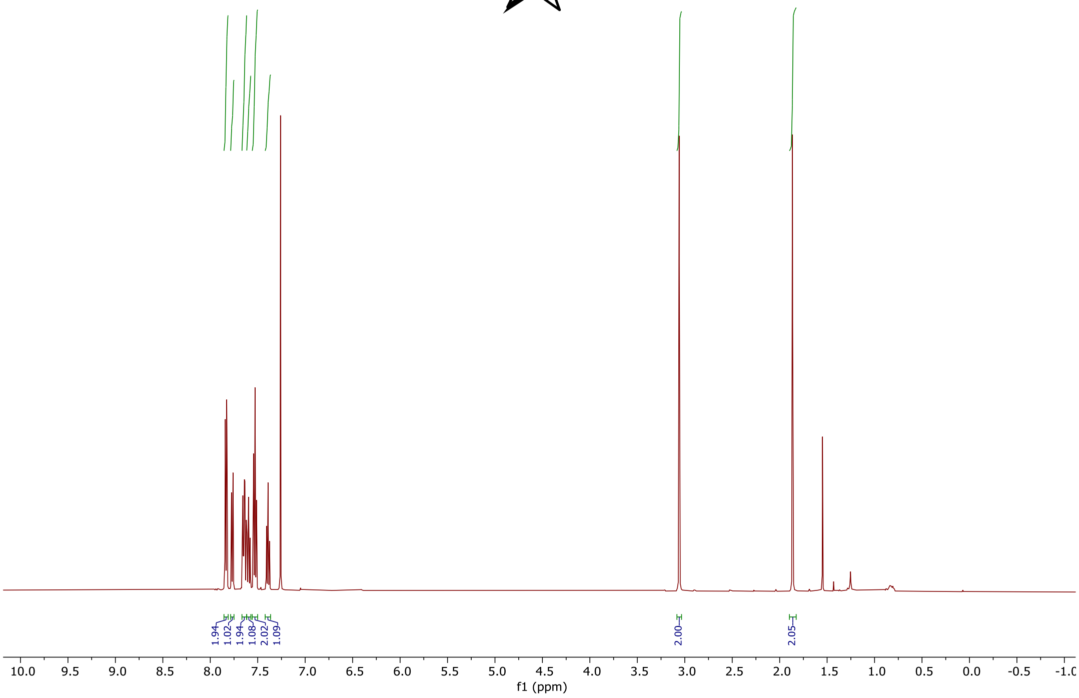

^{13}C NMR (126 MHz, Chloroform-*d*)

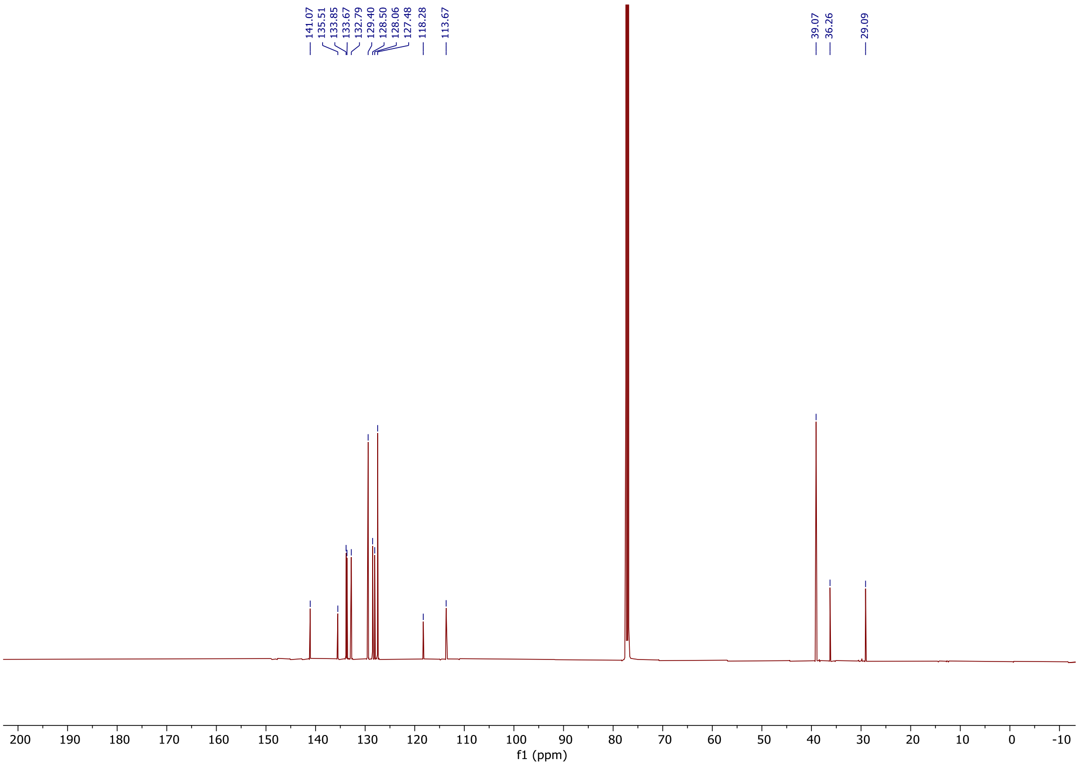

^{1}H COSY (500 MHz, Chloroform-*d*)

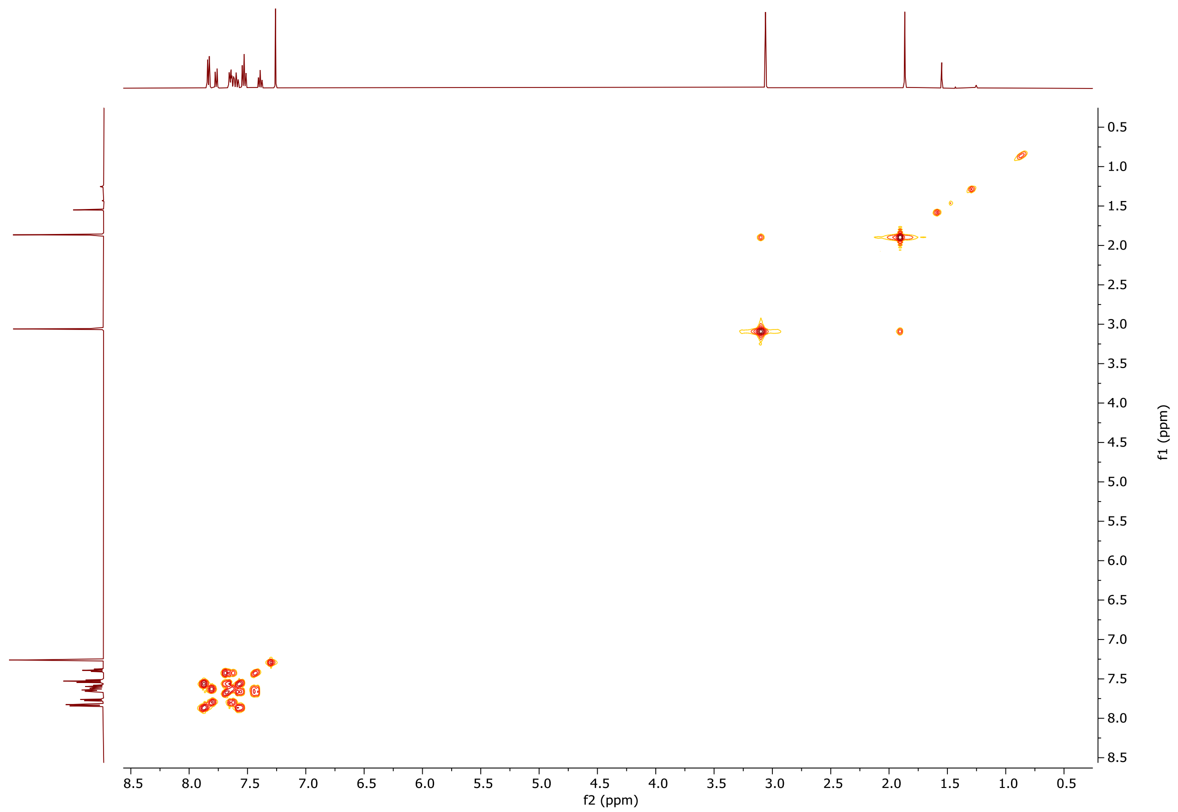

$^{1}H/^{13}C$ HSQC (500/126 MHz, Chloroform-*d*)

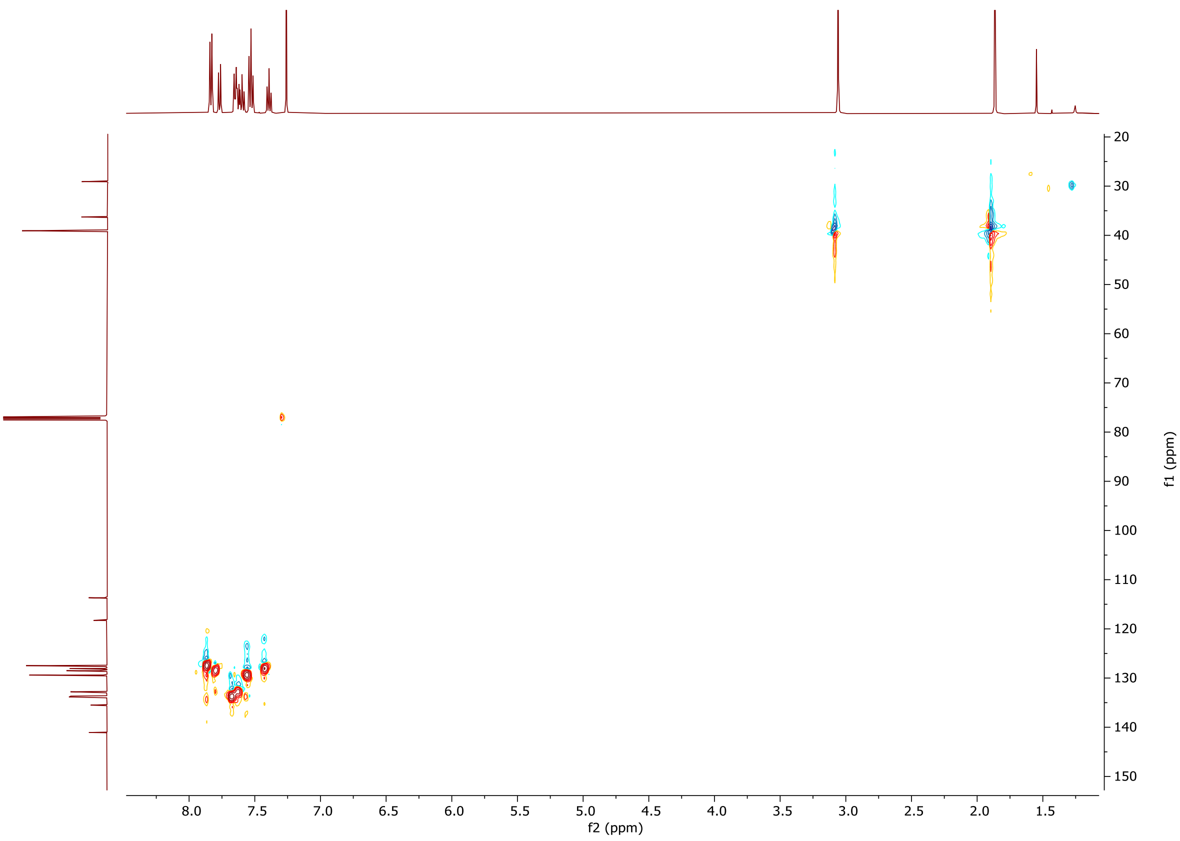

^{1}H/^{13}C HMBC (500/126 MHz, Chloroform-*d*)

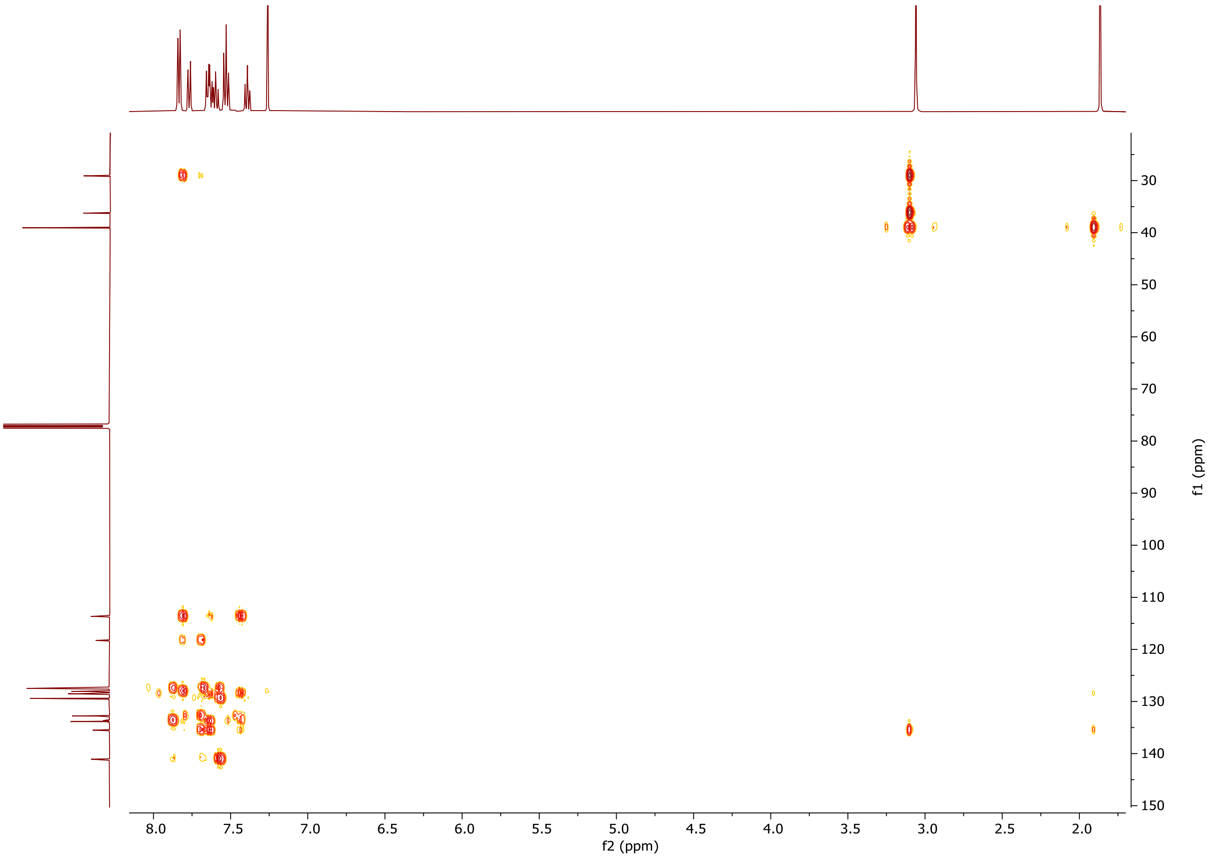

1-(4-bromo-2-fluorophenyl)-3-(phenylsulfonyl)bicyclo[1.1.0]butane, 26

^{1}H NMR (500 MHz, Benzene-*d*)

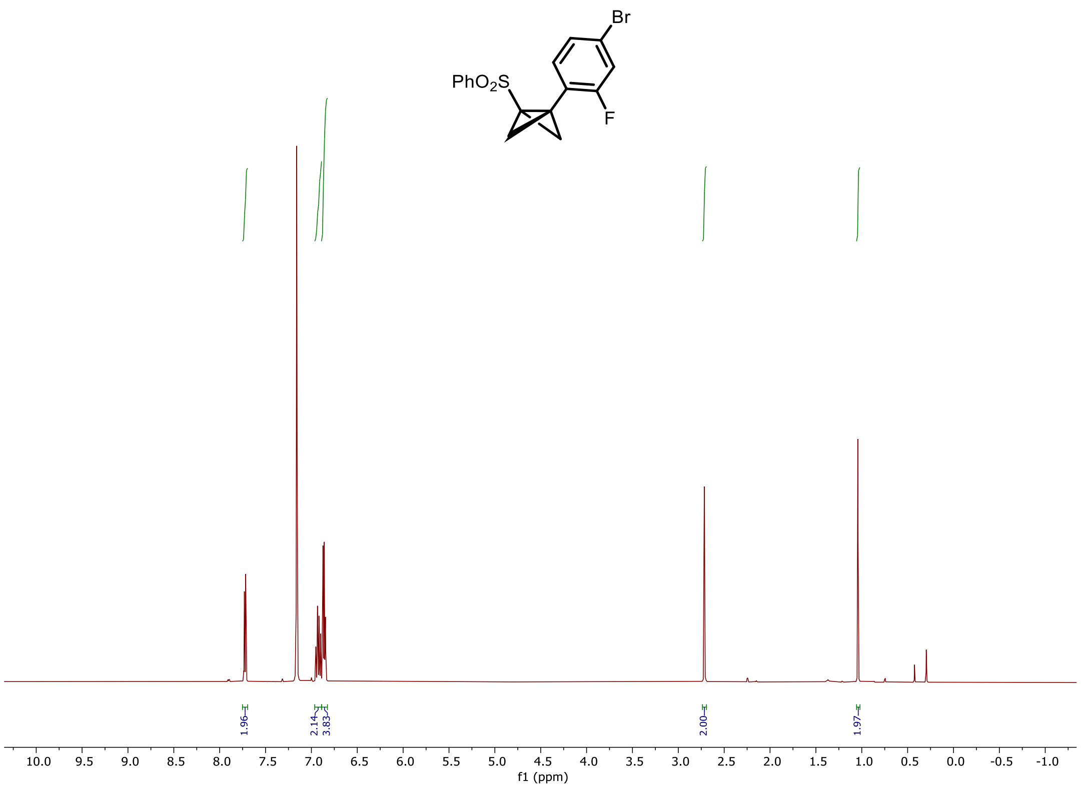

^{13}C NMR (126 MHz, Benzene-*d*)

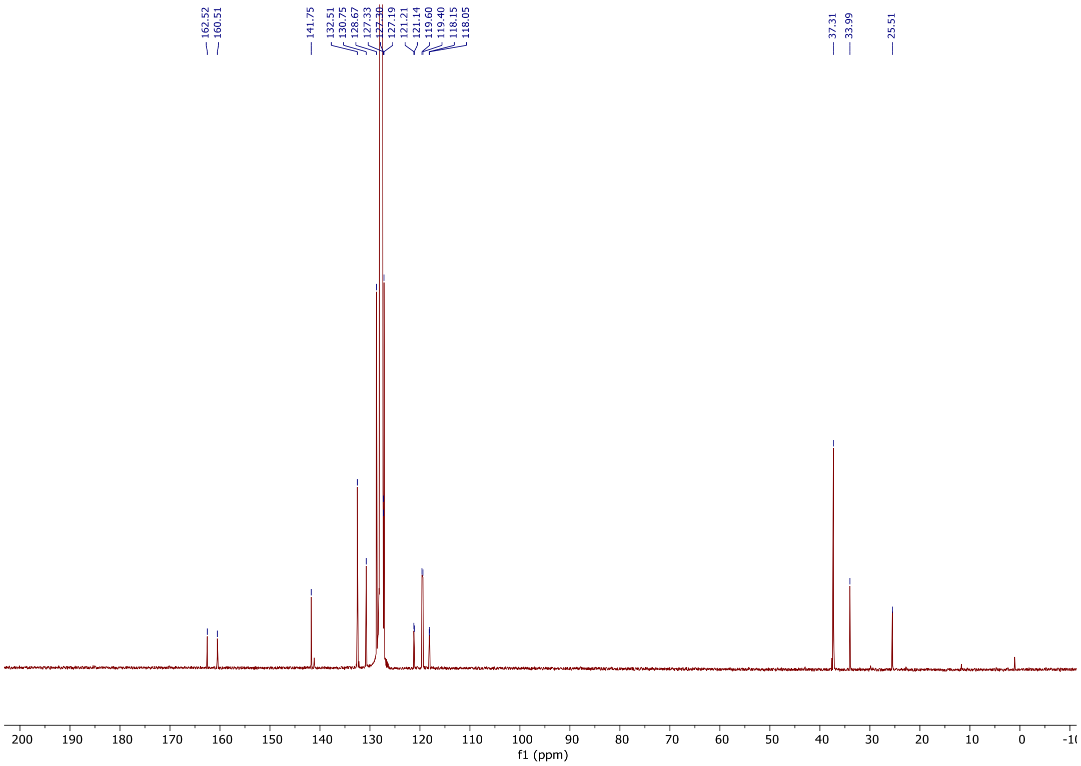

^{1}H COSY (500 MHz, Benzene-*d*)

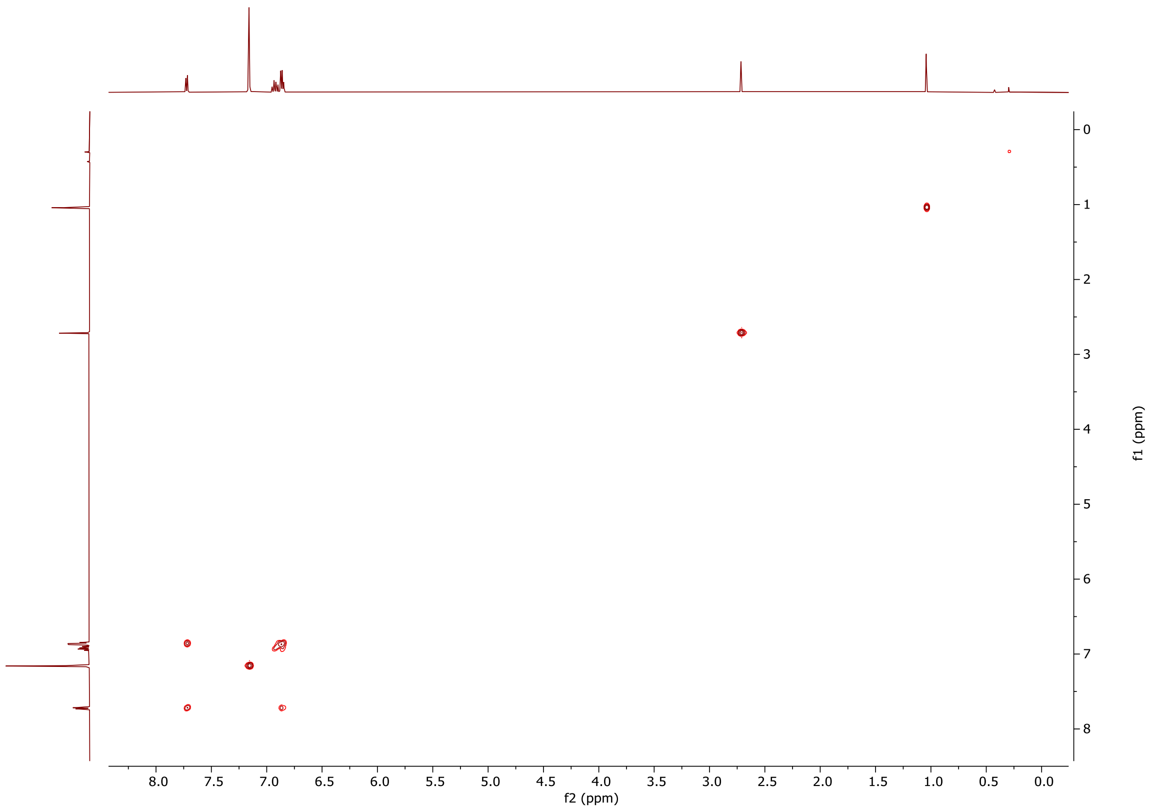

^{1}H/^{13}C HSQC (500/126 MHz, Benzene-*d*)

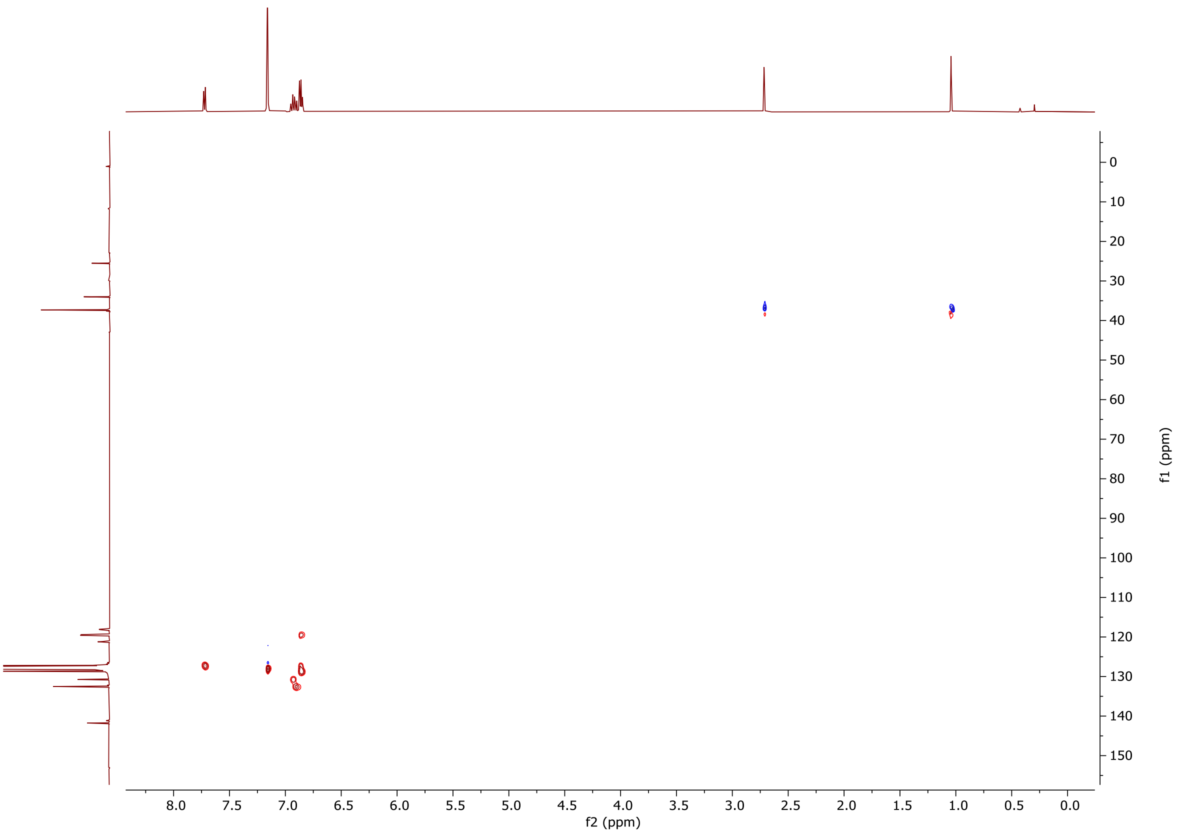

^{1}H/^{13}C HMBC (500/126 MHz, Benzene-*d*)

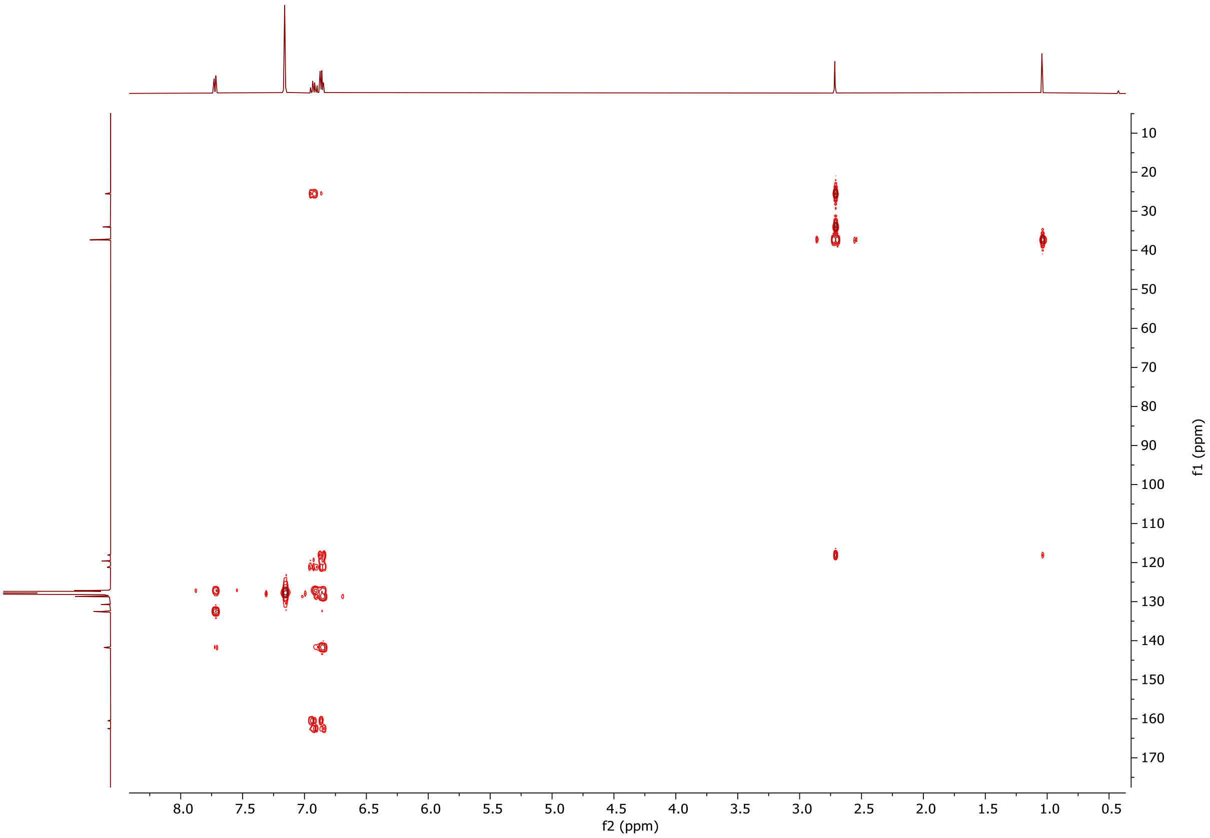

^{19}F NMR (470 MHz, Benzene-*d*)

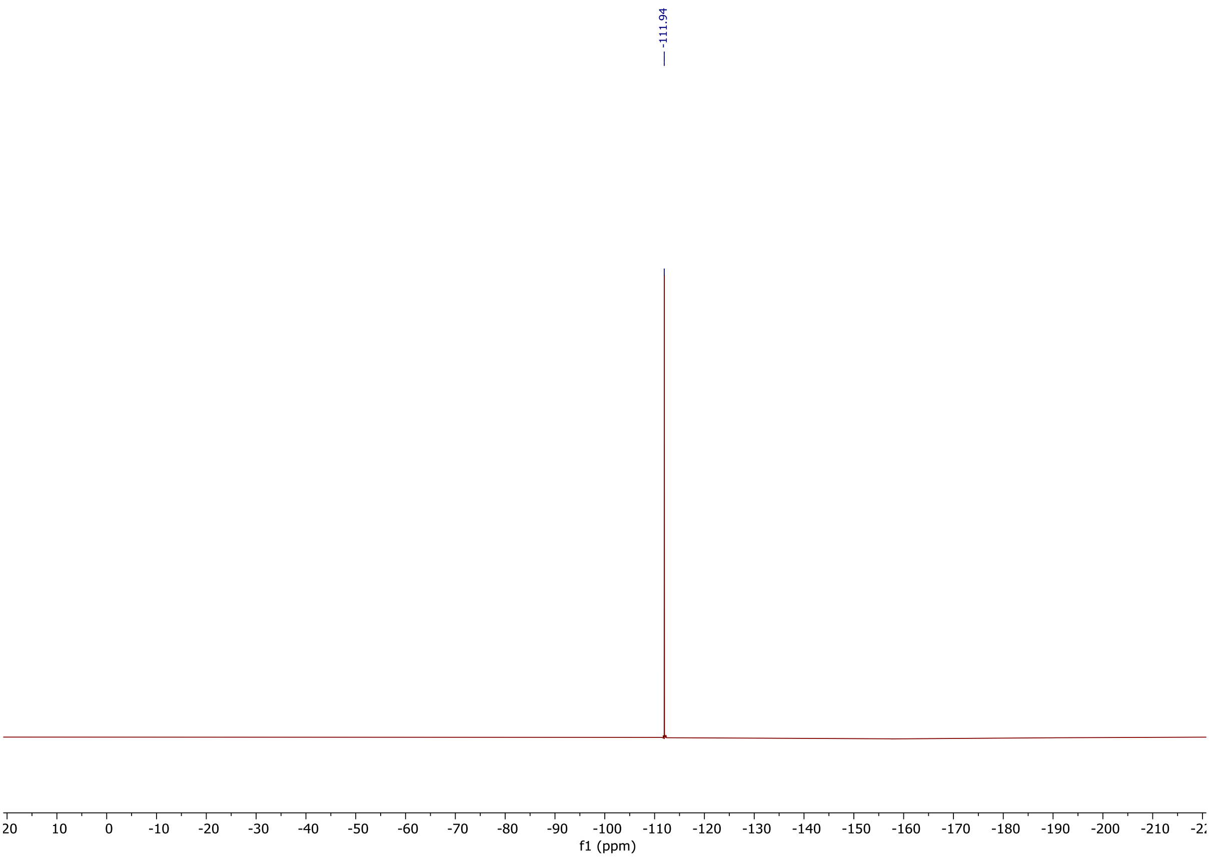

1-(3-(phenylsulfonyl)bicyclo[1.1.0]butan-1-yl)naphthalene, 27

^{1}H NMR (500 MHz, Chloroform-*d*)

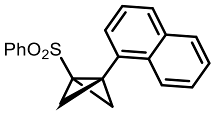

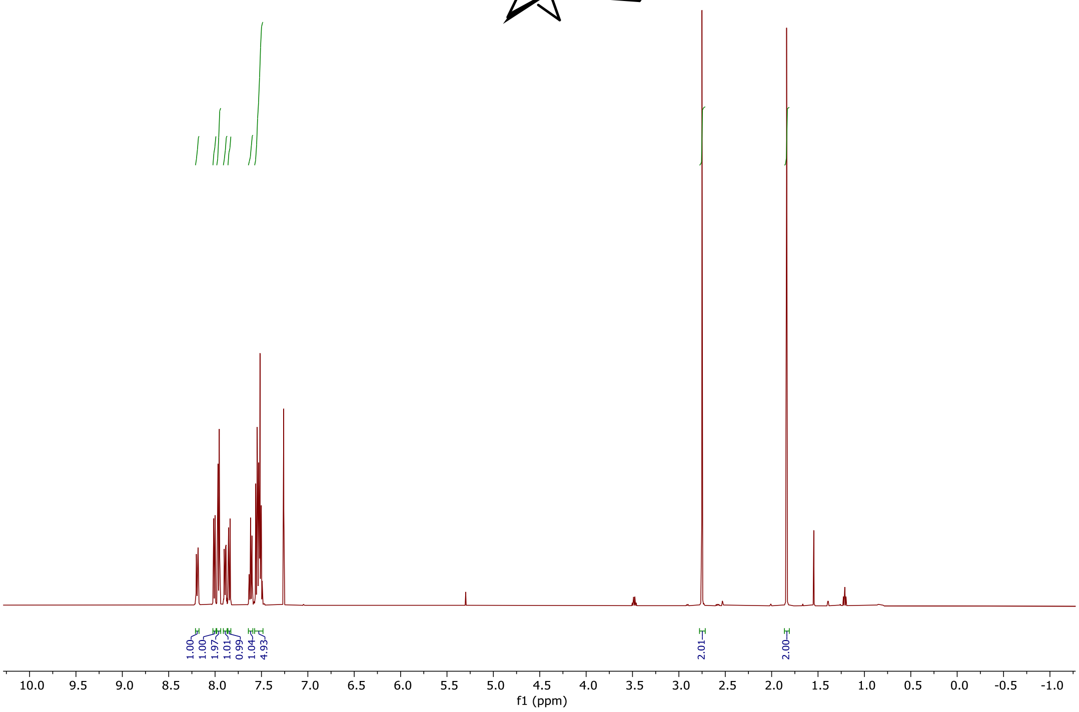

^{13}C NMR (126 MHz, Chloroform-*d*)

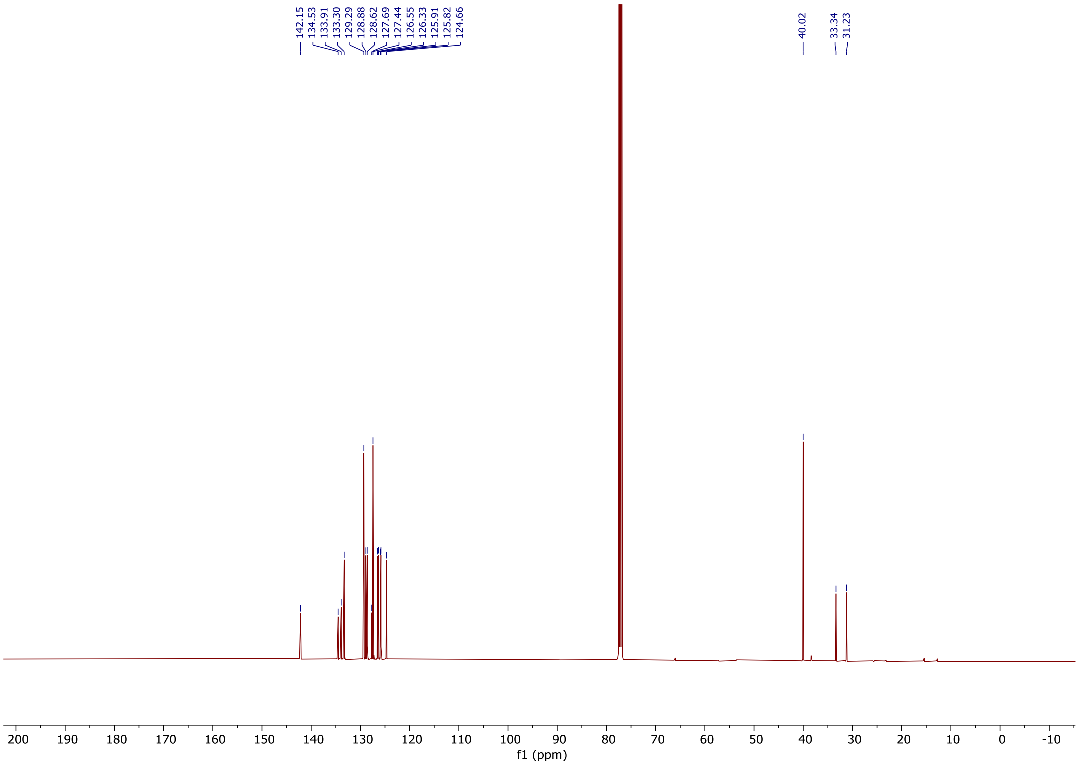

^{1}H COSY (500 MHz, Chloroform-*d*)

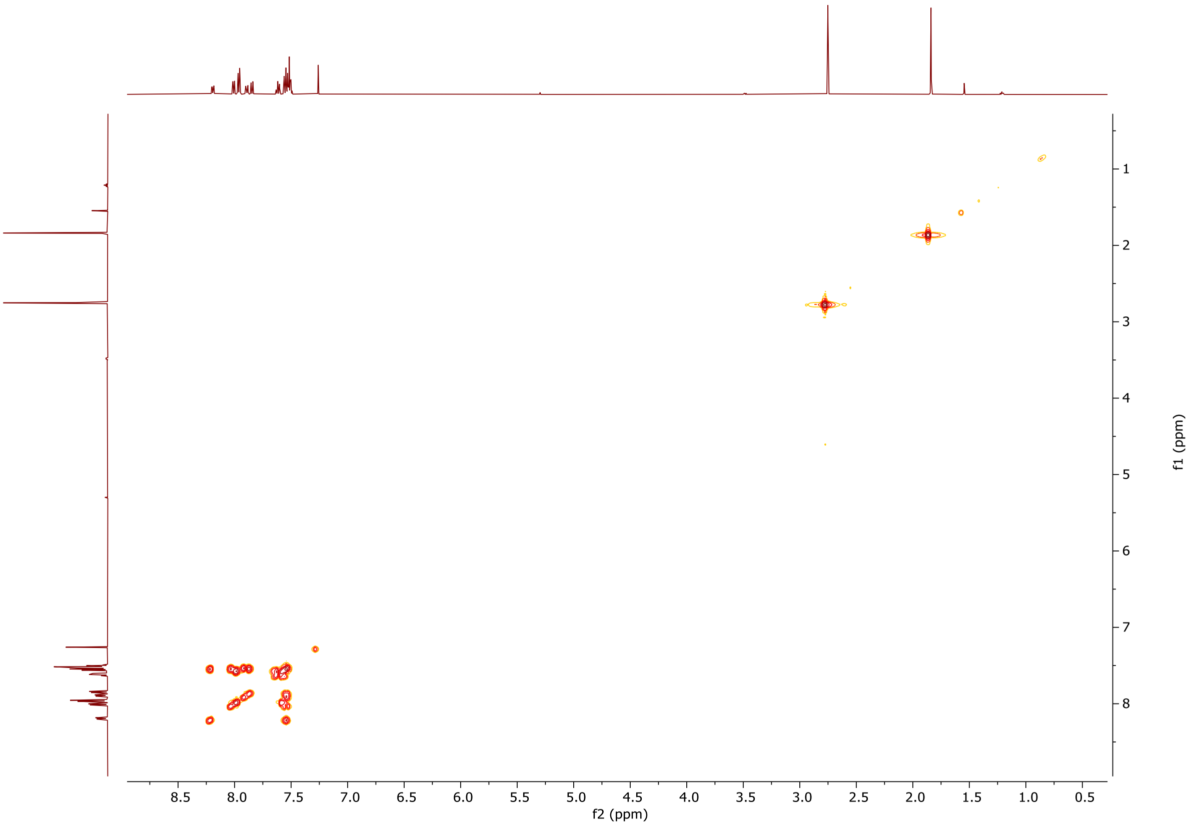

^{1}H/^{13}C HSQC (500/126 MHz, Chloroform-*d*)

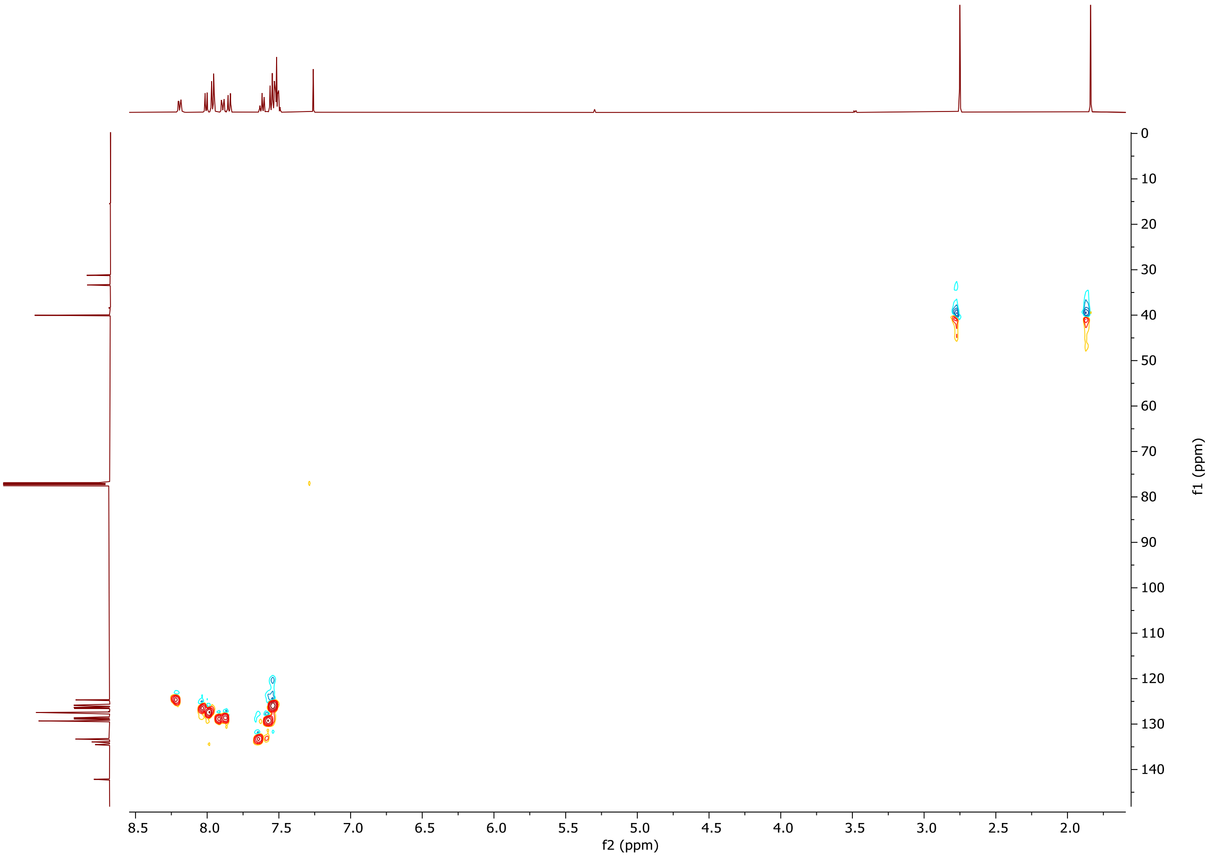

$^1H/^{13}C$ HMBC (500/126 MHz, Chloroform-*d*)

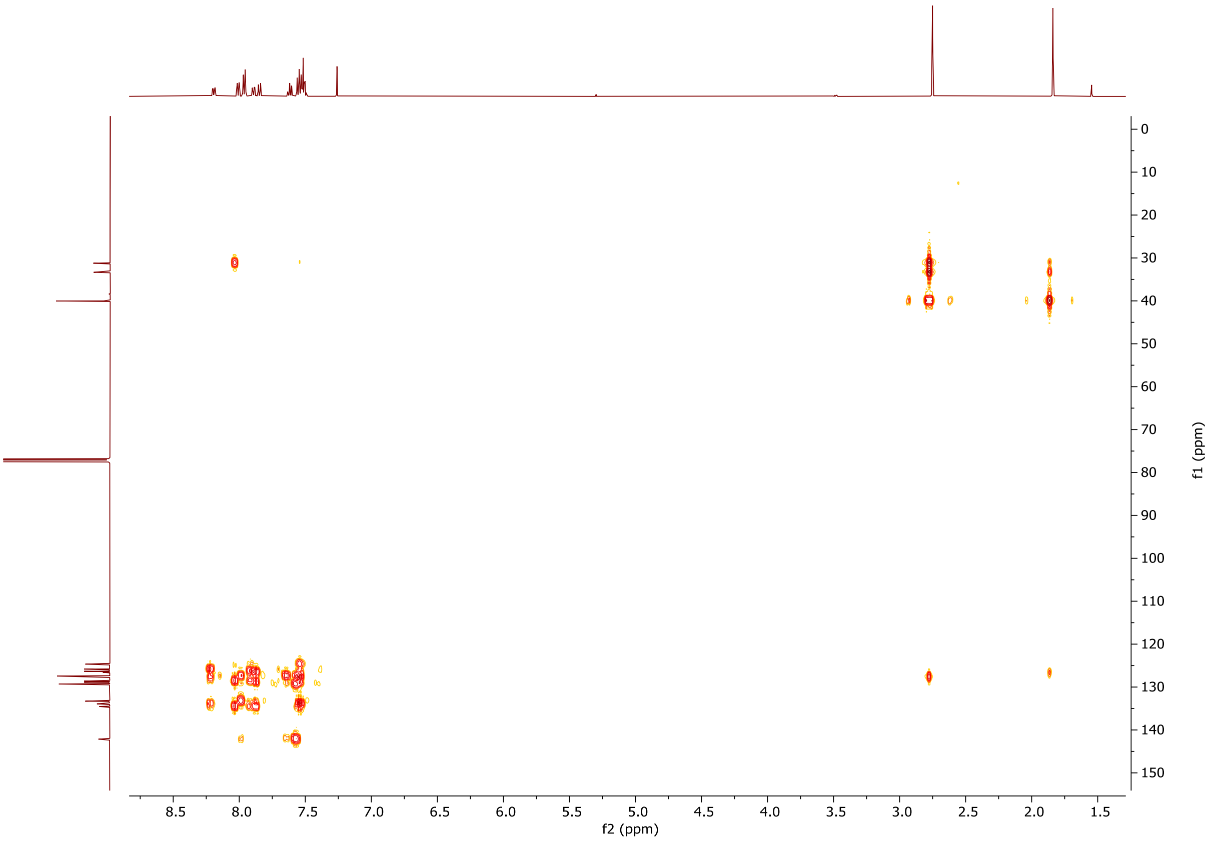

Ethyl (Z)-3-(3-(phenylsulfonyl)bicyclo[1.1.0]butan-1-yl)acrylate, 28

^{1}H NMR (500 MHz, Chloroform-*d*)

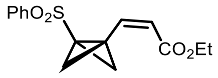

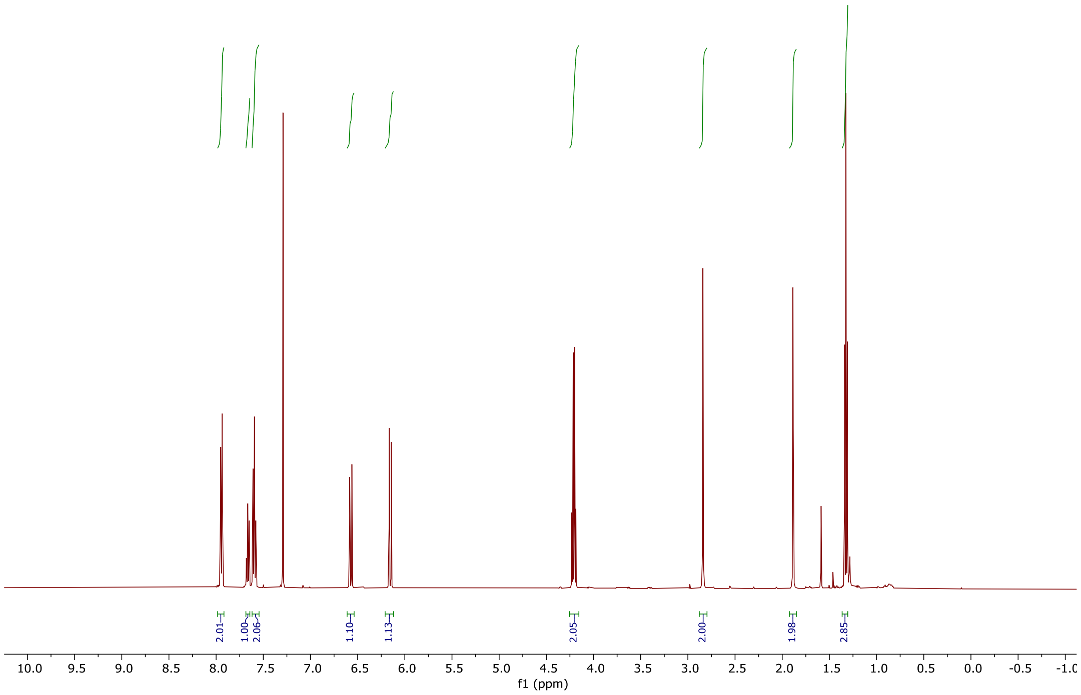

^{13}C NMR (126 MHz, Chloroform-*d*)

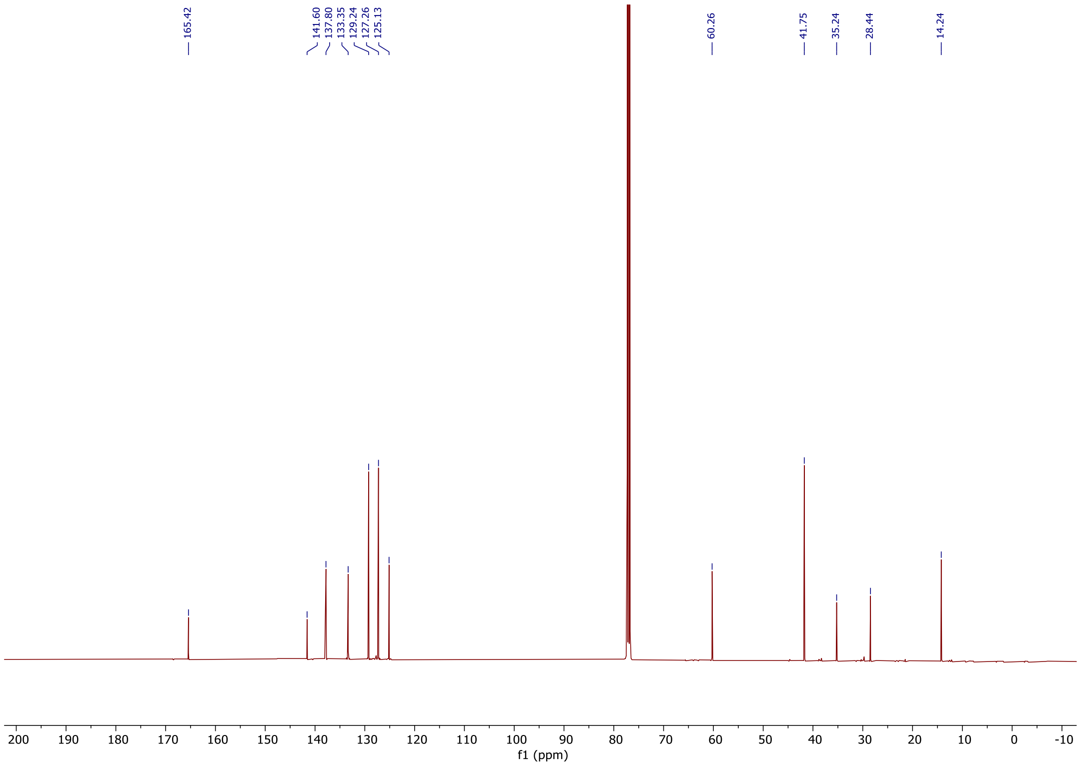

^{1}H COSY (500 MHz, Chloroform-*d*)

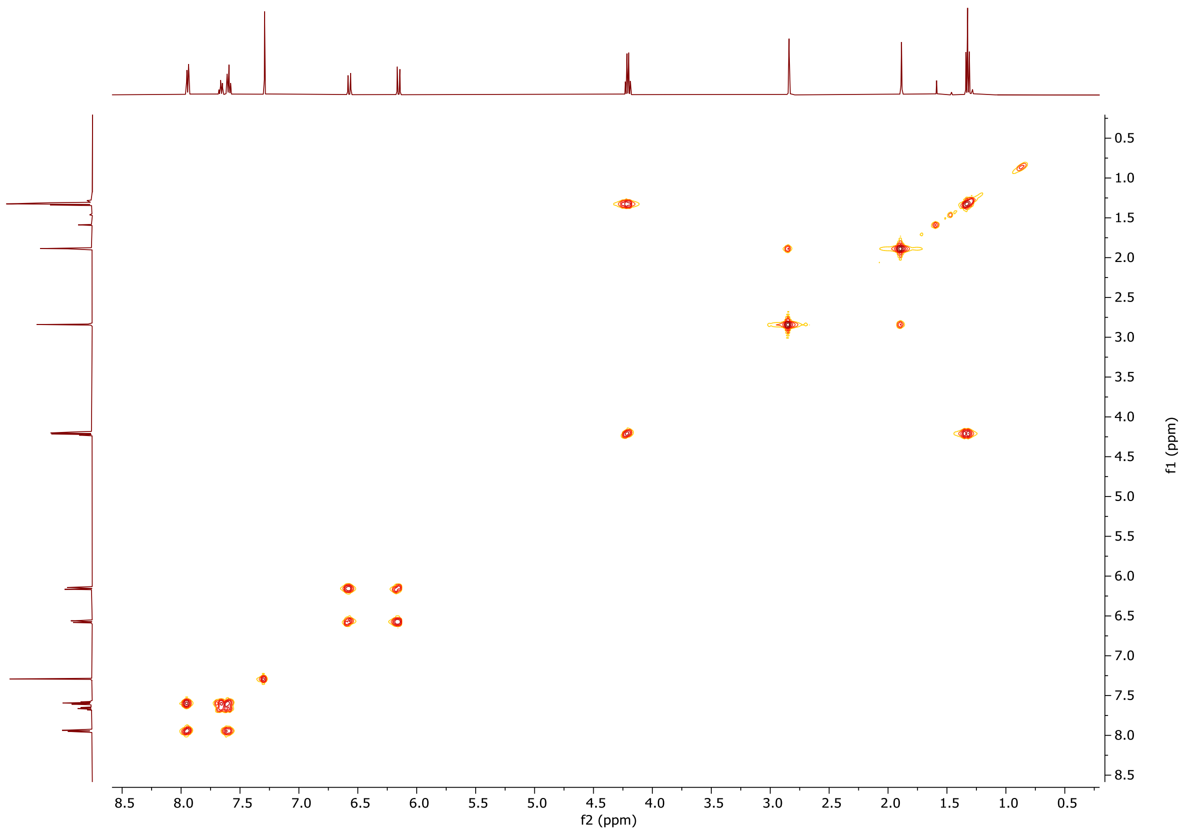

^{1}H/^{13}C HSQC (500/126 MHz, Chloroform-*d*)

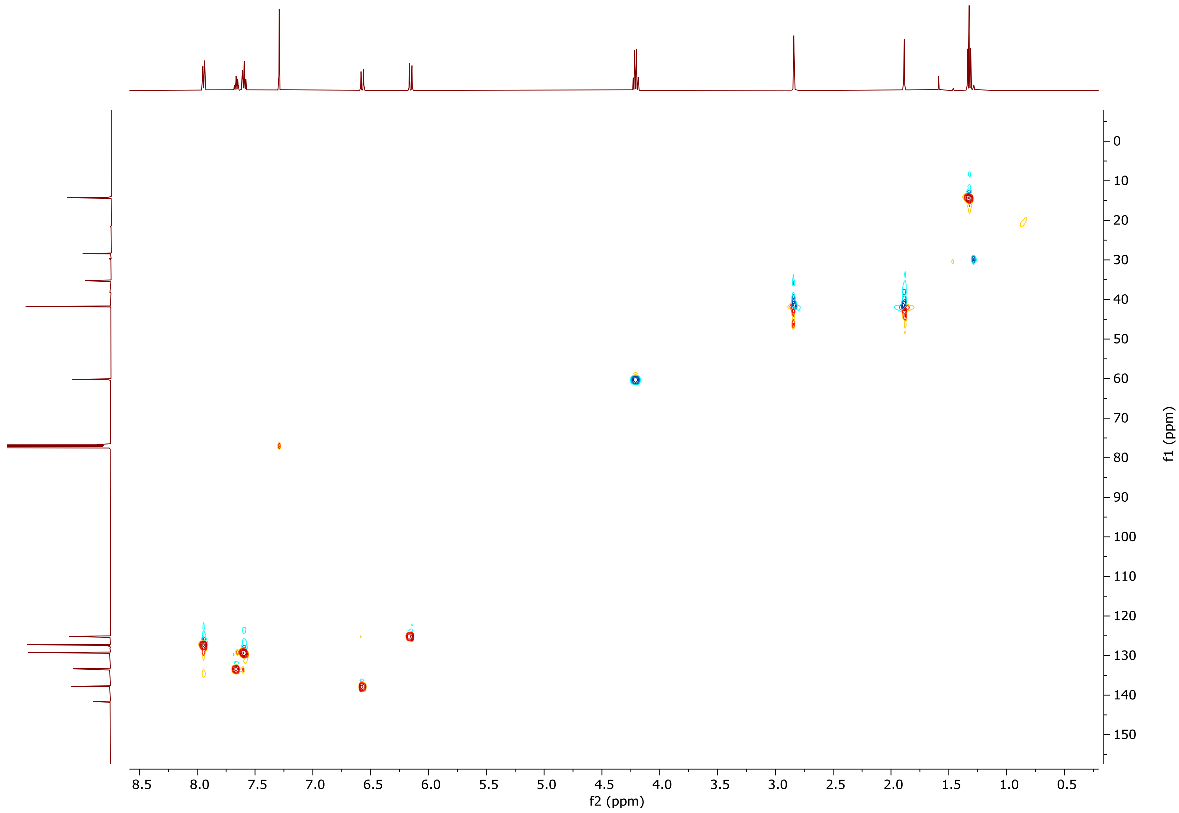

$^{1}H/^{13}C$ HMBC (500/126 MHz, Chloroform-*d*)

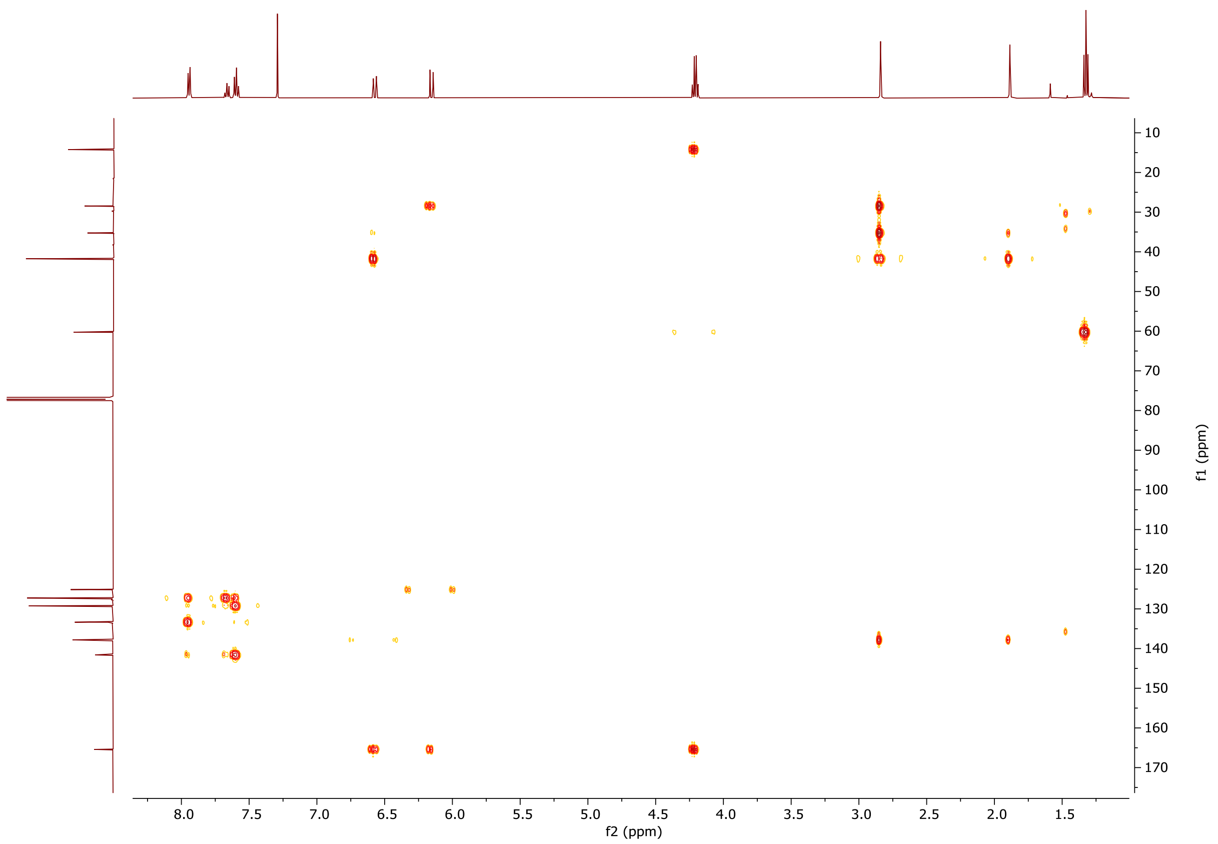

(*E*)-1-(oct-1-en-1-yl)-3-(phenylsulfonyl)bicyclo[1.1.0]butane, (*E*)-29

^{1}H NMR (500 MHz, Benzene-*d*)

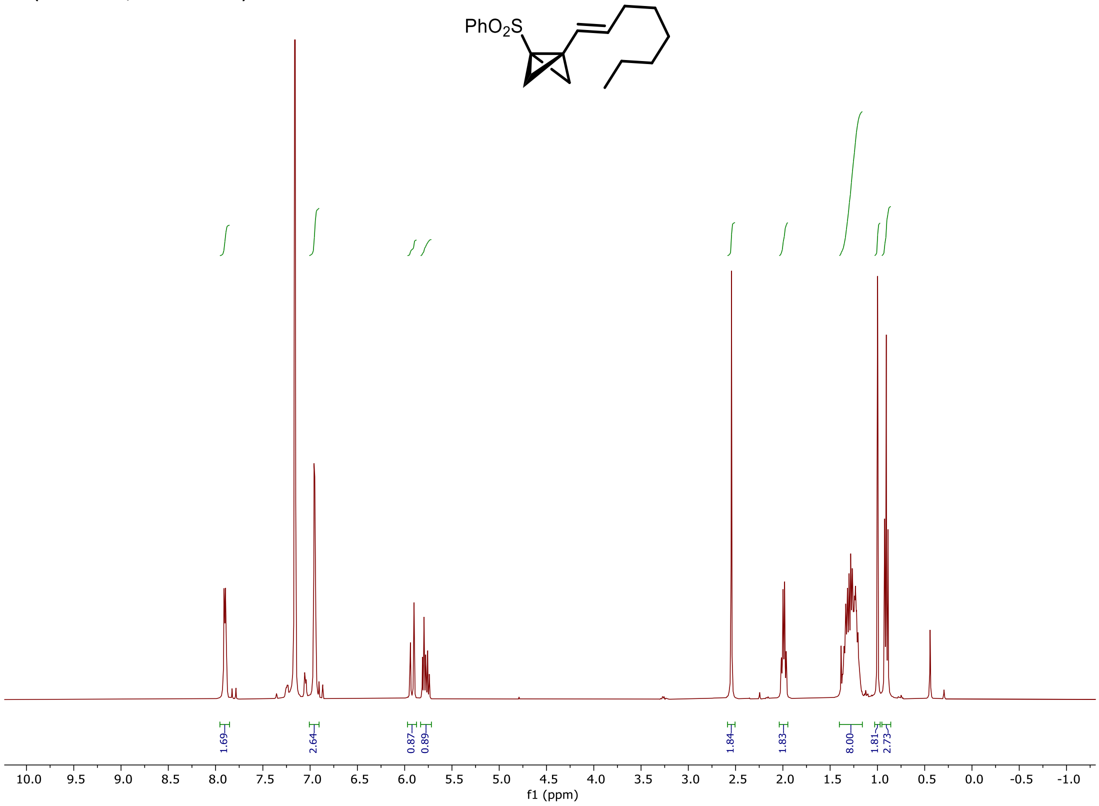

^{13}C NMR (126 MHz, Benzene-*d*)

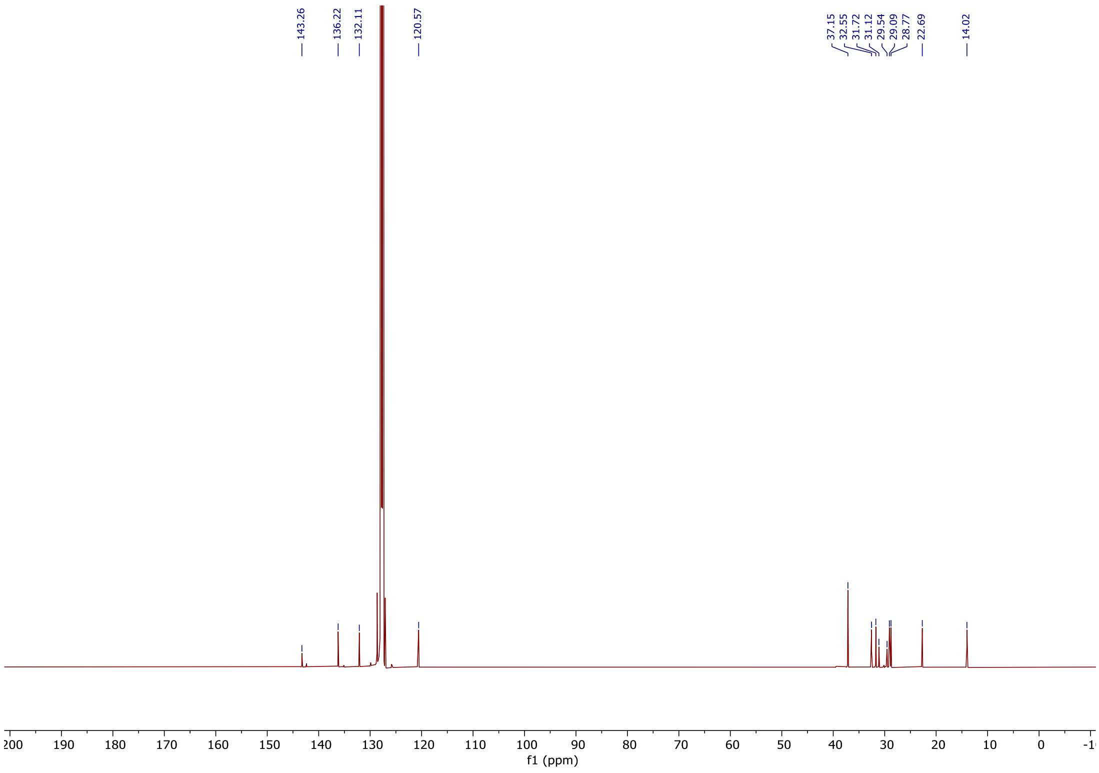

^{1}H COSY (500 MHz, Benzene-*d*)

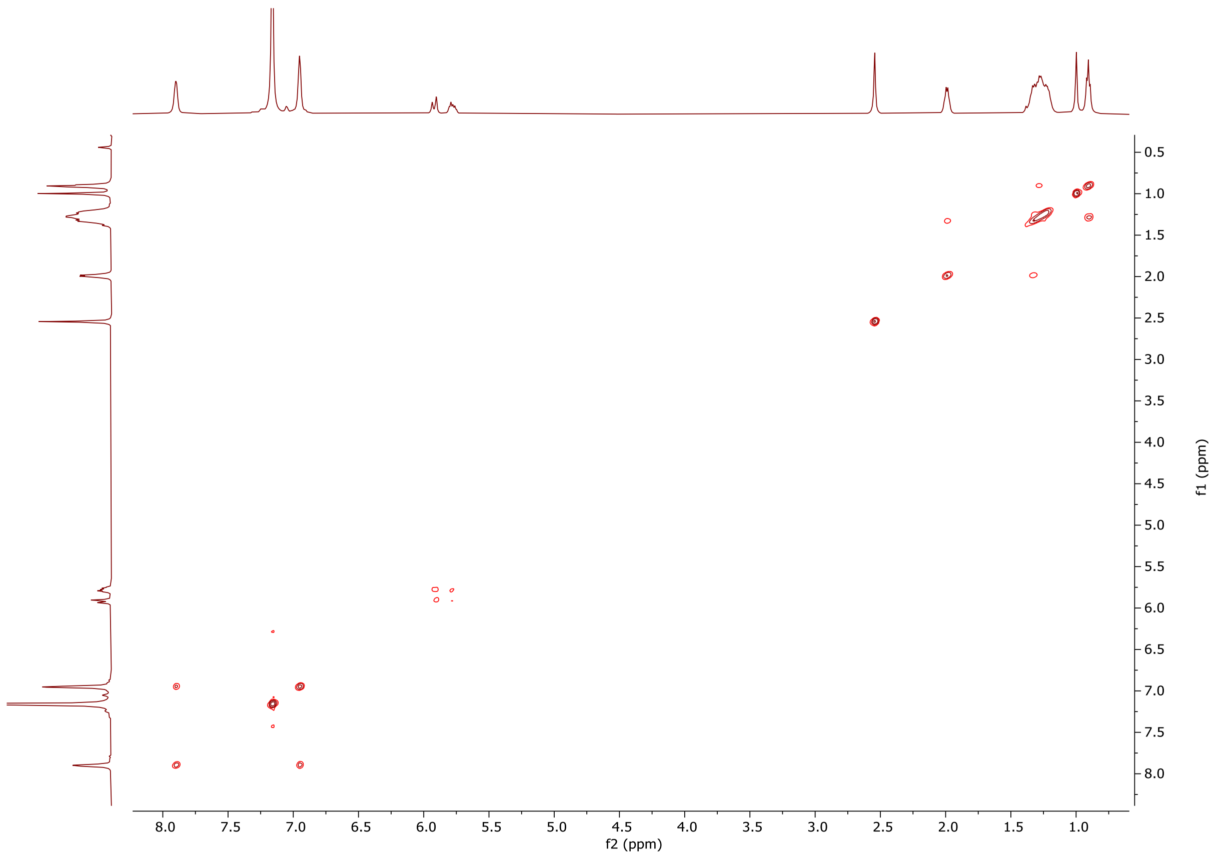

$^{1}H/^{13}C$ HSQC (500/126 MHz, Benzene-*d*)

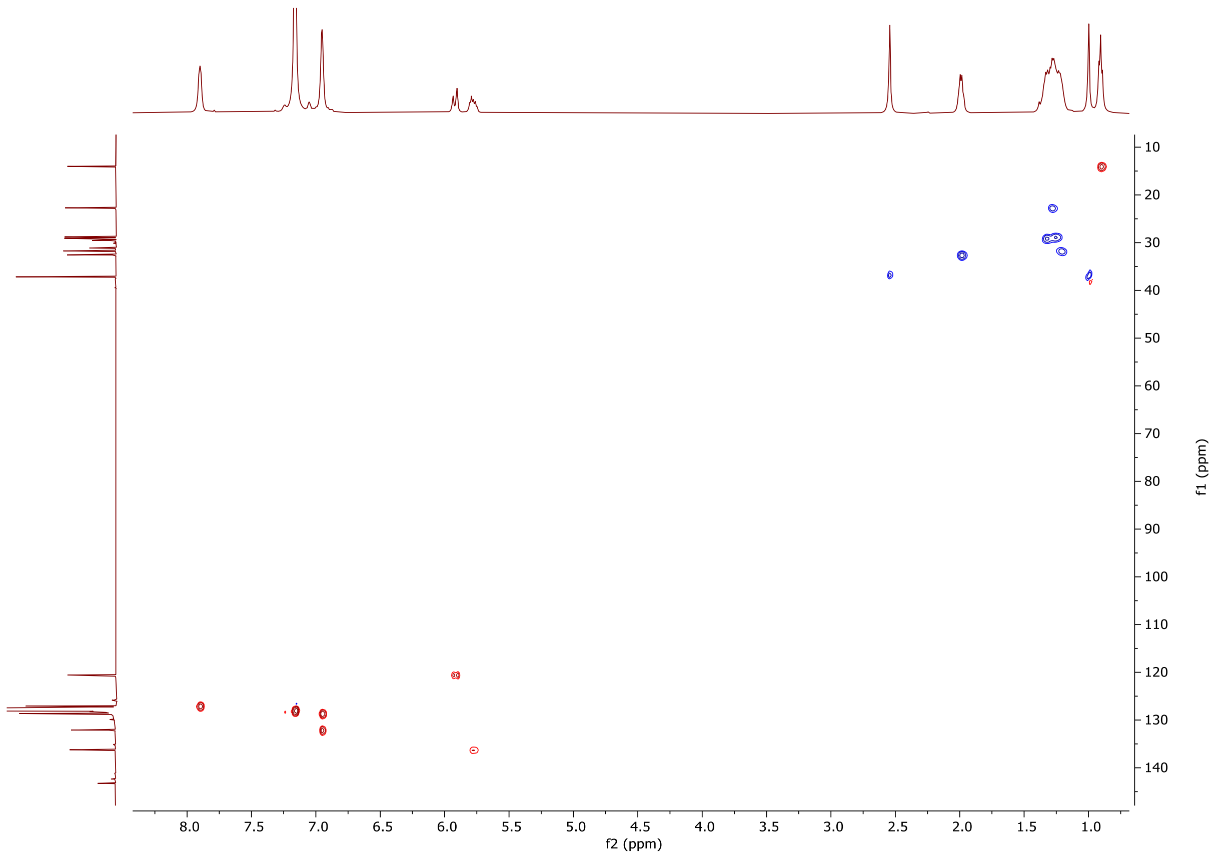

¹H/¹³C HMBC (500/126 MHz, Benzene-*d*)

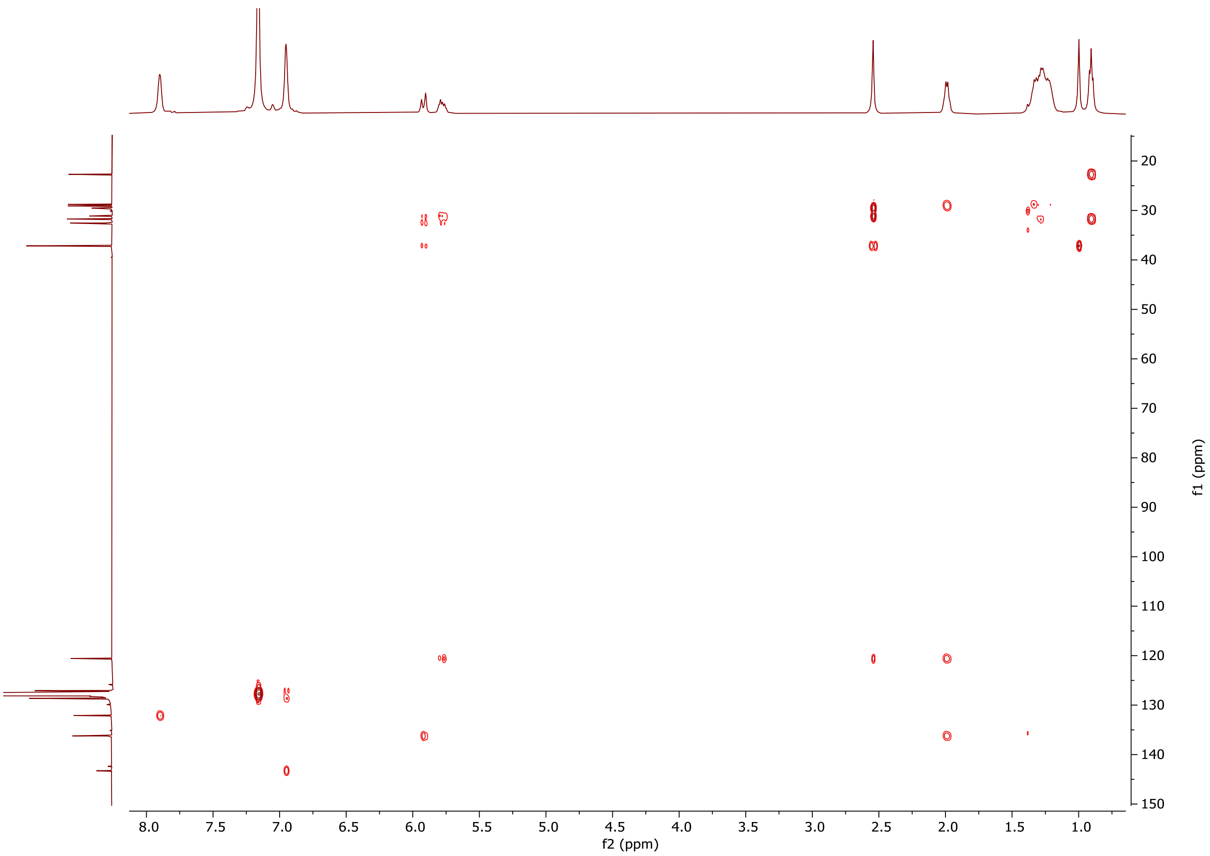

(*Z*)-1-(oct-1-en-1-yl)-3-(phenylsulfonyl)bicyclo[1.1.0]butane, (*Z*)-29

¹H NMR (500 MHz, Benzene-*d*)

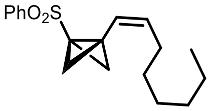

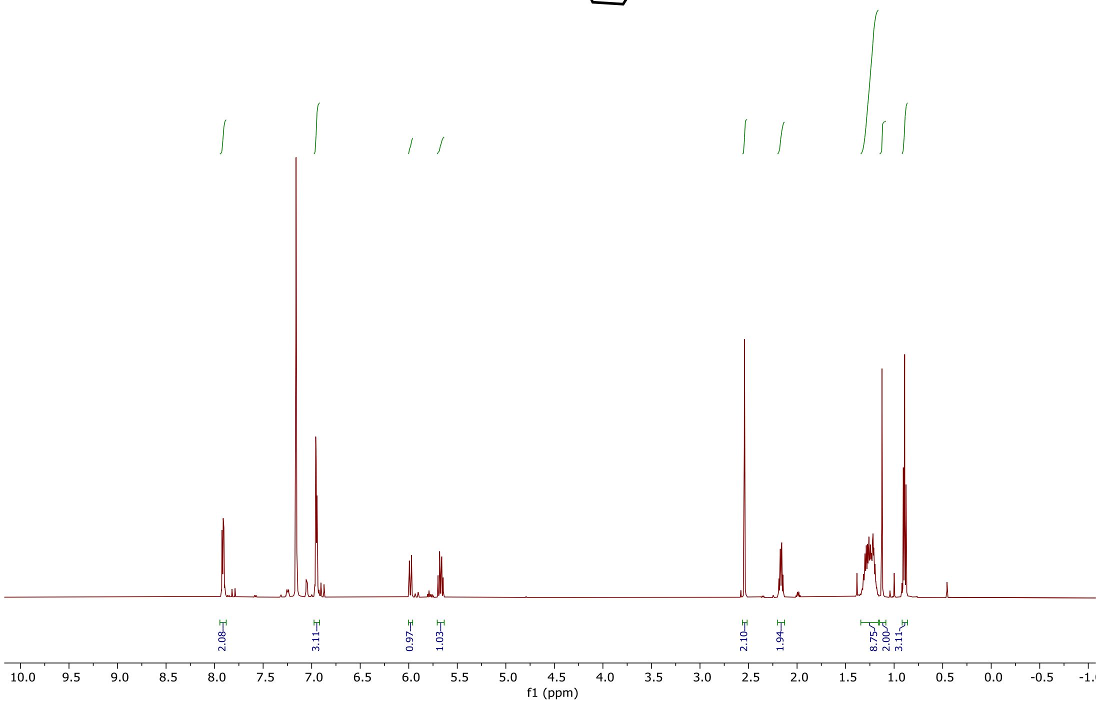

¹³C NMR (126 MHz, Benzene-*d*)

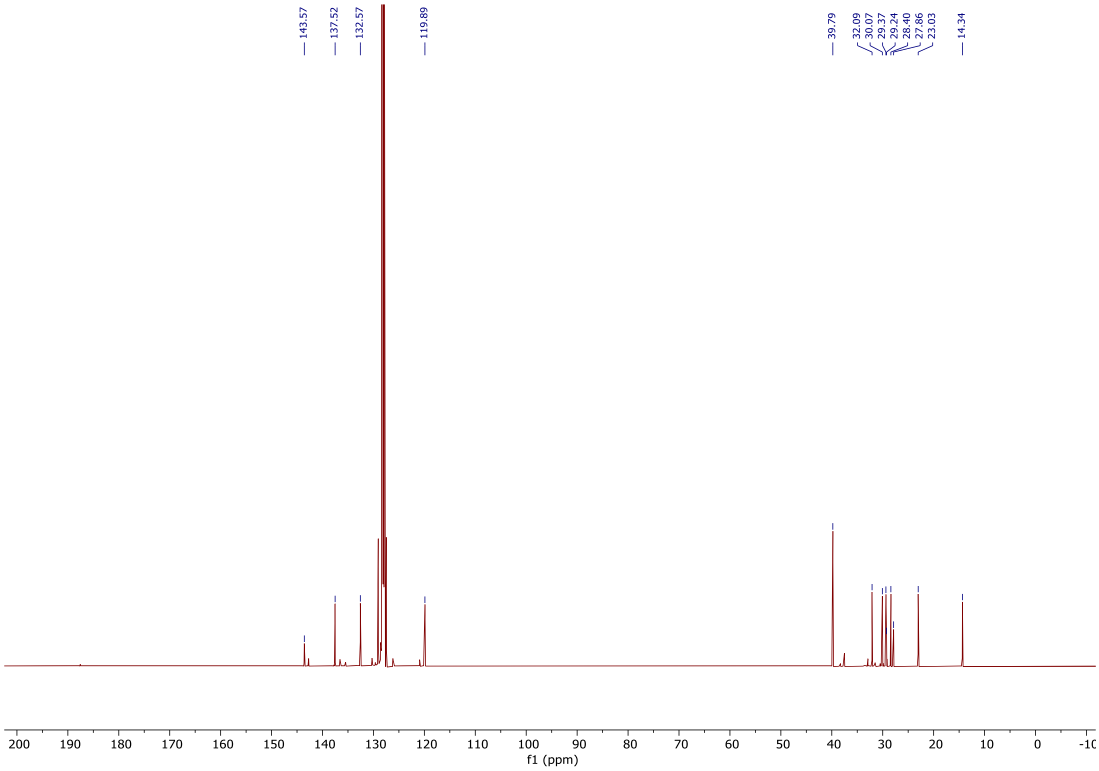

^{1}H COSY (500 MHz, Benzene-*d*)

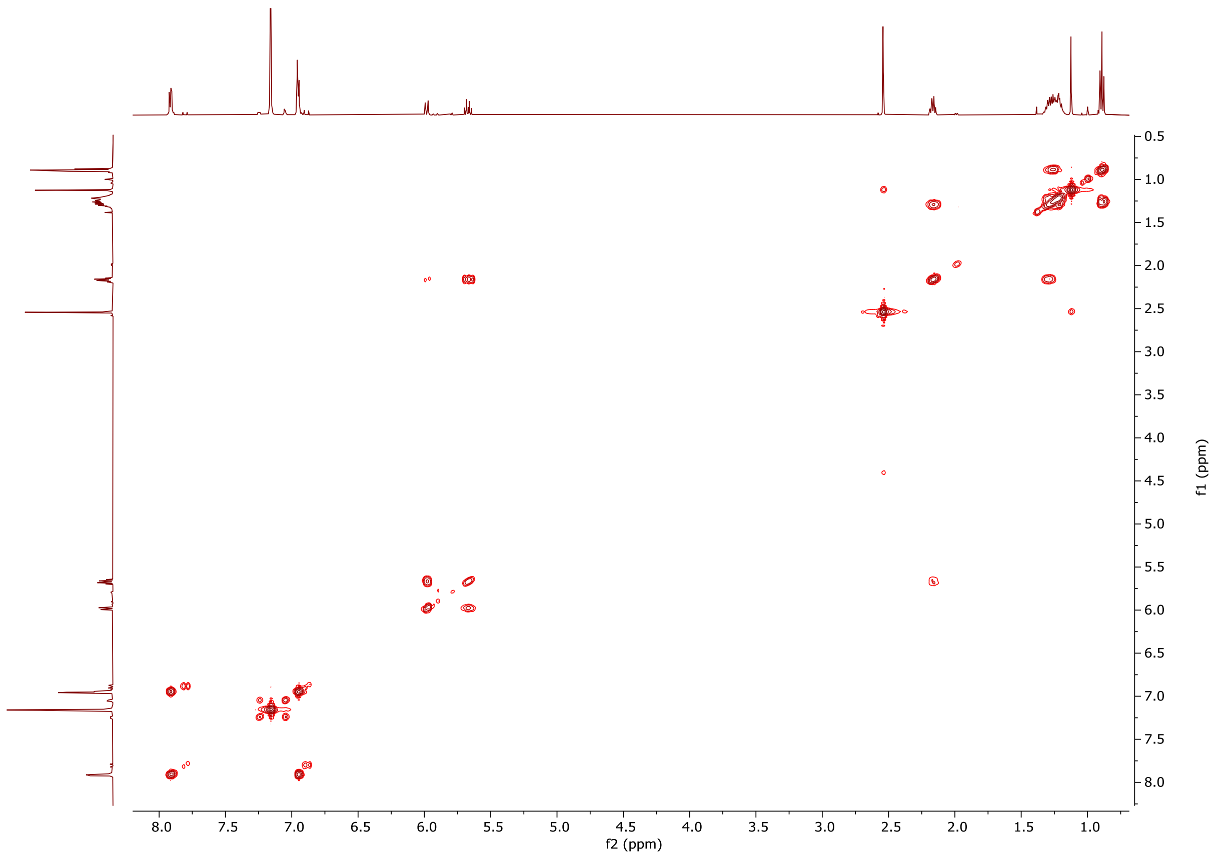

^{1}H/^{13}C HSQC (500/126 MHz, Benzene-*d*)

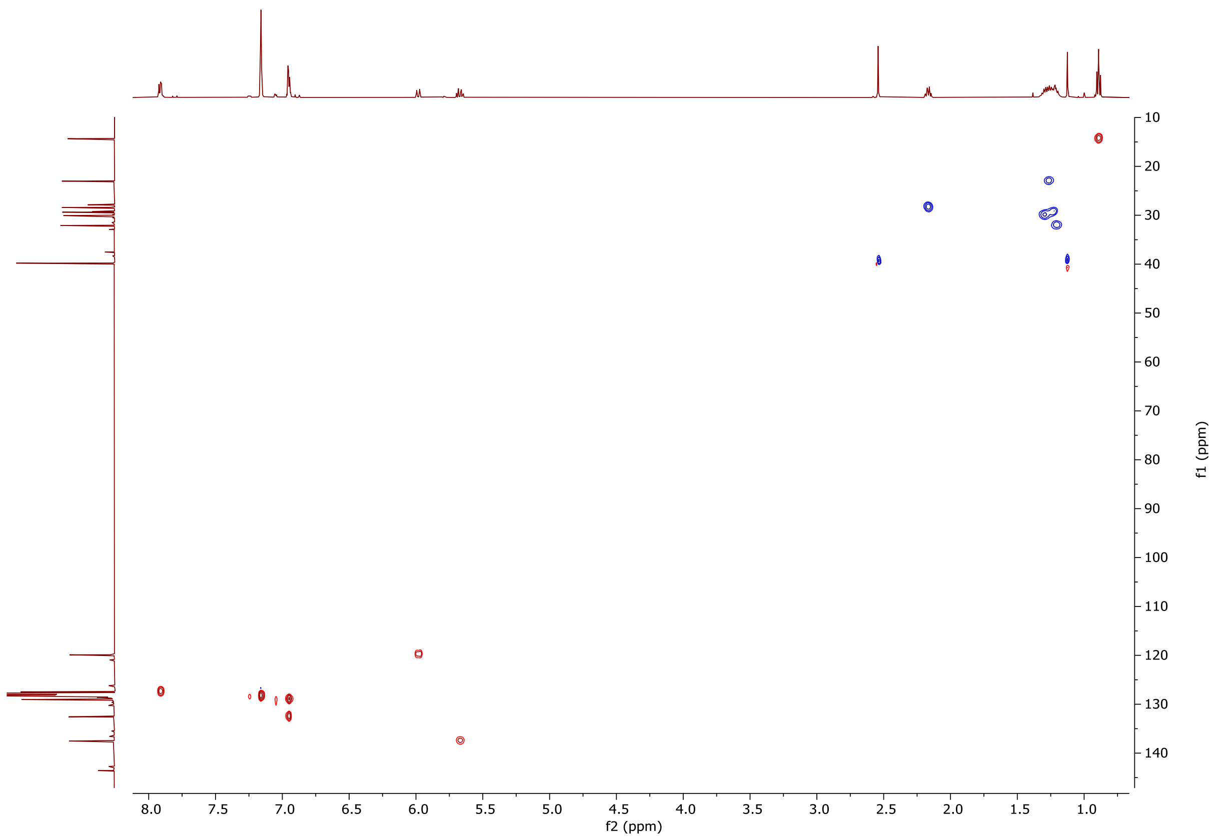

$^{1}H/^{13}C$ HMBC (500/126 MHz, Benzene-*d*)

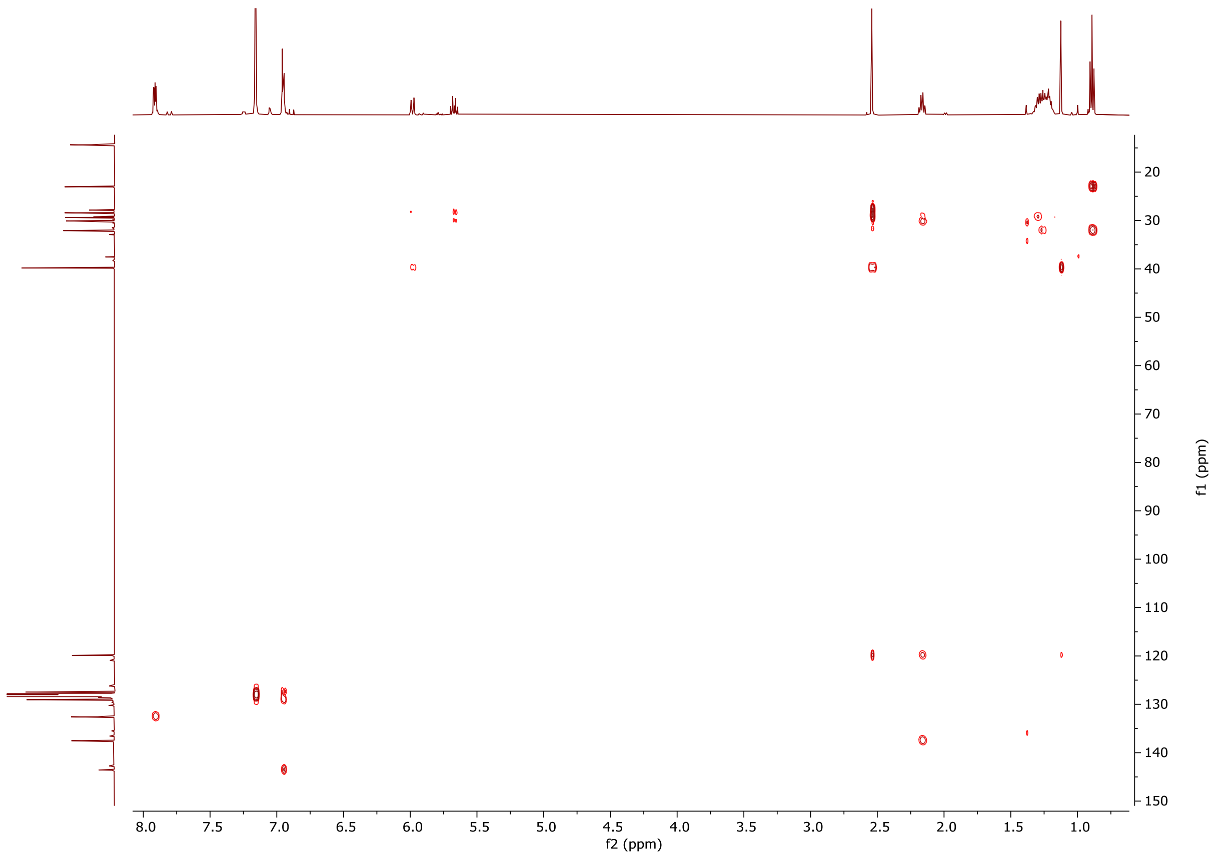

1,3-dimethyl-5-(3-(phenylsulfonyl)cyclobut-1-en-1-yl)pyrimidine-2,4(1H,3H)-dione, 30

^{1}H NMR (500 MHz, Chloroform-*d*)

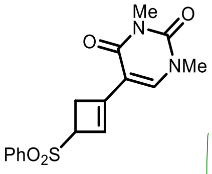

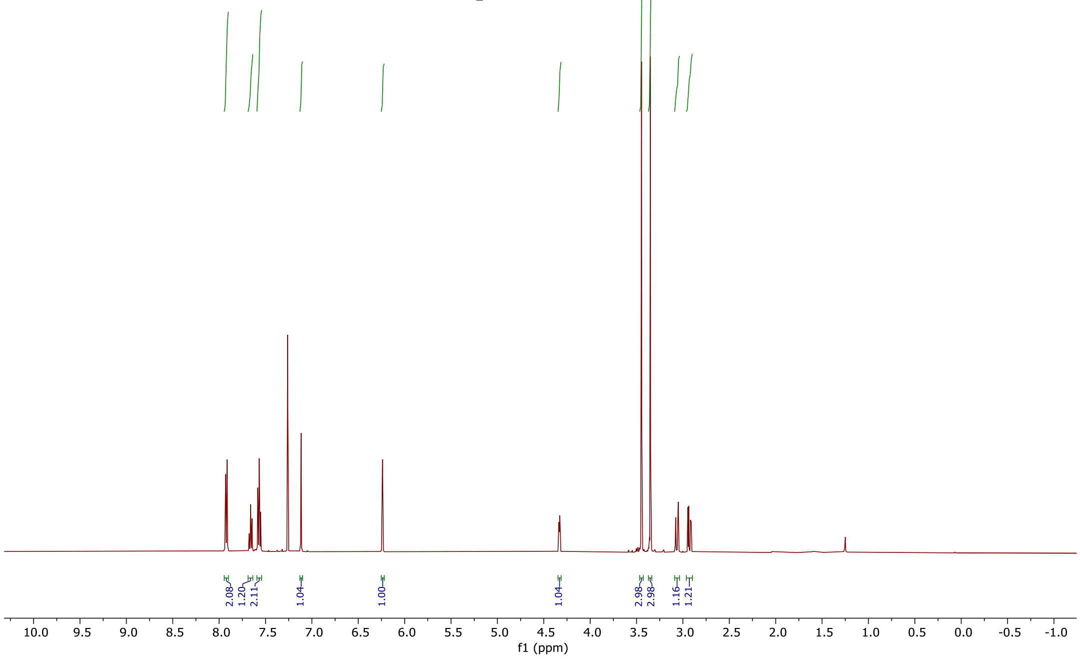

^{13}C NMR (126 MHz, Chloroform-*d*)

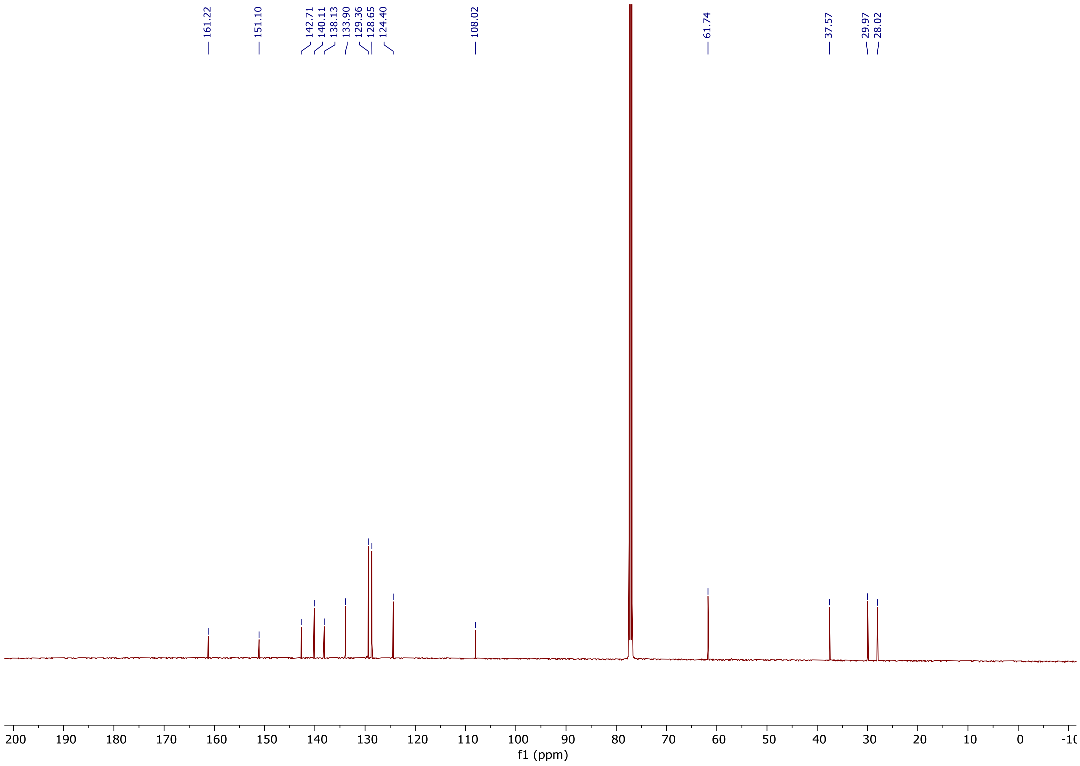

¹H COSY (500 MHz, Chloroform-*d*)

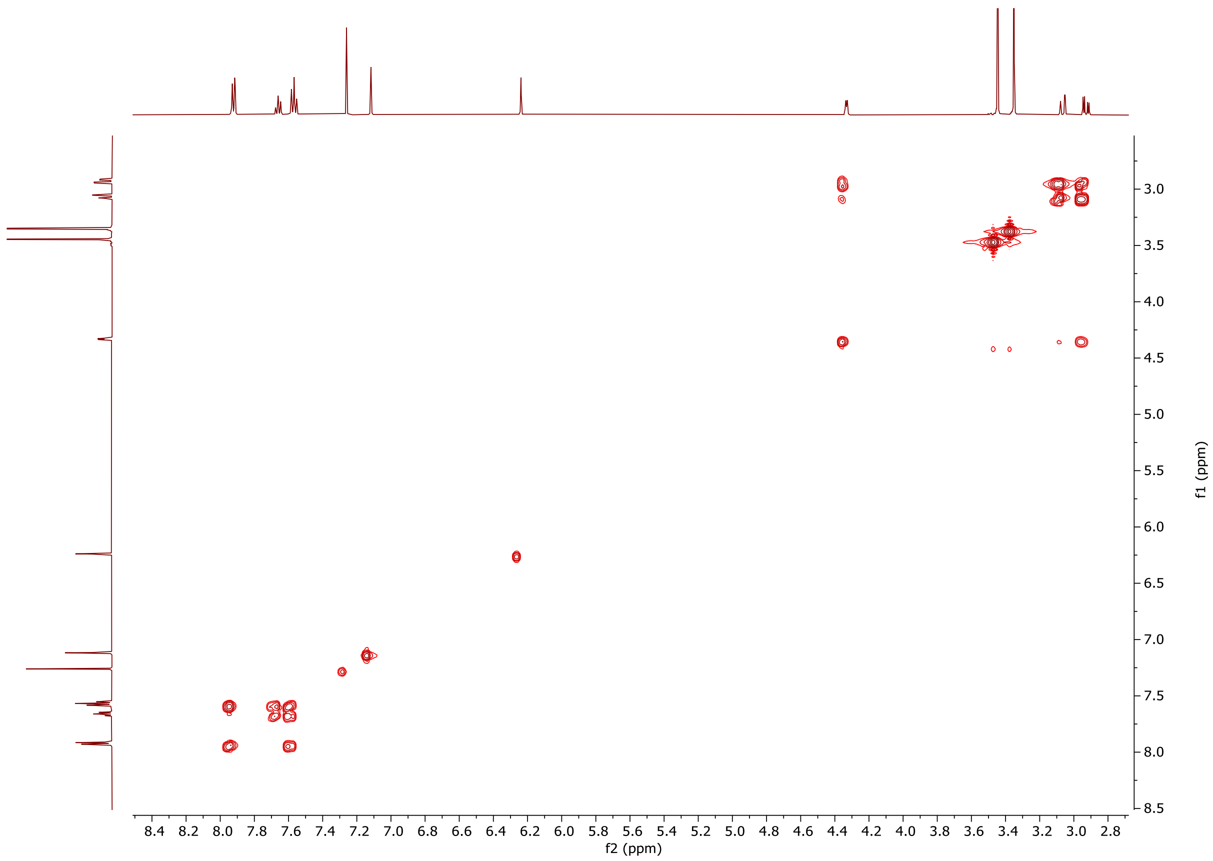

¹H/¹³C HSQC (500/126 MHz, Chloroform-*d*)

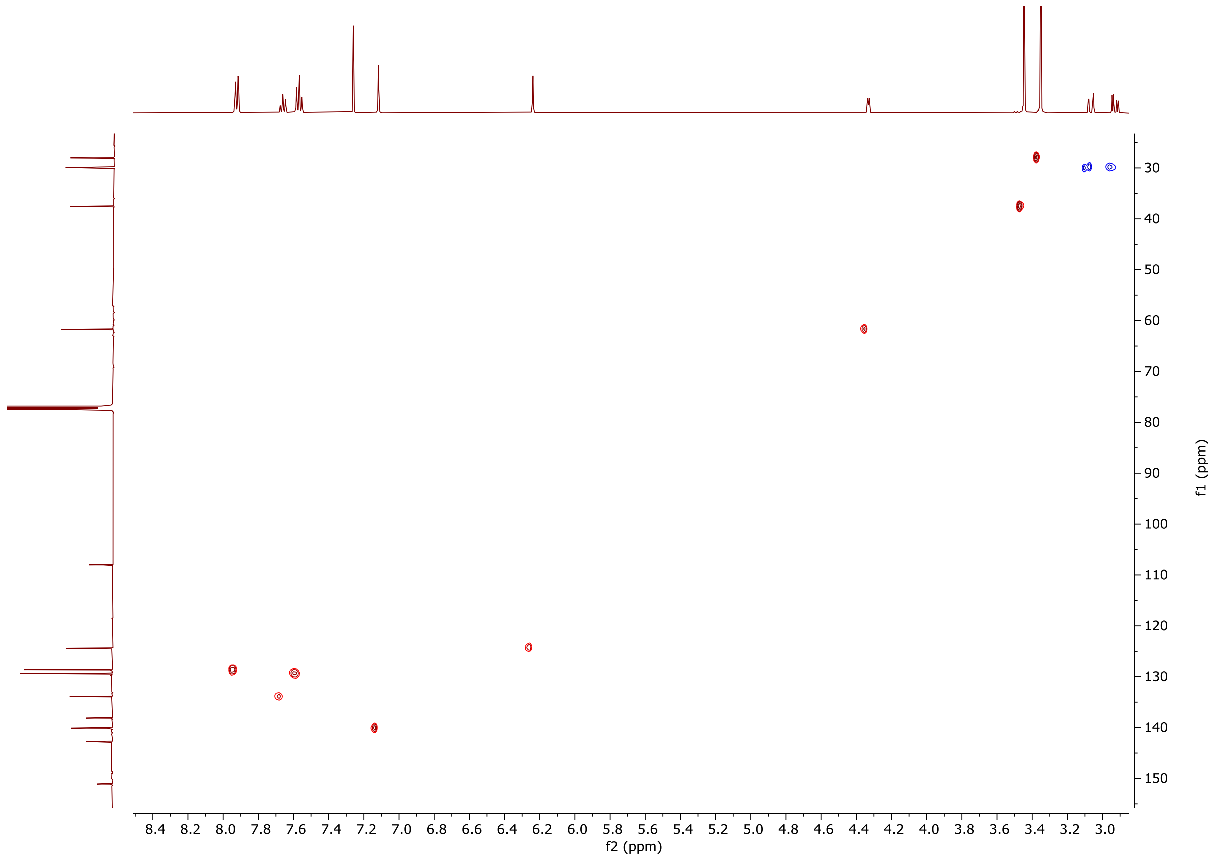

$^{1}H/^{13}C$ HMBC (500/126 MHz, Chloroform-*d*)

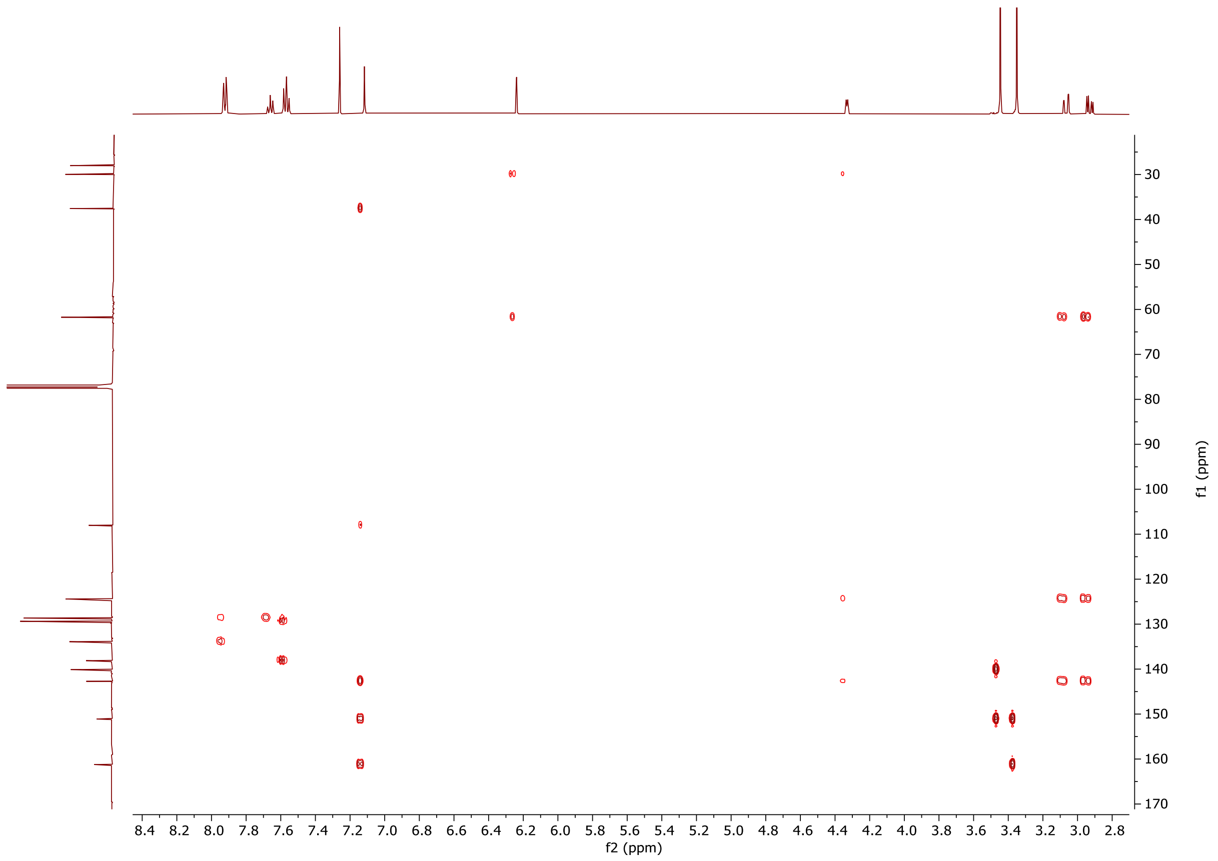

4-((-3-(Phenylsulfonyl)bicyclo[1.1.0]butan-1-yl)ethynyl)benzonitrile, 31

^{1}H NMR (500 MHz, Chloroform-*d*)

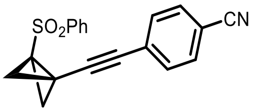

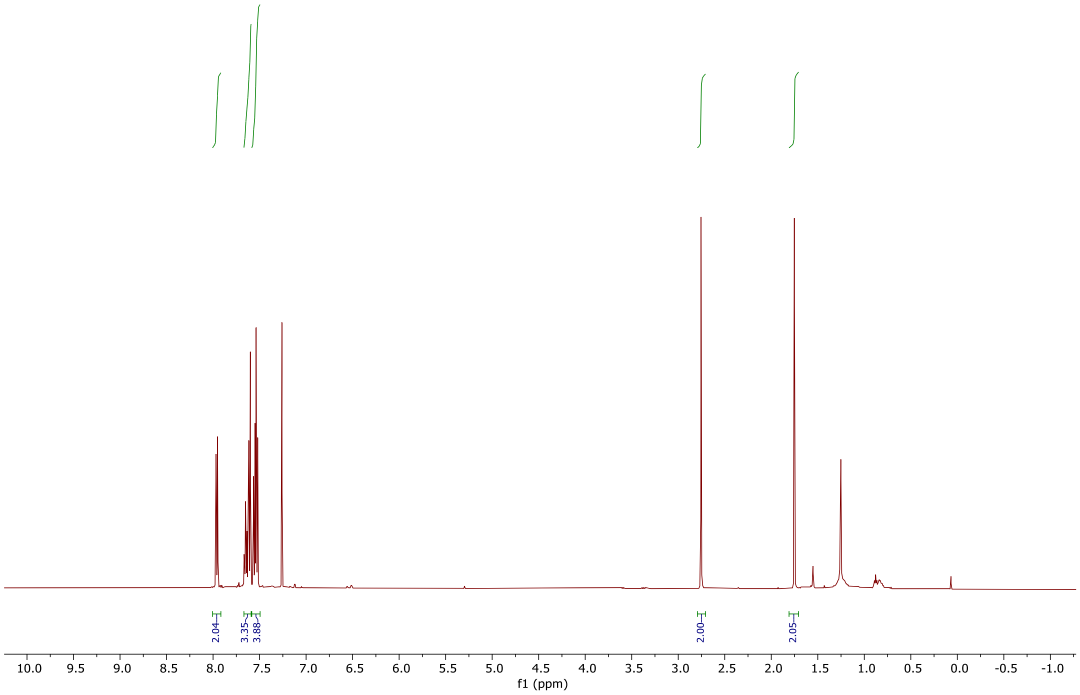

^{13}C NMR (126 MHz, Chloroform-*d*)

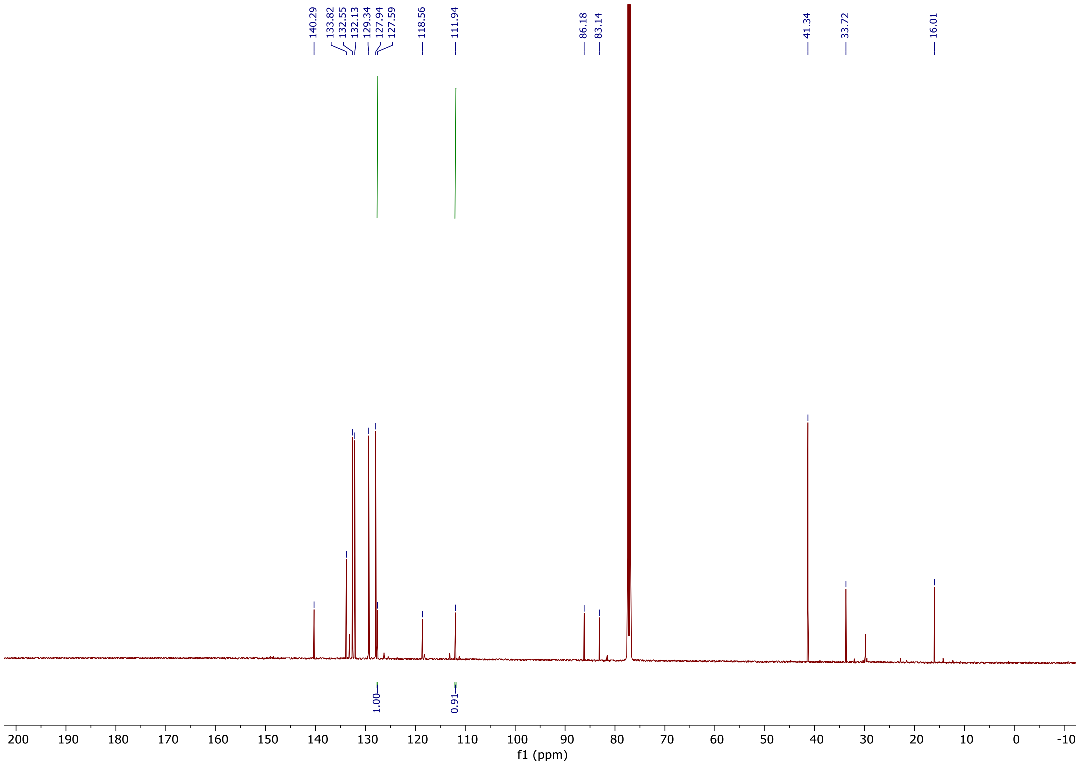

^{1}H COSY (500 MHz, Chloroform-*d*)

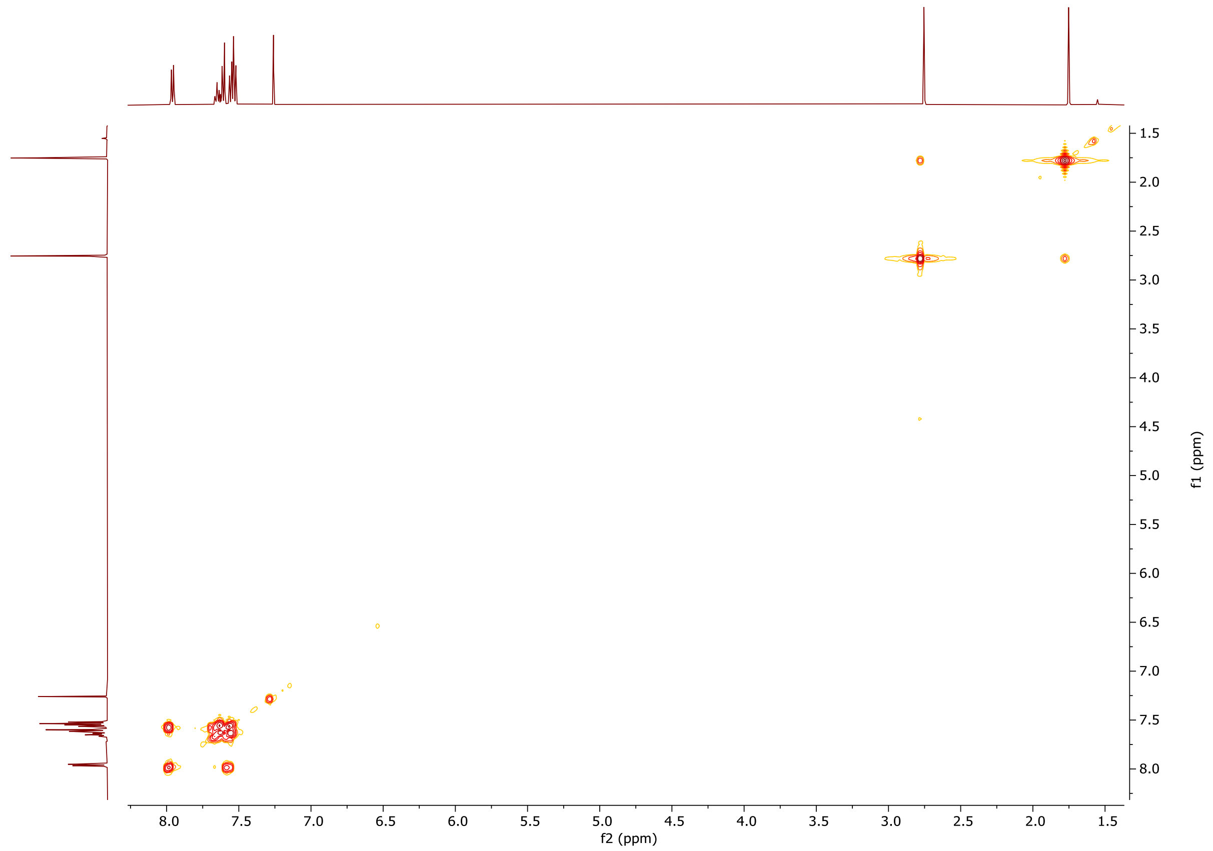

^{1}H/^{13}C HSQC (500/126 MHz, Chloroform-*d*)

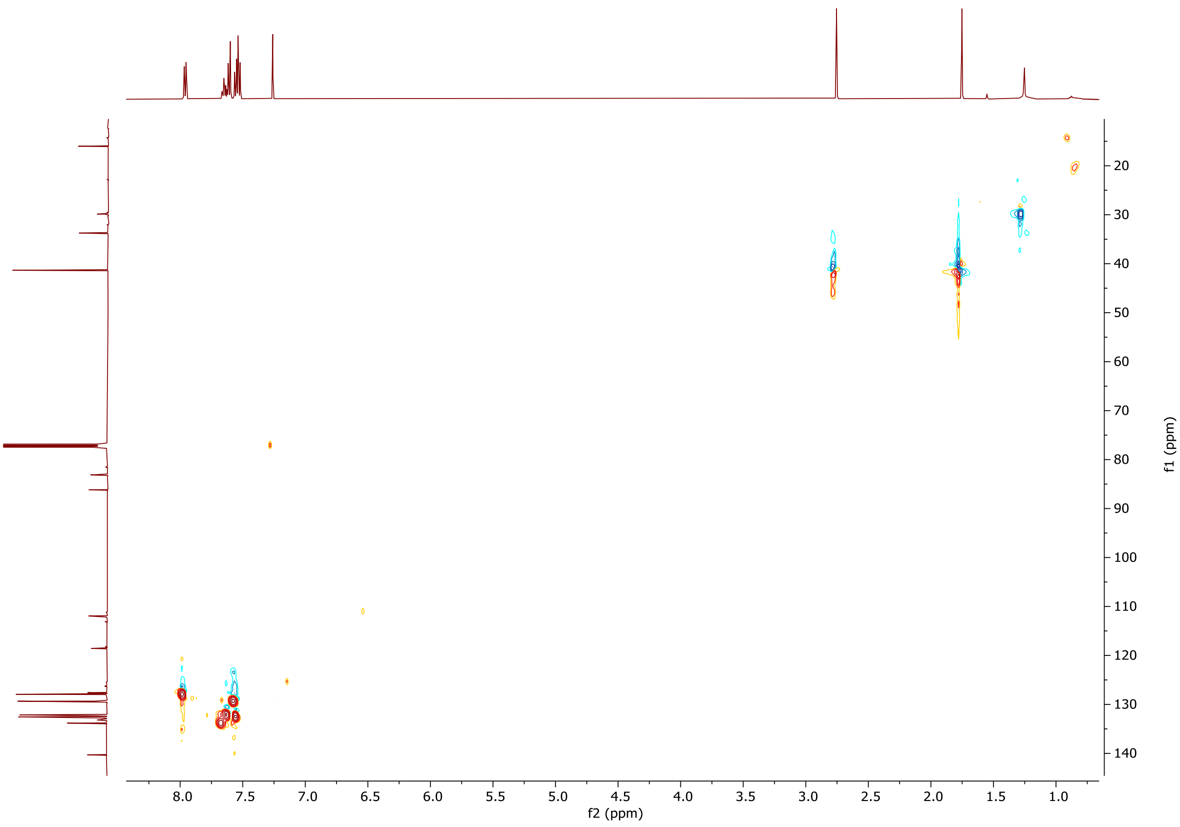

$^{1}H/^{13}C$ HMBC (500/126 MHz, Chloroform-*d*)

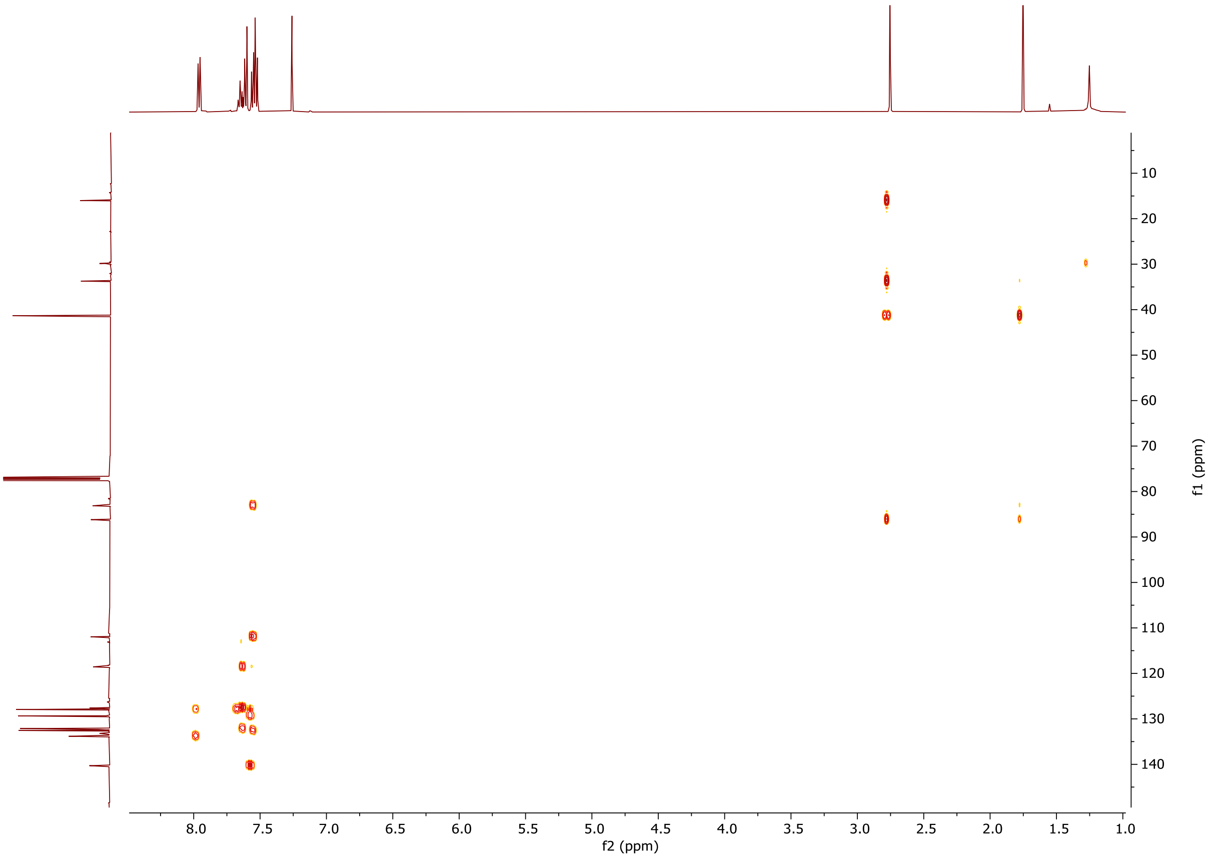

2-(3-(phenylsulfonyl)bicyclo[1.1.0]butan-1-yl)pyridine, 32

^{1}H NMR (500 MHz, Chloroform-*d*)

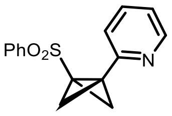

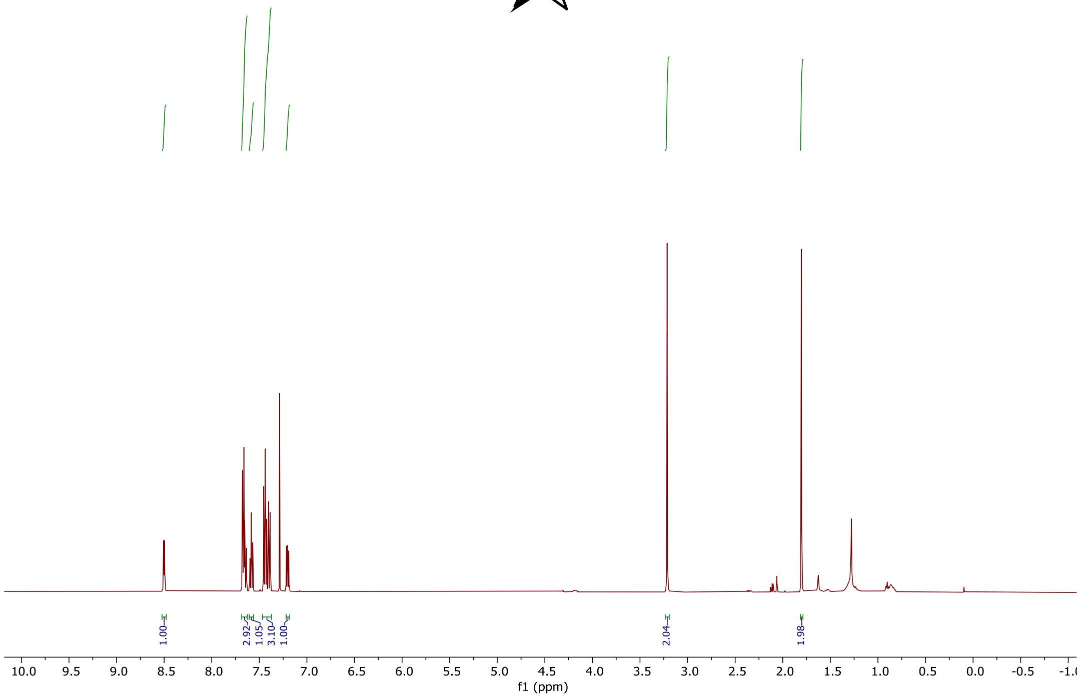

^{13}C NMR (126 MHz, Chloroform-*d*)

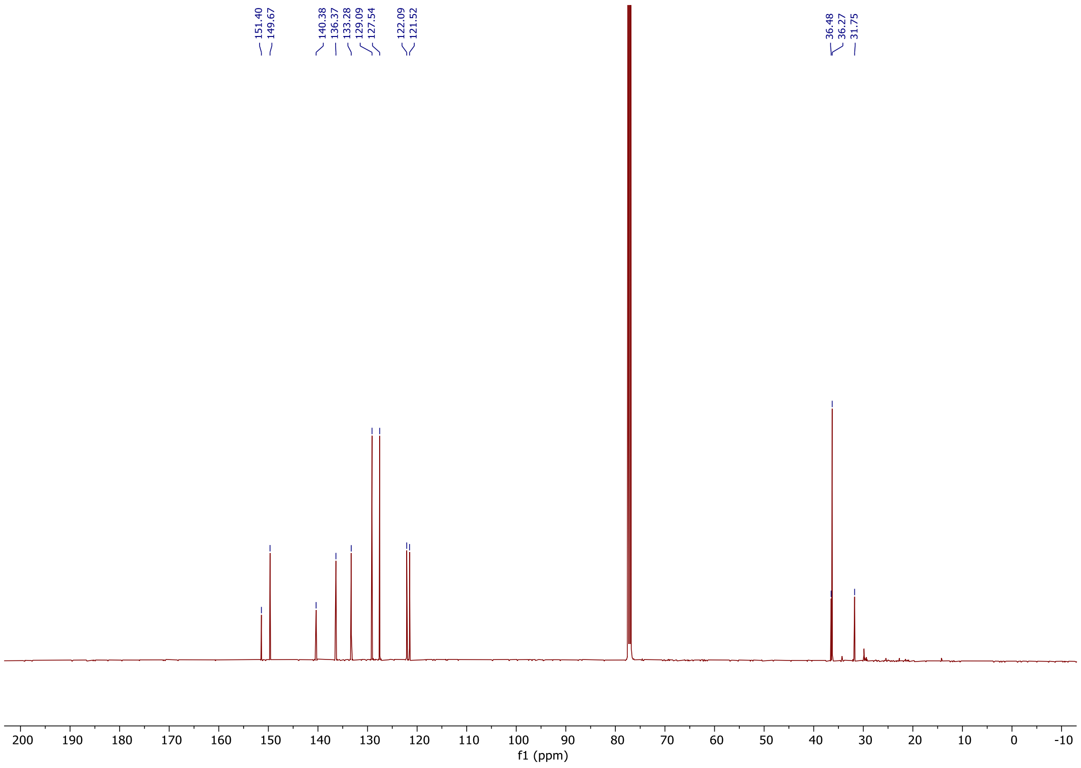

^{1}H COSY (500 MHz, Chloroform-*d*)

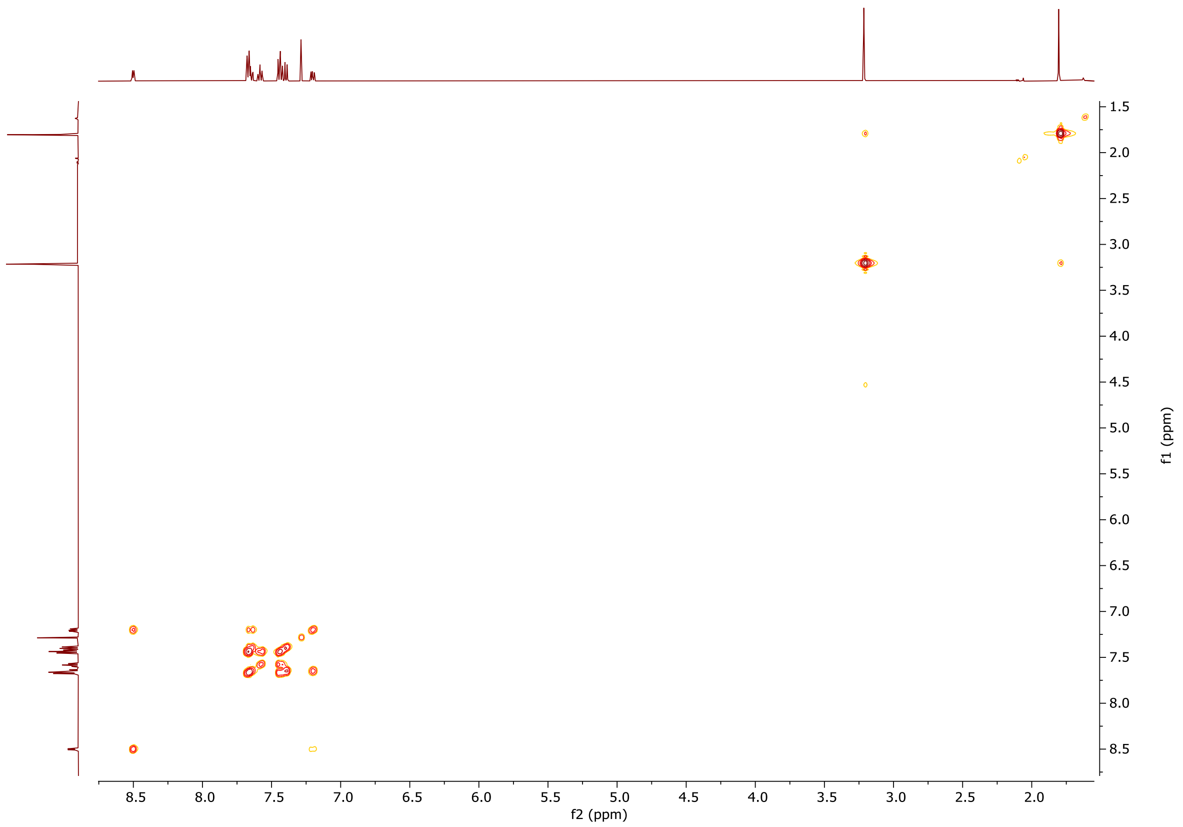

^{1}H/^{13}C HSQC (500/126 MHz, Chloroform-*d*)

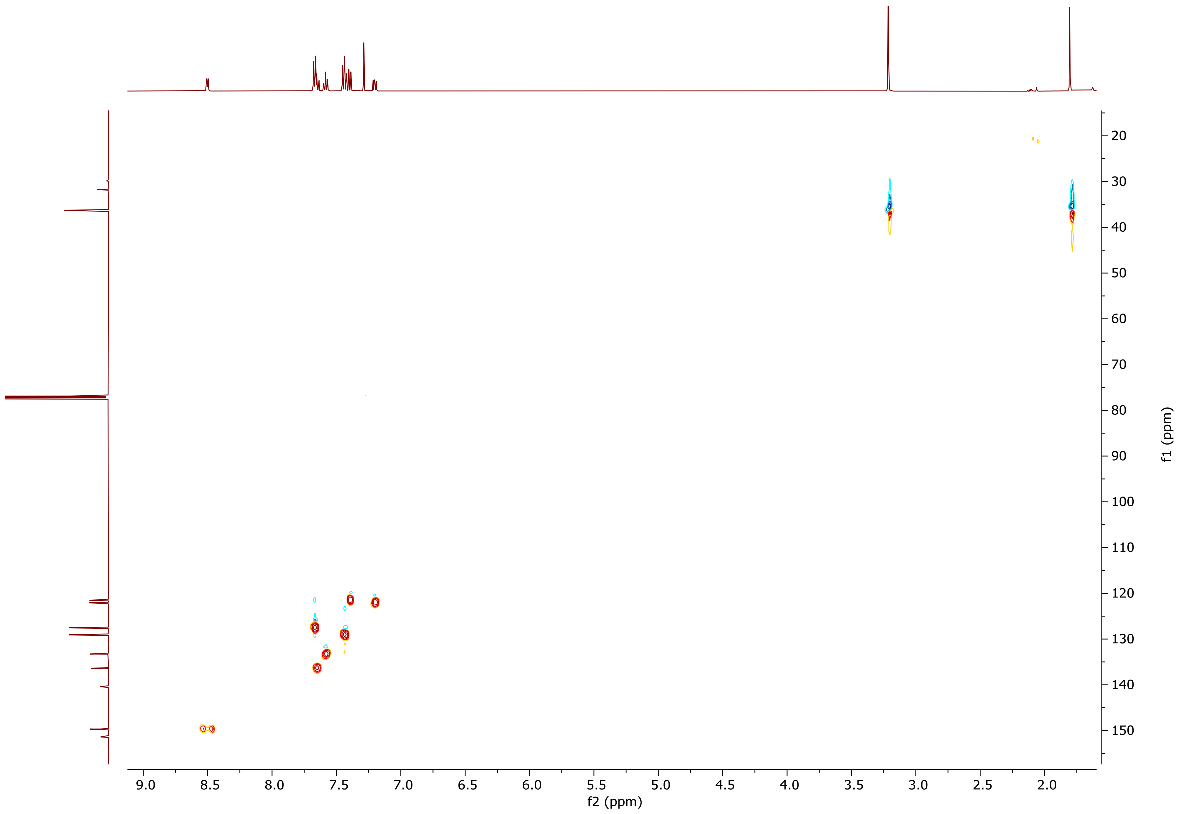

$^{1}H/^{13}C$ HMBC (500/126 MHz, Chloroform-*d*)

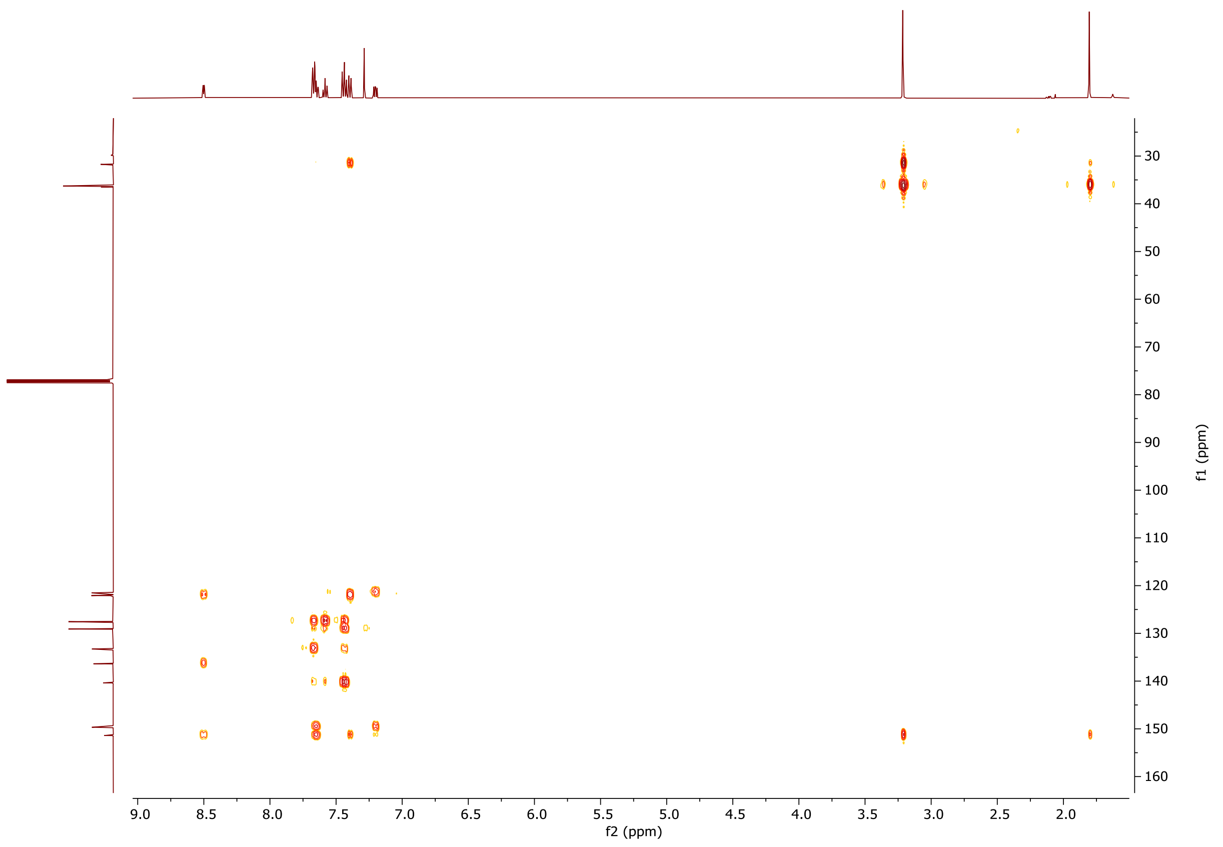

3-(3-(phenylsulfonyl)bicyclo[1.1.0]butan-1-yl)pyridine, 33

^{1}H NMR (500 MHz, Chloroform-*d*)

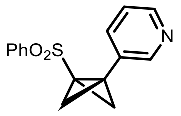

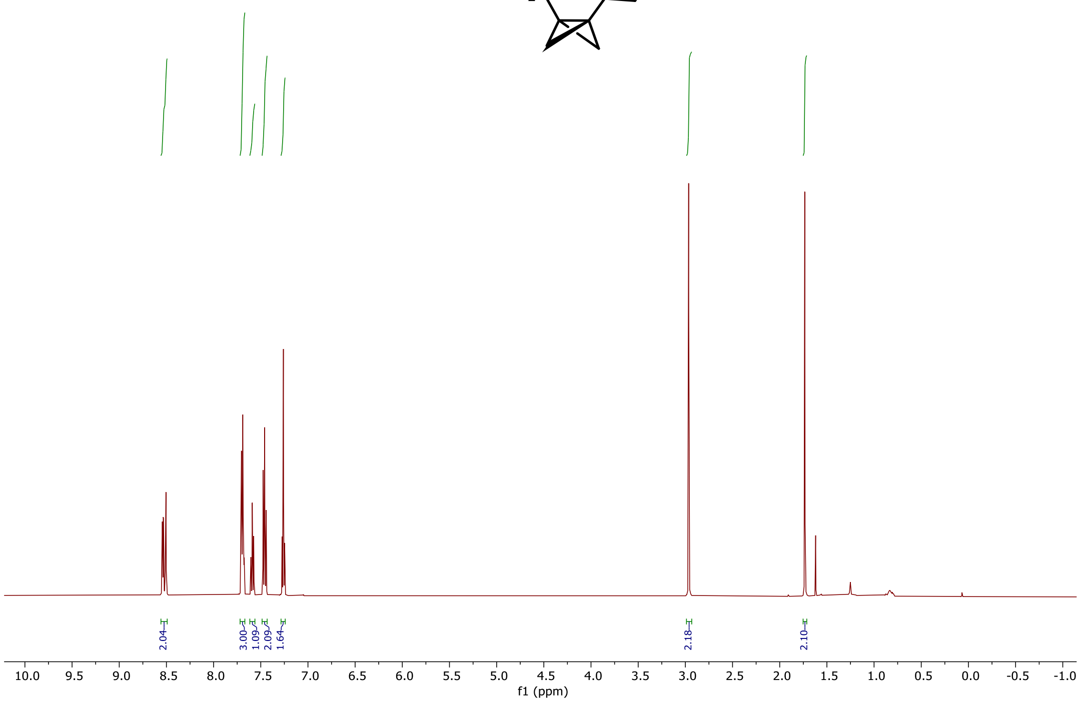

^{13}C NMR (126 MHz, Chloroform-*d*)

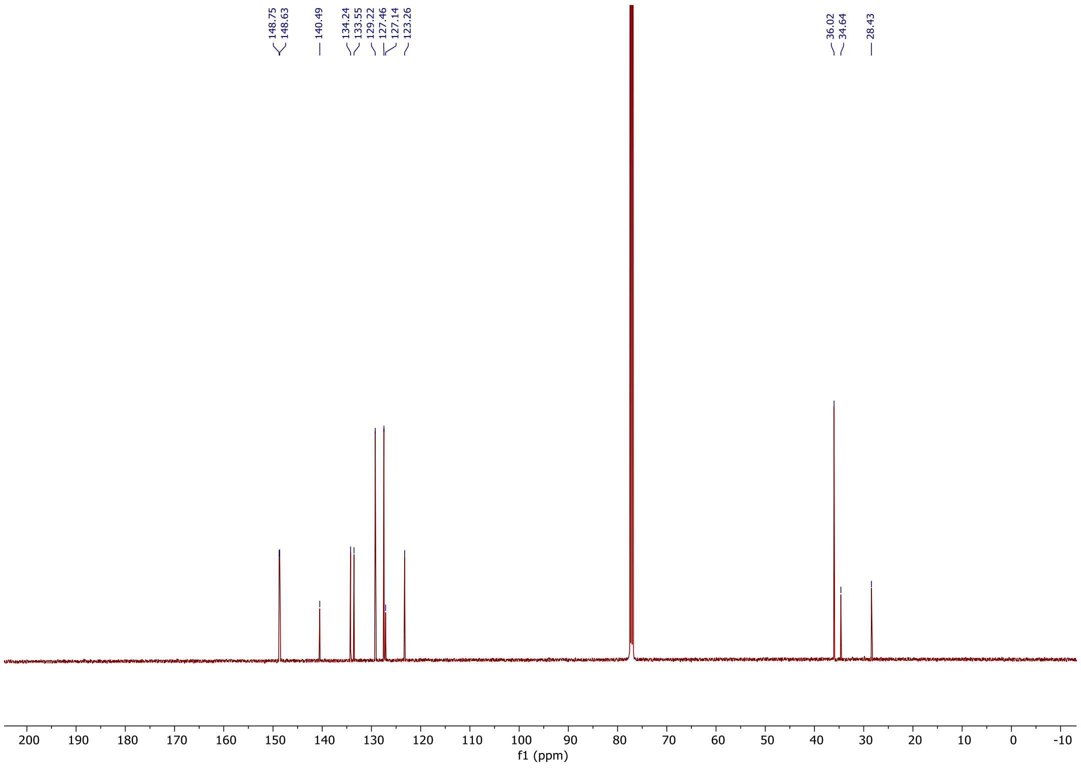

^{1}H COSY (500 MHz, Chloroform-*d*)

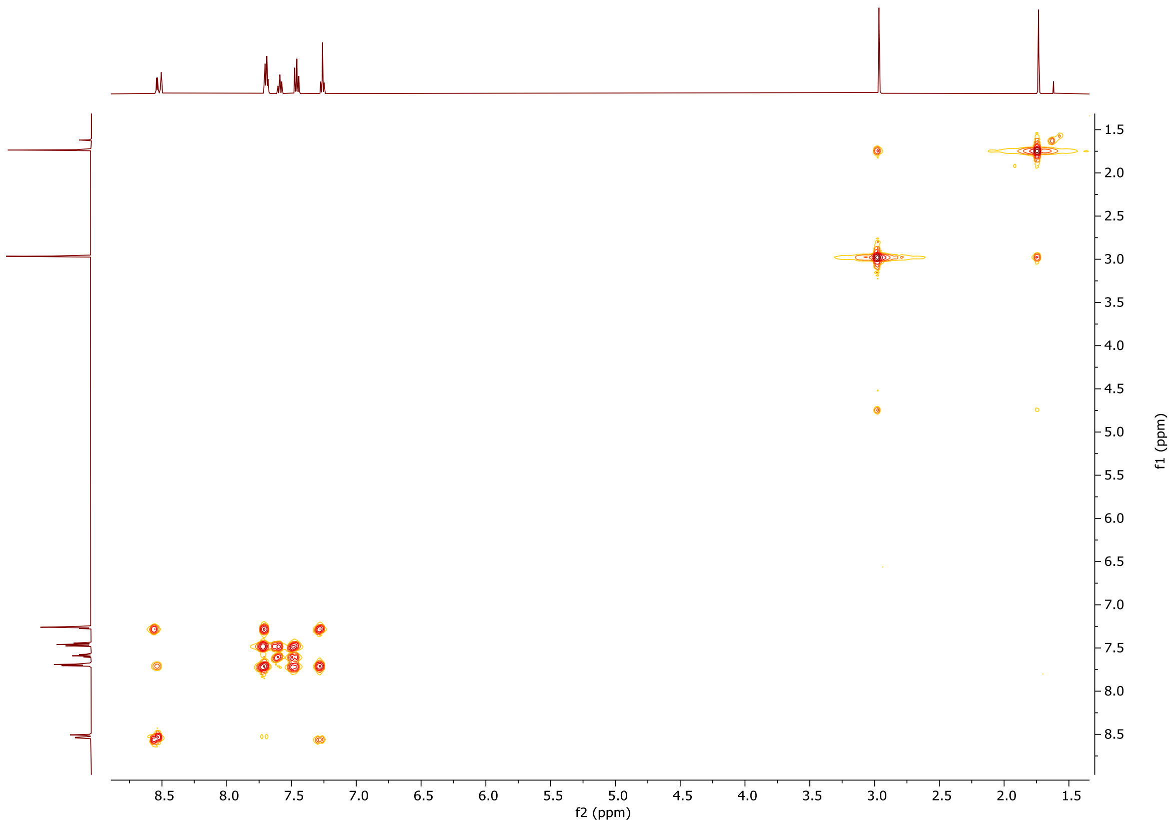

^{1}H/^{13}C HSQC (500/126 MHz, Chloroform-*d*)

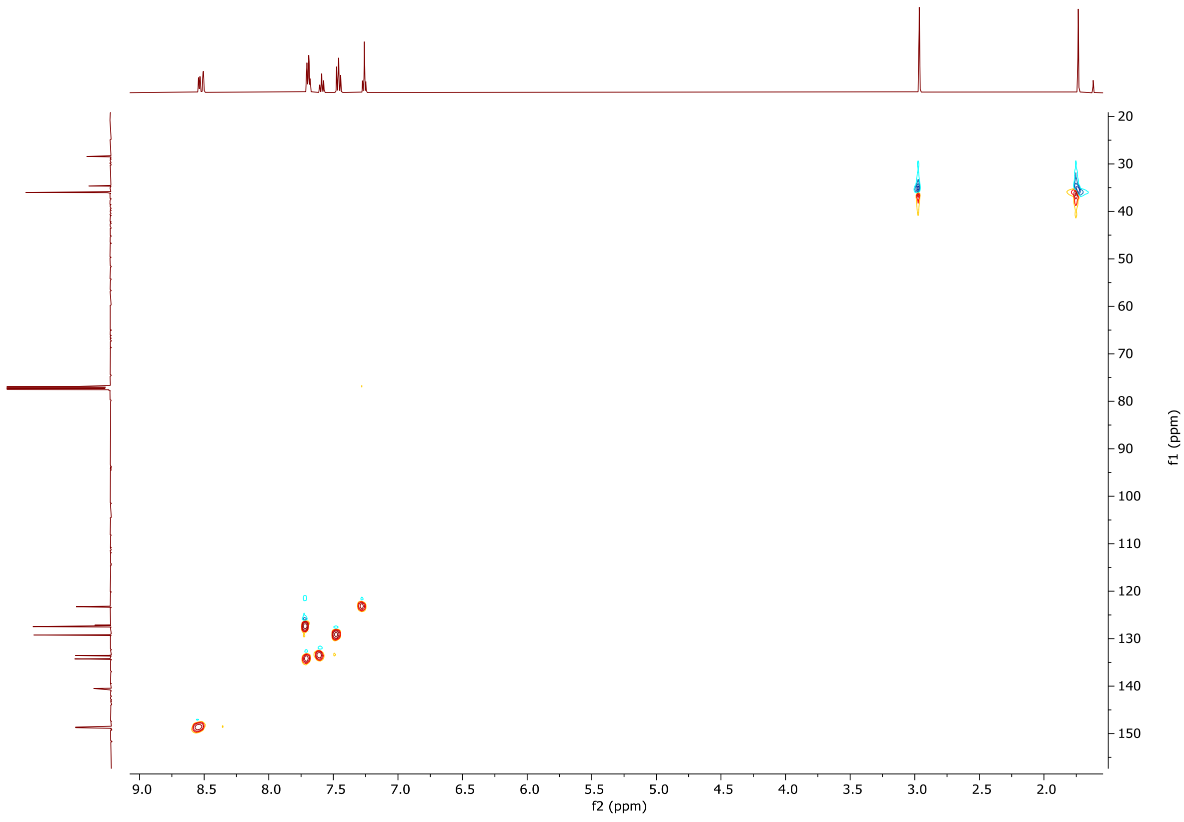

$^{1}H/^{13}C$ HMBC (500/126 MHz, Chloroform-*d*)

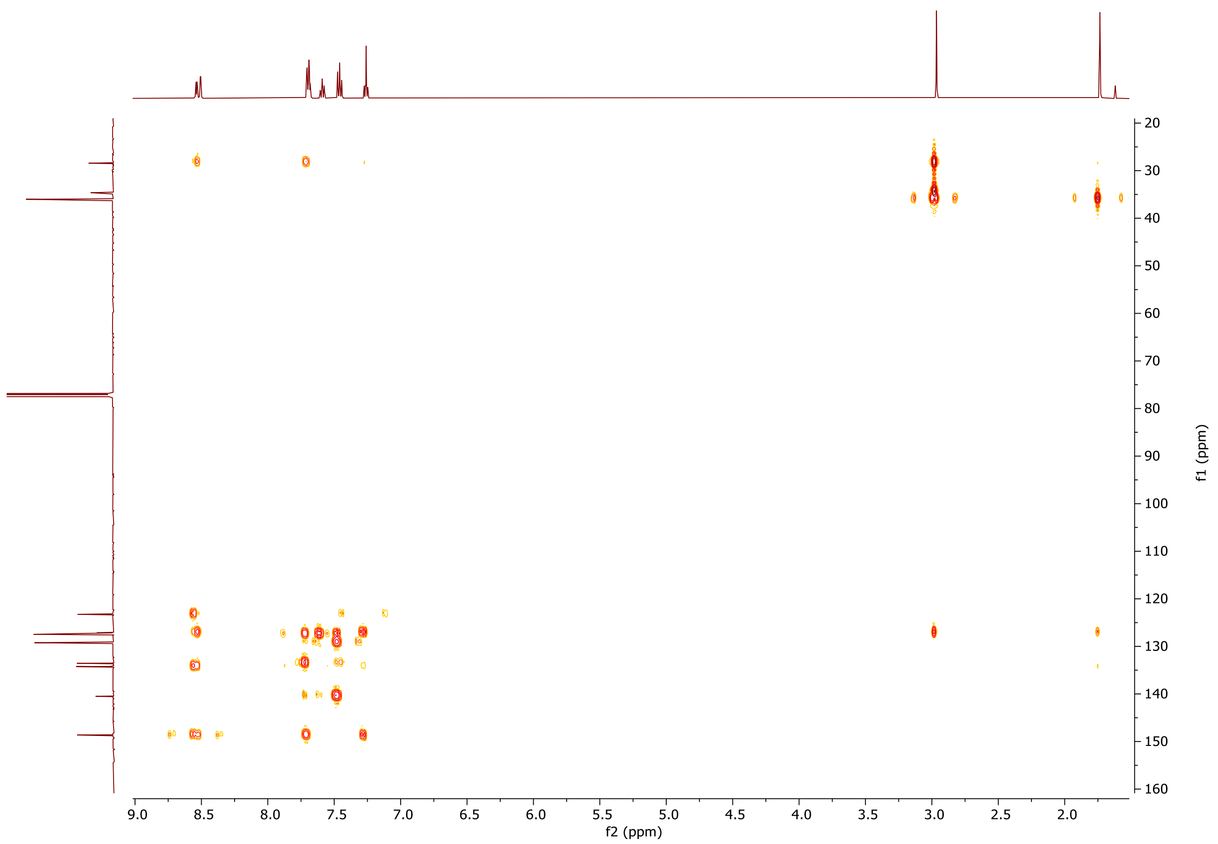

4-(3-(phenylsulfonyl)bicyclo[1.1.0]butan-1-yl)pyridine, 34

^{1}H NMR (500 MHz, Chloroform-*d*)

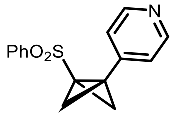

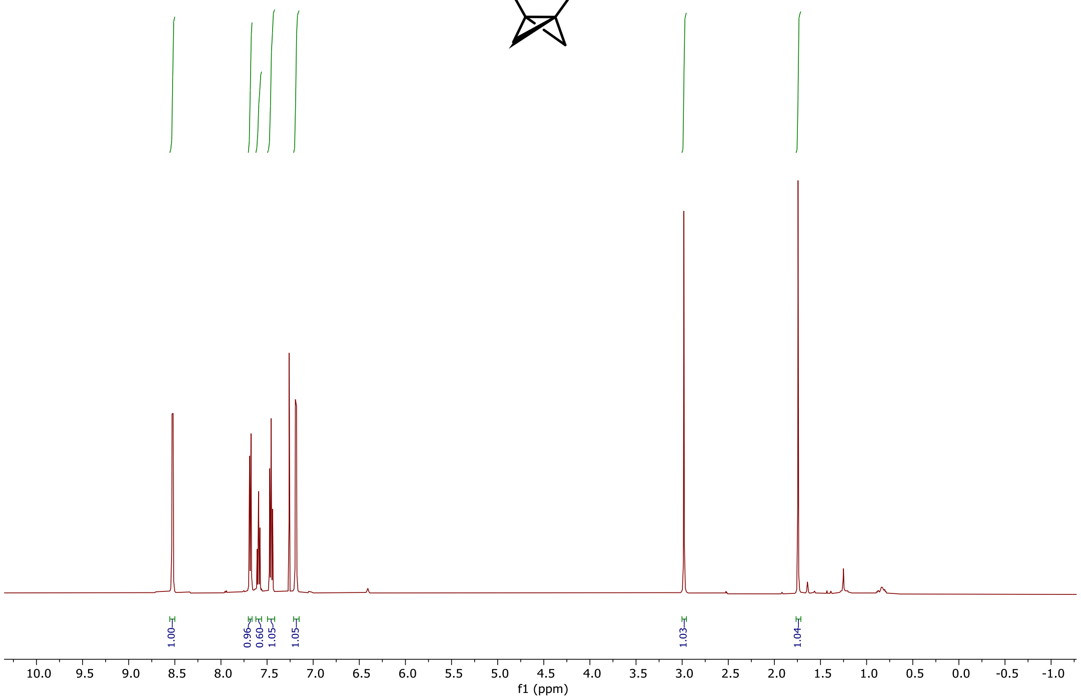

^{13}C NMR (126 MHz, Chloroform-*d*)

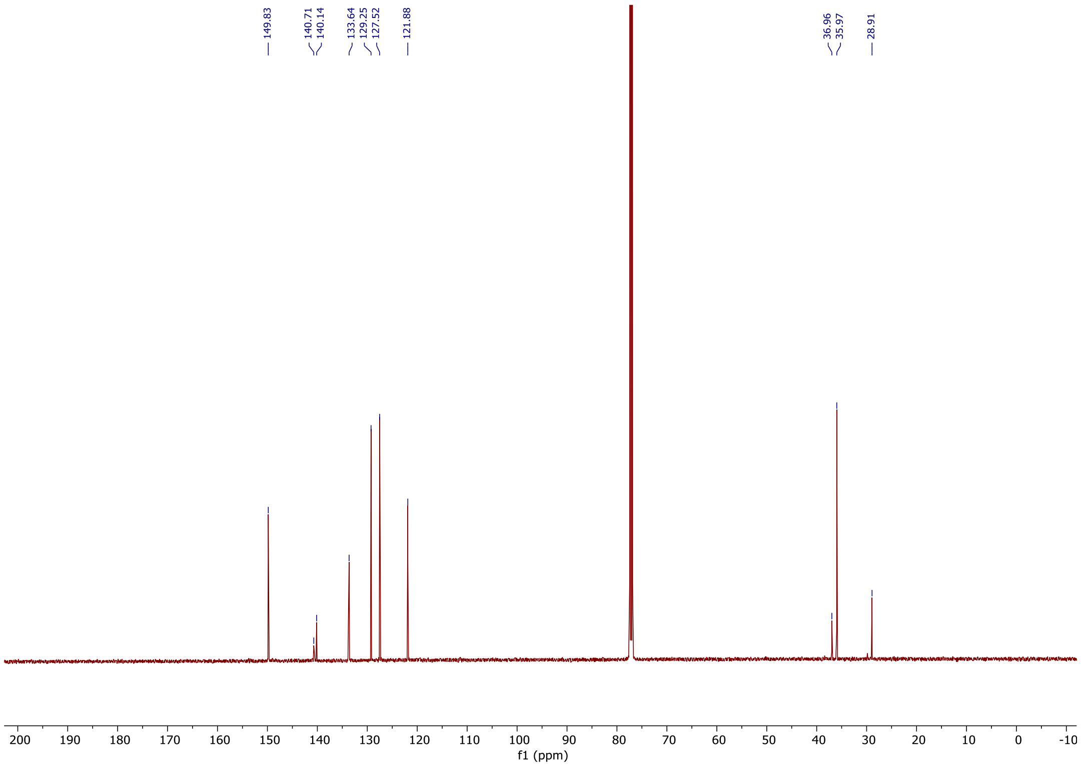

^{1}H COSY (500 MHz, Chloroform-*d*)

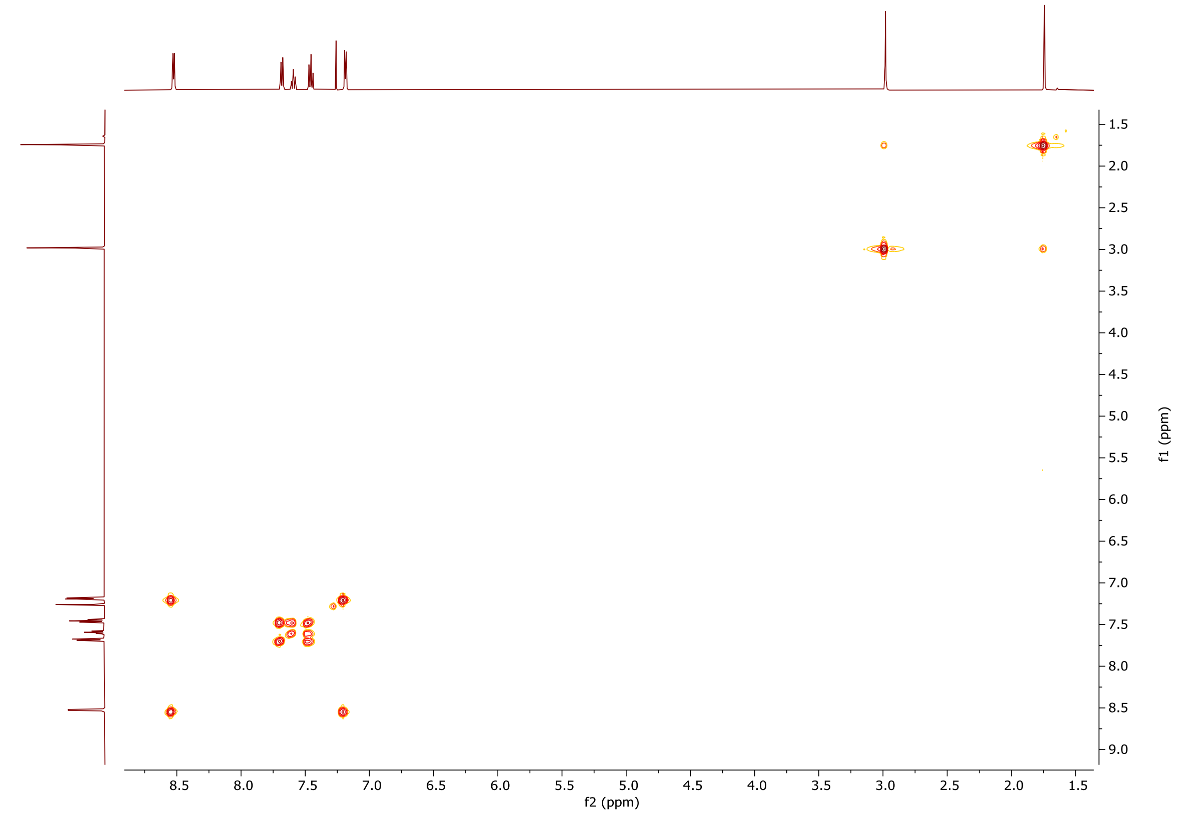

^{1}H/^{13}C HSQC (500/126 MHz, Chloroform-*d*)

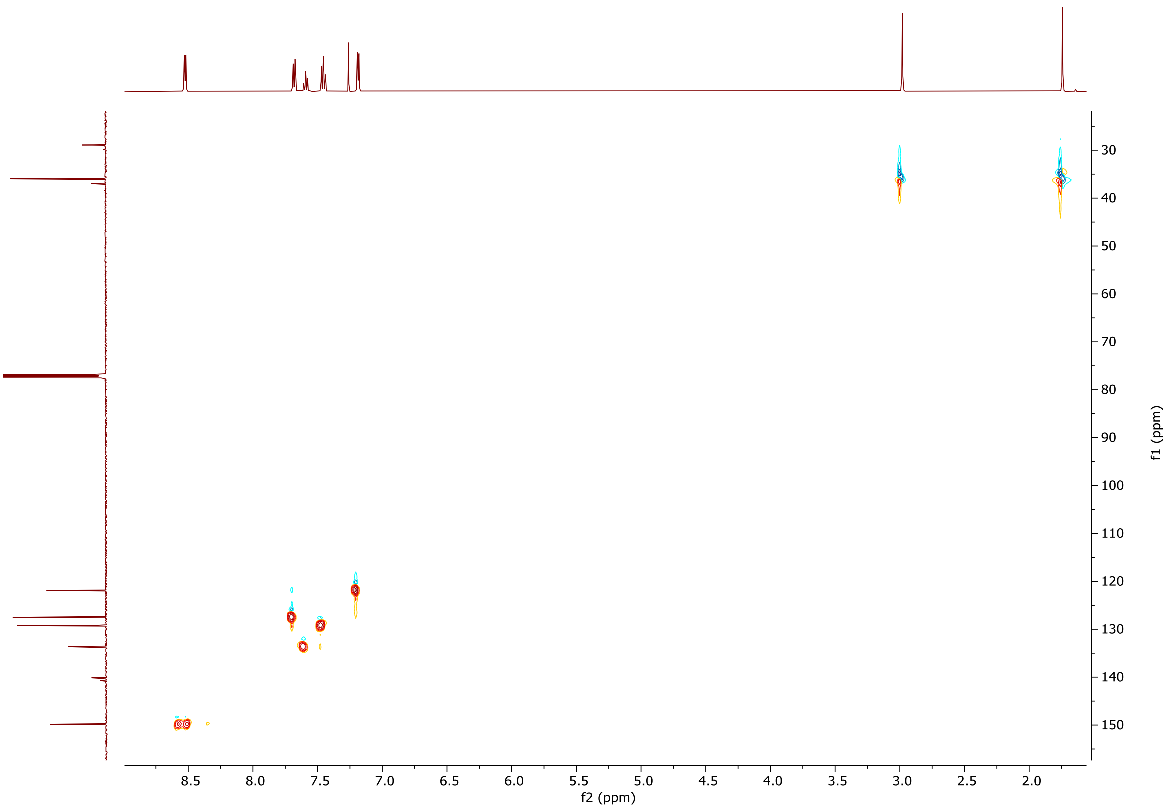

^{1}H/^{13}C HMBC (500/126 MHz, Chloroform-*d*)

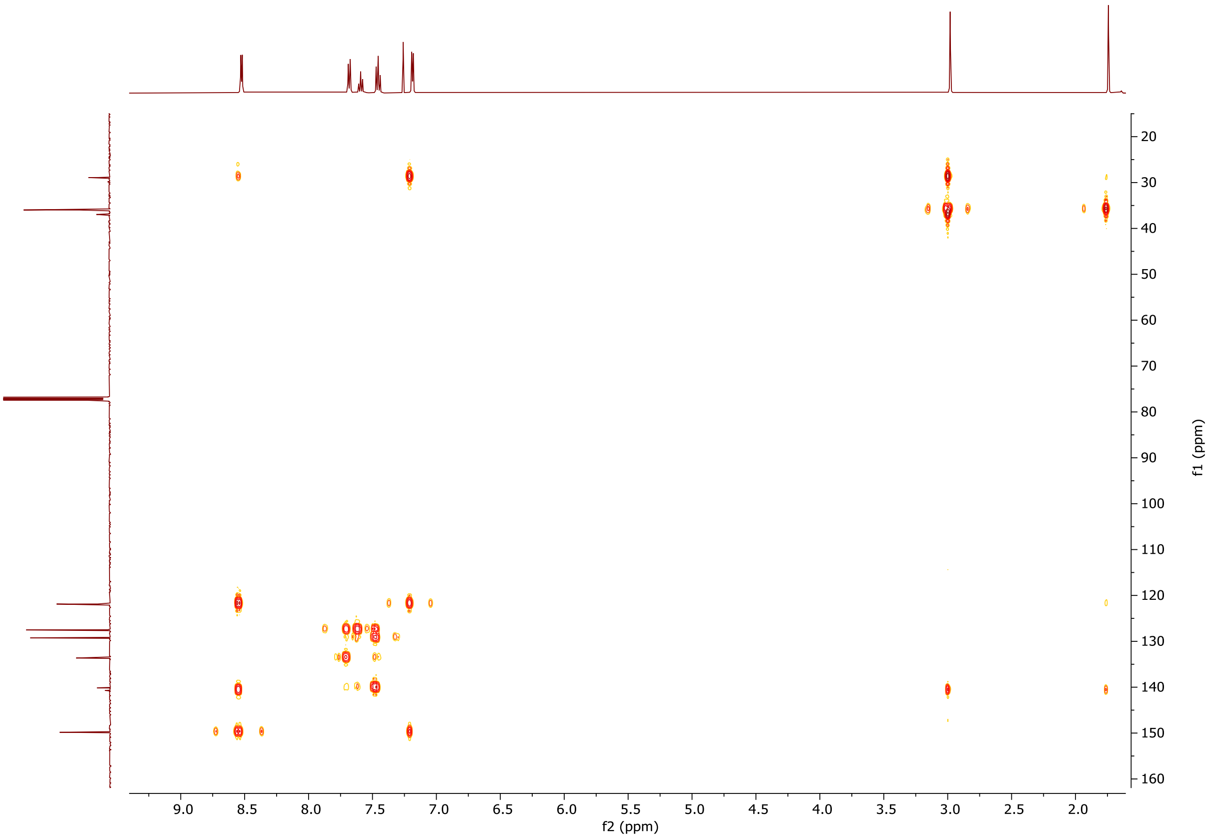

7-chloro-4-(3-(phenylsulfonyl)bicyclo[1.1.0]butan-1-yl)quinolone, 35

^{1}H NMR (500 MHz, Chloroform-*d*)

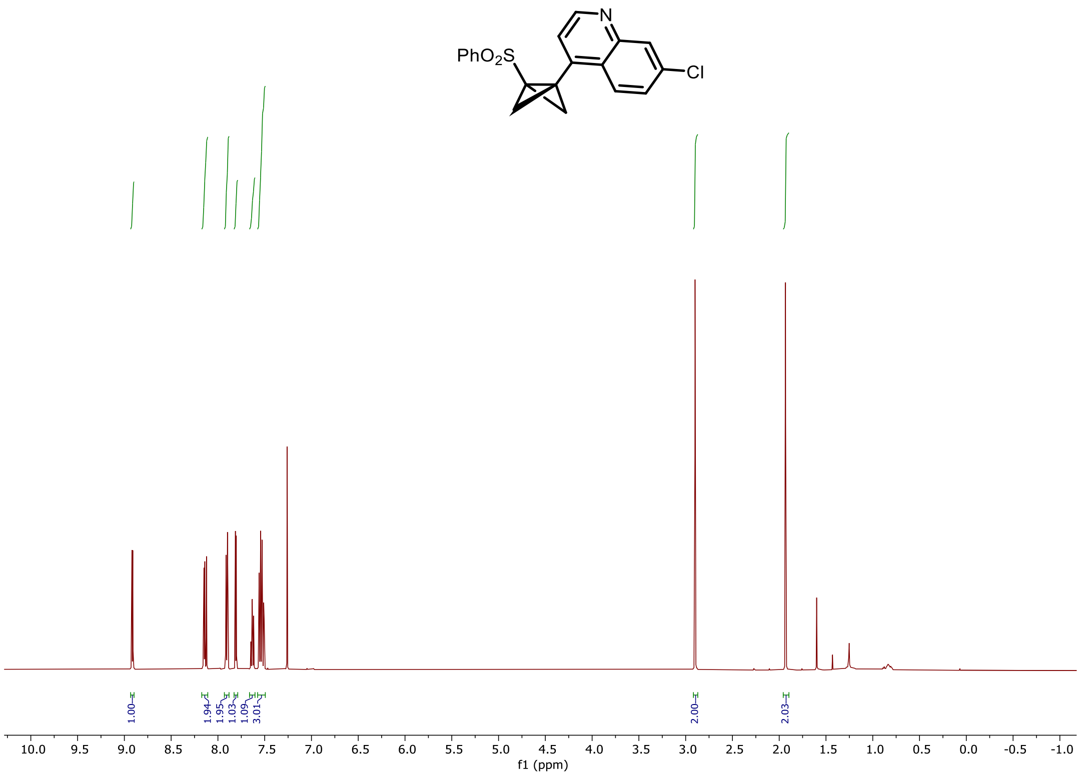

^{13}C NMR (126 MHz, Chloroform-*d*)

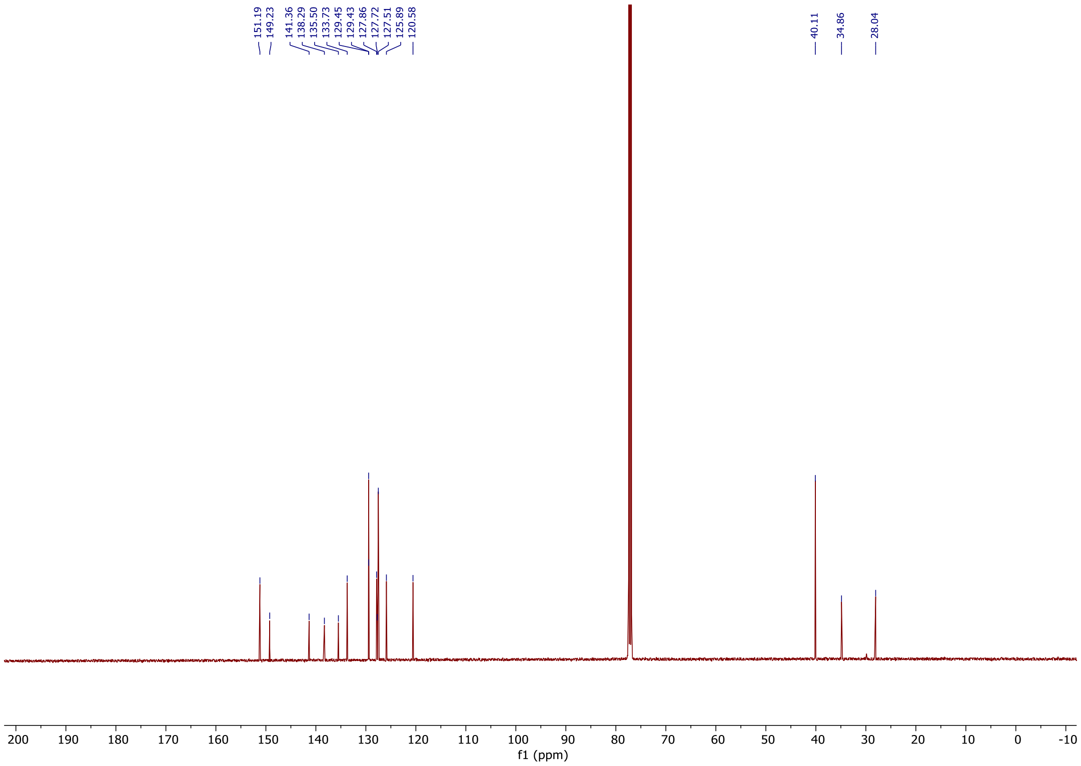

^{1}H COSY (500 MHz, Chloroform-*d*)

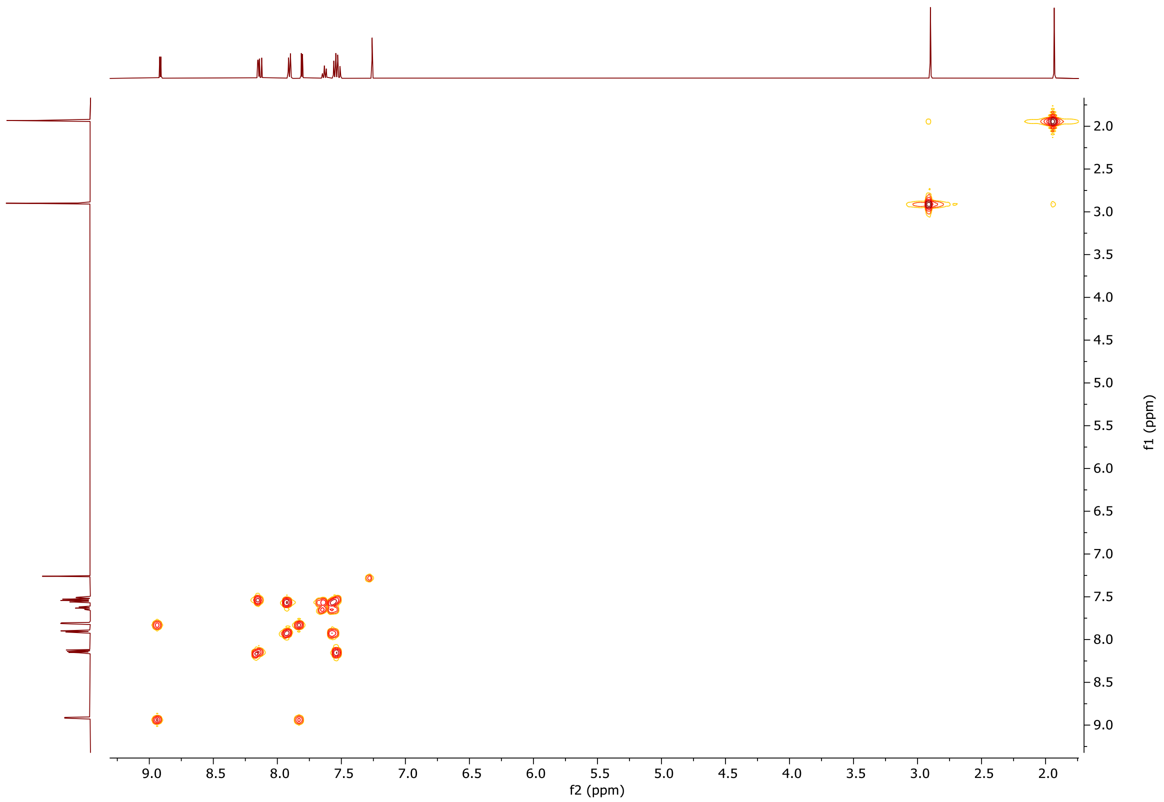

^{1}H/^{13}C HSQC (500/126 MHz, Chloroform-*d*)

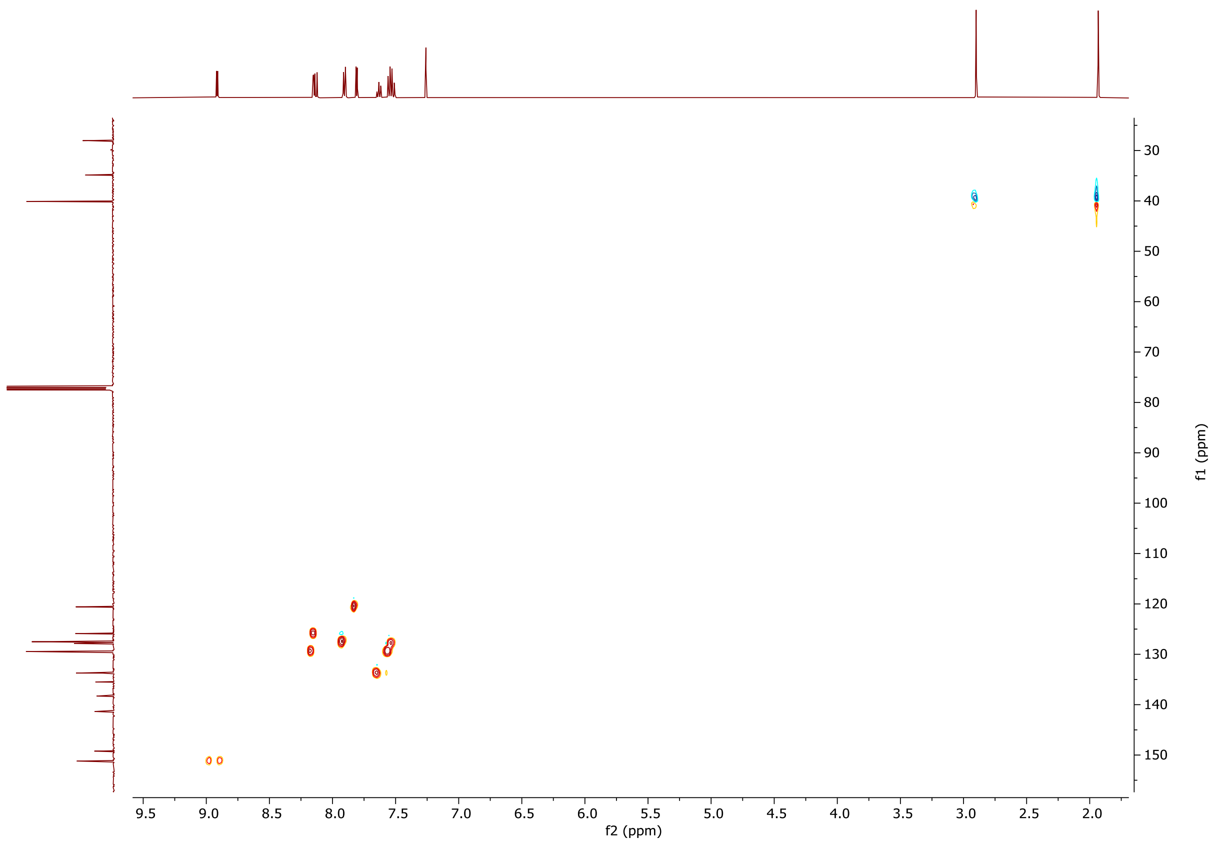

^{1}H/^{13}C HMBC (500/126 MHz, Chloroform-*d*)

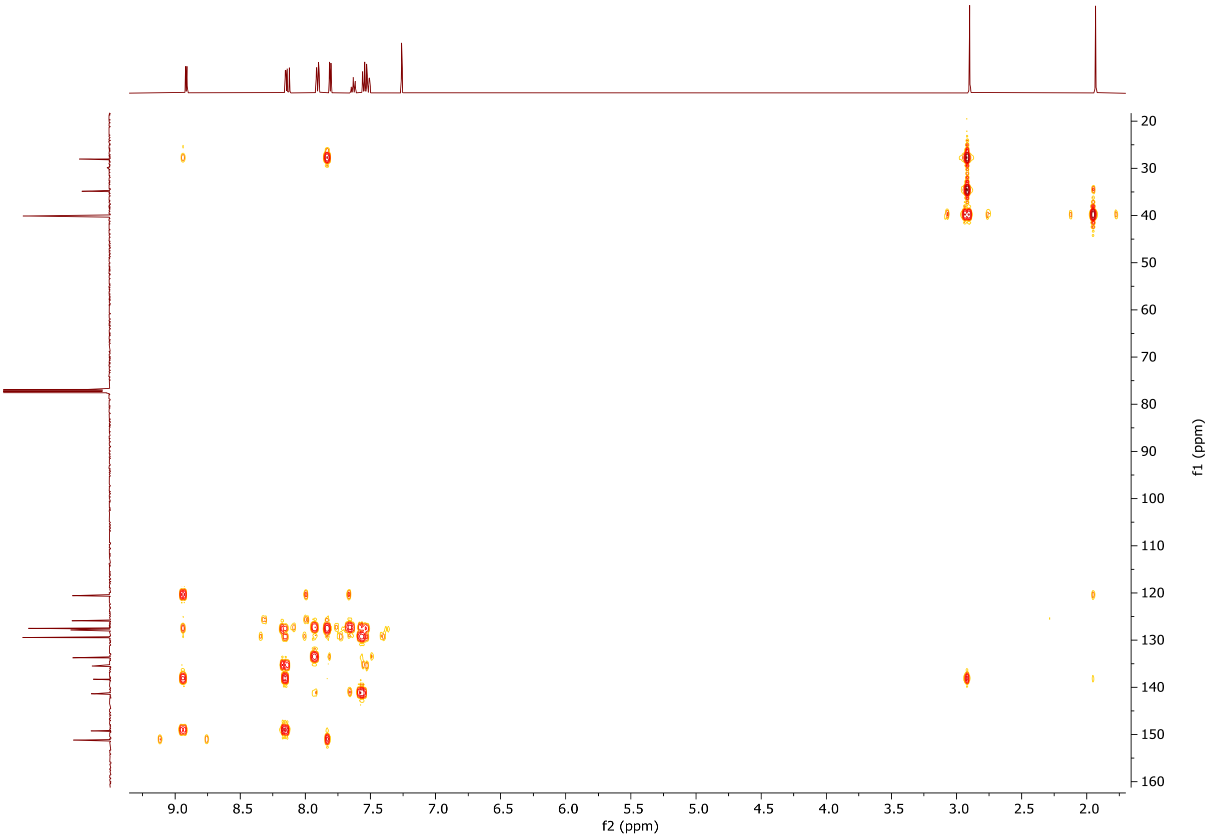

1-(3-(phenylsulfonyl)bicyclo[1.1.0]butan-1-yl)isoquinoline, 36

^{1}H NMR (500 MHz, Chloroform-*d*)

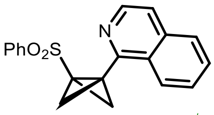

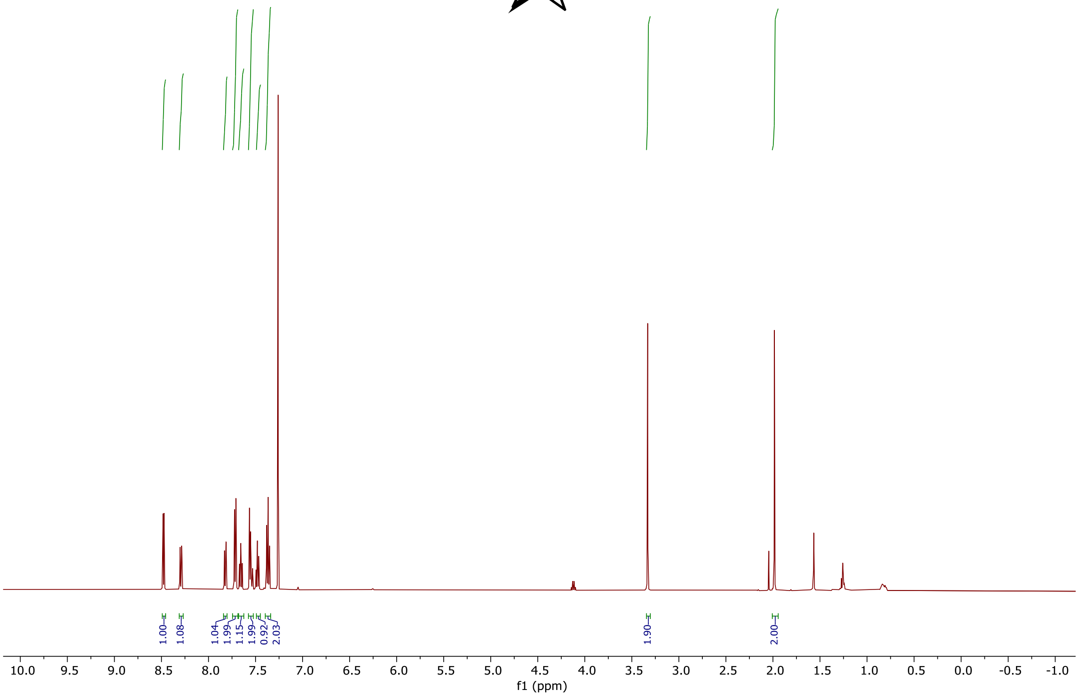

^{13}C NMR (126 MHz, Chloroform-*d*)

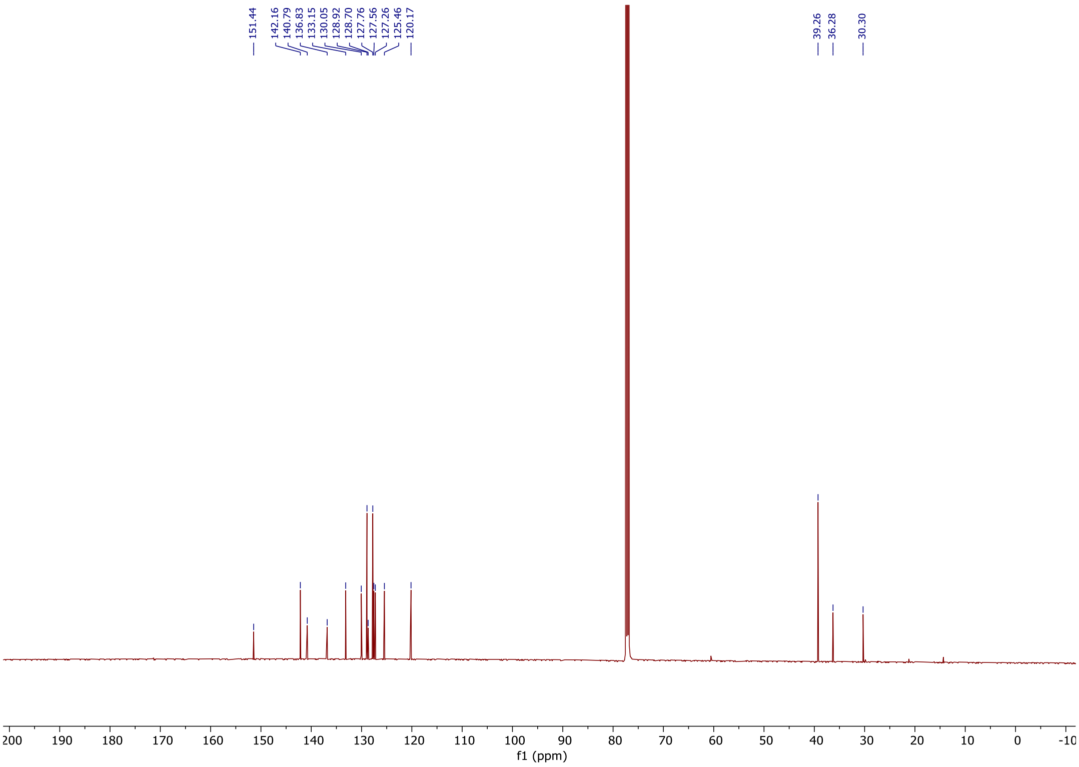

^{1}H COSY (500 MHz, Chloroform-*d*)

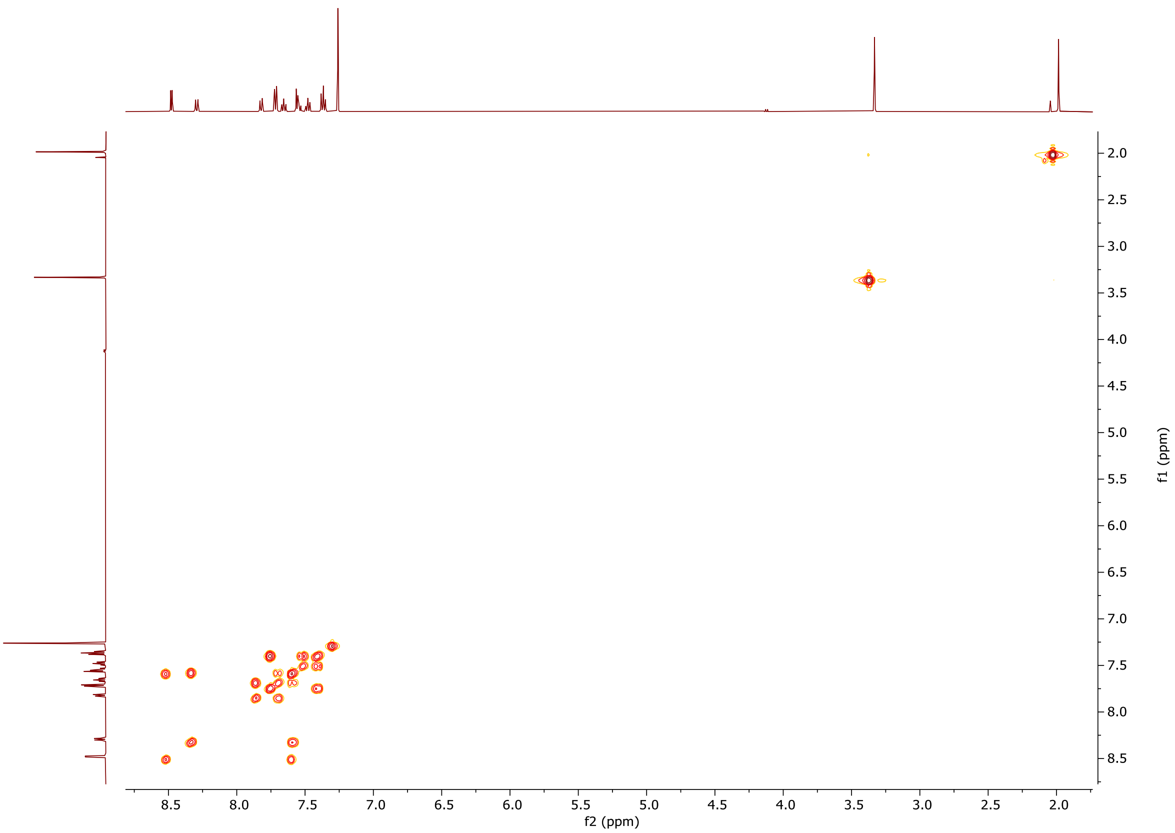

^{1}H/^{13}C HSQC (500/126 MHz, Chloroform-*d*)

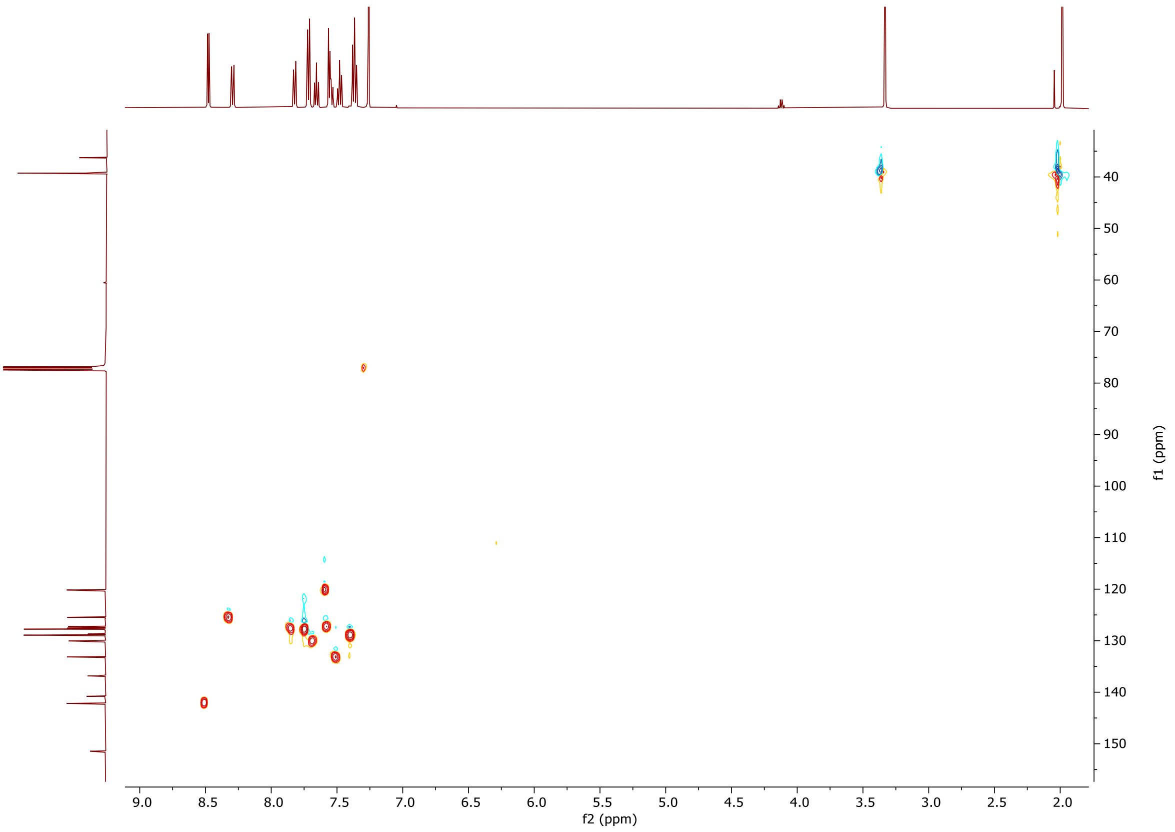

$^{1}H/^{13}C$ HMBC (500/126 MHz, Chloroform-*d*)

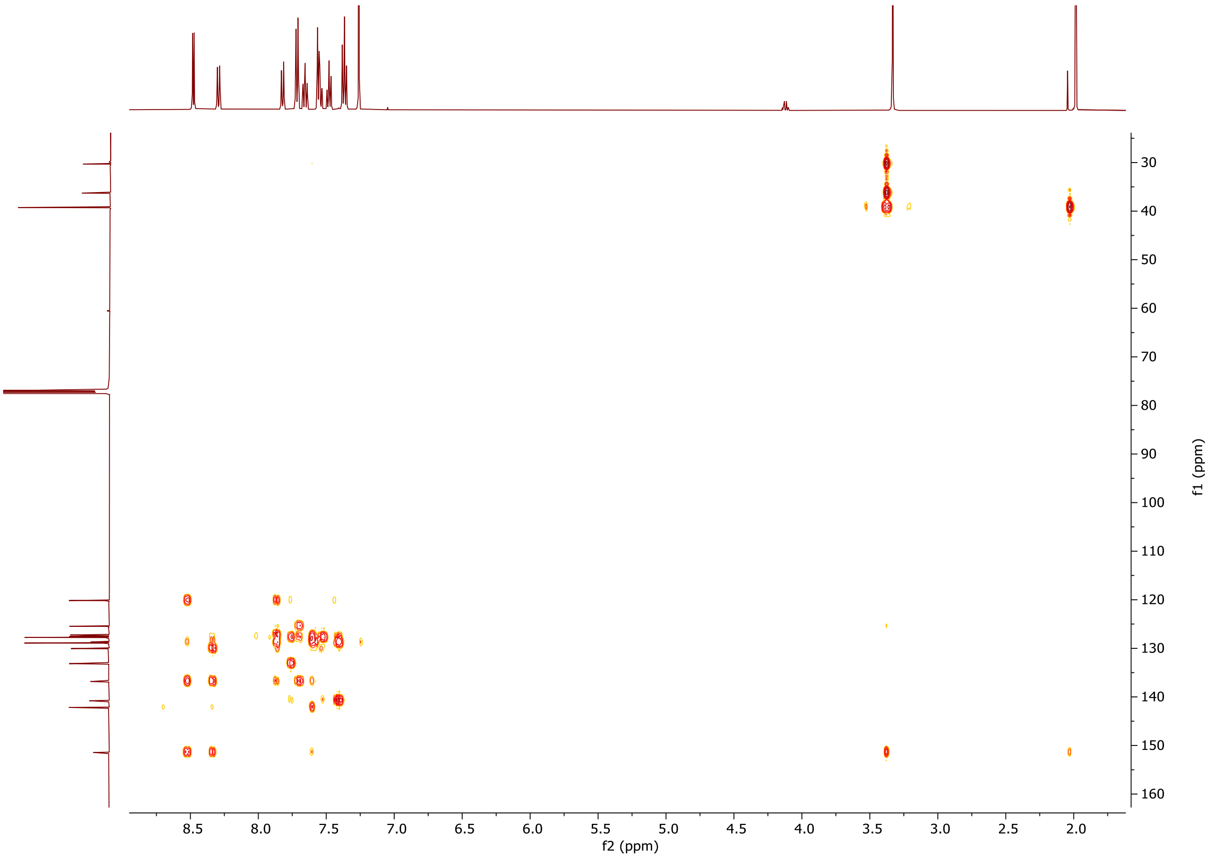

***Tert*-butyl 5-(3-(phenylsulfonyl)bicyclo[1.1.0]butan-1-yl)-1*H*-indole-1-carboxylate, 37**

^{1}H NMR (500 MHz, Benzene-*d*)

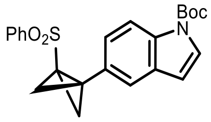

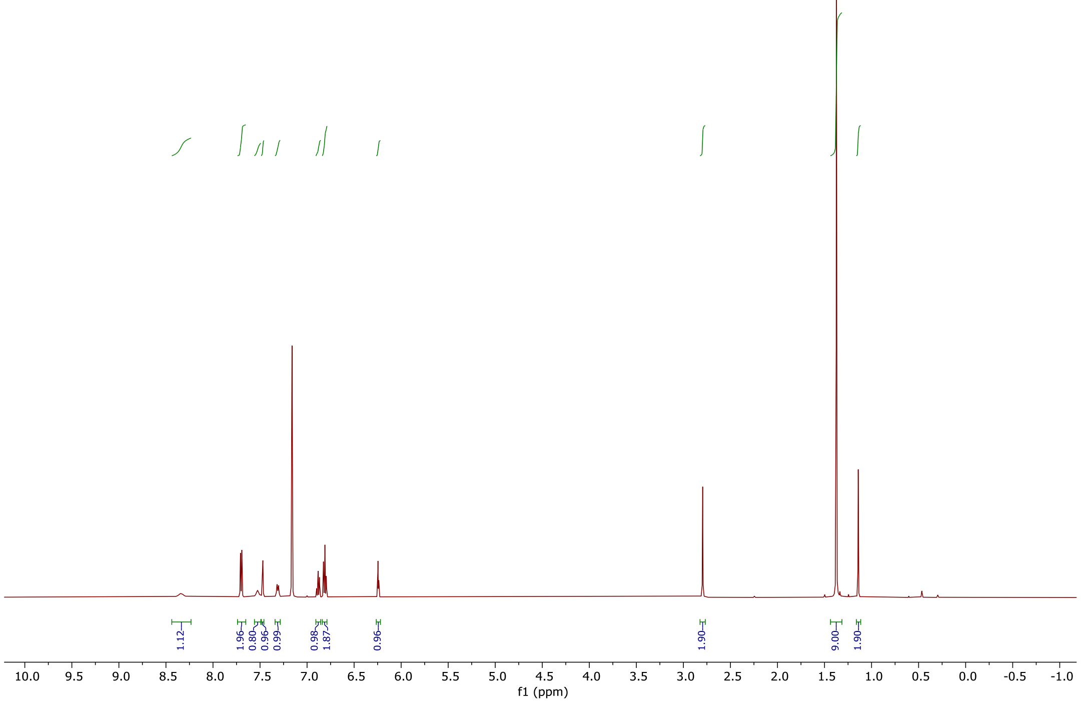

^{13}C NMR (126 MHz, Benzene-*d*)

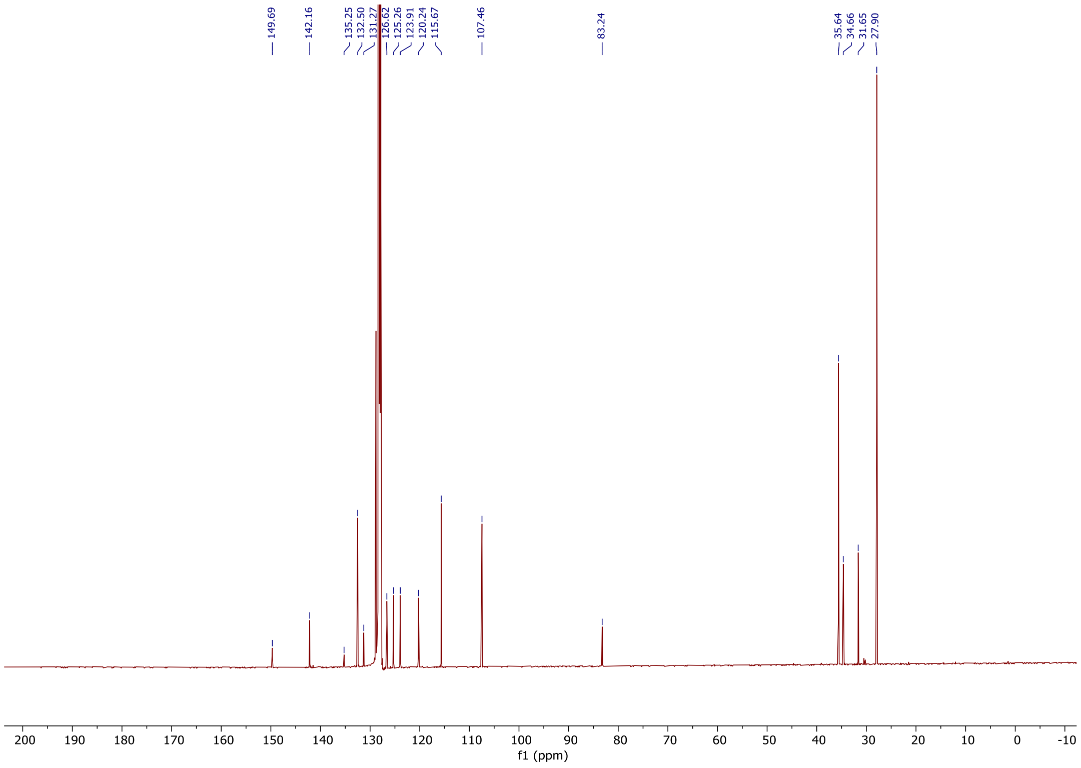

^{1}H COSY (500 MHz, Benzene-*d*)

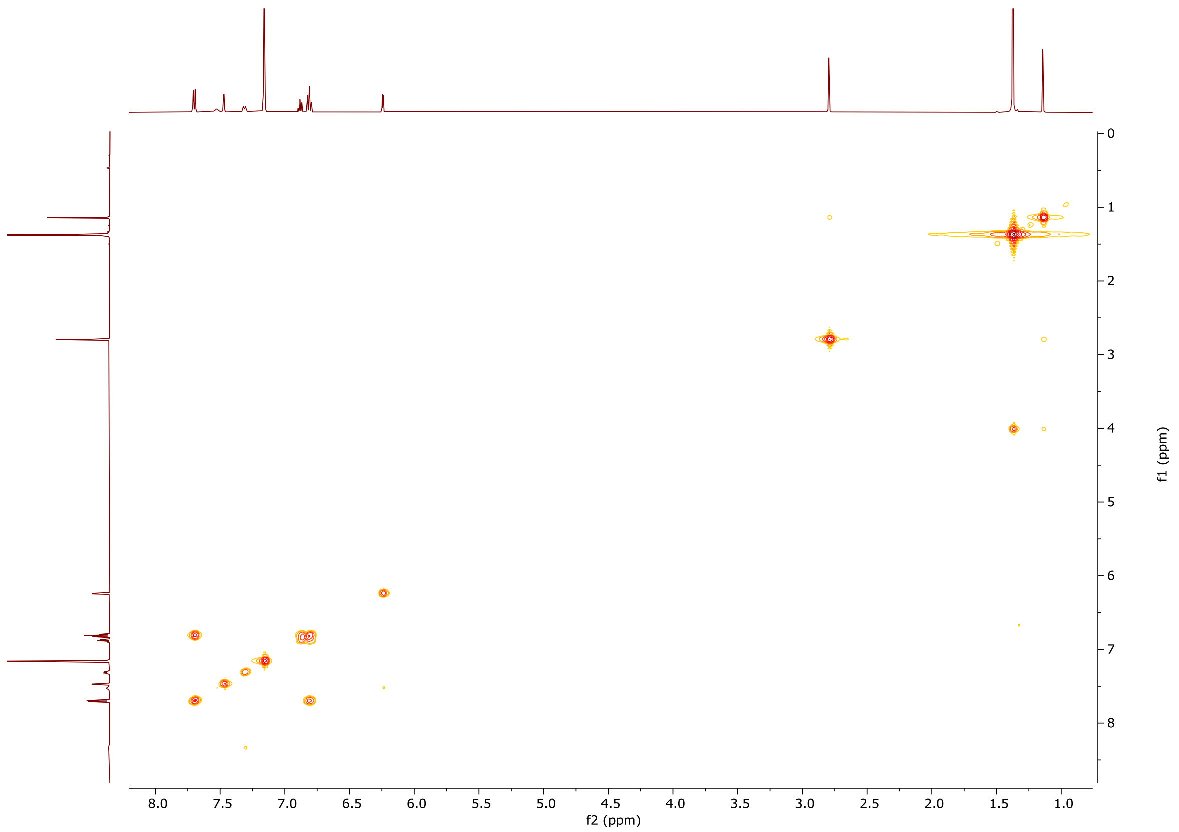

^{1}H/^{13}C HSQC (500/126 MHz, Benzene-*d*)

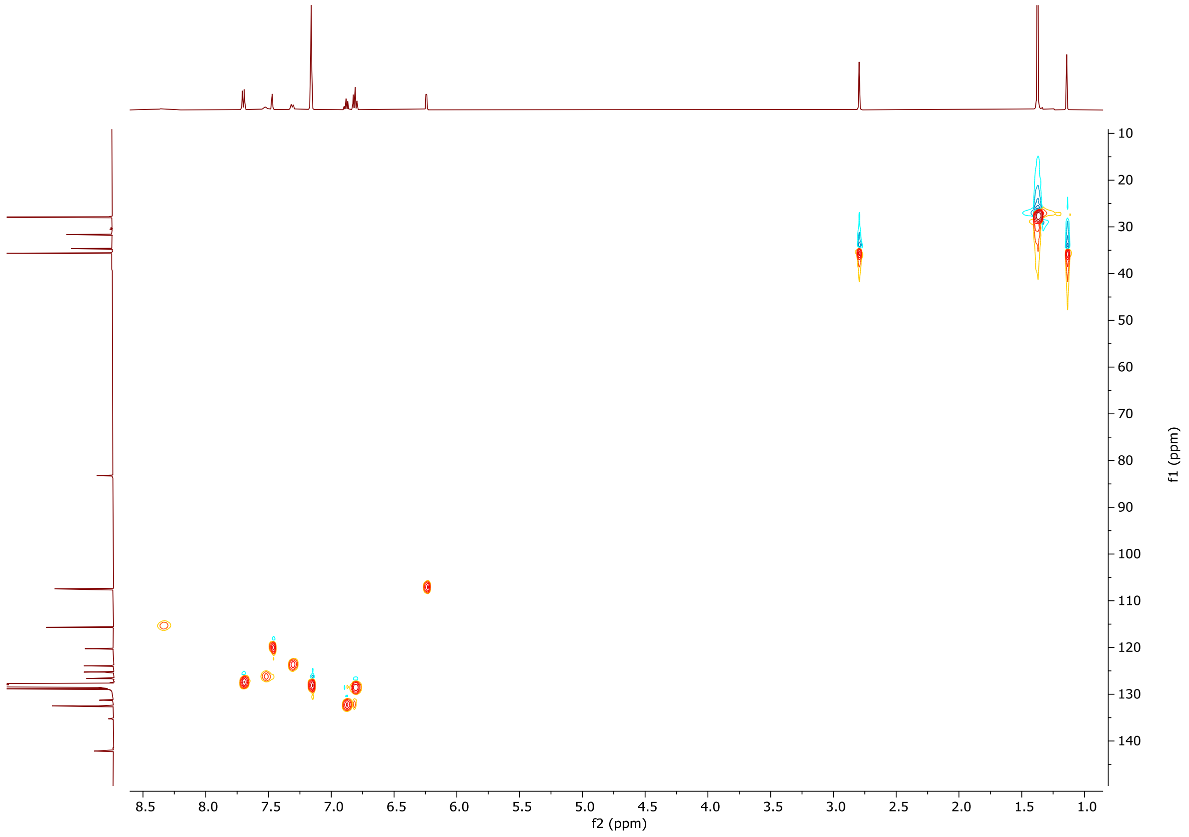

^{1}H/^{13}C HMBC (500/126 MHz, Benzene -*d*)

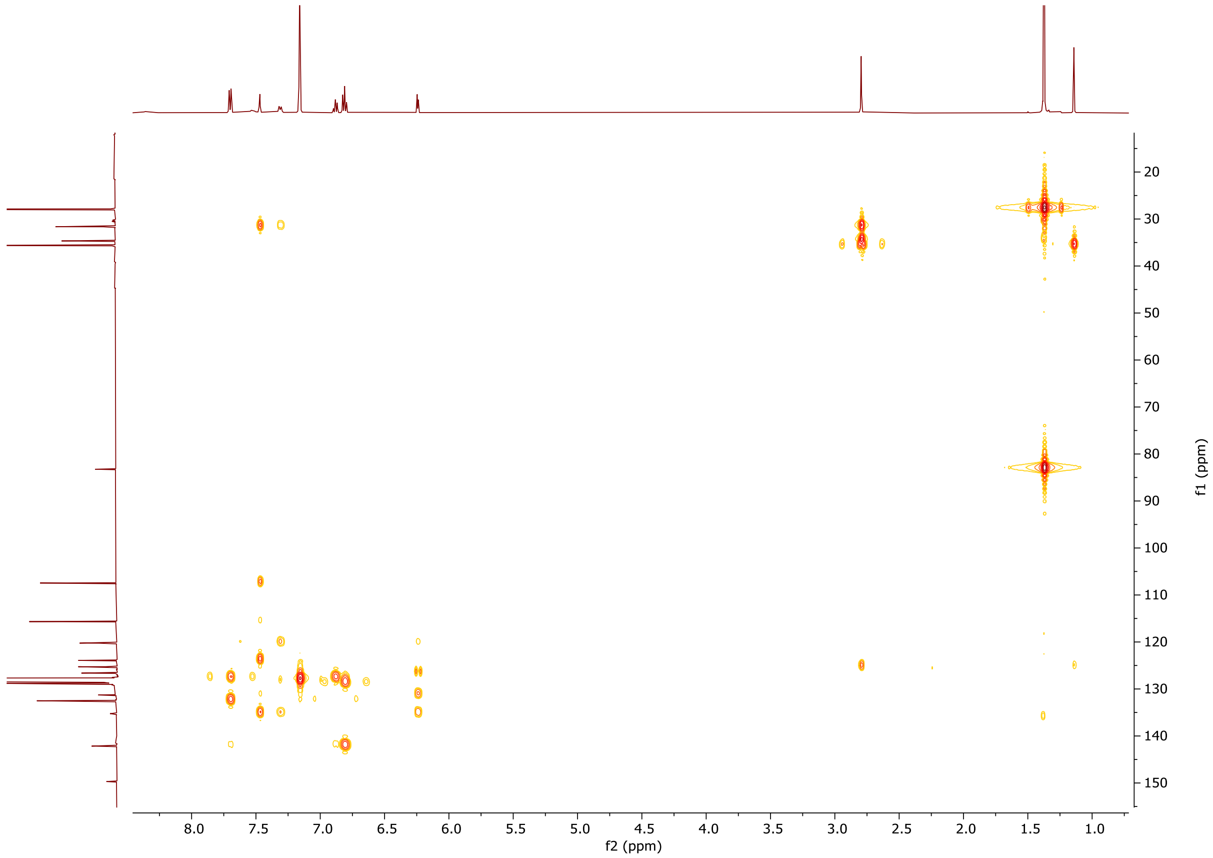

2,6-bis(3-(phenylsulfonyl)bicyclo[1.1.0]butan-1-yl)pyridine, 38

^{1}H NMR (500 MHz, Chloroform-*d*)

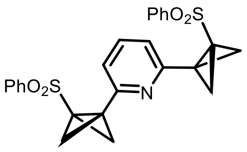

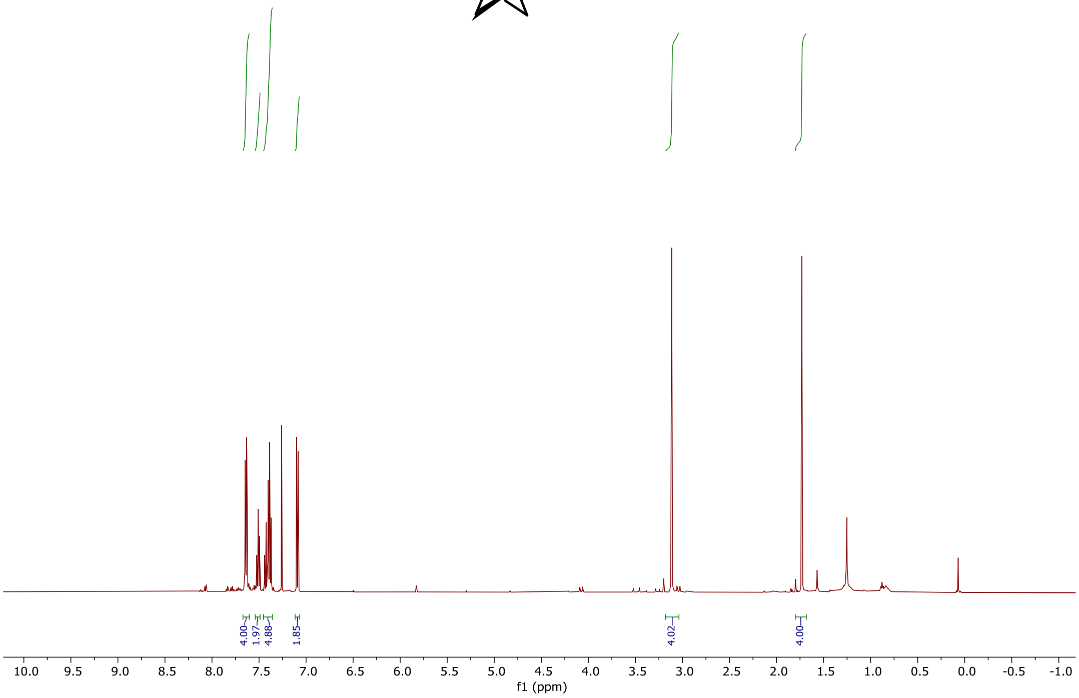

^{13}C NMR (126 MHz, Chloroform-*d*)

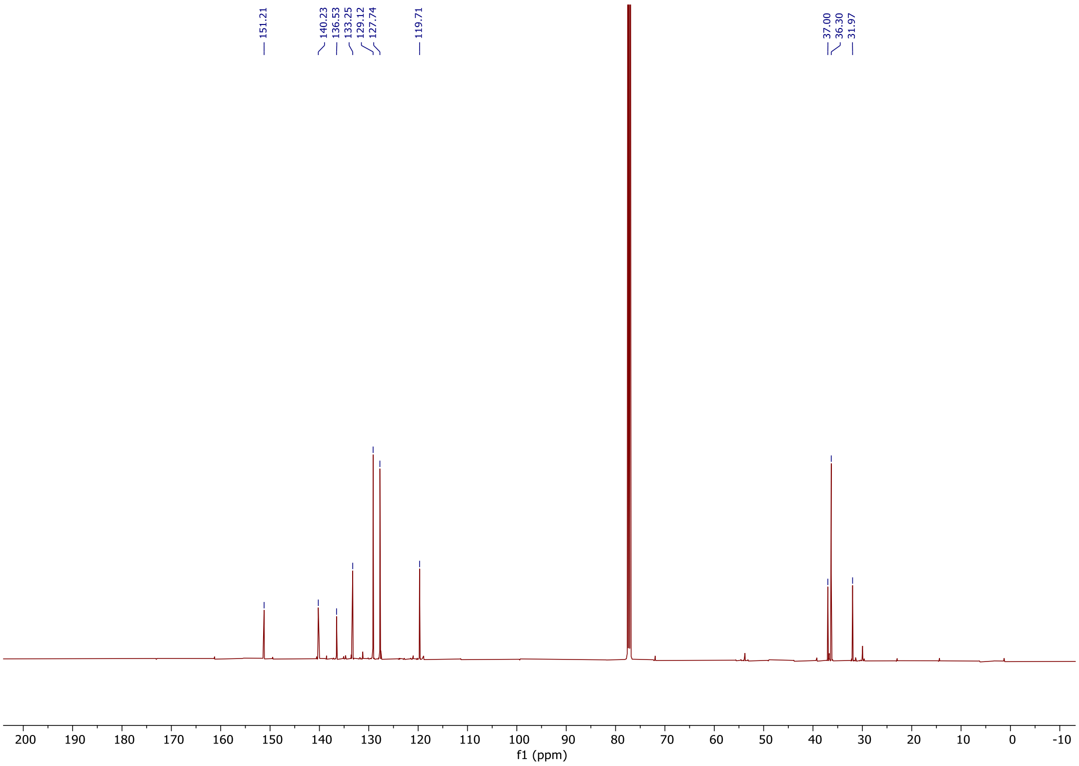

^{1}**H COSY** (500 MHz, Chloroform-*d*)

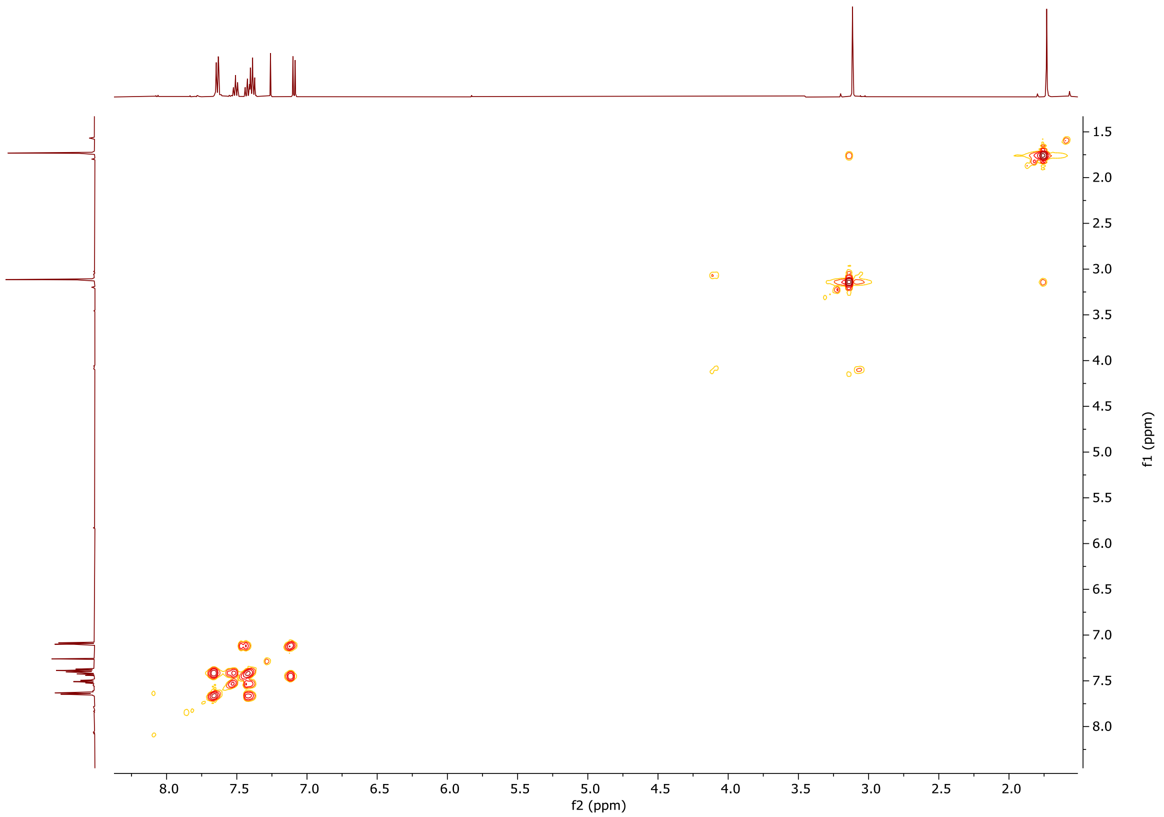

^{1}**H/**^{13}**C HSQC** (500/126 MHz, Chloroform-*d*)

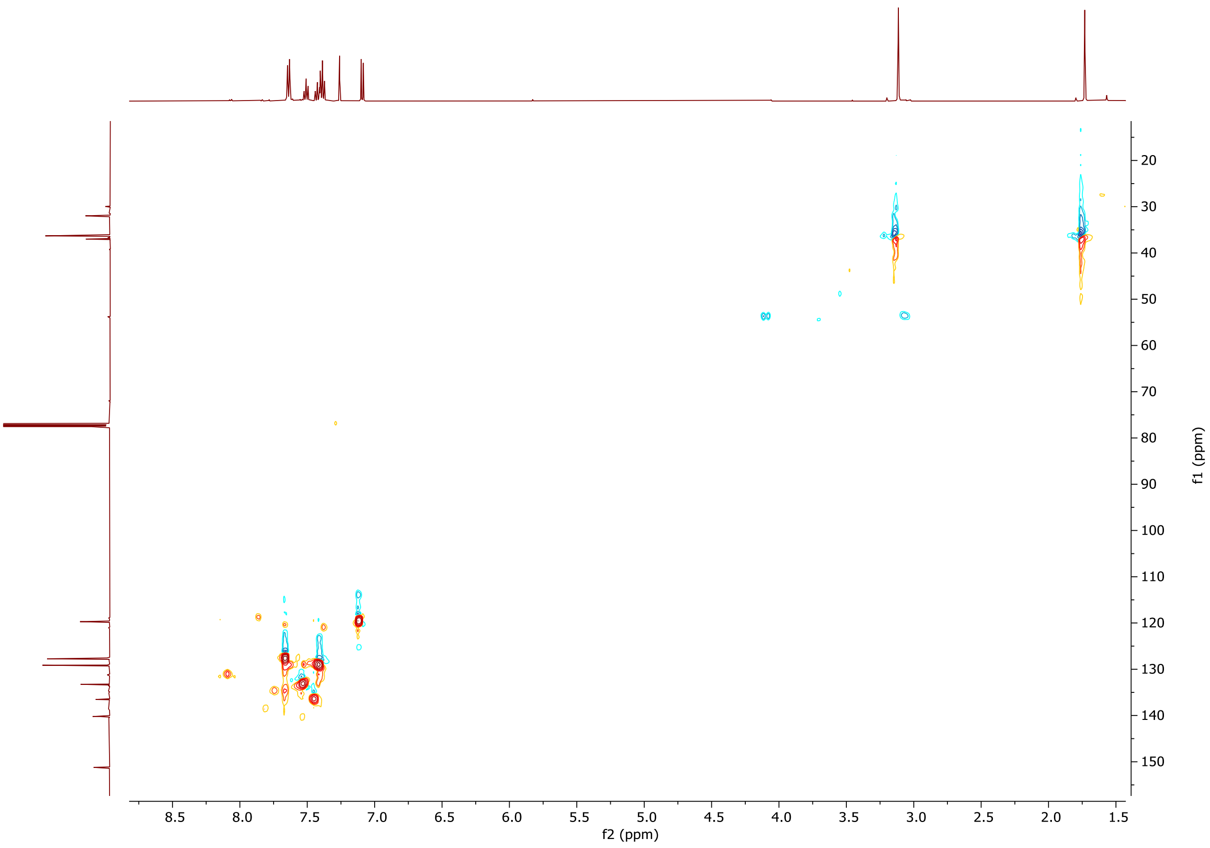

¹H/¹³C HMBC (500/126 MHz, Chloroform-*d*)

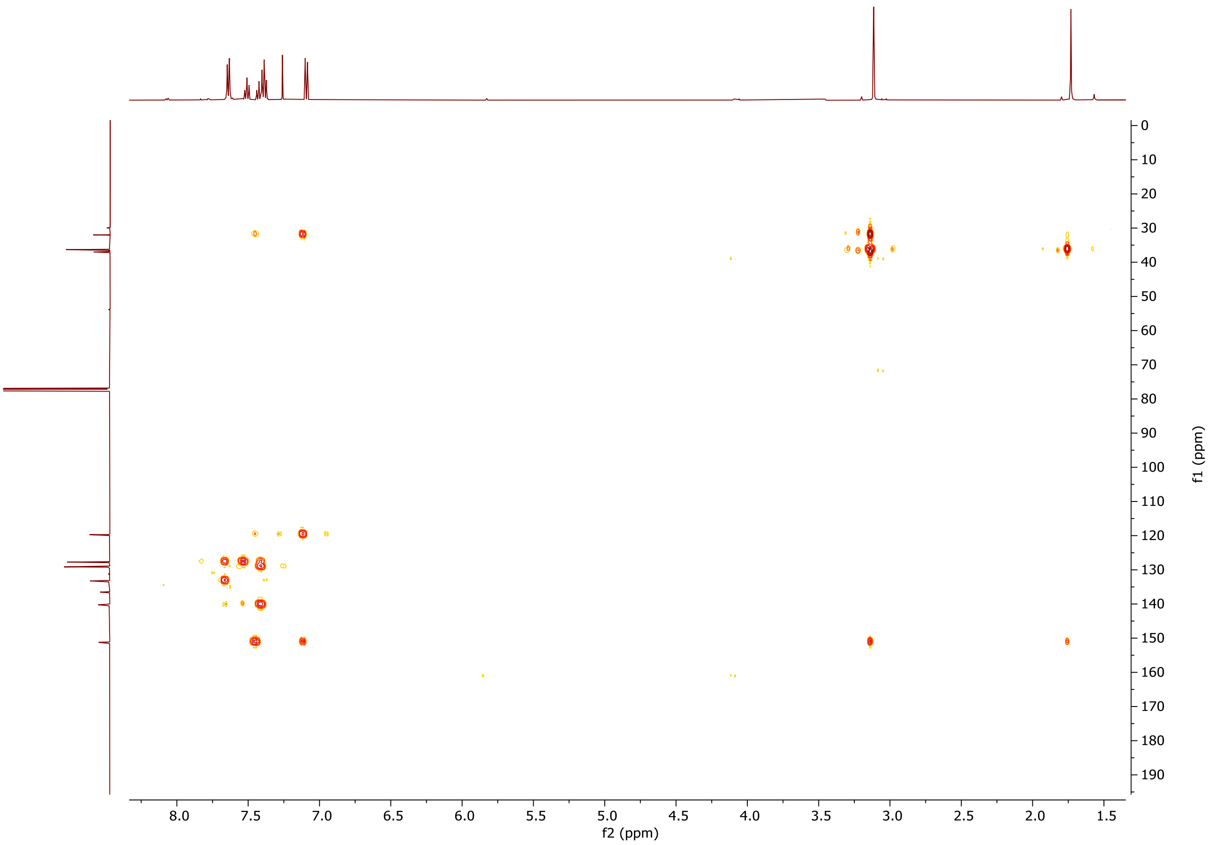

***N*,*N*-diisopropyl-3-phenylbicyclo[1.1.0]butane-1-carboxamide, 39**

^{1}H NMR (400 MHz, Chloroform-*d*)

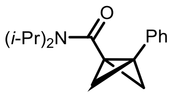

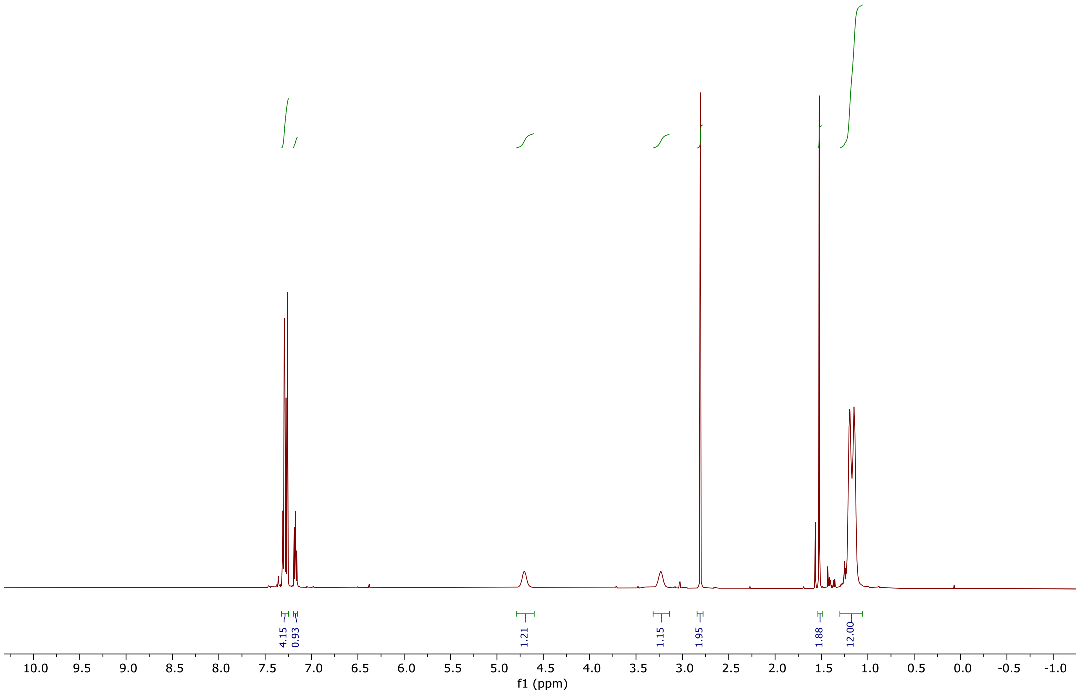

^{13}C NMR (126 MHz, Chloroform-*d*)

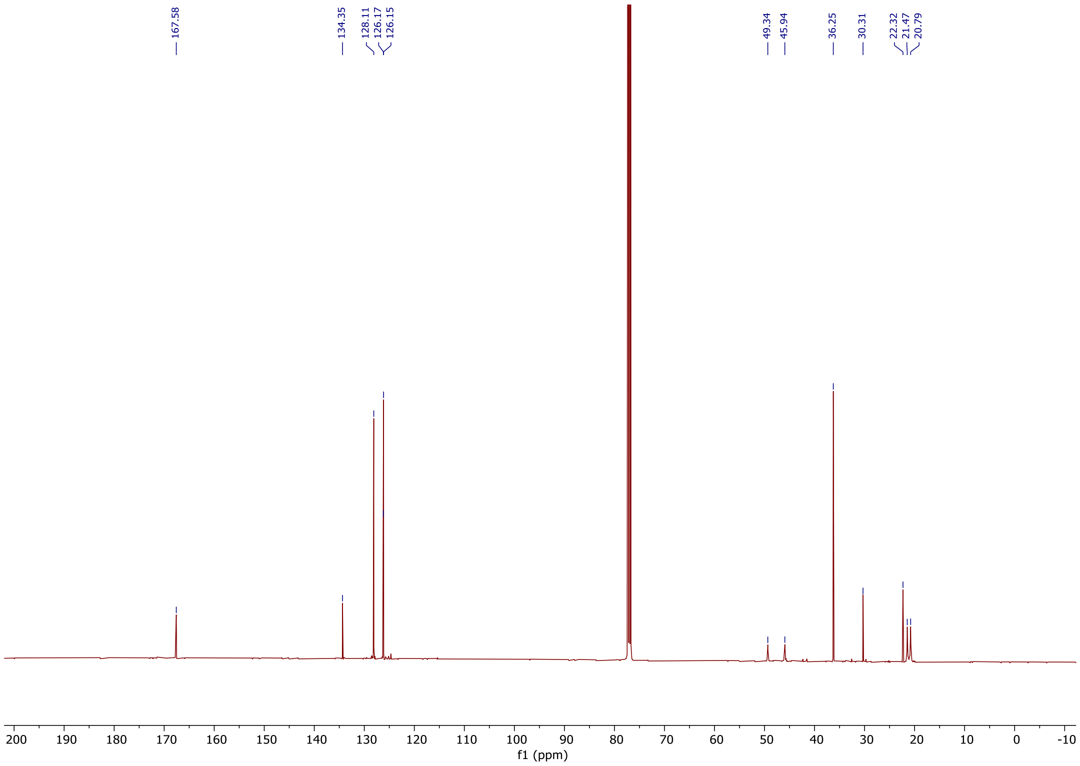

^{1}H COSY (500 MHz, Chloroform-*d*)

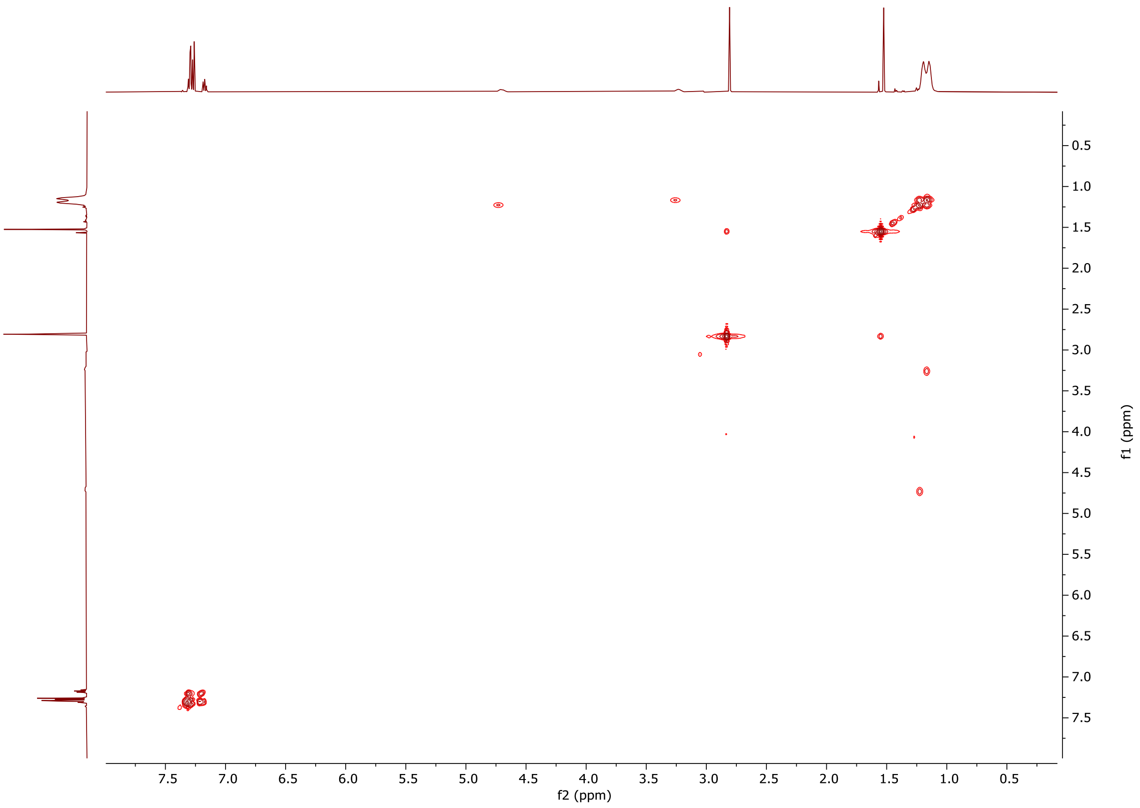

^{1}H/^{13}C HSQC (500/126 MHz, Chloroform-*d*)

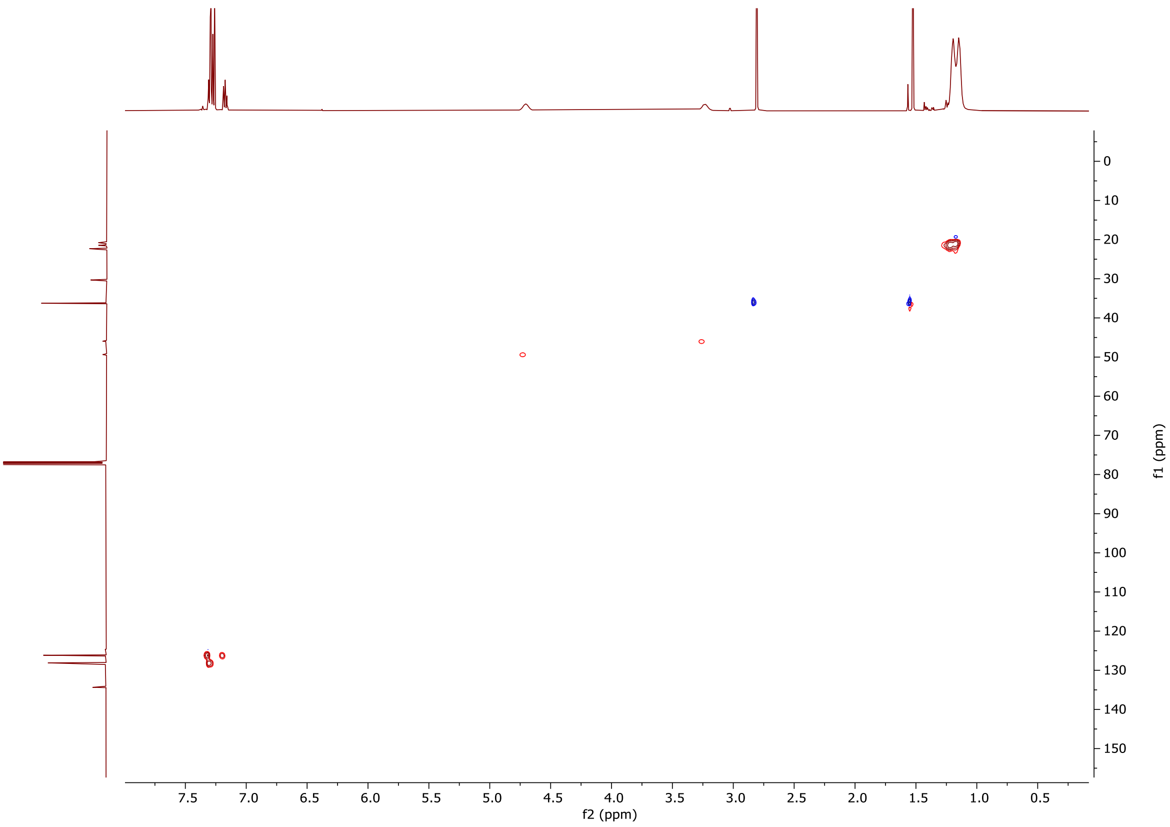

$^{1}H/^{13}C$ HMBC (500/126 MHz, Chloroform-*d*)

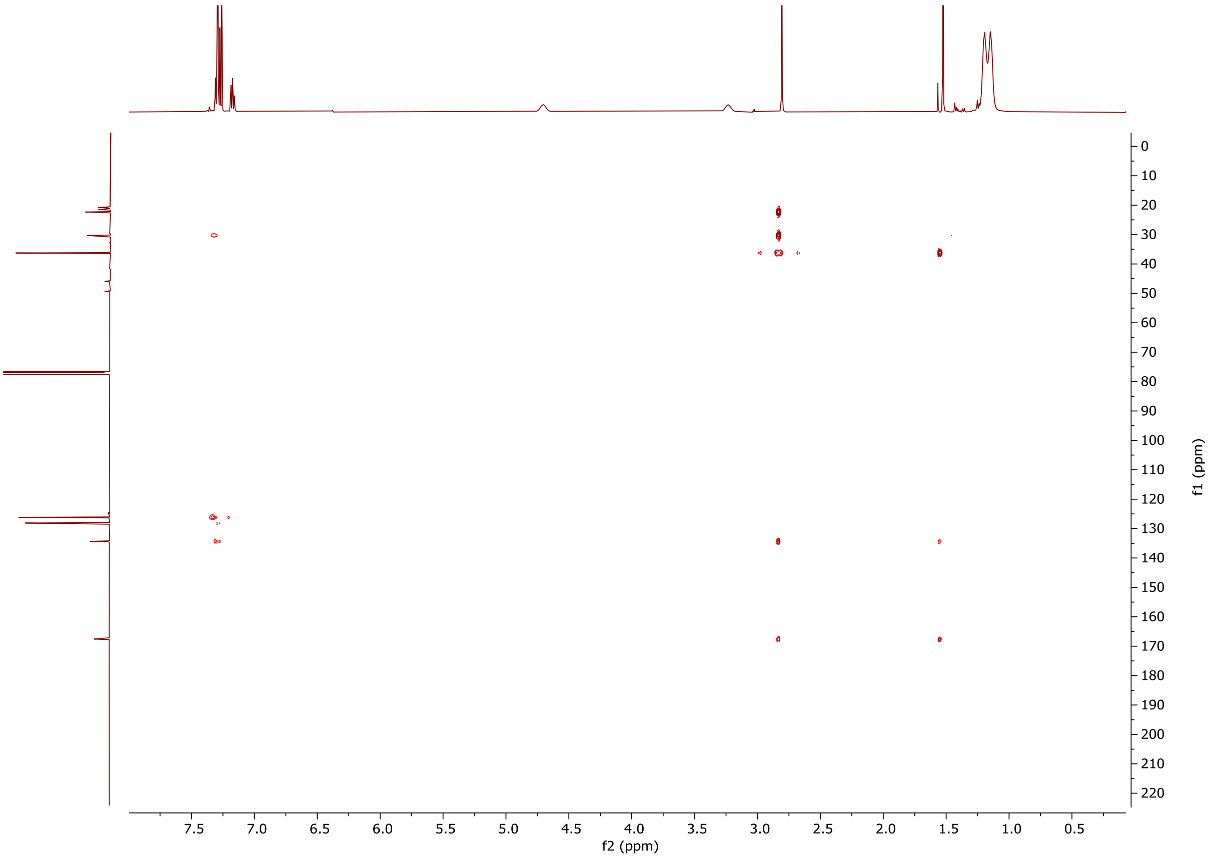

***N*,*N*-diisopropyl-3-(4-(trifluoromethyl)phenyl)bicyclo[1.1.0]butane-1-carboxamide, 40**

^{1}H NMR (500 MHz, Chloroform-*d*)

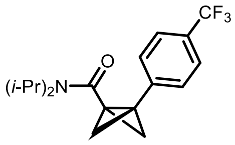

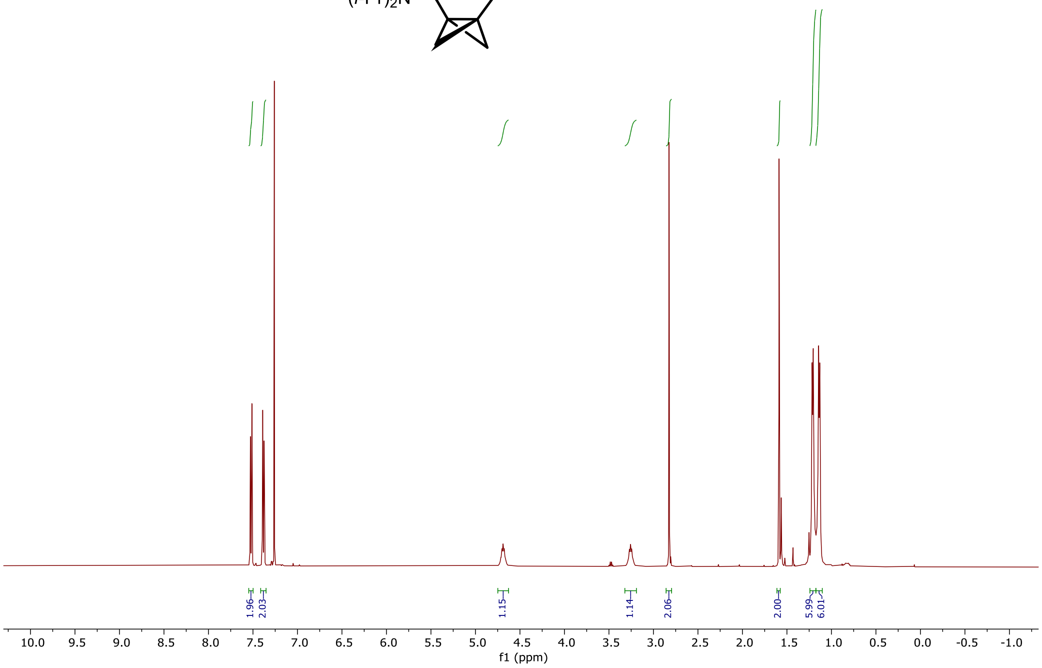

^{13}C NMR (126 MHz, Chloroform-*d*)

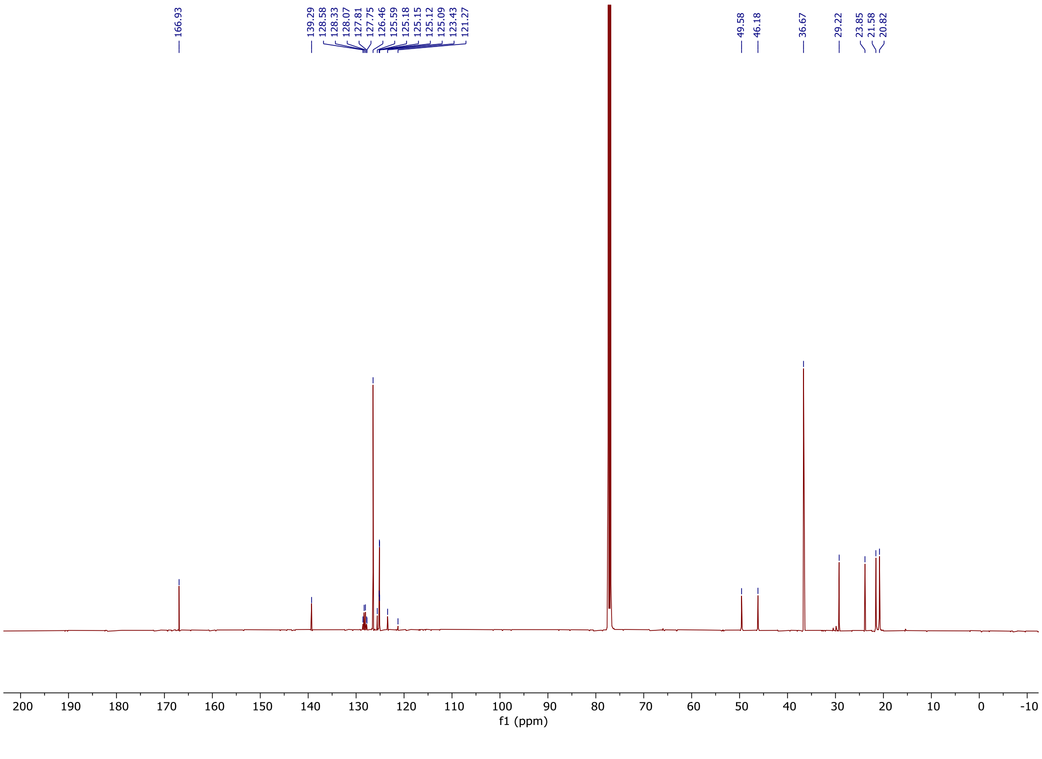

¹H COSY (500 MHz, Chloroform-*d*)

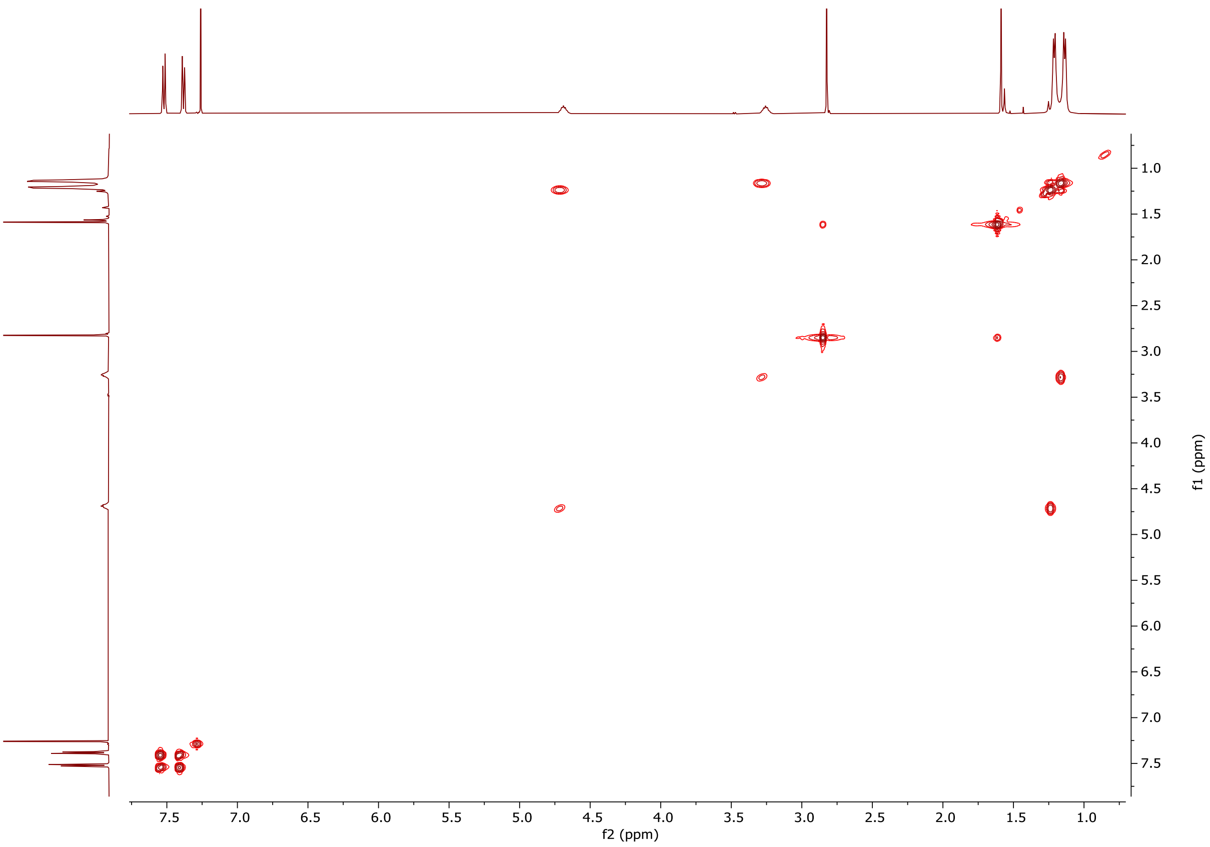

¹H/¹³C HSQC (500/126 MHz, Chloroform-*d*)

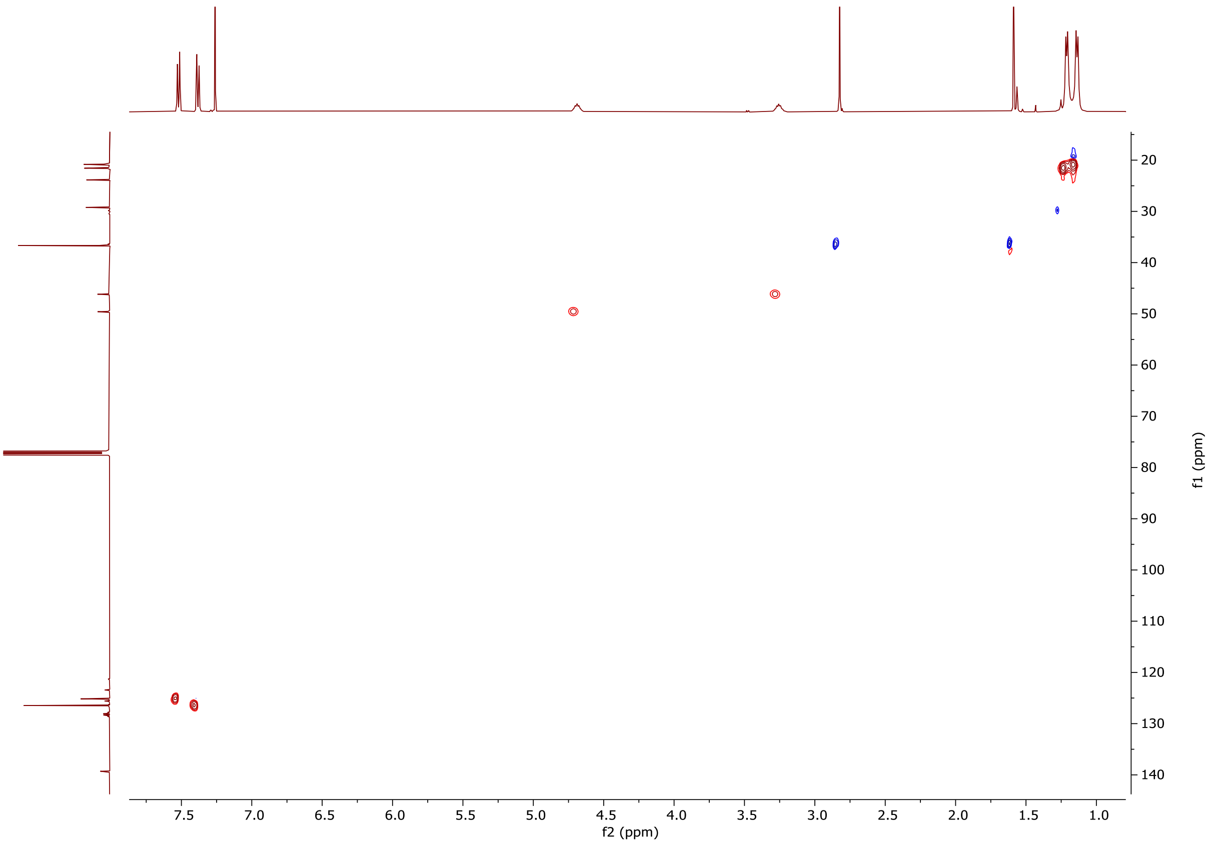

$^1H/^{13}C$ HMBC (500/126 MHz, Chloroform-*d*)

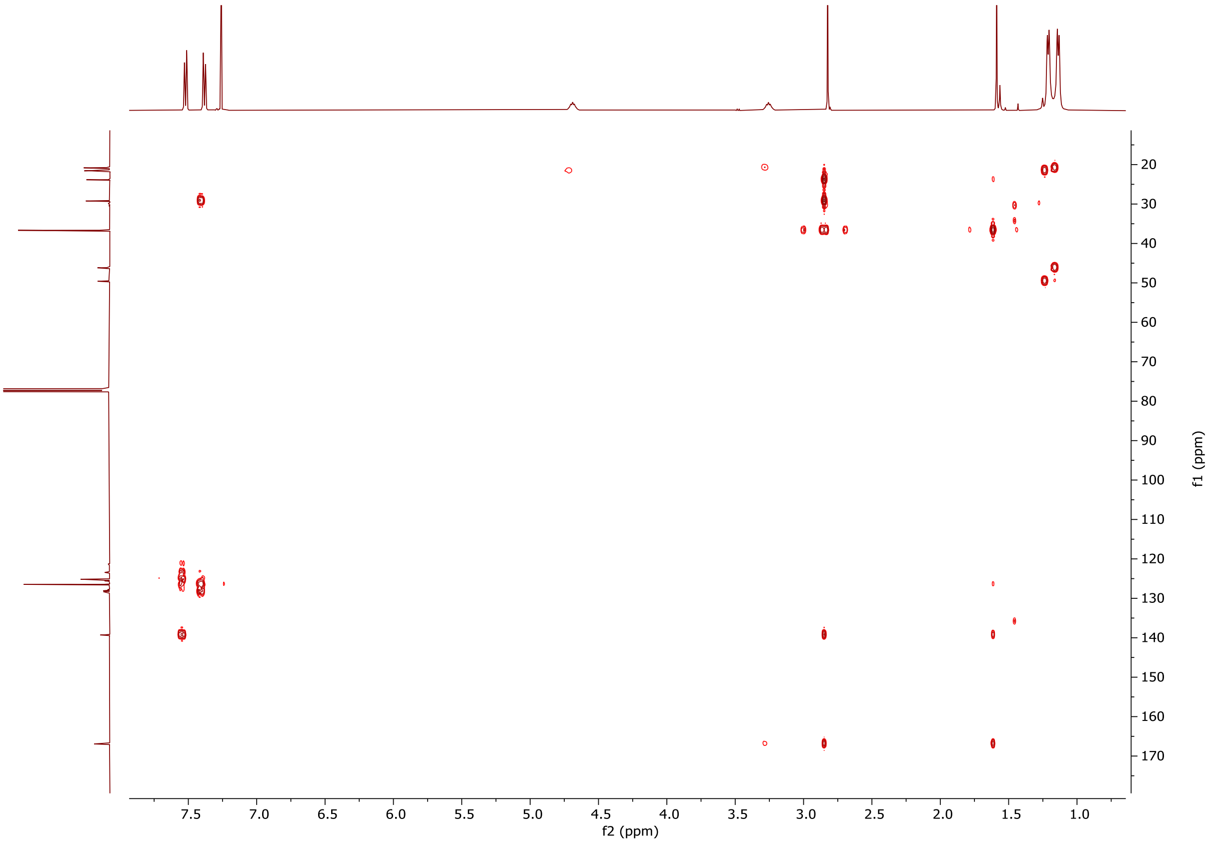

^{19}F NMR (471 MHz, Chloroform-*d*)

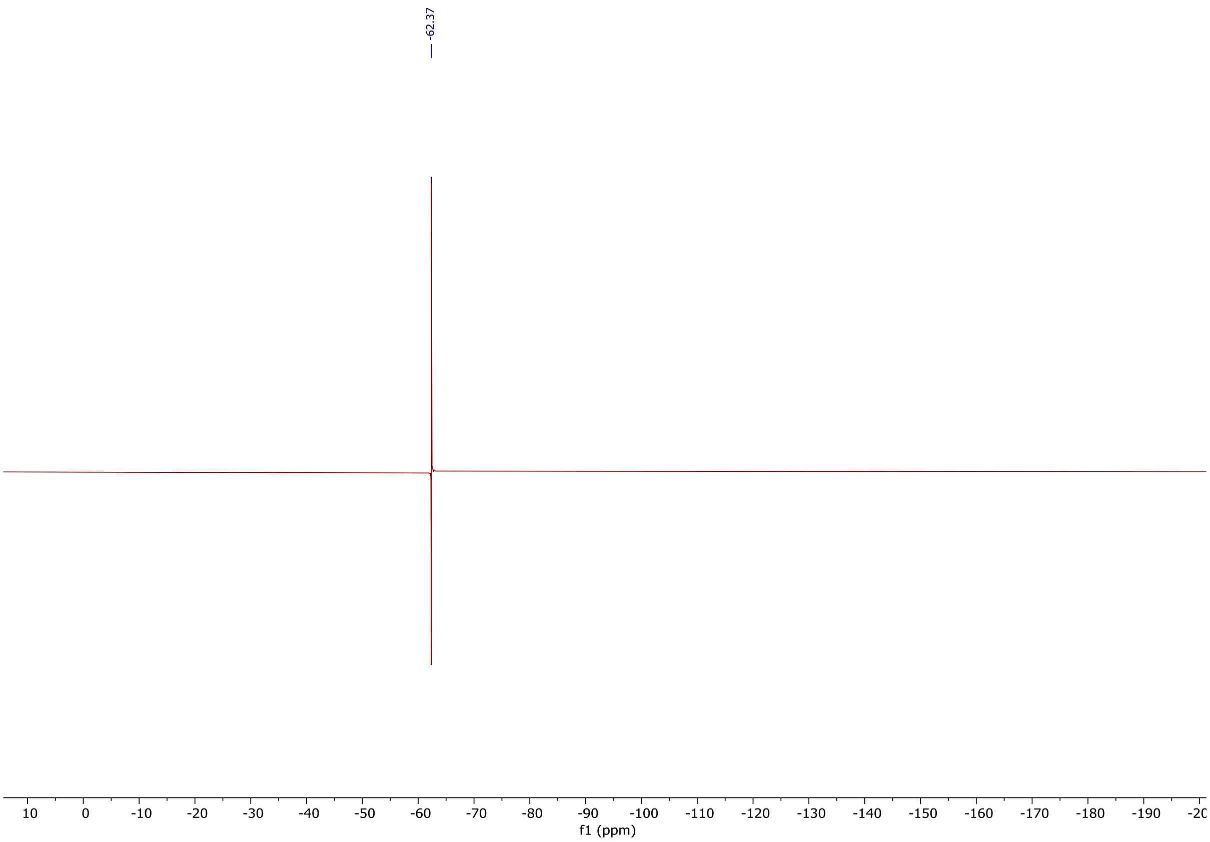

1-phenyl-3-((4-(trifluoromethyl)phenyl)sulfonyl)bicyclo[1.1.0]butane, 41

^{1}H NMR (400 MHz, Benzene-*d*)

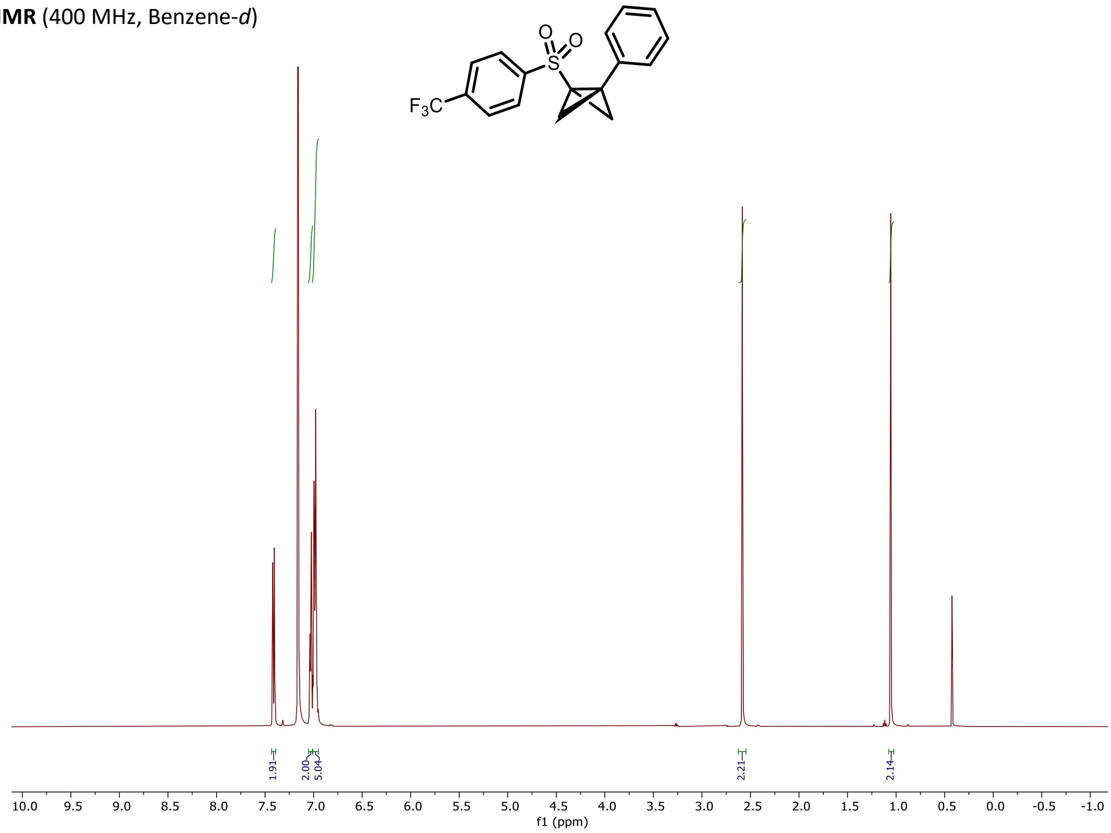

^{13}C NMR (126 MHz, Benzene-*d*)

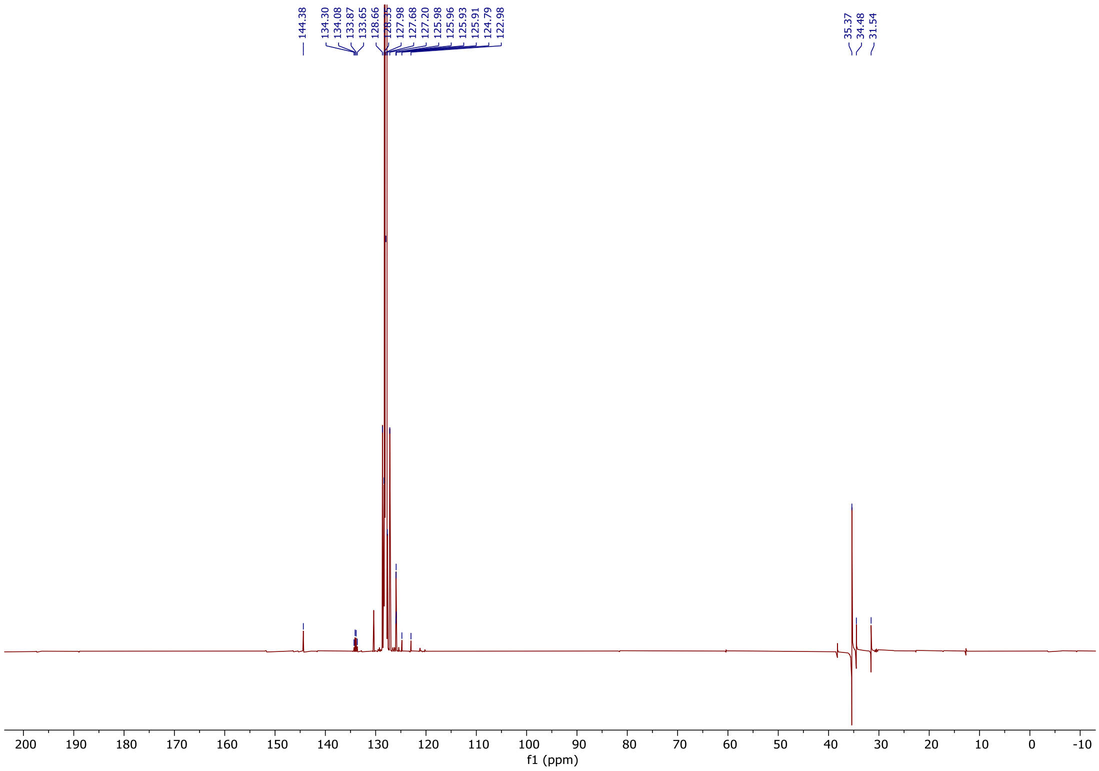

^{1}H COSY (500 MHz, Benzene-*d*)

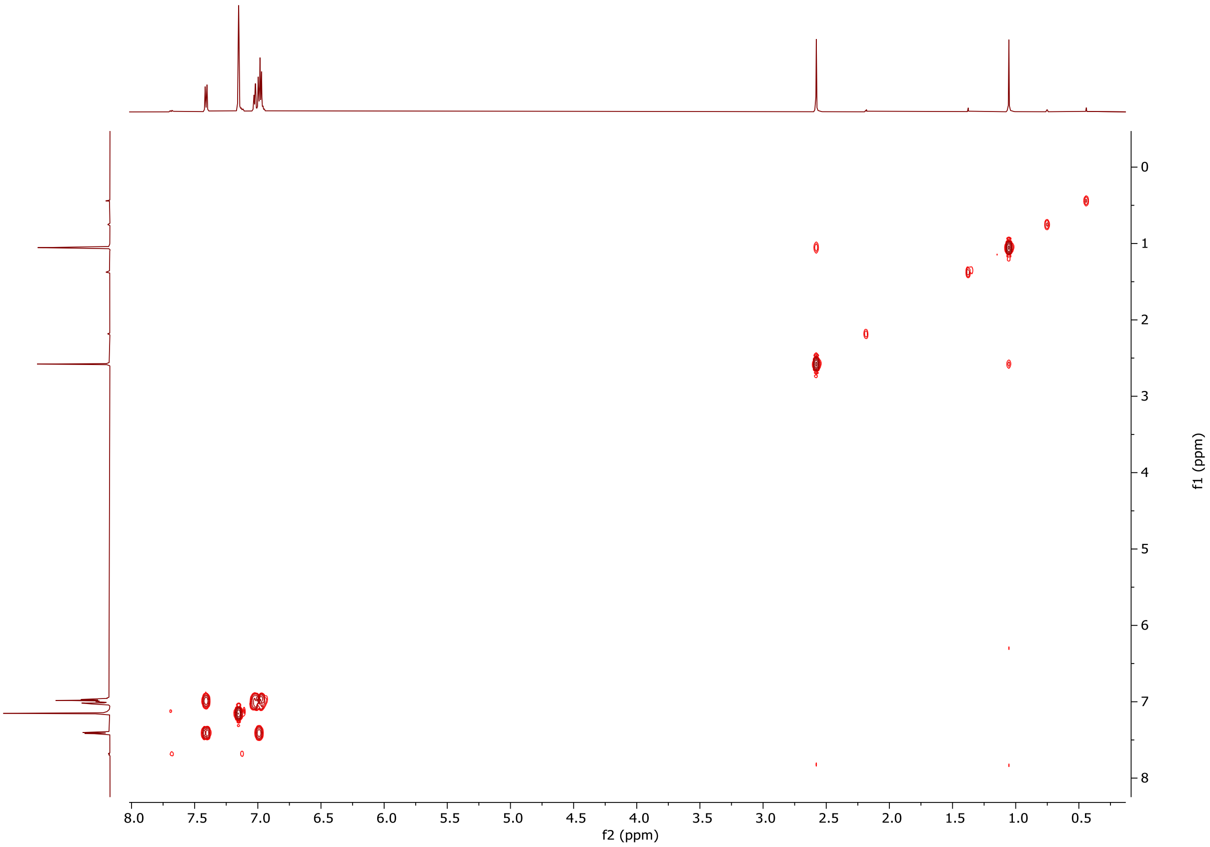

^{1}H/^{13}C HMBC (500/126 MHz, Benzene-*d*)

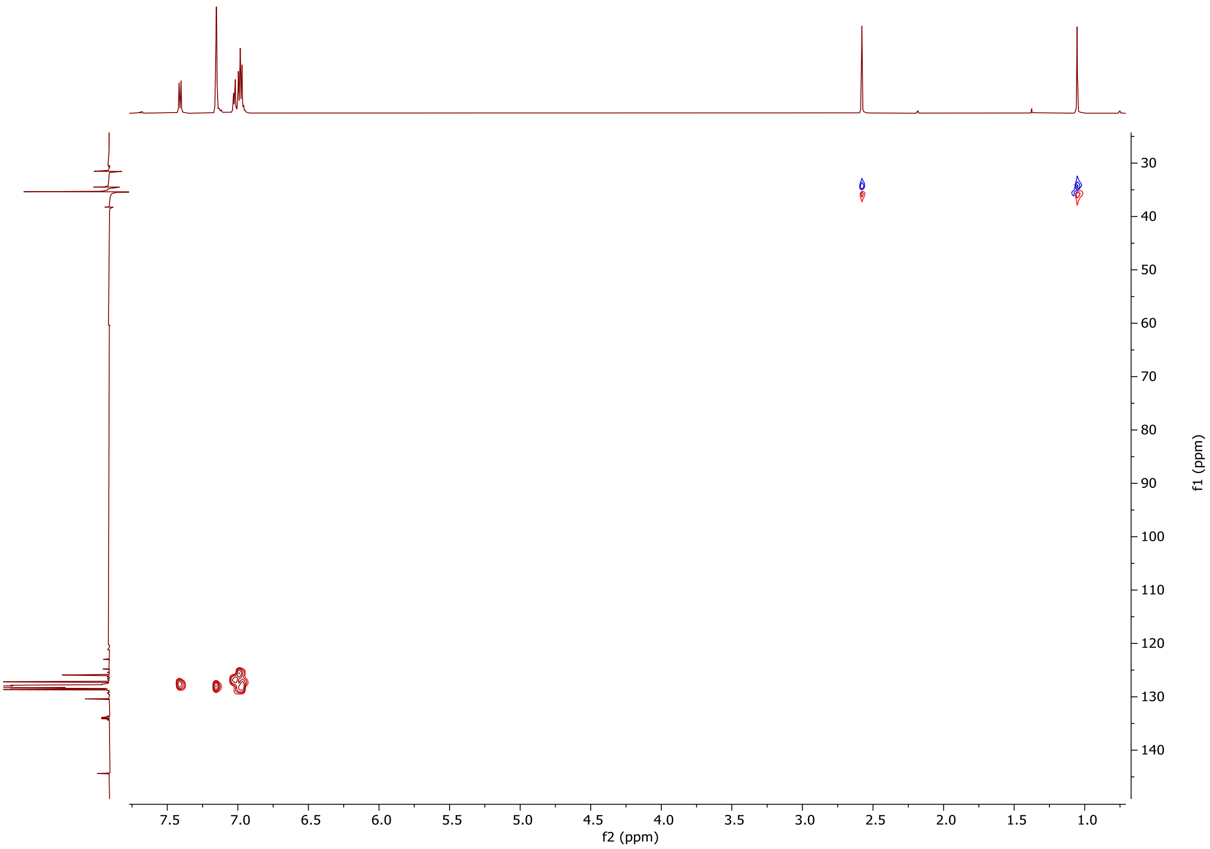

$^1H/^{13}C$ HSQC (500/126 MHz, Benzene-*d*)

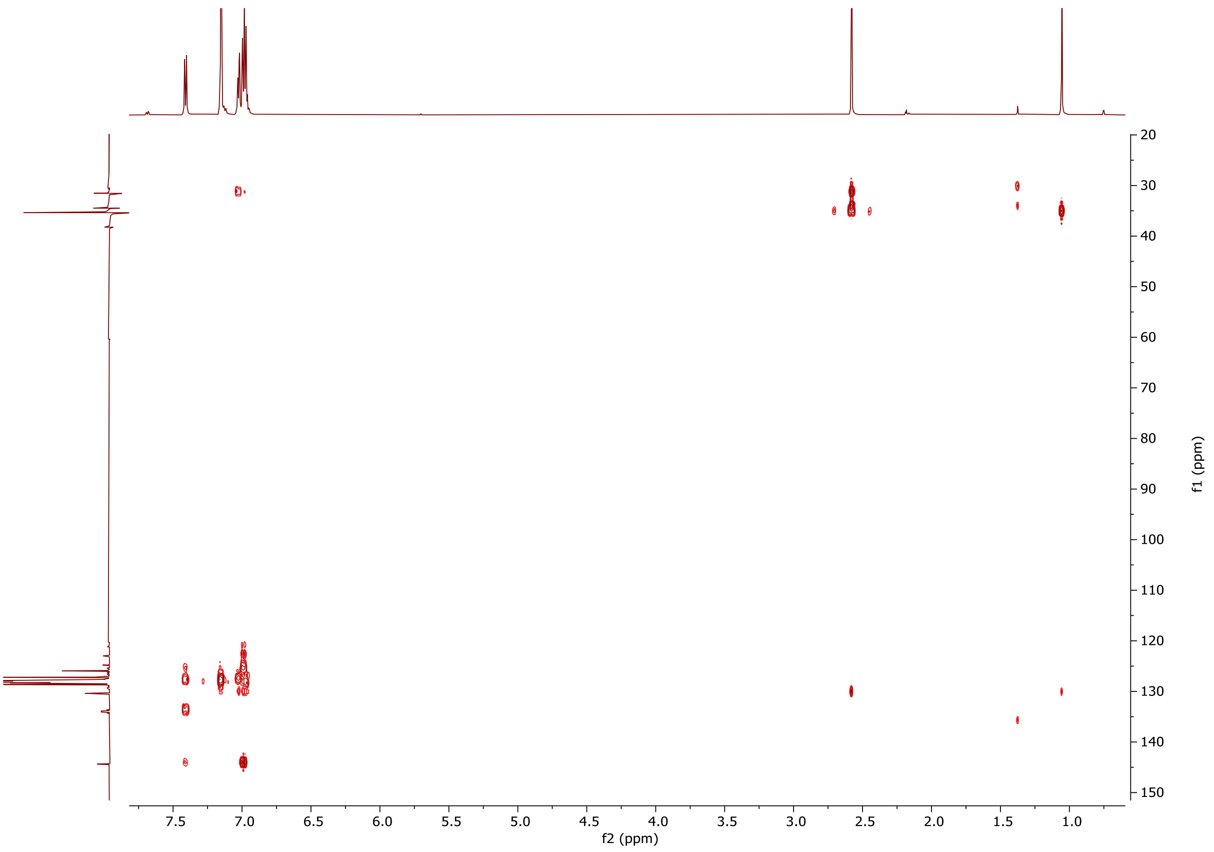

^{19}F NMR (470 MHz, Benzene-*d*)

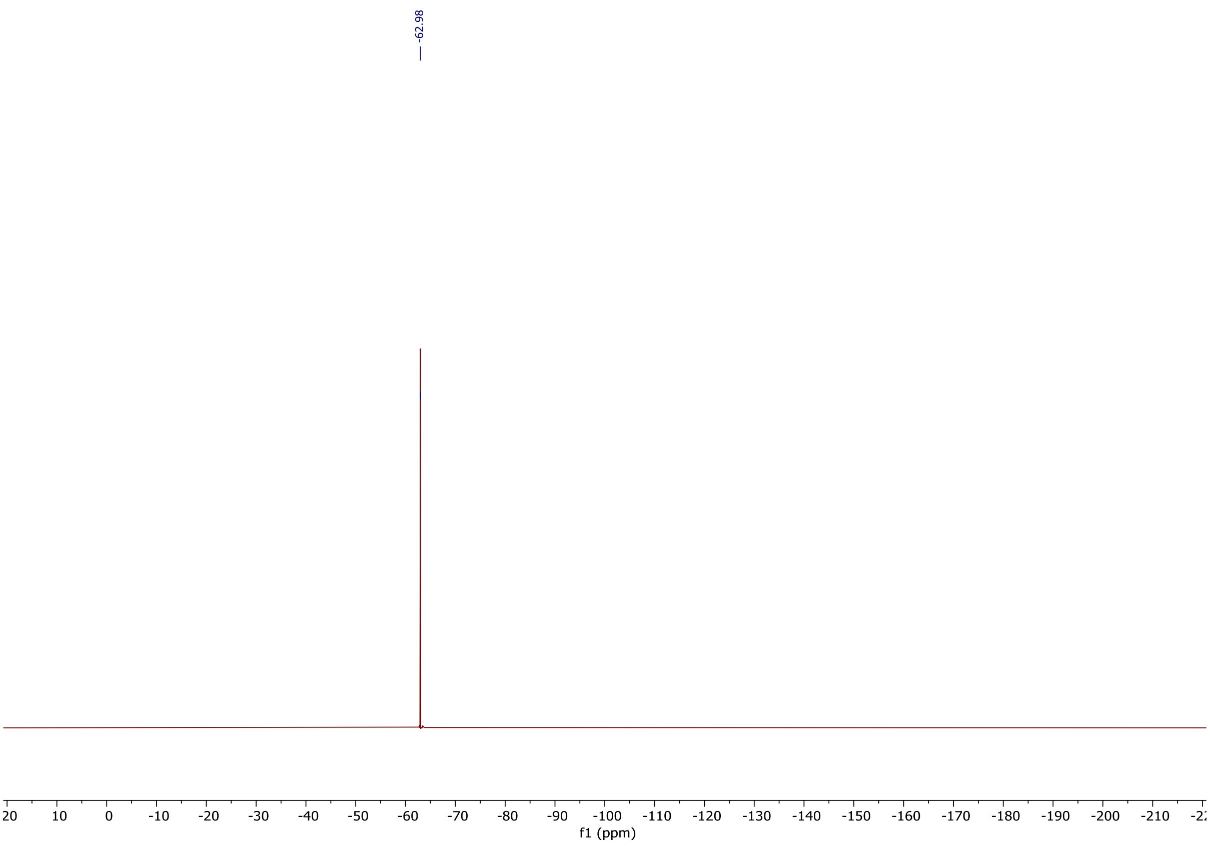

Methyl (2S)-2-((tert-butoxycarbonyl)amino)-3-(4-(3-(phenylsulfonyl)bicyclo[1.1.0]butan-1-yl)phenyl)propanoate, 42

^{1}H NMR (500 MHz, Chloroform-*d*)

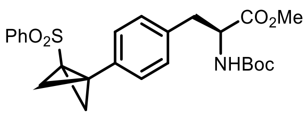

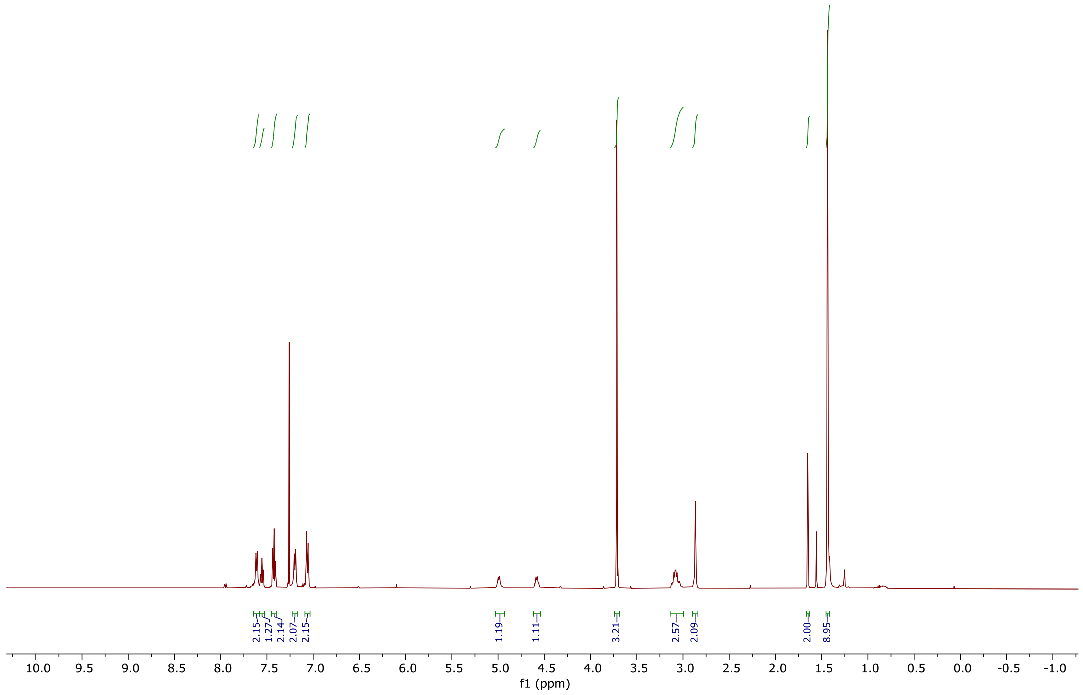

^{13}C NMR (126 MHz, Chloroform-*d*)

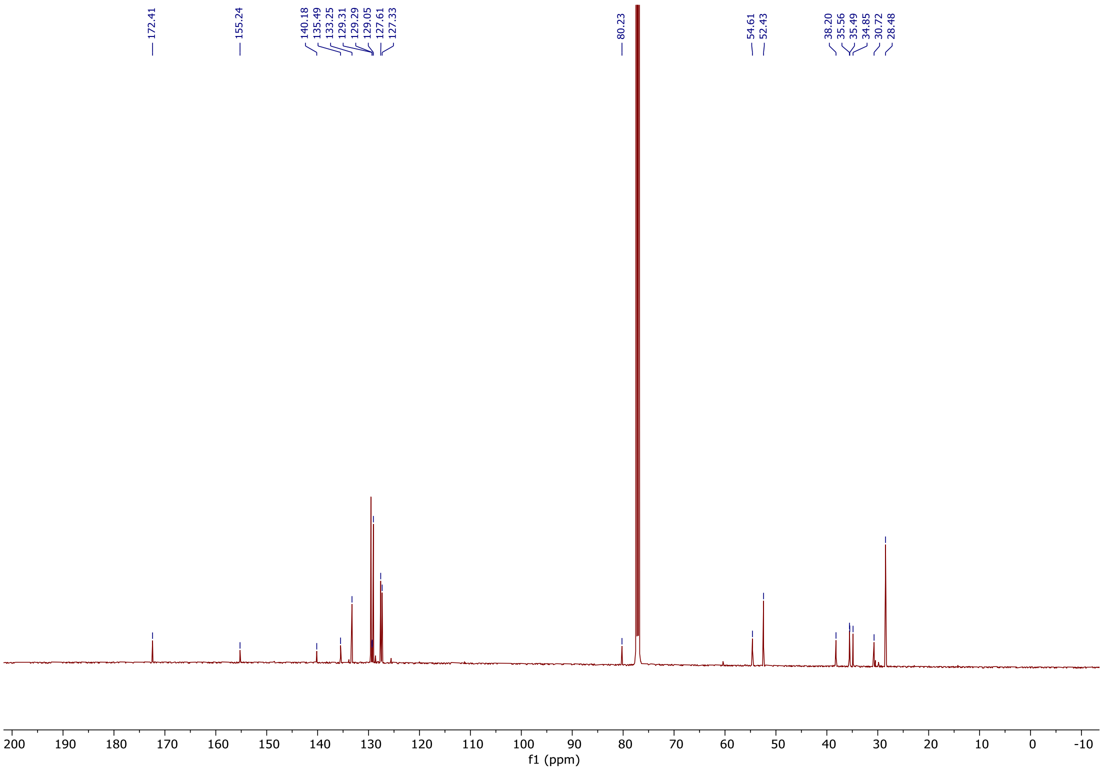

¹H COSY (500 MHz, Chloroform-*d*)

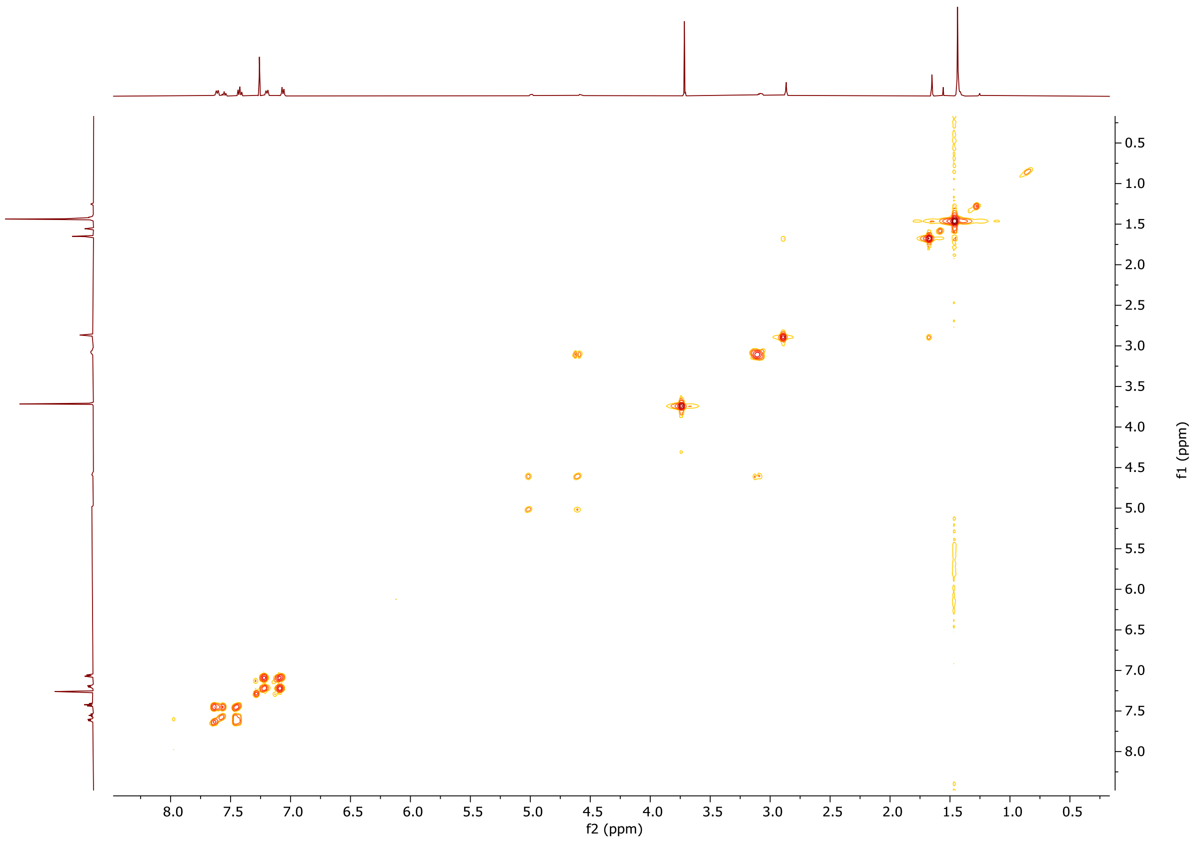

¹H/¹³C HSQC (500/126 MHz, Chloroform-*d*)

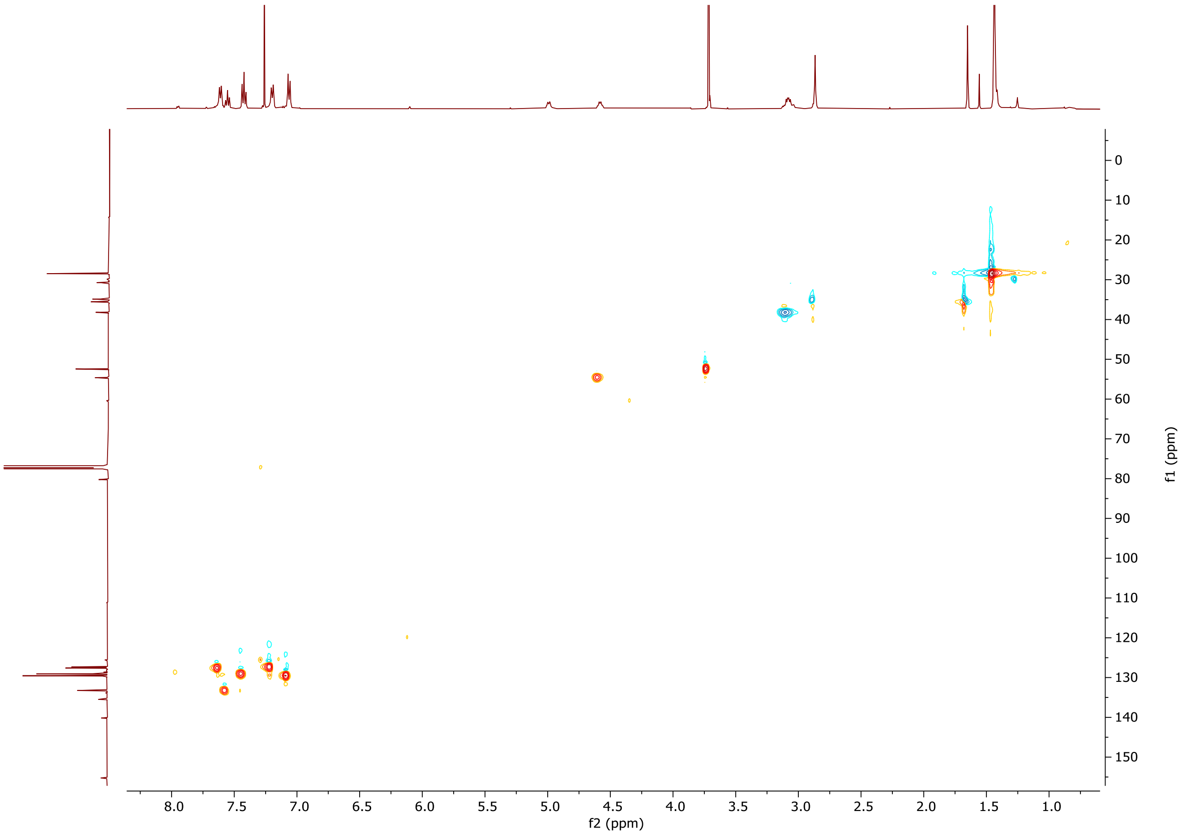

$^{1}H/^{13}C$ HMBC (500/126 MHz, Chloroform-*d*)

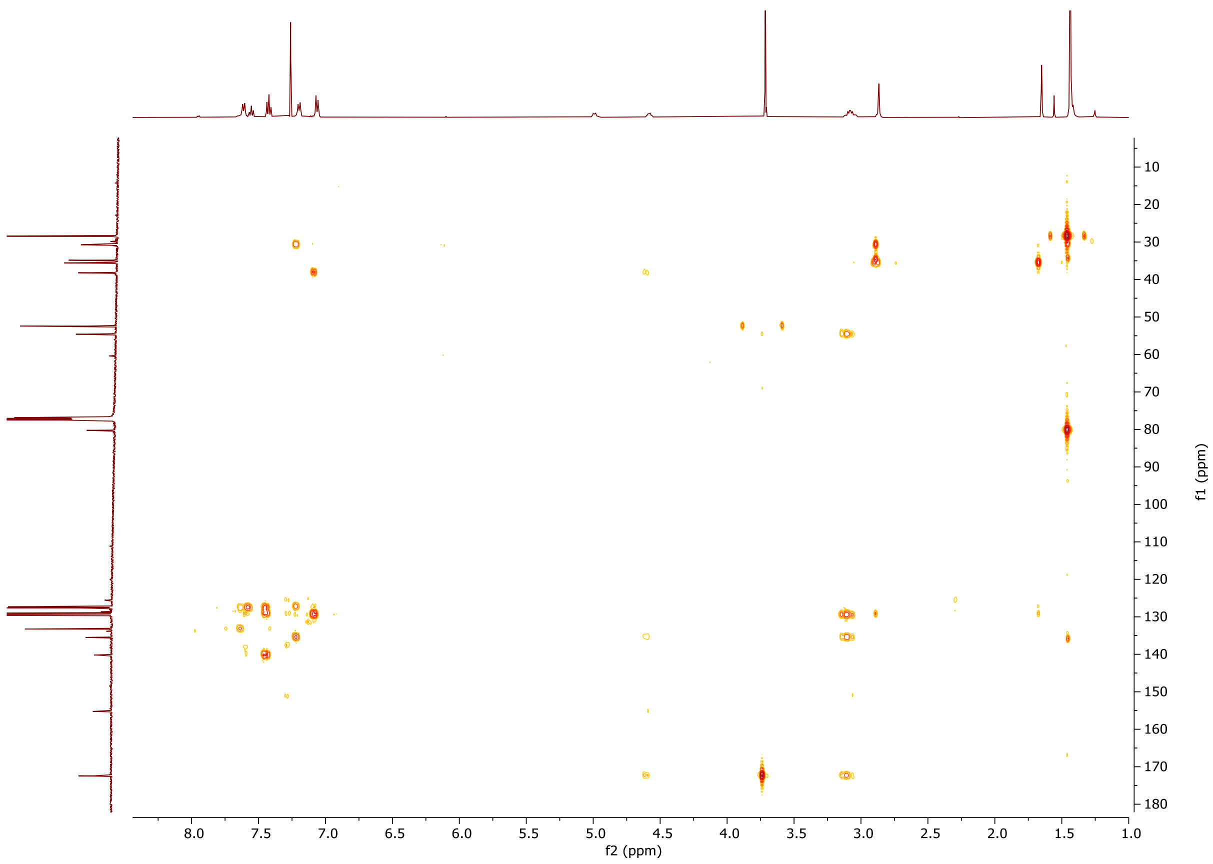

(S)-2-((tert-butoxycarbonyl)amino)-3-methoxy-3-oxopropyl 4-(3-(phenylsulfonyl)bicyclo[1.1.0]butan-1-yl)benzoate, 43

^{1}H NMR (500 MHz, Chloroform-*d*)

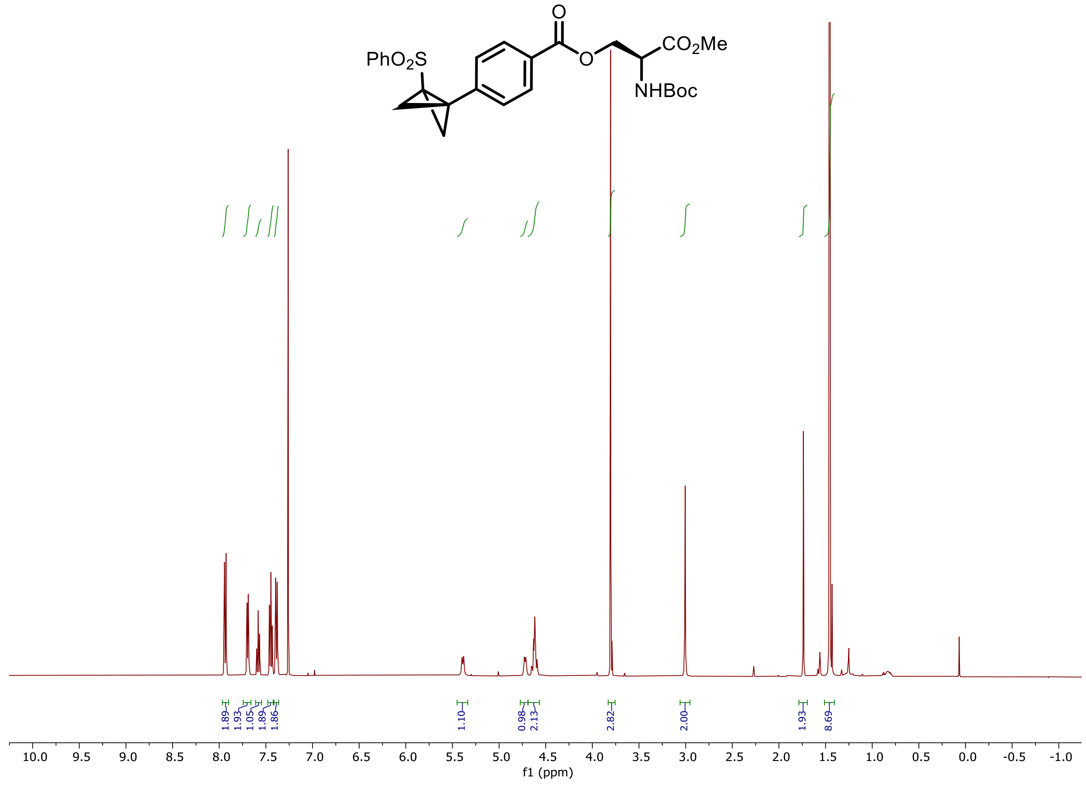

^{13}C NMR (126 MHz, Chloroform-*d*)

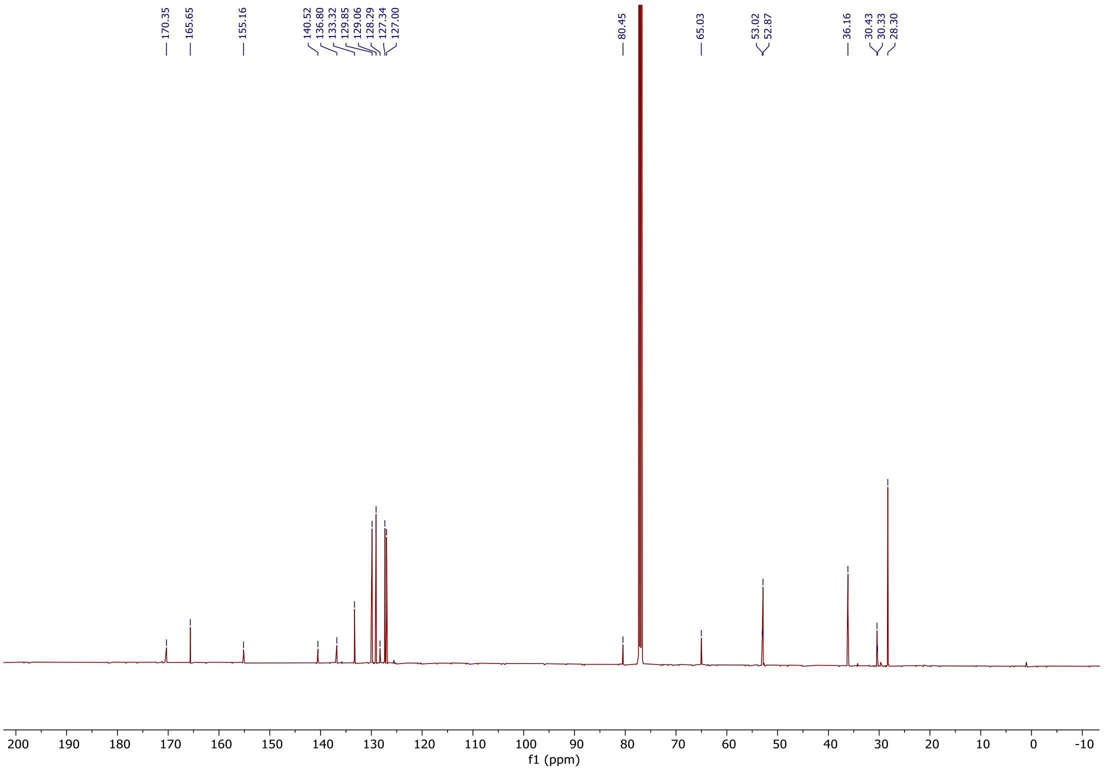

^{1}H COSY (500 MHz, Chloroform-*d*)

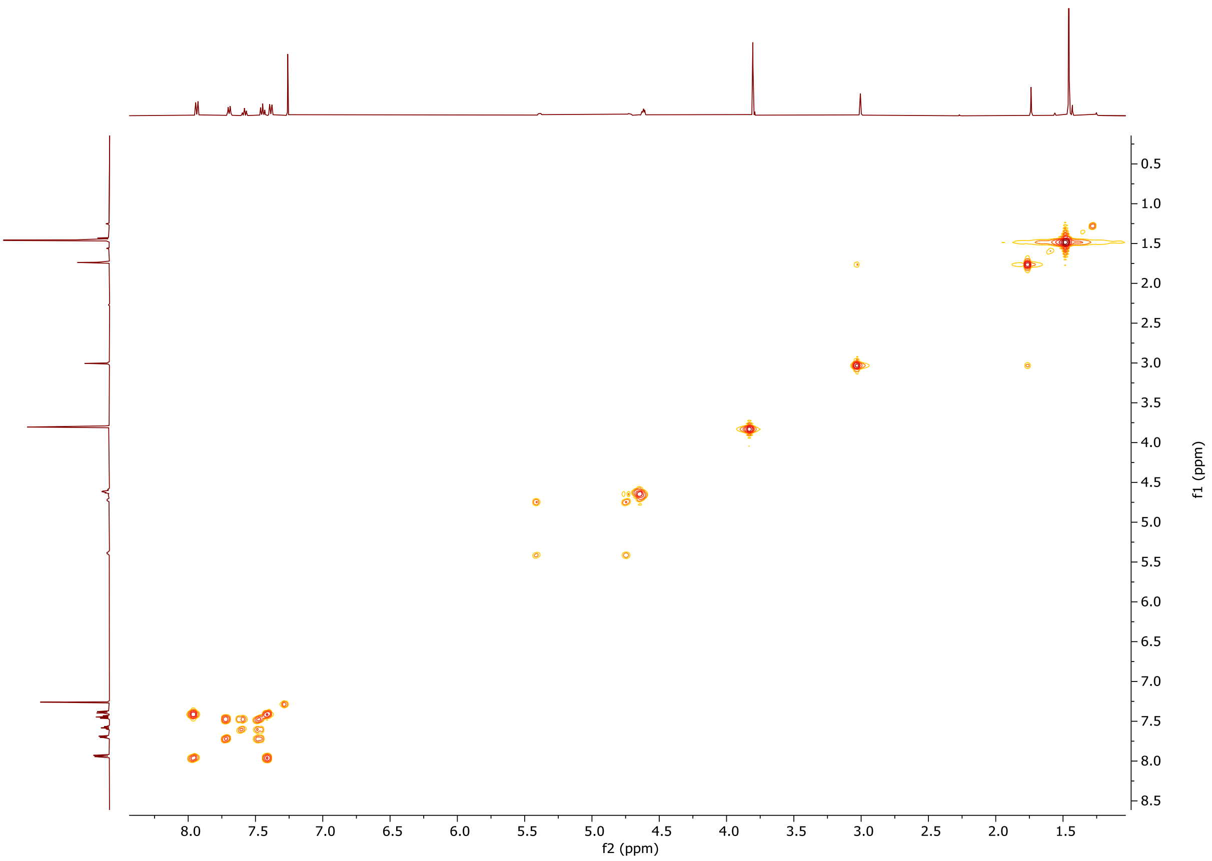

^{1}H/^{13}C HSQC (500/126 MHz, Chloroform-*d*)

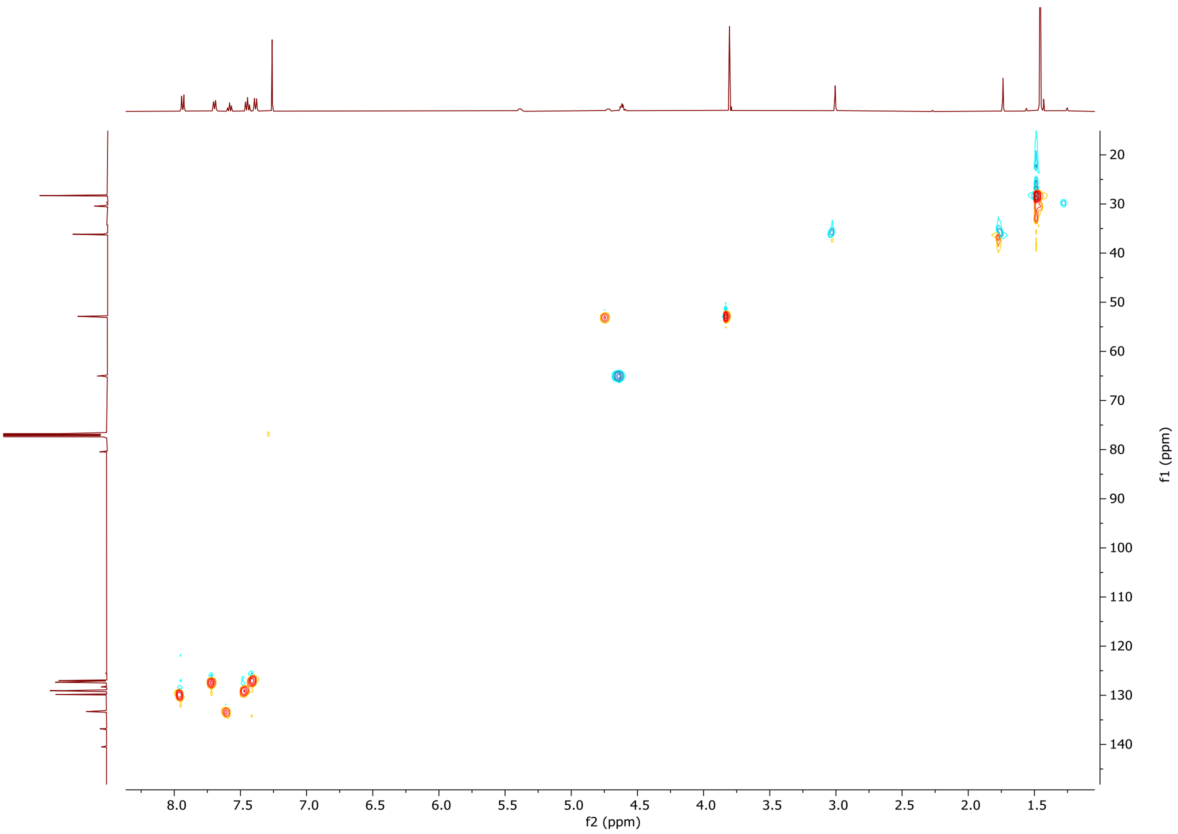

$^{1}H/^{13}C$ HMBC (500/126 MHz, Chloroform-*d*)

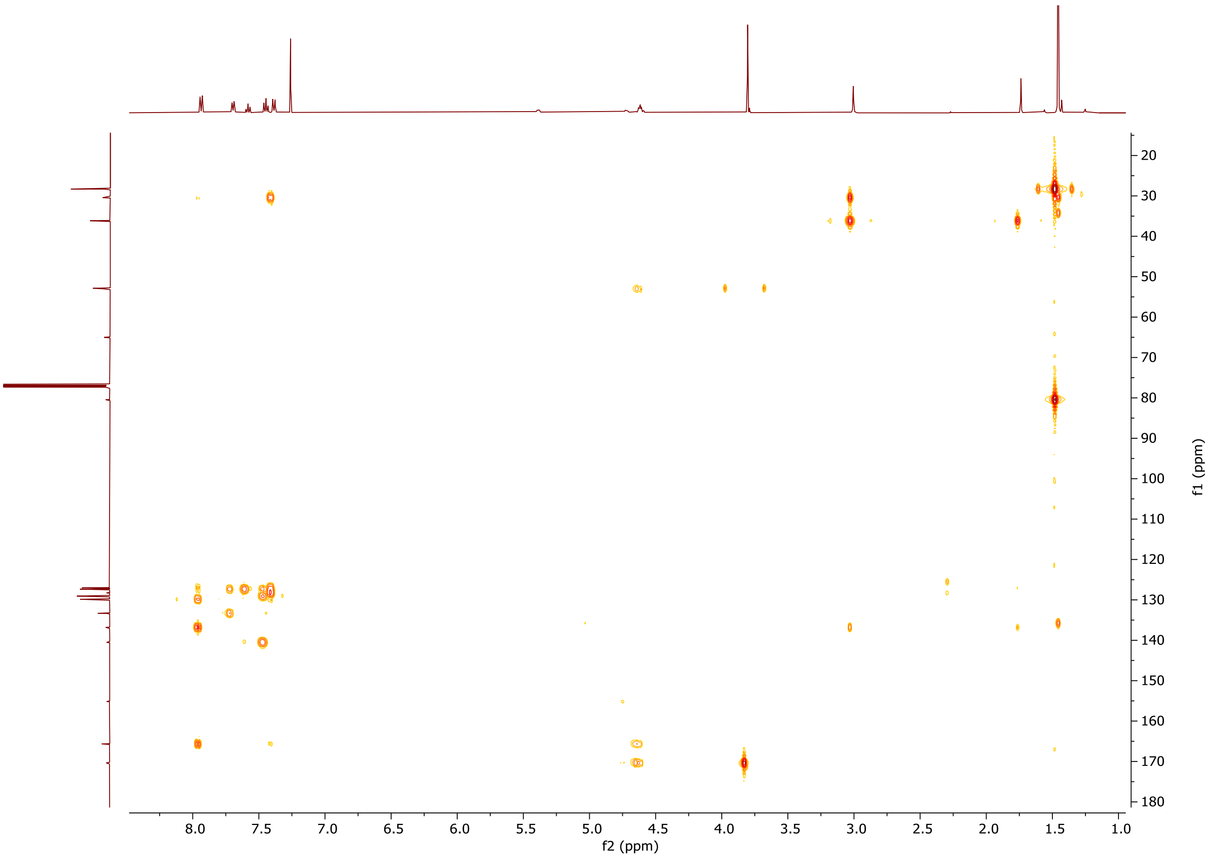

((3aR,5R,5aS,8aS,8bR)-2,2,7,7-tetramethyltetrahydro-5H-bis([1,3]dioxolo)[4,5-b:4',5'-*d*]pyran-5-yl)methyl 4-(3-(phenylsulfonyl)bicyclo[1.1.0]butan-1-yl)benzoate, 44

^{1}H NMR (500 MHz, Chloroform-*d*)

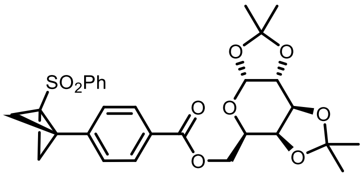

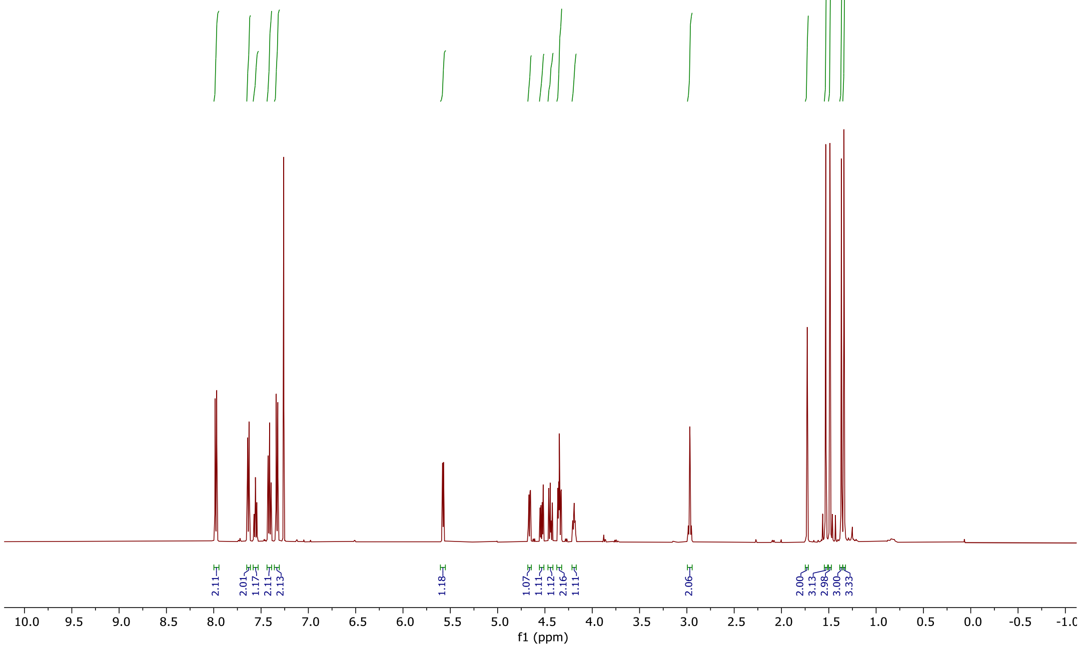

^{13}C NMR (126 MHz, Chloroform-*d*)

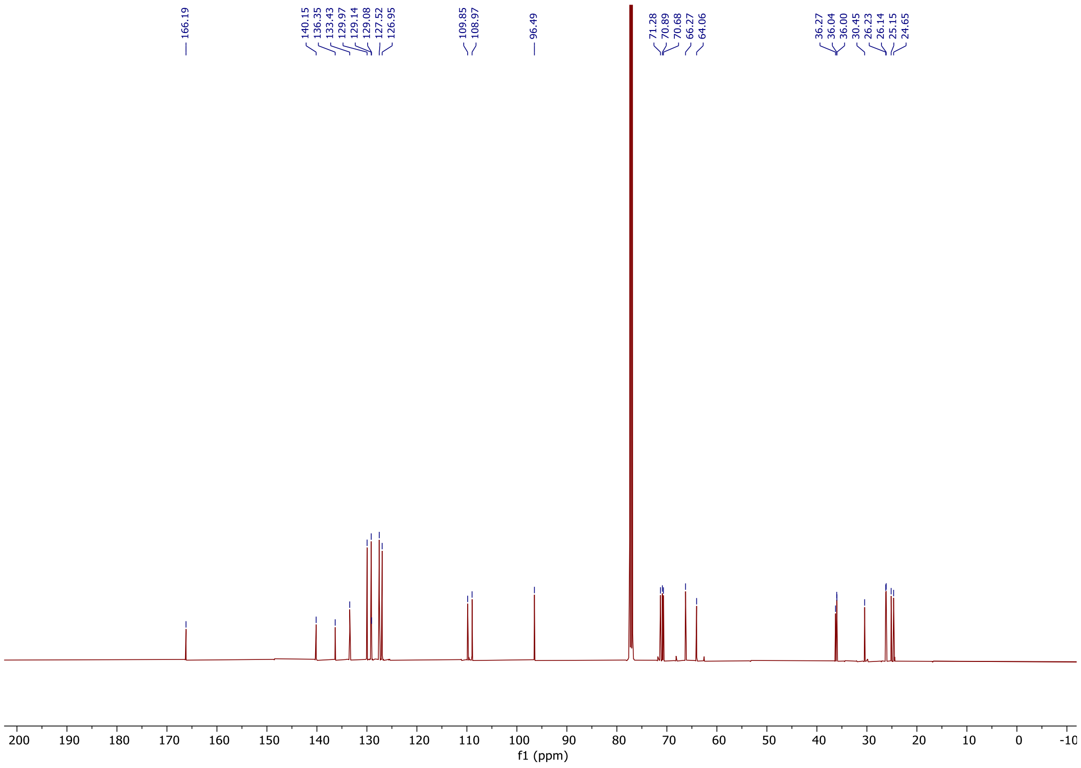

^{1}H COSY (500 MHz, Chloroform-*d*)

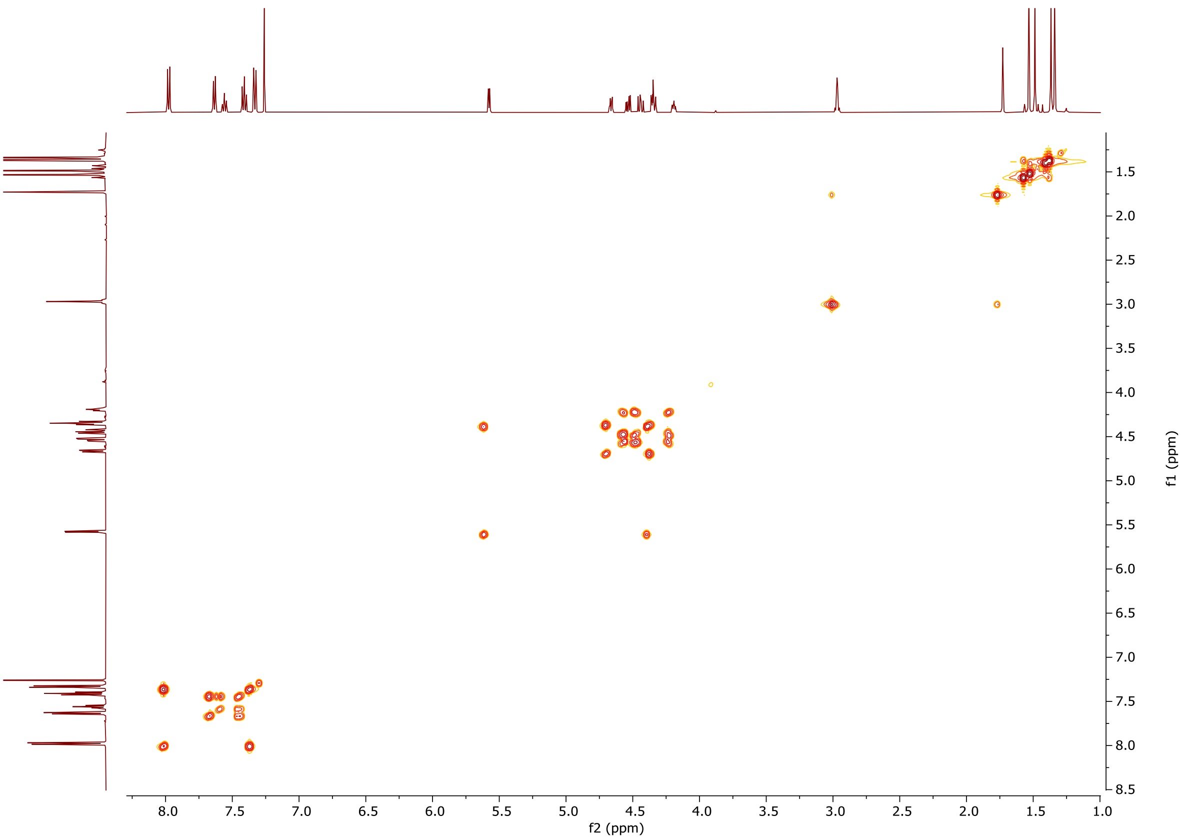

^{1}H/^{13}C HSQC (500/126 MHz, Chloroform-*d*)

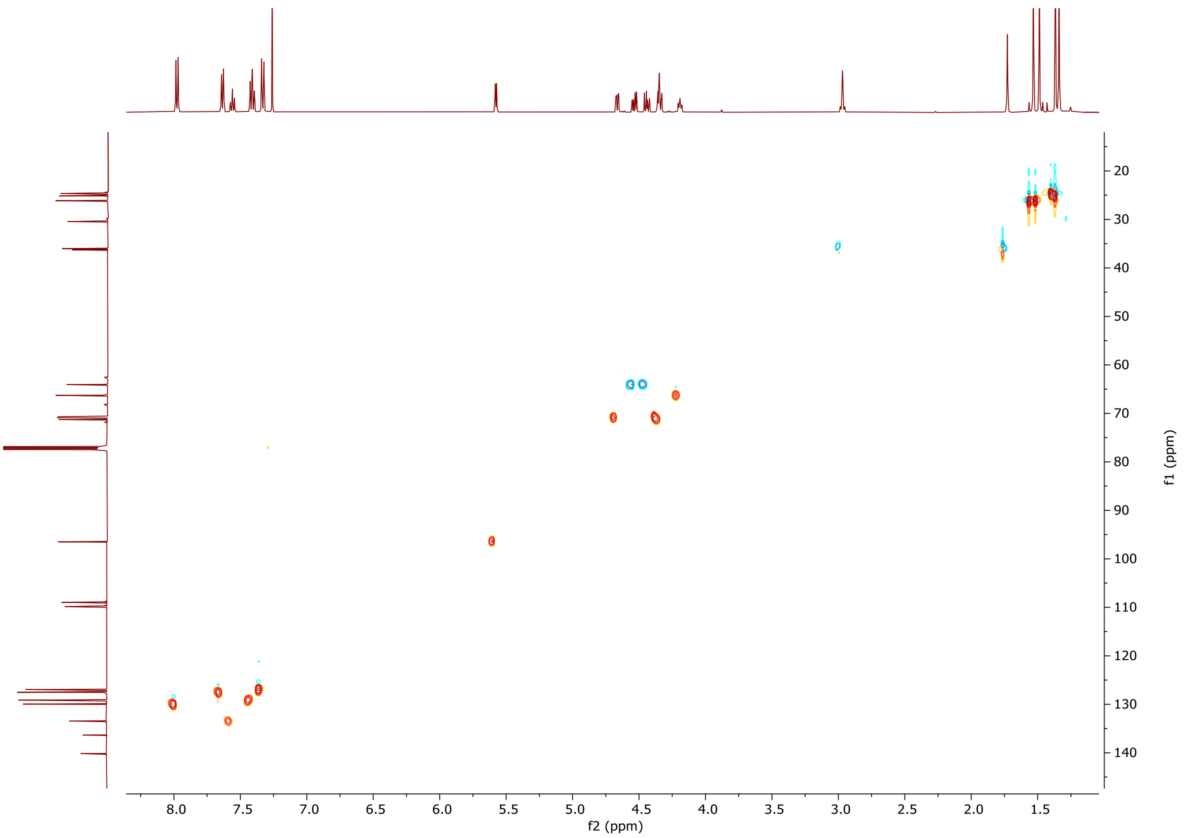

$^{1}H/^{13}C$ HMBC (500/126 MHz, Chloroform-*d*)

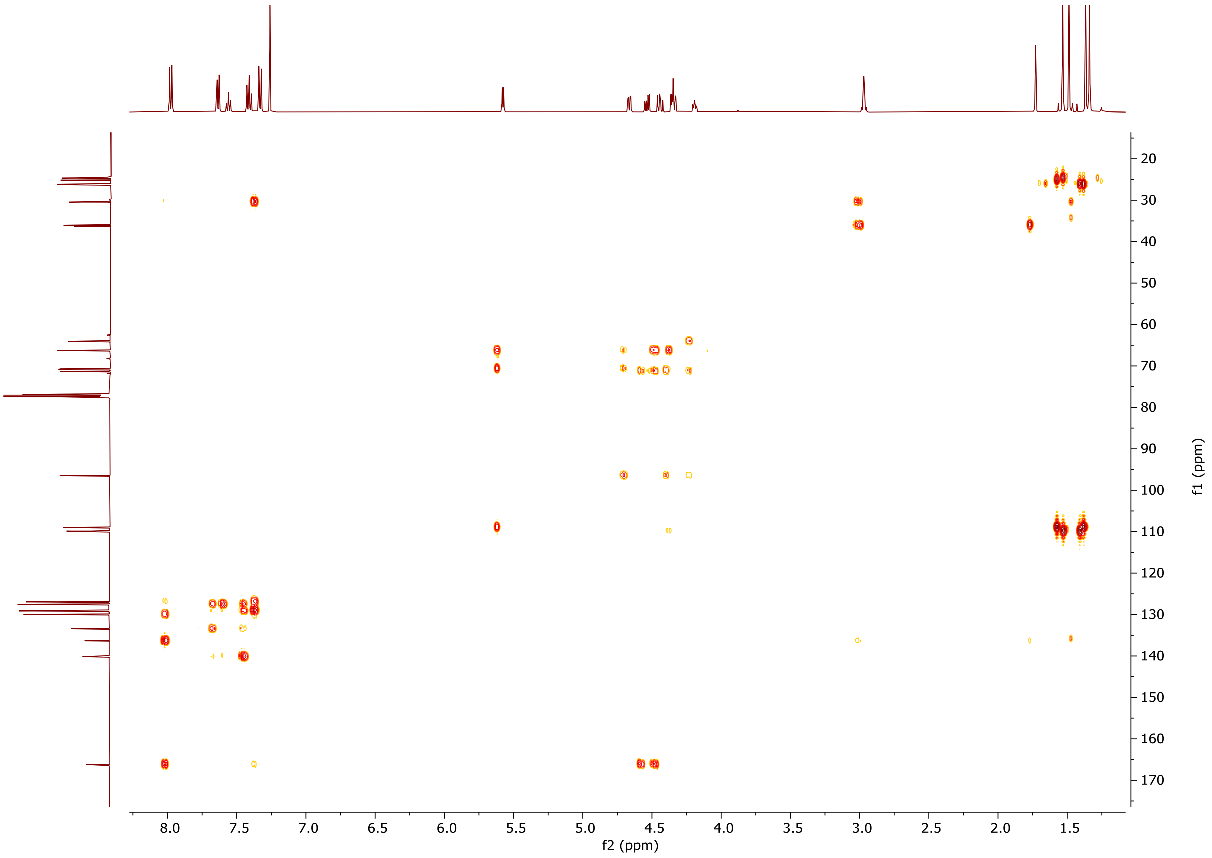

(8R,9S,13S,14S)-13-methyl-3-(3-(phenylsulfonyl)bicyclo[1.1.0]butan-1-yl)-6,7,8,9,11,12,13,14,15,16-decahydro-17H-cyclopenta[a]phenanthren-17-one, 45

^{1}H NMR (400 MHz, Chloroform-*d*)

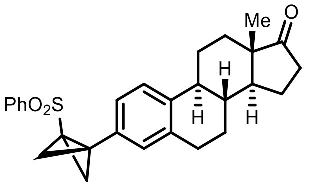

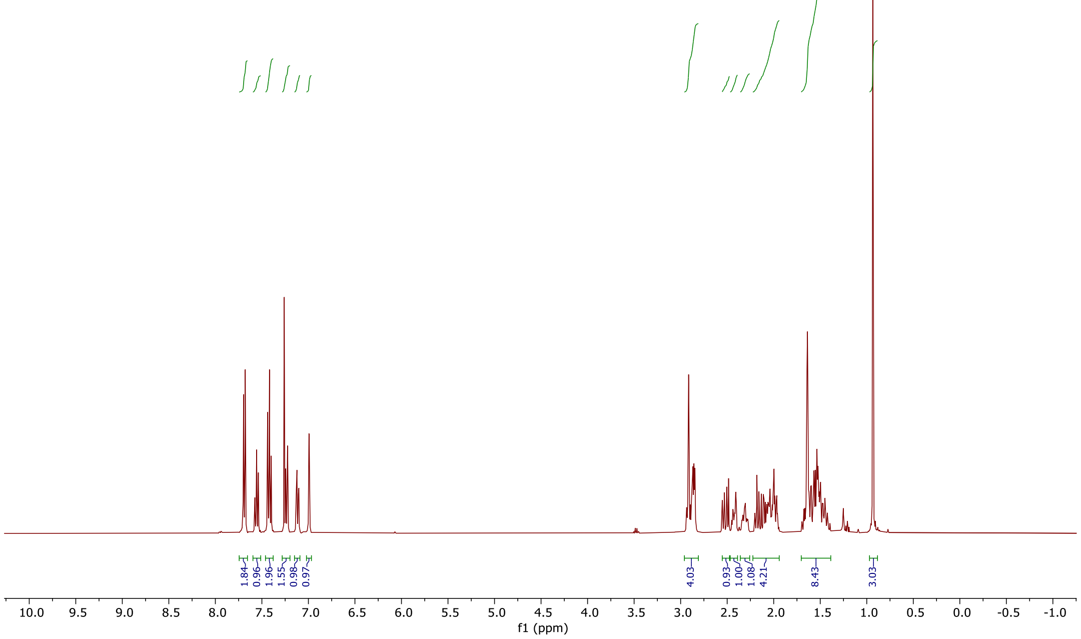

^{13}C NMR (126 MHz, Chloroform-*d*)

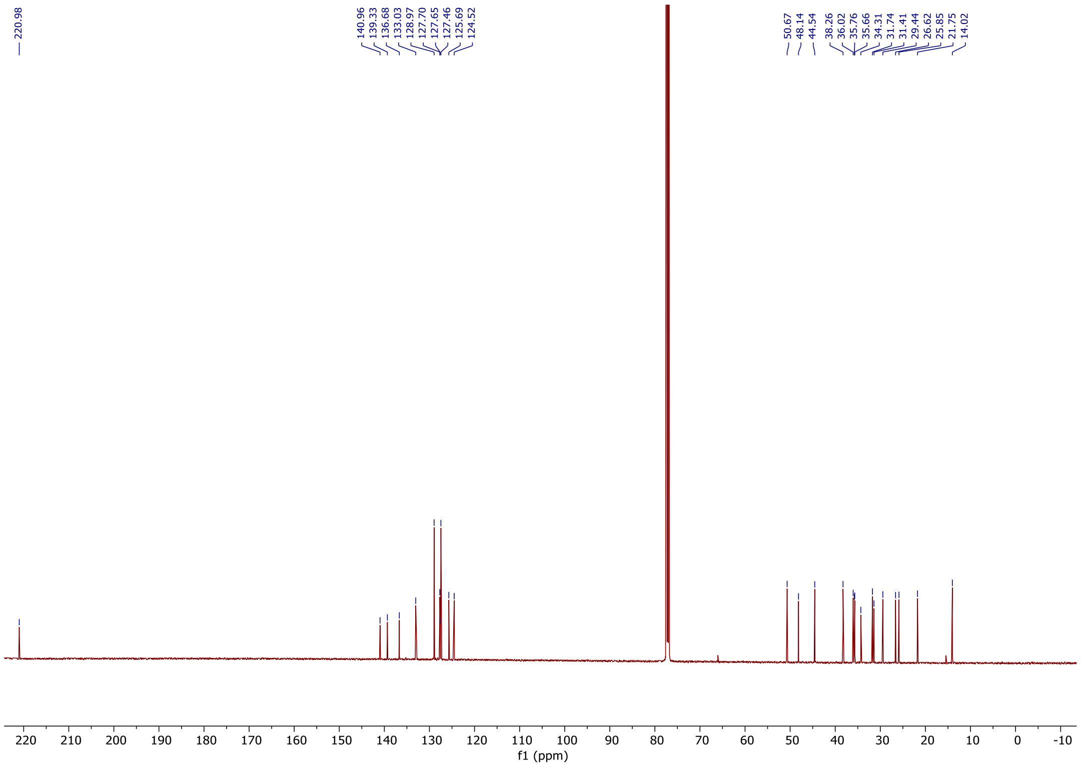

^{1}H COSY (500 MHz, Chloroform-*d*)

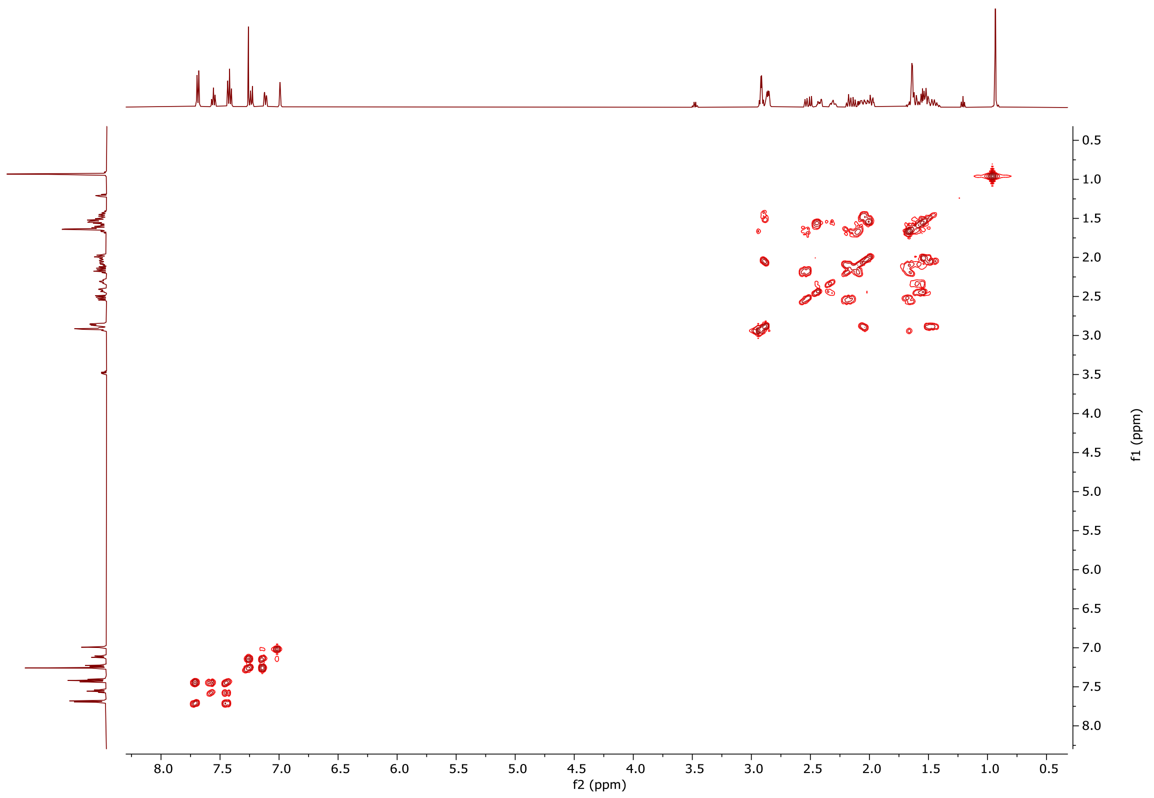

^{1}H/^{13}C HSQC (500/126 MHz, Chloroform-*d*)

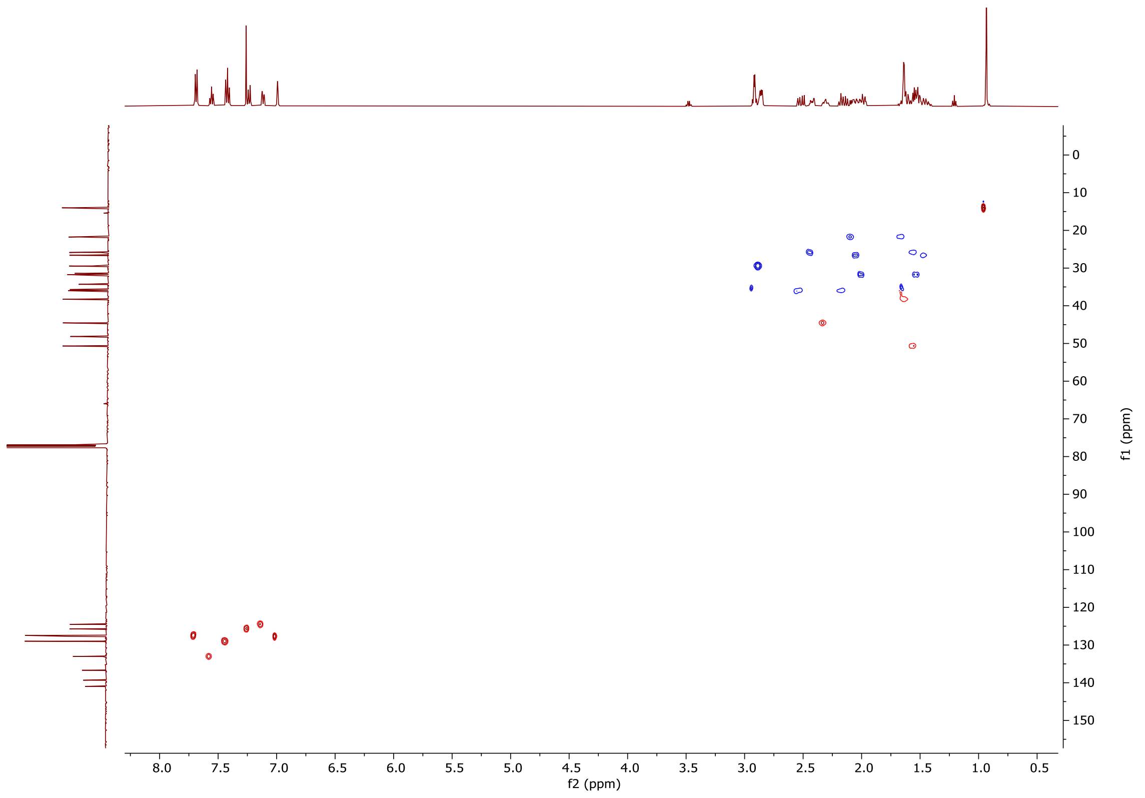

^{1}H/^{13}C HMBC (500/126 MHz, Chloroform-*d*)

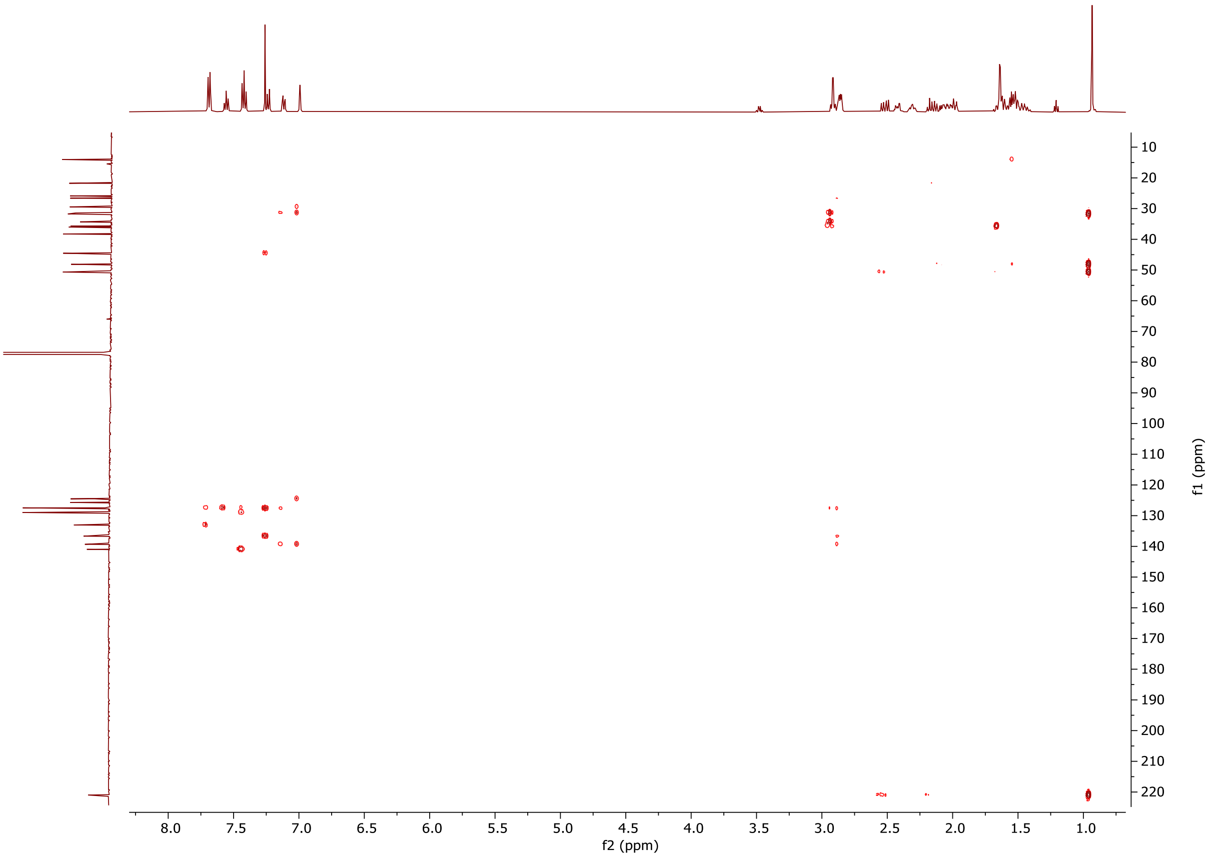

1-methoxy-4-(3-(phenylsulfonyl)cyclobut-1-en-1-yl)benzene, 46

^{1}H NMR (400 MHz, Chloroform-*d*)

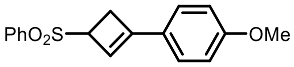

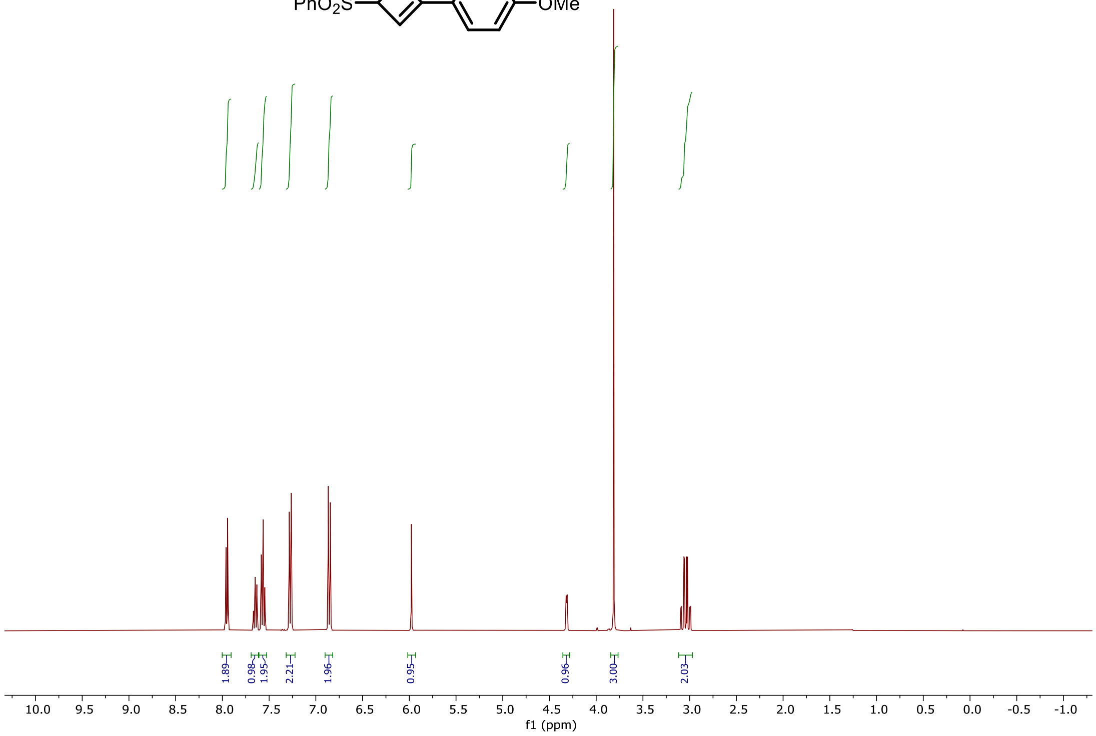

^{13}C NMR (101 MHz, Chloroform-*d*)

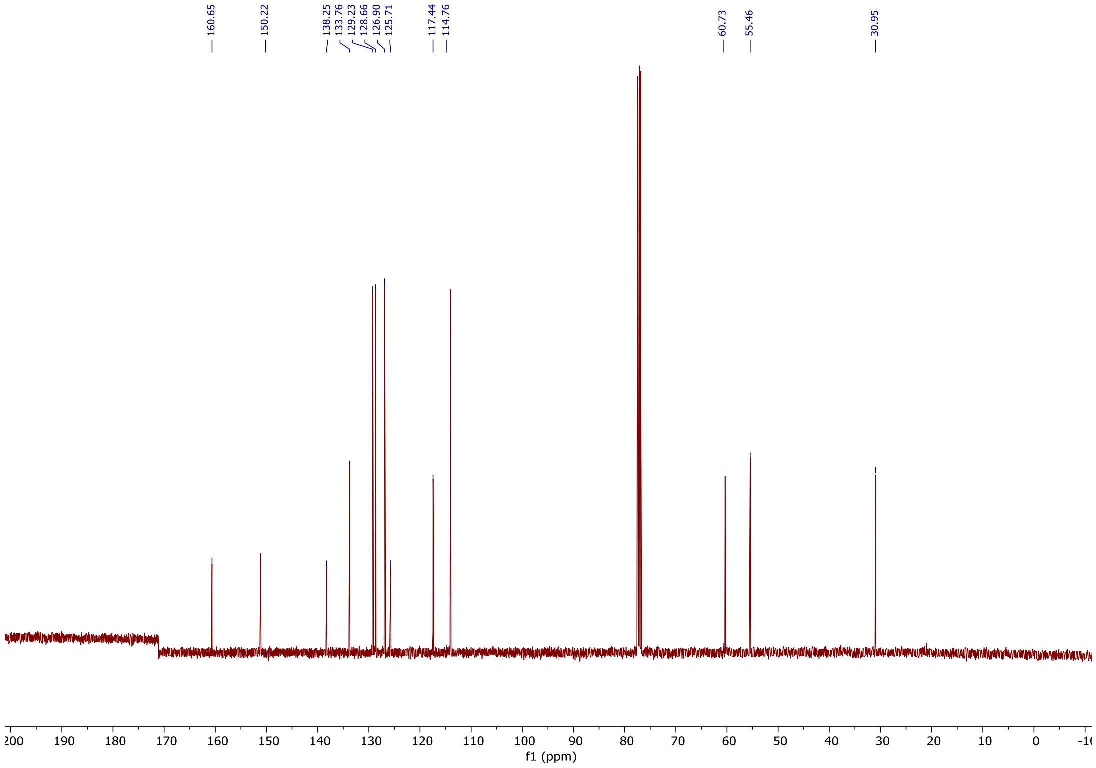

^{1}H COSY (400 MHz, Chloroform-*d*)

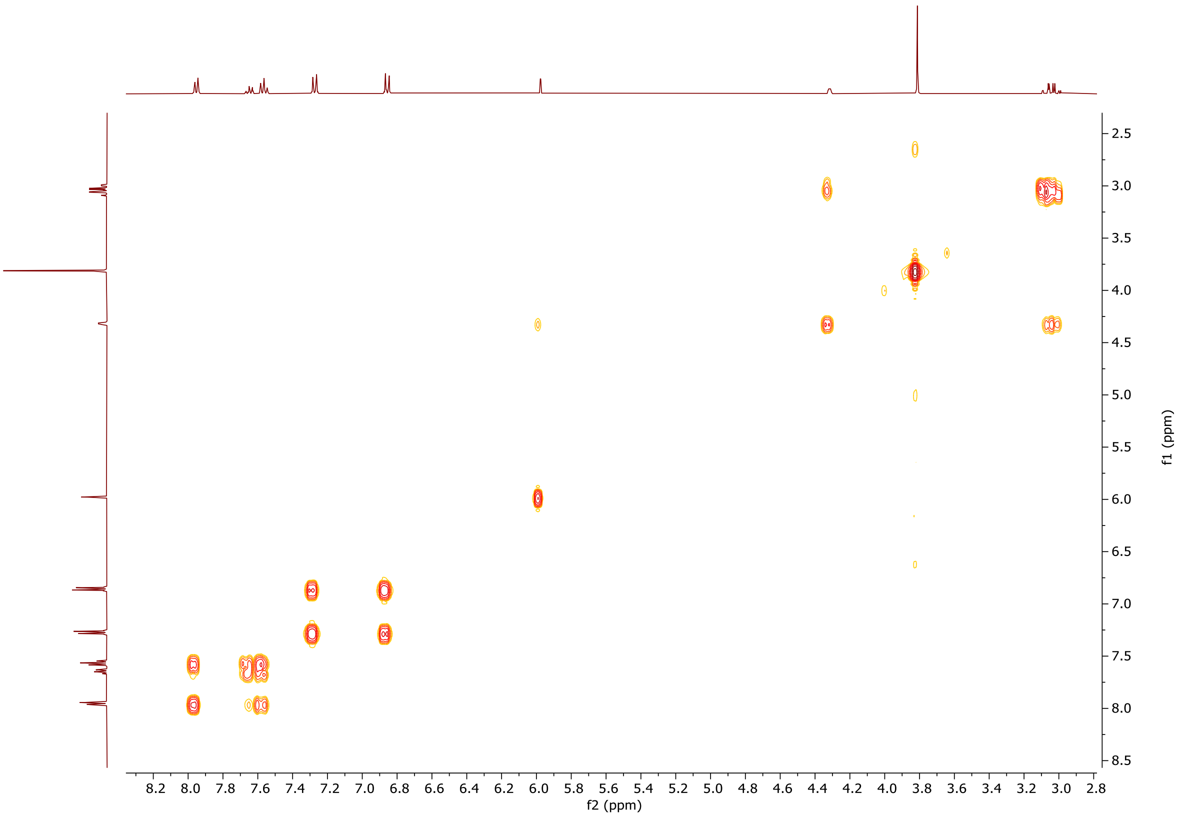

^{1}H/^{13}C HSQC (400/101 MHz, Chloroform-*d*)

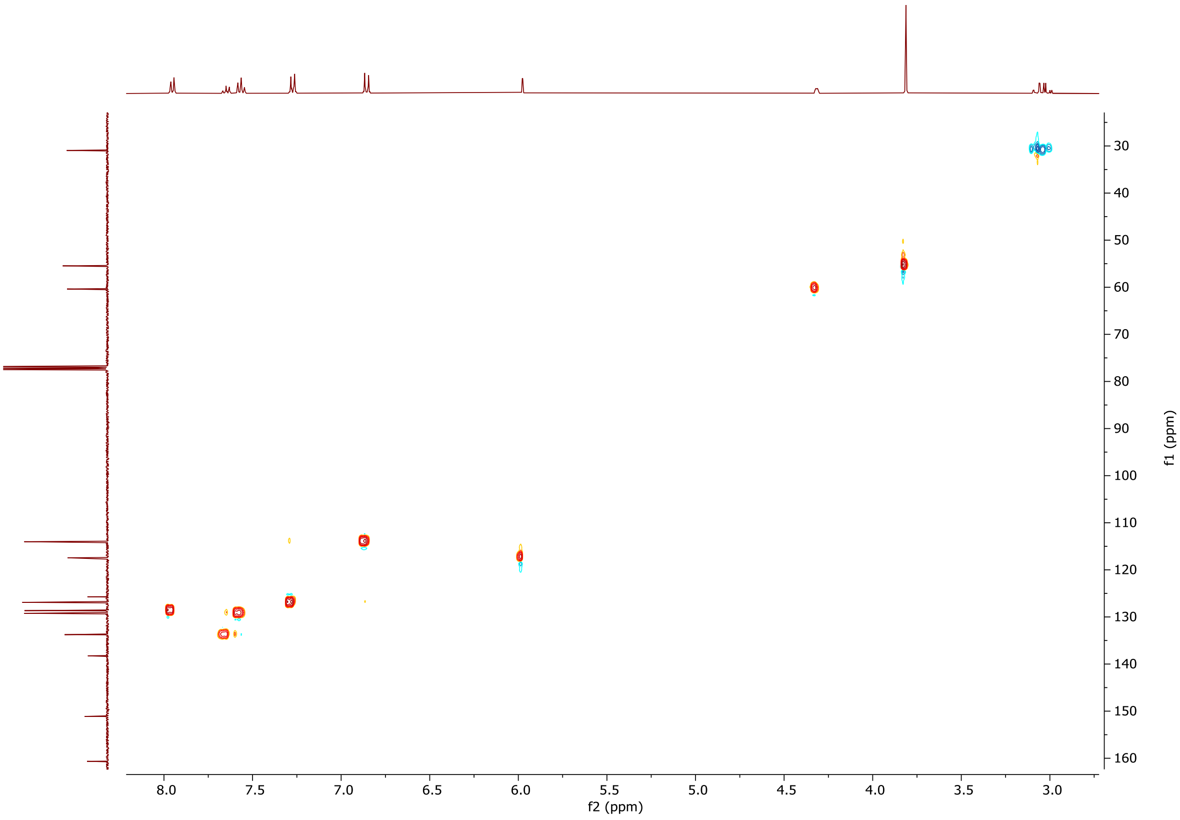

$^1H/^{13}C$ HMBC (500/126 MHz, Chloroform-*d*)

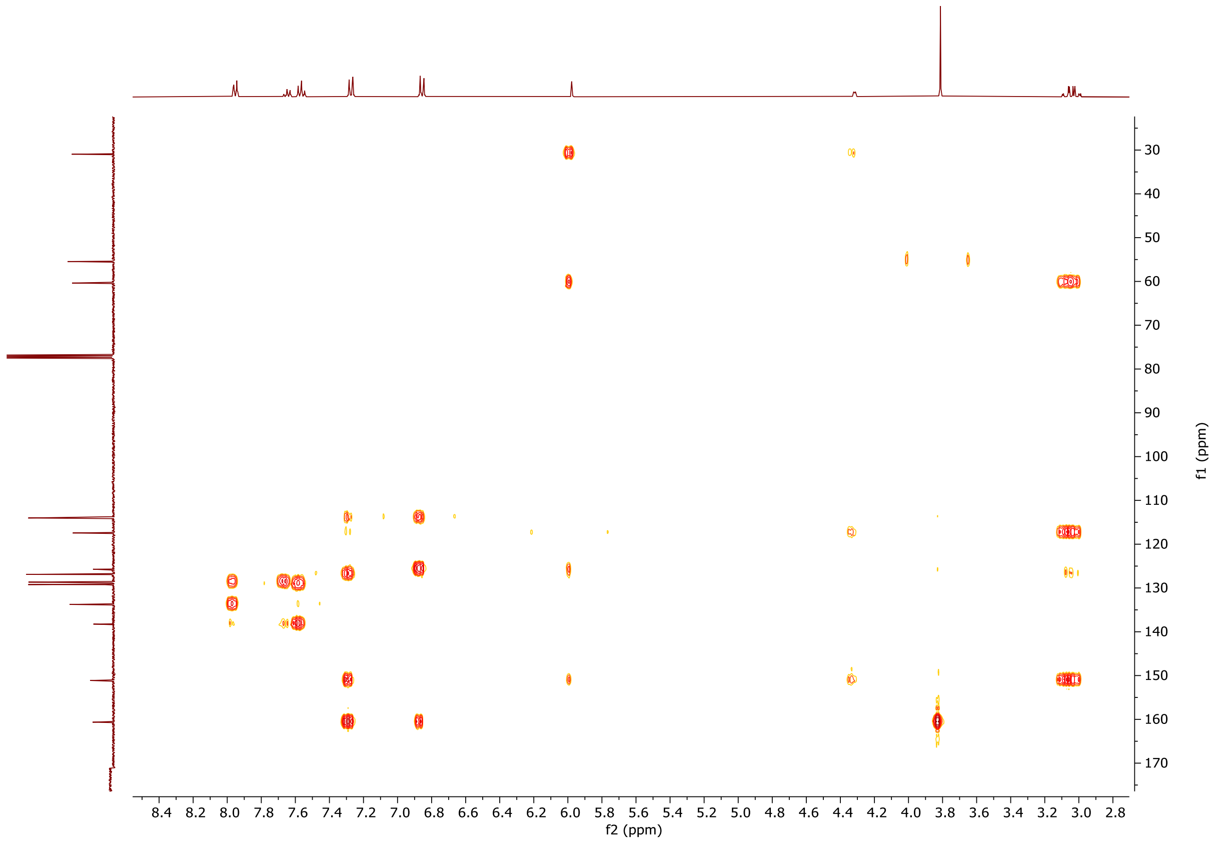

***Tert*-butyldimethyl(3-(3-(phenylsulfonyl)cyclobut-1-en-1-yl)phenoxy)silane, 47**

^{1}H NMR (500 MHz, Chloroform-*d*)

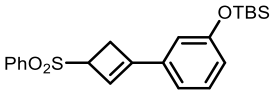

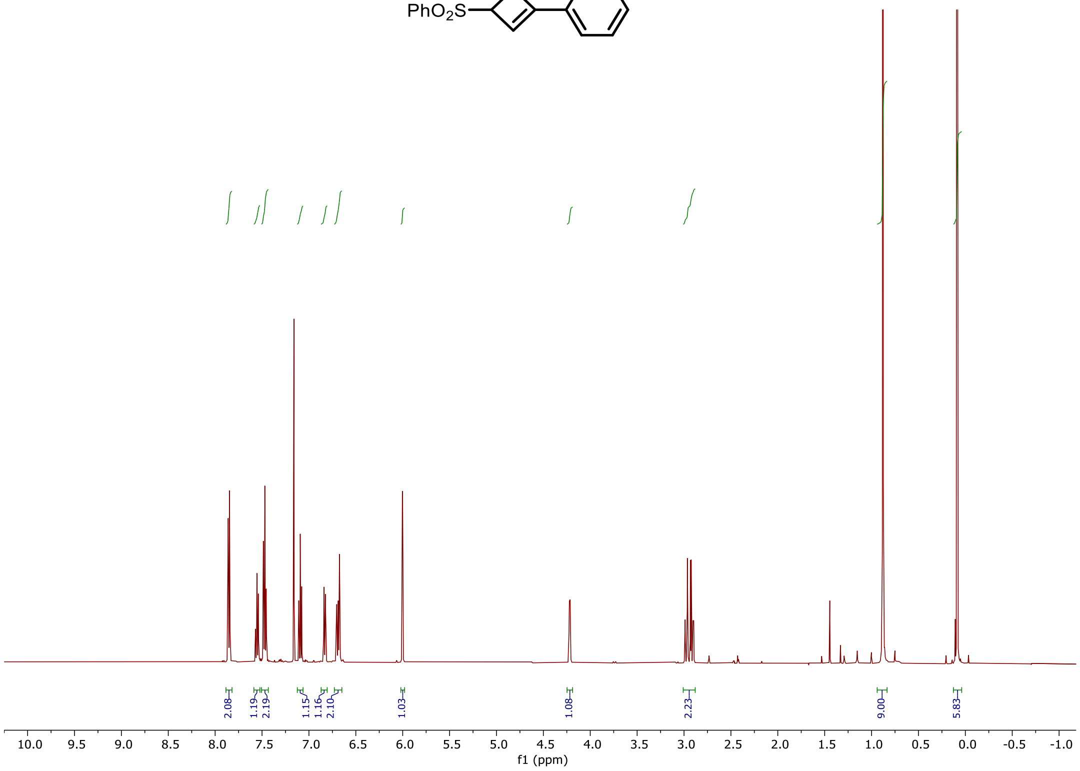

^{13}C NMR (126 MHz, Chloroform-*d*)

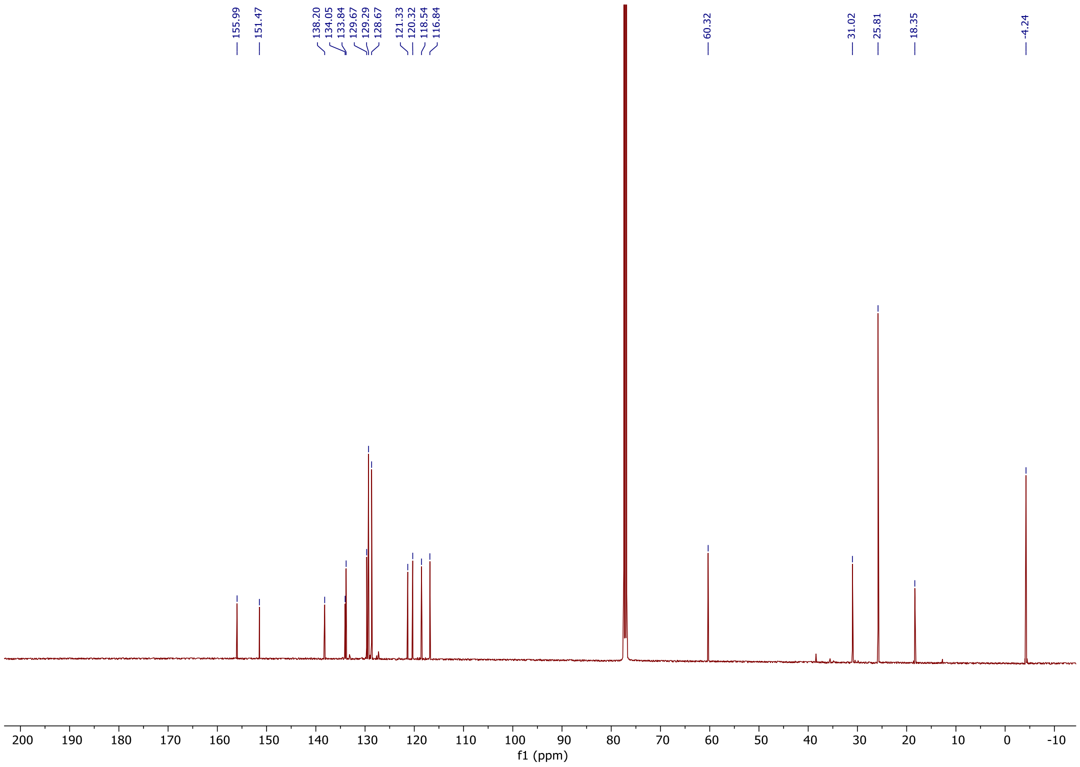

¹H COSY (500 MHz, Chloroform-*d*)

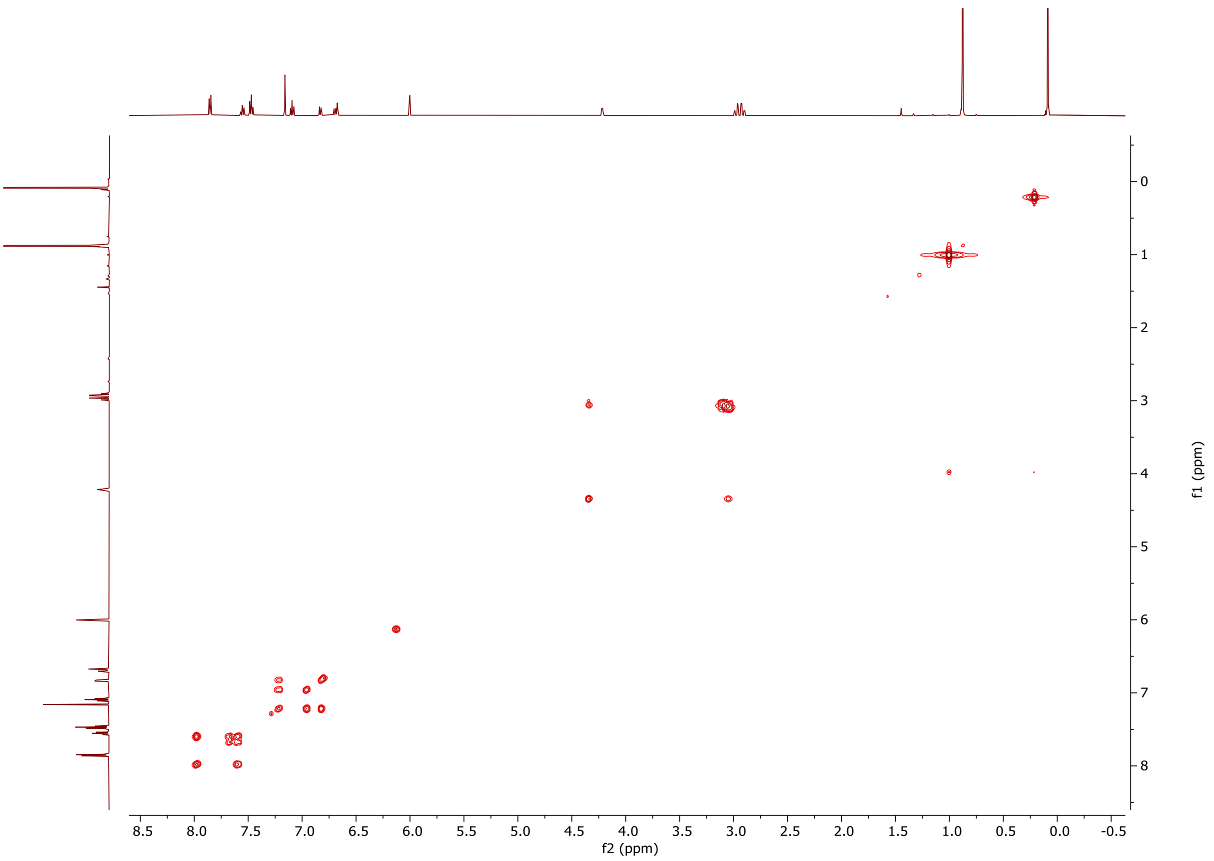

¹H/¹³C HSQC (500/126 MHz, Chloroform-*d*)

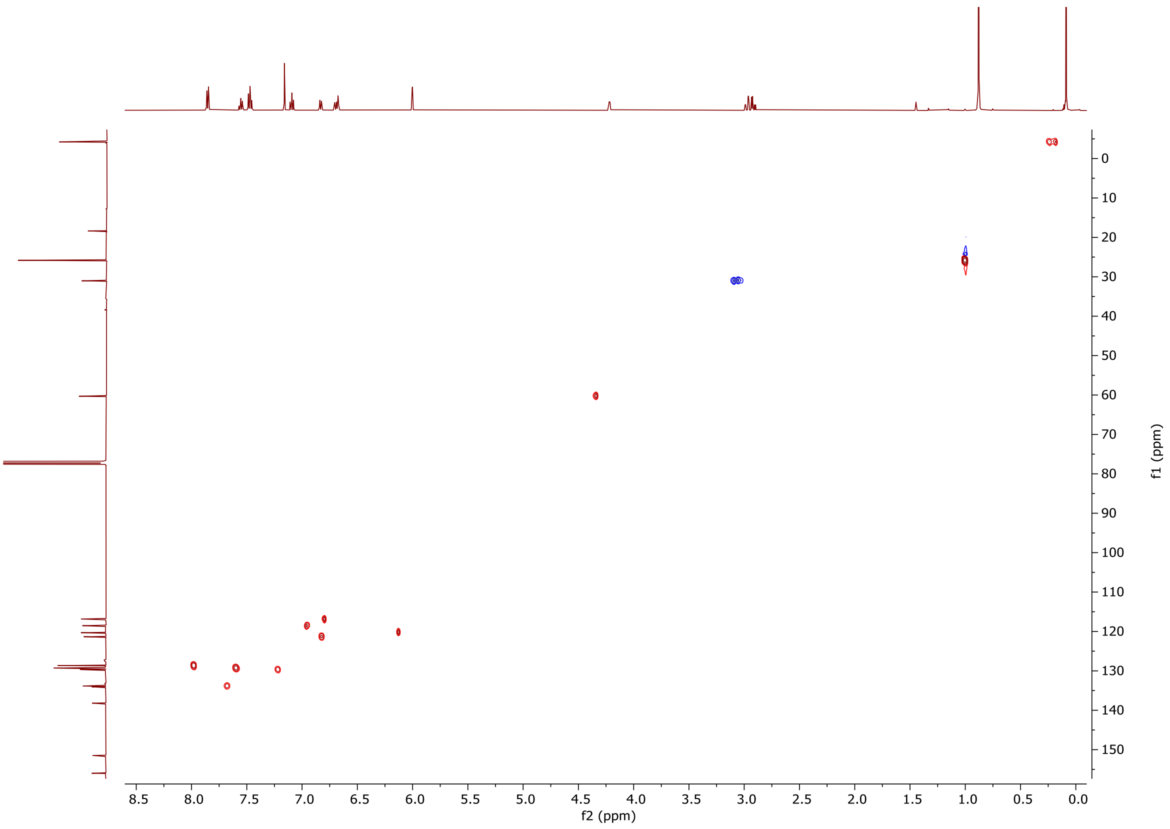

$^{1}H/^{13}C$ HMBC (500/126 MHz, Chloroform-*d*)

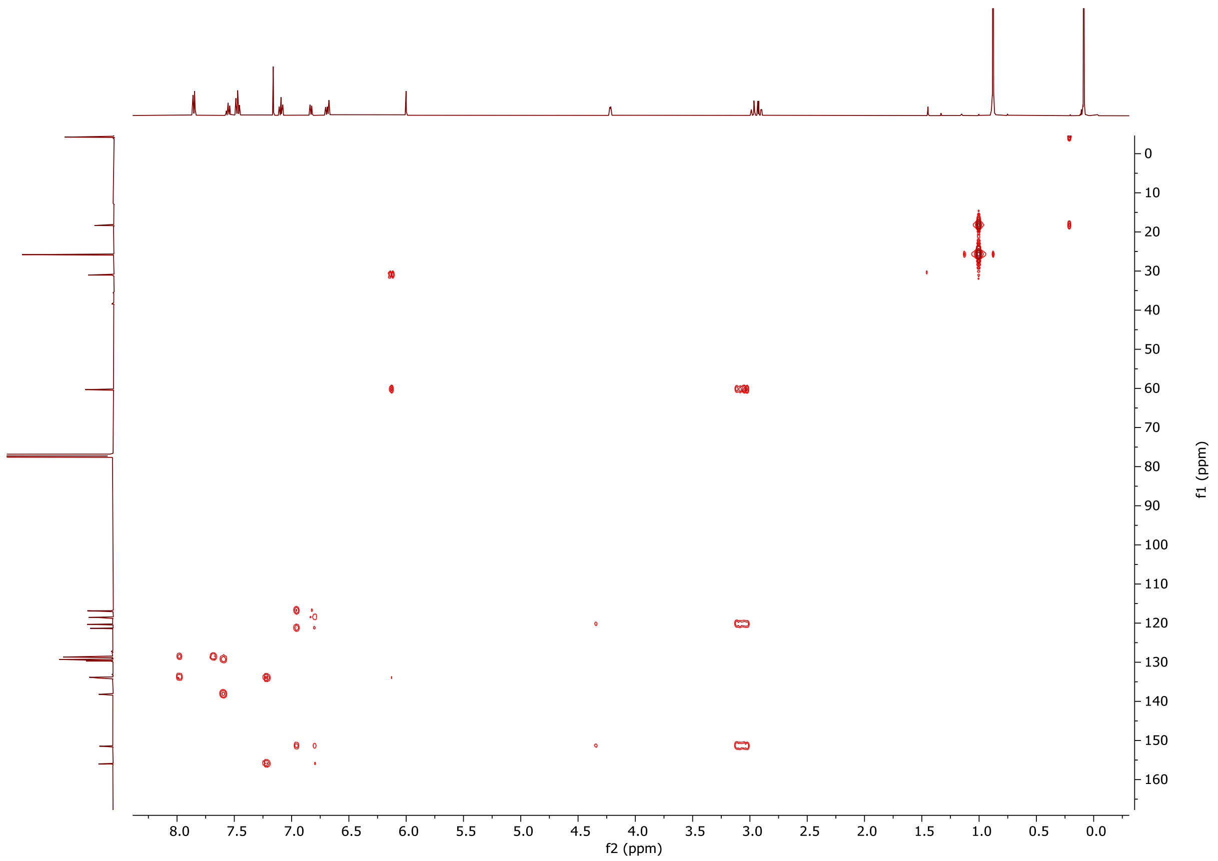

Ethyl (1*S,3*S**,5*R**)-1-phenyl-3-(phenylsulfonyl)bicyclo[2.1.0]pentane-5-carboxylate, 48**

^{1}H NMR (400 MHz, Chloroform-*d*)

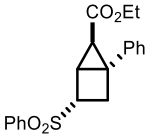

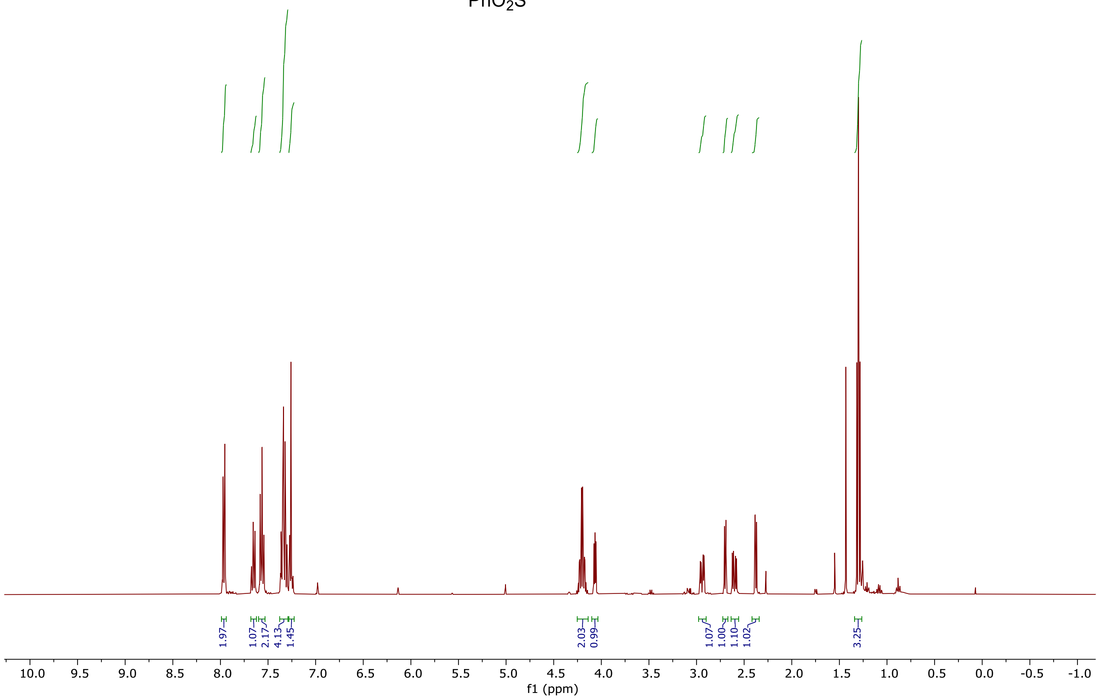

^{13}C NMR (101 MHz, Chloroform-*d*)

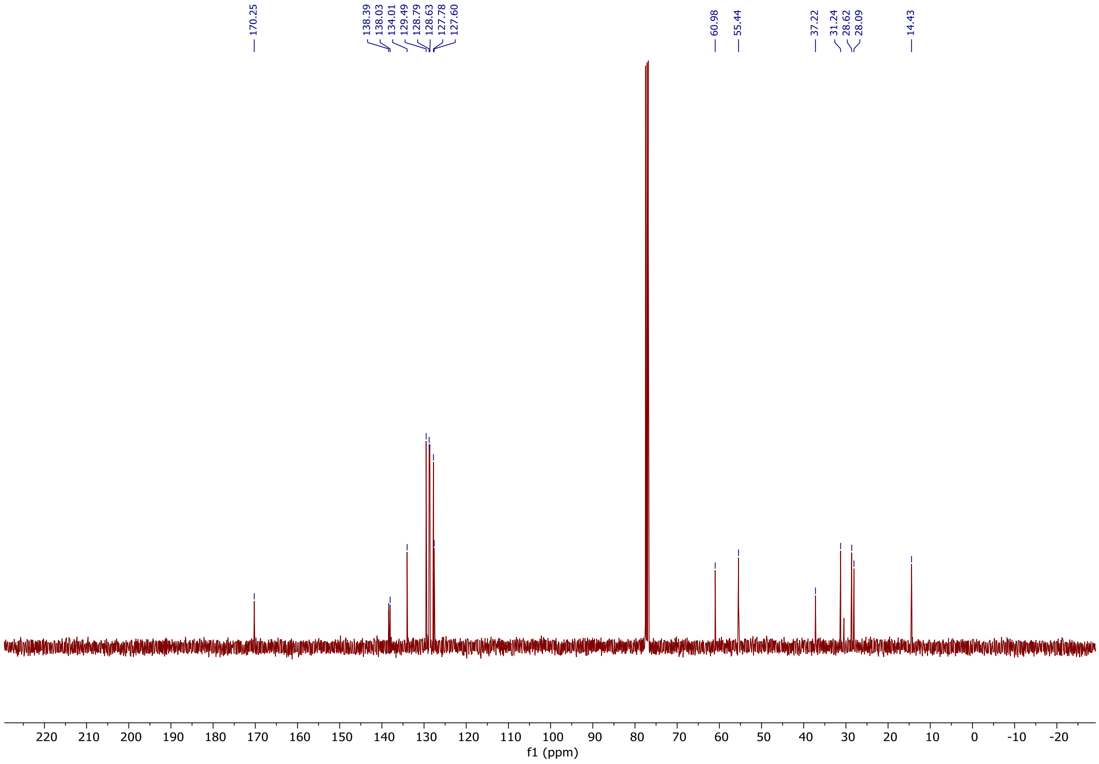

^{1}**H COSY** (400 MHz, Chloroform-*d*)

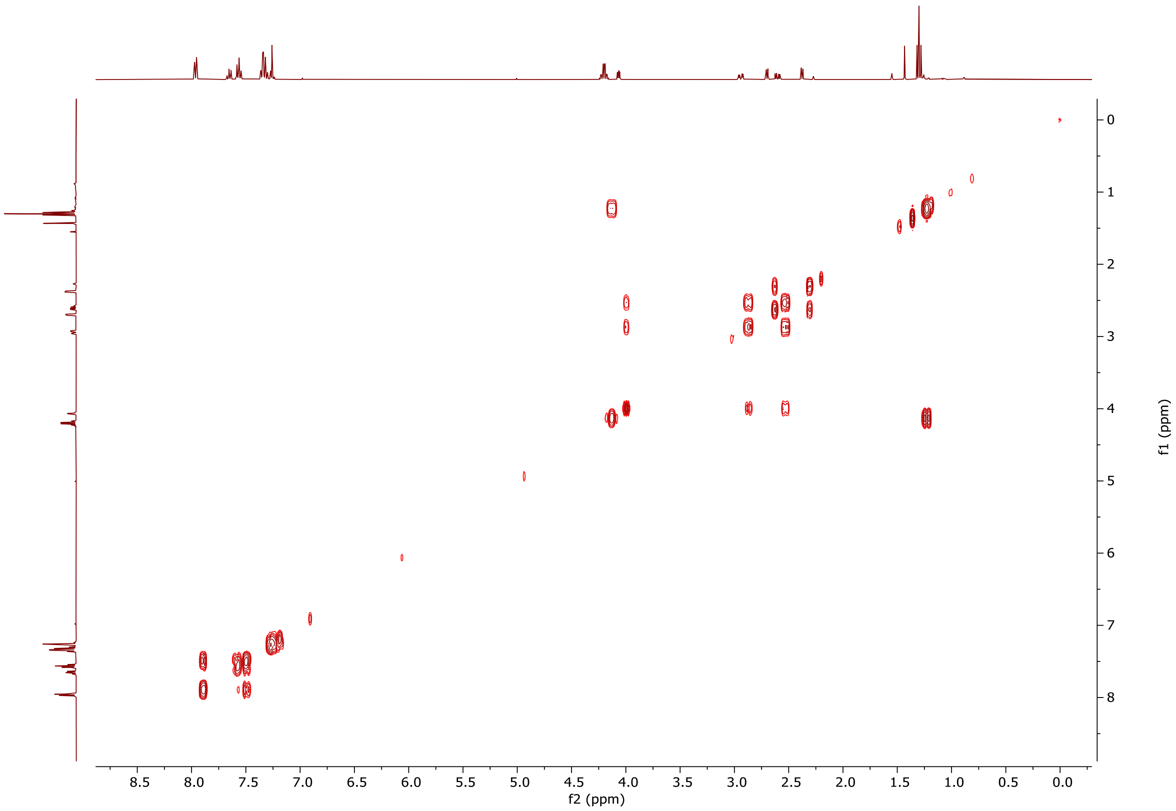

^{1}**H/**^{13}**C HSQC** (400/101 MHz, Chloroform-*d*)

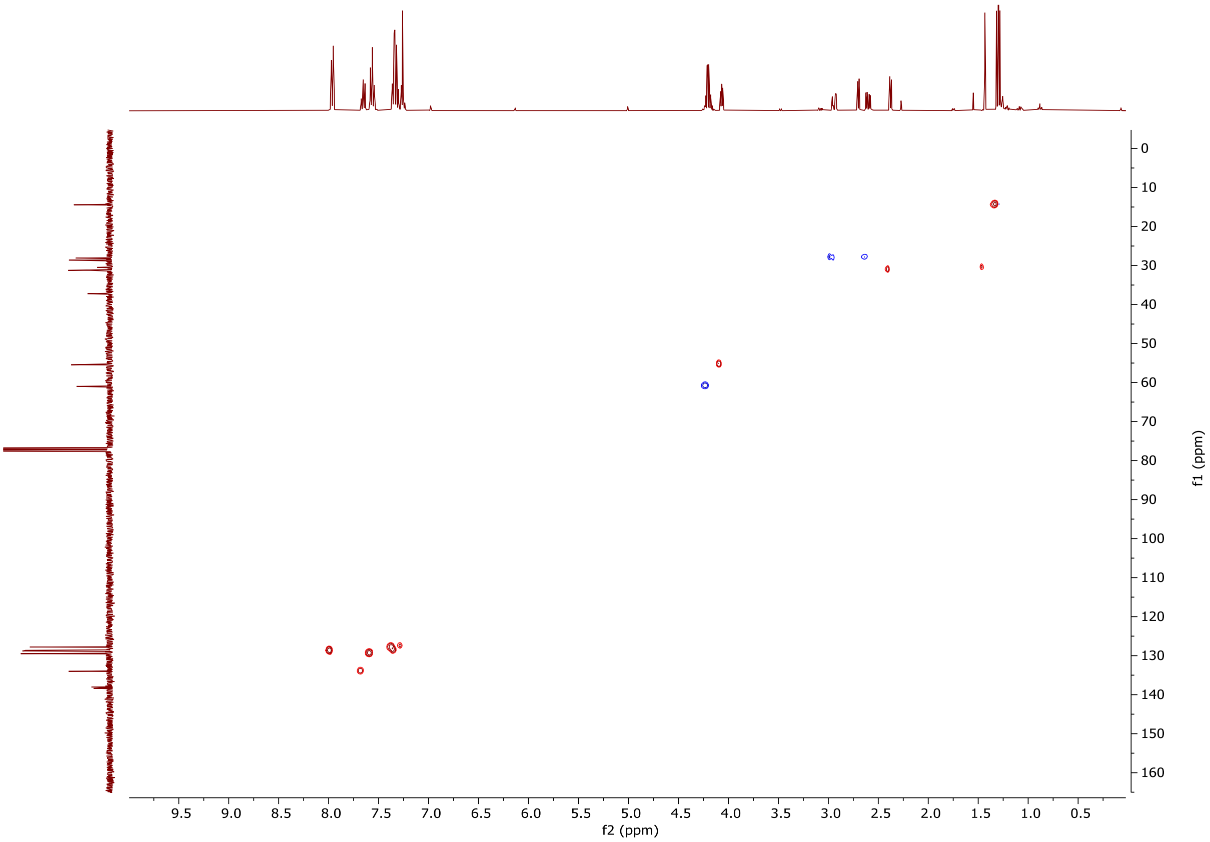

$^1H/^{13}C$ HSQC (500/126 MHz, Chloroform-*d*) **contains Et_2O*

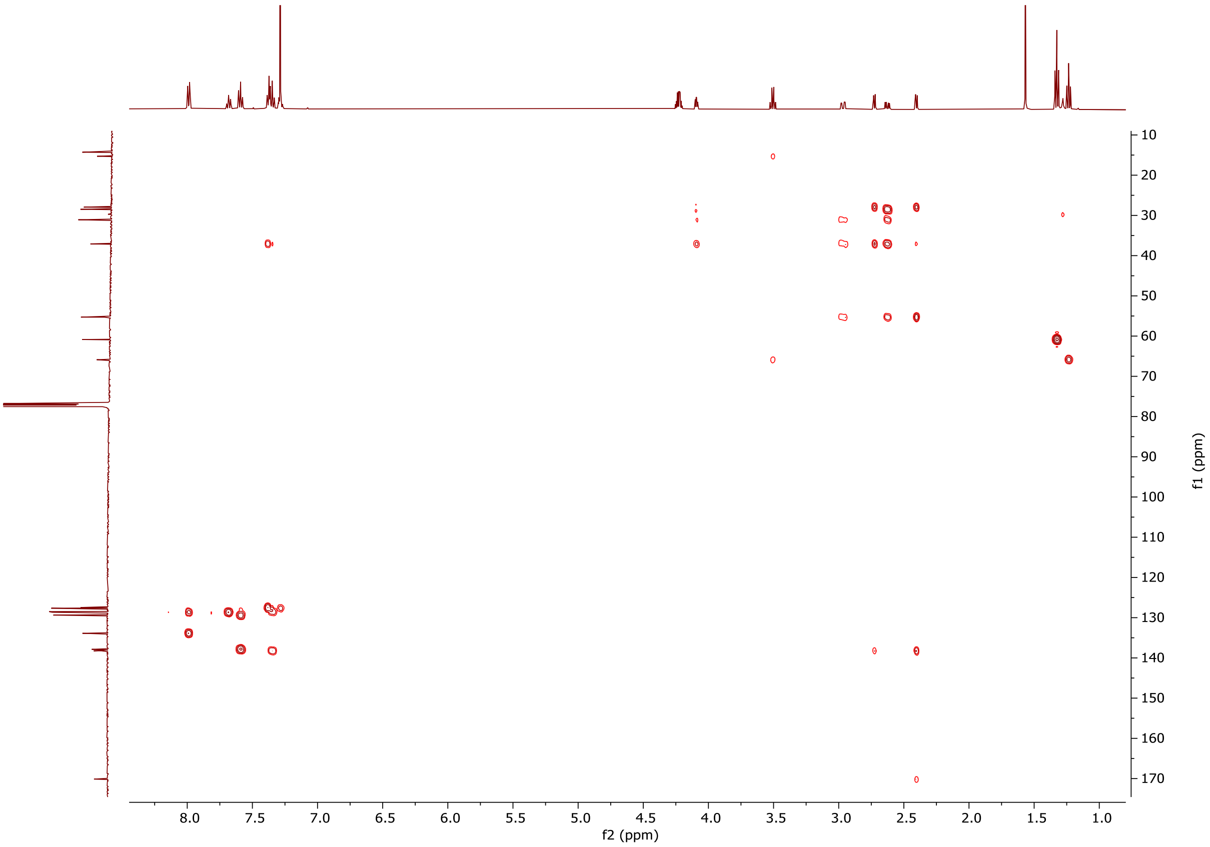

^1H NOESY (500 MHz, Chloroform-*d*) **contains Et_2O*

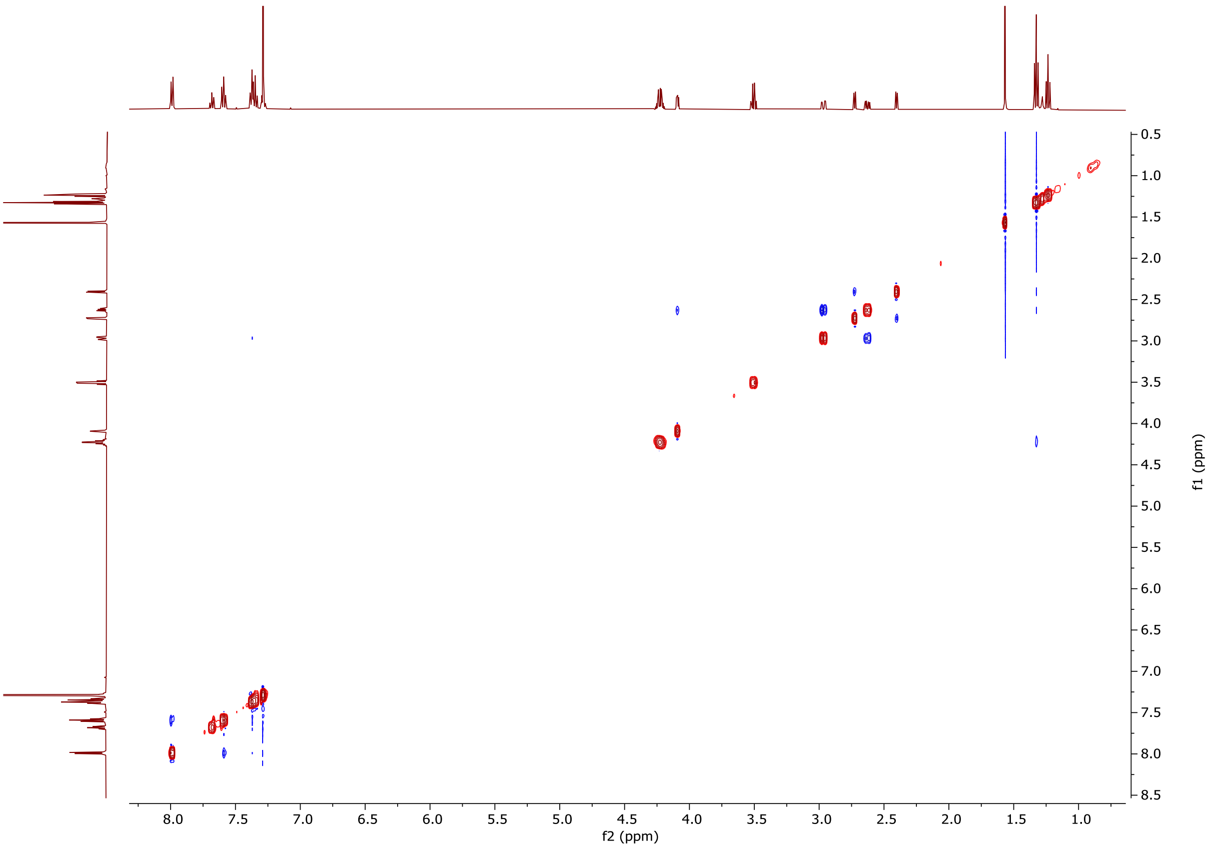

((3-phenylcyclobutyl)sulfonyl)benzene, 49

^{1}H NMR (400 MHz, Chloroform-*d*)

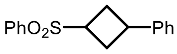

3:1 (syn:anti)

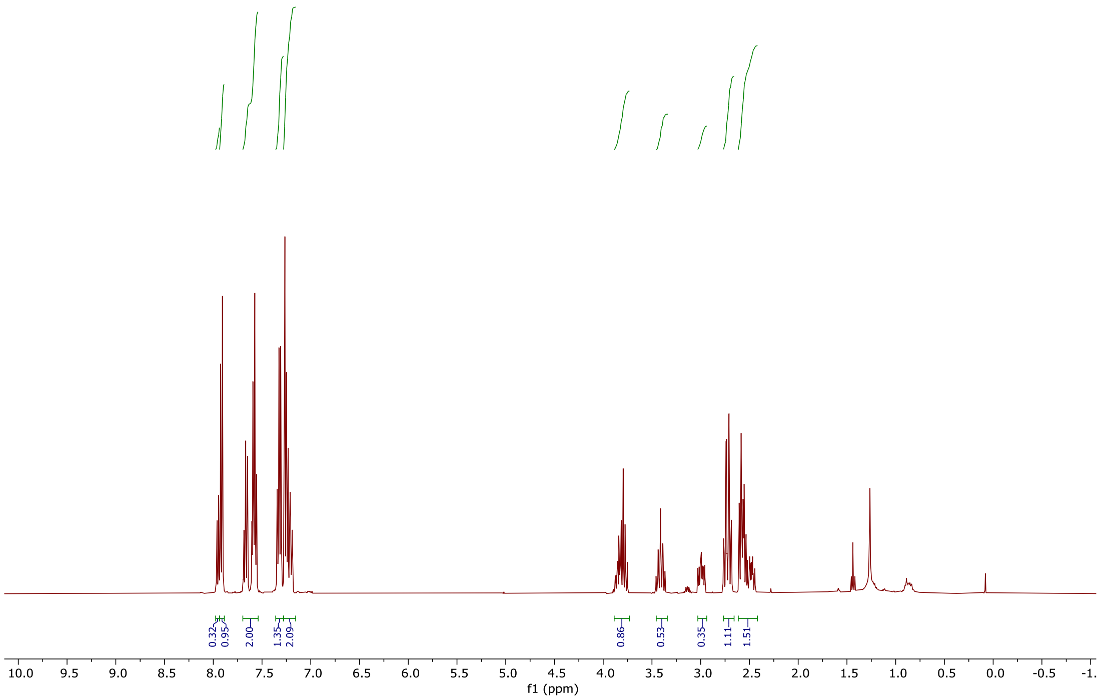

^{13}C NMR (101 MHz, Chloroform-*d*)

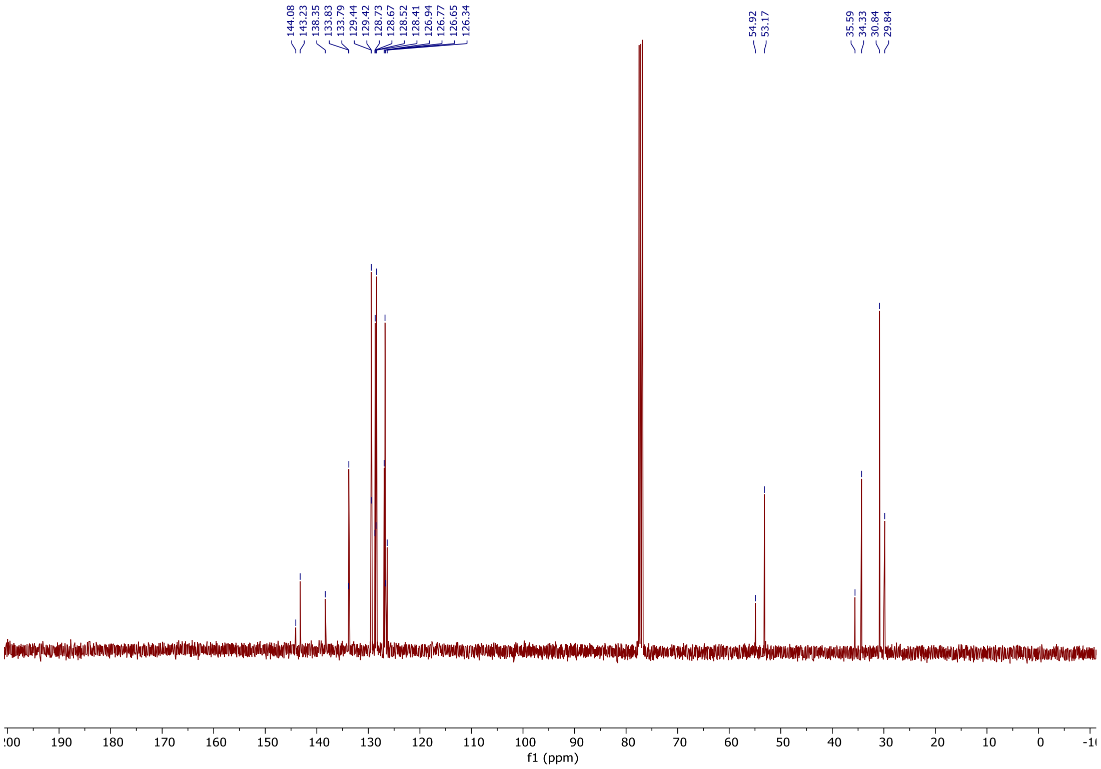

3-chloro-1-methoxy-3-(phenylsulfonyl)cyclobutyl)benzene, 50

^{1}H NMR (500 MHz, Chloroform-*d*)

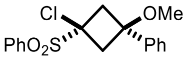

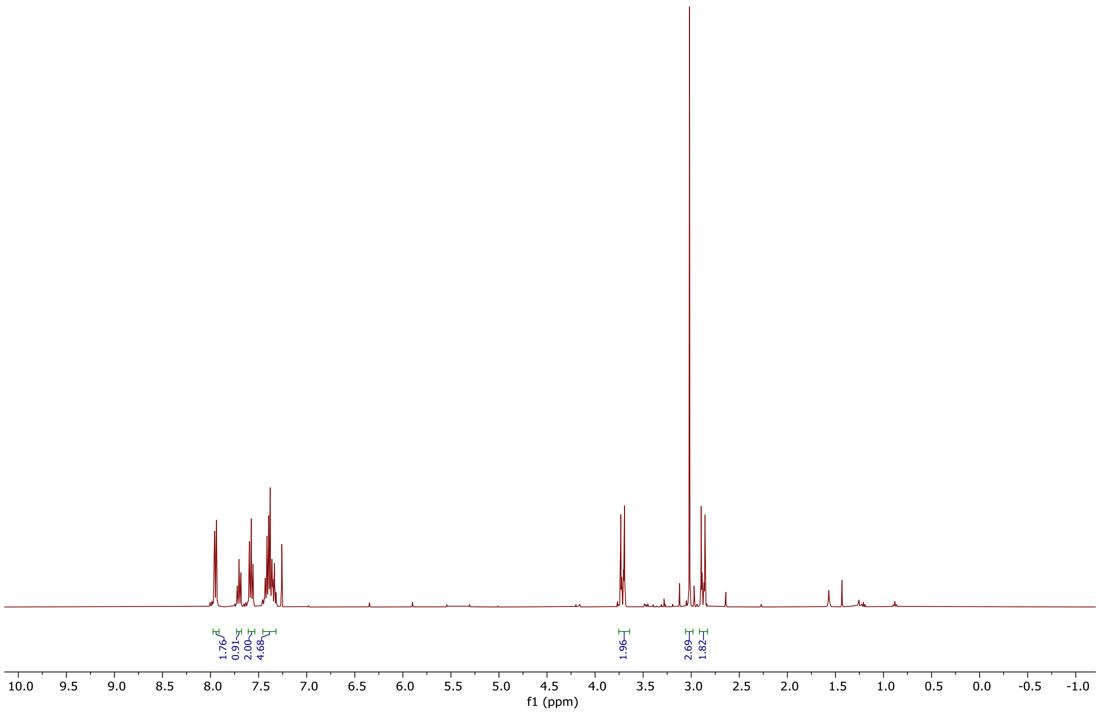

^{13}C NMR (126 MHz, Chloroform-*d*)

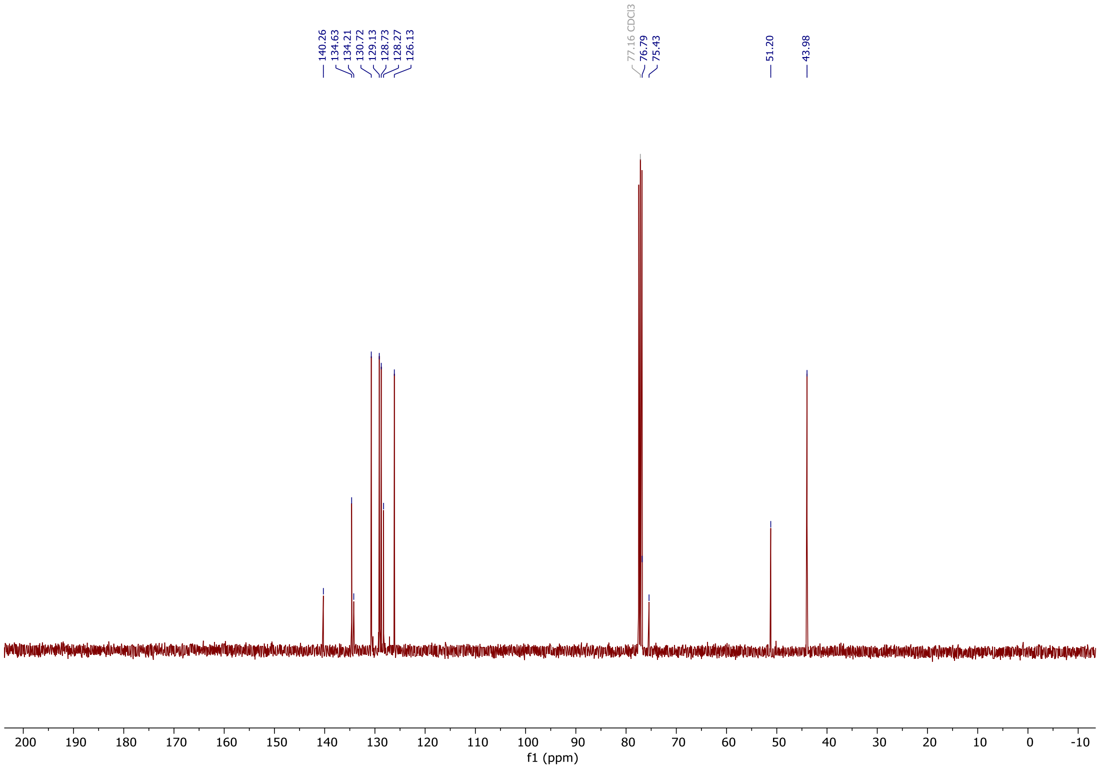

^{1}H COSY (500 MHz, Chloroform-*d*)

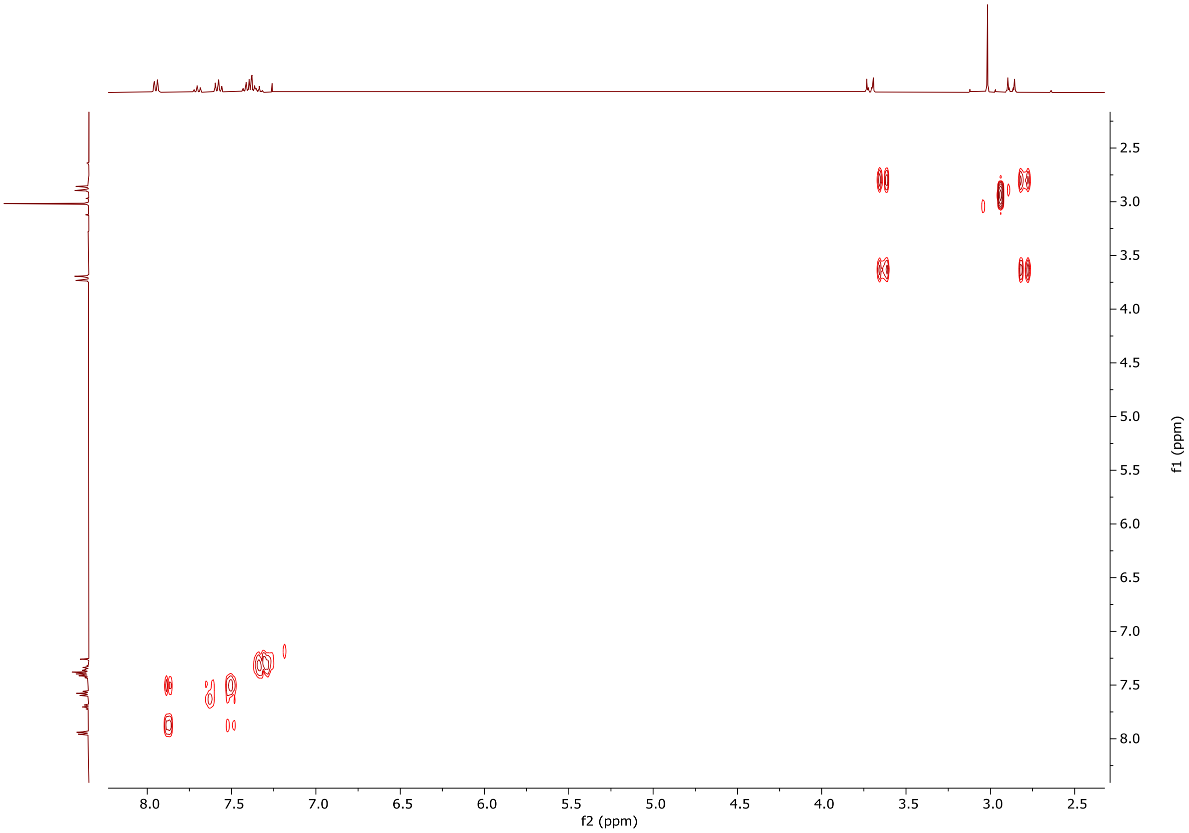

^{1}H/^{13}C HSQC (500/126 MHz, Chloroform-*d*)

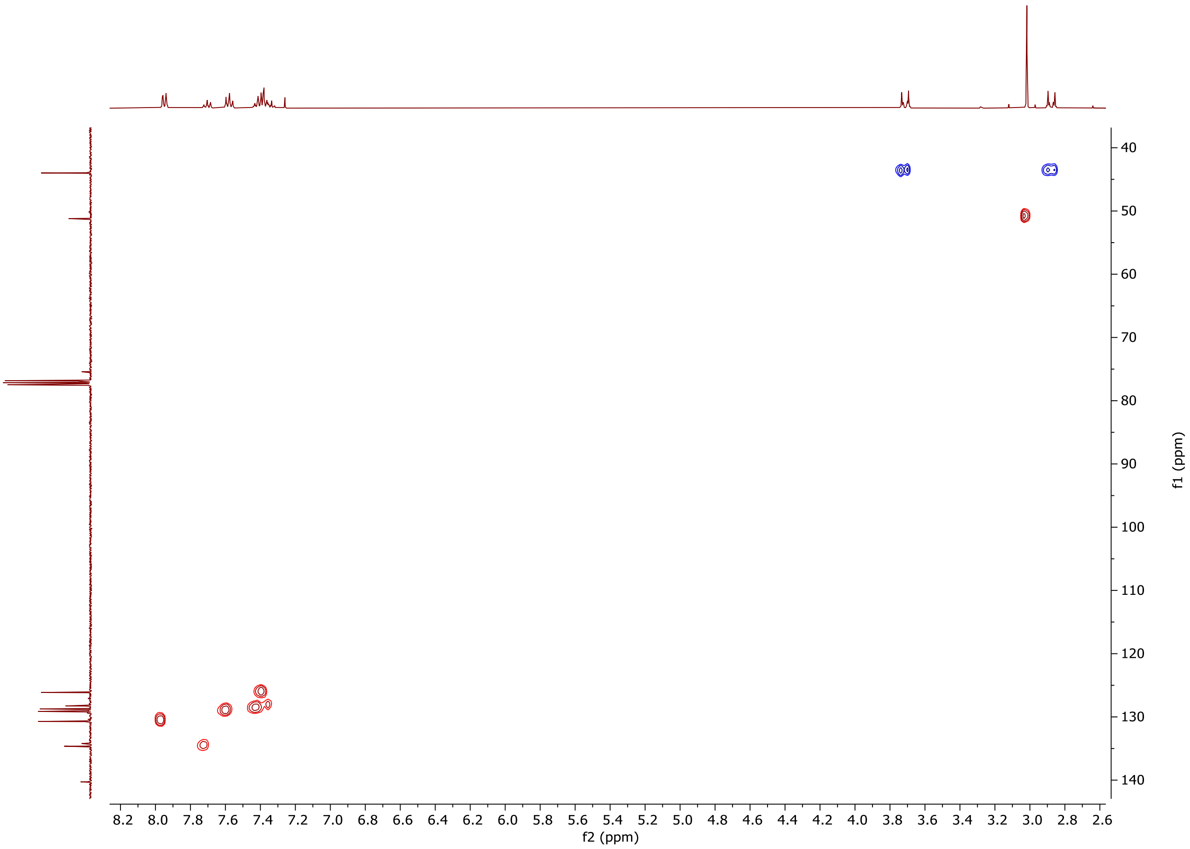

$^{1}H/^{13}C$ HMQC (500/126 MHz, Chloroform-*d*)

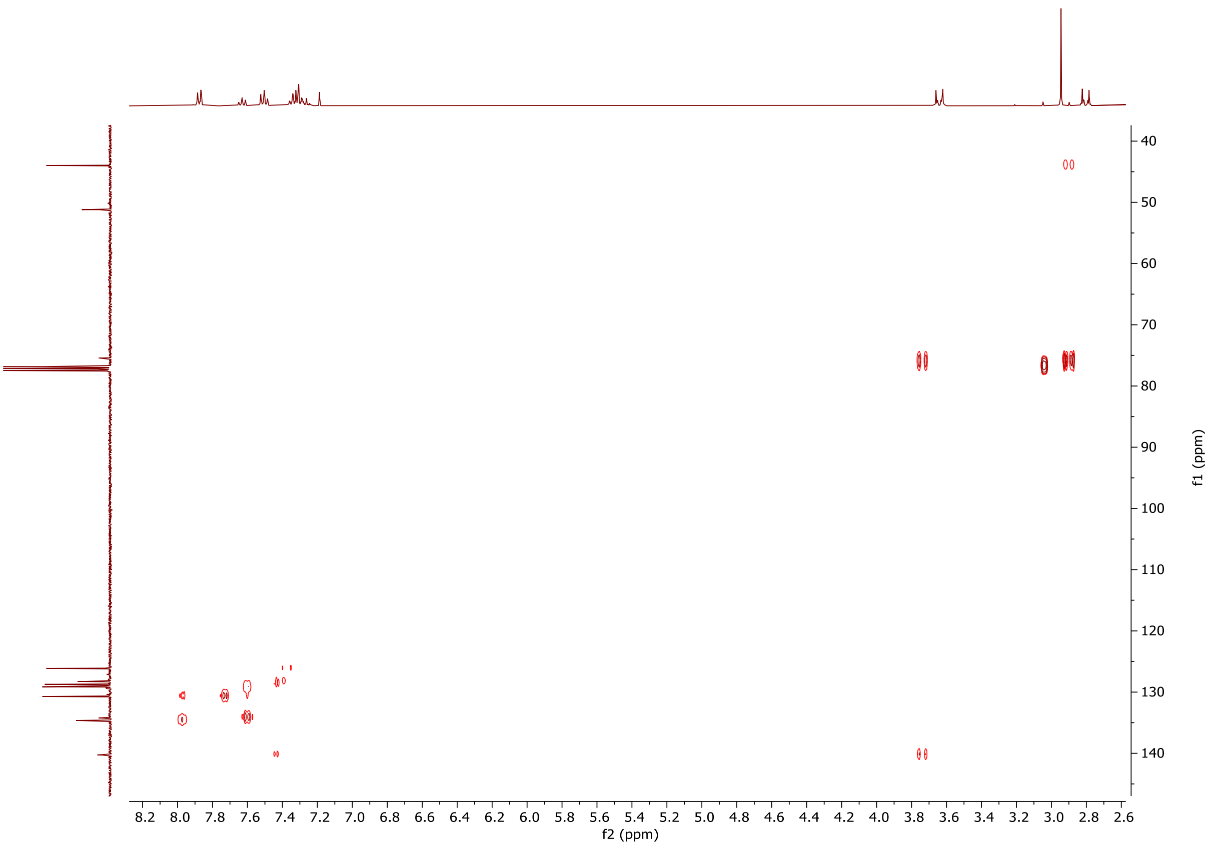

^{1}H NOSEY (400 MHz, Chloroform-*d*)

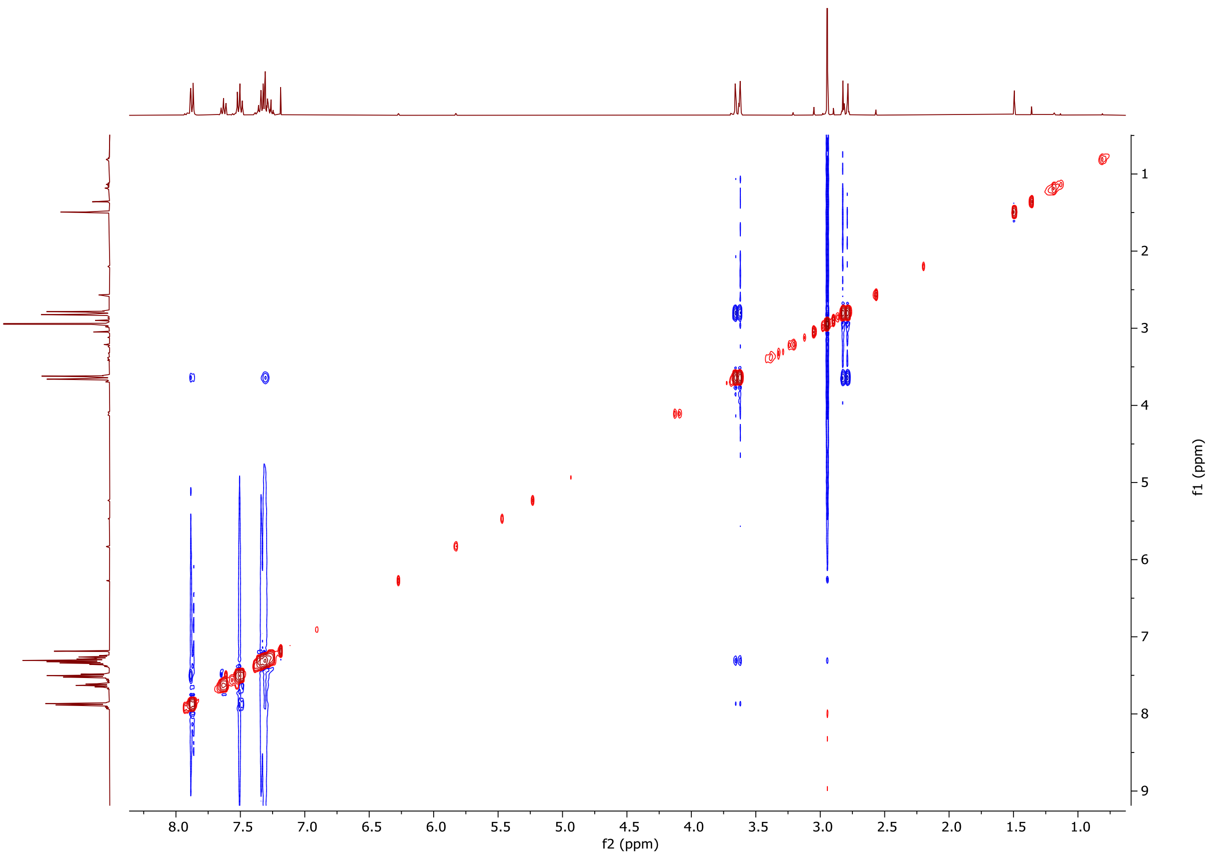

1-((2-fluorophenyl)sulfonyl)-3-phenylbicyclo[1.1.0]butane, 52

^{1}H NMR (500 MHz, Chloroform-*d*)

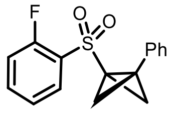

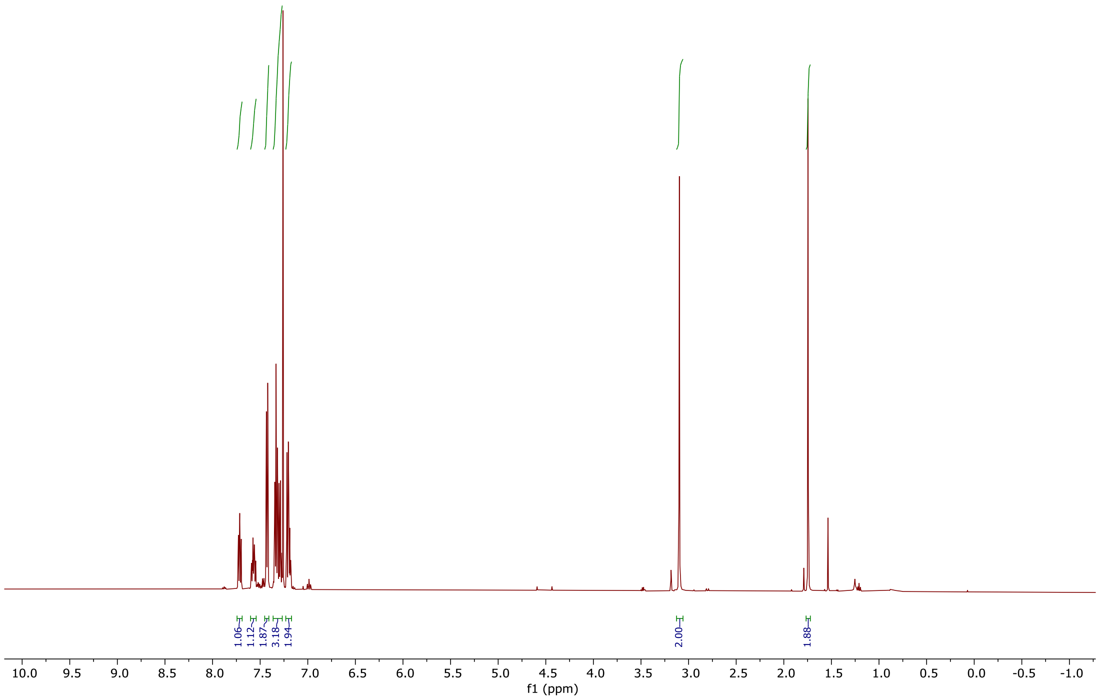

^{13}C NMR (126 MHz, Chloroform-*d*)

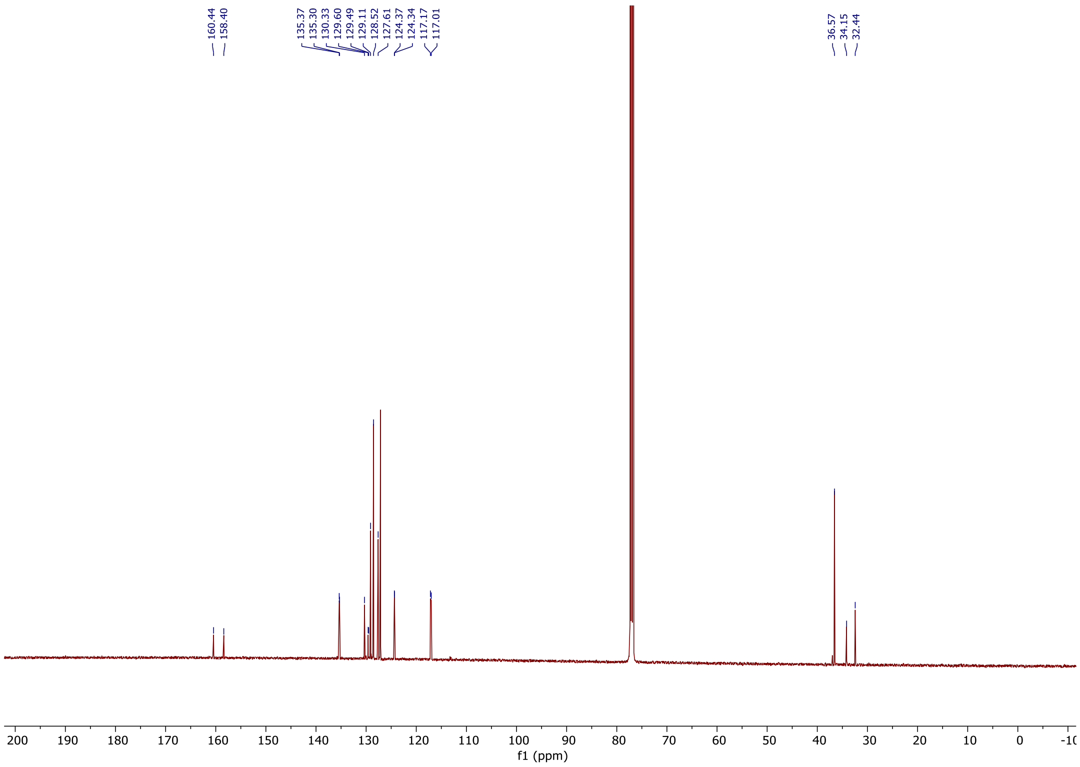

^{1}H COSY (500 MHz, Chloroform-*d*)

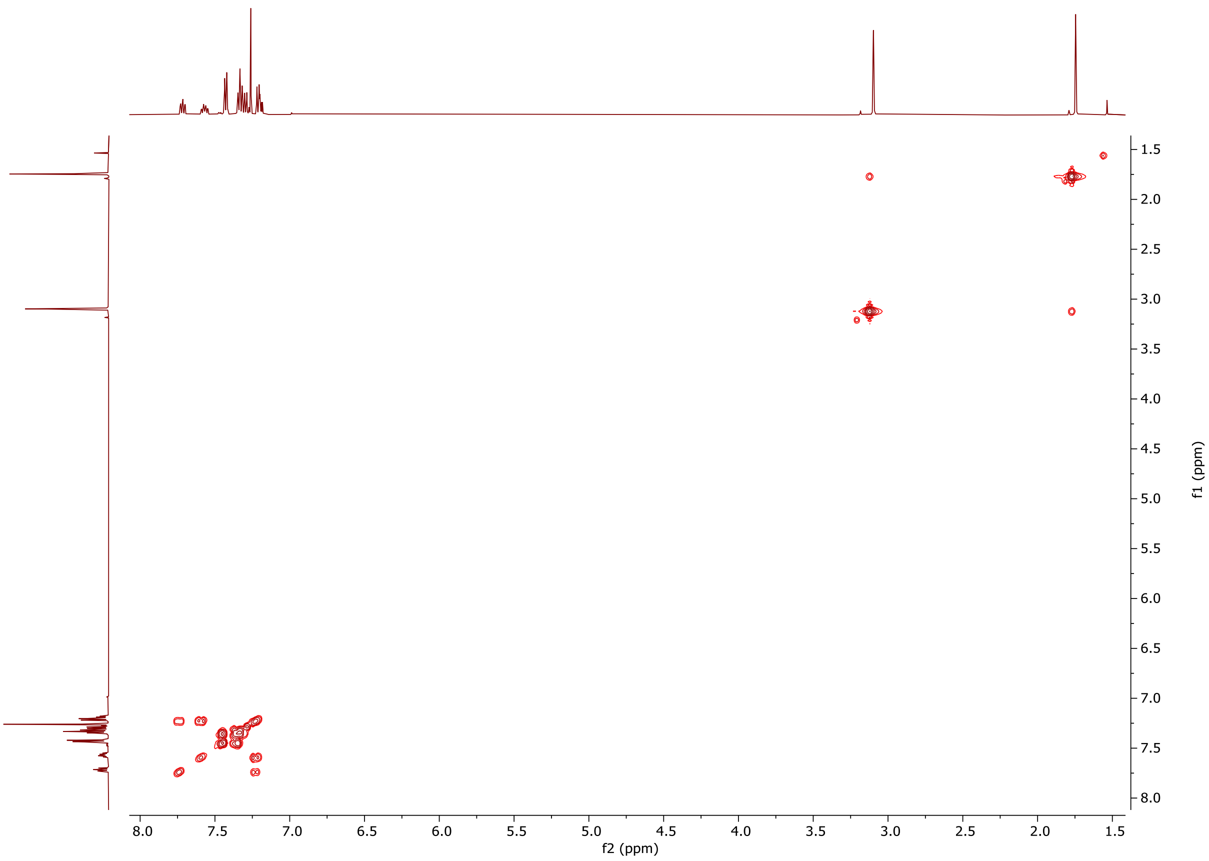

^{1}H/^{13}C HSQC (500/126 MHz, Chloroform-*d*)

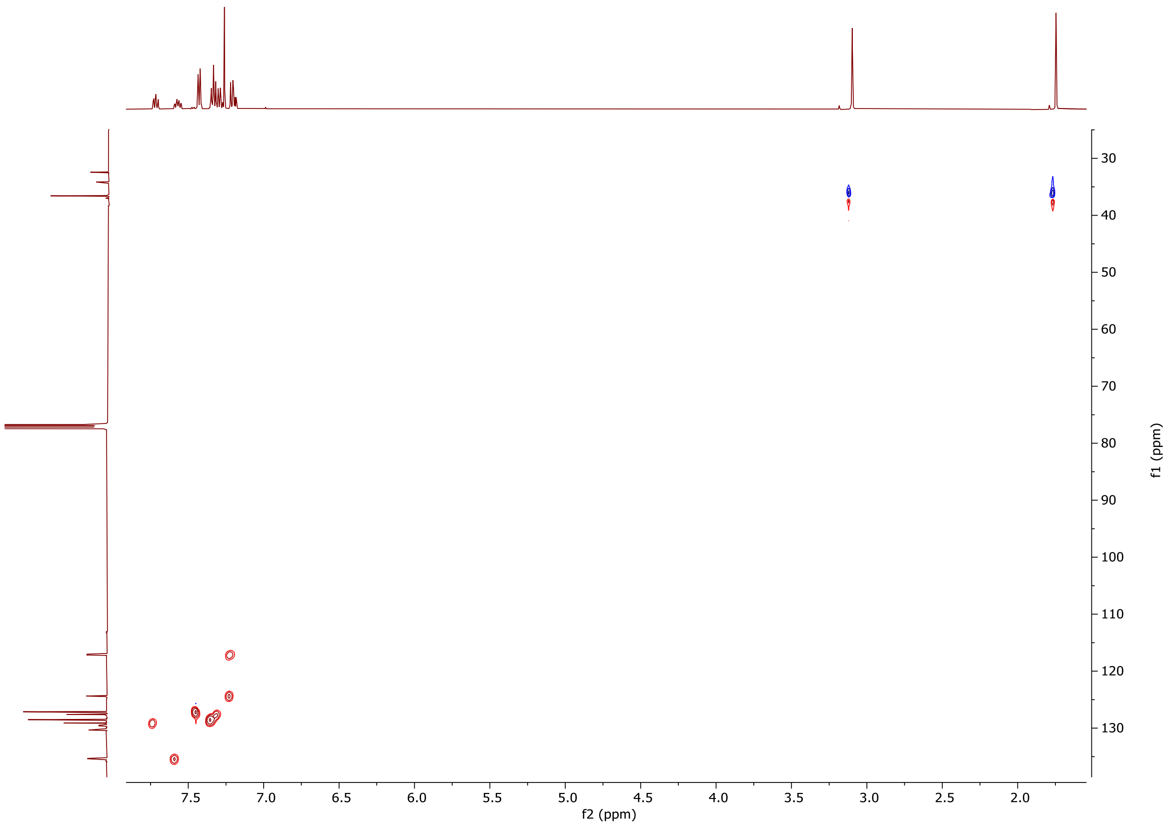

$^{1}H/^{13}C$ **HMBC** (500/126 MHz, Chloroform-*d*)

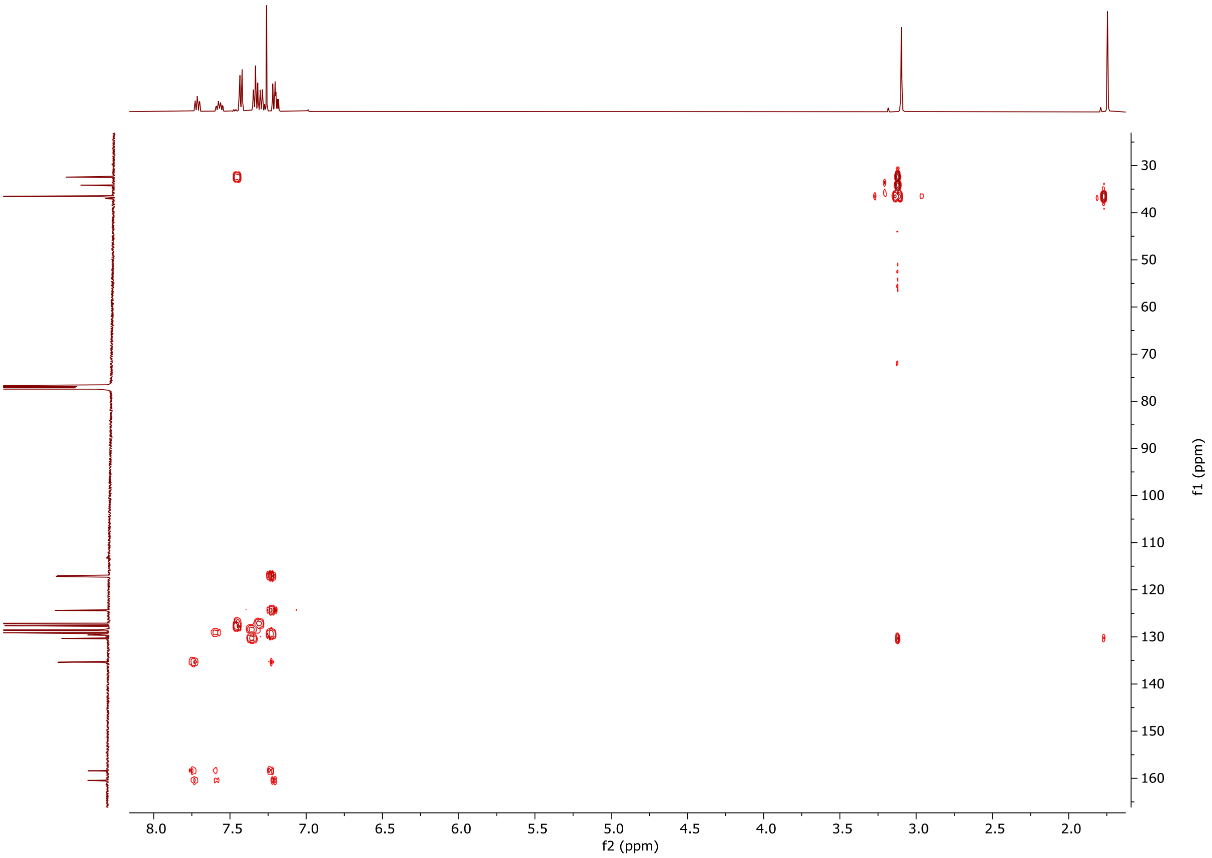

^{19}F **NMR** (470 MHz, Chloroform-*d*)

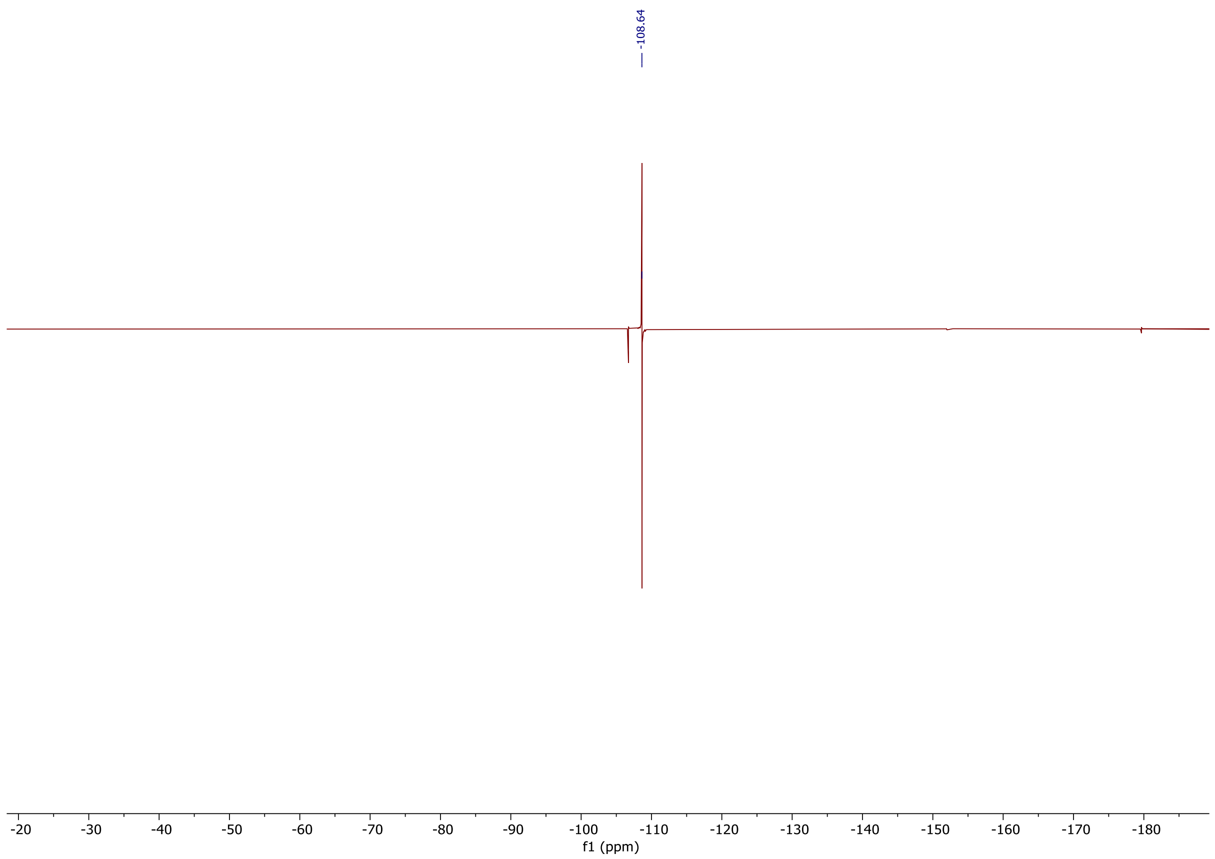
-108.64
-20
-30
-40
-50
-60
-70
-80
-90
-100
-110
-120
-130
-140
-150
-160
-170
-180
f1 (ppm)

Methyl 2'-((3-phenylbicyclo[1.1.0]butan-1-yl)sulfonyl)-[1,1'-biphenyl]-4-carboxylate, 53

^{1}H NMR (500 MHz, Chloroform-*d*)

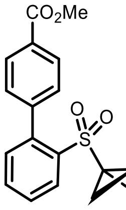

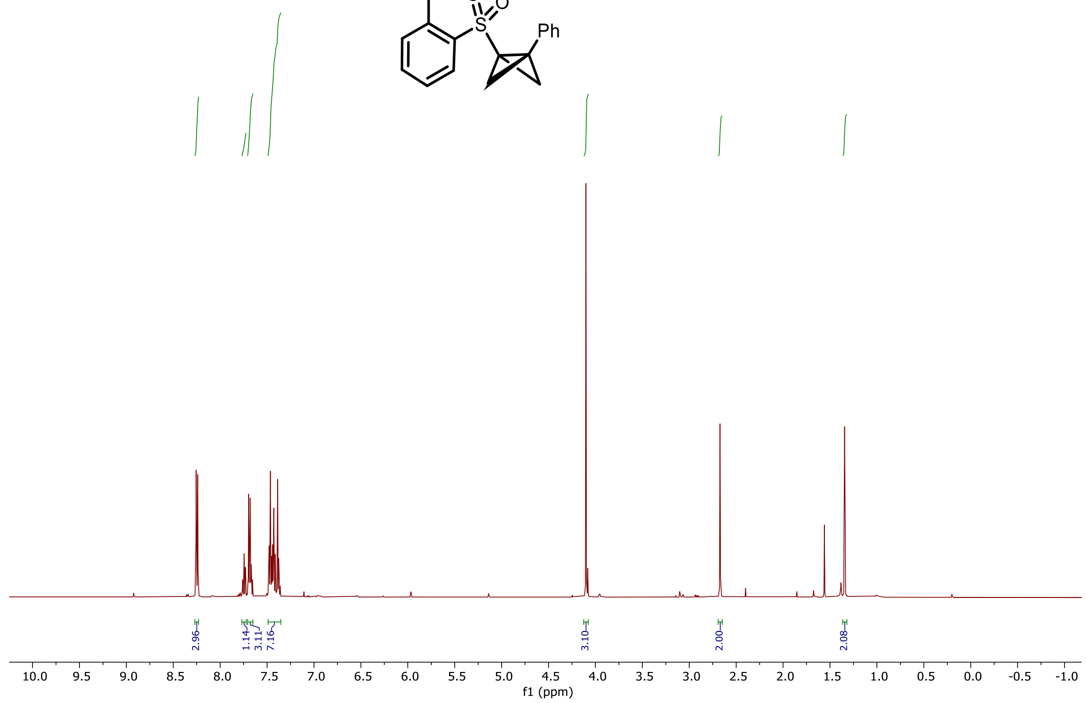

^{13}C NMR (126 MHz, Chloroform-*d*)

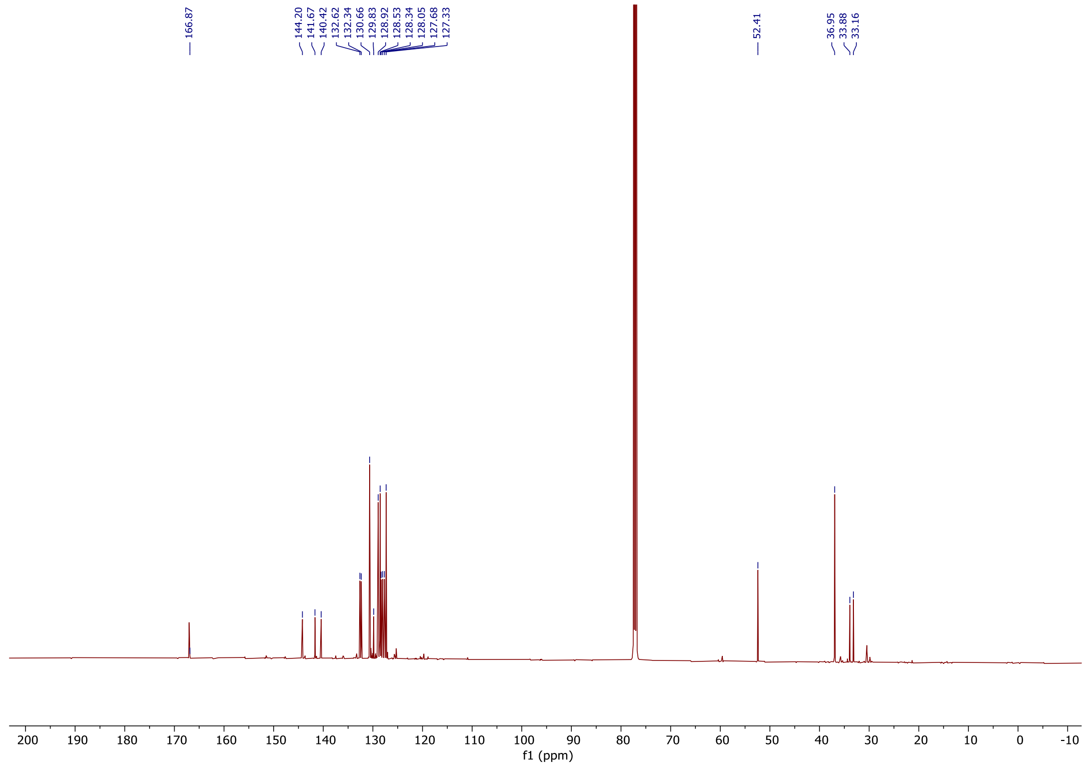

^{1}H COSY (500 MHz, Chloroform-*d*)

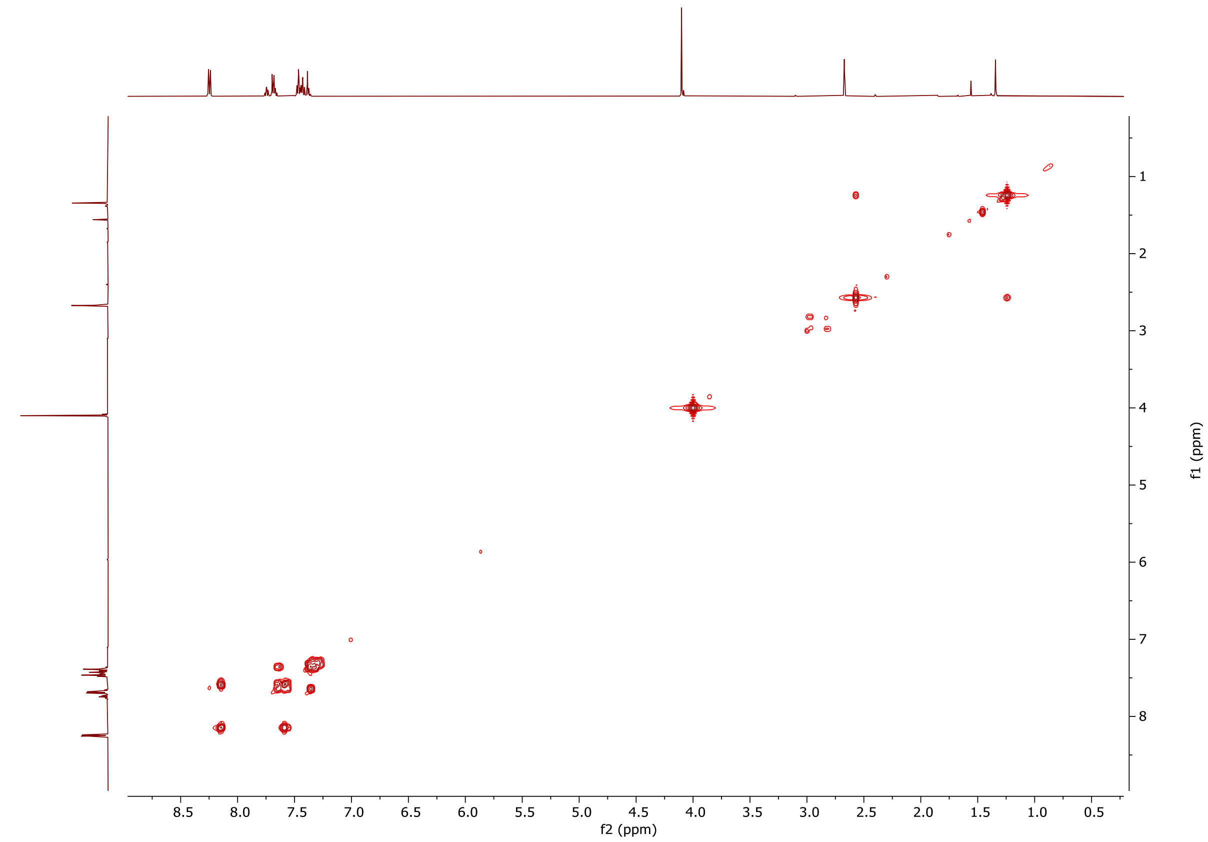

^{1}H/^{13}C HSQC (500/126 MHz, Chloroform-*d*)

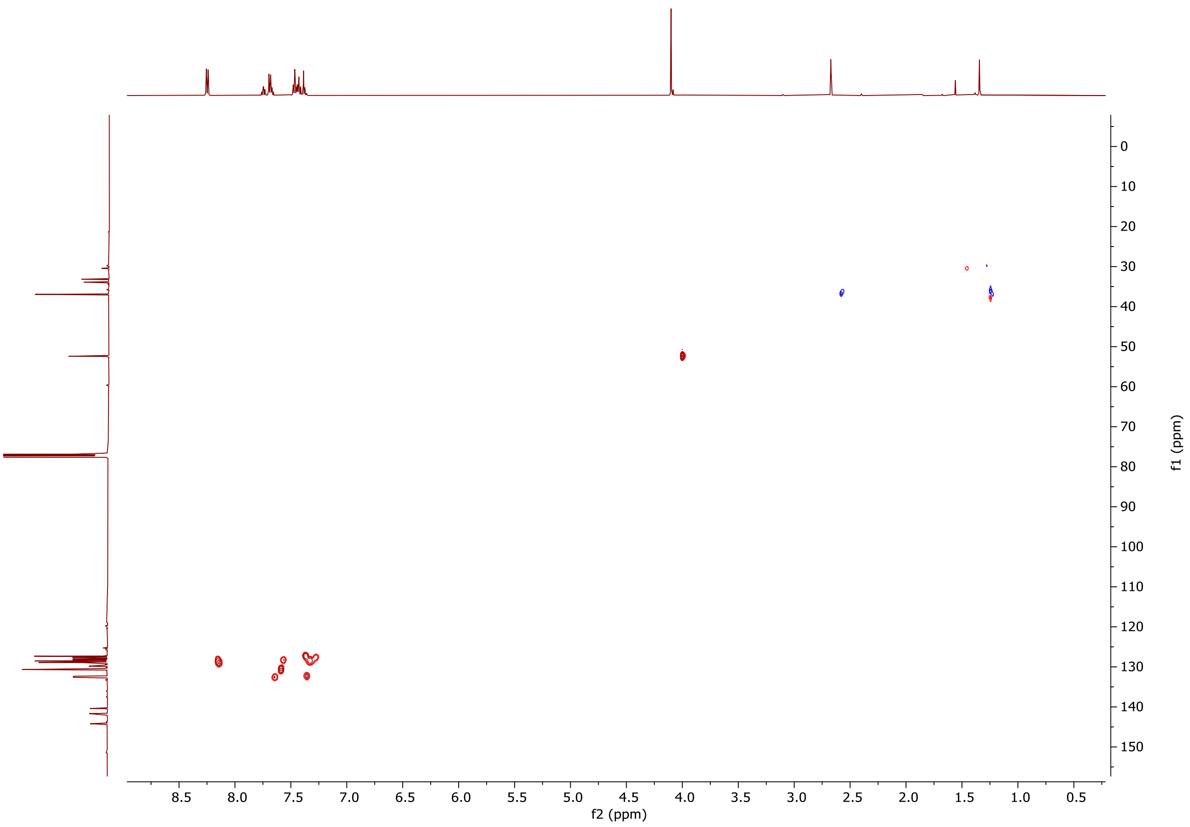

$^{1}H/^{13}C$ HMBC (500/126 MHz, Chloroform-*d*)

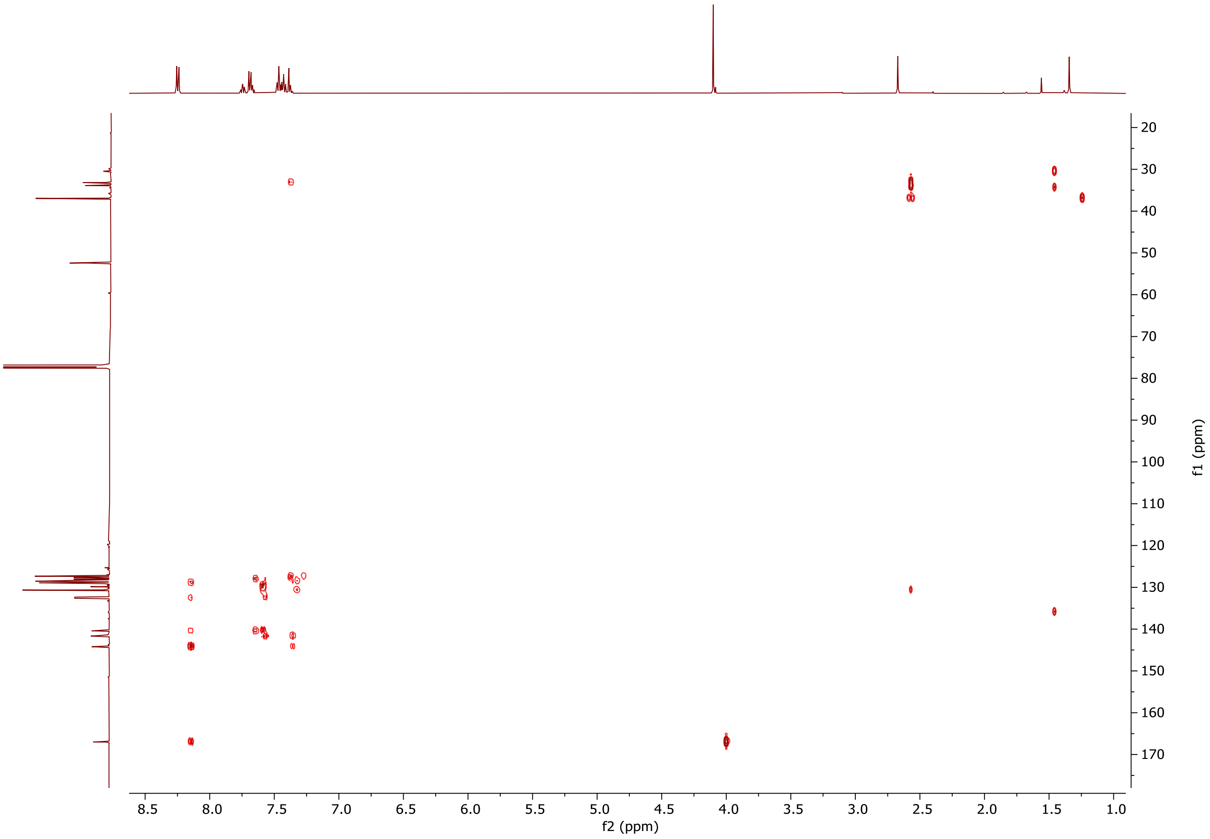

2,2-difluoro-*N*,*N*-diisopropyl-3-phenylbicyclo[1.1.1]pentane-1-carboxamide, 54

^{1}H NMR (500 MHz, Chloroform-*d*)

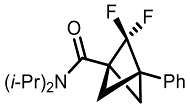

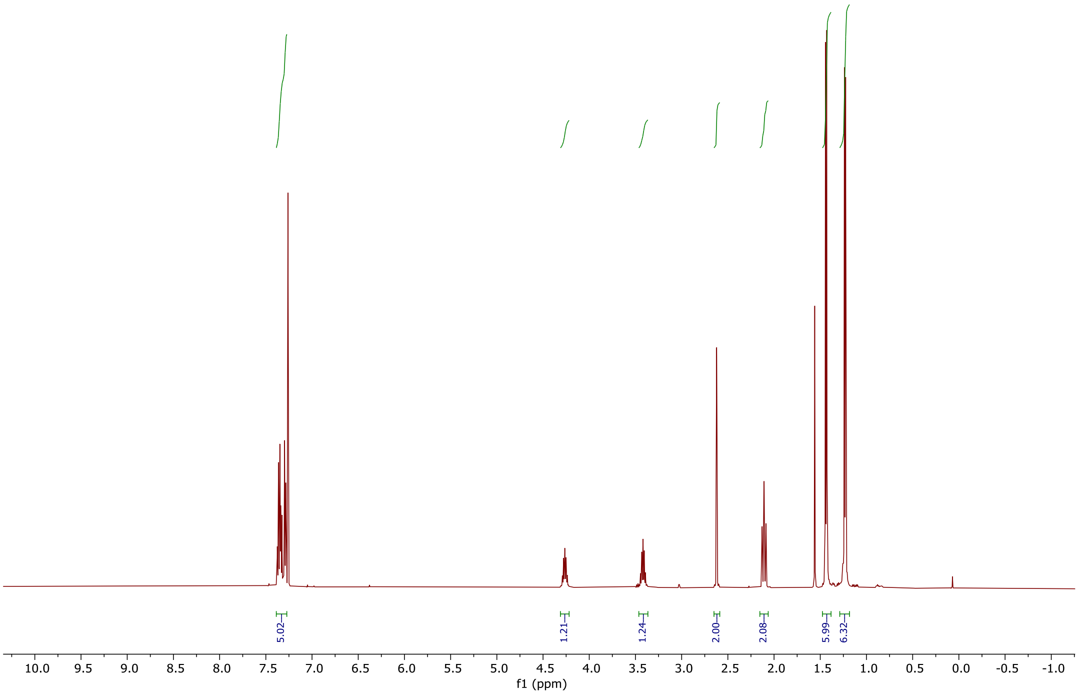

^{13}C NMR (126 MHz, Chloroform-*d*)

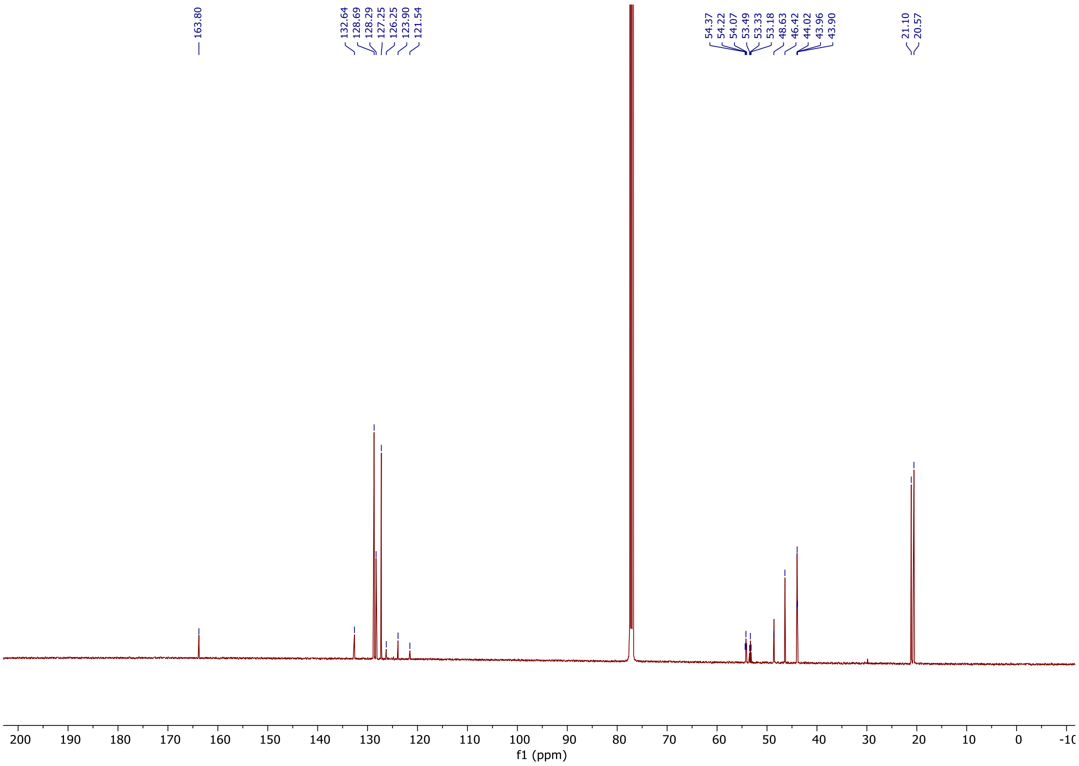

^{1}H COSY (500 MHz, Chloroform-*d*)

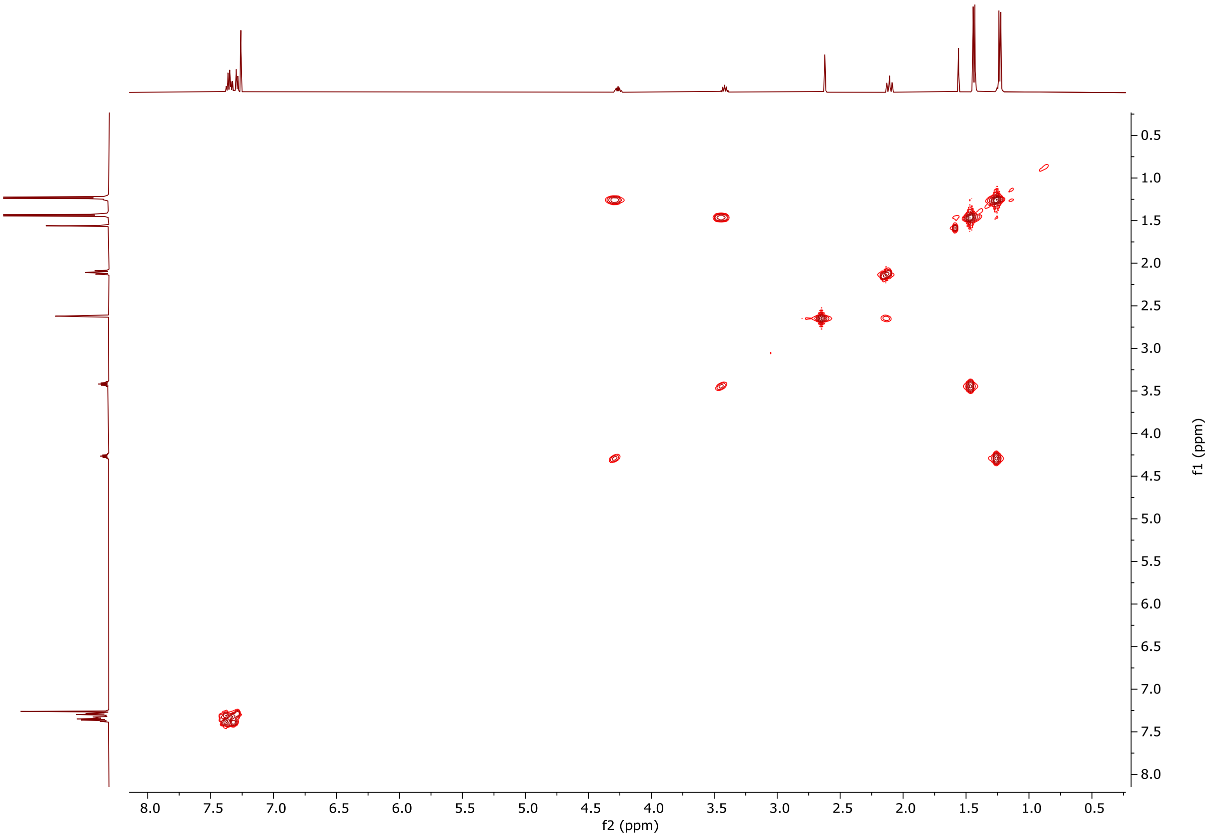

^{1}H/^{13}C HSQC (500/126 MHz, Chloroform-*d*)

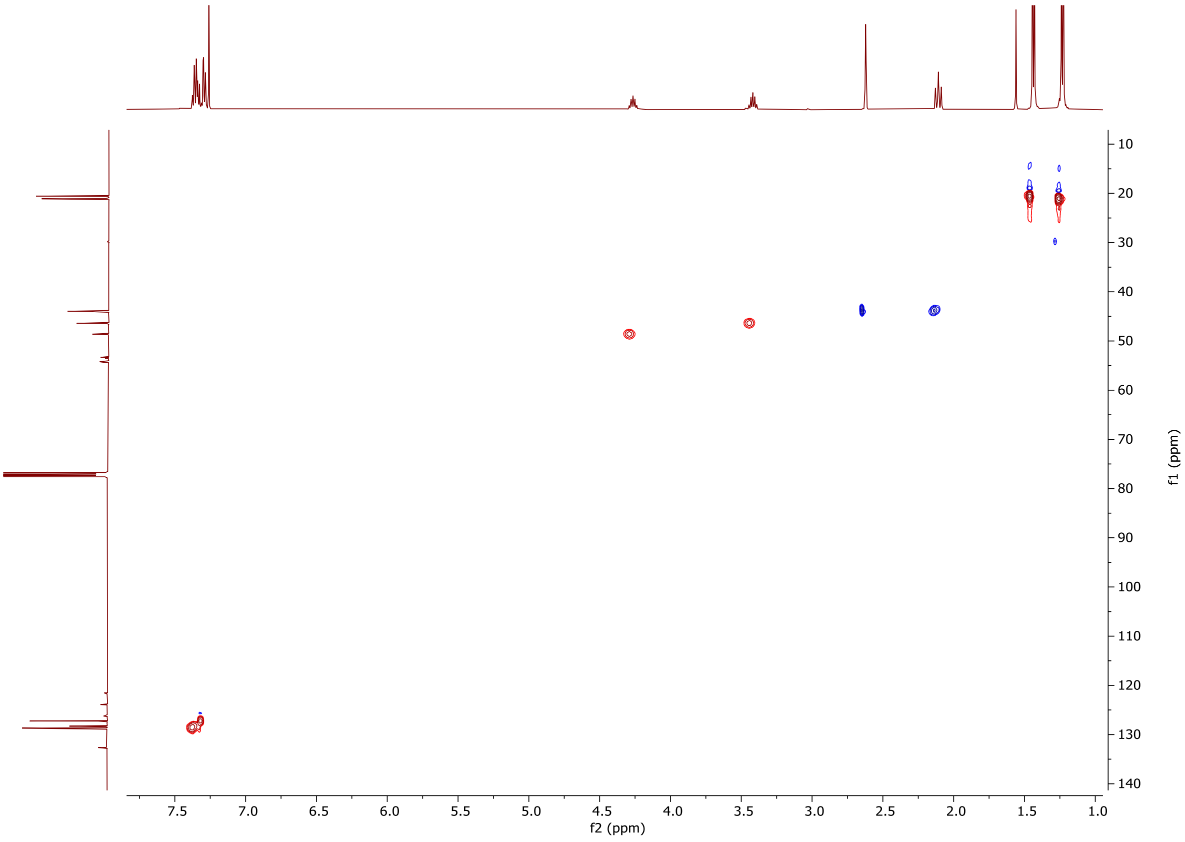

$^{1}H/^{13}C$ HMBC (500/126 MHz, Chloroform-*d*)

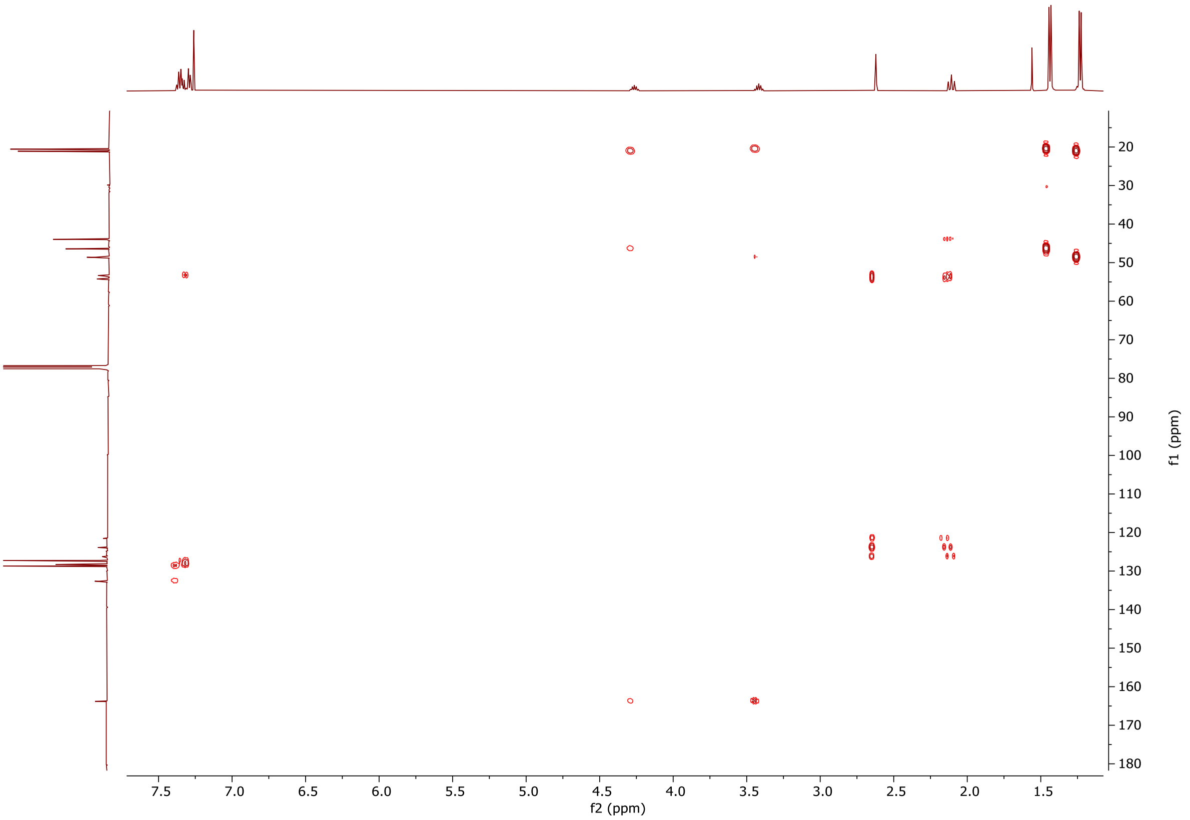

^{19}F NMR (470 MHz, Chloroform-*d*)

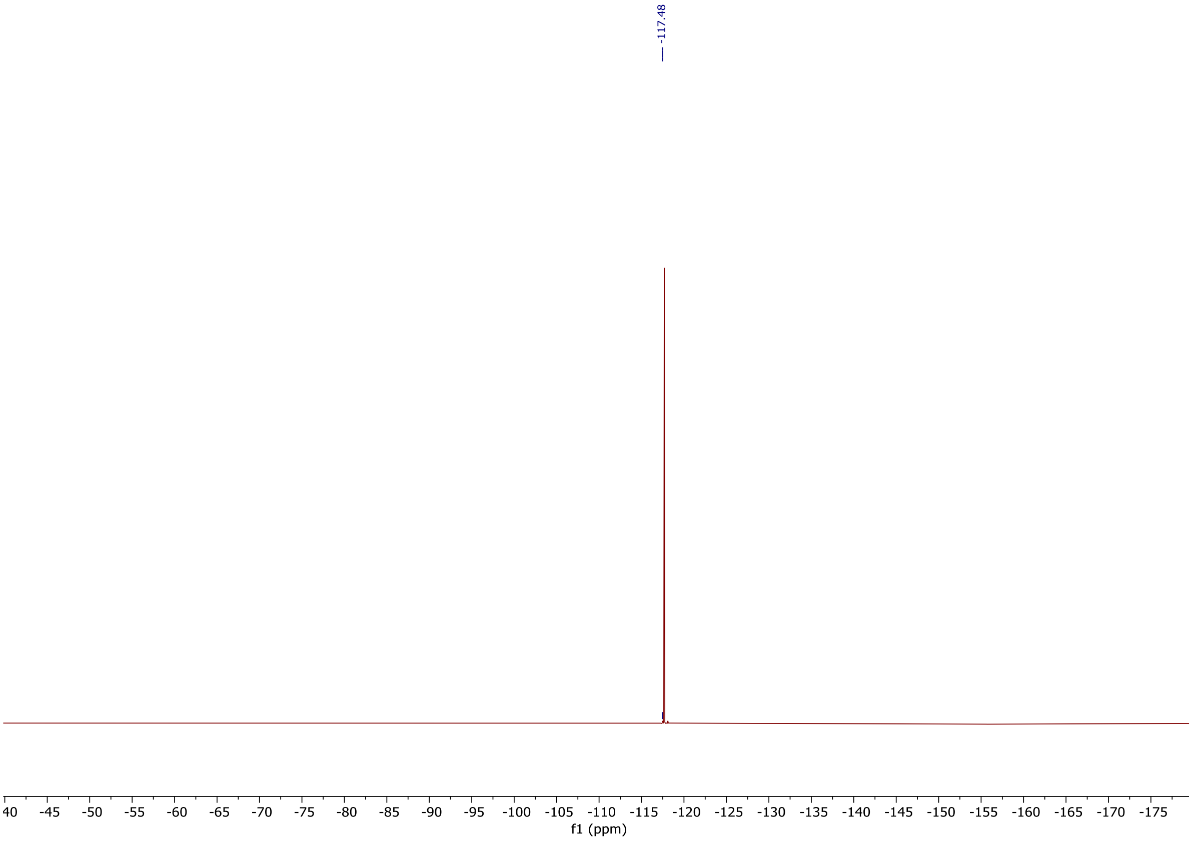

2,2-difluoro-N,N-diisopropyl-3-(4-(trifluoromethyl)phenyl)bicyclo[1.1.1]pentane-1-carboxamide, 56

^{1}H NMR (500 MHz, Chloroform-*d*)

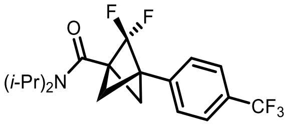

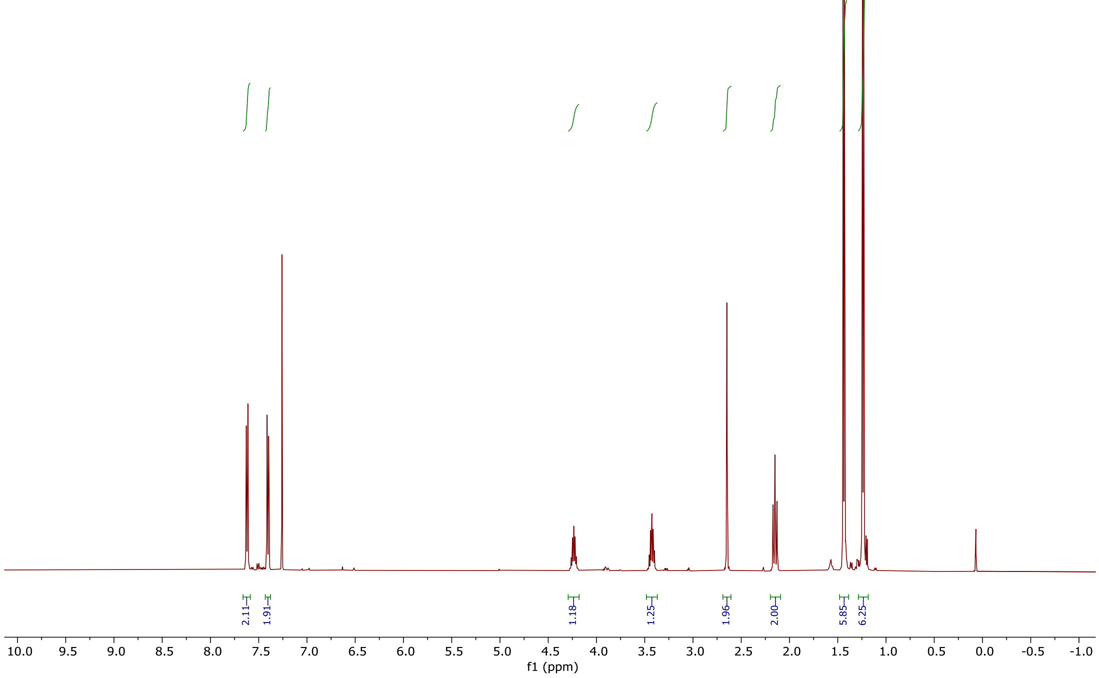

^{13}C NMR (126 MHz, Chloroform-*d*)

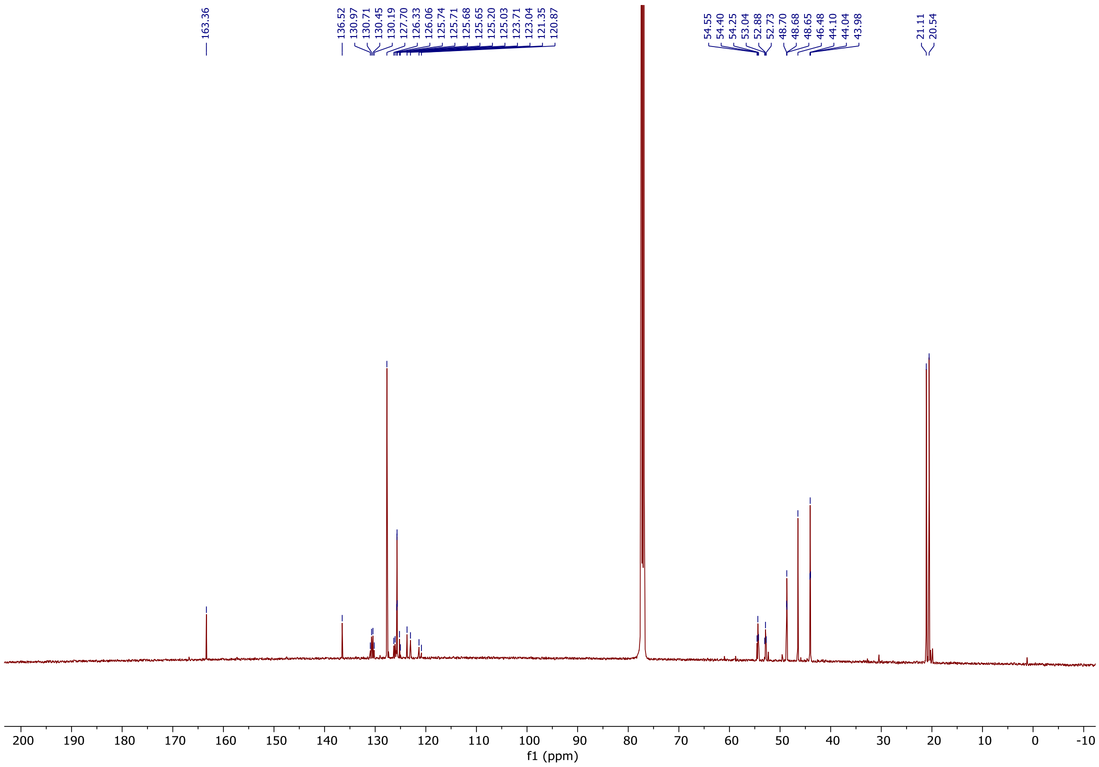

^{1}H COSY (500 MHz, Chloroform-*d*)

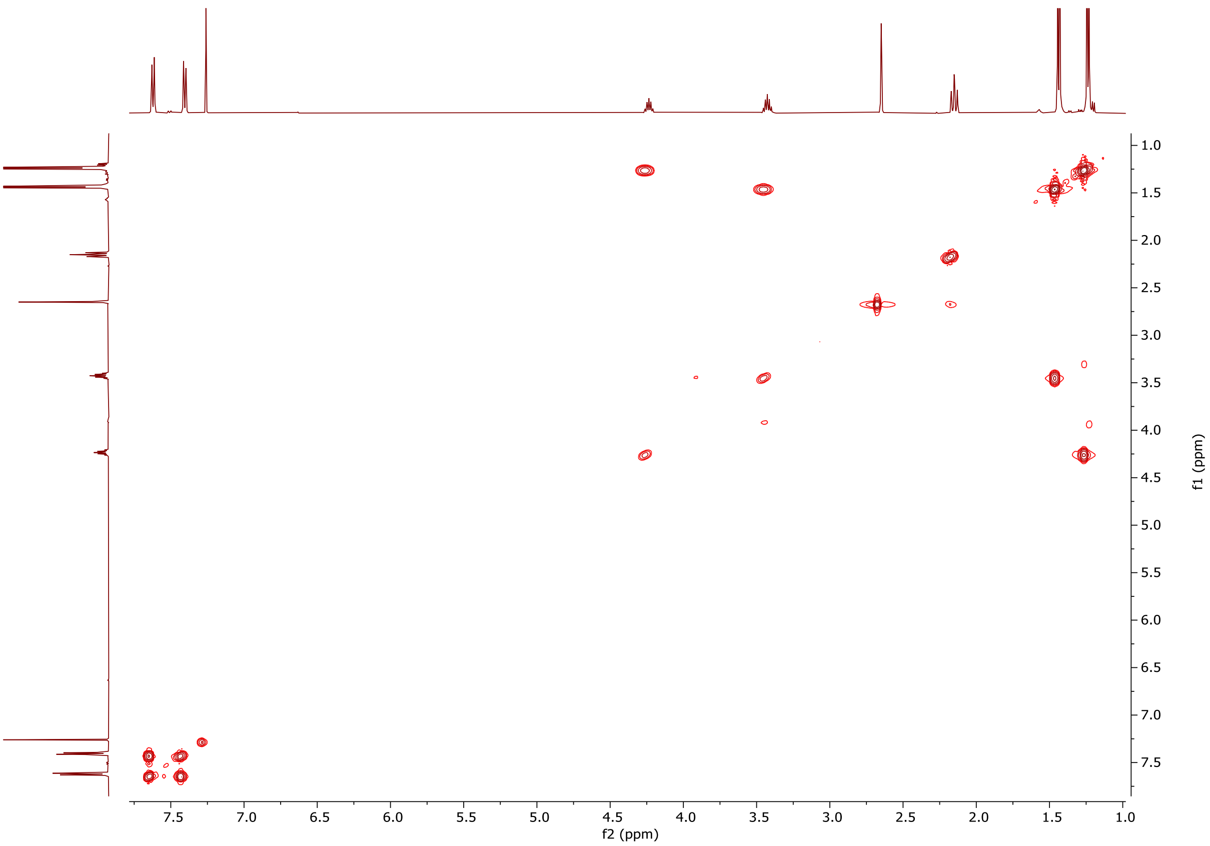

^{1}H/^{13}C HSQC (500/126 MHz, Chloroform-*d*)

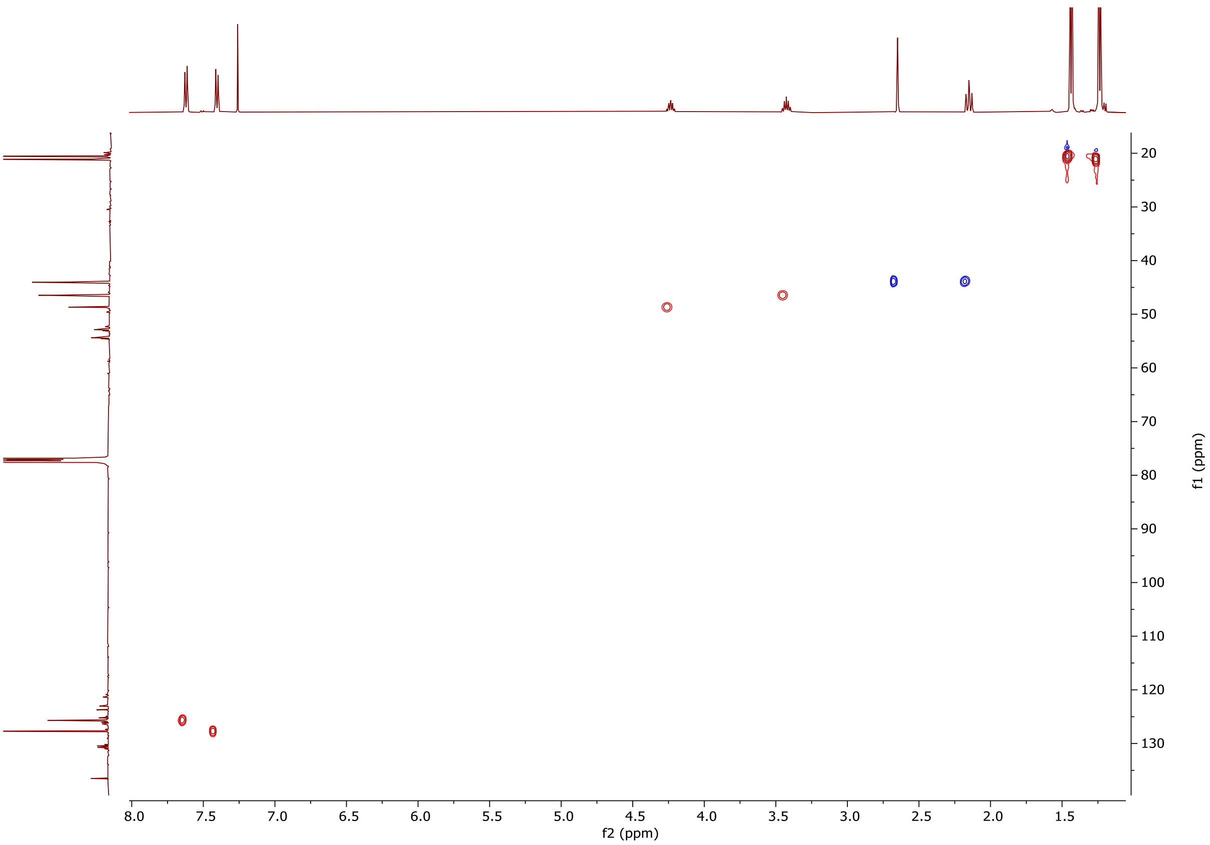

$^{1}H/^{13}C$ HMBC (500/126 MHz, Chloroform-*d*)

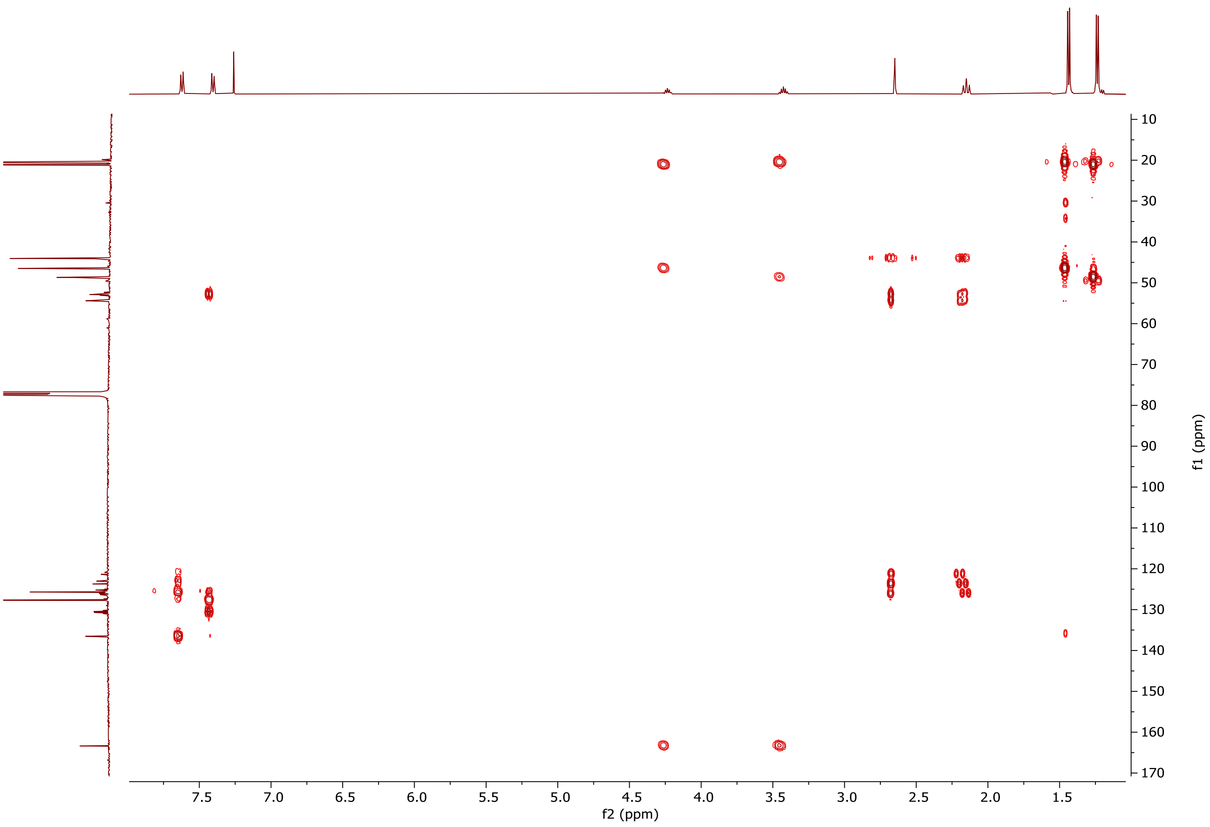

^{19}F NMR (470 MHz, Chloroform-*d*)

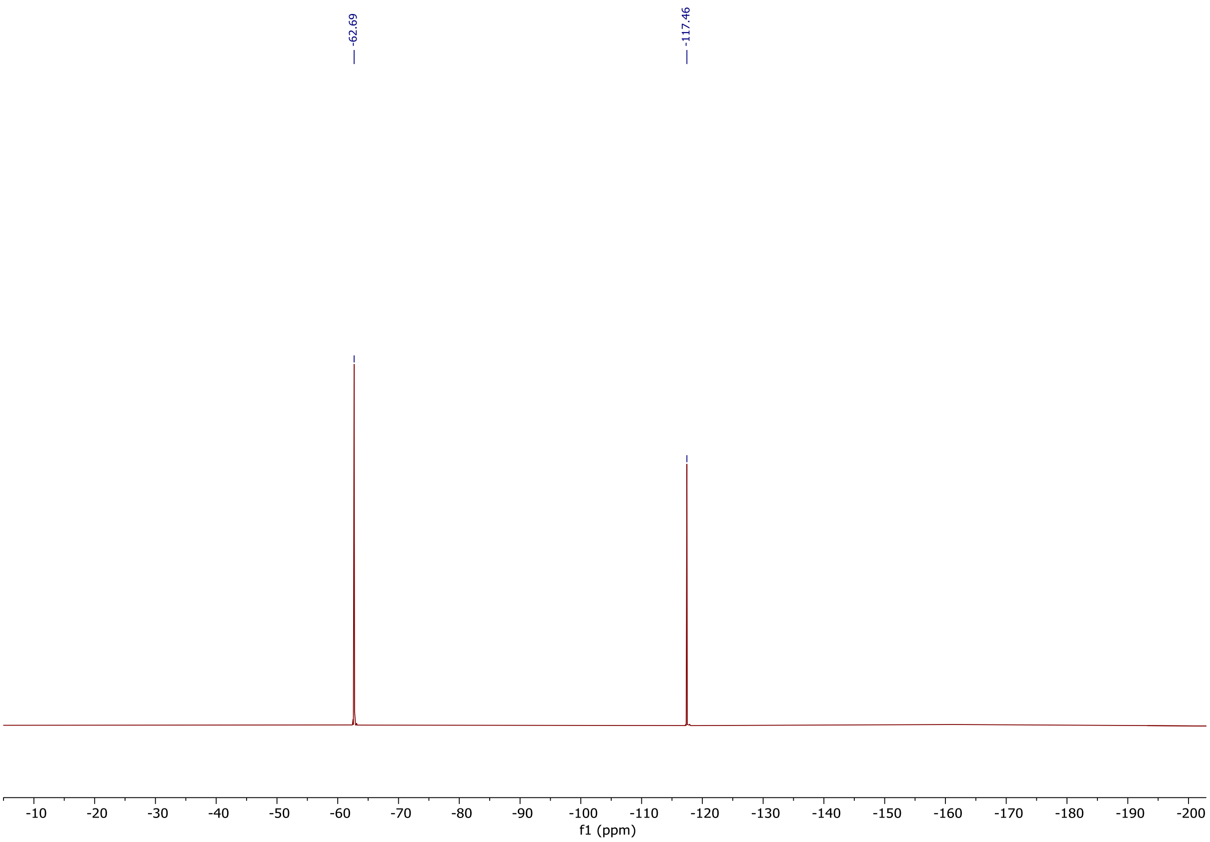

2,2-difluoro-N,N-diisopropyl-3-(4-(trifluoromethyl)phenyl)bicyclo[1.1.1]pentane-1-carboxamide, 57

^{1}H NMR (500 MHz, Chloroform-*d*)

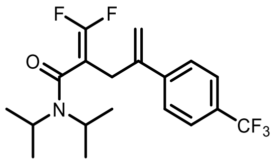

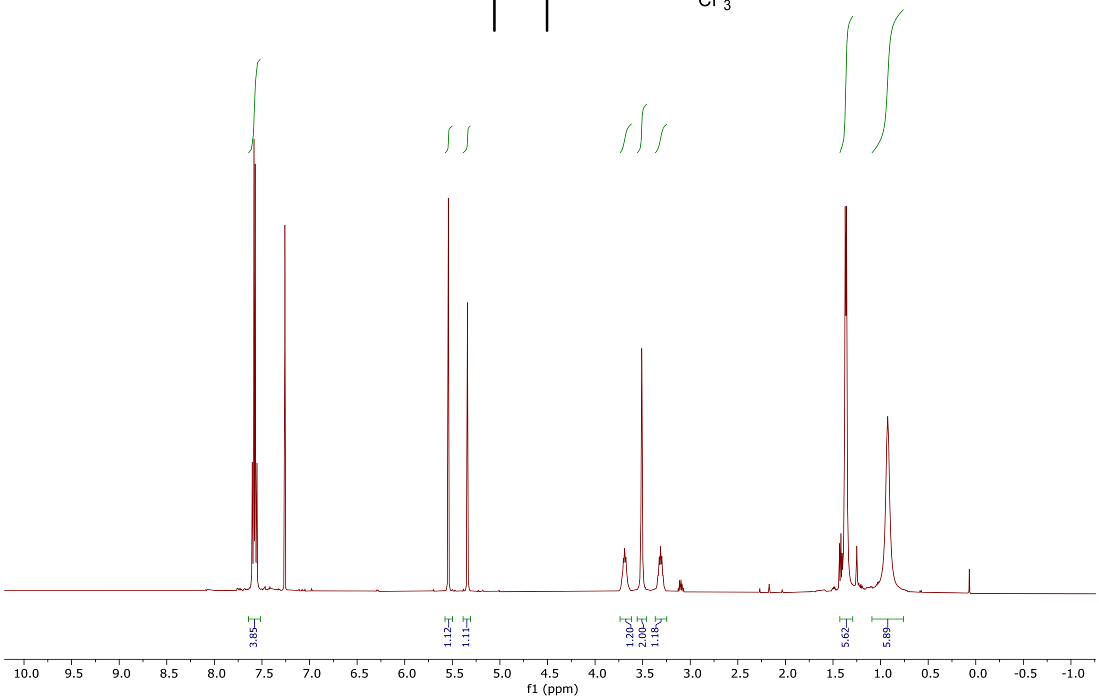

^{13}C NMR (126 MHz, Chloroform-*d*)

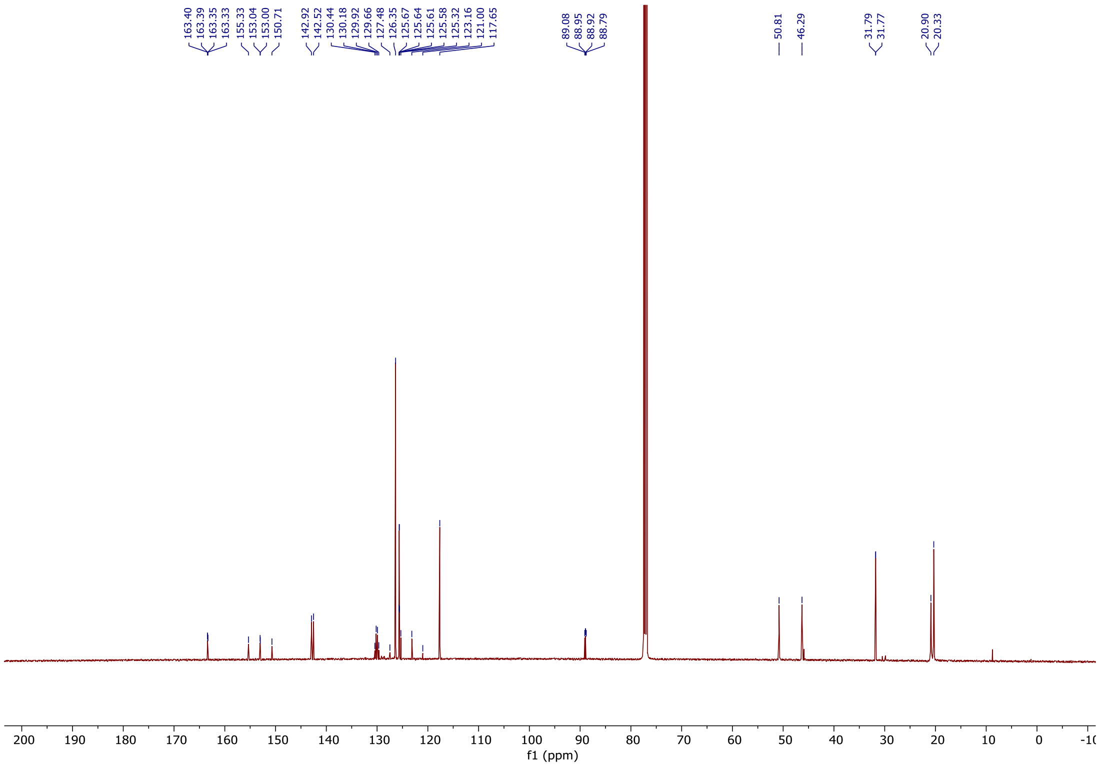

¹H COSY (500 MHz, Chloroform-*d*)

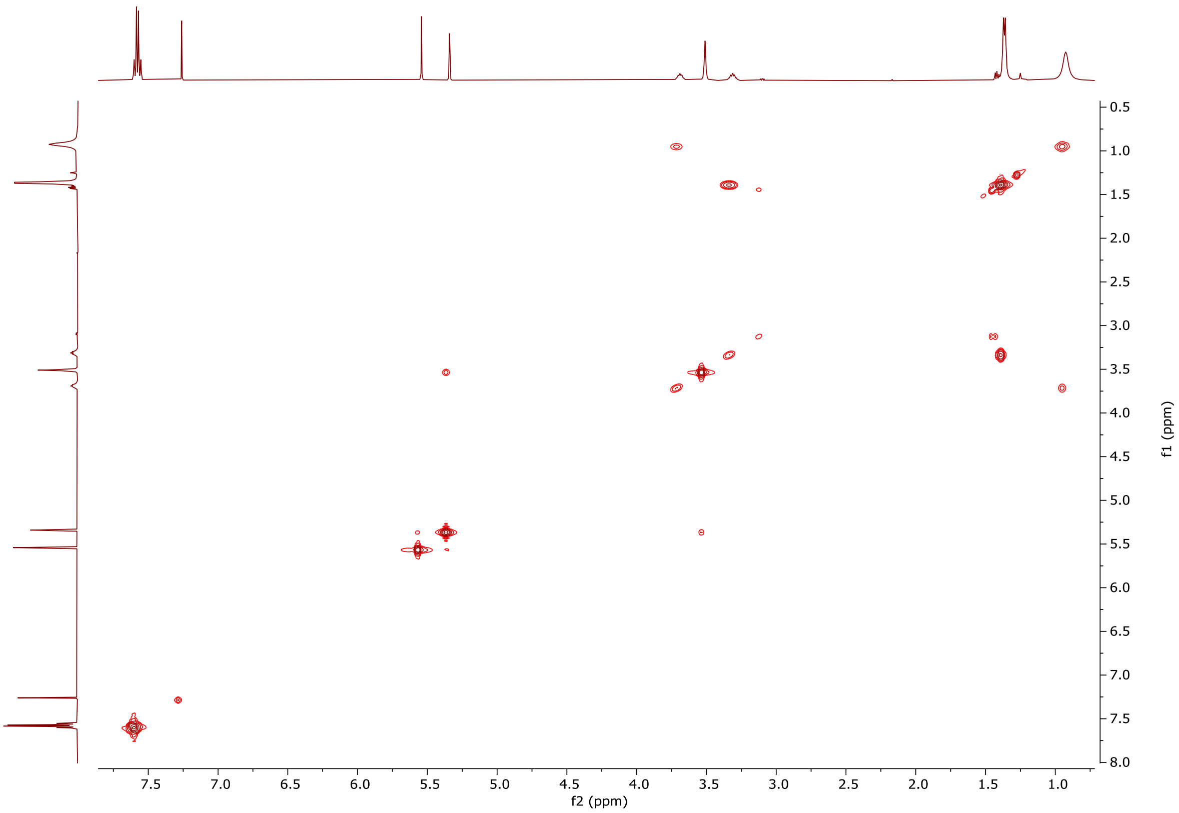

¹H/¹³C HSQC (500/126 MHz, Chloroform-*d*)

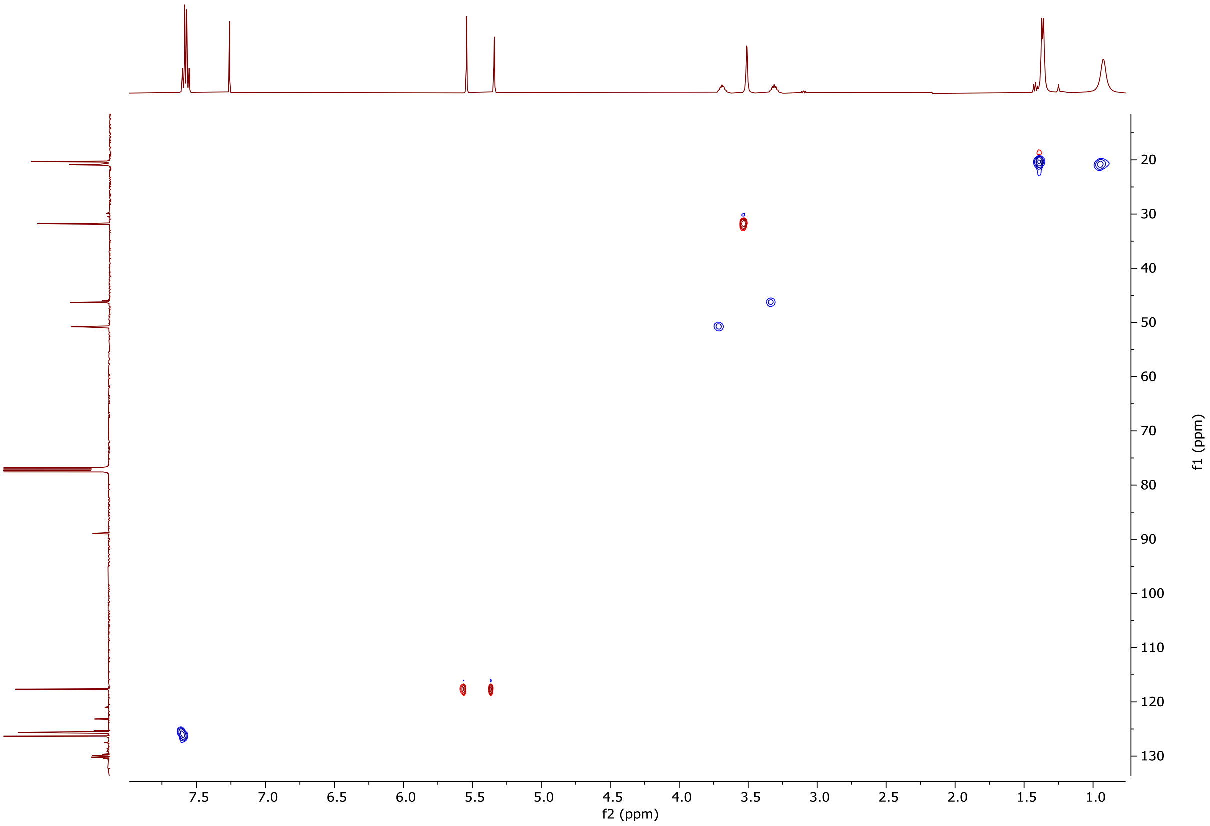

$^{1}H/^{13}C$ HMBC (500/126 MHz, Chloroform-*d*)

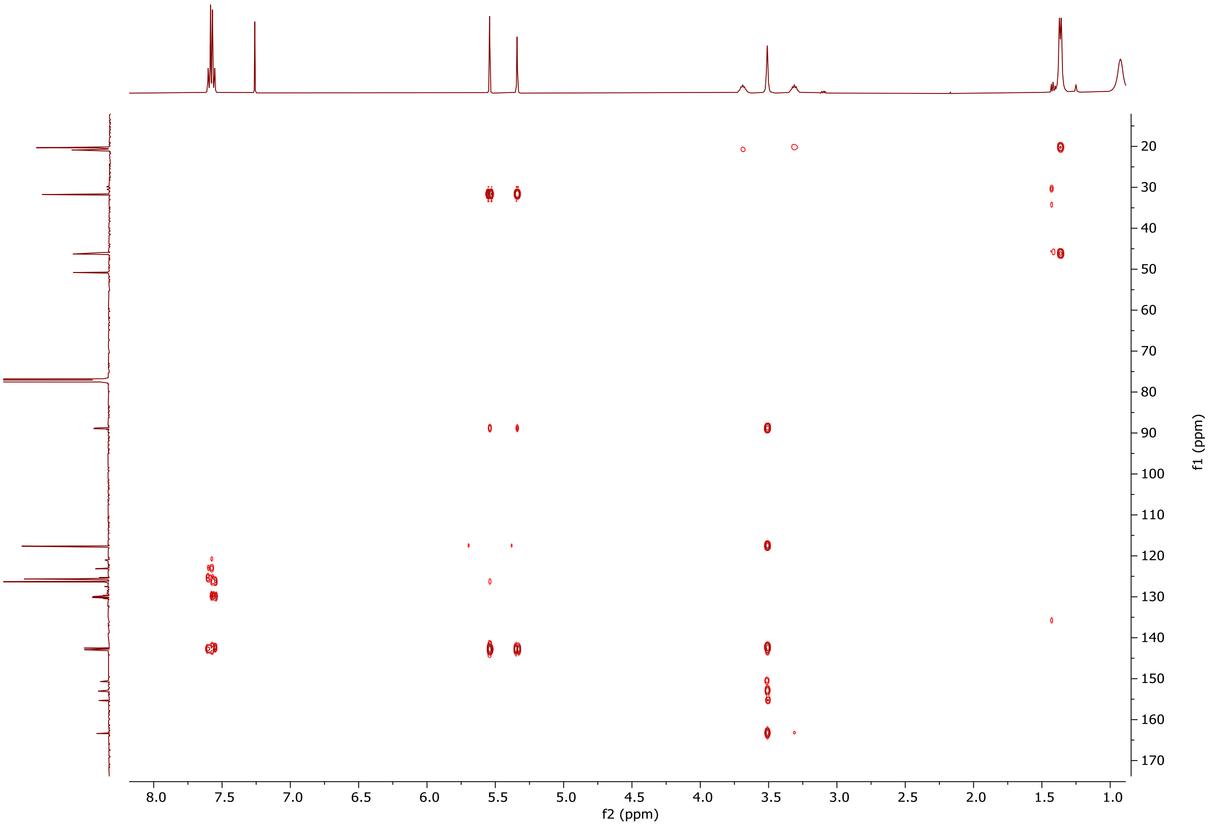

^{19}F NMR (470 MHz, Chloroform-*d*)

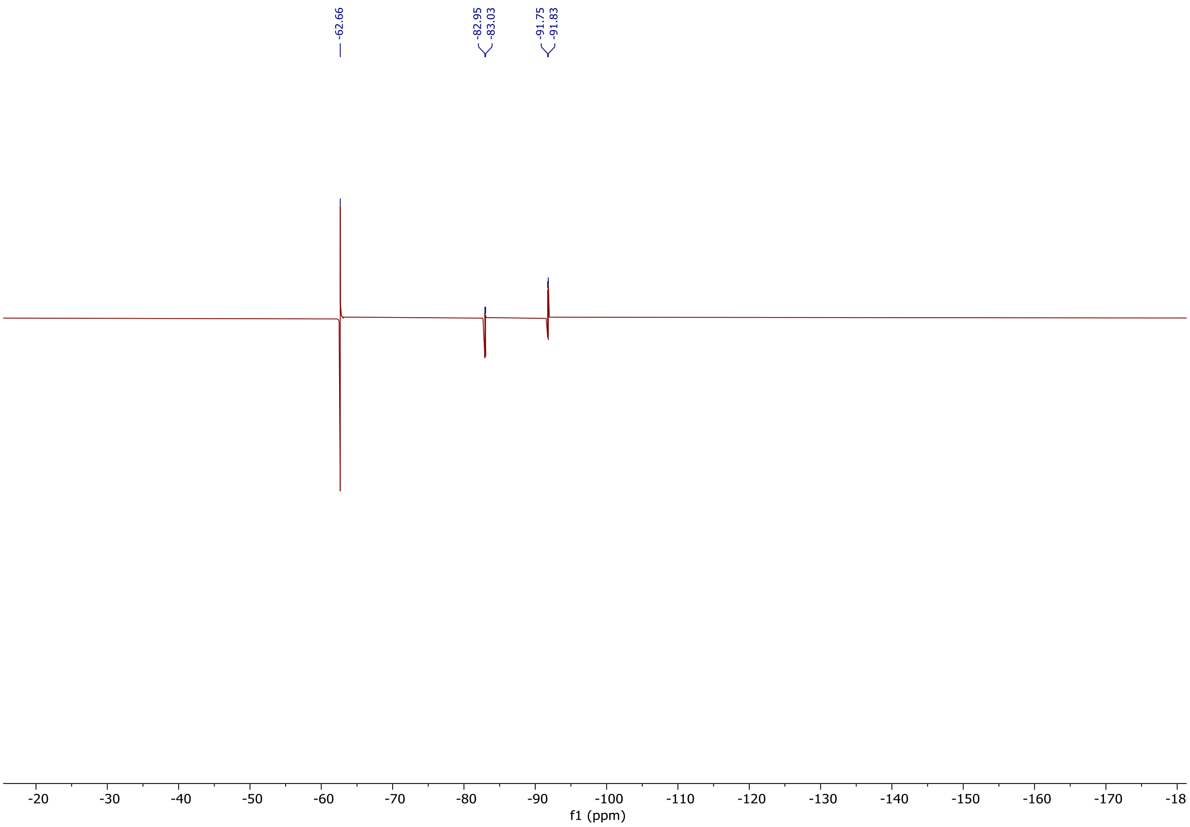

2,2-difluoro-3-(4-(trifluoromethyl)phenyl)bicyclo[1.1.1]pentane-1-carboxylic acid, 58

^{1}H NMR (500 MHz, Chloroform-*d*)

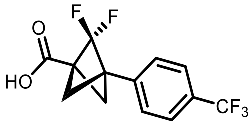

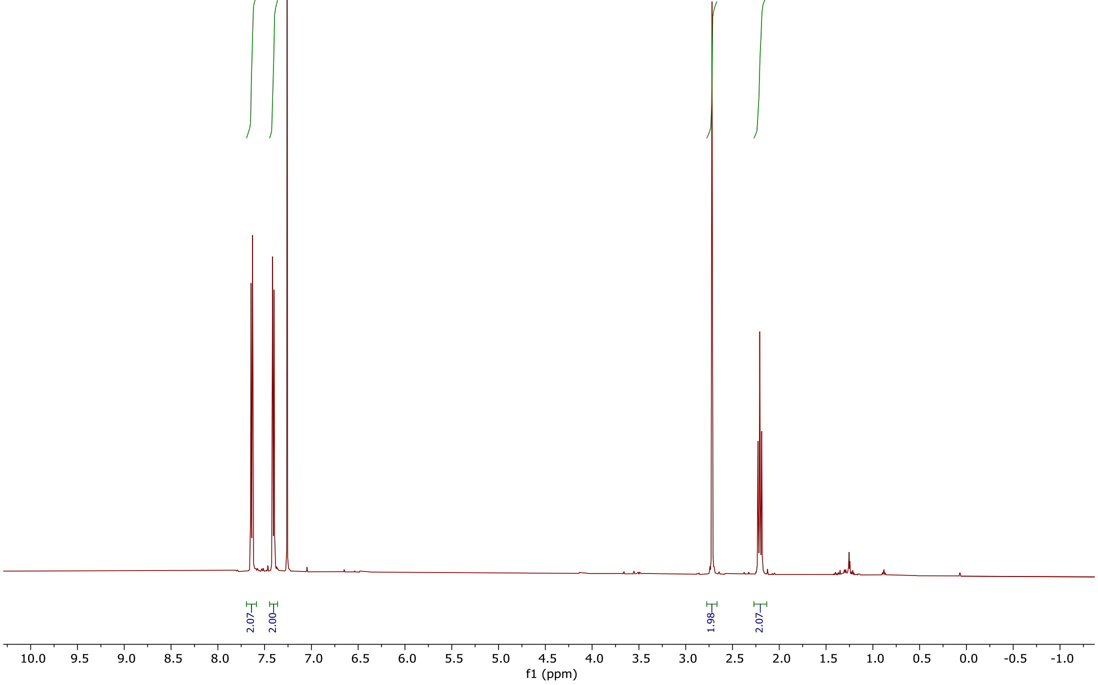

^{13}C NMR (126 MHz, Chloroform-*d*)

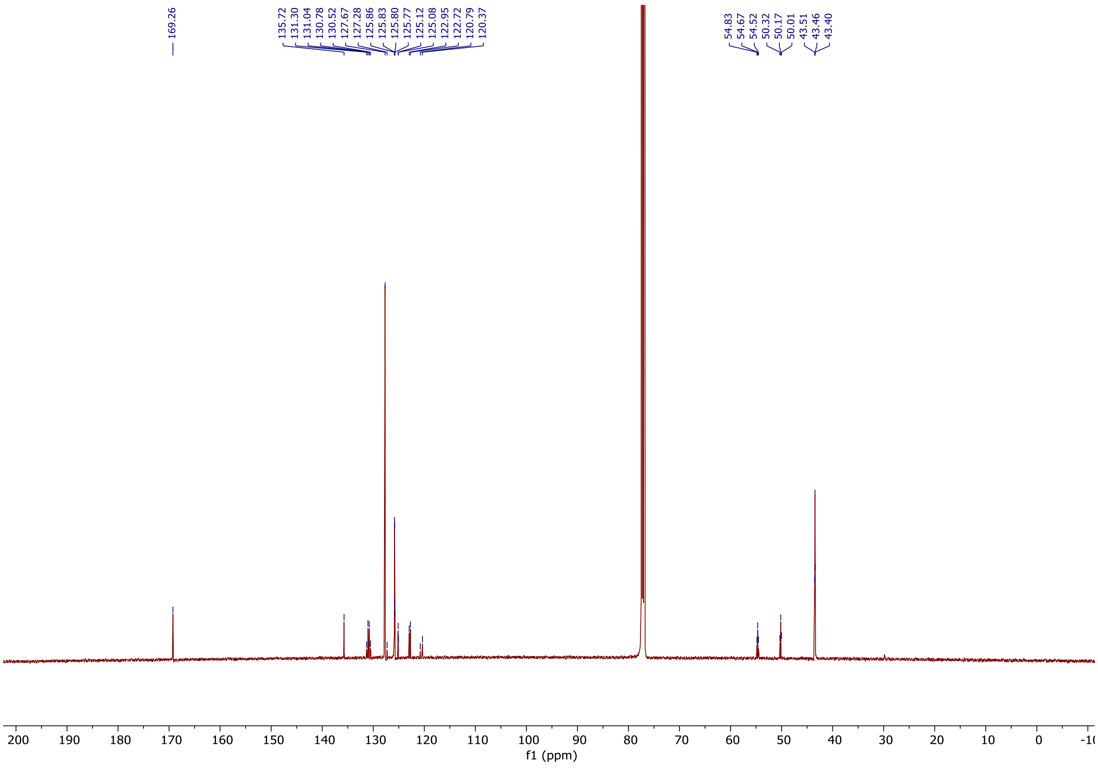

^{1}H COSY (500 MHz, Chloroform-*d*)

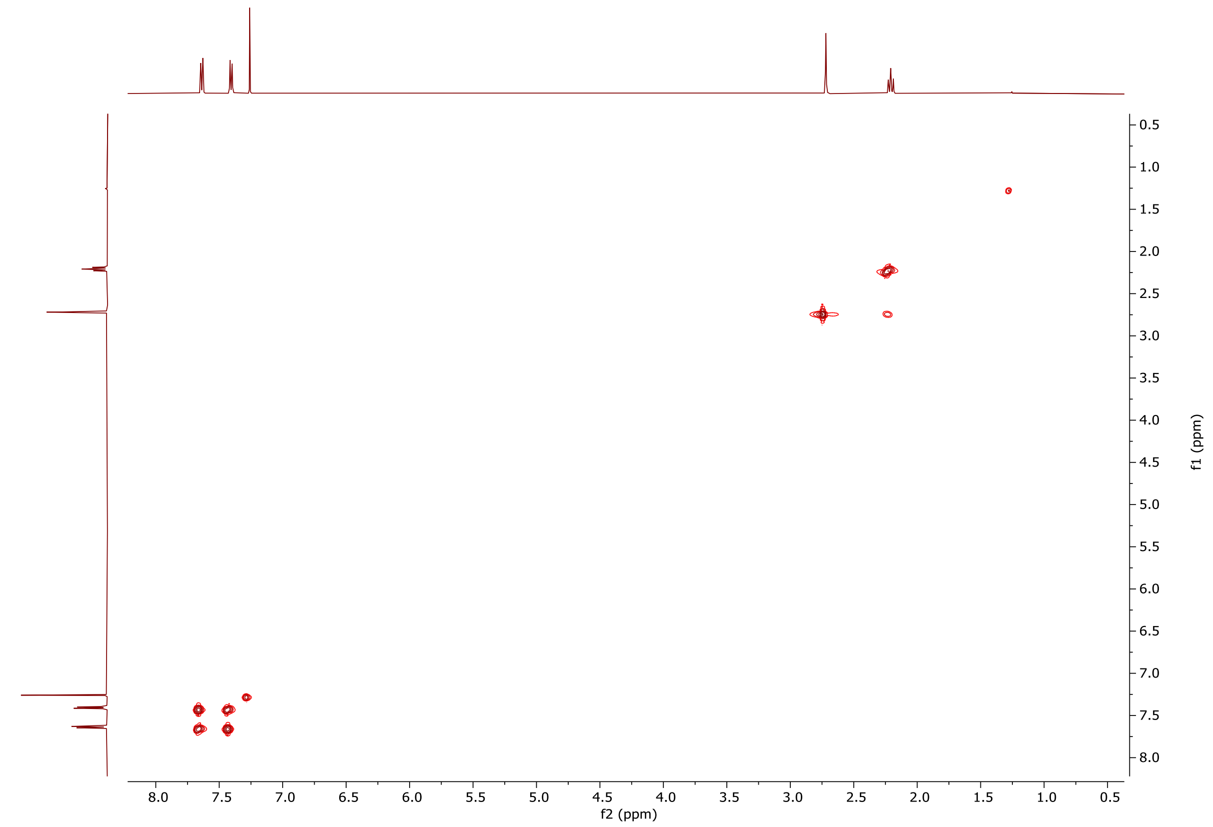

^{1}H/^{13}C HSQC (500/126 MHz, Chloroform-*d*)

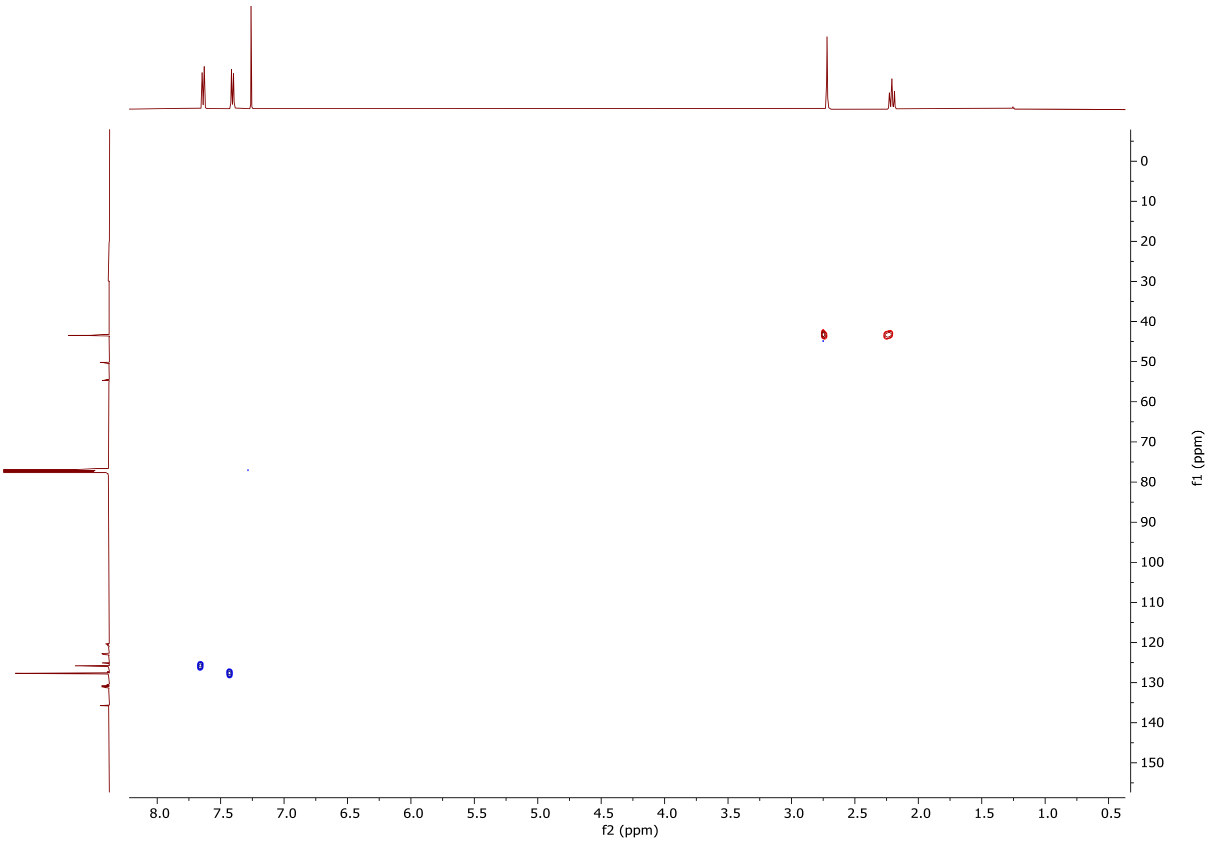

$^1H/^{13}C$ HMBC (500/126 MHz, Chloroform-*d*)

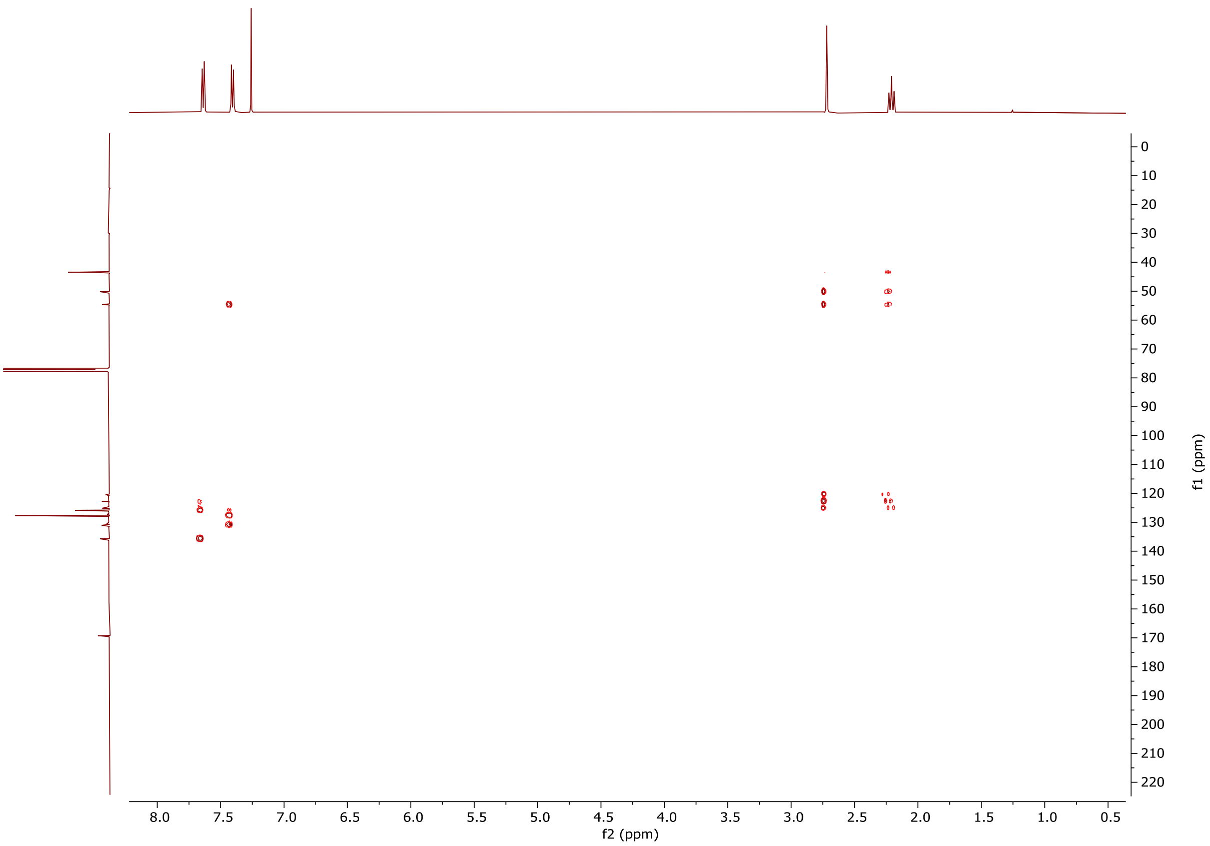

^{19}F NMR (470 MHz, Chloroform-*d*)

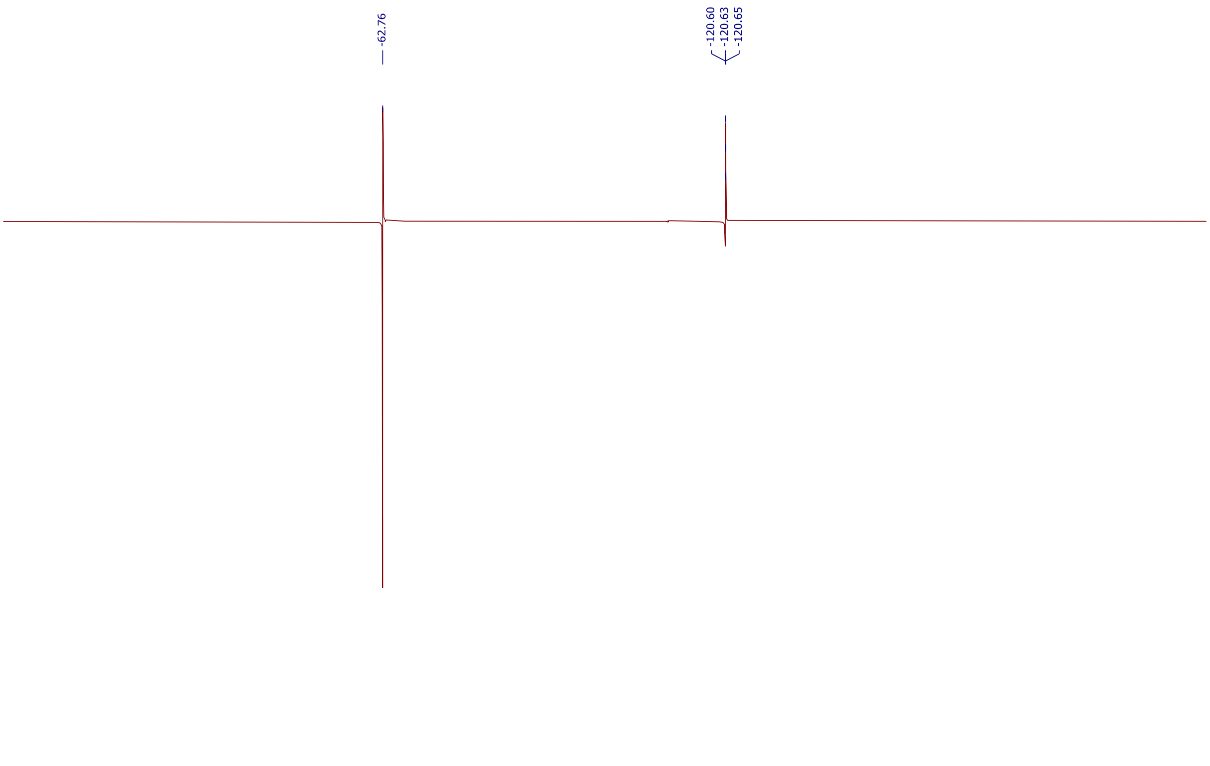

Supplement: SC-012-D1SC01836A-s001 [file SC-012-D1SC01836A-s001.pdf]
